# Uncovering the functional diversity of rare CRISPR-Cas systems with deep terascale clustering

**DOI:** 10.1126/science.adi1910

**Published:** 2023-11-23

**Authors:** Han Altae-Tran, Soumya Kannan, Anthony J. Suberski, Kepler S. Mears, F. Esra Demircioglu, Lukas Moeller, Selin Kocalar, Rachel Oshiro, Kira S. Makarova, Rhiannon K. Macrae, Eugene V. Koonin, Feng Zhang

**Affiliations:** 1 Howard Hughes Medical Institute; Cambridge, MA 02139, USA.; 2 Broad Institute of MIT and Harvard; Cambridge, MA 02142, USA.; 3 McGovern Institute for Brain Research at MIT; Cambridge, MA 02139, USA.; 4 Department of Brain and Cognitive Science, Massachusetts Institute of Technology; Cambridge, MA 02139, USA.; 5 Department of Biological Engineering, Massachusetts Institute of Technology; Cambridge, MA 02139, USA.; 6 National Center for Biotechnology Information, National Library of Medicine, National Institutes of Health; Bethesda, MD 20894, USA.

## Abstract

Microbial systems underpin many biotechnologies, including CRISPR, but the exponential growth of sequence databases makes it difficult to find new systems. Here we describe Fast Locality-Sensitive Hashing-based clustering algorithm (FLSHclust), which performs deep clustering on massive datasets in linearithmic time. We incorporated FLSHclust into a CRISPR discovery pipeline and identified 188 previously unreported CRISPR-linked gene modules, revealing many additional biochemical functions coupled to adaptive immunity. We experimentally characterized 3 HNH nuclease-containing CRISPR systems, including the first type IV system with a specified interference mechanism, and engineered them for genome editing. We also identified and characterized a candidate type VII system, which we show acts on RNA. This work opens new avenues for harnessing CRISPR and broader exploration of the vast functional diversity of microbial proteins.

## One-Sentence Summary

A clustering algorithm, FLSHclust, was developed and applied to discover 188 previously unreported CRISPR-linked gene modules.

## Main Text:

**Fig. 1.**
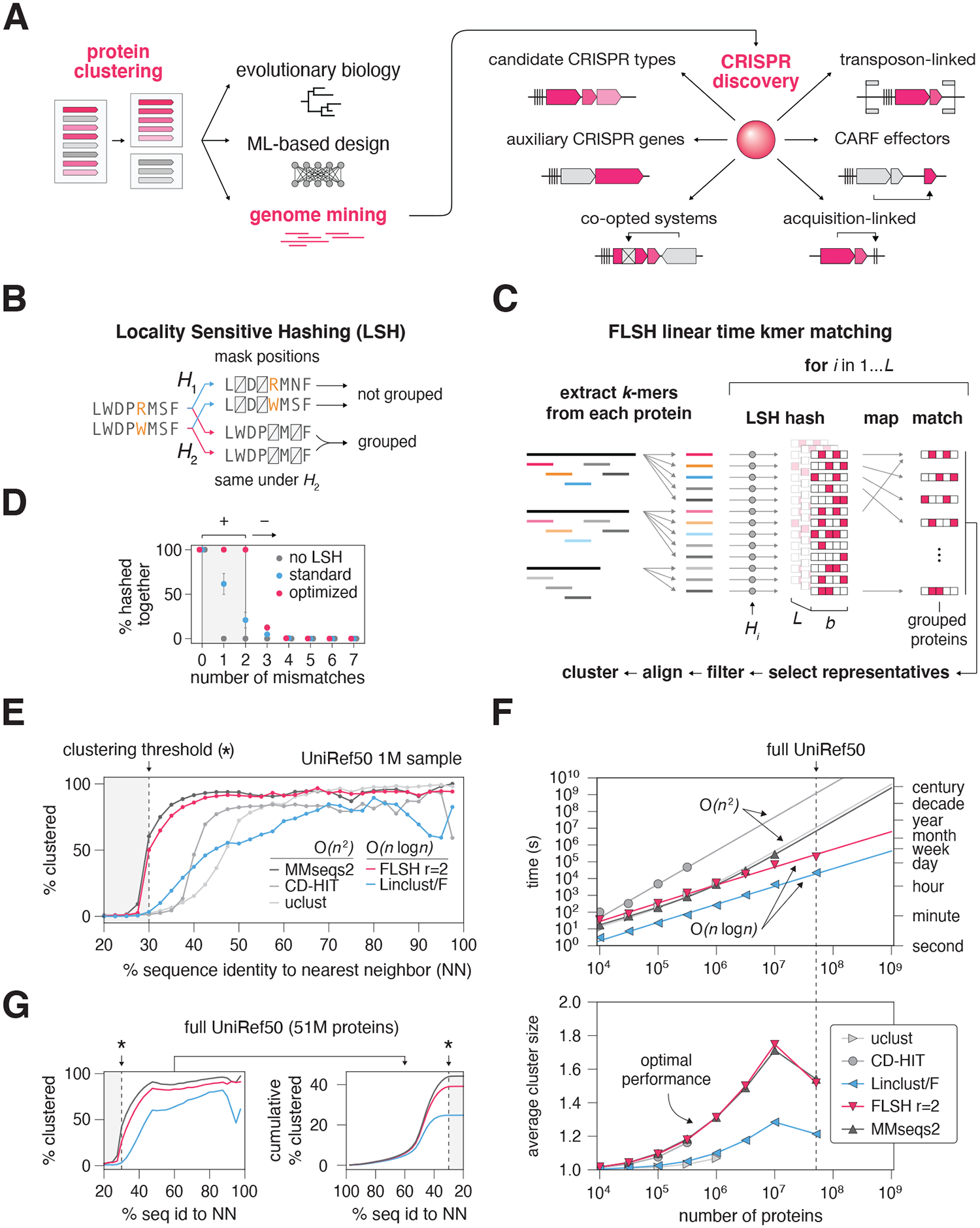
Design and implementation of FLSHclust (**A**) Schematic of applications of protein clustering in biology and bioinformatic. Archetypal examples of biological systems that could be found with genome mining approaches for CRISPR are shown, including CRISPR-Associated Rossmann Fold (CARF) proteins and transposon-linked genes. **(B)** Conceptual schematic of locality-sensitive hashing. In contrast to standard hash-based bucketing, locality-sensitive hashing allows similar, non-identical objects to be bucketed together. The specific family of hash functions shown in the example is randomized positional masking (bit masking) on sequences. This family functions by dropping specific positions in each kmer, where the positions are randomly selected per hash function. **(C)** Schematic of the steps of FLSHclust involving locality-sensitive hashing. First, all kmers are extracted from each protein. Then for each hash function, the hash function is applied to all kmers and kmers with the same hash value are grouped and then processed independently to determine which sequences will be aligned in the next step. **(D)** Optimized hash functions with no false negatives as calculated using Markov Chain Monte Carlo compared to standard randomized hash functions from the same family. Probability of bucketing two kmers together in one of the *L* hash tables as a function of the number of mismatches between the kmers is shown. The parameters used for the LSH family functions are *L*=24 hash functions, kmer length *k*=12, with 3 positions dropped per hash function. For the optimized hash functions, the target number of tolerated mismatches is 2, such that the family has no false negatives in identifying matches between kmers with up to 2 mismatch positions. **(E)** Clustering performance across different algorithms for clustering a 1M protein subset of the UniRef50 database. Linclust/F refers to linclust using 8001 kmers per protein, as opposed to the default of 20. FLSH refers to FLSHclust, with r=2 indicating two tolerated mismatches. Clustering performance shows the fraction of proteins that are grouped into a cluster of size 2 or more as a function of similarity to their nearest neighbors. **(F)** Scaling comparison of various clustering algorithms and FLSHclust against subsets of UniRef50. Above: compute time on 2 nodes each with 64CPUs. Below, average cluster size as a function of number of input sequences. *MMseqs2 on the full UniRef50 dataset required substantially more compute resources to complete within a week and thus was not included in the timing analysis. Theoretical scaling shown with big O notation. **(G)** Comparison of clustering algorithms as in **E)** except on the full UniRef50 dataset. Additionally, a cumulative distribution across all input proteins is shown. Asterisk refers to the clustering threshold of 30%.

Discovery of enzymes and natural biochemical systems advances molecular evolution studies, shines new light on biological processes, and provides a starting point for the development of molecular technologies. Over the past few decades, an enormous variety of protein families and functional systems were discovered through systematic mining of the rapidly growing nucleic acid and protein sequence databases. Many of these efforts employ protein clustering to group similar sequences within large datasets (Fig. 1A). The output of these algorithms can then be used to inform efforts aimed at deep learning on protein sequences, 3D protein structure prediction, and genome mining. One prime example of the latter is the discovery of novel CRISPR systems, which has led to the development of transformative biotechnologies and therapeutic approaches (1–4).

CRISPR systems are microbial RNA-guided adaptive immune systems (5). They are composed of a CRISPR array, which encodes the CRISPR (cr)RNAs that give rise to the guides, an adaptation module, which integrates new spacers into the CRISPR array, and an interference module that consists of effector components guided by the crRNAs to matching targets, which are then cleaved. CRISPR effectors can be either complexes of Cas proteins (e.g., Cascade) in Class 1 CRISPR systems or single, multidomain proteins (e.g., Cas9, Cas12, Cas13) in Class 2 CRISPR systems (6). This inherent modularity and programmability of CRISPR systems has been capitalized on to develop a suite of RNA-guided molecular technologies, starting with Cas9-mediated genome editing (1).

This toolbox was expanded through computational searches that uncovered many new CRISPR systems (3, 7–9). However, existing methods rely on algorithms that have quadratic runtime, such as all-against-all comparisons and protein clustering (9), which quickly become impractical for mining exponentially growing datasets containing billions of proteins (11). Linear scaling clustering methods like LinClust (12) can address some of these issues, but produce small clusters of highly similar sequences that limit the ability to study deep evolutionary relationships. Protein domain profiles, such as PFAM, can be used to identify broad abundant associations (13), but group remote homologs, leading to spurious associations while missing rare ones (14).

To address these limitations and take advantage of the explosive increase of the known structural and functional diversity of proteins, we developed FLSHclust (pronounced “flash clust”), a parallelized, deep clustering algorithm with linearithmic scaling, *O*(*N* log*N*). FLSHclust can handle billions of proteins, enabling efficient analysis of the vast, exponentially growing sequence databases. We apply FLSHclust to identify previously uncharacterized CRISPR systems, including a candidate type VII CRISPR system, generating a catalog of RNA-guided proteins that expand our understanding of the biology and evolution of these systems and provide a starting point for the development of new biotechnologies.

## Fast locality-sensitive hashing allows for deep clustering of all known proteins at terabyte scale

To address the limitation of quadratic time complexity inherent to all-to-all comparisons, we sought to use locality sensitive hashing (LSH), a technique that efficiently groups similar, non-identical objects in linear time at the cost of false positives and negatives (Fig. 1B) (14). Using this approach, we developed Fast LSH-based clustering (FLSHclust) (Fig. 1C, Fig. S1A).

FLSHclust first maps each protein to a reduced amino acid alphabet, then extracts all kmers of length *k* (Fig. 1C). An optimal LSH family with no false negatives (15) is generated using Markov Chain Monte Carlo, and for each hash function, all hashed kmers are grouped into buckets containing similar kmers (Fig. 1D). Two representative sequences are then selected per bucket, and for all sequences in the bucket, a graph edge is formed if an alignment between the sequence and each of the representatives satisfies the clustering criteria. The resulting graph is simplified using a graph degree-aware transformation that breaks long chains. Then, a community detection is applied to form groups of sequences, which are then clustered using greedy clustering to produce a final set of clusters (Fig. S1A for schematic of complete algorithm, Fig. S1B for pseudocode, see Supplementary Text for additional discussion).

We benchmarked the performance and scalability of FLSHclust against several commonly used algorithms, namely MMSeqs2, uclust, CD-HIT, and LinClust (11, 15–17). First, all algorithms were assessed on their ability to cluster 1 million proteins from UniRef50 at 30% sequence identity (Fig. 1E) (11, 15–18). FLSHclust’s clustering performance (with 2 tolerated kmer mismatches) approached that of MMSeqs2, the top-performing quadratic scaling algorithm (Fig. 1E). Moreover, when considering each set of proteins with a given distance to its nearest neighbor (Fig. 1E), FLSHclust succeeded in clustering a higher proportion of these proteins as compared to LinClust, another algorithm with linearithmic scaling (Fig. 1E). We additionally found that FLSHclust produces high inter-cluster distances comparable to MMSeqs2, demonstrating high quality cluster representatives that tend to be no more than 30% sequence identity from one another (Fig S2A).

To characterize scalability, we benchmarked all algorithms on a panel of UniRef50 subsets of different sizes using a 2-node computer grid with 64 CPUs, 416 GB of memory, and 2 TB of SSD storage per node. FLSHclust achieved nearly the same average cluster size as MMSeqs2 at all tested dataset sizes, yet exhibits linearithmic scaling in practice, allowing it to run faster than all tested quadratic scaling algorithms on a suitably large dataset, such as 10 million proteins (Fig. 1F). Moreover, as the size of the input dataset increases, the number of clusters produced by FLSHclust also increases, with the cluster size exhibiting a power law distribution, similar to MMSeqs2 (Fig. S2B). We then compared the clustering performance of FLSHclust, Linclust, and MMSeqs2 (which required a large server to complete) on the full UniRef50 dataset containing 51 million proteins (Fig. 1G) and found that FLSHclust clustered 58% more proteins as compared to Linclust and only 12% fewer compared to MMSeqs2, suggesting that FLSHclust can achieve a similar clustering performance to MMSeqs2 even on large datasets. Lastly, we compared FLSHclust to other clustering algorithms against various clustering thresholds and found that FLSHclust can cluster proteins down to 25% sequence identity with corresponding inter-representative distances (Fig. S2C–D).

Overall, FLSHclust is fully parallelizable and can readily scale to large computing infrastructures while exhibiting high computational efficiency (Fig. S2E–F). Our FLSHclust implementation is also resilient to computational node or network failures due to the underlying fault-tolerant Apache Spark framework, allowing FLSHclust to use thousands of CPUs seamlessly (19). The ability of FLSHclust to comprehensively cluster sequences down to 25% sequence identity while scaling nearly linearly with the number of proteins allows it to complement other clustering algorithms by efficiently operating with datasets exceeding millions or billions of proteins.

## Discovery of previously unreported, rare CRISPR systems

**Fig. 2.**
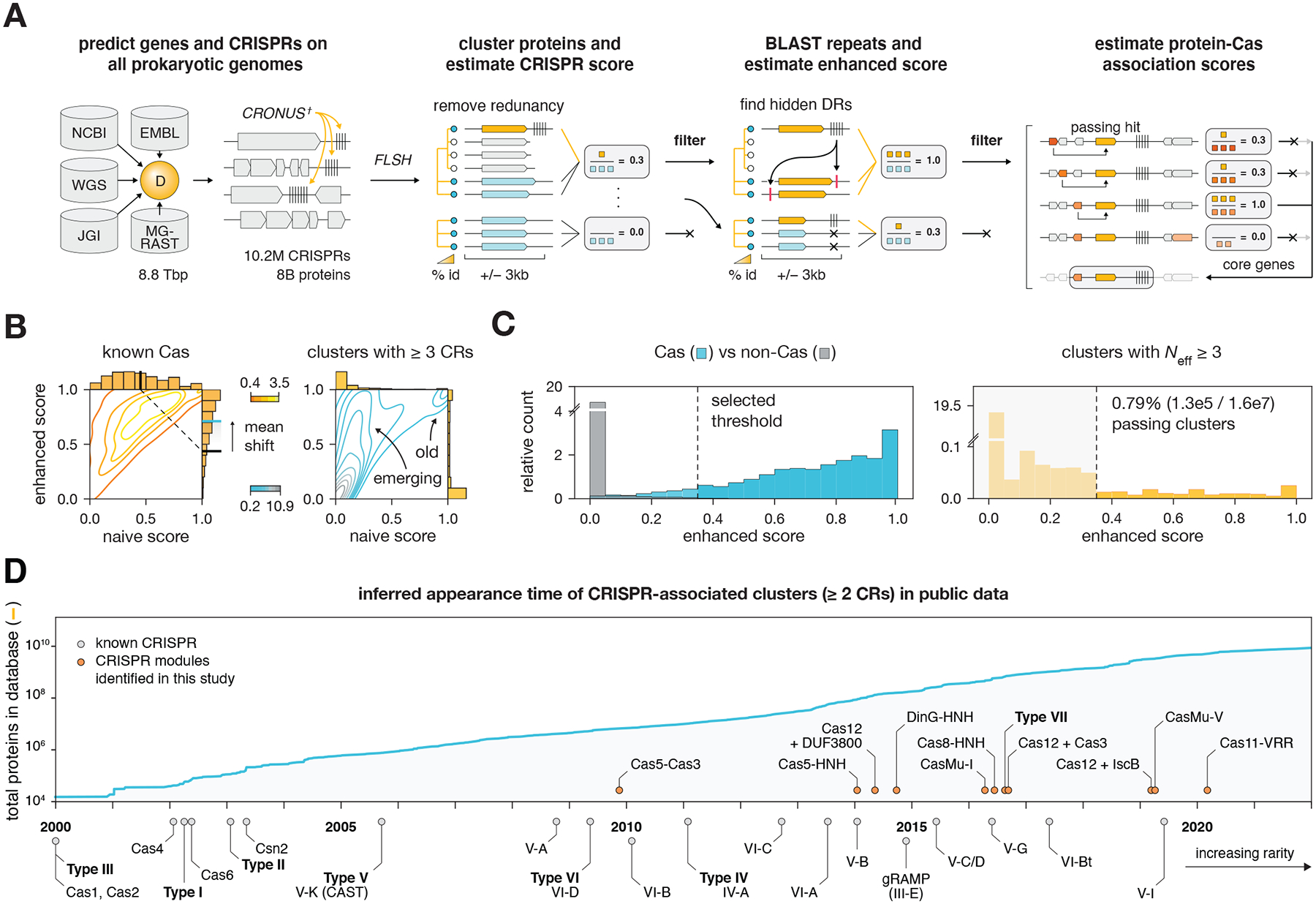
Discovery of hundreds of rare novel CRISPR systems with a sensitive, scalable CRISPR association pipeline. (**A**) Schematic of CRISPR discovery pipeline using no all-to-all comparisons. (**B**) Comparison of naive and enhanced CRISPR association scores for identifying CRISPR-associated clusters. Left: known Cas genes; right: all clusters. (**C**) Selection of CRISPR-associated clusters. Left: relative count of Cas (blue) vs non-Cas (gray) clusters as a function of enhanced CRISPR association score. An empirical threshold of 0.35 enhanced score was selected for identifying CRISPR-associated clusters. Right: relative count of all clusters with *N*_*eff*_ ≥ 3. Dotted line demarcates the 0.35 enhanced score cutoff. ~130,000 clusters with an enhanced score ≥ 0.35 passed for further analysis. *N* CRs: number of non-redundant loci with CRISPR arrays. **(D)** Line graph: Number of proteins over time in the complete dataset including all projects from public data (JGI, NCBI, WGS, and EMBL, excluding MG-RAST). Bottom: Back-calculated times at which CRISPR-associated, non-singleton protein clusters appeared in the public dataset for selected systems. Cluster assignments are fixed across time using the 30% sequence identity clustering from FLSHclust. The appearance time of a *cluster* is the earliest time at which a minimum of 2 non-redundant, CRISPR-associated proteins from the cluster are present in the public dataset. The appearance time of a *system* (e.g., Cas9, etc.) is the earliest appearance time across all related clusters. For multi-gene systems, a signature gene was used to represent the entire system (Type I: Cas7, Type III: Csm3, Type IV: Csf2). The inferred appearance time values is an upper bound for the true CRISPR-associated cluster appearance time in the dataset.

We applied FLSHclust to discover rare CRISPR systems. CRISPR systems have diverse architectures and mechanisms and are divided into 6 types and 33 subtypes (19). To find additional CRISPR systems, we developed a sensitive CRISPR discovery pipeline that combines FLSHclust and CRISPR repeat finders to identify deep clusters of proteins stably associated with CRISPR arrays (Fig. 2A). We curated a database of 8.8 Tbp (tera-base pairs) of prokaryotic genomic and metagenomic contigs (excluding metagenomic contigs < 2 kbp in length) from NCBI, WGS, and JGI (Fig. 2A). Coding sequences were predicted using Genemark (20), and CRISPR arrays were predicted using previously developed CRISPR finders (21–24) and CRONUS, a tool we developed to detect smaller CRISPR arrays that include imperfect repeats as well as other repeat arrays with hypervariable spacers (Materials and Methods, Fig. S3 for benchmarking). The final database contained 8 billion proteins and 10.2 million CRISPR arrays. Using FLSHclust, we iteratively clustered all proteins, resulting in 1.3 billion redundancy-reduced (90% sequence identity) clusters and 499.9 million deep (30% sequence identity) clusters. In contrast to clustering at 50% identity, which produced 646.4 million clusters, clustering at 30% with FLSHclust produced fewer but larger clusters (average cluster size of 2.0 vs 2.5 non-redundant proteins respectively) making them more conducive for estimating evolutionary statistics.

To identify genes stably associated with CRISPR arrays, we computed a CRISPR association score (naive score) for each 30% cluster by calculating the weighted fraction of non-redundant proteins encoded in an operon within 3 kbp of a CRISPR array over the effective sample size of the cluster, *N*_*eff*_, which adjusts for contig truncations that occur in metagenomic data (Materials and Methods). To capture emerging or degrading CRISPR systems, which often only contain a single direct repeat (DR) or highly diverged DRs (25), for each CRISPR-associated cluster, we selected a representative DR and searched its sequence against all other non-redundant loci in the cluster (26). The identified divergent DR sequences were used to compute an enhanced CRISPR-association score. Finally, to expand our search to find genomically distant components of CRISPR systems, all proteins considered to be CRISPR-associated were used as baits for identifying additional associated proteins (Fig. 2A).

To evaluate the performance of this CRISPR search pipeline, we compared the naive and enhanced CRISPR scores of known CRISPR-associated (*cas*) genes and found that the mean naive score of *cas* genes was 0.44, whereas the enhanced score increased to 0.72 (Fig. 2B), highlighting the importance of identifying divergent DRs and mini CRISPR arrays. Using the enhanced score, we compared *cas* and non-*cas* genes and empirically determined a cutoff of 0.35, which included most known *cas* genes while removing most non-*cas* genes (Fig. 2C). We then applied this filter to all protein clusters with an effective sample size *N*_*eff*_ ≥ 3, resulting in ~130,000 clusters with associations to CRISPR-like repeats (out of 16 million total clusters with *N*_*eff*_ ≥ 3). After manual curation, we identified 188 previously unreported CRISPR-linked systems, many of which included proteins or domains not previously linked to CRISPRs. All systems identified in the complete analysis, including those previously known, are provided in the supplement (Table S1, sequences for manually curated set in Data S2–3, protein-protein associations in Data S4; see Table S2 for equivalences of Cas legacy names). Using only the naive score with 50% clusters, we recovered 51 fewer systems, with an additional 12 losses if only CRT (22) was used for identifying CRISPR arrays, underscoring the sensitivity of the complete pipeline (Table S3).

The abundance and distribution of different CRISPR systems is uneven across sequenced bacterial and archaeal genomes (6, 28, 29). To gauge how the increasing diversity of sequencing data correlates with the CRISPR-Cas diversity detectable with our pipeline, we back-calculated the time at which clusters (with a minimum of two non-redundant CRISPR-associated loci) appeared in the public dataset for various CRISPR-Cas subtypes of note (Fig. 2D, Data S1). These calculations track with the abundance of *cas* genes, highlighting the importance of diverse environmental sampling for discovering biochemical, mechanistic, and functional diversity of CRISPR systems. Notably, the systems that we identified here are rare and appeared in the dataset only recently, during the past decade. These include various Class 1-derived systems, such as a type IV-derived system containing a DinG-HNH fusion effector, type I-derived systems containing Cas8-HNH and Cas5-HNH fusion effectors, candidate type VII system, and CRISPR-linked transposons, some of which we experimentally characterized.

## DinG-HNH is a Type IV-A variant with directional, dsDNA nuclease activity

**Fig. 3.**
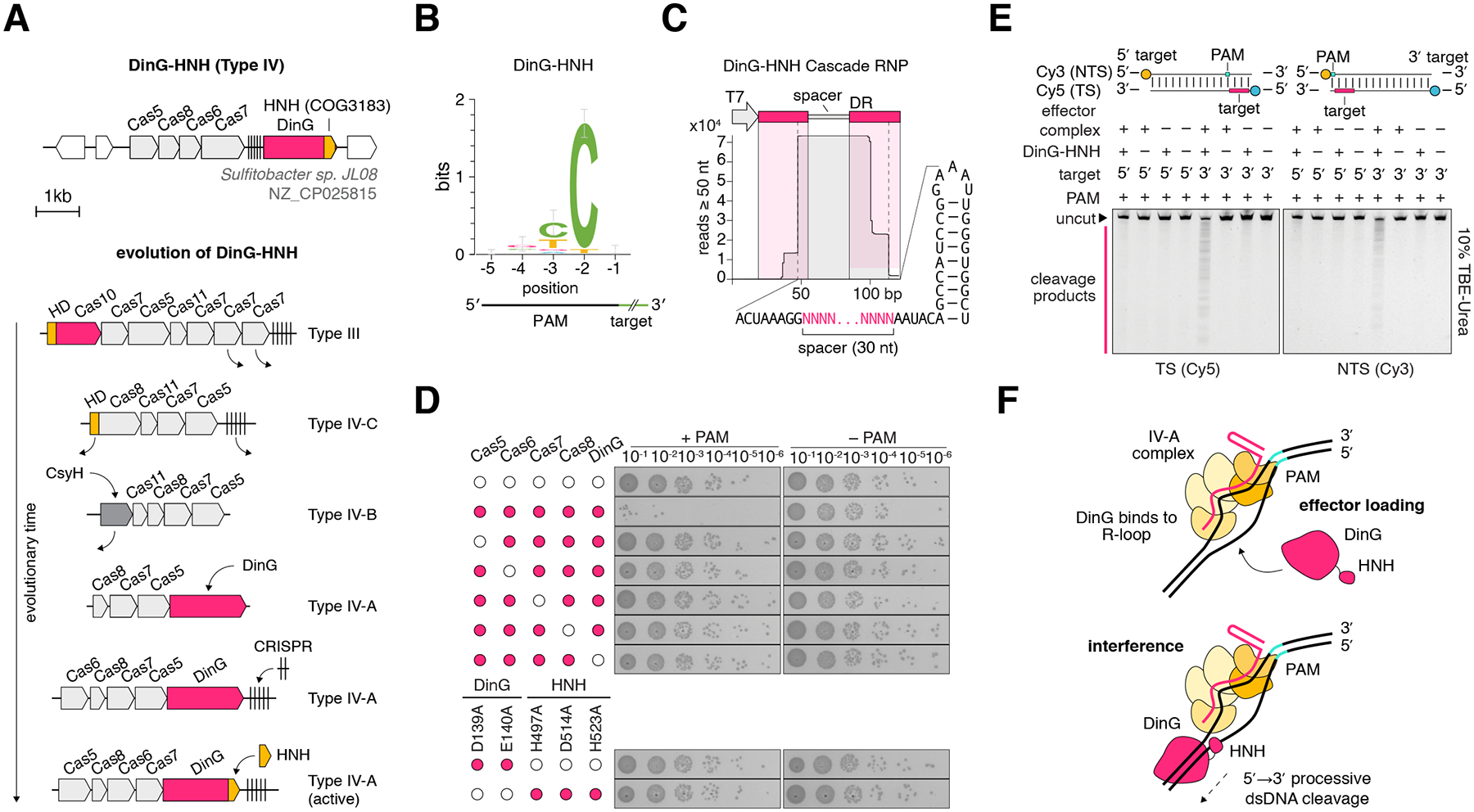
Type IV-A CRISPR systems perform directional dsDNA unwinding and strand-specific cleavage. (**A**) Locus diagram of the experimentally studied DinG-HNH system from Sulfitobacter sp. JL08. (**B**) Sequence logo for the PAM of DinG-HNH as determined by a plasmid depletion assay in *E. coli*. (**C**) Small RNA-seq of DinG-HNH effector complex RNP pulldown. (**D**) *E. coli* transformation assays with DinG-HNH and associated effector complex genes and cognate targets with or without the PAM identified in (B). (**E**) *In vitro* reconstituted DinG-HNH and associated effector complex RNP cleavage of linear dsDNA targets. Targets either contain the cognate target site at the 5′ or 3′ end of the target strand (TS) as indicated. Only targets on the 3′ end of the TS are cleaved. NTS: Non-target strand.

First, we examined the type IV-A variant with an HNH nuclease domain inserted at the C-terminal end of the CRISPR-associated DinG-like DEAD/DEAH-box helicase (Fig. 3A) (30–32). Type IV systems appear to have evolved from active type III systems (30–32) but are poorly characterized, with no documented mechanism of action (33). The insertion of the HNH domain into the DinG protein could reflect an evolutionary trajectory from a type IV system that lost the capacity to cleave DNA back to a system fully capable of adaptive immunity and interference (Fig. 3A) (34, 35). We hypothesized that the HNH domain mediates target cleavage via an unwinding and cleavage mechanism analogous to the processive target cleavage by Cas3 (36). To test this, we heterologously expressed the DinG-HNH system in *E. coli* along with a CRISPR array encoding a reprogrammed spacer sequence targeting a protospacer adjacent to an 8N randomized library (36). We observed depletion of 5′ YCN protospacer-adjacent motifs (PAMs) (Fig. 3B), indicating that the system is capable of programmable, PAM-dependent RNA-guided plasmid interference activity. Small RNA sequencing of the heterologously expressed operon and associated CRISPR array revealed processed crRNAs containing a 30-nt spacer (Fig. 3C).

To validate the observed activity, we performed a plasmid transformation efficiency assay and compared transformation efficiency of a target plasmid in cells containing the complete operon to those containing an empty vector control. We found that transformation efficiency decreased by 3 orders of magnitude when both the complete operon and correct PAM were present (Fig. 3D). Through systematic deletion of each protein, we found that all five components of the effector complex were required for interference activity (Fig. 3D). Furthermore, mutation of the conserved negatively charged residues of the Walker B motif (D139, E140) and the catalytic triad of the HNH domain (H497, D514, H523) in the *dinG* gene abolished activity, implying that both ATP hydrolysis and HNH nuclease activity are required for interference (Fig. 3D) (37).

To characterize the biochemical mechanism of the observed interference activity, we recombinantly expressed and affinity purified both the effector ribonucleoprotein (RNP) complex and DinG-HNH protein (Fig. S4A). When all components were combined with a linear dsDNA target, we observed a ladder of cleavage products on a denaturing gel (Fig. S4B), indicating movement of the DinG helicase along the target DNA. To test if this movement was directional, we constructed two linear dsDNAs with the target site placed near either the 5′ or 3′ end of the target strand (Fig. 3E, S4D). We observed activity only when the target site was positioned close to the 3′ end of the target strand, suggesting DinG loads to the non-target strand (NTS) within the R loop and moves in the 5′→3′ direction along the NTS while continuously cleaving both the target and non-target strands (Fig. 3F) (37, 38).

Together, these results suggest that the role of the DinG helicase-nuclease in these type IV systems is analogous to that of the Cas3 effector protein in type I CRISPR systems, whereby a helicase and a nuclease act in conjunction to unwind and shred the target. However, the helicase moieties of the DinG-HNH and Cas3 are only distantly related whereas the nucleases are unrelated, indicating that this mechanism evolved twice independently.

## Type I Cascade components are functionalized with HNH domains for precise dsDNA cleavage

**Fig. 4.**
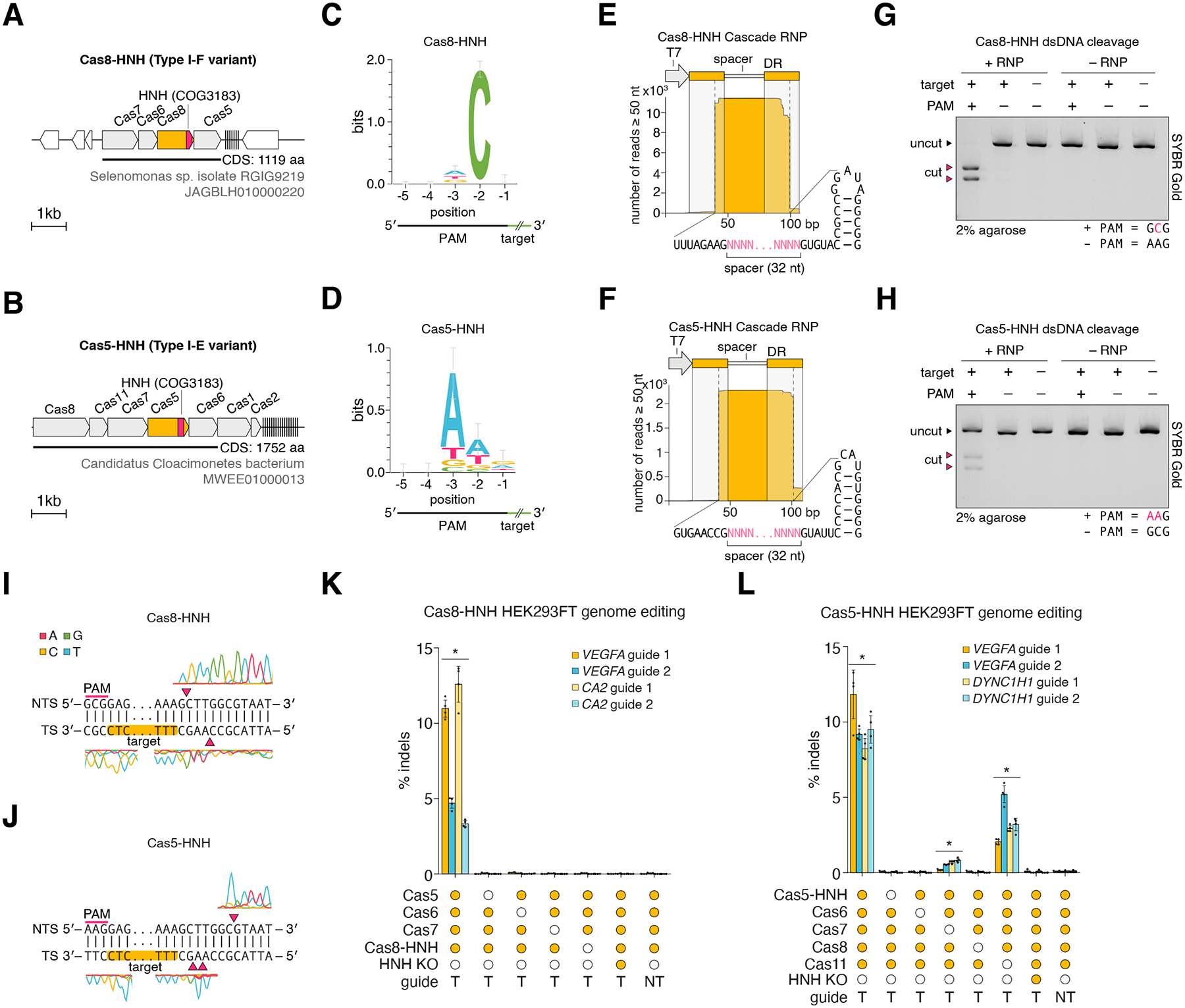
HNH-functionalized Cascade subunits perform precise, RNA-guided dsDNA cleavage. (**A**) Locus diagram of the experimentally studied Cas8-HNH system from Selenomonas sp. isolate RGIG9219. (**B**) Locus diagram of the experimentally studied Cas5-HNH system from Candidatus Cloacimonetes bacterium. (**C**) Sequence logo for the PAM of Cas8-HNH as determined by a plasmid depletion assay in *E. coli*. (**D**) Sequence logo for the PAM of Cas5-HNH as determined by a plasmid depletion assay in *E. coli*. (**E**) Small RNA-seq of Cas8-HNH Cascade RNP pulldown. **(F)** Small RNA-seq of Cas5-HNH Cascade RNP pulldown. **(G)** *In vitro* reconstituted Cas8-HNH Cascade RNP cleavage of linear dsDNA targets, in the presence or absence of a cognate target and/or PAM. **(H)** *In vitro* reconstituted Cas5-HNH Cascade RNP cleavage of linear dsDNA targets, in the presence or absence of a cognate target and/or PAM. **(I)** Sanger sequencing of cleavage products generated by Cas8-HNH. **(J)** Sanger sequencing of cleavage products generated by Cas5-HNH. In both (I) and (J), the polymerase used exhibits non-templated incorporation of a terminal adenine, which results in a thymidine appearing at the end of the trace. **(M)** HEK293FT genome editing at 4 genomic loci by Cas8-HNH in the presence or absence of each Cascade subunit or cognate guideRNA, or with alanine mutation of HNH domain catalytic residues. Error bars denote SD. *P < 0.05 relative to non-targeting (NT) guide condition. T: Targeting guide. **(N)** HEK293FT genome editing at 4 genomic loci by Cas5-HNH in the presence or absence of each Cascade subunit or cognate guideRNA, or with alanine mutation of HNH domain catalytic residues. Error bars denote SD. *P < 0.05 relative to non-targeting (NT) guide condition. T: Targeting guide.

We also identified two novel variants of type I CRISPR systems containing an HNH nuclease domain inserted into one of the Cascade backbone components, either *cas8* or *cas5,* but most examples of which lack *cas3* (Fig. 4A, B). The Cas8-HNH system consists of four genes and is most closely related to type I-F1 CRISPR systems, whereas the Cas5-HNH system consists of five genes and is most closely related to type I-E CRISPR systems. In some cases, the *cas8* was additionally fused to *cas11*, and in other rare cases, remnants or truncations of *cas3* appeared in the vicinity, suggesting *cas3* progressively disappeared from the system (Data S2). Based on the absence of the *cas3* helicase/nuclease gene along with the previously unreported association of an HNH domain, we hypothesized that both these systems might enable precise RNA-guided double-stranded DNA (dsDNA) cleavage, in contrast to the processive degradation activity exhibited by Cas3 in canonical Type I systems (39).

To test this, we performed a PAM discovery assay in *E. coli* and observed depletion of specific PAMs for both systems (Fig. 4C, D), suggesting that both are capable of RNA-guided interference activity. Small RNA sequencing of the recombinantly purified Cascade RNPs showed that Cascade binds to crRNAs in each system, both containing 32-nt spacers (Fig. 4E, F) (39).

Next, we confirmed the ability of the Cas8-HNH and Cas5-HNH Cascade RNPs to cleave dsDNA in a precise, PAM-dependent manner (Fig. 4G, H, S5). Sequencing of the cleavage products for each system showed that Cas8-HNH cleaves the TS and NTS 5 bp and 2 bp downstream of the protospacer, respectively, on the PAM-distal end of the target, generating 5′ overhangs (Fig. 4I). By contrast, Cas5-HNH cleaves the TS and NTS 3–4 bp and 8 bp downstream of the protospacer, respectively, on the PAM-distal end, generating 3′ overhangs (Fig. 4J).

Given that HNH domains have been observed to cleave only a single strand in targeted dsDNA (25, 40), we tested both systems for ssDNA cleavage activity. We observed that both the Cas8-HNH (Fig. S5C) and the Cas5-HNH systems (Fig. S5D) can cleave ssDNA in a PAM-independent manner. We additionally found that the Cas5-HNH system, but not the Cas8-HNH system, exhibited collateral cleavage of ssDNA substrates stimulated by dsDNA and ssDNA targets in a PAM-dependent and PAM-independent manner, respectively (Fig. S5E, F). This is the first reported observation of collateral activity in a type I CRISPR-Cas system, suggesting convergent evolution of this mechanism.

Finally, we tested if Cas8-HNH and Cas5-HNH can programmably generate short insertions/deletions (indels) in mammalian cells. We found that both systems are capable of inducing indels with varying efficiencies up to ~13% (Fig. 4K, L, Table S4). For Cas8-HNH, all protein subunits were required for activity (Fig. 4K). For the Cas5-HNH system, the Cas11/Cse2 subunit was dispensable for indel formation, but its deletion resulted in reduced activity (up to ~6%), while deleting Cas7 resulted in minimal activity (up to ~1%). Deleting any of the other components ablated activity (Fig. 4L). Inactivation of the catalytic residues of the HNH domain in each system also abolished activity, demonstrating that the HNH domain mediates target cleavage in both systems (Fig. 4K, L). To assess the genome-wide specificity of cleavage, we performed tagmentation-based tag integration site sequencing (41). For Cas8-HNH, we detected no off targets for the 4 tested guides, suggesting that this system is highly specific (Fig. S5G). The 3′ overhangs generated by Cas5-HNH cleavage were apparently not compatible with blunt-end ligation required for this assay.

## A candidate type VII CRISPR system is a precise RNA-guided RNA endonuclease complex containing a β-CASP nuclease

**Fig. 5.**
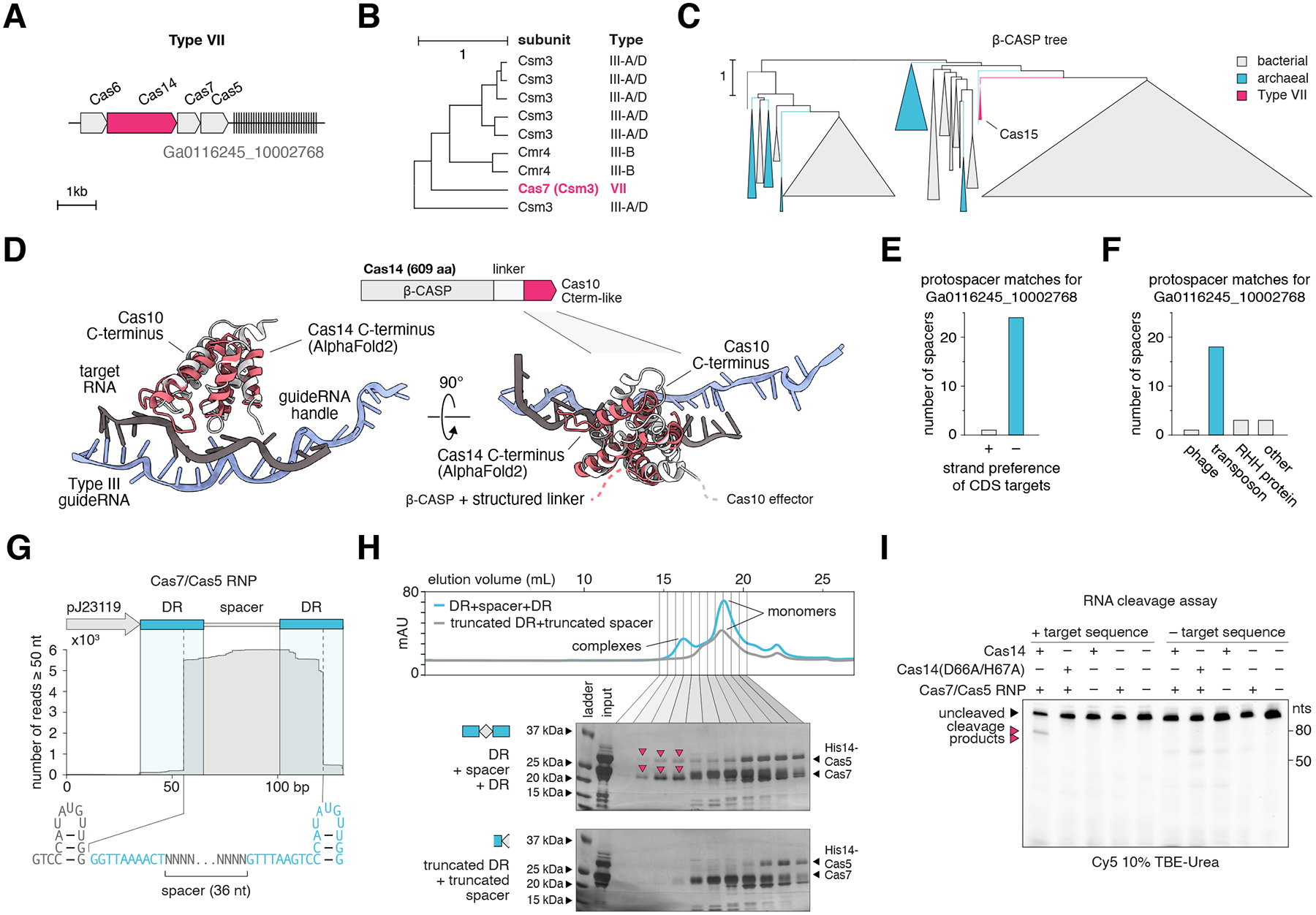
Candidate Type VII CRISPR system **(A)** Locus diagram of the experimentally studied candidate VII system. **(B)** UPGMA dendrogram from HHPred pairwise alignment scores of related Cas7s. **(C)** Phylogenetic tree (FastTree) of beta-CASP proteins from both bacteria and archaea, including the β-CASP proteins linked to the candidate type VII system, which form a distinct clade. **(D)** Top: diagram of the domain architecture of Cas14. Bottom: superposition of Cas14’s C-terminal domain with the Cas10’s C-terminal from PDB: 6NUD showing the Cas10 interface with the target RNA. Both share the 4 helix bundle found in Cas10 and Cas11 that are known to interact with the target strand. **(E)** CDS target strand preferences of the protospacer matches for the CRISPR array of the experimentally studied Type VII locus. **(F)** Targets of the protospacer matches for the CRISPR array of the experimentally studied type VII locus. **(G)** Small RNA-seq of Type VII Cas7-Cas5 RNP pulldown along with the DR sequences. **(H)** Size exclusion chromatography of the Cas7-Cas5 copurified with an expressed DR + spacer + DR or copurified with an expressed truncated DR + truncated spacer **(I)** *In vitro* reconstituted Cas14 and associated effector complex RNP cleavage of Cy5-labeled RNA targets, in the presence or absence of cognate target sequences. (D66A/H67A) represents mutation of key residues in the predicted catalytic Zn(II) binding pocket of Cas14 to alanine.

CRISPR systems evolve through modular replacement of Cas components and subdomains, as exemplified by the DinG-HNH, Cas8-HNH and Cas5-HNH systems characterized above. We further identified a distinct system present in diverse archaea containing a β-CASP nuclease domain protein. This protein is encoded in a predicted operon with Cas7 and Cas5 which, together, may form a minimal effector complex, and in some cases, a Cas6, which is involved in crRNA processing in other CRISPR-Cas systems (Fig. 5A, S6A, Table S5) (42). The Cas5 and the Cas7 of this system are distantly related to the type III-D Cas5 and Cas7 proteins, respectively, with an apparent inactivation of the Cas7 catalytic residues that are required for target RNA cleavage in type III systems (Fig. 5B, S6B–E, H, I).

The β-CASP domain is an ancient nuclease fold found in all domains of life that exhibits RNA endonuclease, 5′ to 3′ RNA exonuclease and/or DNA nuclease activities in various contexts (43). β-CASP domain proteins are involved in Non-Homologous End Joining DNA repair (NHEJ), V(D)J recombination, RNA surveillance, mRNA/rRNA maturation and RNA decay (44–48). Phylogenetic analysis of the β-CASP family supports the origin of the CRISPR-associated members from a distinct, well-defined clade (Fig. 5C, S6F). Structural modeling of the β-CASP protein with AlphaFold2 (49) shows two distinct domains, namely, the N-terminal β-CASP domain (Fig. S7, S6G), and a C-terminal adaptor domain with structural similarity (but no detectable sequence similarity) to the ~200 aa C-terminal domain of Cas10 (Fig. 5D), the large subunit of type III systems that is involved in target RNA interaction (50). Given its unique domain composition and association with CRISPR, we propose to designate the β-CASP domain protein of these systems Cas14, the next structurally distinct effector complex component after Cas12 and Cas13.

Searching for protospacer matches to the CRISPR spacers in these systems revealed a pronounced bias towards the antisense strand of matching target sequences (Fig. 5E, Data S5), suggesting that these systems target RNA. We further observed that spacers primarily target transposon genes, indicating that the system could defend against actively expressed transposons, unlike other known CRISPR types, which primarily target viruses or plasmids (Fig. 5F, S8).

We hypothesized that the Cas14-containing system carries out interference via the β-CASP nuclease domain, in contrast to the distantly related CRISPR subtype III-E, which also likely originated from subtype III-D but retains a Cas7-based interference mechanism (6, 51, 52). We further identified a new type III subtype that, like the Cas14-containing system, encompasses a single Cas7-like and a Cas5-like gene distinct from those of the Cas14-containing system (Fig. S9A). However, these systems also include a Cas10 with an active HD nuclease domain and an inactivated polymerase domain (Fig. S9B). Thus, this type III subtype is predicted to cleave target DNA but lacks the cyclic oligoA-dependent signaling pathway that is integrated in many other type III systems. These findings together point to convergent evolution of minimal effector complexes.

Purification and small RNA-seq of type VII Cas7/Cas5 RNP complexes showed that Cas7 and Cas5 form a complex that co-purifies with a processed crRNA containing both a 5′ and 3′ DR tag, similar to type I and IV systems (Fig. 5G) (52–54). The complex is stable only in the presence of the corresponding crRNA (Fig. 5H). To test cleavage activity, we separately purified Cas14 and mixed it with the purified Cas7-Cas5 RNP complex and labeled target RNA. We observed precise target RNA cleavage only in the presence of all proteins and the cognate target sequence (Fig. 5I, 10). Inactivation of key residues in the predicted Zn(II) binding pocket of the Cas14 β-CASP domain abolished cleavage activity (Fig. 5I). Together, these results suggest that Cas14 is the nuclease effector in these systems.

Given the distant relationship between the effector complex of the Cas14-containing system and those of other known CRISPR types, and the substitution of the effector nuclease with an unrelated nuclease, β-CASP, we propose that the Cas14-containing system is classified as type VII CRISPR-Cas (see Fig. S11 for further comparison across CRISPR types).

## Putative novel CRISPR variants and CRISPR-associated genes

**Fig. 6.**
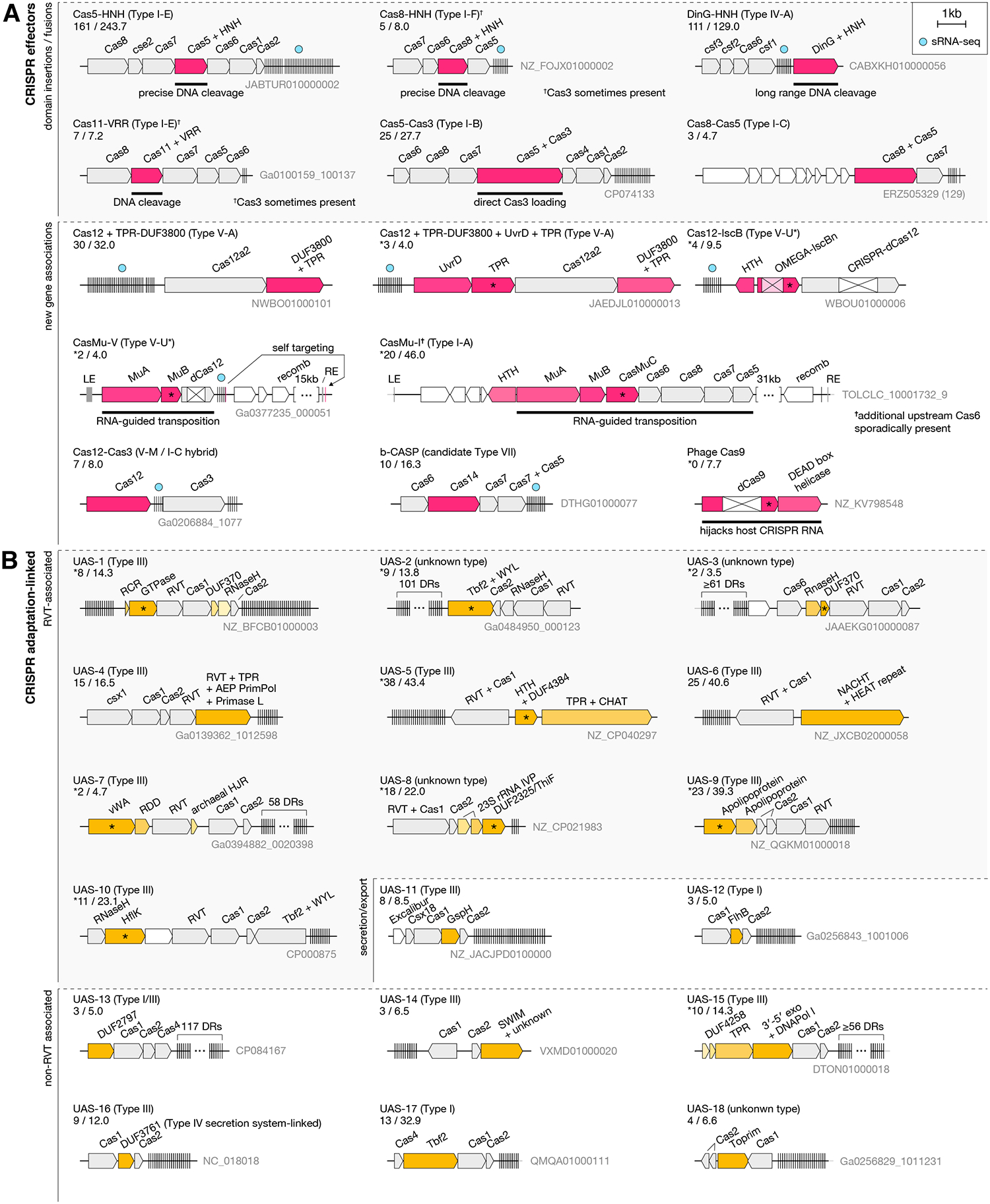

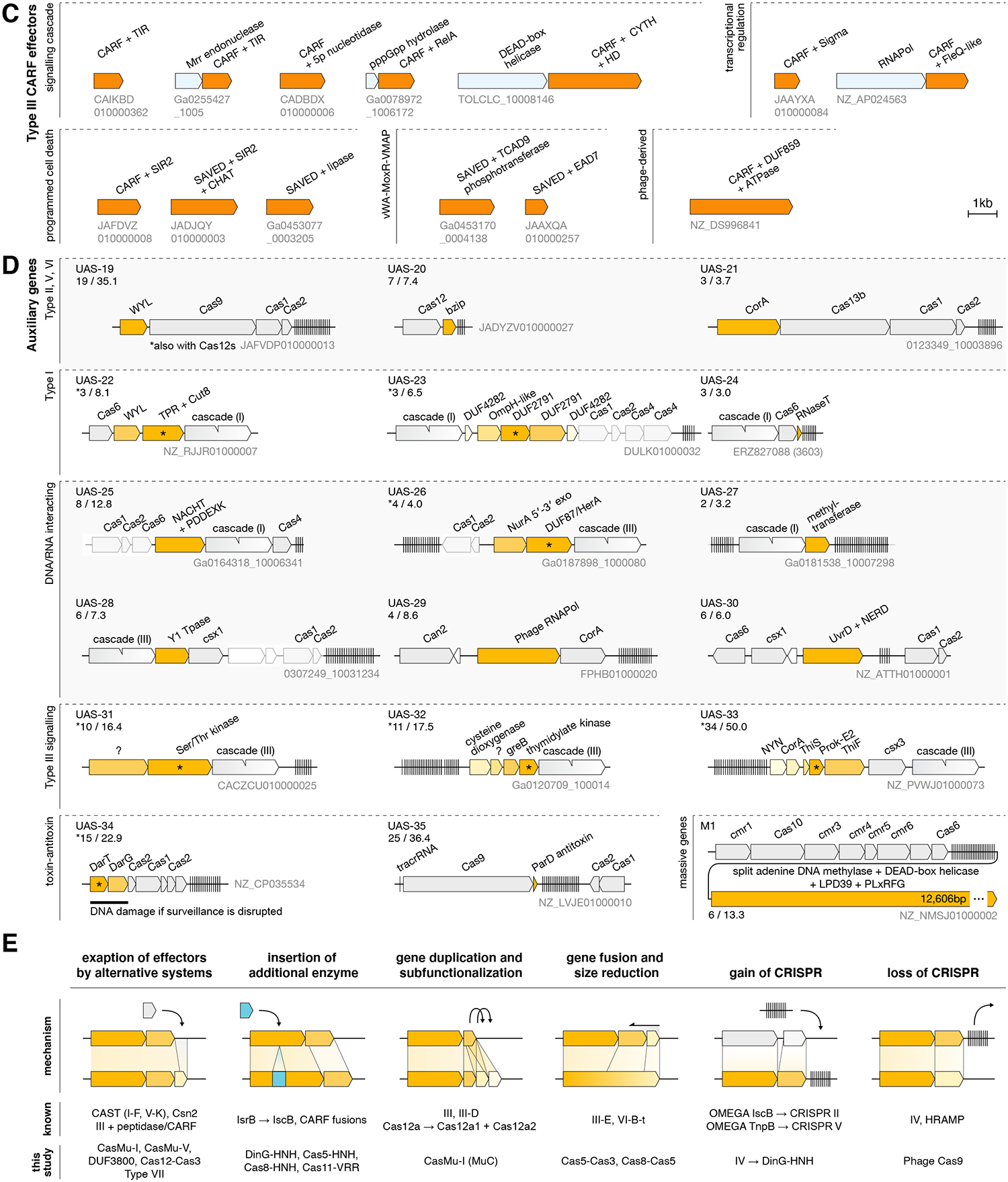
Diverse CRISPR systems identified in this study Genomic loci of identified systems. See Fig. S12–S14 for full set of systems **(A)** CRISPR-Cas effector modules identified in this study. All enhanced CRISPR association scores are shown below the system name as determined by the pipeline with the numerator indicating the number of CRISPR / divergent DR associated loci and the denominator indicating the effective sample size of the cluster. HNH: Nuclease domain with HNH or HNN catalytic motifs. DinG: Damage Inducible gene G helicase. VRR: PDDEXK nuclease domain. TPR: Tetratricopeptide repeat. MuA: DDE transposase gene associated with Mu transposons. MuB, ATPase gene associated with Mu transposons. CasMuC: Unique gene associated mainly with the CasMu-I system. β-CASP: Metallo-β-lactamase. **(B)** Novel associations of CRISPR adaptation modules. Enhanced CRISPR association scores shown as in (**A)**. RVT: Reverse Transcriptase. Tfb2: Transcription factor B subunit 2. WYL: domain named after the 3 conserved amino acids in the domain. AEP: archaeo-eukaryotic primase. PrimPol: Primase Polymerase. HTH: Helix-Turn-Helix domain. CHAT: Caspase HetF Associated with TPRs domain. NACHT: predicted nucleoside-triphosphatase (NTPase) domain. vWA: von Willebrand factor type A. HJR: Holliday Junction Resolvase. RDD: domain named after its conserved amino acids. 23S rRNA IVP: 23S rRNA-Intervening Sequence Protein. ThiF: Sulfur carrier protein ThiS adenylyltransferase. HflK: regulator of FtsH protease. GspH: Type II secretion system protein H. FlhB: Flagellar biosynthetic protein. SWIM: Zinc Finger domain. Toprim: topoisomerase-primase domain. **(C)** CRISPR-linked CARF/SAVED cyclic oligonucleotide binding domain proteins associated with CRISPR arrays. CARF: CRISPR-Associated Rossmann Fold. TIR: Toll/interleukin-1 receptor/resistance protein. RelA: (p)ppGpp synthetase. CYTH: adenylyl cyclase/thiamine triphosphatase. HD: phosphohydrolase. FleQ: transcriptional regulator. SIR2: sirtuin-like domain. vWA-MoxR-VMAP: classical NTP-dependent ternary system involved in conflict systems. TCAD9: Ternary Complex-Associated Domain 9 associated with vWA-MoxR-VMAP. EAD7: Effector-associated domain 7 associated with vWA-MoxR-VMAP. **(D)** Putative CRISPR auxiliary genes. Enhanced CRISPR association scores shown as in (**A)**. bZIP: Basic Leucine Zipper Domain. CorA: Magnesium transporter. OmpH: outer membrane protein. NurA 5′−3′ exo: DNA double stranded break-repair associated exonuclease. HerA: DNA-repair associated helicase. Y1 Tpase: Y1 tyrosine recombinase. UvrD: helicase. NERD: Nuclease-related Domain. GreB: Transcription elongation factor. NYN: Novel Predicted RNAses with a PIN Domain-Like Fold. ThiS: Sulfur Carrier Protein. Prok-E2: Prokaryotic E2 family A. DarT: thymidine ADP-ribosylation enzyme. DarG: ADP-ribosylation reversal enzyme. ParD: Antitoxin component of the ParDE toxin-antitoxin system. LPD39: Large polyvalent protein-associated domain 39. PLxRFG: domain characteric of some very large proteins in bacteria. **(E)** General evolutionary mechanisms that likely gave rise to the diverse CRISPR-Cas effector modules identified previously and in this study.

Our biodiscovery pipeline identified many additional putative novel systems (Fig. 6, S12–14, Data S2). In total, we identified 188 CRISPR-linked gene modules that, to the best of our knowledge, have not been reported previously (Fig. S14A–GF, Data S2). These systems have been designated as UAS-# (Unknown Associated System), and may each contain multiple genes, (designated uas#A, uas#B… if not previously named). From these findings, several themes emerged. First, we identified at least 17 cases where the core effector modules contained new domains or fusions, including the DinG-HNH, Cas8-HNH, Cas5-HNH, and candidate type VII systems (Fig. 6A). We also discovered a VRR-NUC (PD(D/E)XK superfamily) nuclease fused to Cas11 subunit in I-E systems. Apart from these novel domains, we identified a type I-B variant with a fusion of Cas5 to Cas3, which might allow direct loading of Cas3 to the target DNA upon its recognition by Cascade. Similarly, we found a Cas8-Cas5 fusion in an incomplete type I-C system that apparently lacks Cas3 and may function as a DNA binder.

### CRISPR-associated transposons

A second, related theme is the association of new genes with core CRISPR effector modules, which is consistent with previous studies showing that the RNA guided mechanism of CRISPR has been repurposed for different functions (Fig. 6A) (53–55). For example, we discovered Mu transposases (56) associated with type V and type I-A systems (CasMu-V and CasMu-I, respectively), in which the effector nuclease activity was lost, either due to apparent catalytic inactivation of Cas12 via the loss of the RuvC-III motif (type V) or via the loss of the entire *cas3* gene (type I). CasMu-I is additionally associated with an HTH domain-containing protein and a gene denoted cas*muC*, which encodes an inactivated paralog of the associated MuA transposase. Using AlphaFold2, we predicted interaction between the CasMuC protein and Cas8, suggesting that CasMuC may serve as a novel adaptor between the transposase and the CRISPR effector complex (Fig. S15). Using sequence alignments, read mapping, and comparison with other Mu transposon ends, we identified the left and right ends of the transposon for both classes of CasMu systems. In one example of CasMu-V, we further identified a cryptic homing spacer in the CRISPR array matching a site 68bp downstream of the right end, suggesting an RNA-guided homing mechanism (Fig. 6A, S16) (57). Thus, CasMu-V and CasMu-I appear to be distinct CRISPR-associated transposons that employ interference-defective CRISPR systems for reprogrammable RNA-guided transposition, a mechanism that was previously known to exist only for Tn7-like transposons (53).

### Multicomponent Cas12-linked systems

In addition to transposon association, we identified several further examples of previously unknown associations with core CRISPR effector modules. These included combinations of Cas12 with proteins such as Cas3, OMEGA-IscB and an HTH domain, and a TPR-DUF3800 domain-containing protein (Fig. 6A). The Cas12-Cas3 system is a putative Class-1–2 hybrid system in which a Cas12m, which is not known to exhibit DNA cleavage activity (58), may have associated with a Cas3 helicase-nuclease (type I-C like) to provide an interference mechanism beyond DNA binding. The Cas12 associated with an OMEGA-IscB and an HTH domain protein is inactivated, whereas the associated IscB protein has an inactivated RuvC domain and active HNH domain, suggesting it functions as a nickase; these two RNA-guided modules may work in concert to facilitate targeting or in opposition to exclude each other under certain conditions. We found that a sub-branch of Cas12a2 is associated with a TPR + DUF3800 domain protein and occasionally with a UvrD helicase and an additional TPR domain-containing protein. AlphaFold2 prediction of the DUF3800 domain-containing protein indicated that DUF3800 contains an RNaseH nuclease fold with a catalytic rearrangement (Fig. S17). Additionally, the DUF3800 domain has been previously found to be associated with putative ncRNAs (59). Together, this suggests it may function as part of the interference module or in crRNA biogenesis or degradation in these systems. The presence of multiple TPR domains, which facilitate protein-protein interactions (60), suggests interaction between the various components of these systems, possibly with consequences for the interference mechanism.

We tested several of these new type V systems (CasMu-V, Cas12+TPR-DUF3800, Cas12+TPR-DUF3800+UvrD+TPR, Cas12+IscB, Cas12-Cas3) for ncRNA binding by the Cas12 effectors by purifying Cas12 proteins and sequencing any associated RNA. We found that all of these Cas12s co-purified with a cognate ncRNA, usually a processed crRNA derived from the associated CRISPR array (Fig. S18) suggesting these are functional CRISPR systems in which Cas12 operates as an RNA-guided targeting module.

### Biomimicry anti-CRISPR strategy employed by viruses

We next examined the dataset to identify homologs of Cas proteins that have lost CRISPR array association. We found a type II-C Cas9 with a catalytically inactivated RuvC nuclease domain, but an active HNH domain, that is encoded in phage genomes and associated with an SNF2 helicase but not with CRISPR arrays (score of 0) (Fig. 6A, S19A). A putative tracrRNA was found in the vicinity of this phage type II locus. For one of these systems, we identified the corresponding host bacterium in the same sequencing sample, which encoded its own type II-C CRISPR-Cas system with a catalytically active Cas9 (Fig. S19B). Among the spacers in the host CRISPR array, there were 4 matches to the corresponding phage system (Fig. S19C, D). The phage-encoded tracrRNA contained a perfect anti-repeat to the host DRs, such that these two RNAs are predicted to form a more stable complex than the host tracrRNA:crRNA complex (Fig. S19E). Along with the structural similarity of the two Cas9s (Fig. S19F, Fig. S19G), these observations suggest that the phage Cas9 derails the host CRISPR system by forming stable complexes with the crRNAs, which is a distinct mechanism that further adds to the striking diversity of anti-CRISPR strategies employed by viruses (61, 62).

### Diverse auxiliary and adaptation-linked CRISPR genes

Apart from variations on the effector modules, a third emerging theme is linkage between genes not previously known to associate with CRISPR and CRISPR adaptation modules. For example, we found Cas adaptation modules linked with RNaseH (UAS-3, UAS-45) and DNA polymerases (UAS-4, UAS-15), as well as a variety of unexpected genes, such as transmembrane domain proteins (Fig. 6B, Fig. S14U–AS). In addition, we identified numerous CRISPR-Associated Rossmann Fold (CARF) domain-containing putative effectors in the vicinity of type III CRISPR loci, including two-component RNAPol + CARF (UAS-58), pppGpp hydrolase + RelA systems (UAS-50), and ternary complex vWA-MoxR-VMAP coupled domains (UAS-55, UAS-64, UAS-66), suggesting diverse mechanisms of CRISPR-activated signaling cascades potentially linked to other cell stress pathways (Fig. 6C) (63). We found that diverse vWA-related systems associate more broadly with CRISPR loci alongside kinase, phosphatase, transmembrane, and tubulin domain proteins (UAS-7, UAS-87, UAS-91, UAS-100, UAS-129, UAS-139, UAS-149, and UAS-155). Additionally, a variety of putative regulatory, signaling, and nucleic acid-binding proteins were found to be associated with both Class 1 and Class 2 systems as well as numerous toxin-antitoxin modules that could safeguard *cas* genes as previously described for some type I systems, or otherwise interact with the CRISPR machinery (Fig. 6D) (64, 65). We also identified large CRISPR-associated genes encoding functionally uncharacterized giant multidomain proteins (>3,000 aa), one of which, M1, contains multiple DNA interacting domains (Fig. 6D).

### Hypervariable, regularly interspersed repeat array systems

Finally, we identified putative new functional systems associated with regularly interspaced repeat arrays with hypervariable spacers, analogous to CRISPR arrays and ωRNA arrays (25), but lacking any *cas* genes (Fig. S14GJ–GO). These systems are distinct from CRISPR, but might contain novel modular functions as previously observed for hypervariable repeat proteins (67). We identified 6 systems containing predicted nucleic acid interacting proteins associated with other, non-CRISPR interspaced repeat arrays (Fig. S14GJ–GO, S20A). One of these systems included an AddB-like PD(D/E)XK family nuclease/helicase with an inactivated helicase domain associated with CRISPR-like repeats that are preceded by a predicted conserved promoter, suggesting that the array is expressed. We performed small RNA-seq on *E. coli* harboring plasmids carrying these systems and found they expressed small RNAs overlapping the repeats and hypervariable spacer regions of the arrays (Fig. S20B).

A second system included a GGDEF domain (cyclic di-GMP synthetase) and an MFS transporter, with an interspersed repeat array encoded between them, along with additional phospholipase, LCP phosphotransferase and HTH domain proteins (Fig. S20A). We performed small RNA-seq on native organisms harboring GGDEF loci and observed transcription across the identified repeat arrays, with apparent processing of the RNA (Fig. S20C). By analogy with the Cas10 protein of type III CRISPR systems, which contains a divergent GGDEF domain that, in response to virus infection, produces cyclic oligoadenylate that activates downstream effectors, these GGDEF-containing systems could also produce a second messenger activating an RNA-guided component of the system. Thus, these systems generally resemble CRISPR and might represent a novel RNA-guided mechanism with defense or other functions.

### Systems associated with tRNA arrays with variable spacers

We further identified 3 systems associated with interspaced tRNA-arrays separated by similarly sized variable sequences that could modulate the function of the tRNAs through mechanisms such as differential expression or processing of individual tRNAs units (Fig. S14GG–GI, S21). This is consistent with the association of some of these tRNA arrays with nucleic acid processing enzymes, such as RNaseR, RNaseH and DNA Pol III epsilon-like exonuclease. Overall, these systems might represent diverse functions beyond CRISPR that employ repeat arrays with hypervariable spacers to carry out defense and/or regulatory functions.

## Discussion

The continuing and accelerating proliferation of public sequence data has the potential to transform biology, but realizing this potential requires computational approaches that can keep pace with database growth. Central to this effort is moving away from all-to-all comparisons. Here, we used LSH to develop FLSHclust, an algorithm for clustering proteins by sequence similarity that, unlike the currently available methods, can quickly and efficiently cluster millions of sequences, and will be applicable to a broad variety of studies that involve mining large databases. We applied FLSHclust to identify numerous previously unreported CRISPR systems and associated genes. The systems identified here are rare, with many encompassing only a single cluster out of the ~130,000 CRISPR-linked clusters we identified, indicating that the high throughput approach we applied is indispensable for the discovery of previously unknown CRISPR variants as well as rare variants of other functional systems. To identify CRISPR-linked genes, we used the association score, which we refined during this work, with a conservative cut-off. Any such cut-off may lead to false negatives, but given the vast amount of data analyzed, we focused on the most reliable predictions. The discovery of new *cas* genes and CRISPR systems substantially expands the known CRISPR diversity, emphasizing the functional versatility of CRISPR whereby new proteins and domains are often recruited, either replacing pre-existing components or conferring new functions to the pre-existing scaffold of Cas proteins (Fig. 6E).

We observed many new domains and proteins associated with CRISPR effector modules, several of which appear to compensate for the functions of lost components (Fig. 6A), highlighting the modular evolution of CRISPR effectors. We identified HNH nuclease domains as additions to pre-existing CRISPR systems on three independent occasions: DinG-HNH, Cas5-HNH and Cas8-HNH (Fig. 3, 4). The evolution of these systems mimics the origin of type II CRISPR systems, in which an HNH nuclease was inserted into the RuvC-like nuclease domain of the IsrB protein to become IscB, the likely direct ancestor of Cas9 (Fig. 6E) (25). Another notable case is the candidate type VII CRISPR system discovered here, in which the enzymatic domains of Cas10 were functionally replaced by the unrelated β-CASP nuclease (Fig. 5). Although the β-CASP-containing CRISPR systems appear to be distantly related to and most likely derived from type III CRISPR systems (Fig. S6C), which also appears to be the case for type IV systems (69, 70), the limited sequence similarity among the shared components (Fig. S6H–I) and the recruitment of a distinct interference effector suggests classification of these systems as type VII. Similarly, the discovery of a broad variety of proteins and domains associated with CRISPR adaptation modules (Fig. 6B) suggests the existence of many functional and mechanistic variations in this first stage of the CRISPR function. CRISPR systems can also be co-opted for other RNA-guided functions, such as transposition (71–74), and the present work extends this form of exaptation beyond Tn7-like transposons through the discovery of CasMu-I and CasMu-V.

Taken together, the results of this work reveal unprecedented organizational and functional flexibility and modularity of CRISPR systems but also demonstrate that most variants are rare and only found in relatively unusual bacteria and archaea. Apparently, during the billions of years of the evolution of prokaryotes, a limited number of fittest variants spread broadly by horizontal transfer, preventing extensive dissemination of the great majority of emerging variants. The causes of the higher fitness of those (relatively) few successful variants are a major challenge for future studies.

Due to the ability of CRISPR-Cas systems to programmably sense specific nucleic acids and subsequently enact enzymatic functions, the discovery and characterization of novel CRISPR effectors and downstream auxiliary functions has the potential to enable a wide range of applications and improve existing CRISPR-based technologies. Here, we characterized the genome editing activities of Cas8-HNH and Cas5-HNH nucleases, which showed striking precision and hold promise for further development as genome editing tools. The Cas5-HNH system may also have applications in diagnostics given its collateral cleavage activity. Beyond genome editing, CRISPR adaptation machinery has emerged as a powerful tool for molecular recording, highlighting the importance of identifying novel biochemical functions associated with the adaptation genes to expand the function and scope of such technologies. CRISPR-associated CARF/SAVED domain effectors could be developed as sensitive molecular sense-and-respond tools, as they enact diverse enzymatic functions that are allosterically activated by cyclic oligonucleotide binding by the CARF/SAVED domain, which is in turn a response to targeted RNA recognition (71–74). Notably, we report the first identification of multi-component CARF/SAVED systems, suggesting that these systems engage in natural, multi-protein signaling cascades that could be further adapted for biotechnology. This represents only a small fraction of the discovered systems, but it illuminates the vastness and untapped potential of Earth’s biodiversity, and the remaining candidates will serve as a resource for communal exploration.

## Methods summary

A complete “Materials and Methods” section is provided in the supplement.

### FLSHclust implementation

The FLSHclust algorithm was implemented in Python 3 using PySpark for distributed computation on clusters without shared memory or disk. The algorithm is visually depicted in Fig. S1. Complete details and benchmarking comparisons are described in Materials and Methods.

### Sensitive CRISPR discovery pipeline

For CRISPR prediction, 4 CRISPR finders (PILERCR (21), CRT (22), CRISPRFinder (23) and CRONUS) were used with a total of 6 runs based on parameter combinations selected from a calibration against the synthetic CRISPR array benchmark. CRISPR array predictions from the various CRISPR finders were deduplicated by grouping in intervals and the best CRISPR from each interval was selected. Operons were then defined from predicted proteins in each contig, and operonic distance from each operon to CRISPR arrays was calculated. We used a maximum distance threshold of 3000 bp to select protein operons associated with CRISPR arrays. Proteins were then redundancy reduced and we then calculated a weighted naive score for each resulting 30% cluster. Divergent DRs were identified by searching for consensus DRs (identified from each cluster) within a 10 kbp window of each protein in the 30% cluster. The enhanced score was calculated in the same manner as the naive score, now using the searched DRs.

### E. coli PAM discovery assay

Plasmids expressing the proteins and corresponding crRNA from the system of interest and containing a target 8N degenerate flanking library plasmid were transformed by electroporation into Endura Electrocompetent E. coli (Lucigen). After 12–16 h, cells were scraped from transformant plates and miniprepped to recover the resulting libraries, which were prepared and sequenced on an Illumina NextSeq. PAMs were extracted and Weblogos depicting PAMs depleted 5 standard deviations relative to the empty control were visualized using Weblogo3.

### Expression and purification of recombinant proteins

*E. coli* codon optimized proteins and associated ncRNAs were expressed from IPTG-inducible T7 promoters and purified with His14 or TwinStrep tags as specified using nickel or streptavidin affinity resin, respectively, using gravity flow columns. In some cases, purified proteins or RNPs were dialyzed overnight before use.

### Small RNA sequencing

Total RNA was extracted from native organisms, *E. coli* cultures containing plasmids encoding loci of interest, or affinity purified RNP complexes. The purified RNA was then subject to treatment with T4 PNK (NEB) and RNA 5′ polyphosphatase (Biosearch Technologies). Following enzymatic treatments, purified RNA was subject to library preparation with an NEBNext Multiplex Small RNA Library Prep kit (NEB) and sequenced on an Illumina MiSeq or NextSeq.

### In vitro cleavage assays

Nucleic acid substrates were prepared by PCR with Cy3/Cy5 conjugated oligos (IDT) as primers (dsDNA), ordered directly as Cy5-conjugated oligos (IDT) (ssDNA), or in vitro transcribed from PCR templates and labeled with pCp-Cy5 (Jena Biosciences) using T4 RNA ligase 1, ssRNA ligase (High Concentration) (NEB) (RNA). Substrates were mixed with protein and buffer components and incubated at various temperatures, and results were resolved by gel electrophoresis, as specified in Materials and Methods.

### Mammalian genome editing

Genome editing experiments were performed in the HEK293FT cell line (Thermo Fisher Scientific). Cells were transfected with Lipofectamine 3000 and gDNA was harvested 60–72 hours after transfection using QuickExtract DNA Extraction Solution (Lucigen). Target genomic regions were amplified by 2 rounds of PCR with NEBNext High Fidelity 2x PCR Master Mix (NEB) and sequenced on an Illumina MiSeq. Indel frequency was analyzed using CRISPResso2.

## Supplementary Material

supplementary information

data S2

data S1

data S3

data S4

data S6

data S5

Table S2

Table S3

Table S4

Table S5

Table S6

Table S1

## Data Availability

Sequences and information on protein clusters are available in the supplementary materials. Sequences of genes used in the experimental studies are available via online sequence repositories and expression plasmids are available from Addgene under a uniform biological material transfer agreement. Scripts for data analysis and visualization, as well as all redundant genomic loci for all identified systems are available via Zenodo (75). Additional information available via the Zhang Lab website (https://zhanglab.bio).

## References

[1] Wang JY, Doudna JA, CRISPR technology: A decade of genome editing is only the beginning. Science 379, eadd8643 (2023). 36656942 10.1126/science.add8643

[2] Shmakov SA, Faure G, Makarova KS, Wolf YI, Severinov KV, Koonin EV, Systematic prediction of functionally linked genes in bacterial and archaeal genomes. Nature Protocols 14 (2019), pp. 3013–3031. 31520072 10.1038/s41596-019-0211-1 PMC6938587

[3] Yan WX, Hunnewell P, Alfonse LE, Carte JM, Keston-Smith E, Sothiselvam S, Garrity AJ, Chong S, Makarova KS, Koonin EV, Cheng DR, Scott DA, Functionally diverse type V CRISPR-Cas systems. Science 363 (2019), pp. 88–91. 30523077 10.1126/science.aav7271 PMC11258546

[4] Shmakov S, Abudayyeh OO, Makarova KS, Wolf YI, Gootenberg JS, Semenova E, Minakhin L, Joung J, Konermann S, Severinov K, Zhang F, Koonin EV, Discovery and functional characterization of diverse Class 2 CRISPR-Cas systems. Mol. Cell 60, 385 (2015). 26593719 10.1016/j.molcel.2015.10.008 PMC4660269

[5] Hille F, Richter H, Wong SP, Bratovič M, Ressel S, Charpentier E, The biology of CRISPR-Cas: Backward and forward. Cell 172, 1239–1259 (2018). 29522745 10.1016/j.cell.2017.11.032

[6] Makarova KS, Wolf YI, Iranzo J, Shmakov SA, Alkhnbashi OS, Brouns SJJ, Charpentier E, Cheng D, Haft DH, Horvath P, Moineau S, Mojica FJM, Scott D, Shah SA, Siksnys V, Terns MP, Venclovas Č, White MF, Yakunin AF, Yan W, Zhang F, Garrett RA, Backofen R, van der Oost J, Barrangou R, Koonin EV, Evolutionary classification of CRISPR-Cas systems: a burst of class 2 and derived variants. Nat. Rev. Microbiol 18, 67–83 (2020). 31857715 10.1038/s41579-019-0299-x PMC8905525

[7] Kannan S, Altae-Tran H, Jin X, Madigan VJ, Oshiro R, Makarova KS, Koonin EV, Zhang F, Compact RNA editors with small Cas13 proteins. Nat. Biotechnol 40, 194–197 (2022). 34462587 10.1038/s41587-021-01030-2 PMC8929162

[8] Shmakov S, Smargon A, Scott D, Cox D, Pyzocha N, Yan W, Abudayyeh OO, Gootenberg JS, Makarova KS, Wolf YI, Severinov K, Zhang F, Koonin EV, Diversity and evolution of class 2 CRISPR-Cas systems. Nat. Rev. Microbiol 15, 169–182 (2017). 28111461 10.1038/nrmicro.2016.184 PMC5851899

[9] Shmakov SA, Makarova KS, Wolf YI, Severinov KV, Koonin EV, Systematic prediction of genes functionally linked to CRISPR-Cas systems by gene neighborhood analysis. Proc. Natl. Acad. Sci. U. S. A 115, E5307–E5316 (2018). 29784811 10.1073/pnas.1803440115 PMC6003329

[10] Consortium UniProt, UniProt: a worldwide hub of protein knowledge. Nucleic Acids Res 47, D506–D515 (2019). 30395287 10.1093/nar/gky1049 PMC6323992

[11] Steinegger M, Söding J, Clustering huge protein sequence sets in linear time. Nat. Commun 9, 2542 (2018). 29959318 10.1038/s41467-018-04964-5 PMC6026198

[12] Doron S, Melamed S, Ofir G, Leavitt A, Lopatina A, Keren M, Amitai G, Sorek R, Systematic discovery of antiphage defense systems in the microbial pangenome. Science 359 (2018), doi:10.1126/science.aar4120. PMC6387622 29371424

[13] Gao L, Altae-Tran H, Böhning F, Makarova KS, Segel M, Schmid-Burgk JL, Koob J, Wolf YI, Koonin EV, Zhang F, Diverse enzymatic activities mediate antiviral immunity in prokaryotes. Science 369, 1077–1084 (2020). 32855333 10.1126/science.aba0372 PMC7985843

[14] Pagh R, CoveringLSH. ACM Transactions on Algorithms 14 (2018), pp. 1–17.

[15] Li W, Godzik A, Cd-hit: a fast program for clustering and comparing large sets of protein or nucleotide sequences. Bioinformatics 22, 1658–1659 (2006). 16731699 10.1093/bioinformatics/btl158

[16] Steinegger M, Söding J, MMseqs2 enables sensitive protein sequence searching for the analysis of massive data sets. Nat. Biotechnol 35, 1026–1028 (2017). 29035372 10.1038/nbt.3988

[17] Edgar RC, Search and clustering orders of magnitude faster than BLAST. Bioinformatics 26, 2460–2461 (2010). 20709691 10.1093/bioinformatics/btq461

[18] Suzek BE, Wang Y, Huang H, McGarvey PB, Wu CH, UniProt Consortium, UniRef clusters: a comprehensive and scalable alternative for improving sequence similarity searches. Bioinformatics 31, 926–932 (2015). 25398609 10.1093/bioinformatics/btu739 PMC4375400

[19] Zaharia M, An Architecture for Fast and General Data Processing on Large Clusters (Morgan & Claypool, 2016; https://play.google.com/store/books/details?id=a8wvDAAAQBAJ).

[20] Lomsadze A, Gemayel K, Tang S, Borodovsky M, Modeling leaderless transcription and atypical genes results in more accurate gene prediction in prokaryotes. Genome Res 28, 1079–1089 (2018). 29773659 10.1101/gr.230615.117 PMC6028130

[21] Edgar RC, PILER-CR: fast and accurate identification of CRISPR repeats. BMC Bioinformatics 8, 18 (2007). 17239253 10.1186/1471-2105-8-18 PMC1790904

[22] Bland C, Ramsey TL, Sabree F, Lowe M, Brown K, Kyrpides NC, Hugenholtz P, CRISPR recognition tool (CRT): a tool for automatic detection of clustered regularly interspaced palindromic repeats. BMC Bioinformatics 8, 209 (2007). 17577412 10.1186/1471-2105-8-209 PMC1924867

[23] Grissa I, Vergnaud G, Pourcel C, CRISPRFinder: a web tool to identify clustered regularly interspaced short palindromic repeats. Nucleic Acids Res 35, W52–7 (2007). 17537822 10.1093/nar/gkm360 PMC1933234

[24] Biswas A, Staals RHJ, Morales SE, Fineran PC, Brown CM, CRISPRDetect: A flexible algorithm to define CRISPR arrays. BMC Genomics 17, 356 (2016). 27184979 10.1186/s12864-016-2627-0 PMC4869251

[25] Altae-Tran H, Kannan S, Demircioglu FE, Oshiro R, Nety SP, McKay LJ, Dlakić M, Inskeep WP, Makarova KS, Macrae RK, Koonin EV, Zhang F, The widespread IS200/IS605 transposon family encodes diverse programmable RNA-guided endonucleases. Science 374, 57–65 (2021). 34591643 10.1126/science.abj6856 PMC8929163

[26] Faure G, Shmakov SA, Yan WX, Cheng DR, Scott DA, Peters JE, Makarova KS, Koonin EV, CRISPR-Cas in mobile genetic elements: counter-defence and beyond. Nat. Rev. Microbiol 17, 513–525 (2019). 31165781 10.1038/s41579-019-0204-7 PMC11165670

[27] Pourcel C, Touchon M, Villeriot N, Vernadet J-P, Couvin D, Toffano-Nioche C, Vergnaud G, CRISPRCasdb a successor of CRISPRdb containing CRISPR arrays and cas genes from complete genome sequences, and tools to download and query lists of repeats and spacers. Nucleic Acids Res 48, D535–D544 (2020). 31624845 10.1093/nar/gkz915 PMC7145573

[28] Taylor HN, Warner EE, Armbrust MJ, Crowley VM, Olsen KJ, Jackson RN, Structural basis of Type IV CRISPR RNA biogenesis by a Cas6 endoribonuclease. RNA Biol 16, 1438–1447 (2019). 31232162 10.1080/15476286.2019.1634965 PMC6779396

[29] Özcan A, Pausch P, Linden A, Wulf A, Schühle K, Heider J, Urlaub H, Heimerl T, Bange G, Randau L, Type IV CRISPR RNA processing and effector complex formation in Aromatoleum aromaticum. Nat Microbiol 4, 89–96 (2019). 30397343 10.1038/s41564-018-0274-8

[30] Pinilla-Redondo R, Mayo-Muñoz D, Russel J, Garrett RA, Randau L, Sørensen SJ, Shah SA, Type IV CRISPR-Cas systems are highly diverse and involved in competition between plasmids. Nucleic Acids Res 48, 2000–2012 (2020). 31879772 10.1093/nar/gkz1197 PMC7038947

[31] Guo X, Sanchez-Londono M, Gomes-Filho JV, Hernandez-Tamayo R, Rust S, Immelmann LM, Schäfer P, Wiegel J, Graumann PL, Randau L, Characterization of the self-targeting Type IV CRISPR interference system in Pseudomonas oleovorans. Nat Microbiol 7, 1870–1878 (2022). 36175516 10.1038/s41564-022-01229-2

[32] Crowley VM, Catching A, Taylor HN, Borges AL, Metcalf J, Bondy-Denomy J, Jackson RN, A Type IV-A CRISPR-Cas System in Mediates RNA-Guided Plasmid Interference. CRISPR J 2, 434–440 (2019). 31809194 10.1089/crispr.2019.0048 PMC6919247

[33] Moya-Beltrán A, Makarova KS, Acuña LG, Wolf YI, Covarrubias PC, Shmakov SA, Silva C, Tolstoy I, Johnson DB, Koonin EV, Quatrini R, Evolution of Type IV CRISPR-Cas Systems: Insights from CRISPR Loci in Integrative Conjugative Elements of. CRISPR J 4, 656–672 (2021). 34582696 10.1089/crispr.2021.0051 PMC8658065

[34] Mulepati S, Bailey S, In vitro reconstitution of an Escherichia coli RNA-guided immune system reveals unidirectional, ATP-dependent degradation of DNA target. J. Biol. Chem 288, 22184–22192 (2013). 23760266 10.1074/jbc.M113.472233 PMC3829311

[35] Hochstrasser ML, Taylor DW, Bhat P, Guegler CK, Sternberg SH, Nogales E, Doudna JA, CasA mediates Cas3-catalyzed target degradation during CRISPR RNA-guided interference. Proc. Natl. Acad. Sci. U. S. A 111, 6618–6623 (2014). 24748111 10.1073/pnas.1405079111 PMC4020112

[36] Zetsche B, Gootenberg JS, Abudayyeh OO, Slaymaker IM, Makarova KS, Essletzbichler P, Volz SE, Joung J, van der Oost J, Regev A, Koonin EV, Zhang F, Cpf1 is a single RNA-guided endonuclease of a class 2 CRISPR-Cas system. Cell 163, 759–771 (2015). 26422227 10.1016/j.cell.2015.09.038 PMC4638220

[37] Cui N, Zhang J-T, Liu Y, Liu Y, Liu X-Y, Wang C, Huang H, Jia N, Type IV-A CRISPR-Csf complex: Assembly, dsDNA targeting, and CasDinG recruitment. Mol. Cell (2023), doi:10.1016/j.molcel.2023.05.036. 37343553

[38] Domgaard H, Cahoon C, Armbrust MJ, Redman O, Jolley A, Thomas A, Jackson RN, CasDinG is a 5′−3′ dsDNA and RNA/DNA helicase with three accessory domains essential for type IV CRISPR immunity. Nucleic Acids Res, gkad546 (2023). 10.1093/nar/gkad546 PMC10450177 37395408

[39] Haurwitz RE, Jinek M, Wiedenheft B, Zhou K, Doudna JA, Sequence- and structure-specific RNA processing by a CRISPR endonuclease. Science 329, 1355–1358 (2010). 20829488 10.1126/science.1192272 PMC3133607

[40] Jinek M, Chylinski K, Fonfara I, Hauer M, Doudna JA, Charpentier E, A programmable dual-RNA-guided DNA endonuclease in adaptive bacterial immunity. Science 337, 816–821 (2012). 22745249 10.1126/science.1225829 PMC6286148

[41] Schmid-Burgk JL, Gao L, Li D, Gardner Z, Strecker J, Lash B, Zhang F, Highly Parallel Profiling of Cas9 Variant Specificity. Mol. Cell 78, 794–800.e8 (2020). 32187529 10.1016/j.molcel.2020.02.023 PMC7370240

[42] Charpentier E, Richter H, van der Oost J, White MF, Biogenesis pathways of RNA guides in archaeal and bacterial CRISPR-Cas adaptive immunity. FEMS Microbiol. Rev 39, 428–441 (2015). 25994611 10.1093/femsre/fuv023 PMC5965381

[43] Dominski Z, Carpousis AJ, Clouet-d’Orval B, Emergence of the β-CASP ribonucleases: highly conserved and ubiquitous metallo-enzymes involved in messenger RNA maturation and degradation. Biochim. Biophys. Acta 1829, 532–551 (2013). 23403287 10.1016/j.bbagrm.2013.01.010

[44] Mandel CR, Kaneko S, Zhang H, Gebauer D, Vethantham V, Manley JL, Tong L, Polyadenylation factor CPSF-73 is the pre-mRNA 3’-end-processing endonuclease. Nature 444, 953–956 (2006). 17128255 10.1038/nature05363 PMC3866582

[45] Phung DK, Etienne C, Batista M, Langendijk-Genevaux P, Moalic Y, Laurent S, Liuu S, Morales V, Jebbar M, Fichant G, Bouvier M, Flament D, Clouet-d’Orval B, RNA processing machineries in Archaea: the 5’−3’ exoribonuclease aRNase J of the β-CASP family is engaged specifically with the helicase ASH-Ski2 and the 3’−5’ exoribonucleolytic RNA exosome machinery. Nucleic Acids Res 48, 3832–3847 (2020). 32030412 10.1093/nar/gkaa052 PMC7144898

[46] Lieber MR, The mechanism of double-strand DNA break repair by the nonhomologous DNA end-joining pathway. Annu. Rev. Biochem 79, 181–211 (2010). 20192759 10.1146/annurev.biochem.052308.093131 PMC3079308

[47] Callebaut I, Moshous D, Mornon J-P, de Villartay J-P, Metallo-beta-lactamase fold within nucleic acids processing enzymes: the beta-CASP family. Nucleic Acids Res 30, 3592–3601 (2002). 12177301 10.1093/nar/gkf470 PMC134238

[48] Moshous D, Callebaut I, de Chasseval R, Corneo B, Cavazzana-Calvo M, Le Deist F, Tezcan I, Sanal O, Bertrand Y, Philippe N, Fischer A, de Villartay JP, Artemis, a novel DNA double-strand break repair/V(D)J recombination protein, is mutated in human severe combined immune deficiency. Cell 105, 177–186 (2001). 11336668 10.1016/s0092-8674(01)00309-9

[49] Jumper J, Evans R, Pritzel A, Green T, Figurnov M, Ronneberger O, Tunyasuvunakool K, Bates R, Žídek A, Potapenko A, Bridgland A, Meyer C, Kohl SAA, Ballard AJ, Cowie A, Romera-Paredes B, Nikolov S, Jain R, Adler J, Back T, Petersen S, Reiman D, Clancy E, Zielinski M, Steinegger M, Pacholska M, Berghammer T, Bodenstein S, Silver D, Vinyals O, Senior AW, Kavukcuoglu K, Kohli P, Hassabis D, Highly accurate protein structure prediction with AlphaFold. Nature 596, 583–589 (2021). 34265844 10.1038/s41586-021-03819-2 PMC8371605

[50] You L, Ma J, Wang J, Artamonova D, Wang M, Liu L, Xiang H, Severinov K, Zhang X, Wang Y, Structure Studies of the CRISPR-Csm Complex Reveal Mechanism of Co-transcriptional Interference. Cell 176, 239–253.e16 (2019). 30503210 10.1016/j.cell.2018.10.052 PMC6935017

[51] Özcan A, Krajeski R, Ioannidi E, Lee B, Gardner A, Makarova KS, Koonin EV, Abudayyeh OO, Gootenberg JS, Programmable RNA targeting with the single-protein CRISPR effector Cas7–11. Nature 597, 720–725 (2021). 34489594 10.1038/s41586-021-03886-5

[52] van Beljouw SPB, Haagsma AC, Rodríguez-Molina A, van den Berg DF, Vink JNA, Brouns SJJ, The gRAMP CRISPR-Cas effector is an RNA endonuclease complexed with a caspase-like peptidase. Science 373, 1349–1353 (2021). 34446442 10.1126/science.abk2718

[53] Peters JE, Makarova KS, Shmakov S, Koonin EV, Recruitment of CRISPR-Cas systems by Tn7-like transposons. Proc. Natl. Acad. Sci. U. S. A 114, E7358–E7366 (2017). 28811374 10.1073/pnas.1709035114 PMC5584455

[54] Strecker J, Demircioglu FE, Li D, Faure G, Wilkinson ME, Gootenberg JS, Abudayyeh OO, Nishimasu H, Macrae RK, Zhang F, RNA-activated protein cleavage with a CRISPR-associated endopeptidase. Science 378, 874–881 (2022). 36423276 10.1126/science.add7450 PMC10028731

[55] Kato K, Okazaki S, Schmitt-Ulms C, Jiang K, Zhou W, Ishikawa J, Isayama Y, Adachi S, Nishizawa T, Makarova KS, Koonin EV, Abudayyeh OO, Gootenberg JS, Nishimasu H, RNA-triggered protein cleavage and cell growth arrest by the type III-E CRISPR nuclease-protease. Science 378, 882–889 (2022). 36423304 10.1126/science.add7347 PMC11126364

[56] Harshey RM, Transposable Phage Mu. Microbiol Spectr 2 (2014), doi:10.1128/microbiolspec.MDNA3-0007-2014. PMC4486318 26104374

[57] Saito M, Ladha A, Strecker J, Faure G, Neumann E, Altae-Tran H, Macrae RK, Zhang F, Dual modes of CRISPR-associated transposon homing. Cell 184, 2441–2453.e18 (2021). 33770501 10.1016/j.cell.2021.03.006 PMC8276595

[58] Wu WY, Mohanraju P, Liao C, Adiego-Pérez B, Creutzburg SCA, Makarova KS, Keessen K, Lindeboom TA, Khan TS, Prinsen S, Joosten R, Yan WX, Migur A, Laffeber C, Scott DA, Lebbink JHG, Koonin EV, Beisel CL, van der Oost J, The miniature CRISPR-Cas12m effector binds DNA to block transcription. Mol. Cell 82, 4487–4502.e7 (2022). 36427491 10.1016/j.molcel.2022.11.003

[59] Weinberg Z, Lünse CE, Corbino KA, Ames TD, Nelson JW, Roth A, Perkins KR, Sherlock ME, Breaker RR, Detection of 224 candidate structured RNAs by comparative analysis of specific subsets of intergenic regions. Nucleic Acids Res 45, 10811–10823 (2017). 28977401 10.1093/nar/gkx699 PMC5737381

[60] D’Andrea LD, Regan L, TPR proteins: the versatile helix. Trends Biochem. Sci 28, 655–662 (2003). 14659697 10.1016/j.tibs.2003.10.007

[61] Pawluk A, Davidson AR, Maxwell KL, Anti-CRISPR: discovery, mechanism and function. Nat. Rev. Microbiol 16, 12–17 (2017). 29062071 10.1038/nrmicro.2017.120

[62] Pawluk A, Amrani N, Zhang Y, Garcia B, Hidalgo-Reyes Y, Lee J, Edraki A, Shah M, Sontheimer EJ, Maxwell KL, Davidson AR, Naturally Occurring Off-Switches for CRISPR-Cas9. Cell 167 (2016), pp. 1829–1838.e9. 27984730 10.1016/j.cell.2016.11.017 PMC5757841

[63] Kaur G, Burroughs AM, Iyer LM, Aravind L, Highly regulated, diversifying NTP-dependent biological conflict systems with implications for the emergence of multicellularity. Elife 9 (2020), doi:10.7554/eLife.52696. PMC7159879 32101166

[64] Maikova A, Peltier J, Boudry P, Hajnsdorf E, Kint N, Monot M, Poquet I, Martin-Verstraete I, Dupuy B, Soutourina O, Discovery of new type I toxin-antitoxin systems adjacent to CRISPR arrays in Clostridium difficile. Nucleic Acids Res 46, 4733–4751 (2018). 29529286 10.1093/nar/gky124 PMC5961336

[65] Shmakov SA, Barth ZK, Makarova KS, Wolf YI, Brover V, Peters JE, Koonin EV, Widespread CRISPR-derived RNA regulatory elements in CRISPR-Cas systems. Nucleic Acids Res (2023), doi:10.1093/nar/gkad495. PMC10450183 37283088

[66] Altae-Tran H, Gao L, Strecker J, Macrae RK, Zhang F, Computational Identification of Repeat-Containing Proteins and Systems. QRB Discovery 1 (2020), , doi:10.1017/qrd.2020.14. PMC10392669 37528961

[67] Doyle EL, Stoddard BL, Voytas DF, Bogdanove AJ, TAL effectors: highly adaptable phytobacterial virulence factors and readily engineered DNA-targeting proteins. Trends Cell Biol 23, 390–398 (2013). 23707478 10.1016/j.tcb.2013.04.003 PMC3729746

[68] Mohanraju P, Saha C, van Baarlen P, Louwen R, Staals RHJ, van der Oost J, Alternative functions of CRISPR-Cas systems in the evolutionary arms race. Nat. Rev. Microbiol 20, 351–364 (2022). 34992260 10.1038/s41579-021-00663-z

[69] Shipman SL, Nivala J, Macklis JD, Church GM, Molecular recordings by directed CRISPR spacer acquisition. Science 353, aaf1175 (2016). 27284167 10.1126/science.aaf1175 PMC4994893

[70] Schmidt F, Cherepkova MY, Platt RJ, Transcriptional recording by CRISPR spacer acquisition from RNA. Nature 562, 380–385 (2018). 30283135 10.1038/s41586-018-0569-1

[71] Kazlauskiene M, Kostiuk G, Venclovas Č, Tamulaitis G, Siksnys V, A cyclic oligonucleotide signaling pathway in type III CRISPR-Cas systems. Science 357, 605–609 (2017). 28663439 10.1126/science.aao0100

[72] Niewoehner O, Garcia-Doval C, Rostøl JT, Berk C, Schwede F, Bigler L, Hall J, Marraffini LA, Jinek M, Type III CRISPR-Cas systems produce cyclic oligoadenylate second messengers. Nature 548, 543–548 (2017). 28722012 10.1038/nature23467

[73] Rostøl JT, Xie W, Kuryavyi V, Maguin P, Kao K, Froom R, Patel DJ, Marraffini LA, The Card1 nuclease provides defence during type III CRISPR immunity. Nature 590, 624–629 (2021). 33461211 10.1038/s41586-021-03206-x PMC7906951

[74] Rouillon C, Schneberger N, Chi H, Blumenstock K, Da Vela S, Ackermann K, Moecking J, Peter MF, Boenigk W, Seifert R, Bode BE, Schmid-Burgk JL, Svergun D, Geyer M, White MF, Hagelueken G, Antiviral signalling by a cyclic nucleotide activated CRISPR protease. Nature 614, 168–174 (2023). 36423657 10.1038/s41586-022-05571-7

[75] Altae-Tran H, Kannan S, Suberski A, Mears K, Demircioglu FE, Moeller FL, Kocalar S, Oshiro R, Makarova KS, Macrae RK, Koonin EV, Zhang F. Code and genomic loci for “Uncovering the functional diversity of rare CRISPR-Cas systems with deep terascale clustering” (Version 1.0) Zenodo 10.5281/zenodo.8371343 PMC10910872 37995242

[76] Hyatt D, Chen G-L, Locascio PF, Land ML, Larimer FW, Hauser LJ, Prodigal: prokaryotic gene recognition and translation initiation site identification. BMC Bioinformatics 11, 119 (2010). 20211023 10.1186/1471-2105-11-119 PMC2848648

[77] Crawley AB, Henriksen JR, Barrangou R, CRISPRdisco: An Automated Pipeline for the Discovery and Analysis of CRISPR-Cas Systems. CRISPR J 1, 171–181 (2018). 31021201 10.1089/crispr.2017.0022 PMC6636876

[78] Studier FW, Protein production by auto-induction in high density shaking cultures. Protein Expr. Purif 41, 207–234 (2005). 15915565 10.1016/j.pep.2005.01.016

[79] Martin M, Cutadapt removes adapter sequences from high-throughput sequencing reads. EMBnet J 17, 10 (2011).

[80] Clement K, Rees H, Canver MC, Gehrke JM, Farouni R, Hsu JY, Cole MA, Liu DR, Joung JK, Bauer DE, Pinello L, CRISPResso2 provides accurate and rapid genome editing sequence analysis. Nat. Biotechnol 37, 224–226 (2019). 30809026 10.1038/s41587-019-0032-3 PMC6533916

[81] Indyk P, Motwani R, “Approximate nearest neighbors” in Proceedings of the thirtieth annual ACM symposium on Theory of computing - STOC ‘98 (ACM Press, New York, New York, USA, 1998; 10.1145/276698.276876).

[82] Traag VA, Waltman L, van Eck NJ, From Louvain to Leiden: guaranteeing well-connected communities. Sci. Rep 9 (2019), doi:10.1038/s41598-019-41695-z. PMC6435756 30914743

