# Supplementary material for: Uncovering the functional diversity of rare CRISPR-Cas systems with deep terascale clustering
Source: Science. Author manuscript; Available in PMC 2024 Mar 4. (PMC10910872; doi:10.1126/science.adi1910)
Supplement: supplementary information [file NIHMS1969090-supplement-supplementary_information.pdf]

Science
AAAS

Supplementary Materials for
Uncovering the functional diversity of rare CRISPR-Cas systems with deep terascale clustering

Han Altae-Tran[1,2,3,4,5,*], Soumya Kannan[1,2,3,4,5,*], Anthony J. Suberski[1,2,3,4,5,‡], Kepler Mears[1,2,3,4,5,‡], F. Esra Demircioglu[1,2,3,4,5], Lukas Moeller[1,2,3,4,5], Selin Kocalar[1,2,3,4,5], Rachel Oshiro[1,2,3,4,5], Kira S. Makarova[6], Rhiannon K. Macrae[1,2,3,4,5], Eugene V. Koonin[6,†], and Feng Zhang[1,2,3,4,5,†]

This PDF file includes:

Materials and Methods
Supplementary text
Figs. S1 to S21
Captions for Tables S1 to S6
Captions for Data S1 to S6

Other Supplementary Materials for this manuscript include the following:

Tables S1 to S6
Data S1 to S6

Affiliations: (1) Howard Hughes Medical Institute, Cambridge, MA 02139, USA; (2) Broad Institute of MIT and Harvard, Cambridge, MA 02142, USA; (3) McGovern Institute for Brain Research at MIT, Cambridge, MA 02139, USA; (4) Department of Brain and Cognitive Science, Massachusetts Institute of Technology, Cambridge, MA 02139, USA; (5) Department of Biological Engineering, Massachusetts Institute of Technology, Cambridge, MA 02139, USA; (6) National Center for Biotechnology Information, National Library of Medicine, National Institutes of Health, Bethesda, MD 20894, USA.

* These authors contributed equally to this work. ‡ These authors contributed equally to this work.
† Correspondence should be addressed to F.Z. (zhang@broadinstitute.org) and E.V.K (koonin@ncbi.nlm.nih.gov).

List of Supplementary Materials:

Materials and Methods 4

Supplementary text. Extended discussion of FLSHclust algorithm 26

Fig. S1. Complete FLSHclust algorithm 27

Fig. S2. Empirical scaling and benchmarking of FLSHclust vs other clustering algorithms 29

Fig. S3. Performance benchmarks of various CRISPR finders against synthetically generated CRISPRs 34

Fig. S4. Biochemical characterization of DinG-HNH system 43

Fig. S5. Additional characterization of Cas8-HNH and Cas5-HNH effector complexes 45

Fig. S6. Analysis of candidate type VII proteins 46

Fig. S7. Structural comparison of Cas14 β-CASP domain of and human β-CASP protein 49

Fig. S8. Spacer matches for candidate type VII system 50

Fig. S9. New candidate subtype of type III CRISPR 51

Fig. S10. RNA cleavage by candidate type VII system. 52

Fig. S11. Comparison of CRISPR types along with candidate type VII CRISPR type 53

Fig. S12. Index of all systems identified in this study 54

Fig. S13. Representative loci of all systems identified in this study 59

Fig. S14. Limited loci of all systems identified in this study 86

Fig. S15. AlphaFold2 predictions of CasMu-I system 284

Fig. S16. CasMu-V Transposon ends and homing spacer 285

Fig. S17. Structural comparison of DUF3800-TPR protein 286

Fig. S18. Small RNA-seq of Cas12 RNPs from new Type-V associated systems 288

Fig. S19. PhageCas9 compared to a corresponding Cas9 in the host organism 290

Fig. S20. Additional interspaced repeat array systems 294

Fig. S21. Alignments of various tRNA associated systems with variable spacer regions. 295

Table S1. CRISPR association scores of clusters passing filters 298

Table S2. Redundant names for Cas proteins 298

Table S3. Pipeline comparisons 298

Table S4. Guide sequences, data and statistical analysis related to genome editing experiments 298

Table S5. Taxonomic distribution of β-CASP proteins 298

Table S6. List of plasmids used in this study and links to sequences 298

Data S1. Appearance of CRISPR systems in public data 299

Data S2. Genbank files of all redundant loci associated with the manually curated set of hits 299

Data S3. Representative proteins from systems identified in this study 299

Data S4. Protein-protein association analysis results 299

Data S5. Spacer analysis for candidate type VII system 299

Data S6. Genbank files of all plasmids 299

Materials and Methods

FLSHclust implementation

The FLSHclust algorithm was implemented in Python 3 using PySpark for distributed computation on commodity clusters without shared memory or disk. Described below are the general implementation details (see Fig S1 for visual depiction of the algorithm).

The algorithm has the following core parameters: min_seq_id: The minimum sequence identity for including proteins in a cluster, cov: The minimum coverage between sequences for alignment, cov_approx_factor: an approximation parameter for filtering out sequences pairs that will not match the clustering criteria without actually aligning the sequences, k: The main kmer length used for clustering.

The family of hash functions used for locality-sensitive hashing is random position masking, which involves dropping random positions in each kmer before matching them with a hash table. Each hash function drops different combinations of positions. Thus, the set of hash functions can be reduced to the set of position combinations that are dropped, $\{H_i\}$, which essentially is a boolean matrix of positions that are to be dropped out. Optimal functions are generated using Markov Chain Monte Carlo (MCMC) as follows. We use the following method to achieve hash functions with no false negatives for up to r mismatches. All combinations of mismatch positions are enumerated up until a value m_max and stored as our input X. For an initial set of hash functions, $\{H_i\}$, LSH is performed on the entirety of X. The fraction of recovered sequences via LSH as a function of the number of mismatches, m, is stored as R. An energy function of the form $\exp(-R / T)$ is used for MCMC to maximize R as a function of $\{H_i\}$. Optimal hash functions can be obtained and then stored for use. Perfect hash functions are obtained when $R = 1$ at the end of optimization. Not all combinations of parameters will result in perfect hash functions.

Proteins are assigned random numeric ids at the start. Next all protein amino acid sequences are first converted to a reduced alphabet that groups related amino acids together. The reduced alphabet has 13 tokens, which allows each token to be represented by 4 bits, allowing for more space efficient representations than using a full char (8 bits). The map between tokens and amino acids are shown below:

[(0, ['T', 'A', 'S']), (1, ['L', 'M']), (2, ['I', 'V']), (3, ['R', 'K']), (4, ['Q', 'E']), (5, ['D', 'N']), (6, ['Y', 'F']), (7, ['W']), (8, ['C']), (9, ['G']), (10, ['H']), (11, ['P']), (12, ['U', 'O', 'J', 'Z', 'X', 'B', '*'])]

Kmers of length k are then generated for each protein, while excluding any kmers that contain amino acid stop codons ('*') characters. Kmers are stored in compressed format as integers when performing joins and merges to reduce space usage. Compressed formats are calculated efficiently using a rolling calculation with bitshifting. Each reduced amino acid token is stored as 4 bits in the final representation, and the bits are stored in an 8 byte integer. Calculations comparing kmers and number of mismatches between kmers are performed directly on the bit representations when possible. To assist with reducing noise, for each kmer, we additionally add a paired kmer of the same size that is equal to the length k sequence immediately upstream from the kmer, which we refer to as the paired kmer (kmer_pre). Using the paired kmer mode is optional.

Next, we iterate over all L hash functions, starting with $i=0$. All kmers (represented as integers) are mapped to their respective hash values using the hash function H_i. Hash values are matched. Buckets with more than 1 kmer are retained. Two representative protein ids

(rep_proteins) are then selected for each bucket: L_rep, which is the protein id corresponding to the longest sequence in the bucket (using protein id as a tiebreaker), and H_rep, which is the protein with the largest protein id (which was randomly selected at the start), using length (in descending order) as a tiebreaker. All proteins in the bucket are then paired to each of the two representative protein ids. For each protein/rep_protein pair, if cov * cov_approx_factor * length(rep_seq) > length(protein), then remove the pair from further consideration. If paired kmer mode is used, then additionally, the paired kmers between the protein and the rep_protein are compared according to their hamming distances as computed from their bit representations. If the hamming distance divided by k is less than the min_seq_id, we discard the pair from future consideration. All remaining pairs are merged into a persistent bucketed table, and i is incremented from 0 to $L-1$. The persistent bucketed table is maintained across all iterations and holds all pairs of sequences to be aligned. The bucketing is performed on the join keys during save to prevent complete repartitioning of both the persistent and update table at each iteration, drastically reducing runtime. The maximum number of pairs in the table will be $K * N * L$, where K is the maximum sequence length. The iterations can be performed in batches as memory and disk allows.

After all iterations have been completed, the final persistent table is used to generate alignments. Due to the massive size of the table, the task of creating alignments is split further into A iterations, where A is determined by the size of the persistent table as well as the cluster resources so as to prevent consuming the entire storage of the cluster. For each of the A iterations, $j=0,...,A$, all rep_seq ids with a modulo equal to j are considered for alignment. Then all passing protein/rep_protein pairs are materialized using a join to a database containing the original sequences. The sequence identity between each protein and the rep_protein sequences are then determined according to the user's choice of 1) a fast edit distance based estimate of sequence identity or 2) a slower but more Smith-Waterman accurate local sequence alignment using a Blosum62 scoring matrix with a gap open penalty of 11 and a gap extension penalty of 1 (which can be adjusted if desired). Minimum sequence coverage and minimum sequence identity are then enforced according to the clustering criterion min_seq_id and cov, resulting in a set of protein/rep_protein pairs that satisfy the clustering criteria. After all A iterations have been performed and their results merged, the next step is the optional graph simplification.

The resulting set of remaining protein/rep_protein pairs then form the basis for a directed graph. Graph simplification is essential for reducing long chains in the graph that result from deep homology within a family or fold of proteins. These long chains can result in very large communities detected by connected components and is thus important when clustering at low sequence identity and while using connected components for community detection. Graph simplification may not be necessary if other community detection algorithms are used.

The main graph simplification step works by clustering together nodes that are closely connected, in a way that preserves the overall structure of the graph. The first step is to create a new table of nodes and their degrees, where the degree of a node is the number of edges that it is connected to. Next, we group the edges by their destination node and aggregate the information for each node in a list of (degree, source) tuples. The winning edge for each node is then determined as the edge with the highest degree and smallest source node.

The next step is to determine the winning edges for each source and destination node. This is done by ranking the edges for each node based on their winning degree and then selecting the top-ranked edge. Finally, the winning edges for the source nodes are combined with the

winning edges for the destination nodes to form the final set of edges for the simplified graph. Any edges that connect a node to itself are filtered out.

After the graph simplification, a distributed connected components algorithm is performed on the graph to identify communities. Within each community, all sequences are realigned to the representative protein. Extremely large communities are processed separately to avoid skew times due to allocation to a single CPU.

The node with the max original degree in the graph (prior to simplification) within each community is selected as the megarep node. All nodes within the community that have not been aligned to the megarep node are realigned to the megarep node, forming new edges as required. For each cluster, all nodes are then sorted by the number of edges it is connected to within the cluster. The list of sorted nodes is then traversed and for each node visited that has not been assigned to a cluster, a new cluster is created consisting of all nodes that it is connected to that has not been clustered, along with itself. The final list of clusters are then combined across all communities to form the final clustering of the entire dataset. Numeric protein ids were then reassigned back to their original ids using a join.

Clustering comparison

UniRef100, UniRef90, and UniRef50 were downloaded from UniProt on 2022-04-18 for comparing clustering methods. Clustering was performed either on UniRef90 or UniRef50 using multiple software packages as possible to compare their clustering results. For timing comparisons, different clustering software were tested on a OpenMPI/Hadoop cluster with two 64 CPU nodes (each of type n1-highmem64 from Google Cloud with 512 GB of memory and 2TB SSD disk each) against various random samples of differing size from UniRef50 to test their runtimes as a function of the number of input sequences. linearithmic models were fitted to linearithmic scaling functions ($N \log N$) using a least squares fit with heteroskedastic error model where the expected variance scales with the square of the number of input proteins, while also forcing the constant and linear term to be non-negative. Quadratic models were fitted in the same manner for quadratic algorithms but using a quadratic model in place of a linearithmic model. The Nelder-Mead optimization algorithm was used to obtain minima.

For each 1M sample of UniRef50 (for 30 or 25% sequence identity clusterings) (or UniRef90 for 50% sequence identity clusterings, or UniRef100 for 90% squence identity clusterings), as well as for the full UniRef50 dataset, the following was performed. All sequences were searched against the entire sample on a 160 CPU machine with 3.8TB shared memory using MMSeqs2 with sensitivity of 7.5, a minimum coverage of 0.8, and maximum 100 sequences per query, with the --realign mode. An exception was made for the full UniRef50 dataset, in which case only a 1M sample was selected. For each query, the top 100 hits were then realigned to the query using the standard Smith-Waterman algorithm with a Blosum62 matrix, a gap open penalty of 11 and a gap extension penalty of 1. If the number of aligned residues in the query divided by the length of the query was less than the min_cov of 0.8, then the query/target pair was ignored. Similarly, if the number of aligned residues in the target divided by the length of the target was less than the min_cov of 0.8, then the query/target pair was ignored. Of the passing query/target pairs, the target with the maximum sequence identity was selected to be the nearest neighbor. For each clustering result, the following was computed for each range of sequence identities (20-25%, 25-30%, … 95-100%): the percentage of proteins that are clustered (that is the percentage of proteins that has a nearest neighbor within the given sequence identity range while also being found in a non-singleton cluster (cluster with size ≥ 2). The cumulative

(from 100% to 20% sequence identity) percentage of proteins clustered was also obtained by multiplying each sequence identity range by the number of proteins in the range and then summing (starting from the largest sequence identity range, 95-100%, and ending with the smallest sequence identity range, 20-25%), dividing by the total number of proteins in the input dataset.

Inter cluster distances were also analyzed using the following approach. For each clustering method, a random sample of 5000 cluster representatives from the resulting clustering was taken and aligned against all cluster representatives from the clustering using the above BLOSUM62 Smith-Waterman alignment strategy. For each sampled cluster representative, the sequence identity to the nearest (non-self) neighboring cluster representative was then taken to be sequence identity to the nearest cluster. A cumulative distribution (starting from 100% going own to 20% was then generated on the quantity: sequence identity to the nearest cluster over the sample of 5000 cluster representatives. This analysis provided a comparison for how well a given clustering method generated clusters that are sufficiently far apart for the given target clustering threshold.

A power analysis of cluster sizes was also performed as a function of the input dataset size (based on the UniRef50 samples). The distribution of cluster size was plotted on a log-log scale for the resulting clustering from each of UniRef50 samples.

FLSHclust scaling as a function of number of nodes was computed by progressively scaling the number of compute noes and testing for time of completion versus the 3160k sample UniRef50 dataset. Relative completion time was taken to be the time for completion at N nodes divided by time for completion using 1 node. Computational efficiency for each value of number of nodes (1, 2, 4, 8, or 16) was computed using the standard formula, which is the speedup (inverse of relative completion time) divided by the number of nodes.

<u>CRONUS CRISPR repeat tool</u>

CRONUS was implemented with python 3 and operates as follows. For main parameters, CRONUS uses the minimum number of repeats - min_repeats (default 3), the minimum repeat length - min_len (default 15), the minimum sequence identity between repeats - min_id (default 85), the maximal size of the direct repeats - max_repeat_sz (default 200), the maximum spacer length - max_spacer_len (default 200), the seed size of the initial search - seed_size (default 5), the maximum number of repeat types per array - max_repeat_types (default 2). A general description of the program is given below.

First, vmatch2 is run with the query (len N) against itself with a seed length seed_size and a min identity min_id and a max edit distance based on the min_len, min_id, and seed_size. A sparse NxN coverage matrix was then formed containing all of the pairwise matches from vmatch2 with max genomic distance of 400. Connected components were then obtained from treating the coverage matrix as an adjacency matrix. Clusters of related sequences were determined by the connected components algorithm. Match groups were formed based on the connected components, consisting of a sequence along with all of its matches if it belongs to a connected component. For each match group the following was performed: A focus region was defined as the entire region spanning the sequences found in the component plus an additional 200 bp on either side. Next, length 5 kmers from all sequences covered by the connected component are grouped into a list to serve as passable kmers. All kmers of length 5 in the focus region were then identified and counted, provided that they exist in the list of passable kmers. Kmers with a count of 3 or more were included for further processing. For each kmer, the

positions in the focus were taken into a list *d* in sorted order. If *med_len*, the median difference in adjacent positions (called *diff*) in *d* was below 15, the kmer was skipped. The spacing regularity was defined as mean(abs(*diff* - med_len) % *med_len*), where % is the modulo operator. The normalized regularity was equal to the spacing regularity divided by the *med_len*. If the normalized regularity was > 0.35 or if *med_len* >= 300, then the kmers were discarded. For the remaining kmers, if any of them overlap with the positions of the initial matches from the match group, the match was retained. If the final number of matches was less than min_repeats, then the entire match group was skipped. Next, the distances between all remaining matches were calculated. If the 80th percentile of the distance between matches (defined as soft_max_neigh_dist) is greater than 2 * max_spacer_len, then the entire match group was skipped. Next, the match group was filtered to remove deviating matches. Specifically, if a match on either end of the match group is more than 1.5 * soft_max_neigh_dist from the rest of the list, it is removed. This is repeated until no more matches are removed.

Next, we construct a list of start and end positions for each putative DR, as determined by the current match group. We then sort these positions by their starting location. Next, we apply a merging step to combine overlapping DRs into longer contiguous regions. This is done by checking if two adjacent DRs have an overlap of at least overlap_buffer base pairs, and if so, merging them together. After the DRs have been identified and merged, we align the putative DR sequences to each other and process the alignment to determine which DRs should be extended to improve the alignment. DRs are extended if they have a critical threshold of mismatches or gaps compared to the other DRs in the alignment. The extended DRs are then re-aligned with the other DRs, and the process is repeated until either no DRs need to be extended or the maximum number of iterations is reached. Using these alignments of the putative DRs, the DRs are further refined. The full alignment is converted into an embedding matrix, *E* of size N * 5*M, where N is the number of sequences and M is the alignment length, by setting E[i, j+5*f(x[i,j])] = 1 / M for each i,j, and 0 otherwise, where f sends 'A' to 0, 'T' to 1, 'G' to 2, 'C' to 3 and '-' to 4. Lastly, clustering is performed using DBSCAN with the dbscan function from scikit-learn. The parameters used are eps=0.55 and p=1. The eps parameter determines the maximum distance between two samples for them to be considered as in the same neighborhood. The p parameter is the power parameter for the Minkowski metric used by DBSCAN. Using the DBSCAN clusters, the repeat boundaries are refined while considering sequence divergence. The spacer regions are then tested for minimum variability.

To limit the extent of all-to-all comparisons within each contig, contigs are split into 20kb overlapping tiles (with 13334 bp overlaps) and CRONUS is run on each split with resulting overlapping CRISPR arrays deduplicated by prioritizing the longest arrays followed by the arrays with the smallest start site.

Performance comparison of CRISPR finders on synthetic CRISPR array benchmark

A synthetic benchmark was constructed using parameterized probability distributions over CRISPR arrays. These CRISPR arrays were then embedded in totally random nucleotide sequences and then used as inputs for the various CRISPR finders along with different parameter combinations for the CRISPR finders. Tasks were parallelized using Apache Spark on a compute cluster with 6400 CPUs.

Synthetic CRISPRs were generated according to the following process. We start by setting the seed for the random number generator using the "trial" value. Then, we generate a consensus sequence for the direct repeat (DR) by generating a random sequence of canonical

bases (A, C, G, T) of length equal to the prespecified DR length. We also define a range for the DR size that is equal to [floor(dr_len * (1 - rel_indel_range)), ceil(dr_len * (1 + rel_indel_range))]. Similarly, we define a range for the spacer size that takes into account the spacer indel relative range, equal to [floor(dr_len * (1 - spacer_rel_indel_range)), ceil(dr_len * (1 + spacer_rel_indel_range))].

Next, we create empty lists to hold the DR and spacer sequences generated in the loop. We iterate "n_dr" times to generate the DR sequences. For each DR, we start with the consensus sequence and perform mismatches by randomly selecting a base that is different from the consensus base with a probability of "p_mismatch". We then check if an indel should be performed by randomly selecting a value between the defined range for the DR size and comparing it to the original length. If an indel should be performed, we randomly select the position(s) and type of indel(s) to be made. If an insertion is made, we insert a randomly chosen base at the selected position(s). If a deletion is made, we remove the base(s) at the selected position(s). The resulting DR sequence is added to the list.

Similarly, we iterate "n_dr-1" times to generate the spacer sequences between the DRs. For each spacer, we start with the consensus spacer sequence and perform mismatches with a probability of "1 - p_spacer_similarity". This allows control over the level of similarity between spacer sequences. For all tests used, p_spacer_similarity is selected to be 0.3, resulting in highly differing spacer sequences. We then check if an indel should be performed by randomly selecting a value between the defined range for the spacer size and comparing it to the original length. If an indel should be performed, we randomly select the position(s) and type of indel(s) to be made. If an insertion is made, we insert a randomly chosen base at the selected position(s). If a deletion is made, we remove the base(s) at the selected position(s). The resulting spacer sequence is added to the list.

The final CRISPR array is formed by interleaving the spacers into the DR sequences and then concatenating the full array into a single sequence. Then, we generate left and right flanking sequences to be added to the CRISPR sequence. Then we generate a random DNA sequence of length 20000 and insert the synthetic CRISPR array at the randomly selected position in the sequence.

For each CRISPR parameter set of the 35 selected combinations, 2000 random synthetic CRISPRs embedded in random DNA sequences were created using the above procedure, and all CRISPR predictors with selected corresponding parameters were tested against the synthetic sequences, keeping track of time elapsed.

To understand the CRISPR performance of each of the finders, we compiled scores for each CRISPR finder / CRISPR parameter set (condition group) pair as follows. We defined the consensus sequence of a list of DRs to be the DR with the minimum average normalized edit distance to the remaining DRs, where the normalized edit distance is defined as the edit distance between DR_i and DR_j divided by the minimum length of the two DRs (DR_i, DR_j). We then calculated 3 simple metrics of performance: “hit” rate, “DR count error” and “boundary position error” (indels between predicted and true DR). For each synthetic CRISPR, a predicted CRISPR was considered a “hit” if it overlaps with the synthetic CRISPR interval (expanded by a 50 bp buffer). If a hit was found, then we aligned the consensus of the synthetic DRs with the consensus of the predicted DRs using Biopython pairwise2 alignment module and calculated the number of gap columns in the alignment as the boundary prediction error (in bp). The absolute value difference between the number of true DRs and predicted DRs was taken to be the DR count error. We then used bootstrapping with 2000 samples to calculate the mean, upper quartile,

and lower quartile of the means of all three performance metrics as well as elapsed time in order to compare between all CRISPR predictors.

Prokaryotic genome/metagenome CDS prediction and clustering

We used Prodigal (*76*) to predict all genes in the genomes/metagenomes, using the metagenomic option for metagenomic datasets (WGS metagenomes, JGI, MG-RAST). Next we deduplicated all proteins, then iteratively clustered all proteins at 100%, 98%, 90%, 70%, 50%, and 30% sequence identities with a minimum coverage of 80%. For 100%-50% sequence identity, we clustered using the FLSHclust implementation without the use of LSH functions and used the LSH functions for clustering from 50% to 30% sequence identity. All 6 clusterings along with deduplication were performed with the FLSHclust implementation over the course of ~1-2 weeks with intermittent breaks on a Hadoop cluster containing 30 compute nodes with 32CPUs, 256 GB memory and 2TB SSD non-shared storage each.

Sensitive CRISPR discovery pipeline

For CRISPR prediction, 4 CRISPR finders were used with a total of 6 different runs based on parameter combinations selected from a calibration against the synthetic CRISPR array benchmark. Parameters for each CRISPR finder are as follows:

1. PILERCR with minarray=3, mincons=0.9, minid=0.94, maxrepeat=64, maxspacer=64 (codename PILER_1)
2. PILERCR with minarray=3, mincons=0.8, minid=0.85, maxrepeat=128, maxspacer=128 (codename PILER_5)
3. CRT with minNR=3, minRL=16, maxRL=128, minSL=16, maxSL=128 (codename CRT_2)
4. CRISPRFinder with mNS=2 (codename CRISPRFinder)
5. CRONUS with seed_size=5, min_len=16, minid=8, max_repeat_size=128 (codename CRONUS_2)
6. CRONUS with seed_size=11, min_len=16, minid=80, max_repeat_size=512 (codename CRONUS_4)

Next, we deduplicated CRISPR array predictions from the various CRISPR finders as follows. For each contig, we create an empty list of intervals. We loop through each CRISPR and check if it overlaps with any of the existing intervals. If it does, we merge the intervals and add the CRISPR to the corresponding list of CRISPRs in the merged interval. If it doesn't overlap with any existing intervals, we create a new interval for the CRISPR.

Finally, we select the best CRISPR from each interval by selecting the CRISPR with the most DRs, highest priority index, longest length, and earliest start position. Here, the priority index of the various CRISPR finders is determined according to the following order: ['CRISPRFinder', 'PILER_5', 'PILER_1', 'CRT_2', 'CRONUS_4', 'CRONUS_2']. For consistency across the CRISPR arrays, we then redefine the spacers to be the sequences interleaving the predicted DRs as not all CRISPR prediction tools may do this.

We defined the distance (interval_distance) between two genomic feature intervals (e.g. a CRISPR array, or CDS) to be equal to zero if the two intervals overlap, and otherwise the minimum distance between all combinations of the endpoints. We further corrected this distance

for circular contigs (e.g. plasmids and some phage, entire bacterial genomes) by using the nearest distance along a circle as opposed to a line when necessary.

We then used the following procedure to define operons from the set of predicted proteins within a given contig. We create two lists called "proteins_f" and "proteins_r" to store proteins in forward and reverse directions, respectively. We then sort the proteins in both lists based on their starting position for forward proteins and ending position for reverse proteins. We create an empty list called "intervals_f" to store forward protein operons. We loop through each forward protein in "proteins_f" and get its starting and ending positions. If the "intervals_f" list is empty, we create a new interval with the protein and append it to the list. Otherwise, we check if the current protein is within a distance of 200bp to the last interval in the list. If it is, we add the protein to the last operon and update its starting and ending positions to encompass all proteins currently in the operon. If it is not, we create a new interval with the protein and append it to the list. If the DNA strand is circular and there is more than one interval in the "intervals_f" list, we check if the first and last intervals are within the maximum distance of each other. If they are, we merge them into a single interval that crosses the circular boundary. We repeat the above process for the reverse proteins and store the resulting intervals in a list called "intervals_r". The final set of operons is defined by the concatenation of "intervals_f" and "intervals_r"

We then define the operonic distance between a protein and another feature of interest as the interval_distance between the feature of interest and the operon containing the protein. We then computed the operonic distance between each protein and the nearest CRISPR array. For this study, we set max_association_distance = 3000 to be the maximum operonic distance between a protein gene and a CRISPR array at which the protein gene is considered to be associated with a CRISPR array. For each gene, the associated CRISPR array is the CRISPR array closest to the protein within max_association_distance operonic distance from the protein, or an empty array if none exists.

Metagenomic data poses a challenge in inferring associations due to the tendency for short contigs to contain partial systems. However, the information from metagenomic data can still be leveraged for calculating associations by calculating an effective sample size for each protein that incorporates information about distances to the contig edge. For each protein, two quantities are computed, "u_d" and "d_d" which are the upstream and downstream distances to the contig edge from the operon containing the protein. Upstream and downstream are determined relative to the orientation of the protein on the contig. The contig weight for the protein is then computed as the weighted average w = 0.5 * min(1, u_d / max_association_distance) + 0.5 * min(1, d_d / max_association_distance). If the protein is not associated with a CRISPR array (i.e. there is no CRISPR array with an operonic distance of less than 3000 to the protein), the effective sample size is the contig weight. If the protein is considered to be associated with a CRISPR array, then the effective sample size is equal to 1.

Proteins are then redundancy reduced as follows. All proteins within the same c90 cluster (90% identity cluster) are grouped. The proteins are then ordered in lexicographic order using the followed ordered criteria: 1) the number of DRs in the associated CRISPR array (descending order), 2) contig weight of the protein (descending order), 3) the protein id as a tiebreaker (descending order). The rank 1 protein from each c90 cluster as determined from the above ordering is then selected to be the representative protein for the c90 cluster with regards to association calculation. The resulting set of proteins is considered to be the redundancy reduced set for the naive score calculation.

The naive association score is then calculated as follows. For each c30 cluster (30% identity cluster), three quantities were computed using all the non-redundant proteins (c90 cluster representatives defined above) contained within the 30% cluster: the number of non-redundant proteins with CRISPR associations (N_cr), and the sum of the effective sample sizes of all non-redundant proteins (N_eff), as well as the total number of non-redundant proteins (N). The naive score (referred to as weighted_icity) was then calculated to be S = N_cr / N_eff.

All 30% clusters with (weighted_icity >= 0.02 and N_cr >= 2) or (weighted_icity >= 0.1 and N_cr >= 1) were selected for performing blast searches of the respective DRs in order to calculate the enhanced score. Therefore, extremely low confidence clusters were dropped to avoid performing excessive blast searches. The remaining 30% clusters were considered candidate 30% clusters.

We then searched for divergent DRs for the candidate 30% clusters as follows. All non-redundant 90% clusters were assigned to the consensus DR of the closest CRISPR array of the representative cluster member, which were selected as above. Then, for each of the 30% clusters, the consensus DRs of all 90% clusters were collected into a list. For each protein belonging to the candidate 30% clusters, the 10kb vicinity was searched against all of the consensus DRs from the 30% cluster using an expect value of 1e-3 (with the db size equal to the 10kbp window around the protein, accounting for reduced size when necessary due to short contigs), and a word size of 7, a gap open penalty of 6, and a gap extension penalty of 2. We additionally required a minimum coverage of 0.4 to the DR to be considered a divergent DR, that is match length / DR length was required to be >= 0.4. Furthermore, we required that all matches be >= 16bp to reduce noise. Matches with an operonic distance of 3kbp or more were discarded. All remaining matches were considered divergent DRs (alternatively referred to as “searched DRs”). Enhanced CRISPR scores were then calculated in the same manner as the naive score, but using the “searched DRs” in place of the CRISPR arrays when computing all quantities, namely presence of CRISPR within the vicinity (“searched DRs” within 3kbp of the protein) and effective sample size, with the caveat that only “searched DRs” for a given 30% cluster were considered for scoring that cluster (i.e. “searched DRs” for cluster *i* would not be used to count towards the scores for cluster *j*).

Protein-protein associations were then computed using same weighted_icity method as above except with a prespecified cluster id (c30_id) in place of CRISPR arrays and an association distance of 10kb in place of 3kb. Each cluster (c30_id) from the above analysis was used, providing specific associations between other genes and newly identified candidate CRISPR-associated genes.

For selecting an appropriate threshold for the CRISPR association score, we selected a set of Cas and Non-Cas genes. For the Cas genes, we selected Cas9, Cas12, Cas13, Cas7, Csf2, and Cas7_TypeIII (profiles described below in Appearance of CRISPR systems in the public dataset). We took all c98_id clusters (clusters at 98% sequence identity) and checked if any had a hit to these profiles using HMMER with an e-value cutoff of 1e-50 (as determined by searching against the complete set of Cas profiles). A c30_id was considered then to be a Cas cluster (for this analysis) if any 98% cluster had a hit to one of the above Cas profiles. For the Non-Cas genes, the same was performed, except with a more permissive cutoff of 1e-5 and also only using the following PFAM profiles, which were determined to be in general unrelated to CRISPR:
'PF04313', # HSDR_N_1
'PF00144', # Beta-lactamase
'PF00665', # RVE (transposases)

'PF10145', # Phage tail minor protein
'PF00437', # Type II/IV SS
'PF00085', # Thioredoxin
'PF01841', # Transglutaminase
'PF00313', # Cold shock domain
'PF07508', # Recombinase
'PF01765', # Ribosome recycling factor

Pipeline comparisons

Various pipelines that estimate CRISPR association score were all run using a minimum N_eff of 3 and a minimum calculated score of 0.35. The pipelines considered were the full pipeline, and pipelines using the simple weighted score either with 50% clusters or 30% clusters (as produced by FLSHclust) with either all predicted CRISPRs or only those predicted by the CRT_2 condition. The number of passing clusters from each were reported, as well as the number of overlaps between the passing clusters and the full set of 187 clusters corresponding to the 187 identified systems (excluding PhageCas9 system due to this system being identified for having a score of 0).

Appearance of CRISPR systems in the public dataset

All projects from NCBI, JGI, WGS, and EMBL were mapped to their project add dates whenever possible. Projects that could not be matched were discarded from analysis. All predicted proteins were then assigned a date based on the add date of the corresponding project. The appearance of a cluster in the public data was defined as follows. All proteins with an upstream or downstream distance to the contig edge of < 500bp were discarded due to their low quality and likely presence of protein truncations from having incomplete contigs. Then all proteins with a distance of <= 10000 bp to a predicted CRISPR array were retained. For each 90% cluster, the minimum add date of all proteins within the cluster was taken to be the appearance date of the 90% cluster (referred to as non-redundant protein). This step constitutes the redundancy reduction step for CRISPR-associated non-redundant proteins in the calculation. Afterward, for each 30% cluster, all of the CRISPR-associated non-redundant proteins were obtained, and their appearance dates sorted. The date of the 2nd row in the sorted list was designated the appearance date of the cluster in the public data (corresponding to the minimum date at which 2 or more CRISPR-associated non-redundant proteins existed in the public data. The appearance date of a given CRISPR system was then defined as the minimum appearance date of all clusters from the system, as defined by a given signature gene. For the PhageCas9 system, which was not associated with CRISPR arrays, we disregarded the requirement that the loci are CRISPR associated when identifying loci for the appearance date calculation.

The signature genes used for defining the system are as follows: VII: b-CASP, Cas12+DUF3800: DUF3800-TPR, Cas5-HNH: Cas5-HNH, Cas8-HNH: Cas8-HNH, DinG-HNH: DinG-HNH, CasMu-V: Cas12, CasMu-I: CasMuC, Cas12+IscB: IscB, Cas12+Cas3: Cas12, PhageCas9: Cas9, Cas8-Cas3: Cas8-Cas3, Cas5-Cas3: Cas5-Cas3, Cas11-VRR: Cas11-VRR, gRAMP: gRAMP, Cas13bt: Cas13bt.

For the known CRISPR genes, the following combinations of HMMER profiles were used per system, where KOON indicates the profile were taken from (*9*), and when indicated from (*77*):

```
CARF = ['KOON_icity0089',
'KOON_icity0091',
'KOON_icity0093',
'KOON_icity0095',
'KOON_icity0100',
'KOON_icity0102',
'KOON_icity0103',
'KOON_icity0104',
'KOON_icity0119',
'KOON_icity0120',
'KOON_icity0105',
'KOON_cls000179',
'KOON_COG0640',
'KOON_cd09694',
'KOON_cd09699',
'KOON_cd09742',
'KOON_cd09746',
'KOON_COG1517',
'KOON_COG4006',
'KOON_pfam09651',
'KOON_pfam09659',
'KOON_cd09660',
'KOON_cd09668',
'KOON_cd09671',
'KOON_cd09686',
'KOON_cd09702',
'KOON_cd09723',
'KOON_cd09728',
'KOON_cd09732',
'KOON_cd09741',
'KOON_cd09747',
'KOON_cls000955',
'KOON_cls001403',
'KOON_mkCas0085',
'KOON_mkCas0108',
'KOON_mkCas0109',
'KOON_mkCas0112',
'KOON_pfam09002',
'KOON_pfam09455',
'KOON_pfam09623',
'KOON_pfam09670',]

Cas1 = ['KOON_COG1518',
 'KOON_cd09634',
 'KOON_cd09636',
 'KOON_cd09718',
```

```
 'KOON_cd09719',
 'KOON_cd09720',
 'KOON_cd09721',
 'KOON_cd09722',
 'KOON_icity0124',
 'KOON_pfam01867',
 'TIGR00287',
 'TIGR03637',
 'TIGR03638',
 'TIGR03639',
 'TIGR03640',
 'TIGR03641',
 'TIGR03983',
 'TIGR04093',
 'TIGR04329',
 'cas1',  # From CRISPRDisco
 'cas1_updated' # FROM CRISPRDisco
]

Cas2 = ['KOON_COG1343',
 'KOON_COG3512',
 'KOON_cd09638',
 'KOON_cd09648',
 'KOON_cd09725',
 'KOON_cd09755',
 'KOON_icity0022',
 'KOON_mkCas0081',
 'KOON_mkCas0128',
 'KOON_mkCas0179',
 'KOON_mkCas0206',
 'KOON_pfam09707',
 'KOON_pfam09827',
 'TIGR01573',
 'TIGR01873',
 'cas2', # From CRISPRDisco
]

Cas4 = ['KOON_COG1468',
 'KOON_COG4343',
 'KOON_cd09637',
 'KOON_cd09659',
 'KOON_cls000170',
 'KOON_pfam01930',
 'KOON_pfam06023',
 'TIGR00372',
 'TIGR01896',
```

```
 'TIGR04328',
 'cas4' # From CRISPRDisco
]

Cas6 = 'KOON_COG1583',
 'KOON_COG5551',
 'KOON_cd09652',
 'KOON_cd09664',
 'KOON_cd09674',
 'KOON_cd09703',
 'KOON_cd09727',
 'KOON_cd09733',
 'KOON_cd09739',
 'KOON_cd09759',
 'KOON_cd09760',
 'KOON_cls000004',
 'KOON_cls000976',
 'KOON_icity0019',
 'KOON_icity0020',
 'KOON_icity0025',
 'KOON_icity0026',
 'KOON_icity0028',
 'KOON_mkCas0062',
 'KOON_mkCas0066',
 'KOON_mkCas0091',
 'KOON_mkCas0166',
 'KOON_pfam01881',
 'KOON_pfam08798',
 'KOON_pfam09559',
 'KOON_pfam09618',
 'KOON_pfam10040',
 'TIGR01877',
 'TIGR01907',
 'TIGR02563',
 'TIGR02807',
 'cas6' # From CRISPRDisco
]

Cas9 = ['cas9', # From CRISPRDisco
 'TIGR01865',
 'TIGR03031',
 'KOON_cd09643',
 'KOON_cd09704',
 'KOON_COG3513',
 'KOON_icity0002',
 'KOON_mkCas0193'
```

```
]

Cas12a = ['KOON_Cas12a','KOON_Cas12a_var']
Cas12b = ['KOON_Cas12b', 'KOON_Cas12b2']
Cas12c = ['KOON_Cas12c', 'KOON_Cas12c_arbor_variant']
Cas12d = ['KOON_Cas12d']
Cas12e = ['KOON_Cas12e', 'KOON_Cas12e_var'],
Cas12=[
'KOON_Cas14e','KOON_Cas14f','KOON_Cas12a','KOON_Cas12a_var','KOON_Cas12b','KOO
N_Cas12b2','KOON_Cas14g','KOON_Cas12c','KOON_Cas12c_arbor_variant','KOON_Cas12d',
'KOON_Cas12e','KOON_Cas12e_var','KOON_Cas14a','KOON_Cas14b','KOON_V_U3','KOO
N_Cas14c','KOON_Cas14u','KOON_V_U4','KOON_V_U2','KOON_V_U1','KOON_V_U5','KO
ON_Cas14d','KOON_Cas14h','KOON_Cas12g','KOON_Cas12h','KOON_Cas12i','cas12a','cas12
b','cas12c','cas12d']

Cas13a = ['KOON_Fish20601',
'cas13a' # From CRISPRDisco
]

Cas12b = ['KOON_Fish20602',
'KOON_Fish20602a',
'cas13b' # From CRISPRDisco
]

Cas13c = ['KOON_Fish20603',
'cas13c' # From CRISPRDisco
]

Cas13d = ['KOON_Cas13d',
'cas13d' # From CRISPRDisco
]

Cas7 = ['KOON_COG1857',
 'KOON_COG3649',
 'KOON_cd09640',
 'KOON_cd09646',
 'KOON_cd09650',
 'KOON_cd09677',
 'KOON_cd09678',
 'KOON_cd09685',
 'KOON_cd09687',
 'KOON_cd09689',
 'KOON_cd09690',
 'KOON_cd09717',
 'KOON_cd09737',
 'KOON_cd09738',
```

```
 'KOON_cls000038',
 'KOON_cls000079',
 'KOON_mkCas0088',
 'KOON_pfam01905',
 'KOON_pfam05107',
 'KOON_pfam09344',
 'KOON_pfam09615'
]

Cas7_TypeIII = ['KOON_icity0012',
 'KOON_cd09662',
 'KOON_icity0009',
 'KOON_mkCas0142',
 'KOON_cls000184',
 'KOON_cls000253',
 'KOON_cd09657',
 'KOON_cls000708',
 'KOON_mkCas0198',
 'KOON_COG1332',
 'KOON_COG1336',
 'KOON_icity0013',
 'KOON_cd09683',
 'KOON_mkCas0086',
 'KOON_pfam03787',
 'KOON_cls001827',
 'KOON_mkCas0182',
 'KOON_COG1604',
 'KOON_cd09709',
 'KOON_cls000670',
 'KOON_mkCas0143',
 'KOON_icity0006',
 'KOON_COG1367',
 'KOON_pfam09617',
 'KOON_COG1337',
 'KOON_mkCas0095',
 'KOON_mkCas0175',
 'KOON_cls000690',
 'KOON_cd09661',
 'KOON_cd09726',
 'KOON_cd09682',
 'KOON_cd09684',
 'KOON_mkCas0102']

Csf1 = ['KOON_cd09705',  'KOON_icity0010',  'KOON_mkCas0068',  'KOON_mkCas0185',
'KOON_mkCas0210']
```

Csf2 = ['KOON_cd09706', 'KOON_mkCas0162', 'KOON_mkCas0183']

Csf3 = ['KOON_cd09707', 'KOON_mkCas0093']

The signature genes used for the appearance data calculation were as follows: Type I: Cas7, Type II: Cas9, Type III: Cas7_TypeIII, Type IV: Csf2, Type V: Cas12, Type VI: Cas13. For Type V, V-F was excluded due to profile overlap with TnpB, while V-H and V-E were excluded because they were identified from non-public data. The following concerns curation of the c30_ids that are associated with each broad CRISPR-Cas designation. First, to predict gene families, HMMER search was performed using all profiles against each of the 98% cluster representatives with a maximum e-value score of 1e-10, assigning each gene to the profile with the lowest e-value. For each profile set (e.g. Cas9, Cas1…), all 98% cluster centers were considered "hits" if the representative protein matched any profile in the profile set and fit within the size range, assigned as follows: Cas1: (150, 400) , Cas2: (50, 200), Cas4: (200, 500), Cas6: (150, 500), Cas7: (200, 400), Cas7_TypeIII: (150, 400), Csf2: (150, 400), Csf1/3: (1500, 1000), Cas9: (700, 2200), Cas12: (500, 2500), Cas12 subtypes (500, 2000), Cas13: (700, 2000), Csn2: (50, 400). The mean number of passing "hits" per 90% cluster was then aggregated. 90% clusters were considered "passing" if 50% or more of the 98% clusters in the 90% cluster was a "hit". A 30% cluster was then considered a "match" to the signature gene of interest if 10% or more of the 90% clusters in the 30% cluster were considered "passing." For each CRISPR system, the corresponding set of "matches" for the signature gene was used to calculate the appearance date as above. For the general calculation of all type V related clusters, an additional requiring either protein size to be ≥ 800aa or have a weighted CRISPR-association score of ≥ 0.25 to avoid TnpB contamination. V-D and V-C profiles overlapped heavily and were thus combined into one designation

Spacer search for selected CRISPR systems

CRISPR spacers were obtained from predicted CRISPR arrays, including the left and right cryptic "spacers" outside of the array by extending 30bp past the first and last DRs. A consensus DR was obtained for each CRISPR array by taking the DR with the minimum average normalized edit distance to all the other DRs in the array, where we define normalized edit distance as the edit distance between DR_i and DR_j divided by the maximum length of DR_i and DR_j. Each spacer was assigned a representative DR equal to the consensus DR for the CRISPR array it originated from.

Spacers were then searched against the entire genomic/metagenomic database using BLASTN with an e-value cutoff of 1 and word size of 11, and effective database size of 8871099067954. The effective database size was used because the BLASTN search was performed by distributing the query identically over all nodes in the cluster and then using each cluster node to blast the query against different subsets of the full database, such that the full database was searched against across all nodes. Instead of an e-value cutoff, a bit score threshold of 48 was used as a minimum for a significant match. A threshold was selected as opposed to an e-value cutoff to reduce the effect of database size on the search, however, the database size still affected the number of candidate hits below the e-value cutoff of 1. For each query, all target matches were then expanded by 100bp in each direction. The representative DR for the spacer was then searched against the expanded region using BLASTN with a word size of 7. A target was then considered a self-match if any of the DR representative blast hits occurred within

100bp of the spacer target match with a minimum bit score of 24. Here, a self-match is a spacer match to a locus that is identical or similar to the original CRISPR array containing the spacer. All target matches with self-matches as defined above were removed. All remaining matches were considered significant matches to the spacer sequence that are considered unrelated to the original locus.

E. coli PAM discovery assay

100 ng each of one plasmid expressing the proteins and corresponding crRNA from the system of interest and one plasmid containing a target 8N degenerate flanking library plasmid were transformed by electroporation into 30 μL Endura Electrocompetent *E. coli* (Lucigen) as per the manufacturer's protocol, with 3 biological replicates per condition as well as 3 biological replicates of an empty control. After recovery by shaking at 37°C for 1 hour, cells were plated across one 22.7cm x 22.7 cm BioAssay plate with the appropriate antibiotic resistance and grown for 12-16 h at 37°C. Cells were scraped from the plate and mixed well, and 2 mL of the scraped cells were used as input to minipreps (Qiagen). 100 ng miniprepped plasmids were input to PCR to amplify the PAM-containing region with an 8-cycle PCR using NEBNext High Fidelity 2X PCR Master Mix (NEB) with an annealing temperature of 63°C, followed by a second 12-cycle PCR to further add Illumina adaptors and barcodes. Amplified libraries were gel extracted, quantified by a Qubit dsDNA HS assay (Thermo Fisher Scientific) and subject to single-end sequencing on an Illumina NextSeq with Read 1 75 cycles, Index 1 8 cycles and Index 2 8 cycles. PAMs were extracted and Weblogos depicting PAMs depleted 5 standard deviations relative to the empty control were visualized using Weblogo3.

Expression and purification of recombinant proteins

Cas5-HNH, Cas8-HNH and DinG-HNH effector RNP complexes: To purify the Cas5-HNH, Cas8-HNH and DinG-HNH-associated effector complexes in complex with their cognate crRNAs, the *E. coli* codon optimized operons were cloned into pET45b(+) backbone. A His14 tag was inserted at the N-terminus of the Cas11 gene for Cas5-HNH, the Cas8-HNH gene for Cas8-HNH and the Cas5 gene in the DinG-HNH-associated Cascade by Gibson assembly. The crRNAs were similarly cloned into a separate pCOLADuet-1 vector under control of a T7 promoter, and co-expressed with the cognate Cascade proteins in BL21(DE3) strain (NEB). Cells were grown at 37°C in autoinduction terrific broth (TB) medium (*78*) supplemented with 100 μg/ml ampicillin and 100 μg/ml kanamycin until reaching an optical density (OD600) of 0.4-0.6, then shifted to 18°C for overnight induction. The bacteria were harvested by centrifugation and the cell paste was resuspended in lysis buffer (50 mM Tris-HCl pH 8, 200 mM NaCl, 5% glycerol, 40 mM imidazole and 5 mM β-mercaptoethanol) supplemented with protease inhibitors (PMSF and Roche cOmplete, EDTA-free). The resuspended pellet was lysed by two passes with a high-pressure homogenizer (LM20 Microfluidizer, Microfluidics). The lysate was cleared by centrifugation, and the soluble fraction was mixed with Ni Sepharose 6 Fast Flow Affinity Chromatography Media (Cytiva) at 4°C. The resin was first washed with lysis buffer, then with 5 column volumes of buffer A (50 mM Tris-HCl pH 8, 1 M NaCl, 5% glycerol, 40 mM imidazole and 5 mM β-mercaptoethanol), and buffer B (50 mM Tris-HCl pH 8, 500 mM NaCl, 5% glycerol, 40 mM imidazole and 5 mM β-mercaptoethanol) on a gravity flow column. Bound RNP was then eluted in elution buffer (50 mM Tris-HCl pH 8, 500 mM NaCl, 5% glycerol, 300 mM imidazole and 5 mM β-mercaptoethanol). Eluted RNP was then dialyzed overnight into dialysis buffer (20 mM Tris-HCl pH 8, 250 mM NaCl, 5% glycerol and 1 mM DTT).

DinG-HNH protein: DinG-HNH protein was purified similarly to Cas5-HNH, Cas8-HNH and DinG-HNH-associated effector complexes with the following modifications: buffer A contained 50 mM Tris-HCl pH 8, 500 mM NaCl, 5% glycerol, 40 mM imidazole and 5 mM β-mercaptoethanol, buffer B was the same as lysis buffer, and elution buffer contained 20 mM Tris-HCl pH 8, 250 mM NaCl, 5% glycerol, 300 mM imidazole and 5 mM β-mercaptoethanol.

Candidate Type VII proteins and RNP complexes: To purify the candidate type VII Cas7/Cas5 RNP complex and Cas14 protein, cognate crRNAs were cloned into a pET45b(+) vector under control of a T7 promoter by Gibson assembly. The *E. coli* codon optimized operon was synthesized by Twist Biosciences and cloned into the crRNA-containing pET45b(+) backbone by Gibson assembly. A His14 tag was inserted at the N-terminus of the Cas5 gene and a TwinStrep tag was inserted at the N-terminus of the Cas14 gene by Gibson assembly. Proteins were expressed as described above using media supplemented only with 100 µg/ml ampicillin and cell paste was resuspended in His lysis buffer for Cas7/Cas5 RNP complexes (50 mM Tris-HCl pH 8, 250 mM NaCl, 5% glycerol, 40 mM imidazole and 5 mM β-mercaptoethanol) or Strep lysis buffer for Cas14 proteins (50 mM Tris-HCl pH 8, 250 mM NaCl, 5% glycerol, 5 mM β-mercaptoethanol). Cells were lysed and lysate was cleared as described, then the soluble fraction was mixed with Ni Sepharose 6 Fast Flow Affinity Chromatography Media (Cytiva) at 4°C for Cas7/Cas5 RNP complexes or Strep-Tactin Superflow Plus resin (Qiagen) at 4°C for Cas14. Each resin was washed on a gravity flow column as described above with the modification for Cas14 that imidazole was removed for all buffers. Bound RNP was then eluted in His elution buffer (50 mM Tris pH 8, 500 mM NaCl, 5% glycerol, 300 mM imidazole and 5 mM β-mercaptoethanol) or Strep elution buffer (50 mM Tris-HCl pH 8, 500 mM NaCl, 5% glycerol, 5 mM desthiobiotin and 5 mM β-mercaptoethanol) as appropriate and dialyzed overnight into dialysis buffer (20 mM Tris-HCl pH 8, 250 mM NaCl, 5% glycerol and 1 mM DTT).

Cas12 RNP complexes: To purify Cas12s from the various newly identified Type-V associated systems in complex with cognate ncRNAs, Cas12 genes were either *E. coli* or human codon optimized, synthesized by Twist Biosciences and cloned into a pET45b(+) backbone with a His14-TwinStrep-bdSUMO solubility/affinity tag by Gibson assembly. Cognate predicted CRISPR arrays under expression of synthetic promoters (T7 or pJ23119, as specified) or full loci were similarly cloned into a separate pCOLADuet-1 vector and co-expressed with the cognate Cascade proteins in BL21(DE3) strain (NEB). Cells were grown at 37°C in terrific broth (TB) medium supplemented with 100 µg/ml ampicillin and 50 µg/ml kanamycin until reaching an optical density (OD600) of 0.4-0.6, then shifted to 18°C and induced with 0.2 mM IPTG overnight. RNPs were then purified as described for Cas5-HNH/Cas8-HNH/DinG-HNH effector complexes above. Instead of dialyzing, RNPs were directly concentrated using Vivaspin 20 columns with a molecular weight cutoff of 50 kDa (Cytiva) and 300 µL was taken as input for small RNA-sequencing.

<u>Small RNA sequencing</u>

RNPs: Small RNA sequencing of RNPs was performed as previously described (*25*). Briefly, 300 µL of protein elute was mixed with 900 µL TRI reagent (Zymo) and incubated at room temperature for 5 min. 180 µL of chloroform (Sigma Aldrich) was added and the samples were mixed gently and incubated at room temperature for an additional 3 min, then spun at 12000xg for 15 min at 4°C. The aqueous phase was used as input for RNA extraction using a Direct-zol RNA miniprep plus kit (Zymo), with in-column DNAse treatment. The purified RNA was then

subject to treatment as per manufacturer's instructions with 20 U of T4 PNK (NEB) for 1 hour at 37°C, then 20 U of RNA 5′ polyphosphatase (Biosearch Technologies) for 30 min at 37°C, with each enzymatic step followed by cleanup with a Zymo RNA Clean & Concentrator-5 kit as per the manufacturer's instructions. Following enzymatic treatments, purified RNA was subject to library preparation with an NEBNext Multiplex Small RNA Library Prep kit (NEB) as per the manufacturer's instructions, with an extension time of 1 min and 12 cycles in the final PCR. Amplified libraries were gel extracted, quantified by qPCR using a KAPA Library Quantification Kit for Illumina (Roche) on a CFX Opus 384 (BioRad) and sequenced on an Illumina MiSeq with Read 1 45 cycles, Read 2 45 cycles and Index 1 6 cycles. Adapters were trimmed using CutAdapt (*79*) and mapped to loci of interest using BWA. Filled reads were obtained and filled reads filtered as indicated were visualized using a custom Python script (*25*).

Heterologous loci in E. coli: Small RNA sequencing of heterologous loci in *E. coli* was performed as previously described (*25*). Briefly, Stbl3 chemically competent *E. coli* were transformed with plasmids containing the locus of interest. A single colony was used to seed a 5 mL overnight culture. Following overnight growth, cultures were spun down, resuspended in 750 µL TRI reagent (Zymo) and incubated for 5 min at room temperature. 0.5 mm zirconia/silica beads (BioSpec Products) were added and the culture was vortexed for approximately 1 minute to mechanically lyse cells. 200 µL chloroform (Sigma Aldrich) was then added and the above protocol was followed with the following modifications: prior to T4 PNK treatment, ribosomal RNA was removed using an NEBNext rRNA depletion kit (Bacteria) (NEB).

Native expression: Organisms were obtained from NRRL or ATCC as freeze dried cultures, resuspended in the appropriate media and cultured as follows: *Saccharamoronospora internatus* (Agre et. al.) Greiner-Mai et. al. (ATCC 33517) was grown in ISP Medium 4 at 37°C for 4 days and *Amycolatopsis decaplanensis* (NRRL B-24209) and *Prauserella rugosa* (NRRL B-2295) were grown in GYM Streptomyces media at 28°C for 3 days. RNA was extracted and libraries were prepared as described for "Heterologous loci in *E. coli*" above.

In vitro cleavage assays

Cas5-HNH and Cas8-HNH cleavage assays: For dsDNA cleavage, substrates were produced by PCR amplification of pUC19 plasmids containing the target sites and the TAM sequences. Cy3 and Cy5-conjugated DNA oligonucleotides (IDT) were used as primers to generate the labeled dsDNA substrates. For ssDNA cleavage, substrates were ordered as Cy5-conjugated oligonucleotides (IDT). Target cleavage assays contained 8 nM of dsDNA substrate or 25 nM of ssDNA substrate, and RNP in a final 1x reaction buffer of 20 mM Tris pH 8, 50 mM NaCl and 5 mM $MgCl_2$. For the Cas8-HNH system, an RNP concentration of approximately 100 nM was used. For the Cas5-HNH system, RNP concentration could not be estimated due to super-stoichiometric levels of the tagged Cas11 protein in the sample. For collateral cleavage assays, conditions an additional 25 nM of FAM-labeled collateral (untargeted) ssDNA was added. Assays were allowed to proceed at 37°C for 1 hour. Reactions were then treated with 1.6 U of Proteinase K (NEB) at room temperature for 15 min, followed by treatment with 1 µL of 10 mg/mL RNAse A (Qiagen) for 5 min at room temperature. DNA was resolved by gel electrophoresis on 2% agarose E-gels (Thermo Fisher Scientific) or Novex 10% TBE-Urea gels (Thermo Fisher Scientific) as specified and imaged using a BioRad ChemiDoc imaging system.

DinG-HNH cleavage assays: DsDNA substrates were produced by PCR amplification of pUC19 plasmids containing the target sites and the TAM sequences. Cy3 and Cy5-conjugated DNA oligonucleotides (IDT) were used as primers to generate the labeled dsDNA substrates.

Target cleavage assays contained 8 nM of DNA substrate, and 1 μM of each specified protein or RNP in a final 1x reaction buffer of 20 mM Tris-HCl pH 8, 50 mM NaCl, 10 mM $MgCl_2$ and 1 mM ATP. Assays were allowed to proceed at 22°C or temperature as specified for 1 hour. Reactions were then treated with 1.6 U of Proteinase K (NEB) at room temperature for 15 min, followed by treatment with 1 μL of 10 mg/mL RNAse A (Qiagen) for 5 min at room temperature. DNA was resolved by gel electrophoresis on Novex 10% TBE-Urea polyacrylamide gels (Thermo Fisher Scientific) as specified and imaged using a BioRad ChemiDoc imaging system.

Candidate Type VII cleavage assays: Templates for *in vitro* transcription of target RNAs were produced by PCR amplification of pUC19 plasmids containing the target site. RNA was *in vitro* transcribed using the PCR templates with a HiScribe T7 Quick High Yield RNA Synthesis kit (NEB). 50 pmol of target RNA was heated for 3 min at 65°C, then labeled with 100 pmol of pCp-Cy5 (Jena Biosciences) using 68 U T4 RNA ligase 1, ssRNA ligase (High Concentration) (NEB) in a 1X buffer of 50 mM Tris-HCl pH 7.5, 10 mM $MgCl_2$, 2 mM DTT, 2 mM ATP and 10% DMSO at 4°C overnight and purified using a Zymo RNA Clean & Concentrator-5 column as per the manufacturer's instructions. Target cleavage assays contained 25 nM of labeled RNA substrate, ~1 μM of Cas7/Cas5 RNP and ~2 μM of Cas14 protein in a final 1x reaction buffer of 20 mM HEPES pH 8, 100 mM KCl, 40 mM NaCl, 1 mM $MgCl_2$, and 1 mM ATP. Collateral cleavage assays contained an additional 25 nM of either ssRNA labeled as described or ssDNA ordered as a Cy5-conjugated oligonucleotide (IDT) along with an unlabeled ssRNA containing the cognate target sequence. Assays were allowed to proceed at 45°C or temperature as specified for 1 hour. Reactions were then treated with 1.6 U of Proteinase K (NEB) at room temperature for 15 min. RNA was resolved by gel electrophoresis on Novex 10% TBE-Urea polyacrylamide gels (Thermo Fisher Scientific) as specified and imaged using a BioRad ChemiDoc imaging system.

E. coli transformation spot assays

Stbl3 competent cells were transformed with plasmids containing protein(s) of interest and corresponding crRNAs and selected on LB plates with 25μM chloramphenicol and 50μM kanamycin. Overnight cultures in TB were prepared then diluted 1:100 in selective ZymoBroth (Zymo Research) and cultured to an OD_{600} of 0.6-0.8 at 37° C at 220 rpm. Competent cells were prepared with a Mix & Go E. Coli transformation kit (Zymo Research) according to developer instructions. 20μL of competent cells were transformed by heat shock with 100 ng pUC19 or plasmids containing an a crRNA target and appropriate PAM and recovered in 80uL of SOC media (Thermo Fisher Scientific) and for 2 hours at 37° C at 220 rpm. Transformed cells were normalized to OD_{600} = 0.1 then five 1:10 serial dilutions prepared in TB. 5μL of each dilution were induced on LB plates with 25μM chloramphenicol, 50μM kanamycin, 100μM ampicillin and 0.1mM IPTG then incubated 24 hours at 37° C. Plates were imaged with a BioRad ChemiDoc imaging system.

Sanger sequencing of cleavage sites

In vitro cleavage assays were performed as described above and products were run on 2% agarose E-gels (Invitrogen) and cleaved products were extracted using a QIAquick gel extraction kit (Qiagen). Gel extracted cleavage products were submitted for Sanger sequencing by Genewiz/Azenta Life Sciences using primers 5′-

AGTCACGACGTTGTAAAACGACGGCCAGTG-3′ for the non-target strand and 5′-TCATTAGGCACCCCAGGCTTTAC-3′ for the target strand.

Mammalian cell culture and transfection

Mammalian cell culture experiments were performed in the HEK293FT line (Thermo Fisher Scientific) grown in Dulbecco's Modified Eagle Medium with high glucose, sodium pyruvate, and GlutaMAX (Thermo Fisher Scientific), additionally supplemented with 1× penicillin–streptomycin (Thermo Fisher Scientific), 10 mM HEPES (Thermo Fisher Scientific), and 10% fetal bovine serum (VWR Seradigm). All cells were maintained at confluency below 80%.

Unless otherwise indicated, all transfections were performed with Lipofectamine 3000 (Thermo Fisher Scientific). Cells were plated 16-20 hours prior to transfection to ensure 90% confluency at the time of transfection. For 96-well plates, cells were plated at 20,000 cells/well. For each well on the plate, transfection plasmids were combined with 2 µL P3000 transfection enhancer (Thermo Fisher Scientific) per 1 µg DNA and OptiMEM I Reduced Serum Medium (Thermo Fisher Scientific) to a total of 25 µL. Separately, 23 µL of OptiMEM was combined with 2 µL of Lipofectamine 2000. Plasmid/P3000 and Lipofectamine solutions were then combined and pipetted onto cells.

For off-target genome editing, cells were plated in 12-well plates at 200,000 cells/well 16-20 hours prior to transfection to ensure 90% confluency at the time of transfection. To transfect, 200 ng of each protein expression plasmid and 500 ng of each crRNA expression plasmid per well were combined with OptiMEM I Reduced Serum Medium (Thermo Fisher Scientific) up to 250 µL. Separately, 5 µL of GeneJuice transfection reagent (EMD Millipore) was combined with 245 µL OptiMEM I Reduced Serum Medium (Thermo Fisher Scientific). DNA and GeneJuice mixes were combined to a final volume of 500 µL and pipetted onto cells.

Mammalian genome editing

crRNA scaffold backbones were cloned into a pUC19-based human U6 expression backbone by Gibson Assembly. To clone guides, oligos with appropriate overhangs were synthesized by Genewiz, annealed and phosphorylated using T4 PNK (NEB) and cloned into crRNA backbones by restriction-ligation cloning. To clone protein expression constructs, *E. coli* codon optimized genes were cloned into a CMV expression backbone by Gibson assembly using 2X Gibson Assembly Master Mix (NEB) to generate pCMV-FLAG-SV40-Cas protein constructs.

50 ng each protein expression plasmid and 100 ng crRNA expression plasmid were transfected in each of 4 wells as biological replicates in a 96- well plate for each guide condition as described. An empty protein expression plasmid was used for dropout experiments as indicated. After 60-72 hours, genomic DNA was harvested by washing the cells once in 1xDPBS (Sigma Aldrich) and adding 50 µL QuickExtract DNA Extraction Solution (Lucigen). Cells were scraped from the plates to suspend in QuickExtract and cycled at 65°C for 15 min, 68°C for 15 min then 95°C for 10 min to lyse cells. 2.5 µL of lysed cells were used as input into each PCR reaction. For library amplification, target genomic regions were amplified and with a 12-cycle PCR using NEBNext High Fidelity 2X PCR Master Mix (NEB) with an annealing temperature of 63°C for 15 s, followed by a second 18-cycle round of PCR to add Illumina adapters and barcodes. The libraries were gel extracted and subject to single-end sequencing on an Illumina MiSeq with Read 1 300 cycles, Index 1 8 cycles, Index 2 8 cycles.

Insertion/deletion (indel) frequency was analyzed using CRISPResso2 (*80*). In order to eliminate noise from PCR and sequencing error, only indels with at least 2 reads or more than 1

base inserted or deleted were counted towards reported indel frequencies. To assess statistical significance, 2-tailed T-tests were performed using non- targeting guide/crRNA conditions as a negative control.

Off-target genome editing analysis

Off-target genome editing was analyzed by tagmentation-based tag integration site sequencing (TTISS) (*41*). Briefly, donor oligos (5′-phospho-G*T*TGTGAGCAAGGGCGAGGAGGATAACGCCTCTCTCCCAGCGACT*A*T-3′ and 5′-phospho-A*T*AGTCGCTGGGAGAGAGGCGTTATCCTCCTCGCCCTTGCTCACA*A*C-3′, where * indicates a phosphorothioate modification) were annealed duplex buffer (30 mM HEPES pH 7.5, 100 mM KAc) at a final concentration of 10 µM per oligo by incubating in a thermocycler at 95°C for 5 s, then ramping down to 4°C at ~0.1°C/s. Cells were transfected as described in "Mammalian cell culture and transfection" above. 72-96 hours after transfection, cells were harvested by washing each well with 1 mL PBS (Sigma Aldrich), then dry trypsinizing with 200 µL of TrypLE Express Enzyme (no phenol red) (Thermo Fisher Scientific) and resuspended in 1 mL PBS (Sigma Aldrich). Cell samples were then centrifuged at 300xg for 5 min at 4°C to pellet and resuspended in 200 µL PBS (Sigma Aldrich). A DNeasy Blood & Tissue kit (Qiagen) was used to extract genomic DNA as per the manufacturer's instructions.

Genomic DNA was then tagmented by mixing 1µg DNA with Tn5 enzyme and TAPS buffer in a final volume of 100 µL and heating to 55°C for 10 min. The tagmentation reactions were mixed with 500 µL of PB Binding buffer (Qiagen) and purified using a Qiaquick spin column (Qiagen). 10 µL of purified tagmented DNA was input into 2 rounds of PCR to add Illumina handles using 2X KOD Hot Start PCR master mix (EMD Millipore). The final libraries were gel extracted, quantified with a KAPA Library Quantification Kit (Roche), mixed with 10% PhiX Sequencing Control V3 (Illumina) and loaded on an Illumina NextSeq with the following read parameters: Read 1 59 cycles, Index 1 8 cycles, Read 2 25 cycles.

Off-target sites were identified by mapping reads to the hg38 assembly of the human genome using BrowserGenome.org as described (*41*). Identified sites were then filtered for those containing a continuous sequence with up to ⅓ of bases mismatched to the guide sequence and up to 1 mismatch in the PAM sequence to identify "true" off-target cleavage events.

Size exclusion chromatography

Candidate Type VII Cas7/Cas5 complexes were purified as described, with the following modifications: buffer A contained 500 mM NaCl, buffer B contained 250 mM NaCl, and elution buffer contained 250 mM NaCl. The elution was directly diluted into to achieve a final concentration of 20 mM Tris-HCl pH 8, 150 mM NaCl, 100 mM imidazole and 3.6 % glycerol. The sample was concentrated to 500 µL using a Vivaspin 20 column with a molecular weight cutoff of 50 kDa (Cytiva) and purified through a Superose 6 Increase 10/300 GL column (Cytiva) in 20 mM Tris-HCl pH 8, 150 mM NaCl, 1% glycerol. Fractions were collected and analyzed by SDS-PAGE gel electrophoresis.

Supplementary text. Extended discussion of FLSHclust algorithm

Hash-based clustering algorithms divide objects into a set of elements that can be matched individually using hash functions. With proteins, a hash function bucketing scheme allows grouping proteins that share kmers. However, the efficiency of kmer-based grouping for linear time clustering decreases as sequence identity between proteins drops because kmer bucketing requires long strings of identical consecutive matches. Thus, the technique becomes less effective in grouping proteins that share low sequence similarity.

Locality sensitive hashing (LSH) is a well-established technique for approximate nearest neighbor matching (*81*). By expanding a single hash function into a family of hash functions, LSH allows for inexact matching of elements at the cost of false negatives, false positives, and extra computational time (Fig. 1D). While LSH eliminates the need for long consecutive identical sequence matches, the high number of false negatives can adversely affect clustering performance by causing missed matches. However, a recent theorem for binary LSH functions (functions acting on 01 bit vectors) demonstrates the possibility of implementing these functions in a way that guarantees the absence of binary LSH families that have no false negatives (*14*). We applied this theorem to the case of string kmers to generate false negative-free kmer LSH families and subsequently developed the FLSHclust algorithm. Using Markov-Chain Monte Carlo, we generated kmer LSH functions with no false negatives. To do so for a specific kmer length, k, we enumerated all possible combinations of mismatch positions up to r and computed a loss equal to the number of mismatch combinations that go undetected using the entire set of hash functions. The hash functions are then perturbed using MCMC until the loss converges.

The FLSH clustering algorithm is then constructed as follows: first, all amino acids are compressed to a reduced alphabet that groups similar amino acids together. Next, all kmers of size k are extracted from each protein. Then, for each hash function, all kmers are mapped to buckets using the output of the hash function applied to the kmers. 2 representative sequences are deterministically selected per bucket to be temporary cluster centers. All sequences in the bucket are then retrieved and aligned against the cluster centers. A graph edge is formed if the alignment between the two sequences satisfies the clustering sequence identity and minimum coverage criteria. The resulting graph of edges is then simplified using a local density-based transformation that removes weak links that would otherwise create long chains of unrelated sequences in the graph. Next, a community detection algorithm is applied to form groups of sequences. For simplicity, a PREGEL implementation of the breadth first connected components algorithm is used; however notably other algorithms could be used in this place instead, such as the Leiden algorithm (*82*). The most hub-like sequence from each group is selected as the initial cluster center and all sequences in the group are realigned to the initial cluster center forming new edges. Each group of sequences is then clustered using greedy clustering using the new subgraph. To increase parallelizability, the FLSHclust algorithm replaces the global greedy clustering stage included in LinClust (*11*) with a parallelized graph simplification and community detection algorithm followed by local greedy clustering within each detected community (Fig. 1E). The algorithm is parallelizable and is bottlenecked by the maximum size of the detected communities. We implemented FLSHclust in Apache Spark, employing Apache Arrow for in-memory access to enhance fault tolerance and scalability on distributed commodity clusters. Other implementations aimed at speed as opposed to fault tolerance may yield performance improvements in other computing environments.

Fig. S1.

A

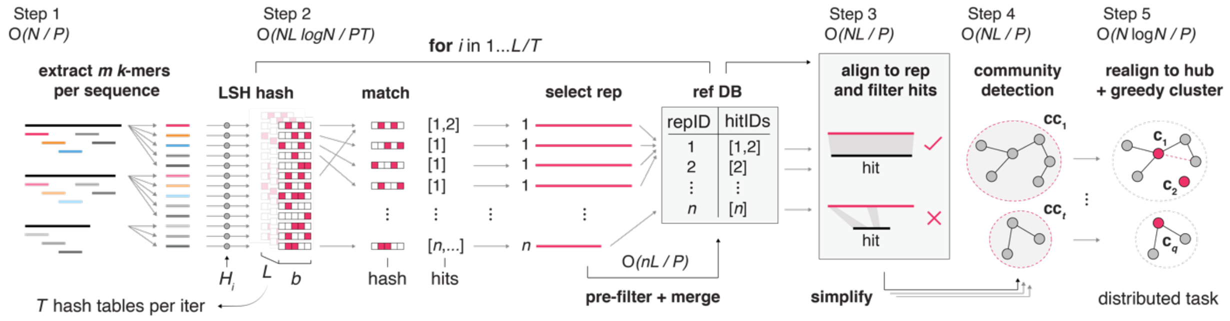

B

Algorithm 1: FLSHclust algorithm

Input: sequences $s \in S$, len$(S) = n$, LSH functions $\{H_i\}_{i=0,\ldots,L-1}$
Parameter: min_cov $\in [0,1]$ // Minimum alignment coverage
Parameter: min_seq_id $\in [0.3,1]$ // Minimum sequence identity
Output: partitioning $\{C_i\}$ with $C_i \subset S$, $C_i \cap C_j = \emptyset$, $\bigcup_i C_i = S$
Data: S all known proteins

$X \leftarrow \emptyset$
for $s \in S$ **do**
  for *length k kmer* $x_k \in s$ **do**
    append x_k to X
$P \leftarrow \emptyset$
for $i \in \{0, \ldots, L-1\}$ **do**
  apply hash function to all kmers $x_k \in X$: $x_k \rightarrow H_i(x_k)$
  for *key, list* $\in$ *groupby* $(H_i(X))$ **do**
    select representative $s_{\text{rep}} \leftarrow R(list)$
    filter $list \leftarrow F(list, s_{\text{rep}})$
    for $s \in list$ **do**
      append (s, s_{rep}) to P
$\hat{E} \leftarrow \emptyset$
for $(s, s_{\text{rep}}) \in P$ **do**
  a $\leftarrow$align(s, s_{rep})
  if cov $(a) \geq$ min_cov *and* seq_id $(a) \geq$ min_seq_id **then**
    append (s, s_{rep}) to $\hat{E}$
$E \leftarrow$ simplify$(\hat{E})$ // density based edge removal
$\hat{C} \leftarrow$ community_detection$(\hat{E})$ // e.g. Connected Components
$C \leftarrow \emptyset$
for $c \in \hat{C}$ **do**
  select representative $s_{\text{rep}} \in c$
  $A_c \leftarrow \emptyset$
  for $s \in c$ **do**
    append align(s, s_{rep}) to A
  for $\hat{c} \in$ greedy_cluster(c, A, s_{rep}) **do**
    append $\hat{c}$ to C
return C

Fig. S1. Complete FLSHclust algorithm

(A) Complete outline of the FLSHclust algorithm. Unlike with typical LSH, which often requires materializing the entire set of L hash tables, FLSHclust only needs to materialize one hash table at a time, storing all potential matches in a reference database. As memory and disk space

permits, up to *T* hash tables can be materialized per iteration, potentially reducing runtime. Time complexities shown (big O notation) are assuming the use of hash join implementations, however if merge join implementations are used, additional logarithmic factors are included in Step 2. *N* is the number of sequences. *L* is the number of hash functions. *P* is the parallel speedup that can be obtained using the given number of processors. (**B)** Pseudocode of the FLSHclust algorithm.

Fig. S2.

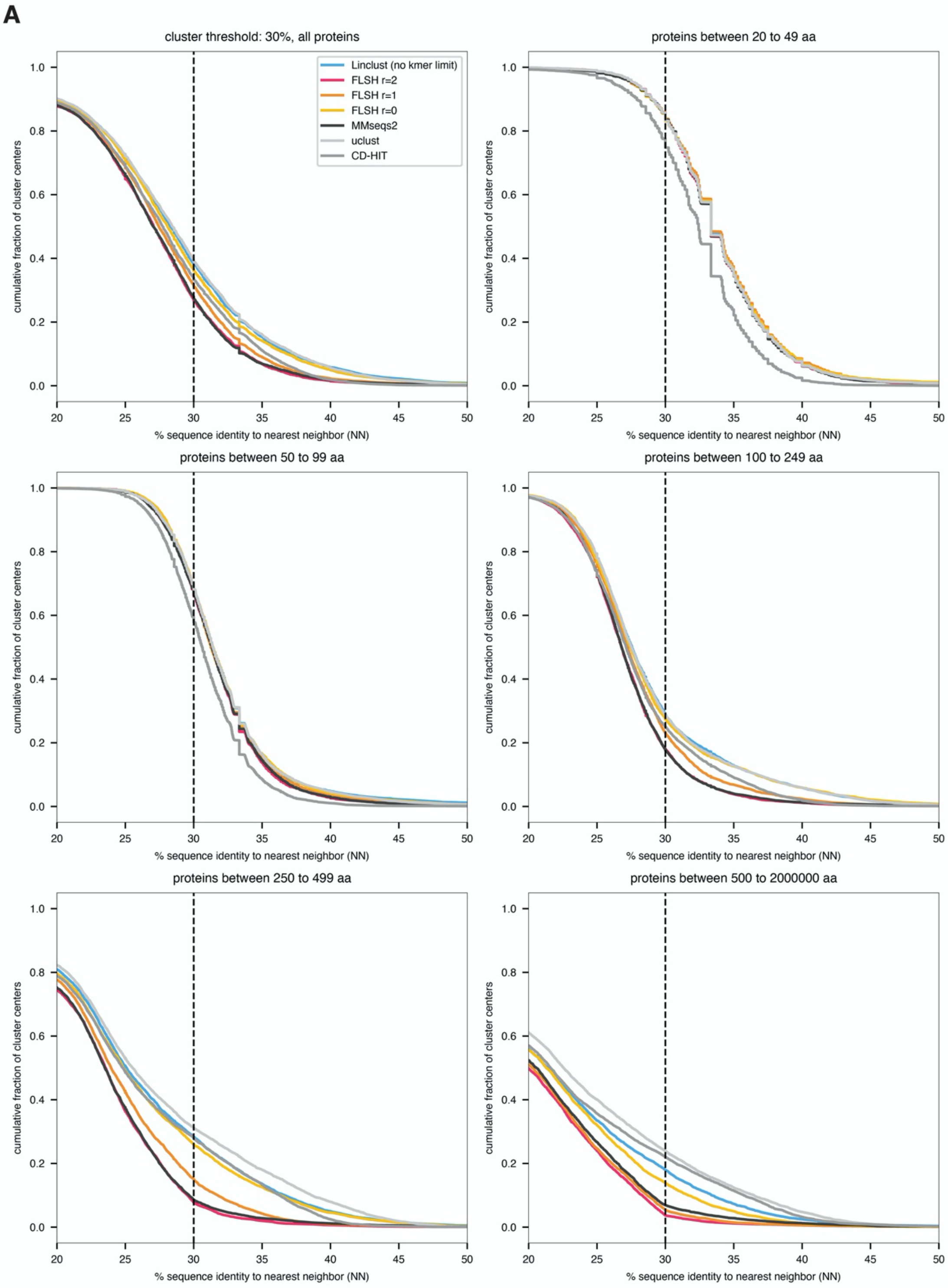
A
cluster threshold: 30%, all proteins
Linclust (no kmer limit)
FLSH r=2
FLSH r=1
FLSH r=0
MMseqs2
uclust
CD-HIT
proteins between 20 to 49 aa
proteins between 50 to 99 aa
proteins between 100 to 249 aa
proteins between 250 to 499 aa
proteins between 500 to 2000000 aa
cumulative fraction of cluster centers
% sequence identity to nearest neighbor (NN)

Fig. S2. (cont'd)

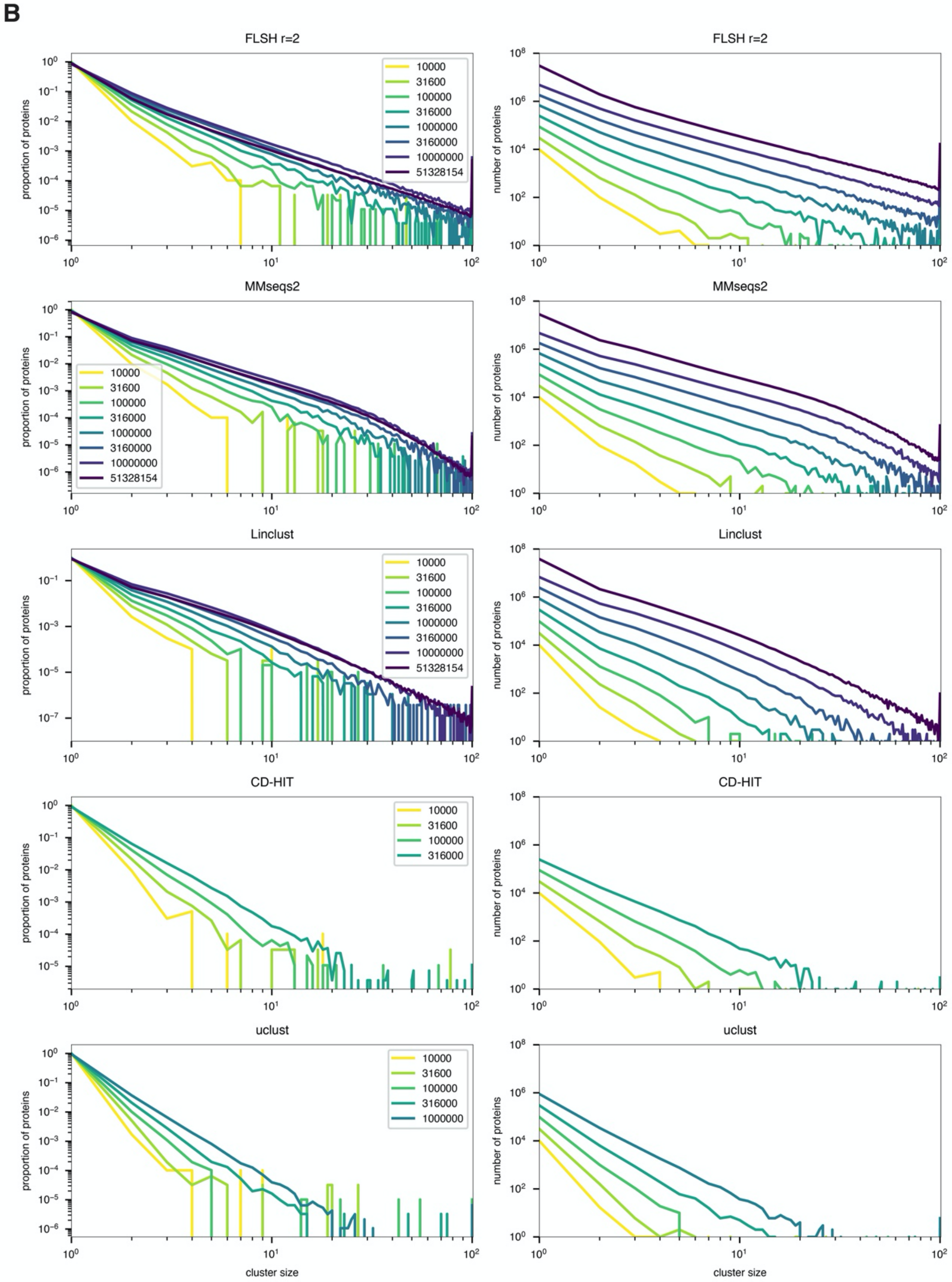
B
FLSH r=2
MMseqs2
Linclust
CD-HIT
uclust
proportion of proteins
number of proteins
cluster size
10000
31600
100000
316000
1000000
3160000
10000000
51328154

Fig. S2. (cont'd)

C

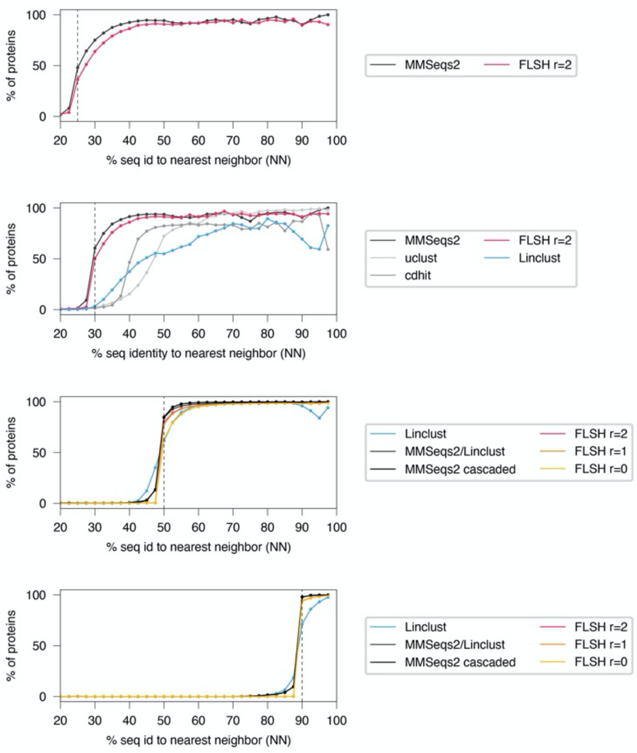

Fig. S2. (cont'd)

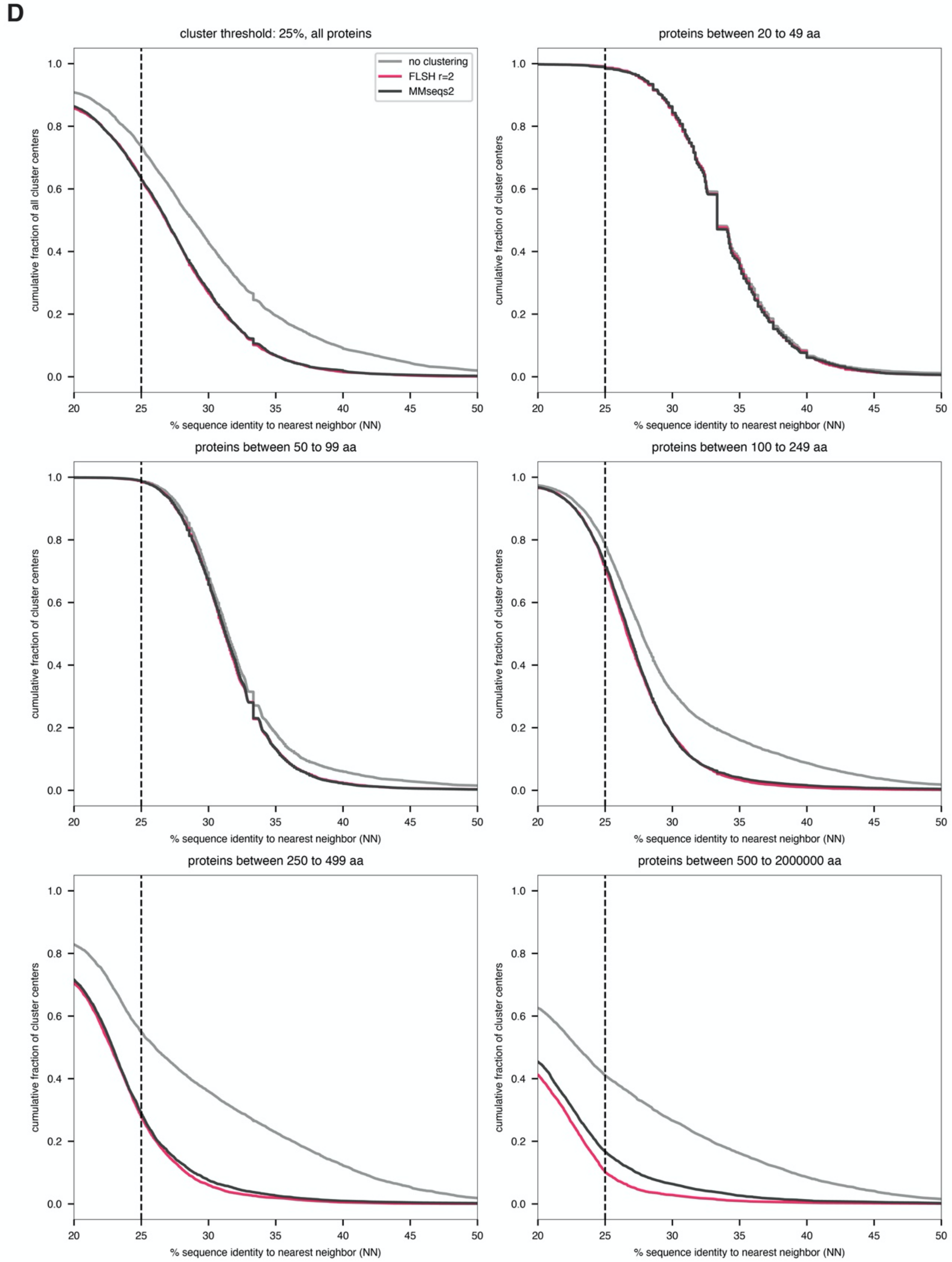
D
cluster threshold: 25%, all proteins
no clustering
FLSH r=2
MMseqs2
cumulative fraction of all cluster centers
% sequence identity to nearest neighbor (NN)
proteins between 20 to 49 aa
proteins between 50 to 99 aa
proteins between 100 to 249 aa
proteins between 250 to 499 aa
proteins between 500 to 2000000 aa
cumulative fraction of cluster centers

Fig. S2. (cont'd)

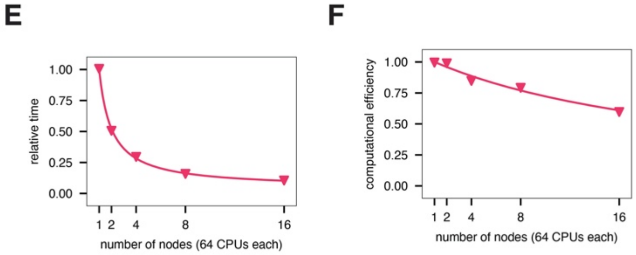

Fig. S2. Empirical scaling and benchmarking of FLSHclust vs other clustering algorithms

(A) Comparison of inter cluster distances for clustering the UniRef50 1M sample at 30% sequence identity. For each method, a random sample of 5000 cluster representatives were selected and aligned to all other cluster representatives from that clustering outcome. Then, the cumulative distribution of the nearest (non-self) neighbor distances were plotted. Separately, the analysis was performed for different subsets of cluster centers satisfying a given protein size range. **(B)** Distribution of cluster sizes for various clustering methods as a function of the number of input sequences from UiRef50. **(C)**. Clustering performance for different clustering tasks (from top to bottom: UniRef50 1M sample at 25% sequence identity, UniRef50 1M sample at 30% sequence identity, UniRef90 1M sample at 50% sequence identity, UniRef100 1M sample at 90% sequence identity). Dotted vertical black line indicates the target clustering threshold. For FLSHclust, *r*, references the target number of mismatches in the FLSH kmer comparison step, with a larger *r* corresponding to exponentially more hash functions (*r*=0 uses 1 hash function, *r*=1 uses 6 hash functions, *r*=2 uses 24 hash functions). **(D)** same as **(A)**, but for the target clustering threshold of 25% sequence identity. **(E)**. Relative time required for FLSHclust to complete clustering the 3.62as a function of number of compute nodes (each with 64 CPUs, 512 GB of memory and 2TB of RAM). **(F)** Parallel computation efficiency for FLSHclust as a function of the number of compute nodes.

Fig. S3.

A

	dr_len	spacer_len	p_mismatch	indel_rel_range	p_indel	p_spacer_similarity	spacer_indel_rel_range	p_spacer_indel	condition_group
**0**	37	30	0.00	0.0	0.0	0.3	0.3	1.0	0
**1**	37	30	0.05	0.0	0.0	0.3	0.3	1.0	1
**2**	37	30	0.10	0.0	0.0	0.3	0.3	1.0	2
**3**	37	30	0.20	0.0	0.0	0.3	0.3	1.0	3
**4**	37	30	0.00	0.1	0.2	0.3	0.3	1.0	4
**5**	37	30	0.00	0.1	0.4	0.3	0.3	1.0	5
**6**	37	30	0.00	0.1	0.6	0.3	0.3	1.0	6
**7**	37	30	0.00	0.1	0.8	0.3	0.3	1.0	7
**8**	37	30	0.10	0.1	0.2	0.3	0.3	1.0	8
**9**	37	80	0.10	0.1	0.2	0.3	0.3	1.0	9
**10**	20	30	0.10	0.1	0.2	0.3	0.3	1.0	10
**11**	72	30	0.10	0.1	0.2	0.3	0.3	1.0	11
**12**	128	30	0.10	0.1	0.2	0.3	0.3	1.0	12
**13**	256	30	0.10	0.1	0.2	0.3	0.3	1.0	13
**14**	400	60	0.20	0.1	0.2	0.3	0.3	1.0	14
**15**	20	30	0.00	0.0	0.0	0.3	0.3	1.0	15
**16**	30	30	0.05	0.0	0.0	0.3	0.3	1.0	16
**17**	24	30	0.05	0.0	0.0	0.3	0.3	1.0	17
**18**	42	30	0.05	0.0	0.0	0.3	0.3	1.0	18
**19**	10	5	0.00	0.0	0.0	0.3	0.3	1.0	19
**20**	20	5	0.00	0.0	0.0	0.3	0.3	1.0	20
**21**	30	5	0.00	0.0	0.0	0.3	0.3	1.0	21
**22**	40	5	0.00	0.0	0.0	0.3	0.3	1.0	22
**23**	50	5	0.00	0.0	0.0	0.3	0.3	1.0	23
**24**	60	5	0.00	0.0	0.0	0.3	0.3	1.0	24
**25**	70	5	0.00	0.0	0.0	0.3	0.3	1.0	25
**26**	80	5	0.00	0.0	0.0	0.3	0.3	1.0	26
**27**	10	10	0.00	0.0	0.0	0.3	0.3	1.0	27
**28**	20	10	0.00	0.0	0.0	0.3	0.3	1.0	28
**29**	30	10	0.00	0.0	0.0	0.3	0.3	1.0	29
**30**	40	10	0.00	0.0	0.0	0.3	0.3	1.0	30
**31**	50	10	0.00	0.0	0.0	0.3	0.3	1.0	31
**32**	60	10	0.00	0.0	0.0	0.3	0.3	1.0	32
**33**	70	10	0.00	0.0	0.0	0.3	0.3	1.0	33
**34**	80	10	0.00	0.0	0.0	0.3	0.3	1.0	34

B

Program	Condition	Parameters
CRT	CRT_1	minNR=3, minRL=16, maxRL=38, minSL=16, maxSL=48
CRT	CRT_2	minNR=3, minRL=16, maxRL=128, minSL=16, maxSL=128
CRT	CRT_3	minNR=3, minRL=16, maxRL=256, minSL=16, maxSL=128
CRT	CRT_4	minNR=3, minRL=16, maxRL=512, minSL=16, maxSL=128
PILER-CR	PILER_1	minarray=3, mincons=0.9, minid=0.94, maxrepeat=64, maxspacer=64
PILER-CR	PILER_2	minarray=3, mincons=0.9, minid=0.94, maxrepeat=128, maxspacer=128
PILER-CR	PILER_3	minarray=3, mincons=0.9, minid=0.94, maxrepeat=256, maxspacer=256
PILER-CR	PILER_4	minarray=3, mincons=0.8, minid=0.85, maxrepeat=512, maxspacer=128
PILER-CR	PILER_5	minarray=3, mincons=0.8, minid=0.85, maxrepeat=128, maxspacer=128
CRISPRDetect	CRISPRDetect	ALL DEFAULT PARAMETERS
CRISPSRFinder	CRISPRFinder	ALL DEFAULT PARAMETERS
CRONUS	CRONUS_1	seed_size=11, min_len=16, minid=90, max_repeat_size=256
CRONUS	CRONUS_2	seed_size=5, min_len=16, minid=80, max_repeat_size=128
CRONUS	CRONUS_3	seed_size=11, min_len=16, minid=80, max_repeat_size=256
CRONUS	CRONUS_4	seed_size=11, min_len=16, minid=80, max_repeat_size=512

C

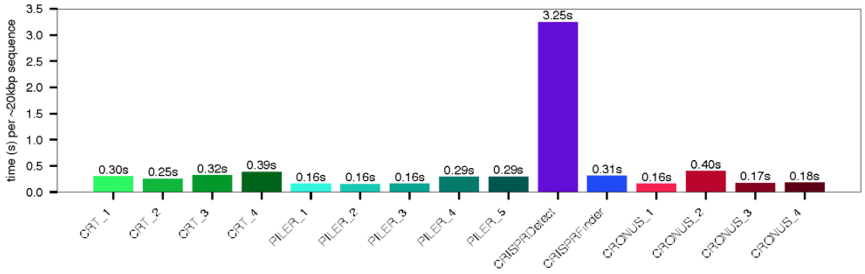

Fig. S3 (cont'd)

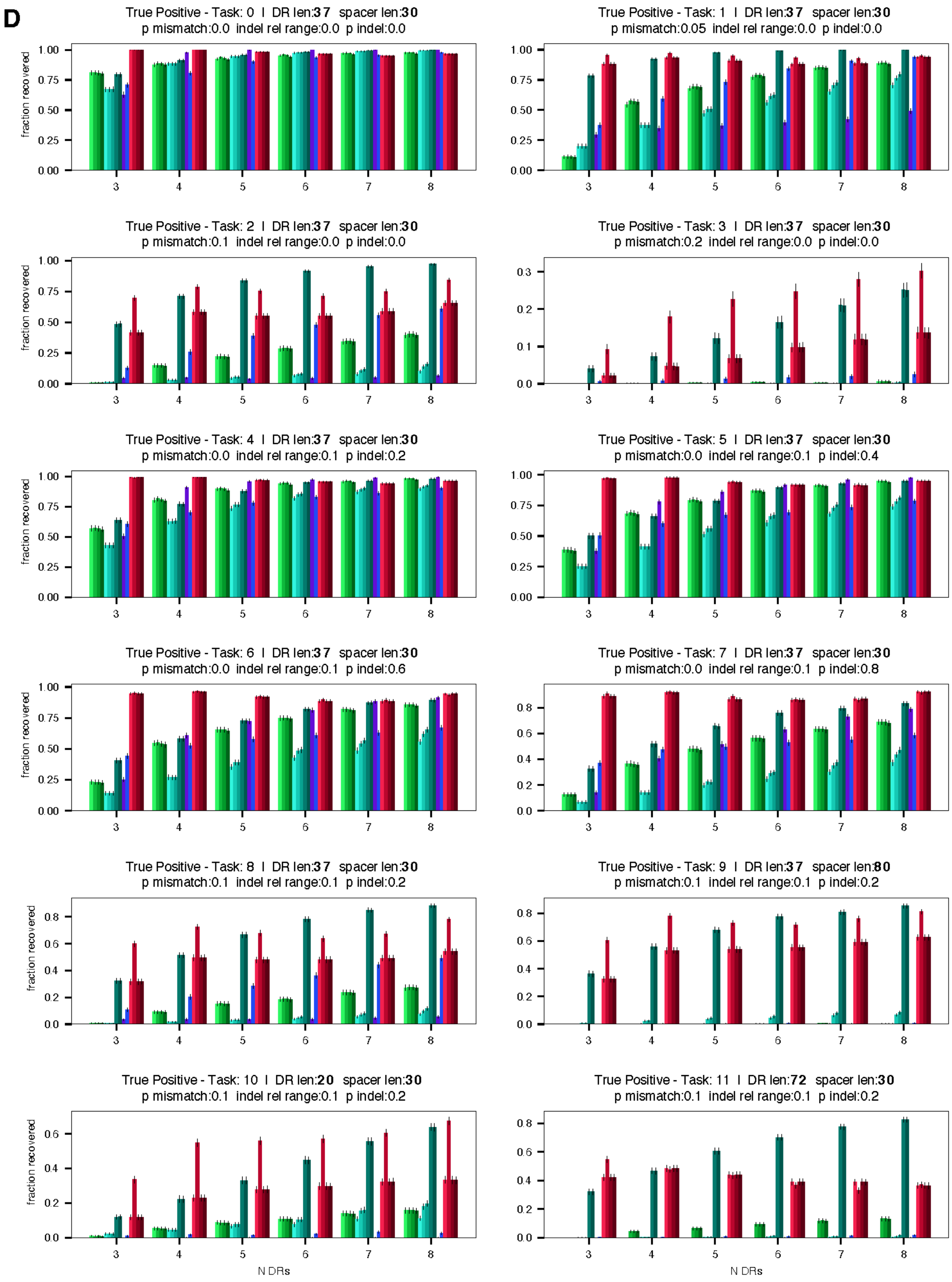
D
True Positive - Task: 0 l DR len:37 spacer len:30
p mismatch:0.0 indel rel range:0.0 p indel:0.0
True Positive - Task: 1 l DR len:37 spacer len:30
p mismatch:0.05 indel rel range:0.0 p indel:0.0
True Positive - Task: 2 l DR len:37 spacer len:30
p mismatch:0.1 indel rel range:0.0 p indel:0.0
True Positive - Task: 3 l DR len:37 spacer len:30
p mismatch:0.2 indel rel range:0.0 p indel:0.0
True Positive - Task: 4 l DR len:37 spacer len:30
p mismatch:0.0 indel rel range:0.1 p indel:0.2
True Positive - Task: 5 l DR len:37 spacer len:30
p mismatch:0.0 indel rel range:0.1 p indel:0.4
True Positive - Task: 6 l DR len:37 spacer len:30
p mismatch:0.0 indel rel range:0.1 p indel:0.6
True Positive - Task: 7 l DR len:37 spacer len:30
p mismatch:0.0 indel rel range:0.1 p indel:0.8
True Positive - Task: 8 l DR len:37 spacer len:30
p mismatch:0.1 indel rel range:0.1 p indel:0.2
True Positive - Task: 9 l DR len:37 spacer len:80
p mismatch:0.1 indel rel range:0.1 p indel:0.2
True Positive - Task: 10 l DR len:20 spacer len:30
p mismatch:0.1 indel rel range:0.1 p indel:0.2
True Positive - Task: 11 l DR len:72 spacer len:30
p mismatch:0.1 indel rel range:0.1 p indel:0.2
fraction recovered
N DRs

Fig. S3 (cont'd)

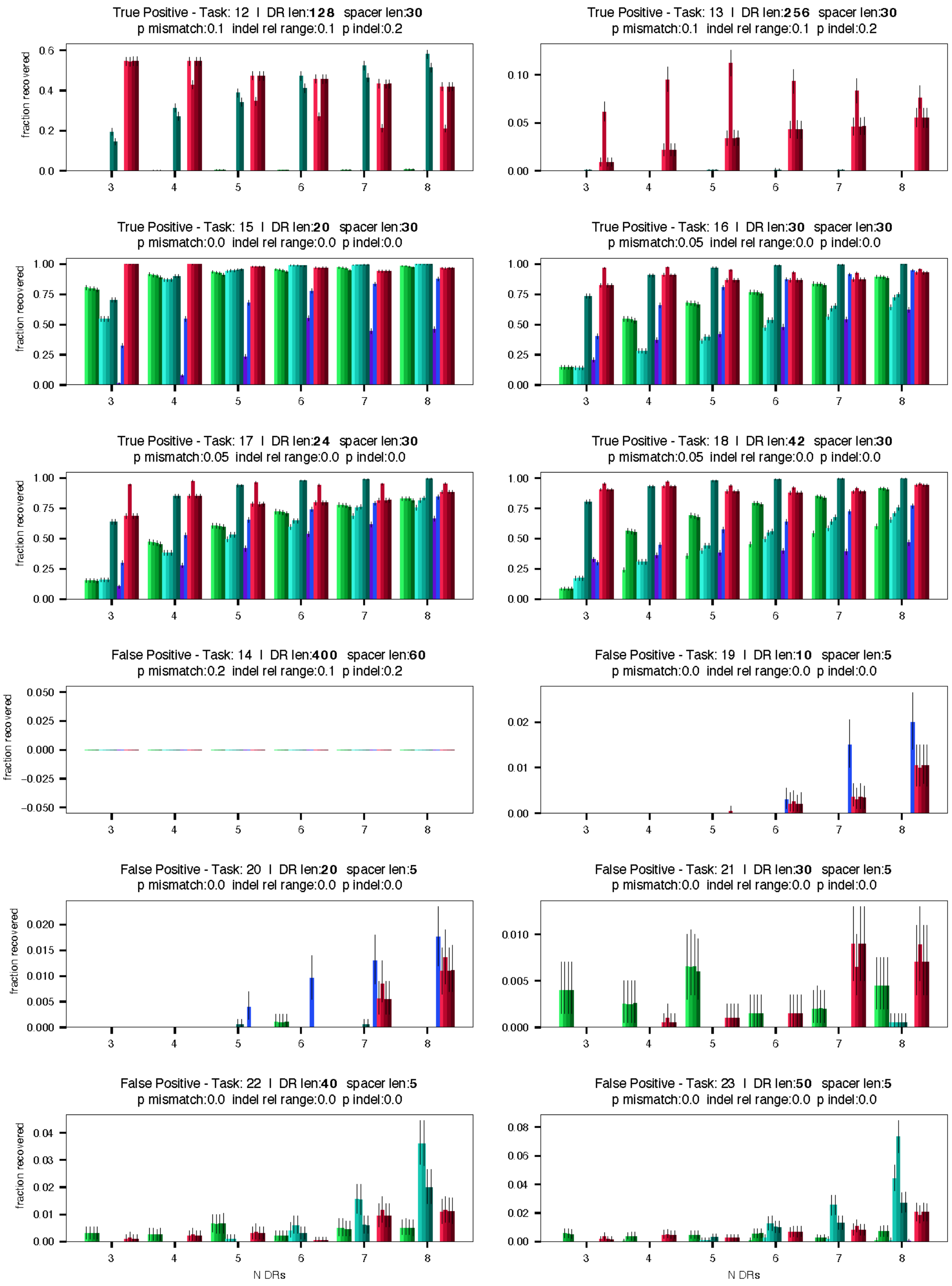
True Positive - Task: 12 I DR len:128 spacer len:30
p mismatch:0.1 indel rel range:0.1 p indel:0.2
True Positive - Task: 13 I DR len:256 spacer len:30
p mismatch:0.1 indel rel range:0.1 p indel:0.2
True Positive - Task: 15 I DR len:20 spacer len:30
p mismatch:0.0 indel rel range:0.0 p indel:0.0
True Positive - Task: 16 I DR len:30 spacer len:30
p mismatch:0.05 indel rel range:0.0 p indel:0.0
True Positive - Task: 17 I DR len:24 spacer len:30
p mismatch:0.05 indel rel range:0.0 p indel:0.0
True Positive - Task: 18 I DR len:42 spacer len:30
p mismatch:0.05 indel rel range:0.0 p indel:0.0
False Positive - Task: 14 I DR len:400 spacer len:60
p mismatch:0.2 indel rel range:0.1 p indel:0.2
False Positive - Task: 19 I DR len:10 spacer len:5
p mismatch:0.0 indel rel range:0.0 p indel:0.0
False Positive - Task: 20 I DR len:20 spacer len:5
p mismatch:0.0 indel rel range:0.0 p indel:0.0
False Positive - Task: 21 I DR len:30 spacer len:5
p mismatch:0.0 indel rel range:0.0 p indel:0.0
False Positive - Task: 22 I DR len:40 spacer len:5
p mismatch:0.0 indel rel range:0.0 p indel:0.0
False Positive - Task: 23 I DR len:50 spacer len:5
p mismatch:0.0 indel rel range:0.0 p indel:0.0
fraction recovered
N DRs

Fig. S3 (cont'd)

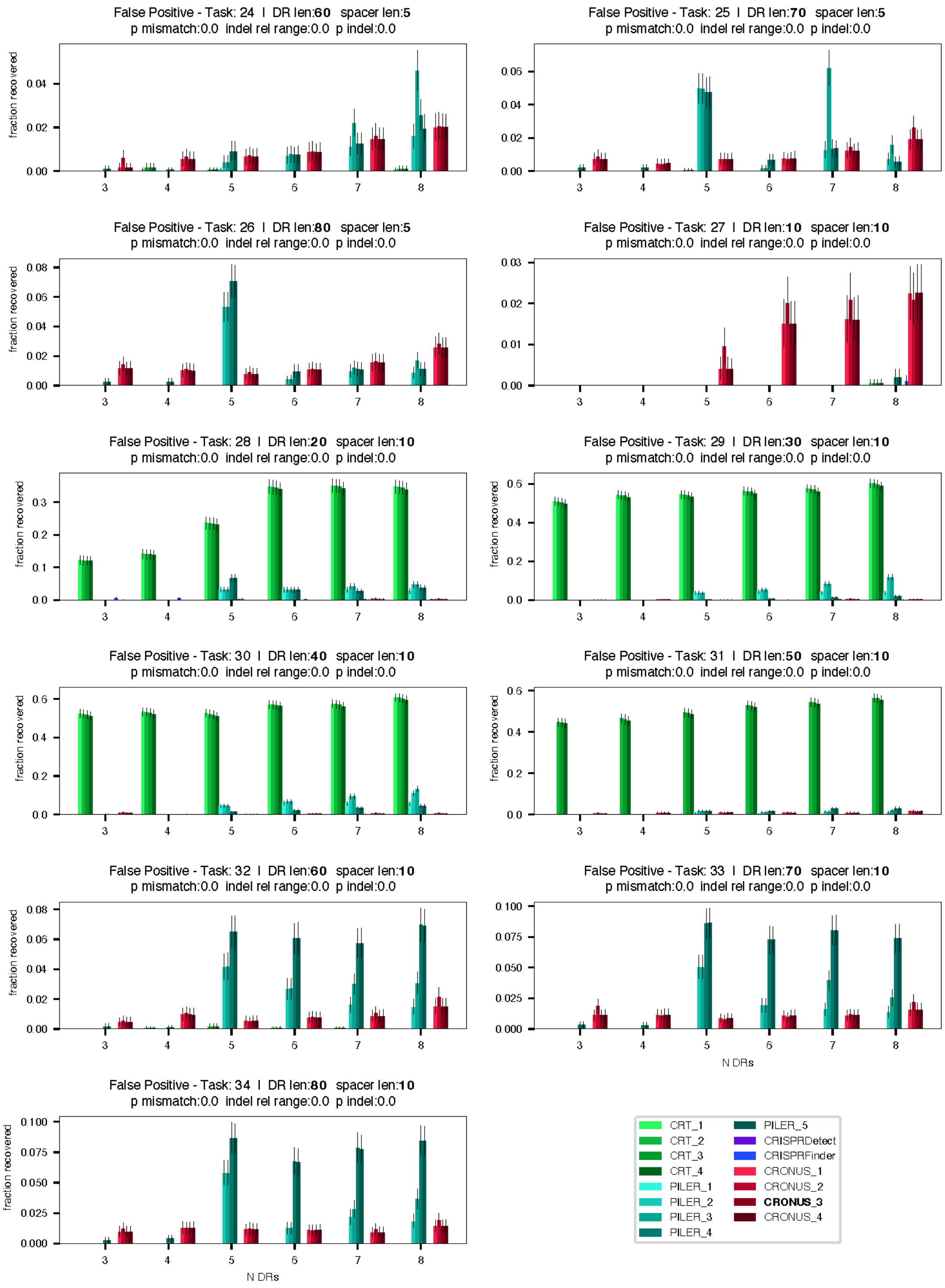
False Positive - Task: 24 | DR len:60 spacer len:5
p mismatch:0.0 indel rel range:0.0 p indel:0.0
False Positive - Task: 25 | DR len:70 spacer len:5
p mismatch:0.0 indel rel range:0.0 p indel:0.0
False Positive - Task: 26 | DR len:80 spacer len:5
p mismatch:0.0 indel rel range:0.0 p indel:0.0
False Positive - Task: 27 | DR len:10 spacer len:10
p mismatch:0.0 indel rel range:0.0 p indel:0.0
False Positive - Task: 28 | DR len:20 spacer len:10
p mismatch:0.0 indel rel range:0.0 p indel:0.0
False Positive - Task: 29 | DR len:30 spacer len:10
p mismatch:0.0 indel rel range:0.0 p indel:0.0
False Positive - Task: 30 | DR len:40 spacer len:10
p mismatch:0.0 indel rel range:0.0 p indel:0.0
False Positive - Task: 31 | DR len:50 spacer len:10
p mismatch:0.0 indel rel range:0.0 p indel:0.0
False Positive - Task: 32 | DR len:60 spacer len:10
p mismatch:0.0 indel rel range:0.0 p indel:0.0
False Positive - Task: 33 | DR len:70 spacer len:10
p mismatch:0.0 indel rel range:0.0 p indel:0.0
False Positive - Task: 34 | DR len:80 spacer len:10
p mismatch:0.0 indel rel range:0.0 p indel:0.0
fraction recovered
N DRs
CRT_1
CRT_2
CRT_3
CRT_4
PILER_1
PILER_2
PILER_3
PILER_4
PILER_5
CRISPRDetect
CRISPRFinder
CRONUS_1
CRONUS_2
CRONUS_3
CRONUS_4

Fig. S3 (cont'd)

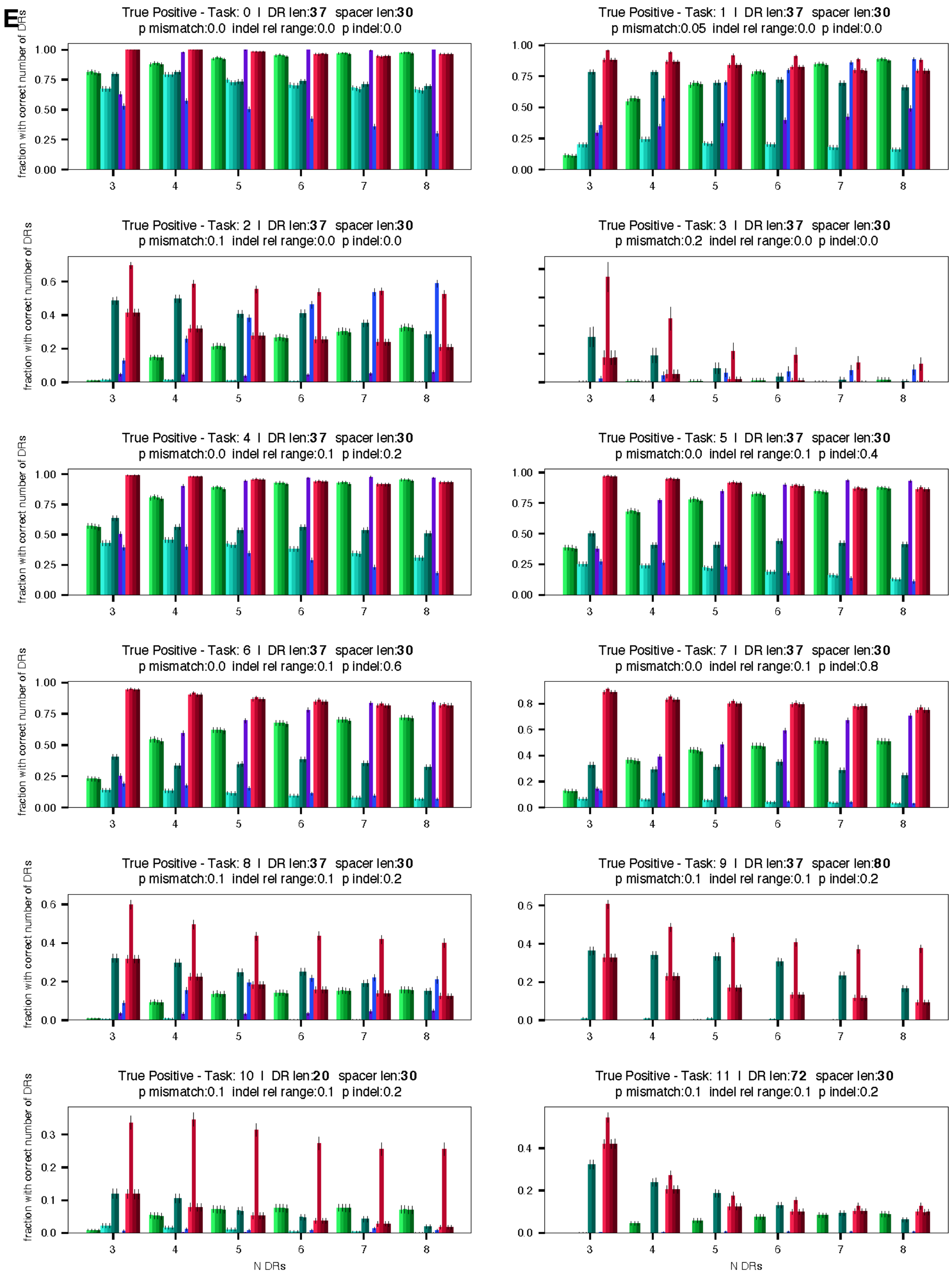
E
True Positive - Task: 0 I DR len:37 spacer len:30
p mismatch:0.0 indel rel range:0.0 p indel:0.0
True Positive - Task: 1 I DR len:37 spacer len:30
p mismatch:0.05 indel rel range:0.0 p indel:0.0
True Positive - Task: 2 I DR len:37 spacer len:30
p mismatch:0.1 indel rel range:0.0 p indel:0.0
True Positive - Task: 3 I DR len:37 spacer len:30
p mismatch:0.2 indel rel range:0.0 p indel:0.0
True Positive - Task: 4 I DR len:37 spacer len:30
p mismatch:0.0 indel rel range:0.1 p indel:0.2
True Positive - Task: 5 I DR len:37 spacer len:30
p mismatch:0.0 indel rel range:0.1 p indel:0.4
True Positive - Task: 6 I DR len:37 spacer len:30
p mismatch:0.0 indel rel range:0.1 p indel:0.6
True Positive - Task: 7 I DR len:37 spacer len:30
p mismatch:0.0 indel rel range:0.1 p indel:0.8
True Positive - Task: 8 I DR len:37 spacer len:30
p mismatch:0.1 indel rel range:0.1 p indel:0.2
True Positive - Task: 9 I DR len:37 spacer len:80
p mismatch:0.1 indel rel range:0.1 p indel:0.2
True Positive - Task: 10 I DR len:20 spacer len:30
p mismatch:0.1 indel rel range:0.1 p indel:0.2
True Positive - Task: 11 I DR len:72 spacer len:30
p mismatch:0.1 indel rel range:0.1 p indel:0.2
fraction with correct number of DRs
N DRs

Fig. S3 (cont'd)

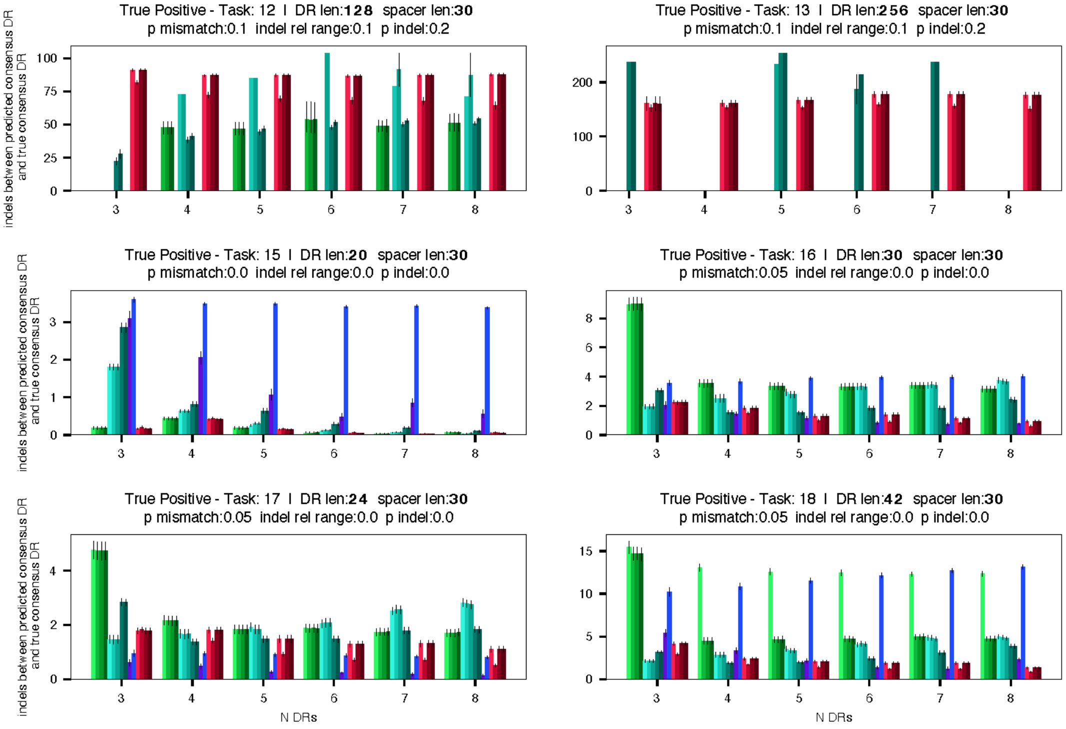
True Positive - Task: 12 I DR len:128 spacer len:30
p mismatch:0.1 indel rel range:0.1 p indel:0.2
True Positive - Task: 13 I DR len:256 spacer len:30
p mismatch:0.1 indel rel range:0.1 p indel:0.2
True Positive - Task: 15 I DR len:20 spacer len:30
p mismatch:0.0 indel rel range:0.0 p indel:0.0
True Positive - Task: 16 I DR len:30 spacer len:30
p mismatch:0.05 indel rel range:0.0 p indel:0.0
True Positive - Task: 17 I DR len:24 spacer len:30
p mismatch:0.05 indel rel range:0.0 p indel:0.0
True Positive - Task: 18 I DR len:42 spacer len:30
p mismatch:0.05 indel rel range:0.0 p indel:0.0
indels between predicted consensus DR and true consensus DR
N DRs

Fig. S3 (cont'd)

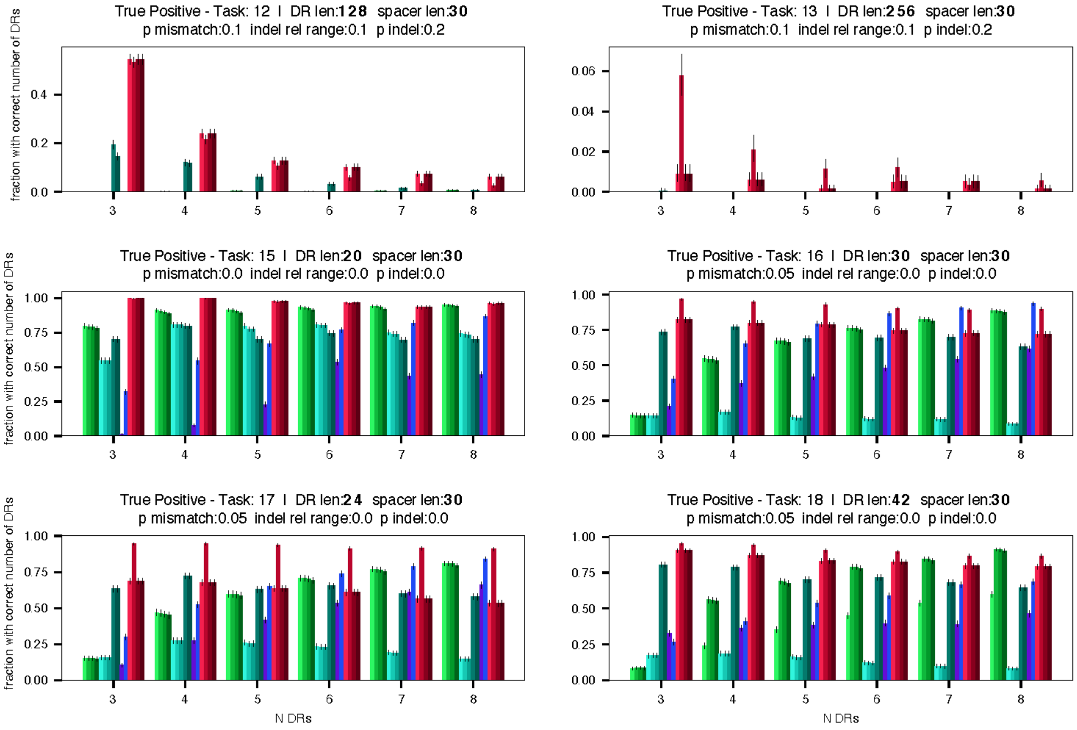

True Positive - Task: 12 I DR len:128 spacer len:30
p mismatch:0.1 indel rel range:0.1 p indel:0.2
True Positive - Task: 13 I DR len:256 spacer len:30
p mismatch:0.1 indel rel range:0.1 p indel:0.2
True Positive - Task: 15 I DR len:20 spacer len:30
p mismatch:0.0 indel rel range:0.0 p indel:0.0
True Positive - Task: 16 I DR len:30 spacer len:30
p mismatch:0.05 indel rel range:0.0 p indel:0.0
True Positive - Task: 17 I DR len:24 spacer len:30
p mismatch:0.05 indel rel range:0.0 p indel:0.0
True Positive - Task: 18 I DR len:42 spacer len:30
p mismatch:0.05 indel rel range:0.0 p indel:0.0
fraction with correct number of DRs
N DRs

Fig. S3 (cont'd)

F

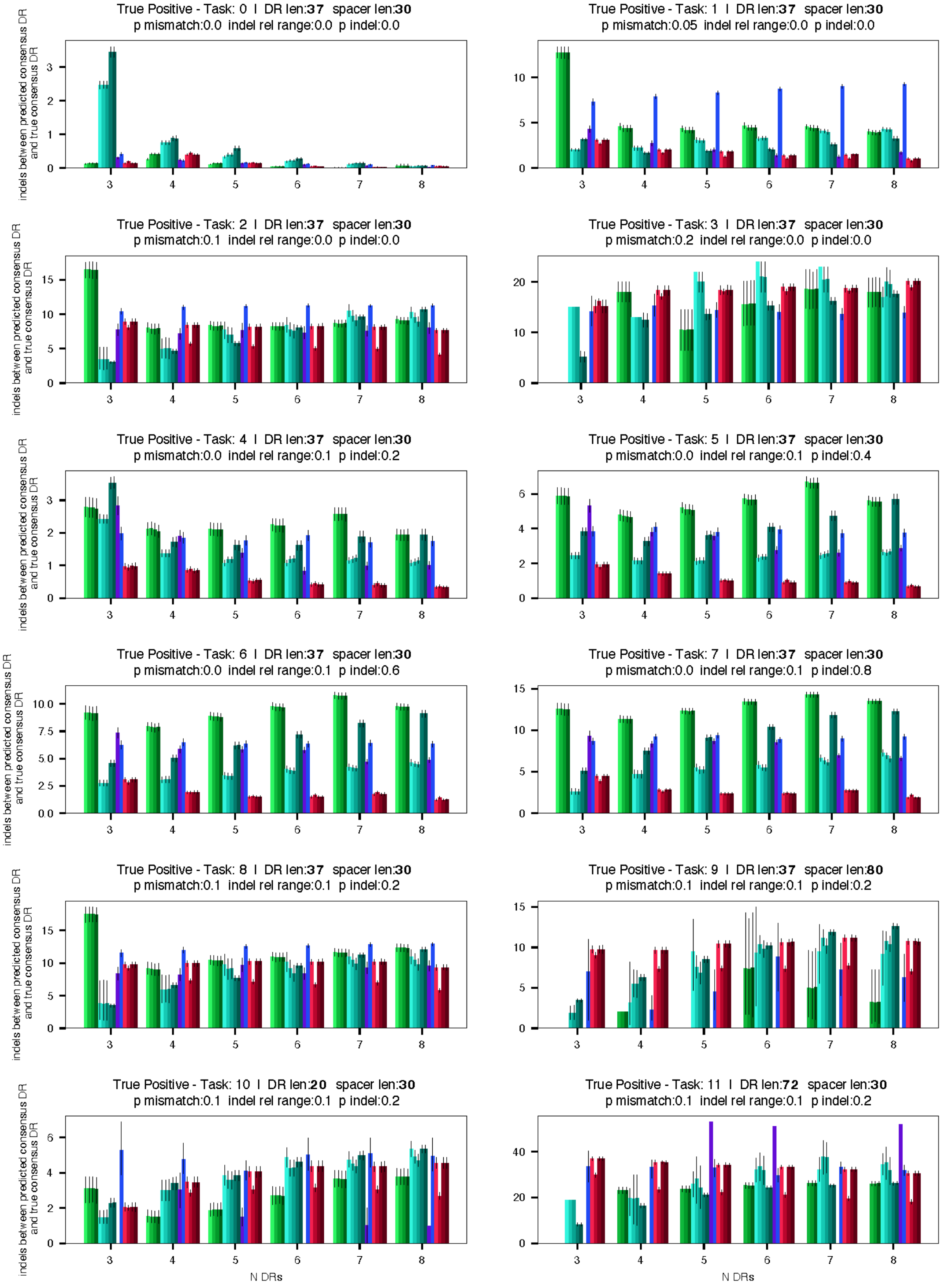

Fig. S3. Performance benchmarks of various CRISPR finders against synthetically generated CRISPRs

(A) Description of all parameter sets used for generating the 35 synthetic CRISPR array datasets. **(B)** Description of all of the CRISPR finder tools and their tested parameters for the benchmark along with their id/label (condition column). **(C)** Average runtime per ~20kb sequence for each of the CRISPR prediction tools. **(D)** Average recovery rates of all tools vs each of the synthetic CRISPR datasets. True Positives (real CRISPR-like arrays) vs False Positives (tandem repeats) are differentiated in the subplot titles. Error bars show 95% confidence bounds as determined by bootstrap with 2000 bootstraps. **(E)** Average fraction of correctly predicted number of DRs with error bars as in **(D)**. **(F)** average number of indels between predicted CRISPR DR and true CRISPR DR with error bars as in **(D)**.

Fig. S4.

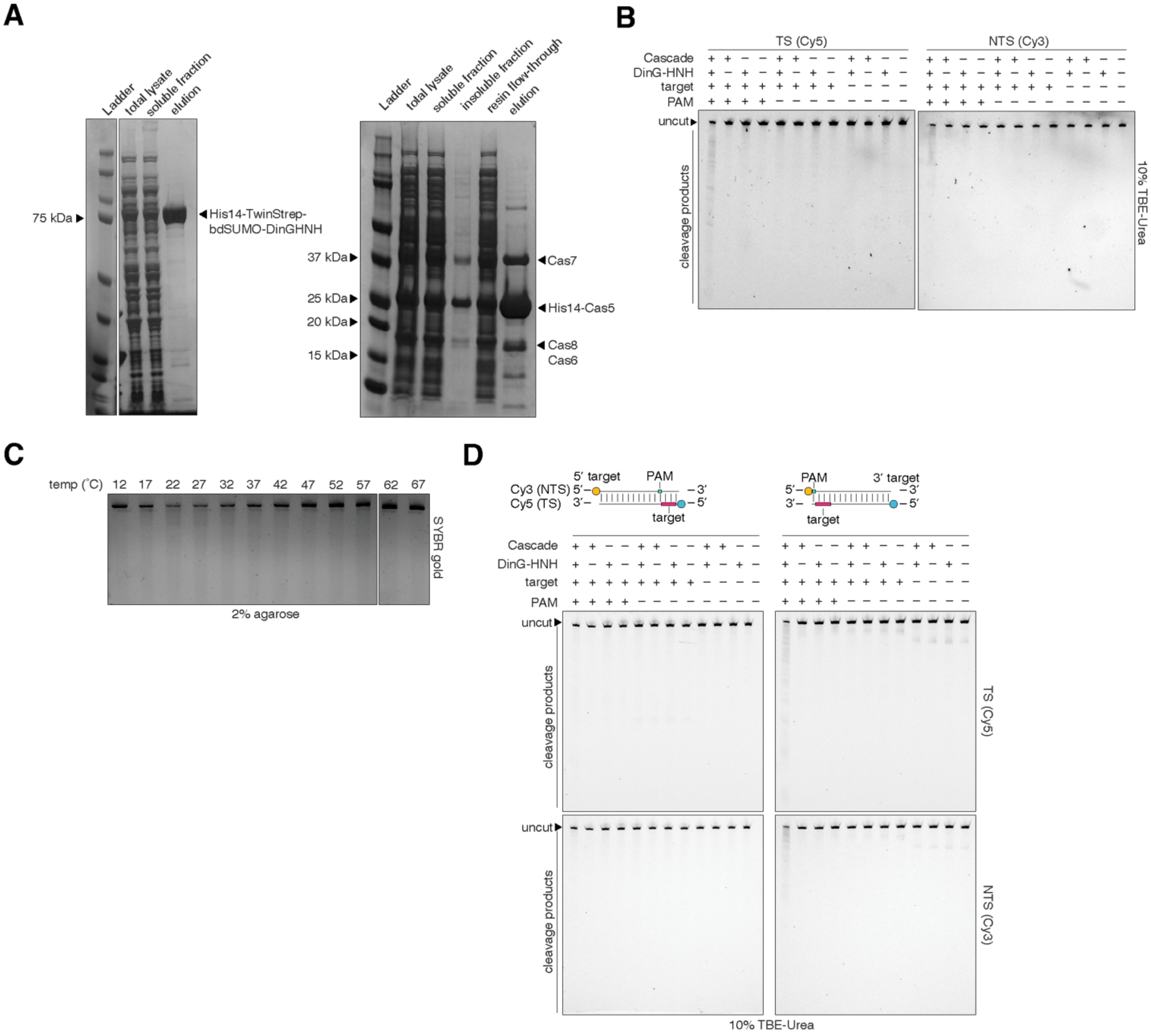

Fig. S4. Biochemical characterization of DinG-HNH system

(A) Affinity purification of DinG-HNH protein (left) and DinG-HNH-associated effector RNP complex (right). **(B)** Target dsDNA cleavage by DinG-HNH and associated effector RNP complex. Reactions were performed at 37°C, run on denaturing TBE-Urea gels and imaged in both the Cy5 and Cy3 channels as indicated. Cleavage of DNA, as indicated by laddering of products in both the Cy5 and Cy3 channels, was observed only when all protein components, the correct PAM and cognate protospacer target were present. **(C)** Target dsDNA cleavage by DinG-HNH and associated effector RNP complex at various temperatures. Products were analyzed on pre-SYBR Gold-stained 2% agarose gels. The greatest decrease in intensity of the uncleaved band was observed at ~22°C. **(D)** Target dsDNA cleavage by DinG-HNH and associated effector RNP complex with target sites placed on the 5′ or 3′ end of the target DNA species. Reactions

were performed at 22°C, run on denaturing TBE-Urea gels and imaged in both the Cy5 and Cy3 channels. The first four lanes of each gel display the same assay samples as shown in Fig. 3E.

Fig. S5.

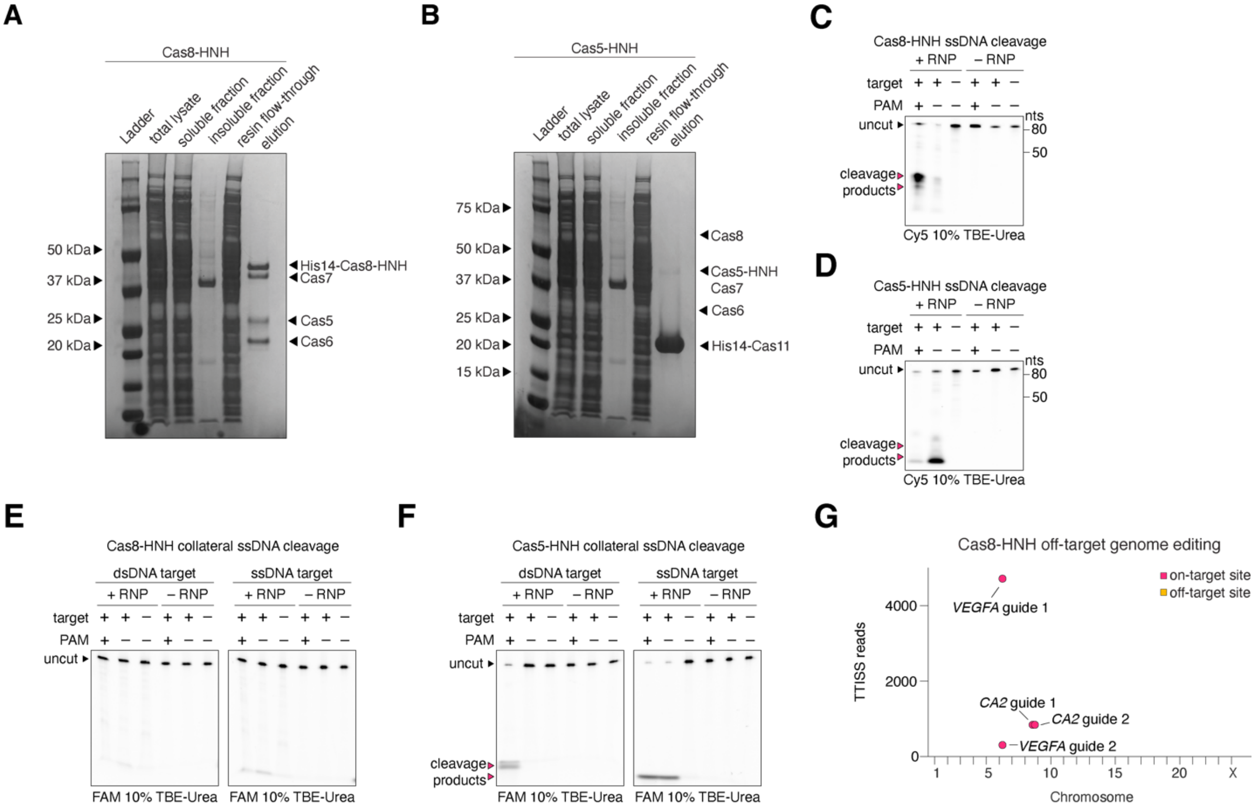

Fig. S5. Additional characterization of Cas8-HNH and Cas5-HNH effector complexes

SDS-PAGE gel electrophoresis of affinity purified **(A)** Cas8-HNH and **(B)** Cas5-HNH effector RNP complexes. **(C)** *In vitro* reconstituted Cas8-HNH Cascade RNP cleavage of linear ssDNA targets, in the presence or absence of a cognate target and/or PAM. + PAM: CCA; – PAM: AAG. **(D)** *In vitro* reconstituted Cas5-HNH Cascade RNP cleavage of linear ssDNA targets, in the presence or absence of a cognate target and/or PAM. + PAM: AAG; – PAM: CCA. **(E)** *In vitro* reconstituted Cas8-HNH Cascade RNP cleavage of collateral linear ssDNA substrates (FAM labeled), in the presence or absence of a dsDNA (left) or ssDNA (right) cognate target and/or PAM (unlabeled). + PAM: CCA; – PAM: AAG **(F)** *In vitro* reconstituted Cas5-HNH Cascade RNP cleavage of collateral linear ssDNA substrates (FAM labeled), in the presence or absence of a dsDNA (left) or ssDNA (right) cognate target and/or PAM (unlabeled). + PAM: AAG; – PAM: CCA. **(G)** TTISS off-target analysis of Cas8-HNH genome editing in HEK293FT cells for 4 guides.

Fig. S6.

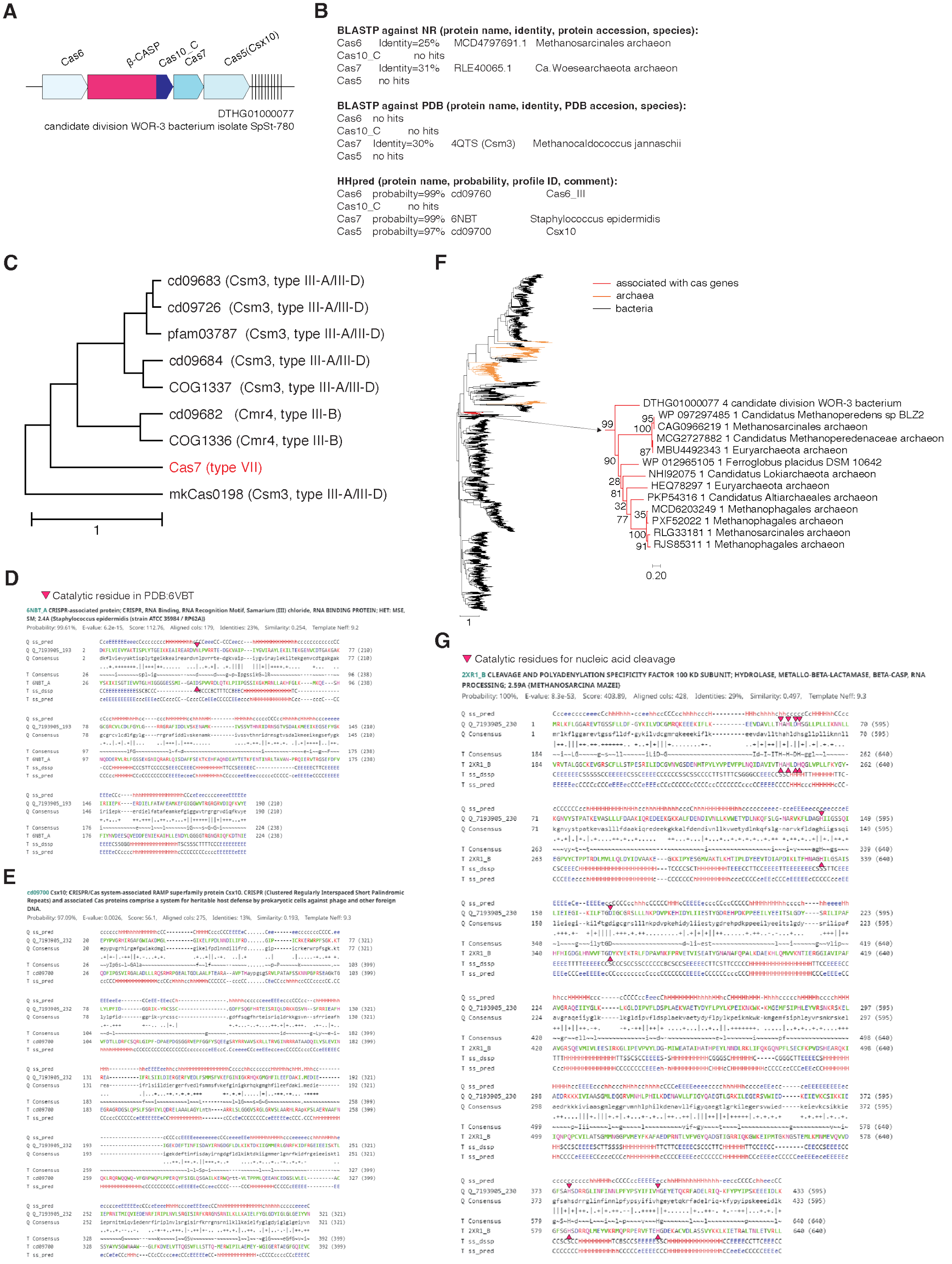
A
Cas6
β-CASP
Cas10_C
Cas7
Cas5(Csx10)
DTHG01000077
candidate division WOR-3 bacterium isolate SpSt-780
B
BLASTP against NR (protein name, identity, protein accession, species):
Cas6 Identity=25% MCD4797691.1 Methanosarcinales archaeon
Cas10_C no hits
Cas7 Identity=31% RLE40065.1 Ca.Woesearchaeota archaeon
Cas5 no hits
BLASTP against PDB (protein name, identity, PDB accesion, species):
Cas6 no hits
Cas10_C no hits
Cas7 Identity=30% 4QTS (Csm3) Methanocaldococcus jannaschii
Cas5 no hits
HHpred (protein name, probability, profile ID, comment):
Cas6 probabilty=99% cd09760 Cas6_III
Cas10_C no hits
Cas7 probabilty=99% 6NBT Staphylococcus epidermidis
Cas5 probabilty=97% cd09700 Csx10
C
cd09683 (Csm3, type III-A/III-D)
cd09726 (Csm3, type III-A/III-D)
pfam03787 (Csm3, type III-A/III-D)
cd09684 (Csm3, type III-A/III-D)
COG1337 (Csm3, type III-A/III-D)
cd09682 (Cmr4, type III-B)
COG1336 (Cmr4, type III-B)
Cas7 (type VII)
mkCas0198 (Csm3, type III-A/III-D)
1
D
Catalytic residue in PDB:6VBT
E
F
associated with cas genes
archaea
bacteria
DTHG01000077 4 candidate division WOR-3 bacterium
WP 097297485 1 Candidatus Methanoperedens sp BLZ2
CAG0966219 1 Methanosarcinales archaeon
MCG2727882 1 Candidatus Methanoperedenaceae archaeon
MBU4492343 1 Euryarchaeota archaeon
WP 012965105 1 Ferroglobus placidus DSM 10642
NHI92075 1 Candidatus Lokiarchaeota archaeon
HEQ78297 1 Euryarchaeota archaeon
PKP54316 1 Candidatus Altiarchaeales archaeon
MCD6203249 1 Methanophagales archaeon
PXF52022 1 Methanophagales archaeon
RLG33181 1 Methanosarcinales archaeon
RJS85311 1 Methanophagales archaeon
99
95
100
87
90
28
81
32
35
77
100
91
0.20
1
G
Catalytic residues for nucleic acid cleavage

Fig. S6. (cont'd)

H

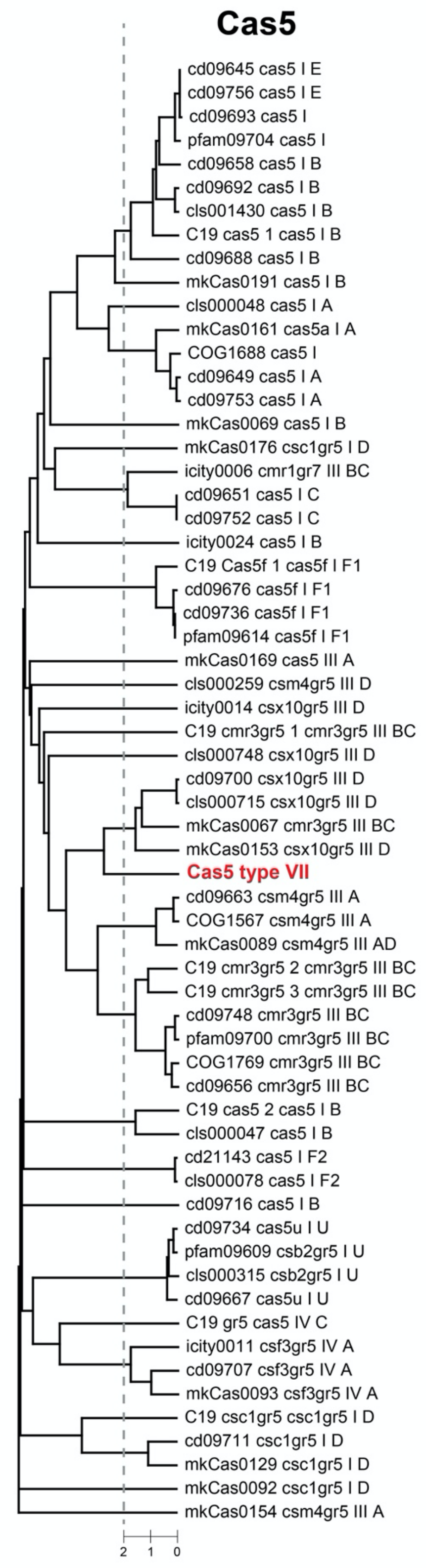

I

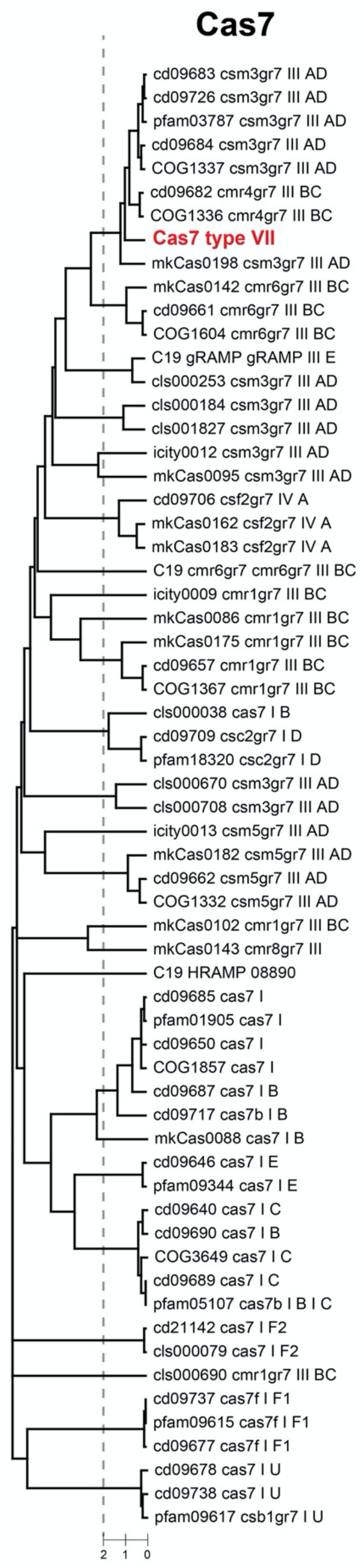

Fig. S6. Analysis of candidate type VII proteins

(A) Locus diagram of a candidate type VII locus in candidate division WOR-3 bacterium isolate SpSt-780. **(B)** Top BLASTP (against NR and PDB) and HHPred results for each of the four components of the candidate type VII system. **(C)** UPGMA tree of representative Cas7 homologs across type III CRISPR and type VII systems. **(D)** Top HHpred result of Cas7 from candidate type VII systems. The catalytic aspartate (red triangles) is not conserved and is mutated to asparagine, suggesting that Cas7 is not capable of RNA cleavage as it is in many type III CRISPR systems. **(E)** Top HHpred result for Cas5, which is most similar to the type III-D Cas5 homolog Csx10. **(F)** FastTree phylogenetic analysis of representative β-CASP domain proteins from bacteria and archaea. Cas14 proteins form a single clade, shown in red. **(G)** Top HHpred result for Cas14. The N-terminal β-CASP domain of Cas14 is similar to the yeast cleavage and polyadenylation specificity factor 100. Catalytic residues that coordinate Zn^{2+} ions required for catalysis are marked by red triangles. **(H)** Broader HHSuite distance-based UPGMA tree of Cas5 proteins from all class 1 systems based on HMM profiles. **(I)** Broader HHSuite distance-based UPGMA tree of Cas7 proteins from all class 1 systems based on HMM profiles.

Fig. S7.

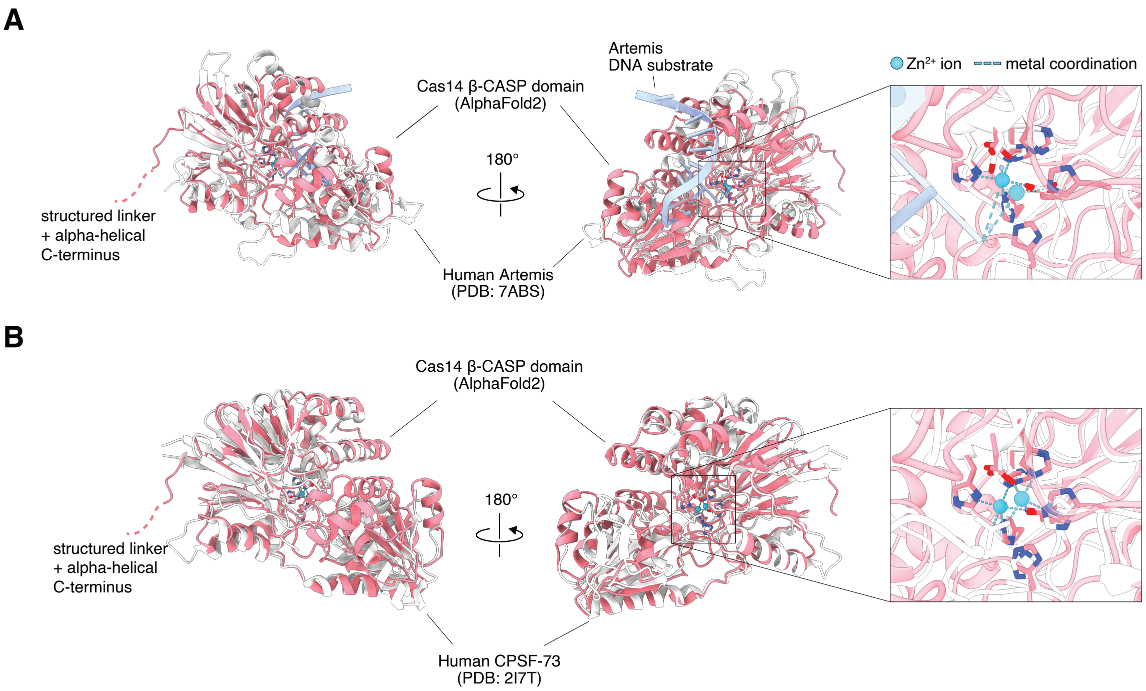

Fig. S7. Structural comparison of Cas14 β-CASP domain of and human β-CASP protein

Superimposition of **(A)** AlphaFold2 prediction of Cas14, with only the β-CASP domain shown, and the X-ray crystal structure of the human Artemis protein in complex with a DNA substrate (PDB: 7ABS) and **(B)** AlphaFold2 prediction of Cas14, with only the β-CASP domain shown, and the X-tray crystal structure of the human CPSF-73 protein. Insets show the catalytic centers, which coordinate two Zn^{2+} ions (blue). Catalytic residues responsible for Zn^{2+} coordination are shown as sticks with colored heteroatoms.

Fig. S8.

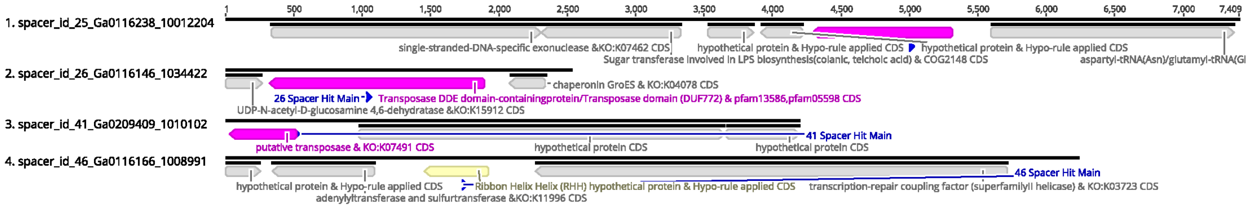

Fig. S8. Spacer matches for candidate type VII system

Four examples of spacers from CRISPR arrays associated with the candidate type VII system, with matching protospacers in predicted transposon genes.

Fig. S9.

A

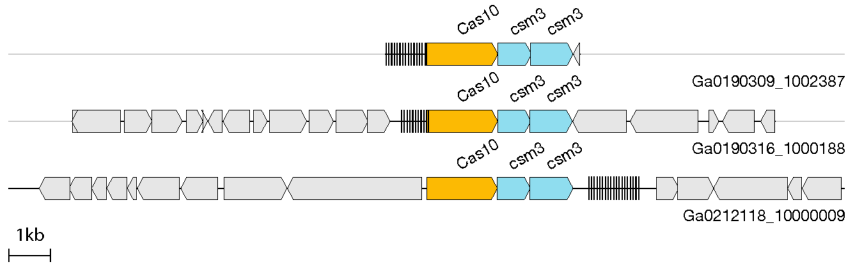

B

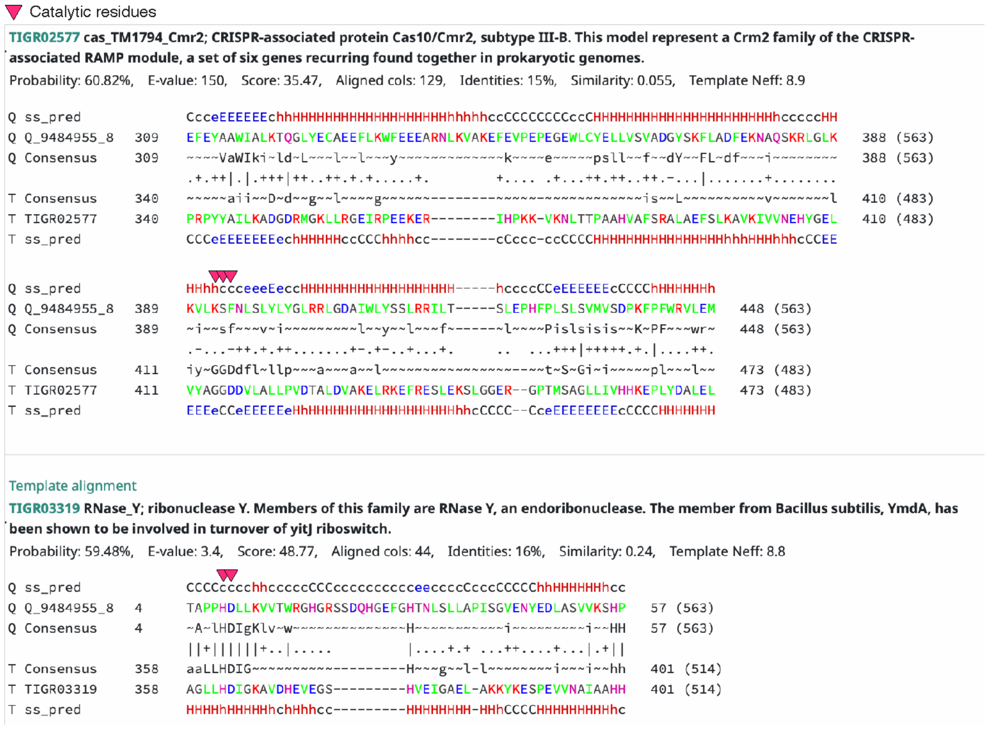

Fig. S9. New candidate subtype of type III CRISPR

(A) Locus schematics of a newly identified type III CRISPR candidate subtype containing 3 proteins associated with CRISPR arrays, namely a Cas10 protein, and two Csm3/Cas7-like proteins, one of which may be Cas5-like. The gene arrangement is reminiscent of the candidate type VII, but contains a Cas10 effector and is evolutionarily distinct, likely originating from distinct proteins. (B) HHpred results of polymerase/cyclase domain and HD nuclease domain of Cas10. The catalytic residues of the polymerase/cyclase are not conserved, suggesting this Cas10 does not actively synthesize cyclic oligonucleotides, while the HD nuclease catalytic residues are intact, suggesting the Cas10 can still cleave DNA.

Fig. S10.

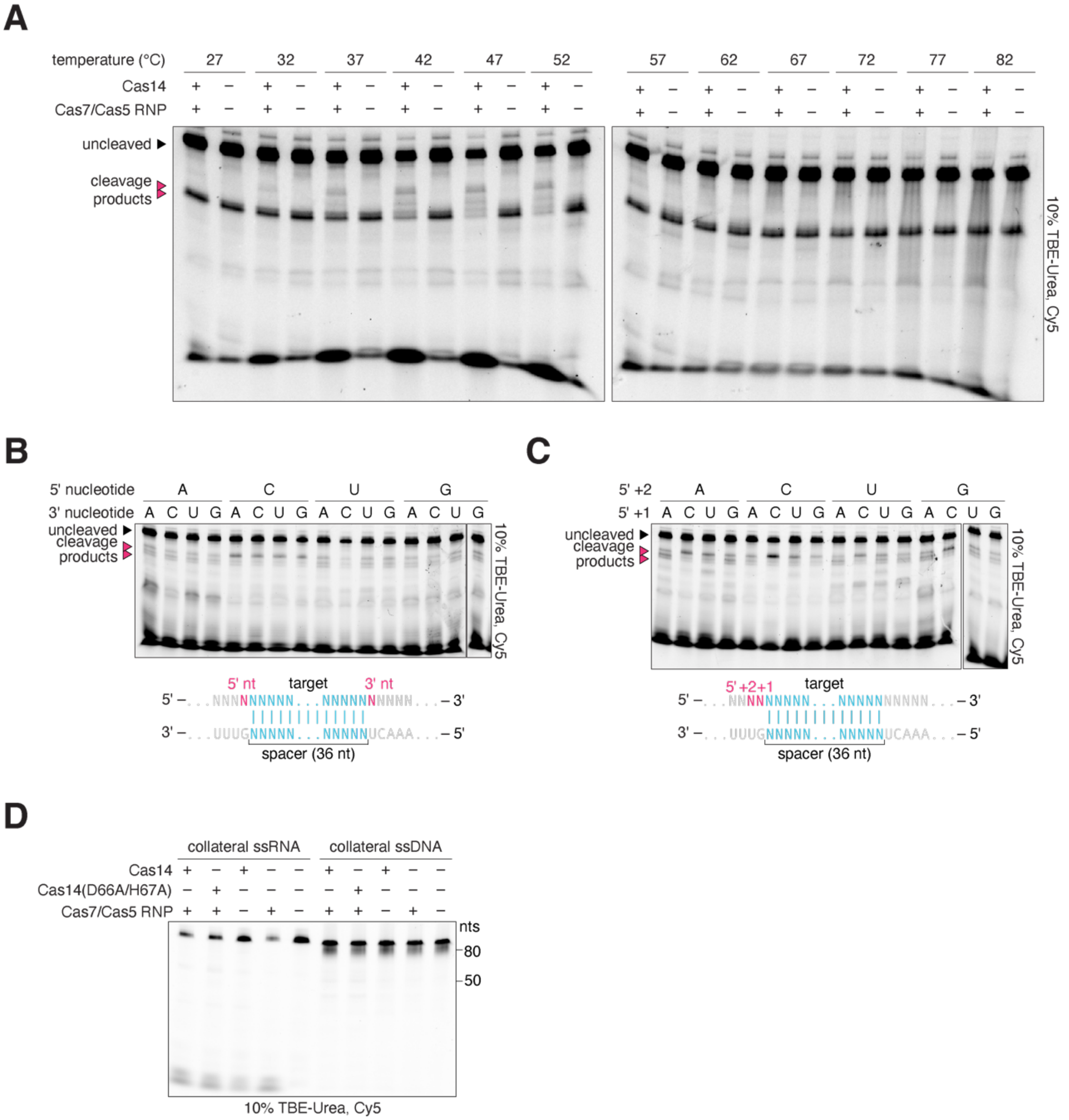

Fig. S10. RNA cleavage by candidate type VII system.

(A) Target RNA cleavage by Cas14 and associated Cas7/Cas5 RNP at various temperatures. Cleavage is apparent in a range from 37°C to 52°C. Targets contain a 3N degenerate sequence on either side of the protospacer. Reactions were conducted at 45°C for all subsequent experiments. **(B)** Cleavage of target RNAs with all combinations of 5' and 3' flanking sequences. Targets with a 5' C are preferred. **(C)** Cleavage of target RNAs with all 5' flanking dinucleotide combinations. Targets with a C directly 5' of the protospacer are preferred. **(D)** Candidate type VII system incubated with collateral ssRNA or ssDNA substrates in the presence of a matching target. Collateral substrates are labeled with Cy5, and matching targets are unlabeled. No collateral cleavage of either ssRNA or ssDNA was observed.

Fig. S11.

Type	Type I			Type III		Type IV	Type VII
Subtype	Typical	I-D	CAST (I-F)	Typical	Type III-E		
Effector Surveilence Complex	Cas7, Cas5, Cas8, [...Cas11]	Cas7, Cas5, [...Cas11]	Cas7, Cas5, Cas8	multiple Cas7s, multiple Cas11s, Cas5	fusion of: multiple Cas7s + multiple Cas11s + Cas5	Cas7, Cas5, Cas8-like, Cas11	Cas7, Cas5
Interference	Cas3	Cas3'' (HD) + Cas10d fusion, [Cas3]		Cas7, Cas10+HD	Cas7	Cas10+HD, or DinG	Cas14 (b-CASP)
Downstream cascade			Tn7 transposase	Cas10 (coA sigalling)	Csx29 signal cascade		
Target	DNA	DNA	DNA	RNA, ssDNA	RNA	DNA	RNA
Biological Mechanism	Cas3 recruitment for cleavage via Cas3 HD	Recruitment of Cas3''/Cas10d fusion for cleavage via Cas3'' HD		Cas7 RNase, Cas10 recruitment for cleavage via HD	Cas7 RNase, Csx29 recruitment for proteolytic cleavage of Csx30	DinG recruitment for DNA unwinding / Cas10 recruitment for cleavage via HD	putative: Cas14 recruitment for cleavage via b-CASP

Cas10 C-terminus homologus to Cas11
Cas15 C-terminus homologus to Cas11/Cas10 C-terminus
Cas3 is composed of a helicase (Cas3') and an HD domain Cas3'', which is homologous to the HD domain from Cas10
*Cas6s are involved in CRISPR RNA processing and often interacts with effector complexes

Type	Type II	Type V		Type VI
Subtype		Typical	CAST (V-K)	
Effector Surveilence Complex	Cas9	Cas12	Cas12	Cas13
Interference	Cas9	Cas12		Cas13
Downstream cascade			Tn7 transposase	
Target	DNA	DNA / RNA	DNA	RNA
Biological Mechanism	Cas9 binding for cleavage via RuvC and HNH	Cas12 binding for cleavage via RuvC	Cas12-mediated recruiment of Tn7 transposon via TniQ	Cas13 binding for cleavage via HEPN

Fig. S11. Comparison of CRISPR types along with candidate type VII CRISPR type

Comparison of various essential system characteristics of different CRISPR types, namely proteins involved in the effector surveillance complex and the proteins involved in the target cleavage interference mechanism. Additionally shown are potential downstream cascade events that may take place for a given system, as well as the target nucleic acid type, and the biological mechanism for the system's activity.

Fig. S12. Index of all systems identified in this study

First row is the designation of the system. Second row is the set of defining domains of the system. Third row contains the enhanced CRISPR association score (if the system is contained in the passing sets). Some systems were included if they were identified through protein-protein associations, even if their enhanced CRISPR-association score did not meet the filtering threshold.

Note: Fig. S12 spans pages **55 to 58**

effector modules

III-UAS
(new III subtype)
6 / 7.0

PhageCas9
(PhageCas9 + Helicase)
N/A

CasMu-I
(CasMu-I)
20 / 46.0

Cas12_DUF3800_TPR_UvrD_TPR
(DUF3800_TPR + Cas12)
11 / 12.0

UAS-36
(Cas3_PDDEXK)
3 / 3.0

Cas11-VRR
(Cse2_VRR)
7 / 7.2

VII
(VII b-CASP)
10 / 16.3

Cas5-HNH
(Cas5_HNH)
161 / 243.7

CasMu-V
(CasMuV MuB)
2 / 4.0

Cas12-Cas3
(Cas12 + Cas3)
7 / 8.0

Cas12-IscB
(Cas12 + IscB + HTH)
4 / 9.5

Cas8-HNH
(Cas8_HNH)
5 / 8.0

DinG-HNH
(DinG_HNH)
111 / 129.0

cas fusions

Cas8-Cas5
(Cas7 + Cas8_Cas5 fusion)
3 / 4.7

UAS-37
(Cas9_Cas1 fusion)
3 / 3.0

Cas5-Cas3
(Cas5_Cas3 fusion)
27 / 27.7

UAS-38
(Cmr6_Cmr1 fusion)
24 / 37.1

UAS-39
(Cas6_Cas5_fusion)
4 / 4.0

UAS-40
(Csa5_Csa4 fusion)
4 / 6.1

UAS-41
(Cas8_Cas5 fusion)
4 / 4.0

acquisition

UAS-42
(DUF3761)
22 / 22.7

UAS-43
(RVT + group II intron PrimPol)
14 / 15.7

UAS-3
(DUF370)
2 / 3.5

UAS-7
(vWA + RDD)
2 / 4.7

UAS-2
(Tbf2 WYL)
9 / 13.8

UAS-44
(DUF2797 large zinc finger protein)
8 / 9.2

UAS-45
(RNaseH)
11 / 21.5

UAS-13
(DUF2797)
6 / 7.9

UAS-4
(DUF1882_primase)
15 / 16.5

UAS-5
(DUF4384 + TPR)
38 / 43.4

UAS-46
(DUF3761)
11 / 13.8

UAS-10
(HlfK)
11 / 23.1

UAS-8
(23S rRNA IVP + DUF2325 + RT sometimes)
18 / 22.0

UAS-11
(type II secretion GspH)
8 / 8.5

UAS-17
(Tbf2)
13 / 32.9

UAS-47
(WYL)
35 / 37.0

UAS-1
(Ras GTPase)
8 / 14.3

UAS-48
(Cas2 + 3'-_5' proofreading exonuclease)
16 / 17.0

UAS-12
(FlhB)
3 / 5.0

UAS-9
(DUF4407 + other protein)
23 / 39.3

UAS-14
(SWIM)
3 / 6.5

UAS-6
(Acquisition-associated NACHT)
25 / 40.6

UAS-16
(DUF3761)
9 / 12.0

UAS-15
(TPR + DNA polA)
10 / 14.3

UAS-49
(large WYL)
51 / 53.9

CARF effectors

UAS-50
(CARF_RelA)
8 / 11.5

UAS-51
(SAVED_TIR2)
4 / 5.2

UAS-52
(CARF_Sigma)
N/A

UAS-53
(CARF_DUF859 + many nearby genes)
4 / 5.0

UAS-54
(SIR2 _ CHAT _ SAVED + SMODS)
5 / 10.3

UAS-55
(EAD7 _ SAVED)
2 / 5.6

UAS-56
(CARF_DUF859_ATPase + many nearby genes)
7 / 8.7

UAS-57
(SAVED TIR)
9 / 18.6

UAS-58
(CARF_ATPase_HTH + RNAPol)
N/A

UAS-59
(CARF_CYTH_HD)
3 / 5.1

UAS-60
(CARF_SIR2)
N/A

UAS-61
(CARF_TIR_Mrr)
11 / 15.3

UAS-62
(csx1 + Phage RNAPol)
5 / 12.2

UAS-63
(CARF_2p_deoxyribosyltransferase)
5 / 7.7

UAS-64
(CARF_EAD7)
N/A

UAS-65
(CARF_5p_nucleotidase)
6 / 6.6

UAS-66
(SAVED_APH_TCAD9)
4 / 4.5

UAS-67
(Cutinase_Lipase_SAVED)
4 / 4.7

auxiliary systems

UAS-68
(SMODS + SAVED CHAT)
N/A

UAS-69
(methyltransferase)
3 / 7.0

UAS-70
(vATP-synt_E)
9 / 9.3

UAS-71
(NERD)
4 / 4.7

UAS-72
(multi-gene system including greA greB)
20 / 26.7

UAS-73
(Mrr nuclease)
7 / 10.5

UAS-74
(PKinase)
2 / 3.4

UAS-75
(Sigma_factor + Csx1 + large Csx1)
7 / 7.5

UAS-76
(HTH _ M78 peptidase)
41 / 53.8

UAS-77
(DUF2283)
3 / 5.5

UAS-78
(Nif11)
7 / 7.7

UAS-79
(XRE HTH regulator operonized with Cas effectors)
8 / 8.9

UAS-80
(RepA replication)
5 / 5.1

UAS-81
(DUF4145)
3 / 3.0

UAS-82
(pentapeptide + unknown domain)
4 / 7.6

UAS-83
(Recep domain + 4 unknown genes)
8 / 8.0

UAS-84
(MazF)
2 / 5.3

UAS-31
(Kinase)
10 / 16.4

UAS-85
(betalactamase)
2 / 5.5

UAS-86
(large Sigma_factor)
3 / 4.0

UAS-87
(vwa + PPC + kinase system)
14 / 25.6

UAS-24
(RNaseT)
3 / 3.0

UAS-88
(Sigma_factor + Csx1)
6 / 7.2

UAS-19
(Cas9 or Cas12 associated WYL)
19 / 35.1

UAS-23
(unknown + DUF2791 + DUF2791_ATPase)
3 / 6.5

UAS-89
(unknown accessory)
8 / 13.6

UAS-90
(nurA dsDNA break repair + DUF87)
9 / 19.4

UAS-91
(vWA + FHA + PP2C)
3 / 6.8

UAS-92
(DUF2281)
13 / 17.6

UAS-93
(PAP_phosphatase_CorA)
63 / 127.1

UAS-28
(Y1 TPase type III)
6 / 7.3

UAS-94
(DUF3800_TPR)
8 / 14.3

UAS-95
(RAMA + DUF444 + PIN)
25 / 34.3

UAS-96
(unknown transmembrane protein)
N/A

UAS-97
(Fic Cascade)
2 / 4.2

UAS-98
(TPR_MALT)
5 / 6.3

UAS-30
(UvrD_NERD nuclease)
6 / 6.0

UAS-99
(Radical SAM protein)
20 / 37.7

UAS-100
(wWA + kinase)
6 / 9.9

UAS-101
(DUF2204)
19 / 47.0

UAS-102
(DUF5343 + Helicase_HNH_ResIII)
3 / 3.0

UAS-103
(TPR HATPase)
2 / 3.0

UAS-104
(ATPase + Metallophosphatase)
3 / 3.0

UAS-105
(M78 peptidase)
5 / 5.6

UAS-106
(DUF3800)
3 / 3.0

UAS-107
(Toprim)
17 / 25.5

UAS-108
(DUF433)
7 / 16.3

UAS-109
(DUF4407 + transpeptidase)
2 / 5.3

UAS-110
(mega peptidase)
2 / 3.5

UAS-111
(TIR cascade)
2 / 4.4

UAS-29
(Phage RNAPol)
4 / 8.6

UAS-112
(Csx20 operonized with Alpha Amylase)
N/A

UAS-113
(RNaseH)
10 / 14.6

UAS-114
(Tfb2_Cas3Cterminus_WYL)
5 / 5.5

UAS-115
(SLATT)
N/A

UAS-116
(Moca oxoreductase + SDR)
21 / 38.1

UAS-117
(DUF4231)
3 / 4.8

UAS-118
(DUF3891)
12 / 23.7

UAS-119
(DUF5679, zinc ribbon)
6 / 9.9

UAS-120
(DUF2225)
2 / 4.1

UAS-121
(RNAPol + Sigma_factor + unknown gene)
15 / 41.0

UAS-122
(PRiA3 + zf)
15 / 36.2

UAS-22
(Cut8_TPR)
3 / 8.1

UAS-21
(Cas13b + CorA)
3 / 3.7

UAS-123
(NACHT)
2 / 3.2

UAS-124
(unknown)
3 / 3.3

UAS-125
(UPF0158)
11 / 24.8

UAS-126
(Cut8)
3 / 6.4

UAS-26
(DUF87 + NurA 5' to 3' nuclease)
4 / 4.0

UAS-127
(NACHT)
3 / 7.4

UAS-128
(Mrr nuclease)
N/A

UAS-129
(Tubulin + other protein + vWa)
16 / 43.6

UAS-130
(Tubulin system)
5 / 13.1

UAS-131
(TIR2)
8 / 18.9

UAS-132
(Cas9 associated Y1)
20 / 27.4

UAS-133
(TPR Rho RNA binding)
5 / 6.4

UAS-18
(toprim)
4 / 6.6

UAS-134
(WYL operonized with cascade)
15 / 29.8

UAS-135
(ATPase_DUF4435 + Cas12)
3 / 3.0

UAS-136
(DUF6155)
4 / 6.7

UAS-137
(Holliday Junction Resolvase)
6 / 6.7

UAS-138
(TIR)
5 / 11.2

UAS-139
(metal system)
3 / 4.3

UAS-140
(DUF5919)
2 / 3.0

UAS-141
(PRiA4 ORF3)
2 / 3.9

UAS-142
(TPR Protein Kinase)
3 / 7.2

UAS-143
(EamA _ F420H2_quin_red)
3 / 3.0

UAS-144
(TPR)
24 / 48.9

UAS-145
(transmembrane NARF)
5 / 10.7

UAS-25
(NACHT_PDDEXK + cascade)
8 / 12.8

UAS-146
(EAD11)
5 / 8.2

UAS-147
(WYL I-D)
36 / 66.5

UAS-27
(methyltransferase + cascade)
2 / 3.2

UAS-148
(mega Cas8-like)
37 / 51.3

UAS-149
(vWA + Tubulin)
N/A

UAS-150
(APH_EcKinase)
3 / 3.0

UAS-151
(Cas8_Fic fusion)
2 / 5.0

UAS-152
(RNaseT)
4 / 7.4

UAS-153
(SNF2 instead of Cas3)
7 / 9.9

UAS-154
(HIRAN Cas9, one case ATPase)
28 / 44.9

UAS-20
(Cas12 + bzip)
7 / 7.4

UAS-155
(transmembrane_vWA + DUF4407)
3 / 4.4

UAS-156
(Mrr nuclease + ATPase)
N/A

UAS-33
(Prok_E2 + ThiF + ThiS + NYN + CorA)
34 / 50.0

UAS-157
(RecJ_HD)
19 / 25.4

toxin-antitoxin

UAS-34
(DarT DarG toxin-antitoxin ribosylation)
15 / 22.9

UAS-35
(addiction gene orf associated with Cas9)
9 / 9.0

UAS-158
(AbiE + DUF87)
8 / 18.7

UAS-159
(Nucleotidyltransferase HEPN, antitoxin)
18 / 48.1

UAS-160
(DUF86 + Nucleotidyltransferase)
104 / 173.3

UAS-161
(Cas9 HicAB TA)
3 / 3.0

UAS-162
(Cas9 TA system)
25 / 36.4

UAS-163
(nucleotidyltransferase + HTH)
4 / 6.9

UAS-164
(AbiEii)
2 / 5.2

UAS-165
(TA DUF3368 _ UPF0175)
11 / 16.6

UAS-166
(AbiEi + AbiEii)
N/A

UAS-167
(TA system)
7 / 7.3

UAS-168
(PHD + PIN TA)
3 / 3.9

UAS-169
(DUF370 + RNaseH TA)
3 / 5.2

UAS-170
(DUF2442 + DUF4160)
3 / 3.0

UAS-171
(small ORFs)
43 / 48.3

UAS-172
(RHH TA system)
23 / 35.0

UAS-173
(DUF3368 TA system)
42 / 56.2

massive

M2
(massive defense genes)
3 / 3.0

M1
(massive defense system)
6 / 13.3

tRNA arrays

(GGDEF tRNA array)
174 / 347.6

(tRNA array DEDDh)
483 / 551.2

(tRNA array ribonuclease)
104 / 112.6

hypervariable interspersed arrays

(DUF3800 standalone)
12 / 12.0

(PDDEXK + Metallophosphatase)
44 / 47.4

(PDDEXK_ATPase)
37 / 39.4

(PLMP_corA-like)
37 / 48.3

(DUF222_HNH)
51 / 111.4

(GGDEF + MFS + Phospholipase)
25 / 35.5

Fig. S13. Representative loci of all systems identified in this study

One representative locus was selected for each system and displayed along with the genes that are considered to be associated (red colored genes). The cluster id below the characteristic domains associated with the system (top middle bold for each system) is shown with regular font and corresponds to the gene with the asterisk. The designated name for the system is shown on the upper left. Gene names for newly identified genes, as applicable, are shown below the genes in bold. All gene names follow the format uas#A, uas#B,… corresponding to the genes that make up the system UAS-#. When space is limited, the uas# portion is omitted visually, but the underlying gene name is still uas-#A, etc. For example, a gene displayed with **C** underneath it, but belonging to UAS-293 would have a gene name of uas293C. Signature effector genes are colored in darker blue. Other cas genes are shown as light blue. Cas6 is shown as light green. Unrelated or other genes are shown as light grey. White genes with shorter height are genes that overlap with CRISPR arrays (vertical black lines). Above each gene are redundancy reduced predictions of the protein domains via HMMER on the PFAM database. Enhanced CRISPR association scores for the cluster ids are shown as applicable: some systems were included if they were identified through protein-protein associations, or exceptionally low association score (in the case of PhageCas9), even if their enhanced CRISPR-association score did not meet the filtering threshold, in which case the CRISPR association-scores are not shown.

Note: Fig. S13 spans pages **60 to 85**

Sub table of contents

Effector Modules 60

Cas Fusions 62

Acquisition 63

CARF 66

Auxiliary 69

Toxin-Antitoxin 81

Massive Genes 83

Non-CRISPR tRNA arrays 84

Non-CRISPR arrays with hypervariable spacers 85

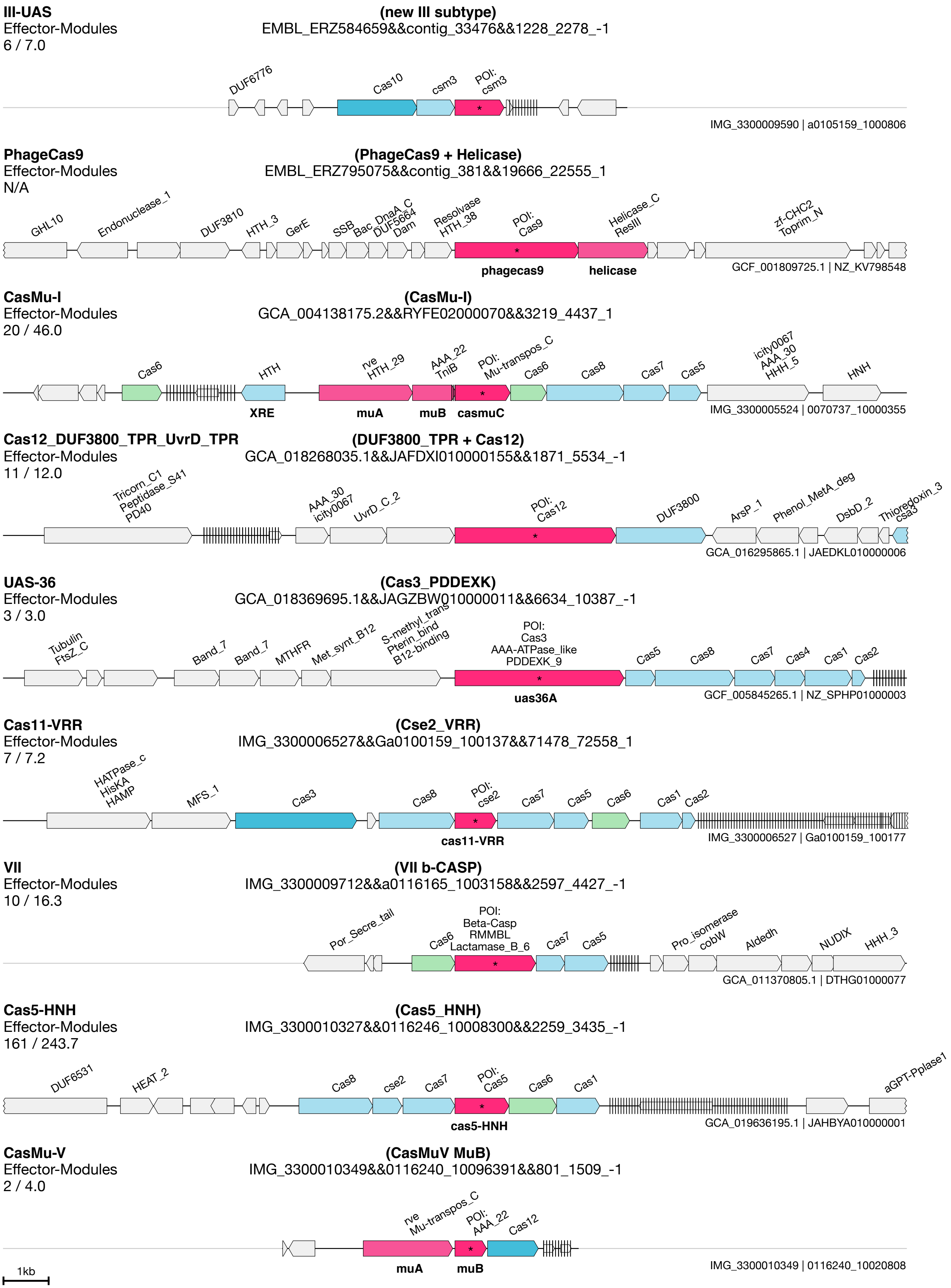
III-UAS
Effector-Modules
6 / 7.0
(new III subtype)
EMBL_ERZ584659&&contig_33476&&1228_2278_-1
DUF6776
Cas10
csm3
POI:
csm3
IMG_3300009590 | a0105159_1000806
PhageCas9
Effector-Modules
N/A
(PhageCas9 + Helicase)
EMBL_ERZ795075&&contig_381&&19666_22555_1
GHL10
Endonuclease_1
DUF3810
HTH_3
GerE
SSB
Bac_DnaA_C
DUF5664
Dam
Resolvase
HTH_38
POI:
Cas9
Helicase_C
ResIII
zf-CHC2
Toprim_N
phagecas9
helicase
GCF_001809725.1 | NZ_KV798548
CasMu-I
Effector-Modules
20 / 46.0
(CasMu-I)
GCA_004138175.2&&RYFE02000070&&3219_4437_1
Cas6
HTH
rve
HTH_29
AAA_22
TniB
POI:
Mu-transpos_C
Cas6
Cas8
Cas7
Cas5
icity0067
AAA_30
HHH_5
HNH
XRE
muA
muB
casmuC
IMG_3300005524 | 0070737_10000355
Cas12_DUF3800_TPR_UvrD_TPR
Effector-Modules
11 / 12.0
(DUF3800_TPR + Cas12)
GCA_018268035.1&&JAFDXI010000155&&1871_5534_-1
Tricorn_C1
Peptidase_S41
PD40
AAA_30
icity0067
UvrD_C_2
POI:
Cas12
DUF3800
ArsP_1
Phenol_MetA_deg
DsbD_2
Thioredoxin_3
csa3
GCA_016295865.1 | JAEDKL010000006
UAS-36
Effector-Modules
3 / 3.0
(Cas3_PDDEXK)
GCA_018369695.1&&JAGZBW010000011&&6634_10387_-1
Tubulin
FtsZ_C
Band_7
Band_7
MTHFR
Met_synt_B12
S-methyl_trans
Pterin_bind
B12-binding
POI:
Cas3
AAA-ATPase_like
PDDEXK_9
Cas5
Cas8
Cas7
Cas4
Cas1
Cas2
uas36A
GCF_005845265.1 | NZ_SPHP01000003
Cas11-VRR
Effector-Modules
7 / 7.2
(Cse2_VRR)
IMG_3300006527&&Ga0100159_100137&&71478_72558_1
HATPase_c
HisKA
HAMP
MFS_1
Cas3
Cas8
POI:
cse2
Cas7
Cas5
Cas6
Cas1
Cas2
cas11-VRR
IMG_3300006527 | Ga0100159_100177
VII
Effector-Modules
10 / 16.3
(VII b-CASP)
IMG_3300009712&&a0116165_1003158&&2597_4427_-1
Por_Secre_tail
Cas6
POI:
Beta-Casp
RMMBL
Lactamase_B_6
Cas7
Cas5
Pro_isomerase
cobW
Aldedh
NUDIX
HHH_3
GCA_011370805.1 | DTHG01000077
Cas5-HNH
Effector-Modules
161 / 243.7
(Cas5_HNH)
IMG_3300010327&&0116246_10008300&&2259_3435_-1
DUF6531
HEAT_2
Cas8
cse2
Cas7
POI:
Cas5
Cas6
Cas1
aGPT-Pplase1
cas5-HNH
GCA_019636195.1 | JAHBYA010000001
CasMu-V
Effector-Modules
2 / 4.0
(CasMuV MuB)
IMG_3300010349&&0116240_10096391&&801_1509_-1
rve
Mu-transpos_C
POI:
AAA_22
Cas12
muA
muB
IMG_3300010349 | 0116240_10020808
1kb

Cas12-Cas3
Effector-Modules
7 / 8.0

(Cas12 + Cas3)
IMG_3300020641&&Ga0206884_1077&&6347_8576_1

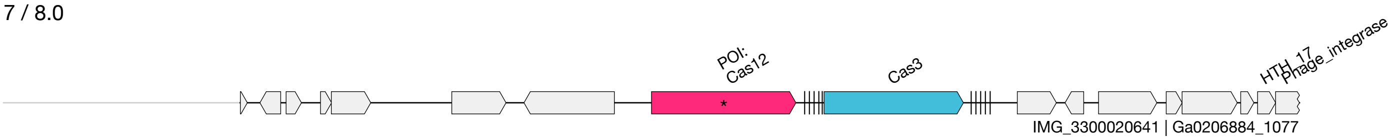

Cas12-IscB
Effector-Modules
4 / 9.5

(Cas12 + IscB + HTH)
IMG_3300027823&&0209490_10014851&&1048_2638_1

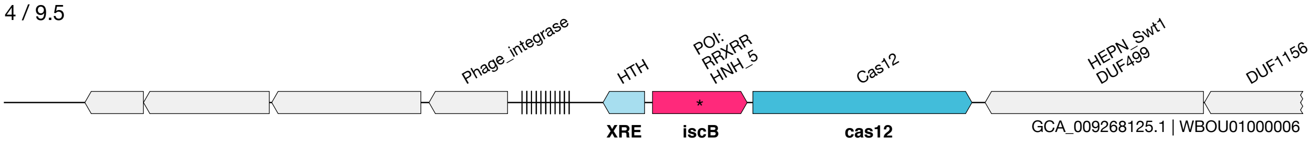

Cas8-HNH
Effector-Modules
5 / 8.0

(Cas8_HNH)
IMG_3300031760&&0326513_10063483&&752_1769_-1

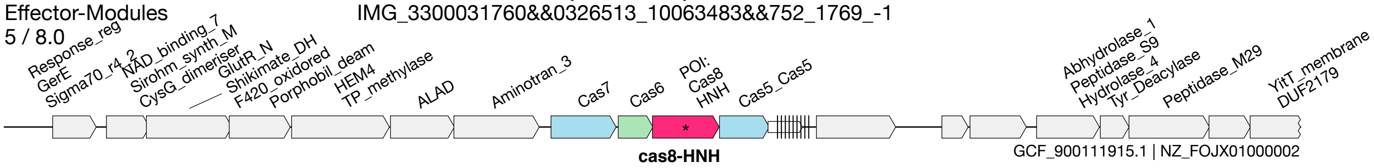

DinG-HNH
Effector-Modules
111 / 129.0

(DinG_HNH)
IMG_3300044617&&a0453743_0023453&&2386_4123_-1

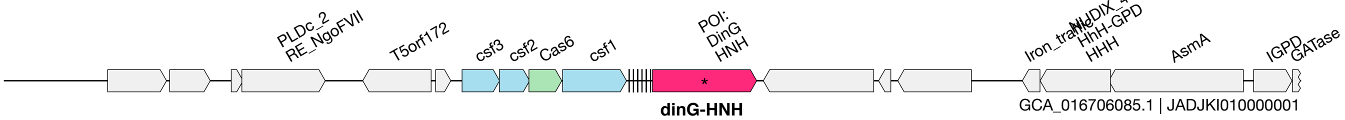

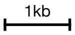

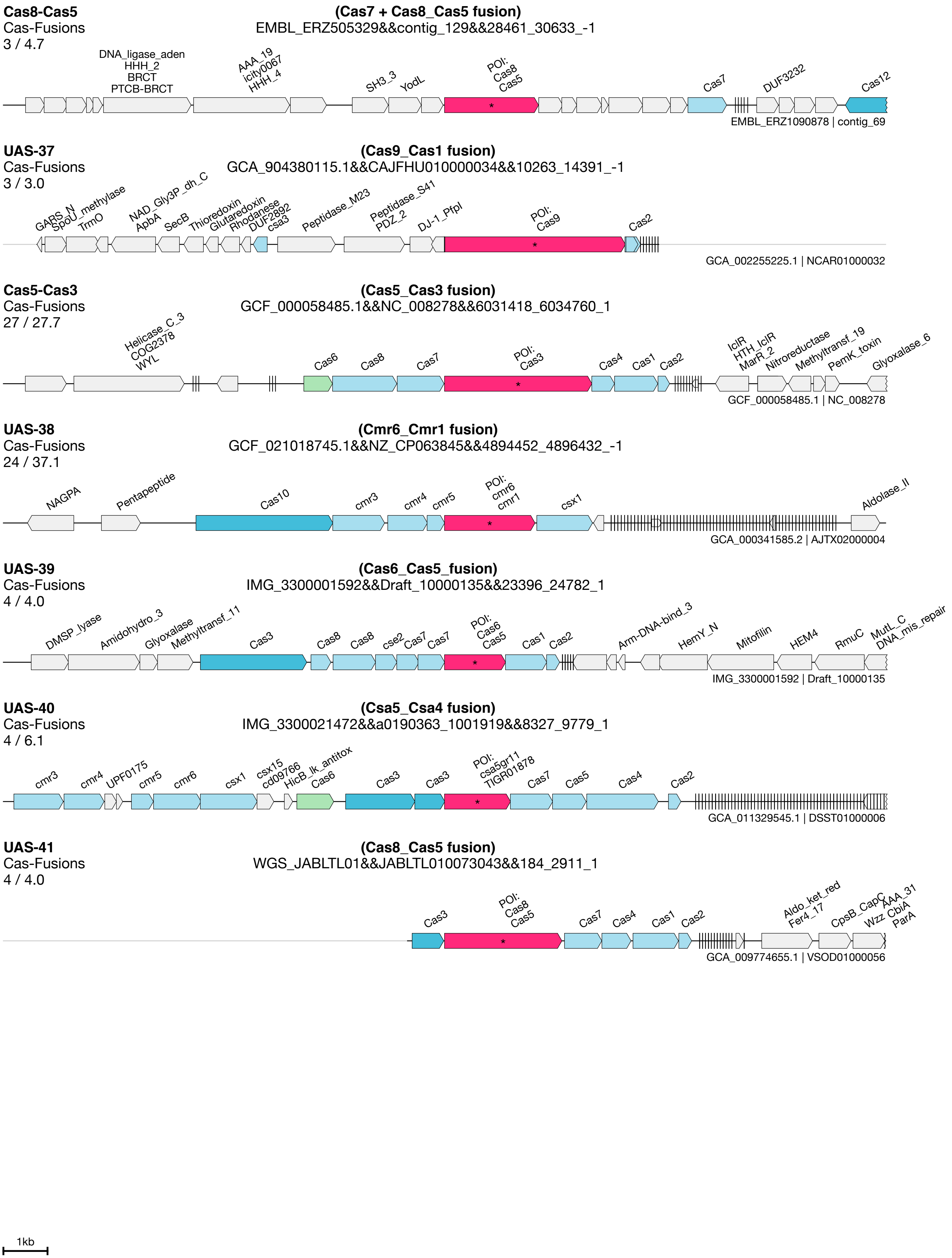

Cas8-Cas5
Cas-Fusions
3 / 4.7
(Cas7 + Cas8_Cas5 fusion)
EMBL_ERZ505329&&contig_129&&28461_30633_-1
DNA_ligase_aden
HHH_2
BRCT
PTCB-BRCT
AAA_19
icity0067
HHH_4
SH3_3
YodL
POI:
Cas8
Cas5
Cas7
DUF3232
Cas12
EMBL_ERZ1090878 | contig_69
UAS-37
Cas-Fusions
3 / 3.0
(Cas9_Cas1 fusion)
GCA_904380115.1&&CAJFHU010000034&&10263_14391_-1
GARS_N
SpoU_methylase
TrmO
NAD_Gly3P_dh_C
ApbA
SecB
Thioredoxin
Glutaredoxin
Rhodanese
DUF2892
csa3
Peptidase_M23
Peptidase_S41
PDZ_2
DJ-1_PfpI
POI:
Cas9
Cas2
GCA_002255225.1 | NCAR01000032
Cas5-Cas3
Cas-Fusions
27 / 27.7
(Cas5_Cas3 fusion)
GCF_000058485.1&&NC_008278&&6031418_6034760_1
Helicase_C_3
COG2378
WYL
Cas6
Cas8
Cas7
POI:
Cas3
Cas4
Cas1
Cas2
IclR
HTH_IclR
MarR_2
Nitroreductase
Methyltransf_19
PemK_toxin
Glyoxalase_6
GCF_000058485.1 | NC_008278
UAS-38
Cas-Fusions
24 / 37.1
(Cmr6_Cmr1 fusion)
GCF_021018745.1&&NZ_CP063845&&4894452_4896432_-1
NAGPA
Pentapeptide
Cas10
cmr3
cmr4
cmr5
POI:
cmr6
cmr1
csx1
Aldolase_II
GCA_000341585.2 | AJTX02000004
UAS-39
Cas-Fusions
4 / 4.0
(Cas6_Cas5_fusion)
IMG_3300001592&&Draft_10000135&&23396_24782_1
DMSP_lyase
Amidohydro_3
Glyoxalase
Methyltransf_11
Cas3
Cas8
Cas8
cse2
Cas7
Cas7
POI:
Cas6
Cas5
Cas1
Cas2
Arm-DNA-bind_3
HemY_N
Mitofilin
HEM4
RmuC
MutL_C
DNA_mis_repair
IMG_3300001592 | Draft_10000135
UAS-40
Cas-Fusions
4 / 6.1
(Csa5_Csa4 fusion)
IMG_3300021472&&a0190363_1001919&&8327_9779_1
cmr3
cmr4
UPF0175
cmr5
cmr6
csx1
csx15
cd09766
HicB_lk_antitox
Cas6
Cas3
Cas3
POI:
csa5gr11
TIGR01878
Cas7
Cas5
Cas4
Cas2
GCA_011329545.1 | DSST01000006
UAS-41
Cas-Fusions
4 / 4.0
(Cas8_Cas5 fusion)
WGS_JABLTL01&&JABLTL010073043&&184_2911_1
Cas3
POI:
Cas8
Cas5
Cas7
Cas4
Cas1
Cas2
Aldo_ket_red
Fer4_17
CpsB_CapC
AAA_31
Wzz
CbiA
ParA
GCA_009774655.1 | VSOD01000056
1kb

UAS-42
Acquisition
22 / 22.7

(DUF3761)
EMBL_ERZ724514&&contig_3192&&343_1033_-1

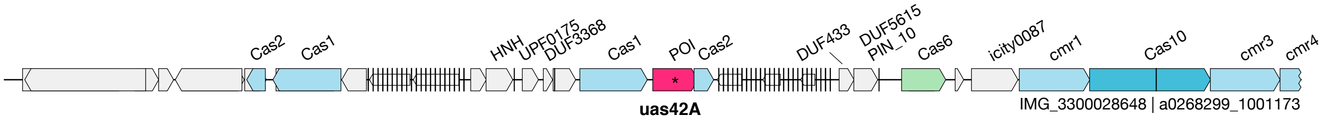

UAS-43
Acquisition
14 / 15.7

(RVT + group II intron PrimPol)
GCA_002358875.1&&DEPS01000038&&15824_19211_-1

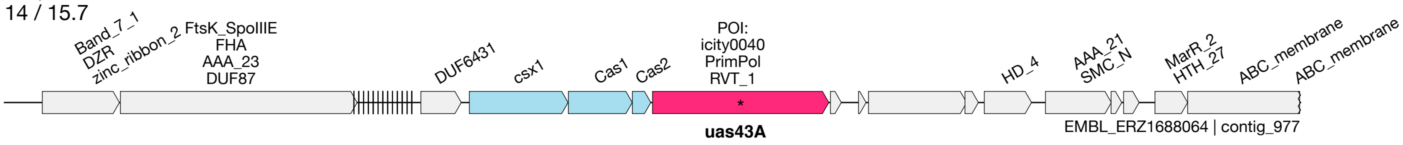

UAS-3
Acquisition
2 / 3.5

(DUF370)
GCA_013359445.1&&JABWBV010000281&&2844_3129_-1

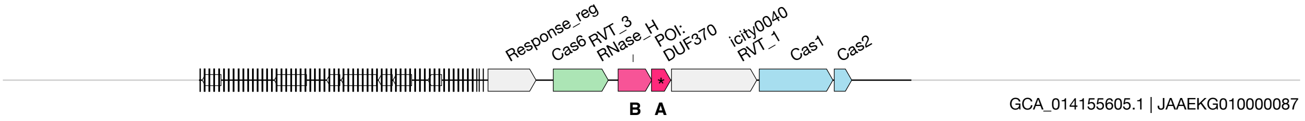

UAS-7
Acquisition
2 / 4.7

(vWA + RDD)
GCA_016179065.1&&JACOSD010000405&&5712_7455_-1

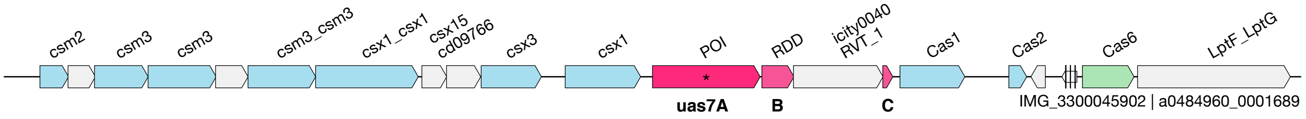

UAS-2
Acquisition
9 / 13.8

(Tbf2 WYL)
GCA_016223105.1&&JACRPR010000238&&1_1870_1

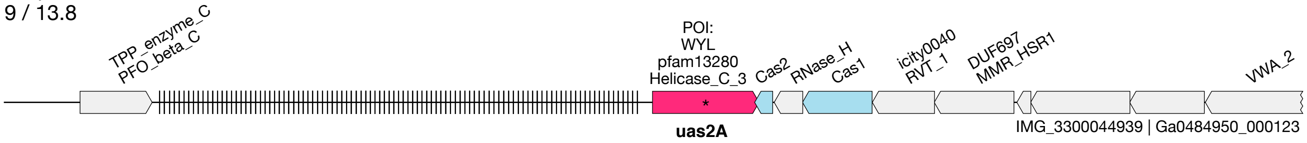

UAS-44
Acquisition
8 / 9.2

(DUF2797 large zinc finger protein)
GCA_016840085.1&&JAEOTA010000034&&18467_19766_-1

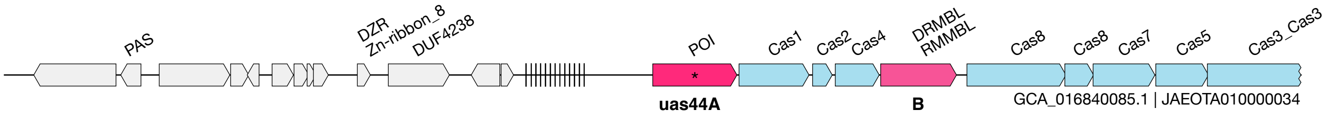

UAS-45
Acquisition
11 / 21.5

(RNaseH)
GCA_016876015.1&&VGOD01000184&&7922_8489_1

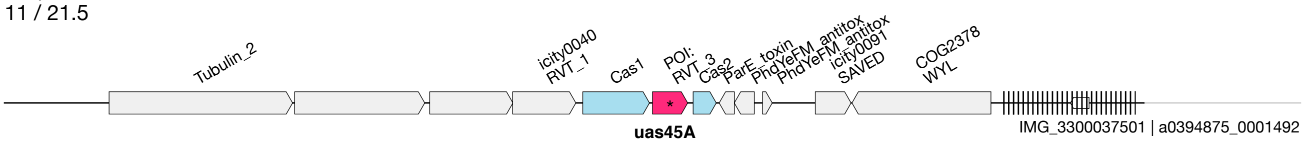

UAS-13
Acquisition
6 / 7.9

(DUF2797)
GCA_018238085.1&&JABCUA010000354&&227_1190_-1

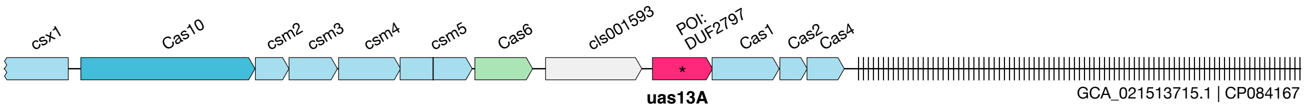

UAS-4
Acquisition
15 / 16.5

(DUF1882_primase)
GCA_018712075.1&&DVHN01000112&&0_1944_1

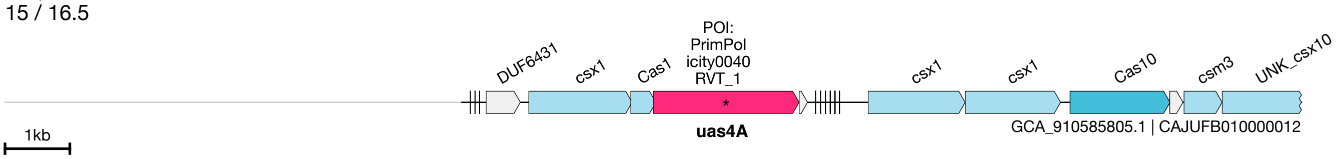

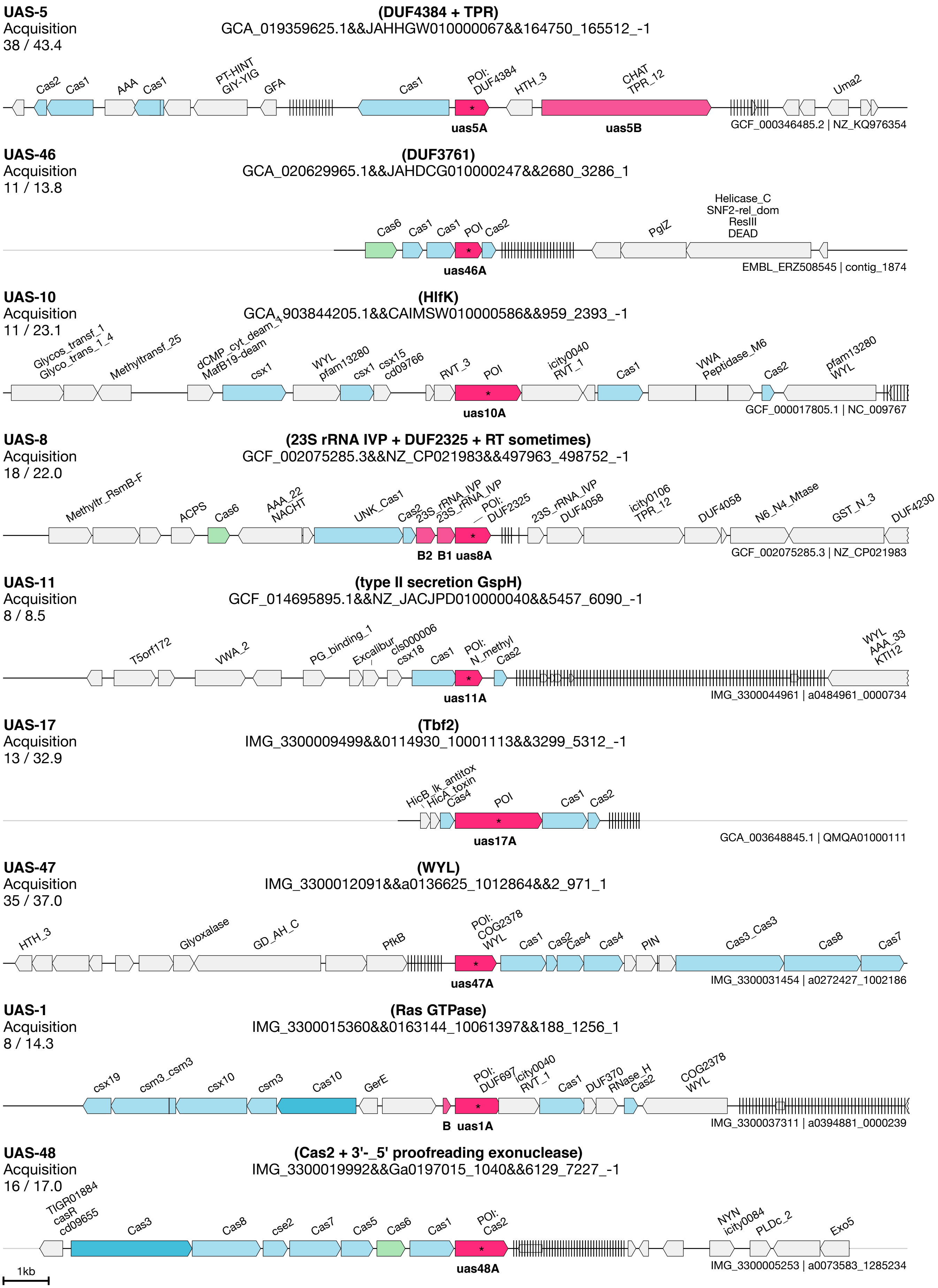

UAS-5
Acquisition
38 / 43.4
(DUF4384 + TPR)
GCA_019359625.1&&JAHHGW010000067&&164750_165512_-1
Cas2
Cas1
AAA
Cas1
PT-HINT
GIY-YIG
GFA
Cas1
POI:
DUF4384
HTH_3
CHAT
TPR_12
Uma2
uas5A
uas5B
GCF_000346485.2 | NZ_KQ976354
UAS-46
Acquisition
11 / 13.8
(DUF3761)
GCA_020629965.1&&JAHDCG010000247&&2680_3286_1
Cas6
Cas1
Cas1
POI
Cas2
PglZ
Helicase_C
SNF2-rel_dom
ResIII
DEAD
uas46A
EMBL_ERZ508545 | contig_1874
UAS-10
Acquisition
11 / 23.1
(HlfK)
GCA_903844205.1&&CAIMSW010000586&&959_2393_-1
Glycos_transf_1
Glyco_trans_1_4
Methyltransf_25
dCMP_cyt_deam_1
MafB19-deam
csx1
WYL
pfam13280
csx1
csx15
cd09766
RVT_3
POI
icity0040
RVT_1
Cas1
VWA
Peptidase_M6
Cas2
pfam13280
WYL
uas10A
GCF_000017805.1 | NC_009767
UAS-8
Acquisition
18 / 22.0
(23S rRNA IVP + DUF2325 + RT sometimes)
GCF_002075285.3&&NZ_CP021983&&497963_498752_-1
Methyltr_RsmB-F
ACPS
Cas6
AAA_22
NACHT
UNK_Cas1
Cas2
23S_rRNA_IVP
23S_rRNA_IVP
POI:
DUF2325
23S_rRNA_IVP
DUF4058
icity0106
TPR_12
DUF4058
N6_N4_Mtase
GST_N_3
DUF4230
B2 B1 uas8A
GCF_002075285.3 | NZ_CP021983
UAS-11
Acquisition
8 / 8.5
(type II secretion GspH)
GCF_014695895.1&&NZ_JACJPD010000040&&5457_6090_-1
T5orf172
VWA_2
PG_binding_1
Excalibur
cls000006
csx18
Cas1
POI:
N_methyl
Cas2
WYL
AAA_33
KTI12
uas11A
IMG_3300044961 | a0484961_0000734
UAS-17
Acquisition
13 / 32.9
(Tbf2)
IMG_3300009499&&0114930_10001113&&3299_5312_-1
HicB_lk_antitox
HicA_toxin
Cas4
POI
Cas1
Cas2
uas17A
GCA_003648845.1 | QMQA01000111
UAS-47
Acquisition
35 / 37.0
(WYL)
IMG_3300012091&&a0136625_1012864&&2_971_1
HTH_3
Glyoxalase
GD_AH_C
PfkB
POI:
COG2378
WYL
Cas1
Cas2
Cas4
Cas4
PIN
Cas3_Cas3
Cas8
Cas7
uas47A
IMG_3300031454 | a0272427_1002186
UAS-1
Acquisition
8 / 14.3
(Ras GTPase)
IMG_3300015360&&0163144_10061397&&188_1256_1
csx19
csm3_csm3
csx10
csm3
Cas10
GerE
POI:
DUF697
icity0040
RVT_1
Cas1
DUF370
RNase_H
Cas2
COG2378
WYL
B uas1A
IMG_3300037311 | a0394881_0000239
UAS-48
Acquisition
16 / 17.0
(Cas2 + 3'-_5' proofreading exonuclease)
IMG_3300019992&&Ga0197015_1040&&6129_7227_-1
TIGR01884
casR
cd09655
Cas3
Cas8
cse2
Cas7
Cas5
Cas6
Cas1
POI:
Cas2
NYN
icity0084
PLDc_2
Exo5
uas48A
IMG_3300005253 | a0073583_1285234
1kb

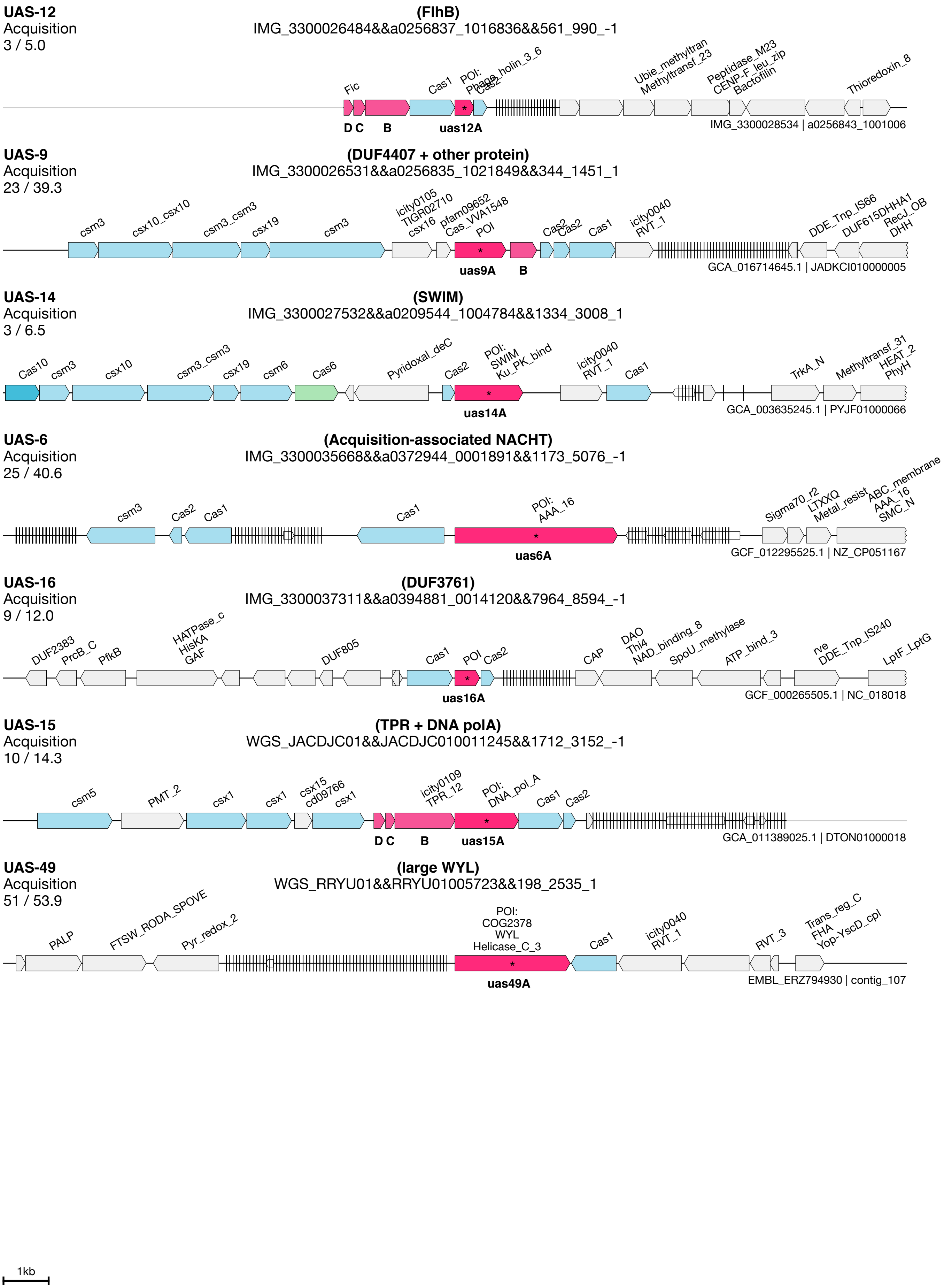
UAS-12
Acquisition
3 / 5.0
(FlhB)
IMG_3300026484&&a0256837_1016836&&561_990_-1
Fic
Cas1
POI:
Phage_holin_3_6
Cas2
Ubie_methyltran
Methyltransf_23
Peptidase_M23
CENP-F_leu_zip
Bactofilin
Thioredoxin_8
D C
B
uas12A
IMG_3300028534 | a0256843_1001006
UAS-9
Acquisition
23 / 39.3
(DUF4407 + other protein)
IMG_3300026531&&a0256835_1021849&&344_1451_1
csm3
csx10_csx10
csm3_csm3
csx19
csm3
icity0105
TIGR02710
csx16
pfam09652
Cas_VVA1548
POI
Cas2
Cas2
Cas1
icity0040
RVT_1
DDE_Tnp_IS66
DUF615DHHA1
RecJ_OB
DHH
uas9A
B
GCA_016714645.1 | JADKCI010000005
UAS-14
Acquisition
3 / 6.5
(SWIM)
IMG_3300027532&&a0209544_1004784&&1334_3008_1
Cas10
csm3
csx10
csm3_csm3
csx19
csm6
Cas6
Pyridoxal_deC
Cas2
POI:
SWIM
Ku_PK_bind
icity0040
RVT_1
Cas1
TrkA_N
Methyltransf_31
HEAT_2
PhyH
uas14A
GCA_003635245.1 | PYJF01000066
UAS-6
Acquisition
25 / 40.6
(Acquisition-associated NACHT)
IMG_3300035668&&a0372944_0001891&&1173_5076_-1
csm3
Cas2
Cas1
Cas1
POI:
AAA_16
Sigma70_r2
LTXXQ
Metal_resist
ABC_membrane
AAA_16
SMC_N
uas6A
GCF_012295525.1 | NZ_CP051167
UAS-16
Acquisition
9 / 12.0
(DUF3761)
IMG_3300037311&&a0394881_0014120&&7964_8594_-1
DUF2383
PrcB_C
PfkB
HATPase_c
HisKA
GAF
DUF805
Cas1
POI
Cas2
CAP
DAO
Thi4
NAD_binding_8
SpoU_methylase
ATP_bind_3
rve
DDE_Tnp_IS240
LptF_LptG
uas16A
GCF_000265505.1 | NC_018018
UAS-15
Acquisition
10 / 14.3
(TPR + DNA polA)
WGS_JACDJC01&&JACDJC010011245&&1712_3152_-1
csm5
PMT_2
csx1
csx1
csx15
cd09766
csx1
icity0109
TPR_12
POI:
DNA_pol_A
Cas1
Cas2
D C
B
uas15A
GCA_011389025.1 | DTON01000018
UAS-49
Acquisition
51 / 53.9
(large WYL)
WGS_RRYU01&&RRYU01005723&&198_2535_1
PALP
FTSW_RODA_SPOVE
Pyr_redox_2
POI:
COG2378
WYL
Helicase_C_3
Cas1
icity0040
RVT_1
RVT_3
Trans_reg_C
FHA
Yop-YscD_cpl
uas49A
EMBL_ERZ794930 | contig_107
1kb

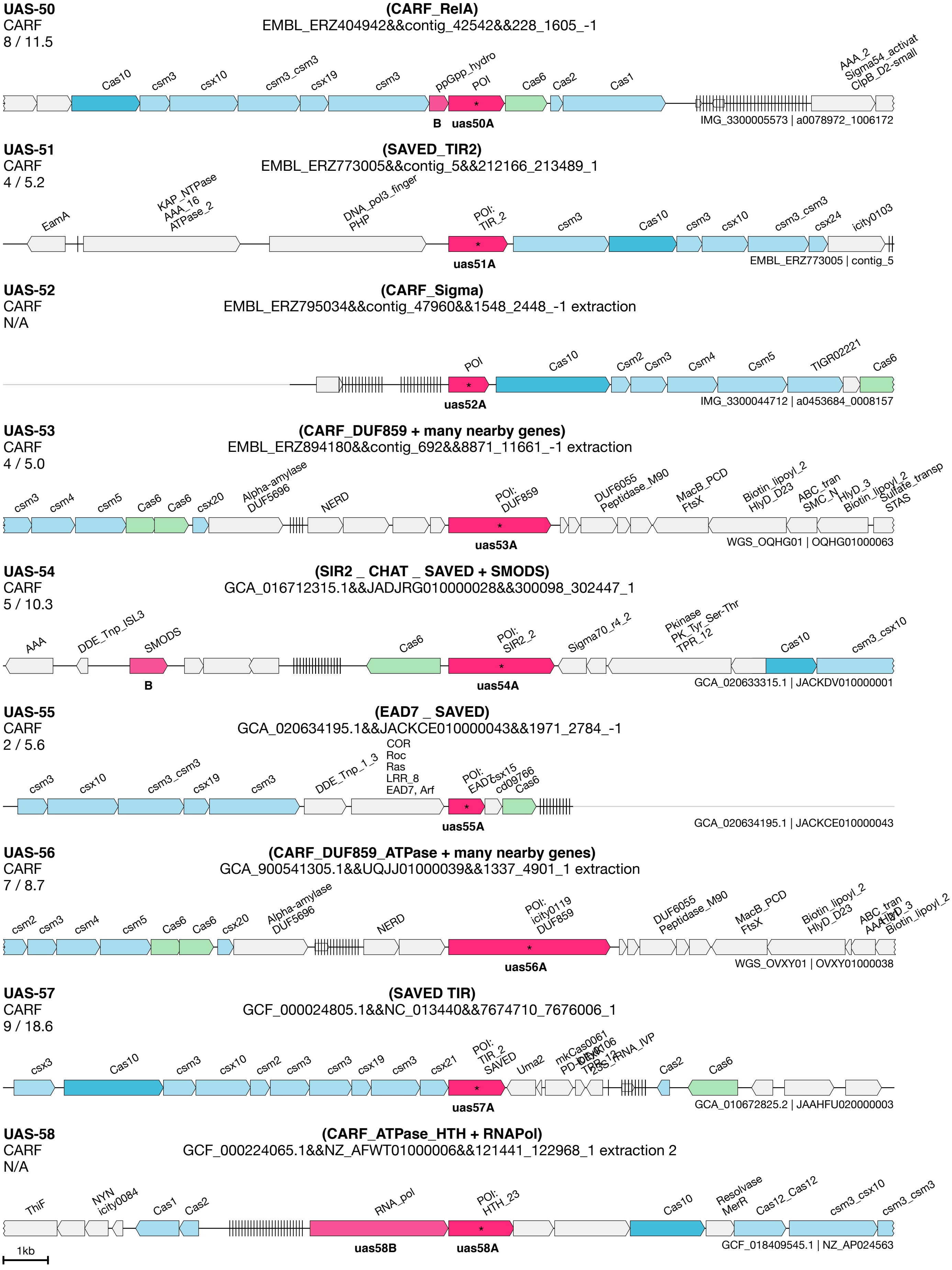
UAS-50
CARF
8 / 11.5
(CARF_RelA)
EMBL_ERZ404942&&contig_42542&&228_1605_-1
Cas10 csm3 csx10 csm3_csm3 csx19 csm3 ppGpp_hydro POI Cas6 Cas2 Cas1
AAA_2 Sigma54_activat ClpB_D2-small
B uas50A
IMG_3300005573 | a0078972_1006172
UAS-51
CARF
4 / 5.2
(SAVED_TIR2)
EMBL_ERZ773005&&contig_5&&212166_213489_1
EamA KAP_NTPase AAA_16 ATPase_2 DNA_pol3_finger PHP POI: TIR_2 csm3 Cas10 csm3 csx10 csm3_csm3 csx24 icity0103
uas51A
EMBL_ERZ773005 | contig_5
UAS-52
CARF
N/A
(CARF_Sigma)
EMBL_ERZ795034&&contig_47960&&1548_2448_-1 extraction
POI Cas10 Csm2 Csm3 Csm4 Csm5 TIGR02221 Cas6
uas52A
IMG_3300044712 | a0453684_0008157
UAS-53
CARF
4 / 5.0
(CARF_DUF859 + many nearby genes)
EMBL_ERZ894180&&contig_692&&8871_11661_-1 extraction
csm3 csm4 csm5 Cas6 Cas6 csx20 Alpha-amylase DUF5696 NERD POI: DUF859 DUF6055 Peptidase_M90 MacB_PCD FtsX Biotin_lipoyl_2 HlyD_D23 ABC_tran SMC_N HlyD_3 Biotin_lipoyl_2 Sulfate_transp STAS
uas53A
WGS_OQHG01 | OQHG01000063
UAS-54
CARF
5 / 10.3
(SIR2 _ CHAT _ SAVED + SMODS)
GCA_016712315.1&&JADJRG010000028&&300098_302447_1
AAA DDE_Tnp_ISL3 SMODS Cas6 POI: SIR2_2 Sigma70_r4_2 Pkinase PK_Tyr_Ser-Thr TPR_12 Cas10 csm3_csx10
B uas54A
GCA_020633315.1 | JACKDV010000001
UAS-55
CARF
2 / 5.6
(EAD7 _ SAVED)
GCA_020634195.1&&JACKCE010000043&&1971_2784_-1
csm3 csx10 csm3_csm3 csx19 csm3 DDE_Tnp_1_3 COR Roc Ras LRR_8 EAD7, Arf POI: EAD7 csx15 cd09766 Cas6
uas55A
GCA_020634195.1 | JACKCE010000043
UAS-56
CARF
7 / 8.7
(CARF_DUF859_ATPase + many nearby genes)
GCA_900541305.1&&UQJJ01000039&&1337_4901_1 extraction
csm2 csm3 csm4 csm5 Cas6 Cas6 csx20 Alpha-amylase DUF5696 NERD POI: icity0119 DUF859 DUF6055 Peptidase_M90 MacB_PCD FtsX Biotin_lipoyl_2 HlyD_D23 ABC_tran HlyD_3 Biotin_lipoyl_2
uas56A
WGS_OVXY01 | OVXY01000038
UAS-57
CARF
9 / 18.6
(SAVED TIR)
GCF_000024805.1&&NC_013440&&7674710_7676006_1
csx3 Cas10 csm3 csx10 csm2 csm3 csm3 csx19 csm3 csx21 POI: TIR_2 SAVED Uma2 mkCas0061 PD-DExK TPR_12 23S_rRNA_IVP Cas2 Cas6
uas57A
GCA_010672825.2 | JAAHFU020000003
UAS-58
CARF
N/A
(CARF_ATPase_HTH + RNAPol)
GCF_000224065.1&&NZ_AFWT01000006&&121441_122968_1 extraction 2
ThiF NYN icity0084 Cas1 Cas2 RNA_pol POI: HTH_23 Cas10 Resolvase MerR Cas12_Cas12 csm3_csx10 csm3_csm3
uas58B uas58A
GCF_018409545.1 | NZ_AP024563
1kb

UAS-59
CARF
3 / 5.1

(CARF_CYTH_HD)
IMG_3300002498&&TOLCLC_10008146&&3727_6961_-1 extraction

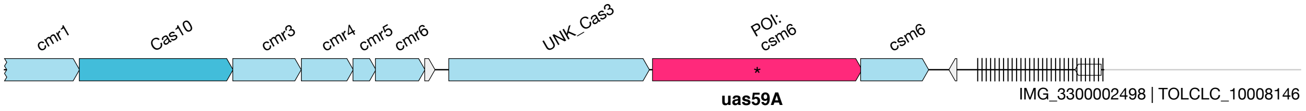

UAS-60
CARF
N/A

(CARF_SIR2)
IMG_3300009523&&a0116221_1002542&&6420_8025_-1_loci_to_order

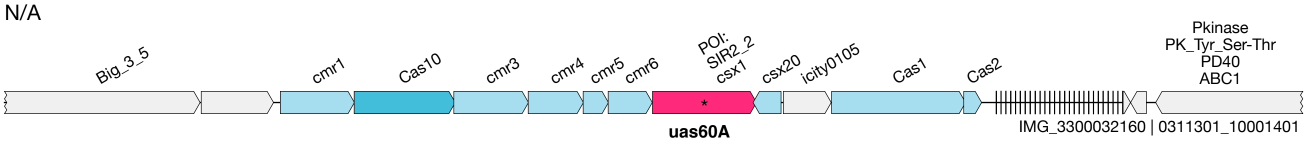

UAS-61
CARF
11 / 15.3

(CARF_TIR_Mrr)
IMG_3300020198&&0194120_10015339&&5702_6689_1 extraction

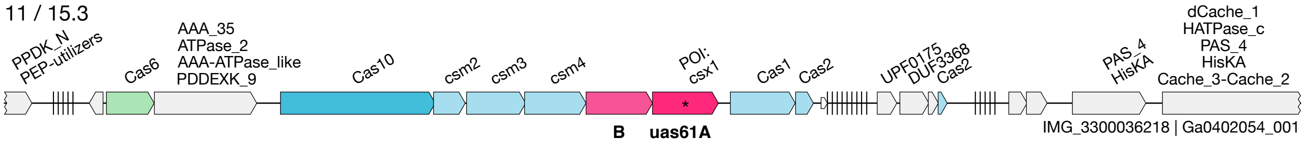

UAS-62
CARF
5 / 12.2

(csx1 + Phage RNAPol)
IMG_3300026484&&a0256837_1008848&&1106_4061_-1

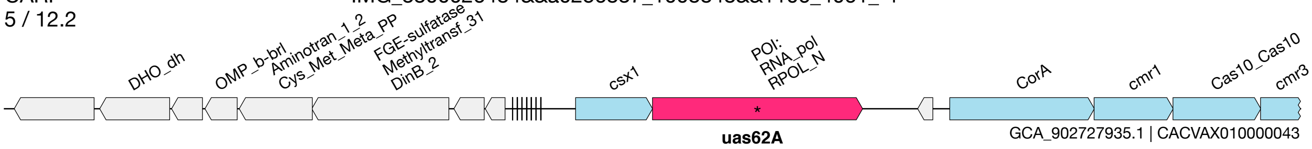

UAS-63
CARF
5 / 7.7

(CARF_2p_deoxyribosyltransferase)
IMG_3300031539&&0307380_10010365&&4440_5463_-1

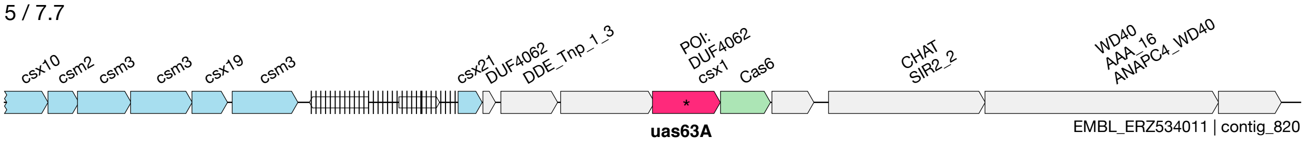

UAS-64
CARF
N/A

(CARF_EAD7)
IMG_3300031566&&0307378_10017289&&164_1499_1 extraction

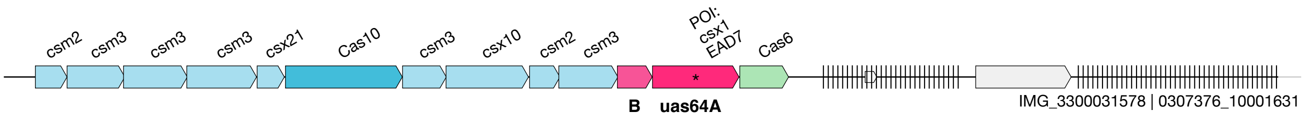

UAS-65
CARF
6 / 6.6

(CARF_5p_nucleotidase)
IMG_3300031994&&0310691_10021322&&2165_3863_1 extraction

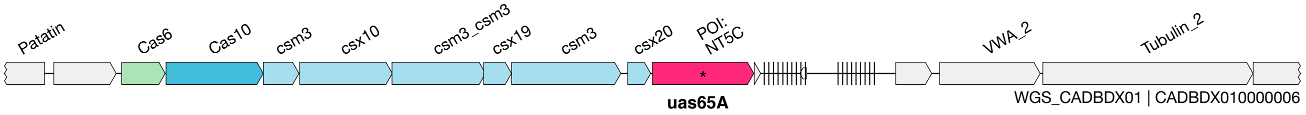

UAS-66
CARF
4 / 4.5

(SAVED_APH_TCAD9)
IMG_3300034111&&a0335063_0013299&&1300_3253_1

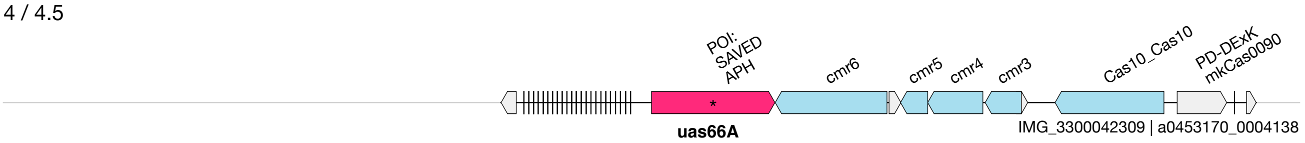

UAS-67
CARF
4 / 4.7

(Cutinase_Lipase_SAVED)
IMG_3300042939&&a0453142_0007685&&8027_9725_-1

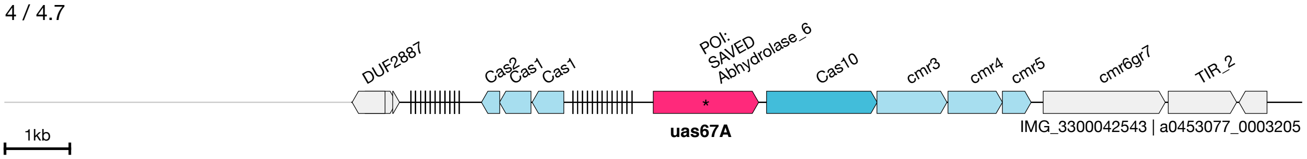

UAS-68
CARF
N/A

(SMODS + SAVED CHAT)
IMG_3300046790&&a0495499_0000077&&7424_8333_1

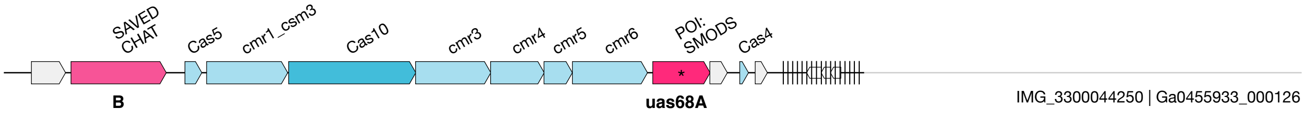

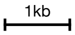

UAS-69
Auxiliary
3 / 7.0

(methyltransferase)
EMBL_ERZ1030305&&contig_263&&8899_9757_1

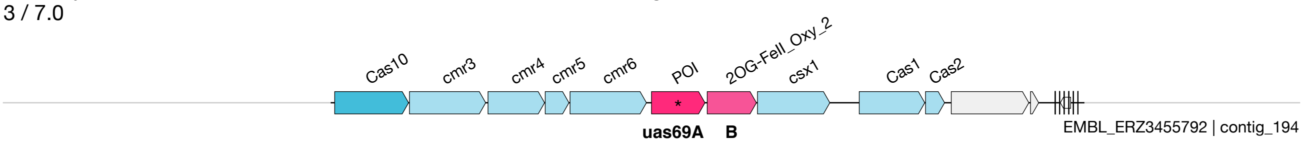

UAS-70
Auxiliary
9 / 9.3

(vATP-synt_E)
EMBL_ERZ1699236&&contig_1829&&258_2607_-1

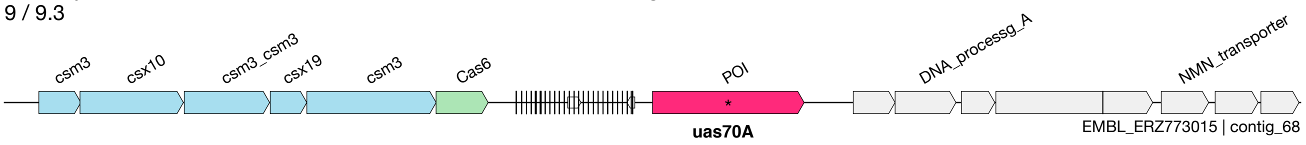

UAS-71
Auxiliary
4 / 4.7

(NERD)
EMBL_ERZ1764562&&contig_378&&16539_17331_1

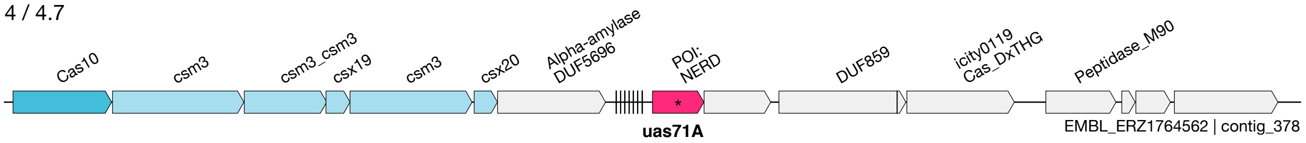

UAS-72
Auxiliary
20 / 26.7

(multi-gene system including greA greB)
EMBL_ERZ3455816&&contig_9406&&2751_3312_1

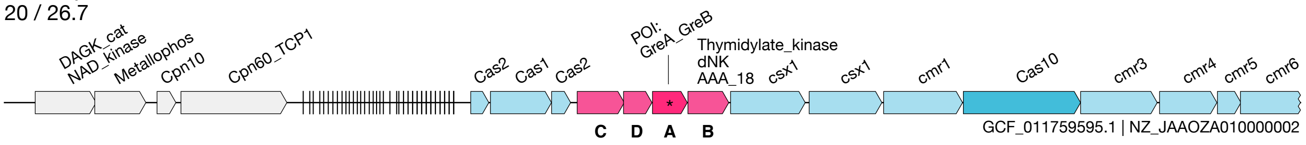

UAS-73
Auxiliary
7 / 10.5

(Mrr nuclease)
EMBL_ERZ405284&&contig_1793&&4535_5441_1

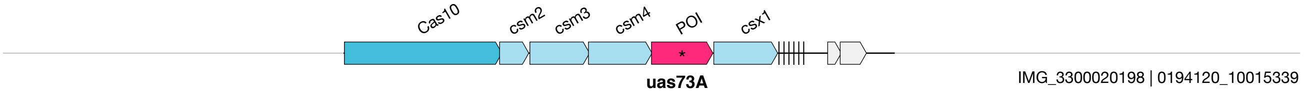

UAS-74
Auxiliary
2 / 3.4

(PKinase)
EMBL_ERZ4560480&&contig_4482&&7569_10323_-1

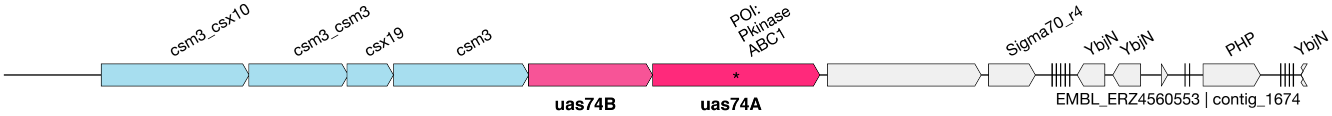

UAS-75
Auxiliary
7 / 7.5

(Sigma_factor + Csx1 + large Csx1)
EMBL_ERZ4560494&&contig_2628&&7202_8435_1

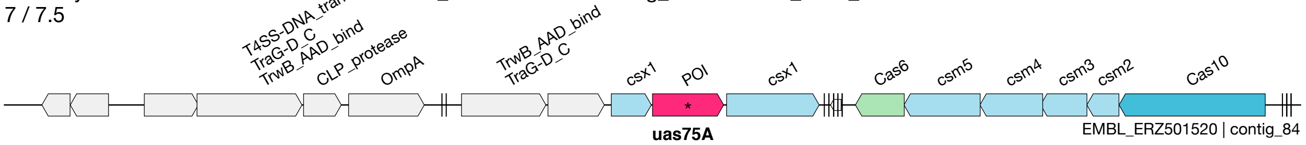

UAS-76
Auxiliary
41 / 53.8

(HTH _ M78 peptidase)
EMBL_ERZ4560656&&contig_7640&&76_1108_-1

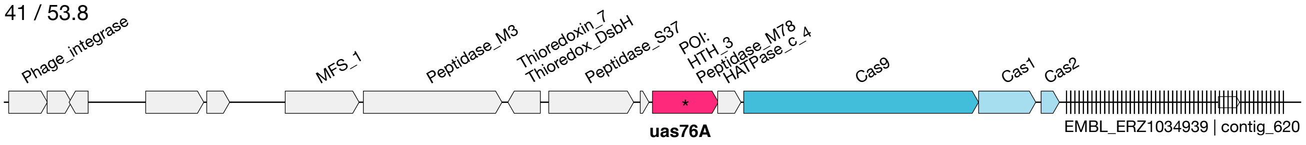

UAS-77
Auxiliary
3 / 5.5

(DUF2283)
EMBL_ERZ584659&&contig_1773&&510_1521_-1

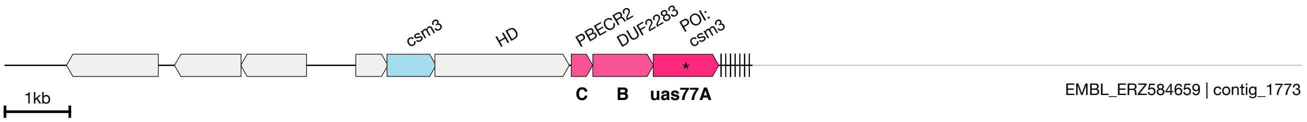

UAS-78
Auxiliary
7 / 7.7

(Nif11)
EMBL_ERZ650341&&contig_14933&&186_501_1

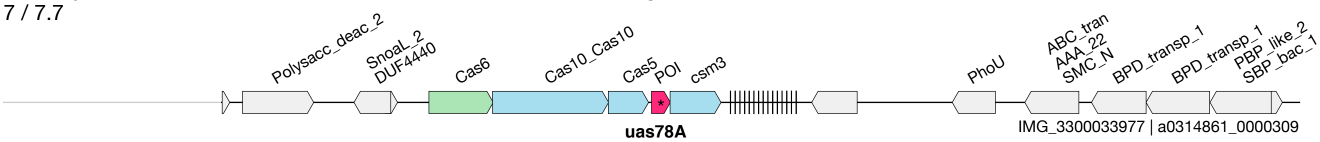

UAS-79
Auxiliary
8 / 8.9

(XRE HTH regulator operonized with Cas effectors)
EMBL_ERZ653703&&contig_1622&&2425_3673_-1

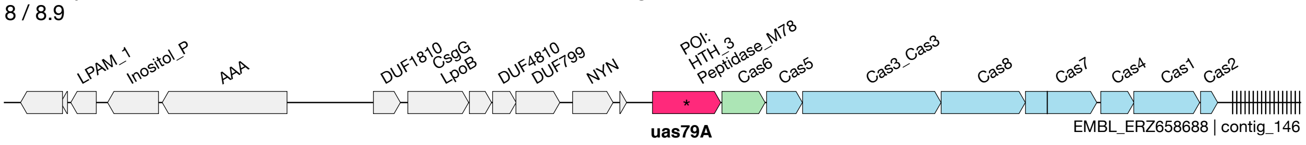

UAS-80
Auxiliary
5 / 5.1

(RepA replication)
EMBL_ERZ724078&&contig_5708&&5799_7854_-1

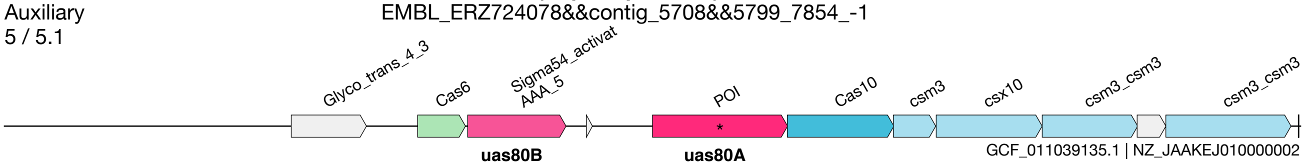

UAS-81
Auxiliary
3 / 3.0

(DUF4145)
EMBL_ERZ724507&&contig_6357&&940_1633_-1

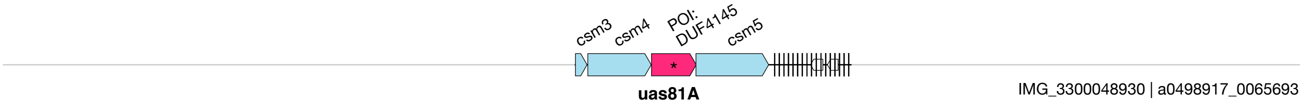

UAS-82
Auxiliary
4 / 7.6

(pentapeptide + unknown domain)
EMBL_ERZ772753&&contig_7251&&225_1815_-1

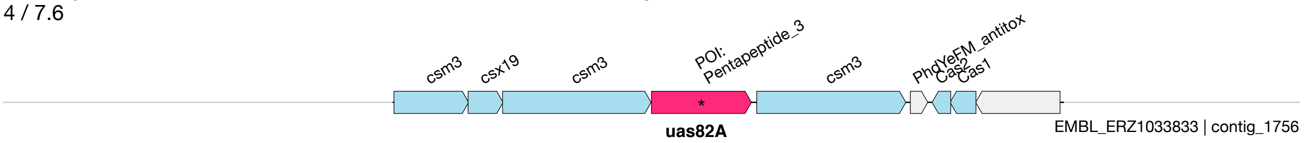

UAS-83
Auxiliary
8 / 8.0

(Recep domain + 4 unknown genes)
EMBL_ERZ773219&&contig_530&&18381_19626_1

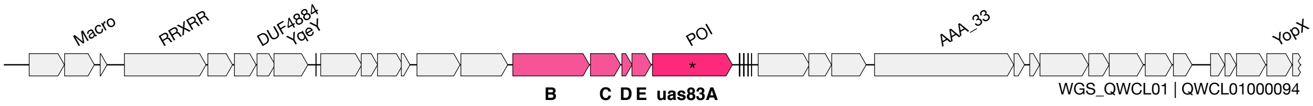

UAS-84
Auxiliary
2 / 5.3

(MazF)
EMBL_ERZ773360&&contig_3930&&5259_6435_-1

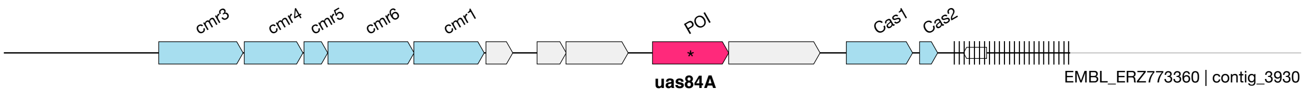

UAS-31
Auxiliary
10 / 16.4

(Kinase)
EMBL_ERZ778503&&contig_16504&&109_2353_-1

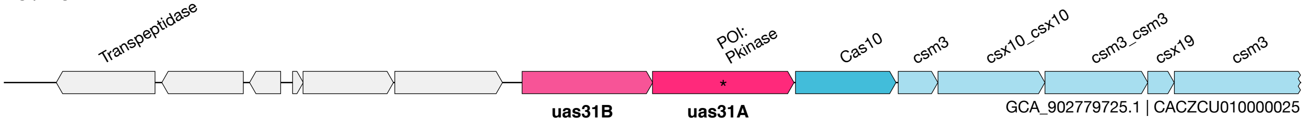

UAS-85
Auxiliary
2 / 5.5

(betalactamase)
EMBL_ERZ778607&&contig_14514&&540_2586_-1

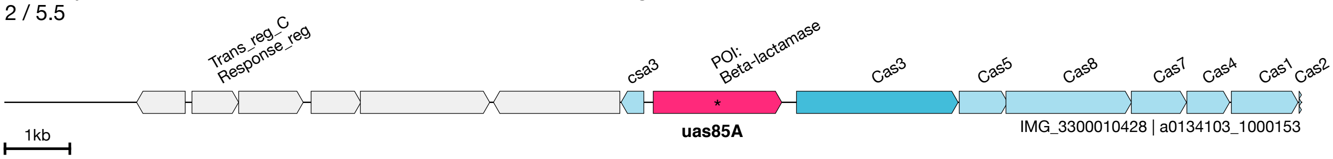

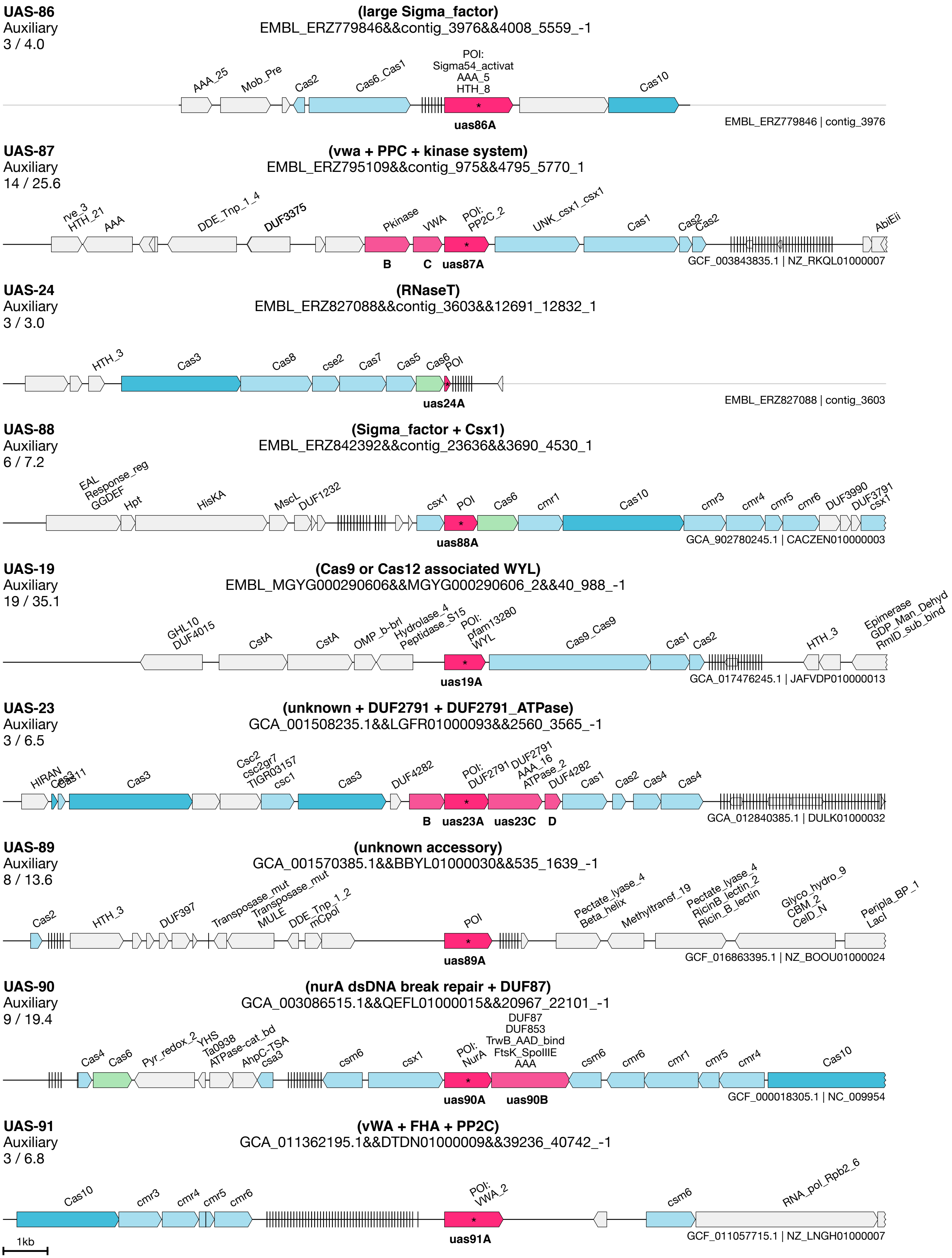
UAS-86
Auxiliary
3 / 4.0
(large Sigma_factor)
EMBL_ERZ779846&&contig_3976&&4008_5559_-1
AAA_25
Mob_Pre
Cas2
Cas6_Cas1
POI:
Sigma54_activat
AAA_5
HTH_8
Cas10
uas86A
EMBL_ERZ779846 | contig_3976
UAS-87
Auxiliary
14 / 25.6
(vwa + PPC + kinase system)
EMBL_ERZ795109&&contig_975&&4795_5770_1
rve_3
HTH_21
AAA
DDE_Tnp_1_4
DUF3375
Pkinase
VWA
POI:
PP2C_2
UNK_csx1_csx1
Cas1
Cas2
Cas2
AbiEii
B
C
uas87A
GCF_003843835.1 | NZ_RKQL01000007
UAS-24
Auxiliary
3 / 3.0
(RNaseT)
EMBL_ERZ827088&&contig_3603&&12691_12832_1
HTH_3
Cas3
Cas8
cse2
Cas7
Cas5
Cas6
POI
uas24A
EMBL_ERZ827088 | contig_3603
UAS-88
Auxiliary
6 / 7.2
(Sigma_factor + Csx1)
EMBL_ERZ842392&&contig_23636&&3690_4530_1
EAL
Response_reg
GGDEF
Hpt
HisKA
MscL
DUF1232
csx1
POI
Cas6
cmr1
Cas10
cmr3
cmr4
cmr5
cmr6
DUF3990
DUF3791
csx1
uas88A
GCA_902780245.1 | CACZEN010000003
UAS-19
Auxiliary
19 / 35.1
(Cas9 or Cas12 associated WYL)
EMBL_MGYG000290606&&MGYG000290606_2&&40_988_-1
GHL10
DUF4015
CstA
CstA
OMP_b-brl
Hydrolase_4
Peptidase_S15
POI:
pfam13280
WYL
Cas9_Cas9
Cas1
Cas2
HTH_3
Epimerase
GDP_Man_Dehyd
RmlD_sub_bind
uas19A
GCA_017476245.1 | JAFVDP010000013
UAS-23
Auxiliary
3 / 6.5
(unknown + DUF2791 + DUF2791_ATPase)
GCA_001508235.1&&LGFR01000093&&2560_3565_-1
HIRAN
Cas3
Cas11
Cas3
Csc2
csc2gr7
TIGR03157
csc1
Cas3
DUF4282
POI:
DUF2791
DUF2791
AAA_16
ATPase_2
DUF4282
Cas1
Cas2
Cas4
Cas4
B
uas23A
uas23C
D
GCA_012840385.1 | DULK01000032
UAS-89
Auxiliary
8 / 13.6
(unknown accessory)
GCA_001570385.1&&BBYL01000030&&535_1639_-1
Cas2
HTH_3
DUF397
Transposase_mut
Transposase_mut
MULE
DDE_Tnp_1_2
mCpol
POI
Pectate_lyase_4
Beta_helix
Methyltransf_19
Pectate_lyase_4
RicinB_lectin_2
Ricin_B_lectin
Glyco_hydro_9
CBM_2
CelD_N
Peripla_BP_1
LacI
uas89A
GCF_016863395.1 | NZ_BOOU01000024
UAS-90
Auxiliary
9 / 19.4
(nurA dsDNA break repair + DUF87)
GCA_003086515.1&&QEFL01000015&&20967_22101_-1
Cas4
Cas6
Pyr_redox_2
YHS
Ta0938
ATPase-cat_bd
AhpC-TSA
csa3
csm6
csx1
POI:
NurA
DUF87
DUF853
TrwB_AAD_bind
FtsK_SpoIIIE
AAA
csm6
cmr6
cmr1
cmr5
cmr4
Cas10
uas90A
uas90B
GCF_000018305.1 | NC_009954
UAS-91
Auxiliary
3 / 6.8
(vWA + FHA + PP2C)
GCA_011362195.1&&DTDN01000009&&39236_40742_-1
Cas10
cmr3
cmr4
cmr5
cmr6
POI:
VWA_2
csm6
RNA_pol_Rpb2_6
uas91A
GCF_011057715.1 | NZ_LNGH01000007
1kb

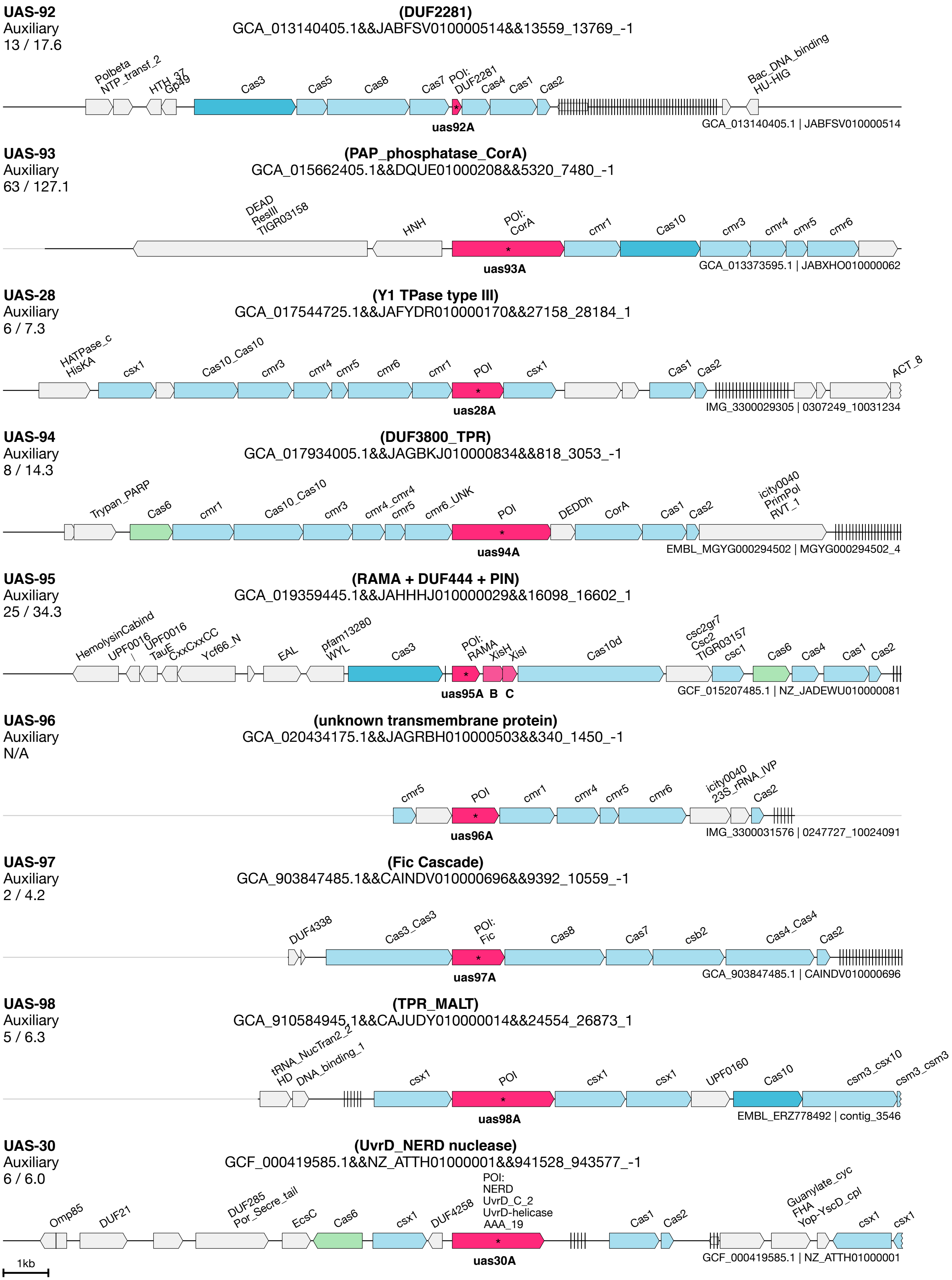
UAS-92
Auxiliary
13 / 17.6
(DUF2281)
GCA_013140405.1&&JABFSV010000514&&13559_13769_-1
Polbeta
NTP_transf_2
HTH_37
Gp49
Cas3
Cas5
Cas8
Cas7
POI:
DUF2281
Cas4
Cas1
Cas2
Bac_DNA_binding
HU-HIG
uas92A
GCA_013140405.1 | JABFSV010000514
UAS-93
Auxiliary
63 / 127.1
(PAP_phosphatase_CorA)
GCA_015662405.1&&DQUE01000208&&5320_7480_-1
DEAD
ResIII
TIGR03158
HNH
POI:
CorA
cmr1
Cas10
cmr3
cmr4
cmr5
cmr6
uas93A
GCA_013373595.1 | JABXHO010000062
UAS-28
Auxiliary
6 / 7.3
(Y1 TPase type III)
GCA_017544725.1&&JAFYDR010000170&&27158_28184_1
HATPase_c
HisKA
csx1
Cas10_Cas10
cmr3
cmr4
cmr5
cmr6
cmr1
POI
csx1
Cas1
Cas2
ACT_8
uas28A
IMG_3300029305 | 0307249_10031234
UAS-94
Auxiliary
8 / 14.3
(DUF3800_TPR)
GCA_017934005.1&&JAGBKJ010000834&&818_3053_-1
Trypan_PARP
Cas6
cmr1
Cas10_Cas10
cmr3
cmr4_cmr4
cmr5
cmr6_UNK
POI
DEDDh
CorA
Cas1
Cas2
icity0040
PrimPol
RVT_1
uas94A
EMBL_MGYG000294502 | MGYG000294502_4
UAS-95
Auxiliary
25 / 34.3
(RAMA + DUF444 + PIN)
GCA_019359445.1&&JAHHHJ010000029&&16098_16602_1
HemolysinCabind
UPF0016
UPF0016
TauE
CxxCxxCC
Ycf66_N
EAL
pfam13280
WYL
Cas3
POI:
RAMA
XisH
XisI
Cas10d
csc2gr7
Csc2
TIGR03157
csc1
Cas6
Cas4
Cas1
Cas2
uas95A B C
GCF_015207485.1 | NZ_JADEWU010000081
UAS-96
Auxiliary
N/A
(unknown transmembrane protein)
GCA_020434175.1&&JAGRBH010000503&&340_1450_-1
cmr5
POI
cmr1
cmr4
cmr5
cmr6
icity0040
23S_rRNA_IVP
Cas2
uas96A
IMG_3300031576 | 0247727_10024091
UAS-97
Auxiliary
2 / 4.2
(Fic Cascade)
GCA_903847485.1&&CAINDV010000696&&9392_10559_-1
DUF4338
Cas3_Cas3
POI:
Fic
Cas8
Cas7
csb2
Cas4_Cas4
Cas2
uas97A
GCA_903847485.1 | CAINDV010000696
UAS-98
Auxiliary
5 / 6.3
(TPR_MALT)
GCA_910584945.1&&CAJUDY010000014&&24554_26873_1
tRNA_NucTran2_2
HD
DNA_binding_1
csx1
POI
csx1
csx1
UPF0160
Cas10
csm3_csx10
csm3_csm3
uas98A
EMBL_ERZ778492 | contig_3546
UAS-30
Auxiliary
6 / 6.0
(UvrD_NERD nuclease)
GCF_000419585.1&&NZ_ATTH01000001&&941528_943577_-1
Omp85
DUF21
DUF285
Por_Secre_tail
EcsC
Cas6
csx1
DUF4258
POI:
NERD
UvrD_C_2
UvrD-helicase
AAA_19
Cas1
Cas2
Guanylate_cyc
FHA
Yop-YscD_cpl
csx1
csx1
uas30A
GCF_000419585.1 | NZ_ATTH01000001
1kb

UAS-99
Auxiliary
20 / 37.7

(Radical SAM protein)
GCF_001029445.1&&NZ_LDOT01000053&&7692_9432_1

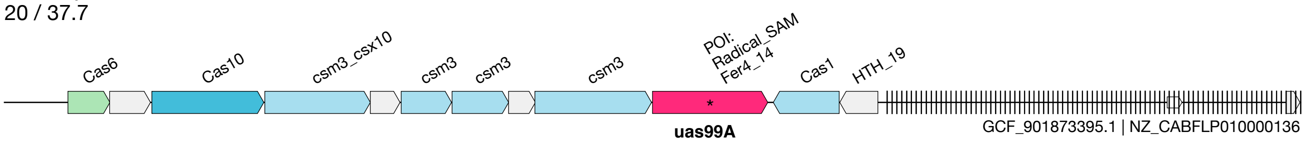

UAS-100
Auxiliary
6 / 9.9

(wWA + kinase)
GCF_001655635.1&&NZ_LSNH01000001&&2031044_2032025_-1

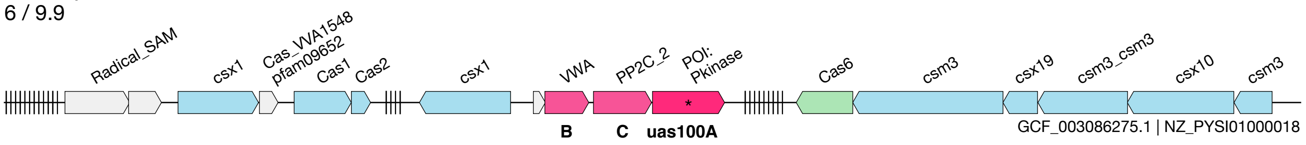

UAS-101
Auxiliary
19 / 47.0

(DUF2204)
GCF_003668875.1&&NZ_RAWM01000178&&1907_2396_-1

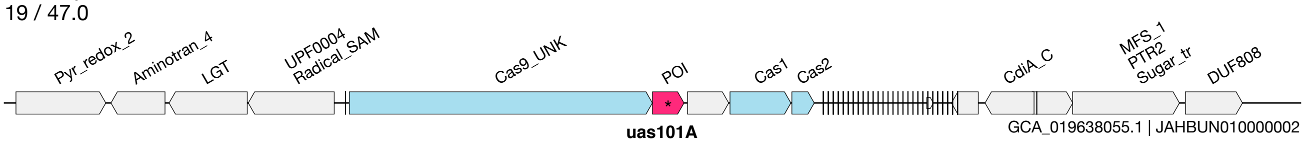

UAS-102
Auxiliary
3 / 3.0

(DUF5343 + Helicase_HNH_ResIII)
GCF_016526145.1&&NZ_JADDUE010000010&&220137_220869_-1

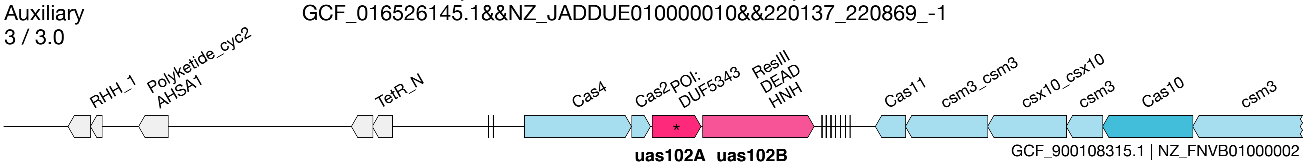

UAS-103
Auxiliary
2 / 3.0

(TPR HATPase)
GCF_016757215.1&&NZ_JAESIY010000005&&313654_315544_1

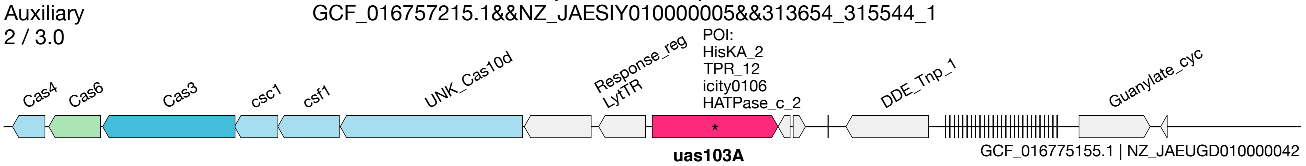

UAS-104
Auxiliary
3 / 3.0

(ATPase + Metallophosphatase)
GCF_018502485.1&&NZ_CP075546&&993250_997099_1

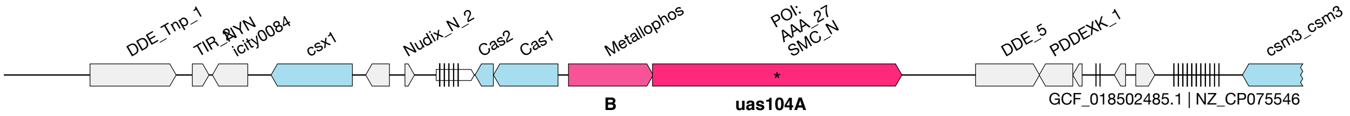

UAS-105
Auxiliary
5 / 5.6

(M78 peptidase)
GCF_021531665.1&&NZ_JADYUH010000077&&9506_10499_-1

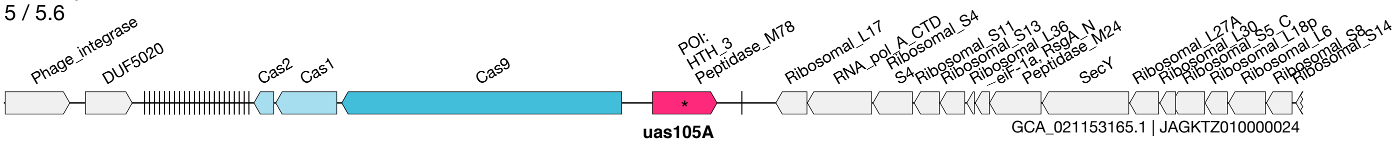

UAS-106
Auxiliary
3 / 3.0

(DUF3800)
GCF_900453405.1&&NZ_UGPM01000003&&1785257_1786514_-1

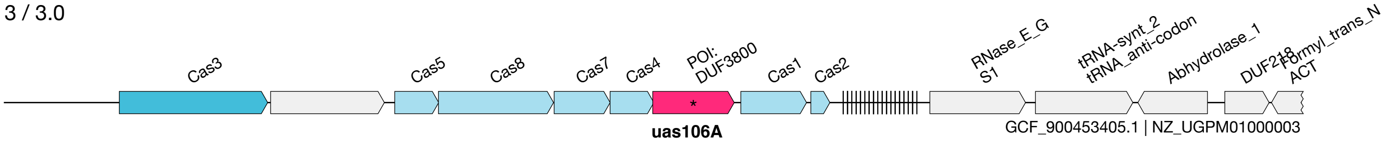

UAS-107
Auxiliary
17 / 25.5

(Toprim)
GCF_902500215.1&&NZ_CABVOU010000021&&55311_56466_-1

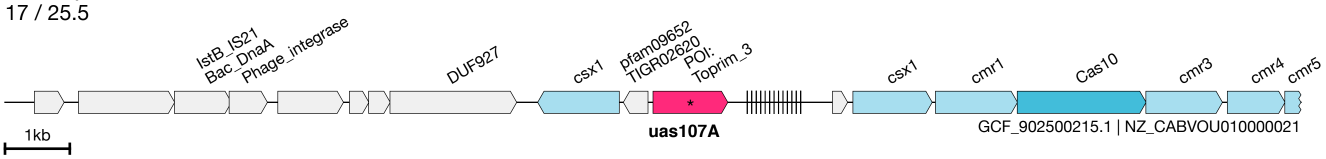

UAS-108
Auxiliary
7 / 16.3

(DUF433)
IMG_3300000574&&7J11328_10007417&&1272_1539_-1

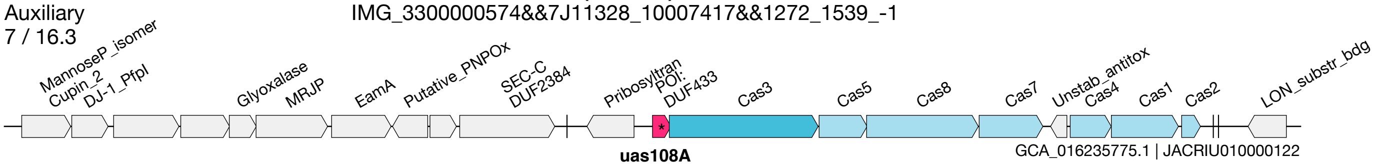

UAS-109
Auxiliary
2 / 5.3

(DUF4407 + transpeptidase)
IMG_3300000574&&7J11328_10033202&&130_1651_1

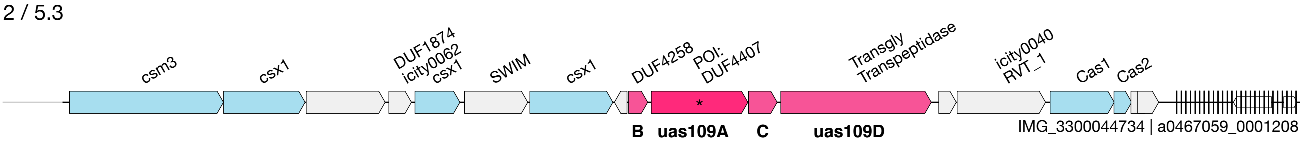

UAS-110
Auxiliary
2 / 3.5

(mega peptidase)
IMG_3300001598&&EMG_10018732&&309_3072_1

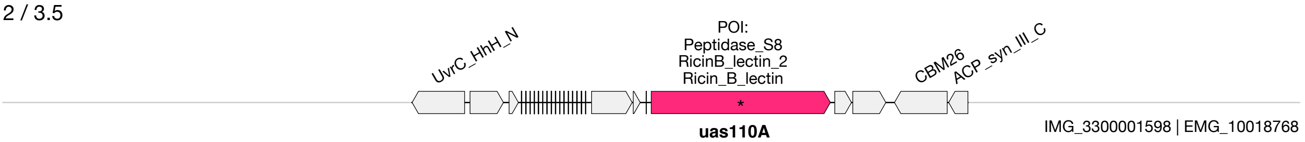

UAS-111
Auxiliary
2 / 4.4

(TIR cascade)
IMG_3300002223&&7J26845_10007859&&1305_2472_1

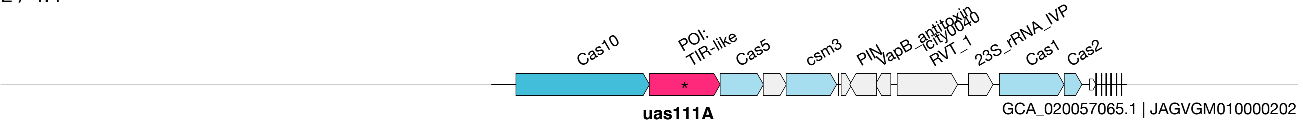

UAS-29
Auxiliary
4 / 8.6

(Phage RNAPol)
IMG_3300003177&&DffDRAFT_1003423&&1014_3828_-1

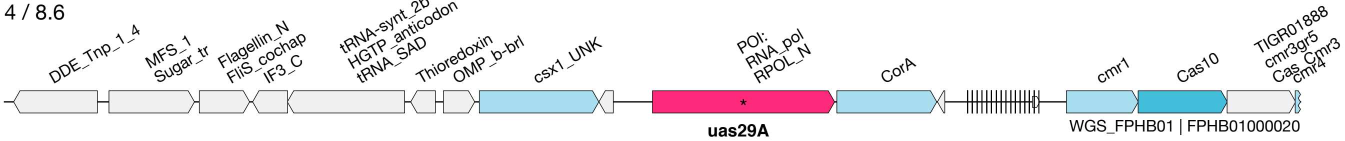

UAS-112
Auxiliary
N/A

(Csx20 operonized with Alpha Amylase)
IMG_3300004628&&a0070399_1037568&&498_2109_1

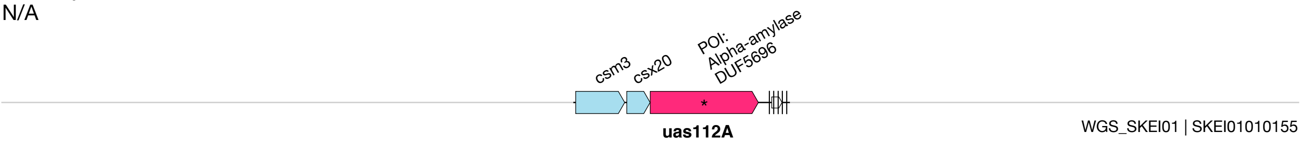

UAS-113
Auxiliary
10 / 14.6

(RNaseH)
IMG_3300005573&&a0078972_1000107&&101916_102516_1

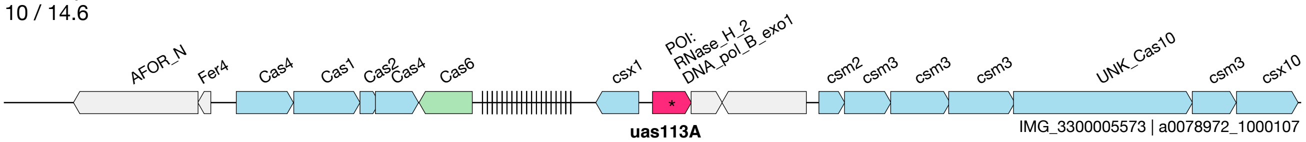

UAS-114
Auxiliary
5 / 5.5

(Tfb2_Cas3Cterminus_WYL)
IMG_3300007521&&0105044_10068709&&481_2728_1

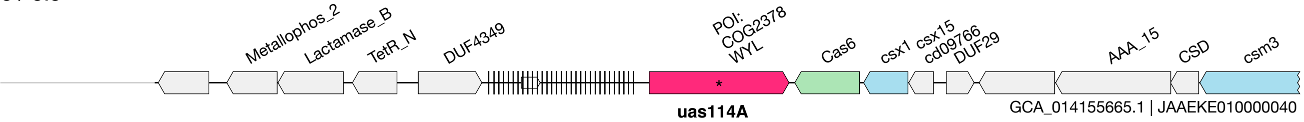

UAS-115
Auxiliary
N/A

(SLATT)
IMG_3300007964&&a0100400_1019481&&1335_1944_-1

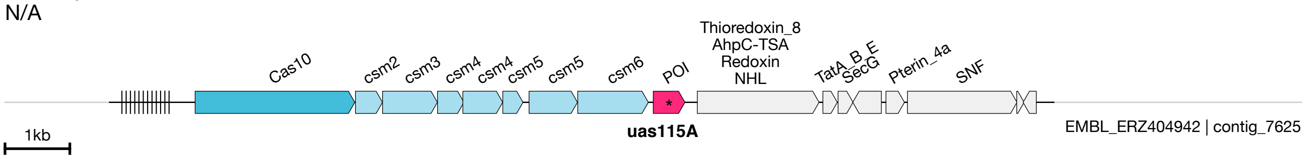

UAS-116
Auxiliary
21 / 38.1

(Moca oxoreductase + SDR)
IMG_3300008252&&0105357_10033588&&329_1373_1

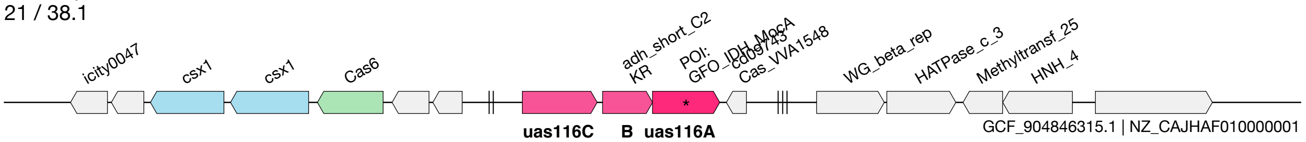

UAS-117
Auxiliary
3 / 4.8

(DUF4231)
IMG_3300009032&&0105048_10143319&&1427_2051_-1

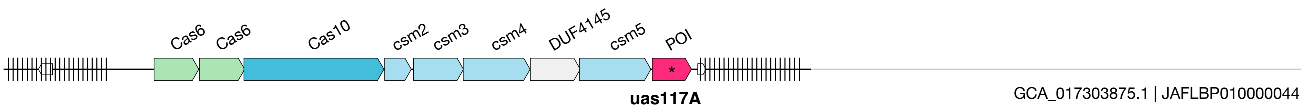

UAS-118
Auxiliary
12 / 23.7

(DUF3891)
IMG_3300009083&&0105047_10063153&&274_994_-1

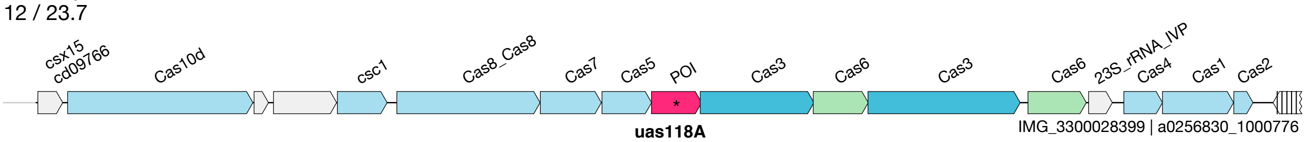

UAS-119
Auxiliary
6 / 9.9

(DUF5679, zinc ribbon)
IMG_3300009175&&0073936_10076949&&1196_1451_-1

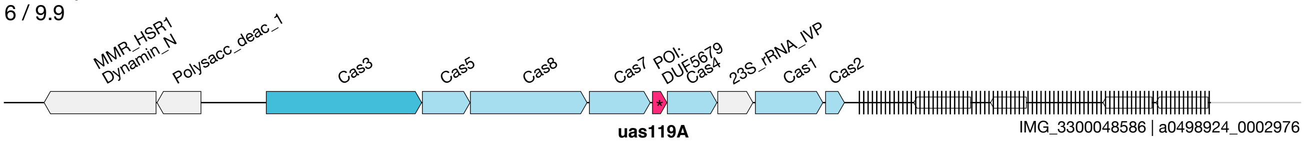

UAS-120
Auxiliary
2 / 4.1

(DUF2225)
IMG_3300009503&&0123519_10124867&&69_1659_-1

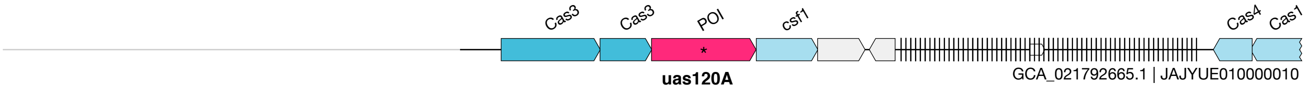

UAS-121
Auxiliary
15 / 41.0

(RNAPol + Sigma_factor + unknown gene)
IMG_3300009585&&a0105160_1000939&&4188_8637_1

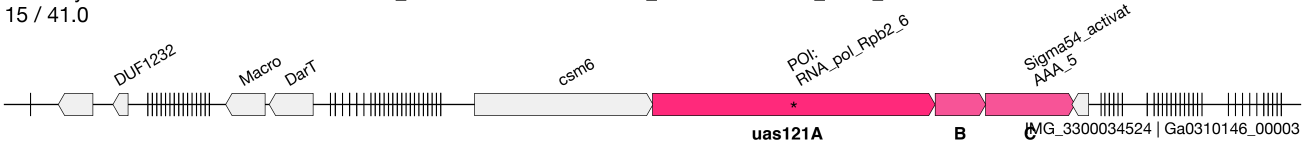

UAS-122
Auxiliary
15 / 36.2

(PRiA3 + zf)
IMG_3300012931&&0153915_10003429&&308_1193_1

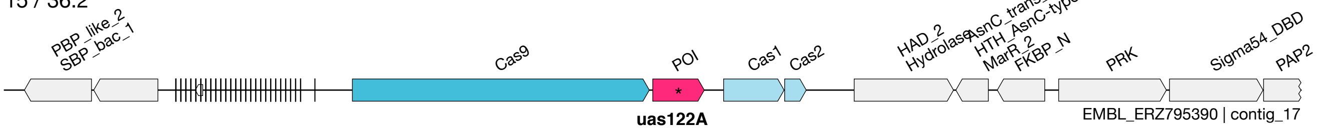

UAS-22
Auxiliary
3 / 8.1

(Cut8_TPR)
IMG_3300012943&&0164241_10011111&&1329_2733_-1

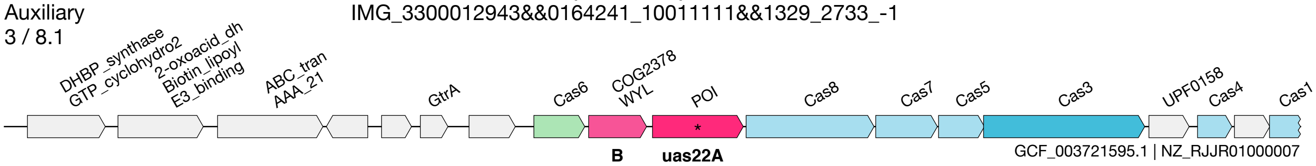

UAS-21
Auxiliary
3 / 3.7

(Cas13b + CorA)
IMG_3300012983&&0123349_10003896&&1100_3251_1

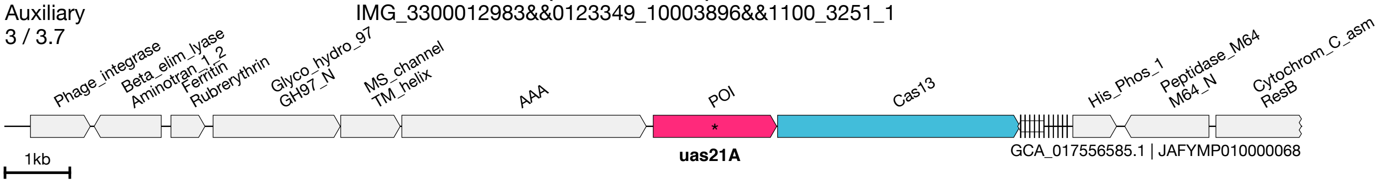

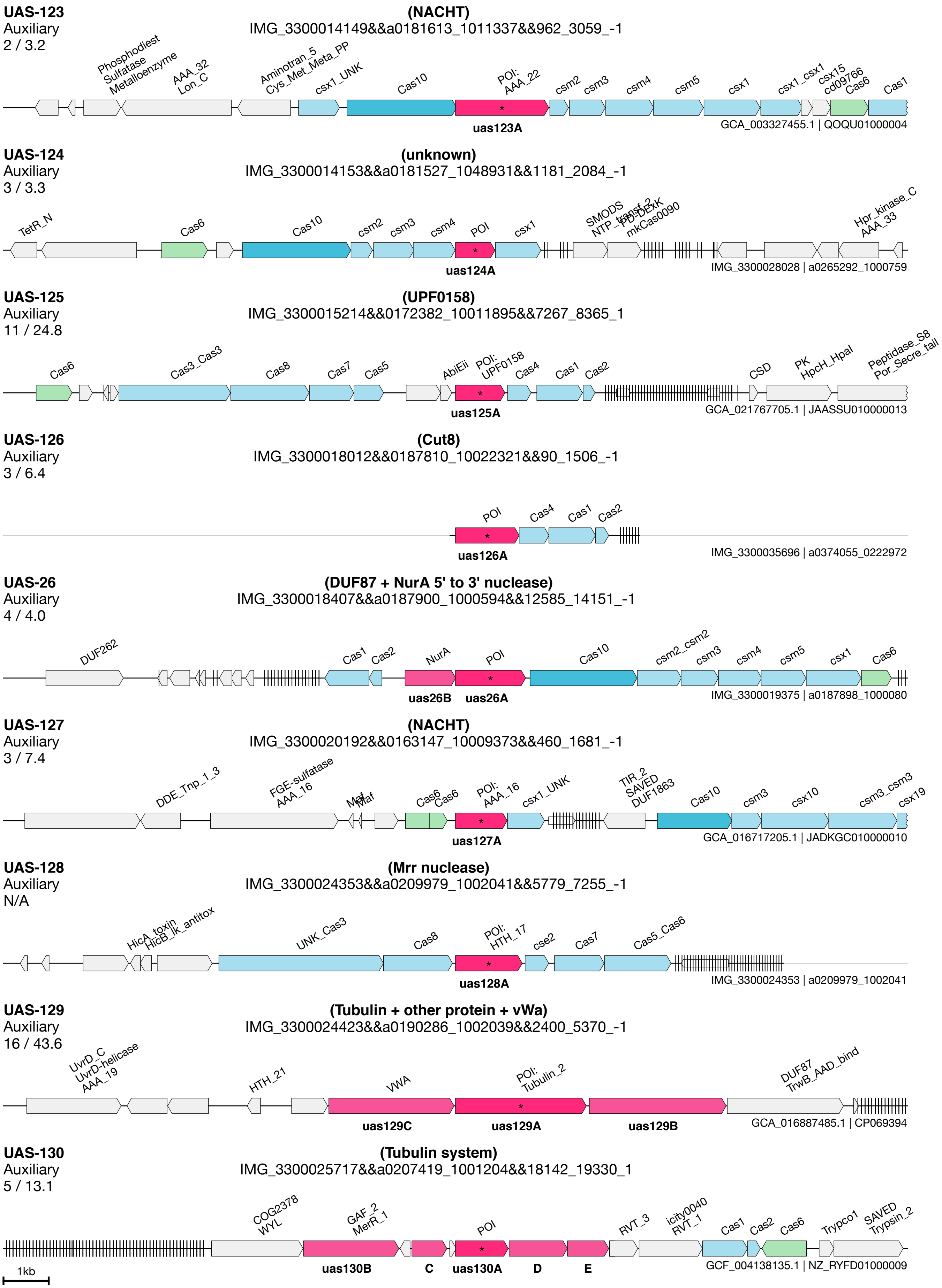
UAS-123
Auxiliary
2 / 3.2
(NACHT)
IMG_3300014149&&a0181613_1011337&&962_3059_-1
Phosphodiest
Sulfatase
Metalloenzyme
AAA_32
Lon_C
Aminotran_5
Cys_Met_Meta_PP
csx1_UNK
Cas10
POI:
AAA_22
csm2
csm3
csm4
csm5
csx1
csx1_csx1
csx15
cd09766
Cas6
Cas1
uas123A
GCA_003327455.1 | QOQU01000004
UAS-124
Auxiliary
3 / 3.3
(unknown)
IMG_3300014153&&a0181527_1048931&&1181_2084_-1
TetR_N
Cas6
Cas10
csm2
csm3
csm4
POI
csx1
SMODS
NTP_transf_2
PD-DExK
mkCas0090
Hpr_kinase_C
AAA_33
uas124A
IMG_3300028028 | a0265292_1000759
UAS-125
Auxiliary
11 / 24.8
(UPF0158)
IMG_3300015214&&0172382_10011895&&7267_8365_1
Cas6
Cas3_Cas3
Cas8
Cas7
Cas5
AbiEii
POI:
UPF0158
Cas4
Cas1
Cas2
CSD
PK
HpcH_HpaI
Peptidase_S8
Por_Secre_tail
uas125A
GCA_021767705.1 | JAASSU010000013
UAS-126
Auxiliary
3 / 6.4
(Cut8)
IMG_3300018012&&0187810_10022321&&90_1506_-1
POI
Cas4
Cas1
Cas2
uas126A
IMG_3300035696 | a0374055_0222972
UAS-26
Auxiliary
4 / 4.0
(DUF87 + NurA 5' to 3' nuclease)
IMG_3300018407&&a0187900_1000594&&12585_14151_-1
DUF262
Cas1
Cas2
NurA
POI
Cas10
csm2_csm2
csm3
csm4
csm5
csx1
Cas6
uas26B
uas26A
IMG_3300019375 | a0187898_1000080
UAS-127
Auxiliary
3 / 7.4
(NACHT)
IMG_3300020192&&0163147_10009373&&460_1681_-1
DDE_Tnp_1_3
FGE-sulfatase
AAA_16
Maf
Maf
Cas6
Cas6
POI:
AAA_16
csx1_UNK
TIR_2
SAVED
DUF1863
Cas10
csm3
csx10
csm3_csm3
csx19
uas127A
GCA_016717205.1 | JADKGC010000010
UAS-128
Auxiliary
N/A
(Mrr nuclease)
IMG_3300024353&&a0209979_1002041&&5779_7255_-1
HicA_toxin
HicB_lk_antitox
UNK_Cas3
Cas8
POI:
HTH_17
cse2
Cas7
Cas5_Cas6
uas128A
IMG_3300024353 | a0209979_1002041
UAS-129
Auxiliary
16 / 43.6
(Tubulin + other protein + vWa)
IMG_3300024423&&a0190286_1002039&&2400_5370_-1
UvrD_C
UvrD-helicase
AAA_19
HTH_21
VWA
POI:
Tubulin_2
DUF87
TrwB_AAD_bind
uas129C
uas129A
uas129B
GCA_016887485.1 | CP069394
UAS-130
Auxiliary
5 / 13.1
(Tubulin system)
IMG_3300025717&&a0207419_1001204&&18142_19330_1
COG2378
WYL
GAF_2
MerR_1
POI
RVT_3
icity0040
RVT_1
Cas1
Cas2
Cas6
Trypco1
SAVED
Trypsin_2
uas130B
C
uas130A
D
E
GCF_004138135.1 | NZ_RYFD01000009
1kb

UAS-131
Auxiliary
8 / 18.9

(TIR2)
IMG_3300026304&&a0209240_1021588&&337_1153_1

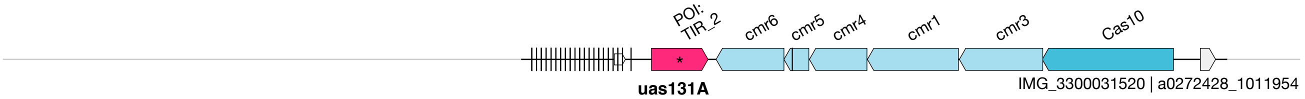

UAS-132
Auxiliary
20 / 27.4

(Cas9 associated Y1)
IMG_3300026516&&a0209573_1000325&&56350_57046_-1

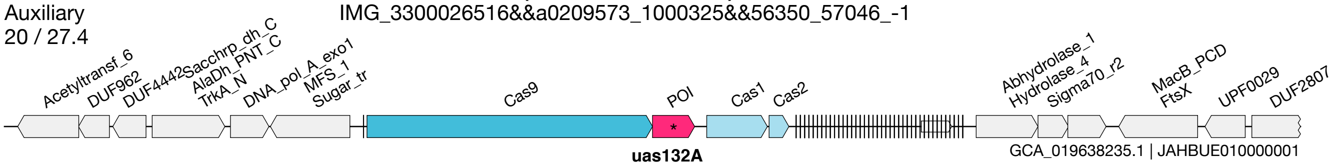

UAS-133
Auxiliary
5 / 6.4

(TPR Rho RNA binding)
IMG_3300026546&&0256913_10013832&&2_1481_1

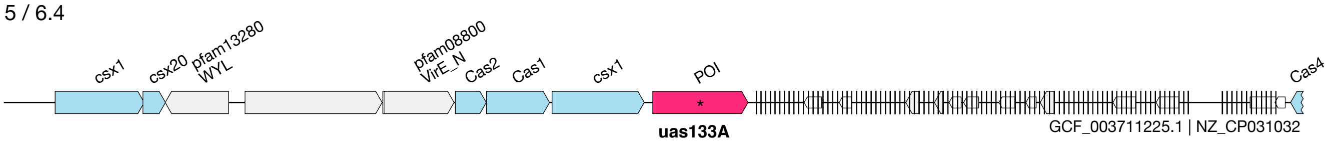

UAS-18
Auxiliary
4 / 6.6

(toprim)
IMG_3300026546&&0256913_10022199&&462_1617_-1

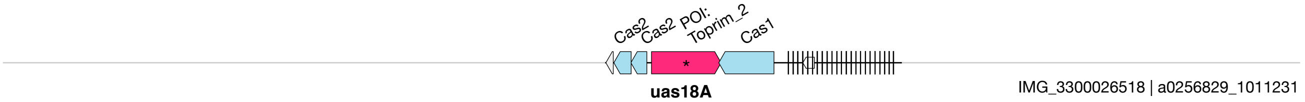

UAS-134
Auxiliary
15 / 29.8

(WYL operonized with cascade)
IMG_3300027878&&0209181_10066870&&0_1041_1

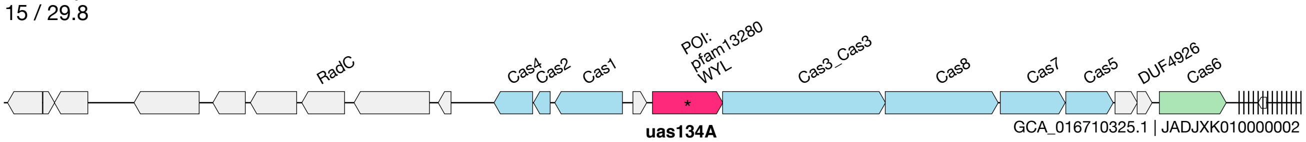

UAS-135
Auxiliary
3 / 3.0

(ATPase_DUF4435 + Cas12)
IMG_3300028048&&0256405_10015800&&2772_4671_-1

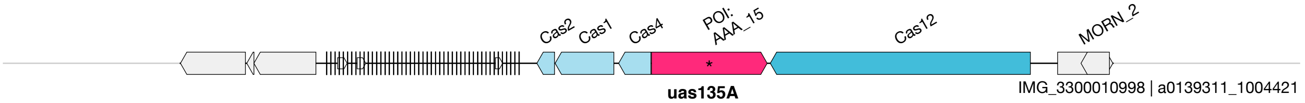

UAS-136
Auxiliary
4 / 6.7

(DUF6155)
IMG_3300028191&&a0265595_1000410&&37454_38072_1

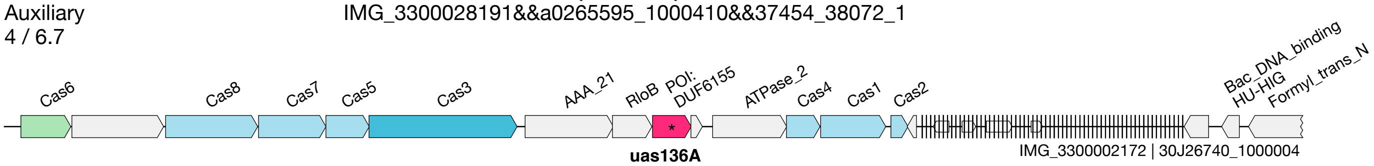

UAS-137
Auxiliary
6 / 6.7

(Holliday Junction Resolvase)
IMG_3300031520&&a0272428_1002147&&25604_25976_-1

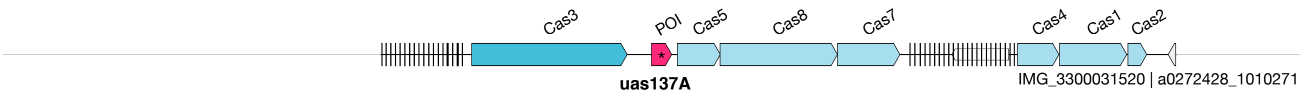

UAS-138
Auxiliary
5 / 11.2

(TIR)
IMG_3300031631&&a0307987_1000023&&70180_70972_-1

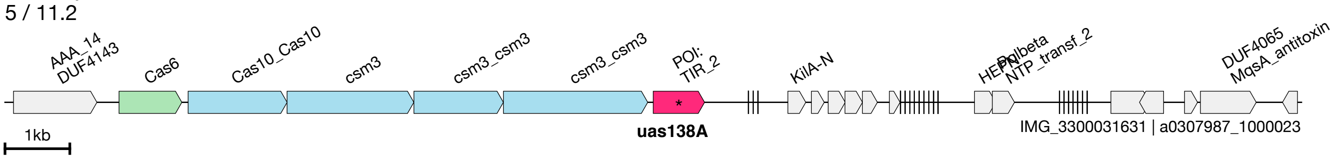

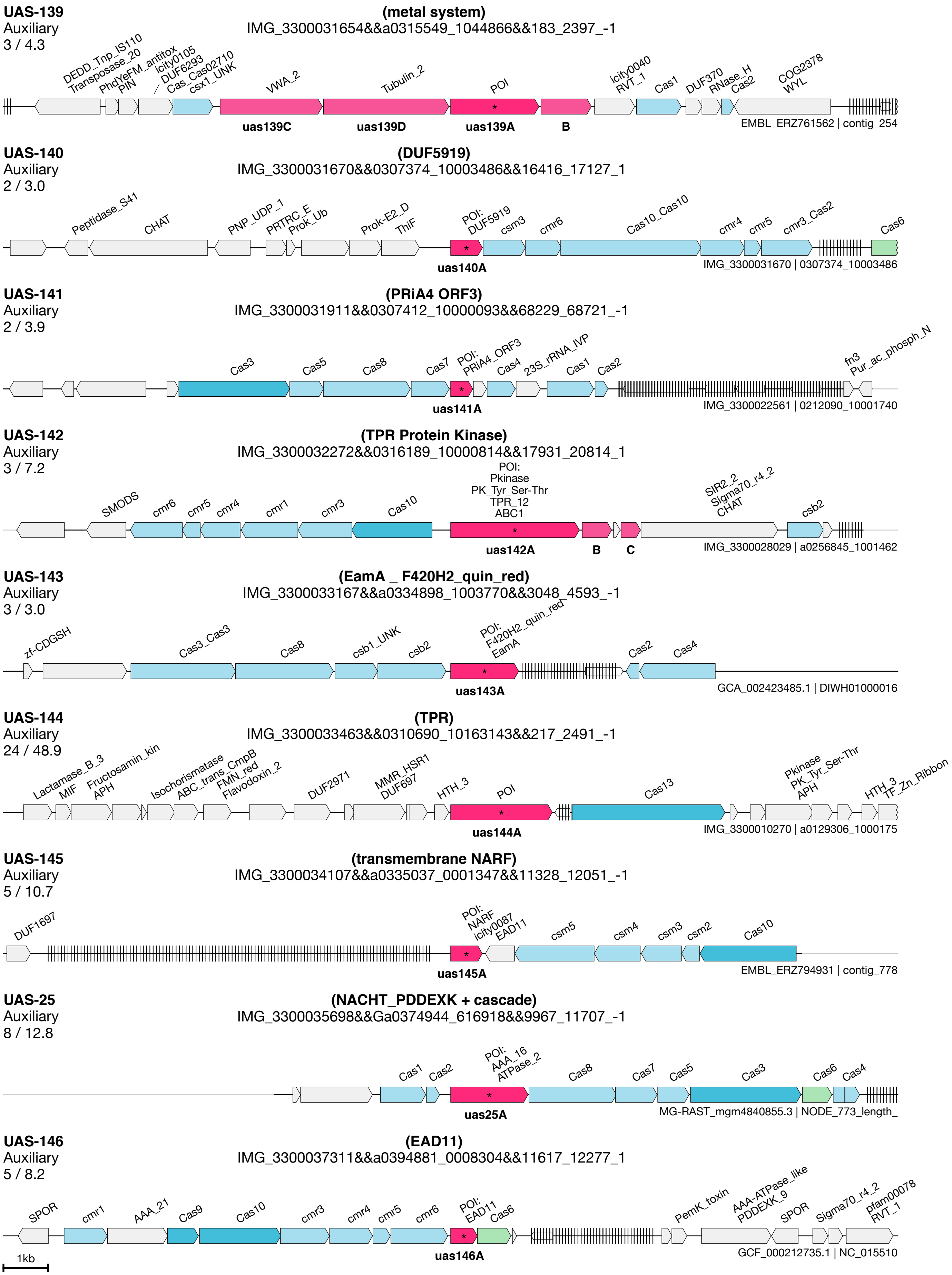

UAS-139
Auxiliary
3 / 4.3
(metal system)
IMG_3300031654&&a0315549_1044866&&183_2397_-1
DEDD_Tnp_IS110
Transposase_20
PhdYeFM_antitox
PIN
icity0105
DUF6293
Cas_Cas02710
csx1_UNK
VWA_2
Tubulin_2
POI
icity0040
RVT_1
Cas1
DUF370
RNase_H
Cas2
COG2378
WYL
uas139C
uas139D
uas139A
B
EMBL_ERZ761562 | contig_254
UAS-140
Auxiliary
2 / 3.0
(DUF5919)
IMG_3300031670&&0307374_10003486&&16416_17127_1
Peptidase_S41
CHAT
PNP_UDP_1
PRTRC_E
Prok_Ub
Prok-E2_D
ThiF
POI:
DUF5919
csm3
cmr6
Cas10_Cas10
cmr4
cmr5
cmr3_Cas2
Cas6
uas140A
IMG_3300031670 | 0307374_10003486
UAS-141
Auxiliary
2 / 3.9
(PRiA4 ORF3)
IMG_3300031911&&0307412_10000093&&68229_68721_-1
Cas3
Cas5
Cas8
Cas7
POI:
PRiA4_ORF3
Cas4
23S_rRNA_IVP
Cas1
Cas2
fn3
Pur_ac_phosph_N
uas141A
IMG_3300022561 | 0212090_10001740
UAS-142
Auxiliary
3 / 7.2
(TPR Protein Kinase)
IMG_3300032272&&0316189_10000814&&17931_20814_1
SMODS
cmr6
cmr5
cmr4
cmr1
cmr3
Cas10
POI:
Pkinase
PK_Tyr_Ser-Thr
TPR_12
ABC1
SIR2_2
Sigma70_r4_2
CHAT
csb2
uas142A
B
C
IMG_3300028029 | a0256845_1001462
UAS-143
Auxiliary
3 / 3.0
(EamA _ F420H2_quin_red)
IMG_3300033167&&a0334898_1003770&&3048_4593_-1
zf-CDGSH
Cas3_Cas3
Cas8
csb1_UNK
csb2
POI:
F420H2_quin_red
EamA
Cas2
Cas4
uas143A
GCA_002423485.1 | DIWH01000016
UAS-144
Auxiliary
24 / 48.9
(TPR)
IMG_3300033463&&0310690_10163143&&217_2491_-1
Lactamase_B_3
MIF
Fructosamin_kin
APH
Isochorismatase
ABC_trans_CmpB
FMN_red
Flavodoxin_2
DUF2971
MMR_HSR1
DUF697
HTH_3
POI
Cas13
Pkinase
PK_Tyr_Ser-Thr
APH
HTH_3
TF_Zn_Ribbon
uas144A
IMG_3300010270 | a0129306_1000175
UAS-145
Auxiliary
5 / 10.7
(transmembrane NARF)
IMG_3300034107&&a0335037_0001347&&11328_12051_-1
DUF1697
POI:
NARF
icity0087
EAD11
csm5
csm4
csm3
csm2
Cas10
uas145A
EMBL_ERZ794931 | contig_778
UAS-25
Auxiliary
8 / 12.8
(NACHT_PDDEXK + cascade)
IMG_3300035698&&Ga0374944_616918&&9967_11707_-1
Cas1
Cas2
POI:
AAA_16
ATPase_2
Cas8
Cas7
Cas5
Cas3
Cas6
Cas4
uas25A
MG-RAST_mgm4840855.3 | NODE_773_length_
UAS-146
Auxiliary
5 / 8.2
(EAD11)
IMG_3300037311&&a0394881_0008304&&11617_12277_1
SPOR
cmr1
AAA_21
Cas9
Cas10
cmr3
cmr4
cmr5
cmr6
POI:
EAD11
Cas6
PemK_toxin
AAA-ATPase_like
PDDEXK_9
SPOR
Sigma70_r4_2
pfam00078
RVT_1
uas146A
GCF_000212735.1 | NC_015510
1kb

UAS-147
Auxiliary
36 / 66.5

(WYL I-D)
IMG_3300037509&&a0394184_0000482&&19069_20062_1

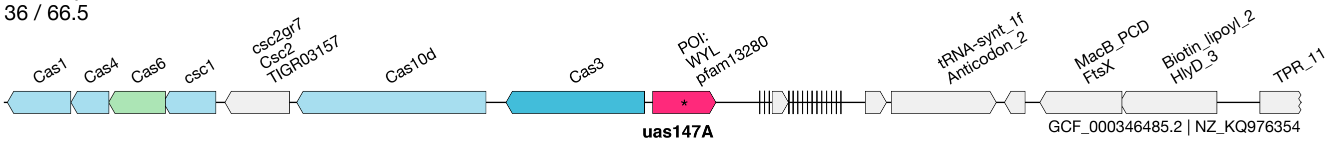

UAS-27
Auxiliary
2 / 3.2

(methyltransferase + cascade)
IMG_3300041417&&a0439242_0072051&&1423_2242_1

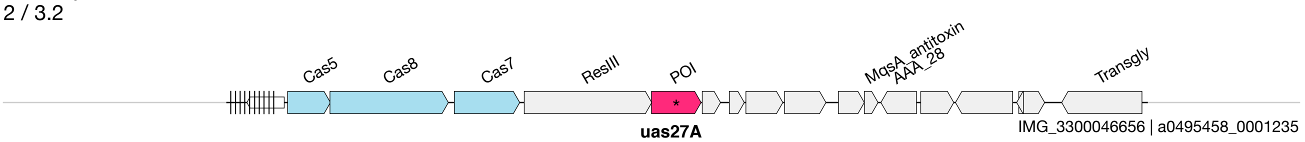

UAS-148
Auxiliary
37 / 51.3

(mega Cas8-like)
IMG_3300042340&&a0453917_0000106&&32810_35291_-1

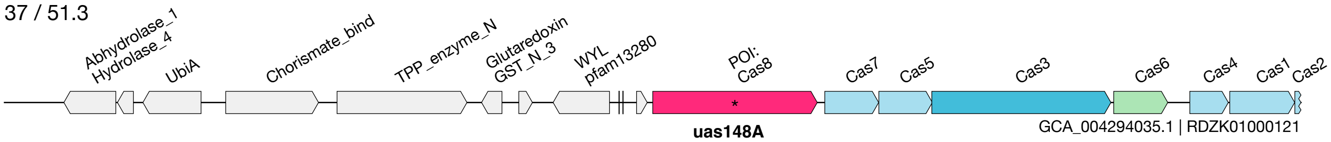

UAS-149
Auxiliary
N/A

(vWA + Tubulin)
IMG_3300043390&&Ga0454692_000076&&4479_7614_1

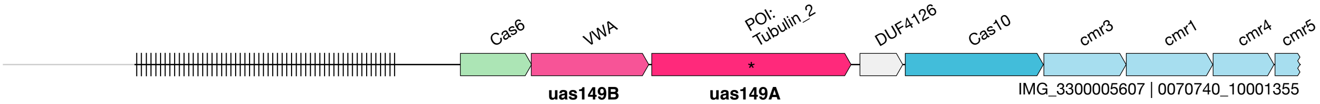

UAS-150
Auxiliary
3 / 3.0

(APH_EcKinase)
IMG_3300044730&&a0453757_0004423&&6344_8234_-1

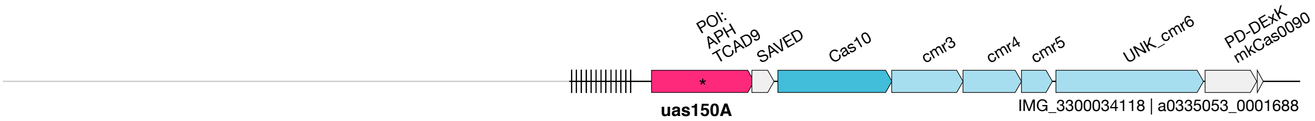

UAS-151
Auxiliary
2 / 5.0

(Cas8_Fic fusion)
IMG_3300045107&&a0484955_0013512&&3038_6074_-1

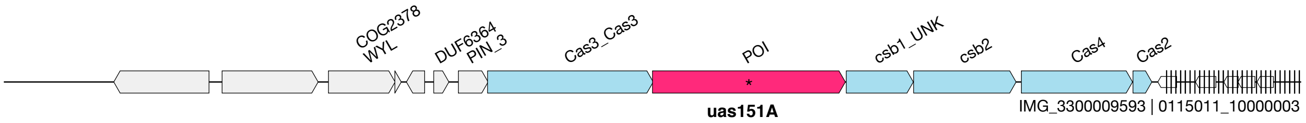

UAS-152
Auxiliary
4 / 7.4

(RNaseT)
IMG_3300046534&&a0491194_0030312&&2144_2783_-1

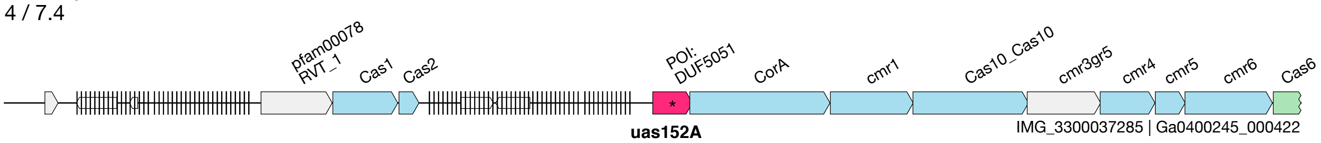

UAS-153
Auxiliary
7 / 9.9

(SNF2 instead of Cas3)
IMG_3300047317&&a0495604_0021803&&2293_4057_-1

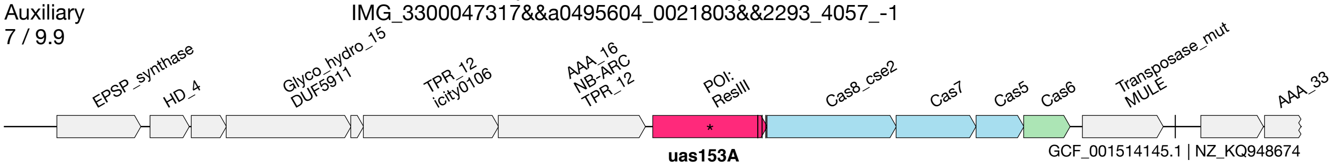

UAS-154
Auxiliary
28 / 44.9

(HIRAN Cas9, one case ATPase)
IMG_3300048583&&a0498911_0096679&&1007_1916_1

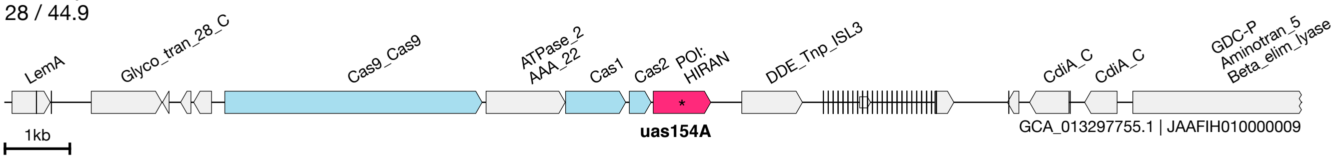

UAS-20
Auxiliary
7 / 7.4

(Cas12 + bzip)
IMG_3300048589&&a0498927_0037426&&1005_1464_1

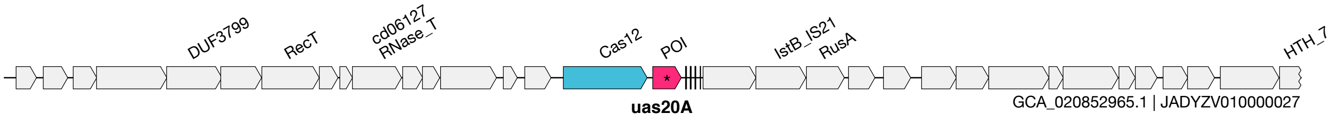

UAS-155
Auxiliary
3 / 4.4

(transmembrane_vWA + DUF4407)
IMG_3300048987&&a0467049_0000162&&19133_19934_1

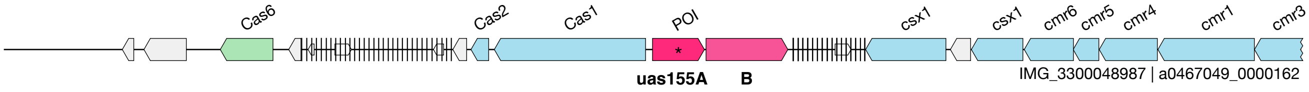

UAS-156
Auxiliary
N/A

(Mrr nuclease + ATPase)
WGS_CADHRT01&&CADHRT010000022&&25862_26885_1

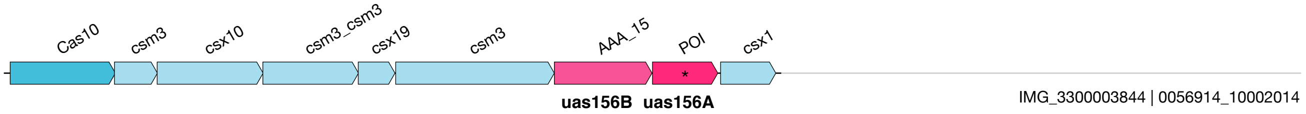

UAS-33
Auxiliary
34 / 50.0

(Prok_E2 + ThiF + ThiS + NYN + CorA)
WGS_JAAUYB01&&JAAUYB010070855&&3244_3730_1

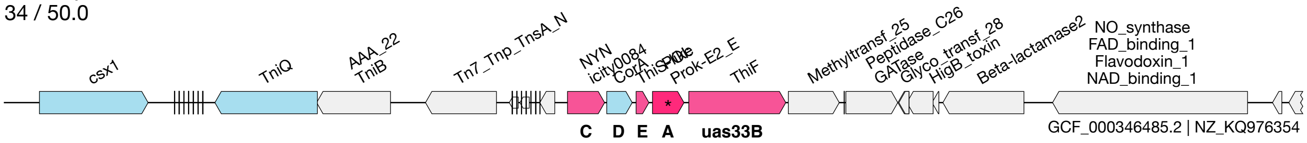

UAS-157
Auxiliary
19 / 25.4

(RecJ_HD)
WGS_MTBK01&&MTBK01177382&&2891_4688_1

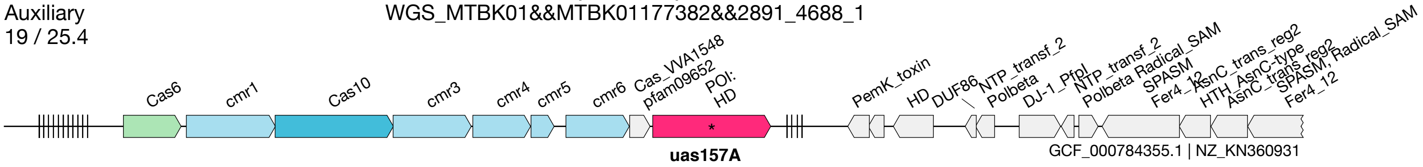

1kb

UAS-34 **(DarT DarG toxin-antitoxin ribosylation)**
Toxin-Antitoxin EMBL_ERZ1033877&&contig_98&&37922_38591_-1
15 / 22.9

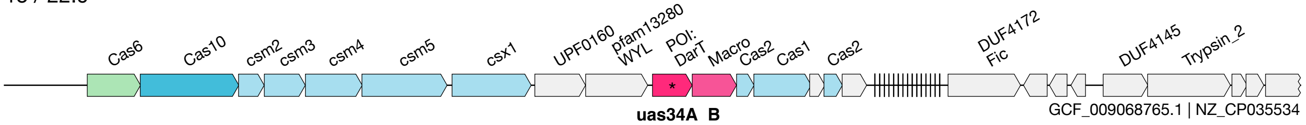

UAS-35 **(addiction gene orf associated with Cas9)**
Toxin-Antitoxin EMBL_ERZ1505400&&contig_18950&&117_258_1
9 / 9.0

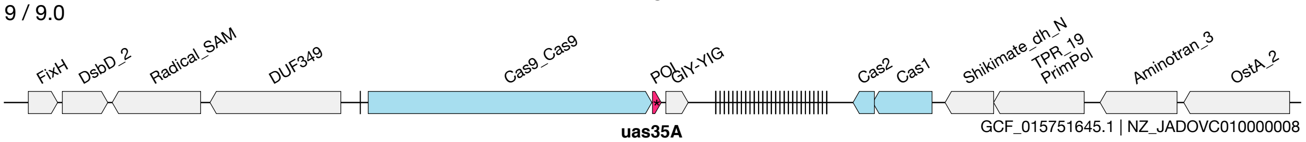

UAS-158 **(AbiE + DUF87)**
Toxin-Antitoxin EMBL_ERZ1742239&&contig_29570&&162_1638_1
8 / 18.7

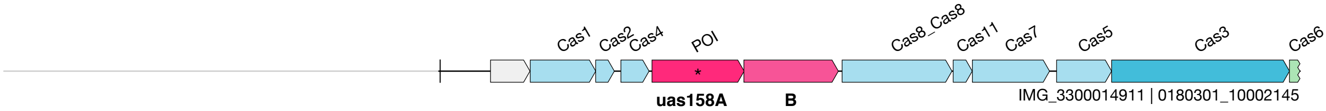

UAS-159 **(Nucleotidyltransferase HEPN, antitoxin)**
Toxin-Antitoxin EMBL_ERZ511474&&contig_784&&79398_79716_-1
18 / 48.1

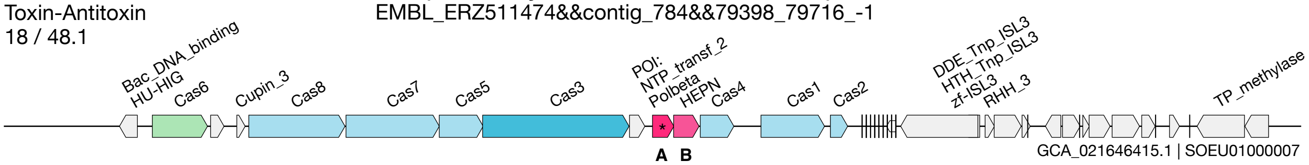

UAS-160 **(DUF86 + Nucleotidyltransferase)**
Toxin-Antitoxin GCA_013285545.1&&JAAXLU010000071&&33674_34109_-1
104 / 173.3

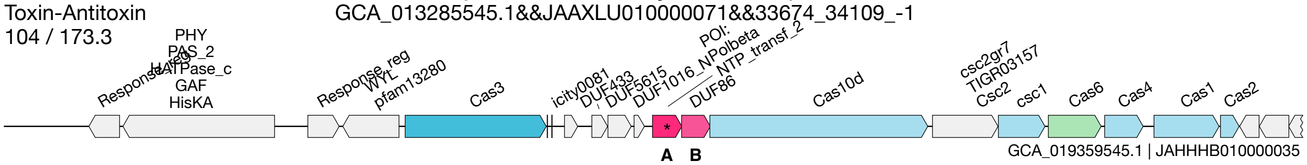

UAS-161 **(Cas9 HicAB TA)**
Toxin-Antitoxin GCF_003337175.1&&NZ_NETH01000035&&6357_6546_1
3 / 3.0

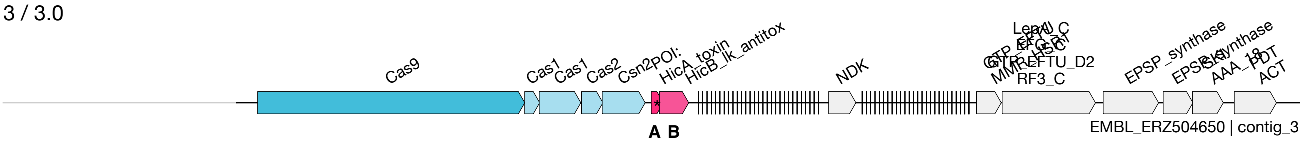

UAS-162 **(Cas9 TA system)**
Toxin-Antitoxin GCF_012037625.1&&NZ_JAAVJS010000002&&280535_280832_-1
25 / 36.4

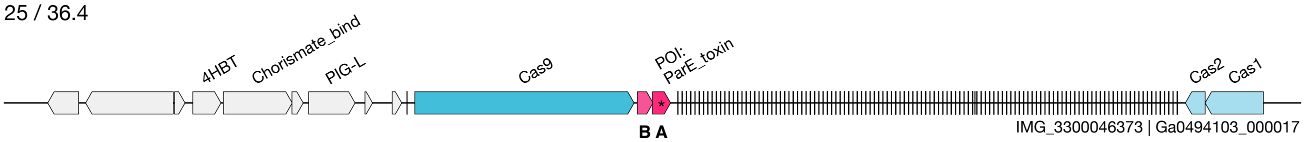

UAS-163 **(nucleotidyltransferase + HTH)**
Toxin-Antitoxin IMG_3300001380&&6J14229_10033242&&787_1210_1
4 / 6.9

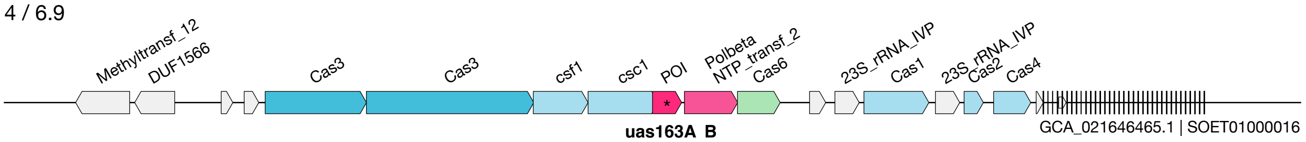

UAS-164 **(AbiEii)**
Toxin-Antitoxin IMG_3300005452&&a0068706_1004208&&1242_2085_1
2 / 5.2

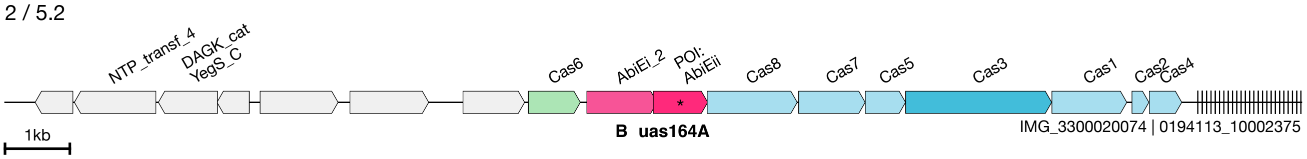

UAS-165
Toxin-Antitoxin
11 / 16.6

(TA DUF3368 _ UPF0175)
IMG_3300009455&&0114939_10000745&&24543_24870_1

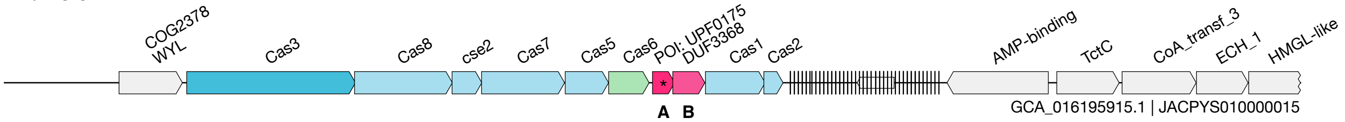

UAS-166
Toxin-Antitoxin
N/A

(AbiEi + AbiEii)
IMG_3300013103&&0164318_10140015&&1204_2056_1

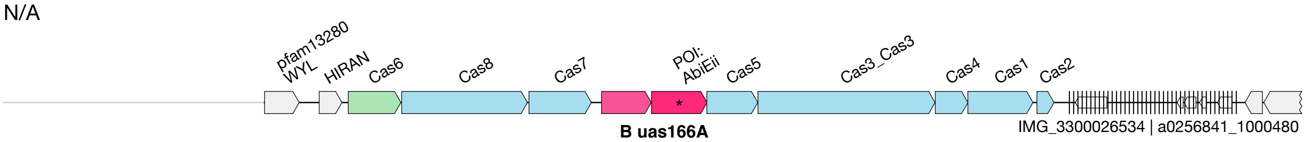

UAS-167
Toxin-Antitoxin
7 / 7.3

(TA system)
IMG_3300014205&&0172380_10021645&&1390_1759_1

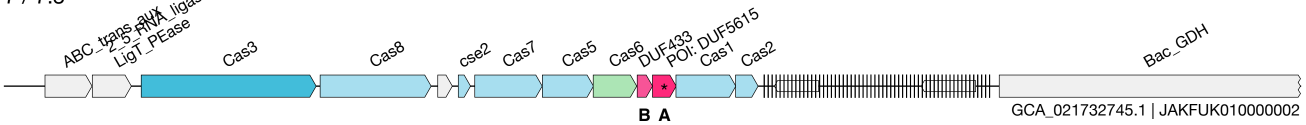

UAS-168
Toxin-Antitoxin
3 / 3.9

(PHD + PIN TA)
IMG_3300025308&&0209211_10037079&&3340_3646_-1

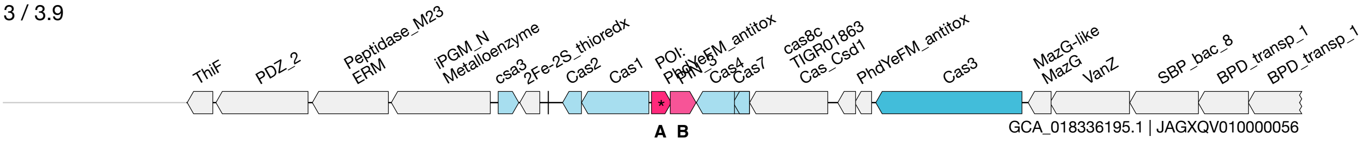

UAS-169
Toxin-Antitoxin
3 / 5.2

(DUF370 + RNaseH TA)
IMG_3300031358&&a0307438_1001316&&8395_8671_1

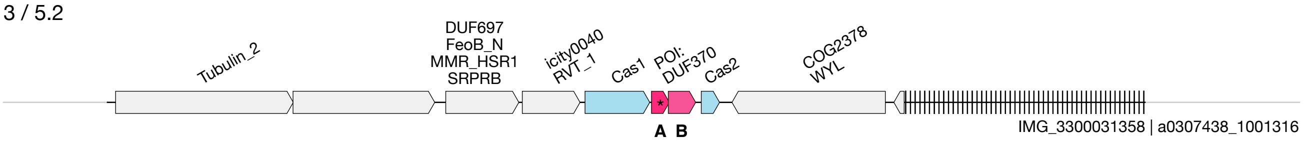

UAS-170
Toxin-Antitoxin
3 / 3.0

(DUF2442 + DUF4160)
IMG_3300034066&&a0335019_0002251&&7799_8117_-1

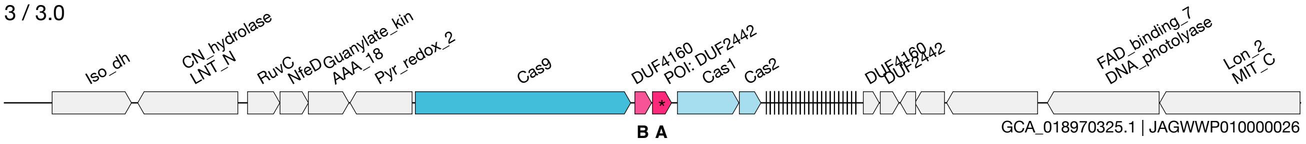

UAS-171
Toxin-Antitoxin
43 / 48.3

(small ORFs)
IMG_3300044960&&Ga0484958_001236&&27209_27470_-1

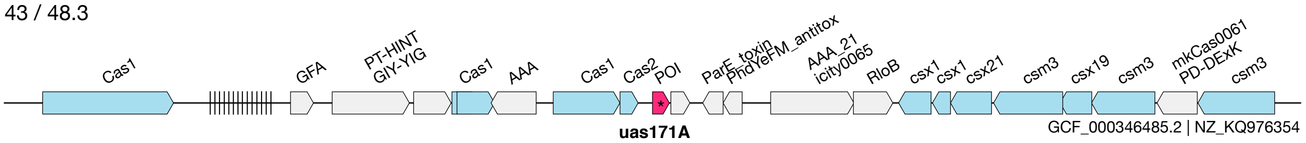

UAS-172
Toxin-Antitoxin
23 / 35.0

(RHH TA system)
IMG_3300045747&&a0485825_0053347&&1617_1944_1

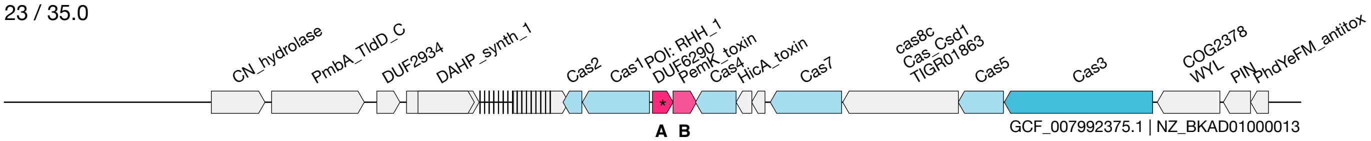

UAS-173
Toxin-Antitoxin
42 / 56.2

(DUF3368 TA system)
MG-RAST_mgm4611649.3&&465870&&0_567_1

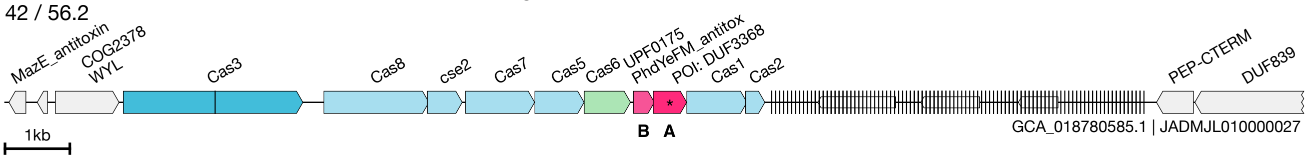

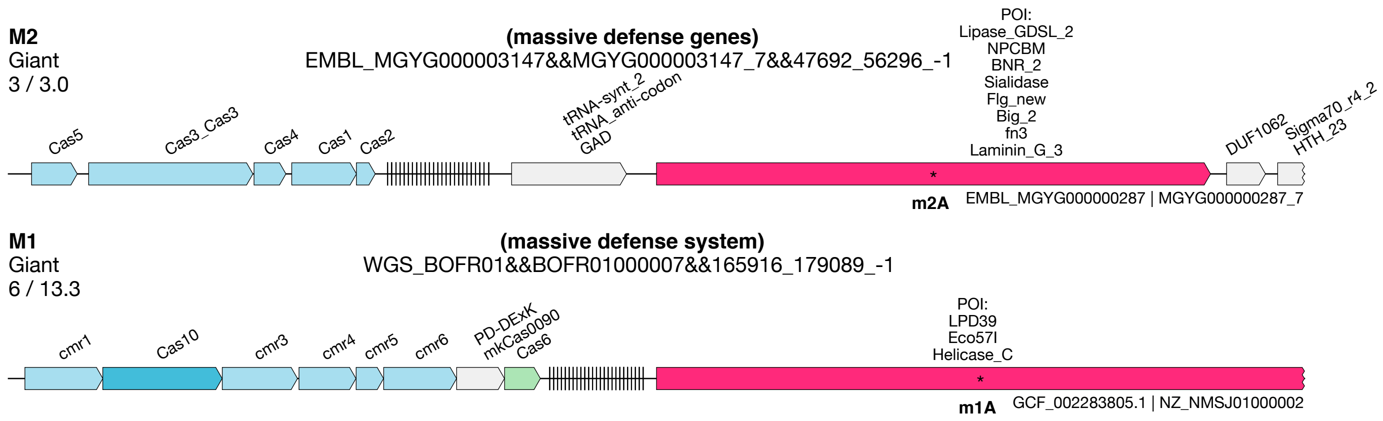
M2
Giant
3 / 3.0
(massive defense genes)
EMBL_MGYG000003147&&MGYG000003147_7&&47692_56296_-1
POI:
Lipase_GDSL_2
NPCBM
BNR_2
Sialidase
Flg_new
Big_2
fn3
Laminin_G_3
Cas5
Cas3_Cas3
Cas4
Cas1
Cas2
tRNA-synt_2
tRNA_anti-codon
GAD
DUF1062
Sigma70_r4_2
HTH_23
*
m2A
EMBL_MGYG000000287 | MGYG000000287_7
M1
Giant
6 / 13.3
(massive defense system)
WGS_BOFR01&&BOFR01000007&&165916_179089_-1
POI:
LPD39
Eco57I
Helicase_C
cmr1
Cas10
cmr3
cmr4
cmr5
cmr6
PD-DExK
mkCas0090
Cas6
*
m1A
GCF_002283805.1 | NZ_NMSJ01000002

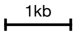
1kb

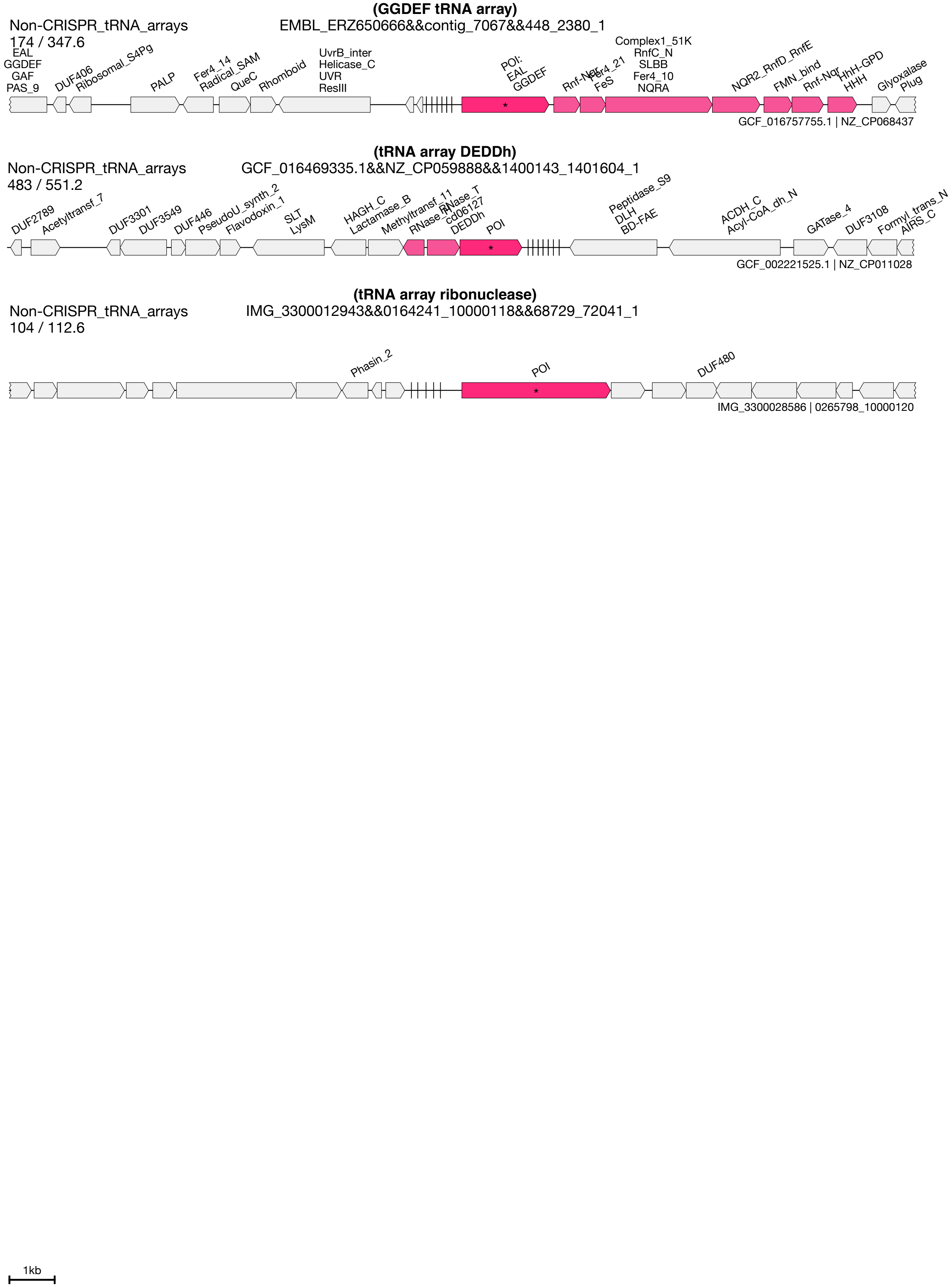
(GGDEF tRNA array)
Non-CRISPR_tRNA_arrays
EMBL_ERZ650666&&contig_7067&&448_2380_1
174 / 347.6
EAL
GGDEF
GAF
PAS_9
DUF406
Ribosomal_S4Pg
PALP
Fer4_14
Radical_SAM
QueC
Rhomboid
UvrB_inter
Helicase_C
UVR
ResIII
POI:
EAL
GGDEF
Rnf-Nqr
Fer4_21
FeS
Complex1_51K
RnfC_N
SLBB
Fer4_10
NQRA
NQR2_RnfD_RnfE
FMN_bind
Rnf-Nqr
HhH-GPD
HHH
Glyoxalase
Plug
GCF_016757755.1 | NZ_CP068437
(tRNA array DEDDh)
Non-CRISPR_tRNA_arrays
GCF_016469335.1&&NZ_CP059888&&1400143_1401604_1
483 / 551.2
DUF2789
Acetyltransf_7
DUF3301
DUF3549
DUF446
PseudoU_synth_2
Flavodoxin_1
SLT
LysM
HAGH_C
Lactamase_B
Methyltransf_11
RNase_T
RNase_T
cd06127
DEDDh
POI
Peptidase_S9
DLH
BD-FAE
ACDH_C
Acyl-CoA_dh_N
GATase_4
DUF3108
Formyl_trans_N
AIRS_C
GCF_002221525.1 | NZ_CP011028
(tRNA array ribonuclease)
Non-CRISPR_tRNA_arrays
IMG_3300012943&&0164241_10000118&&68729_72041_1
104 / 112.6
Phasin_2
POI
DUF480
IMG_3300028586 | 0265798_10000120
1kb

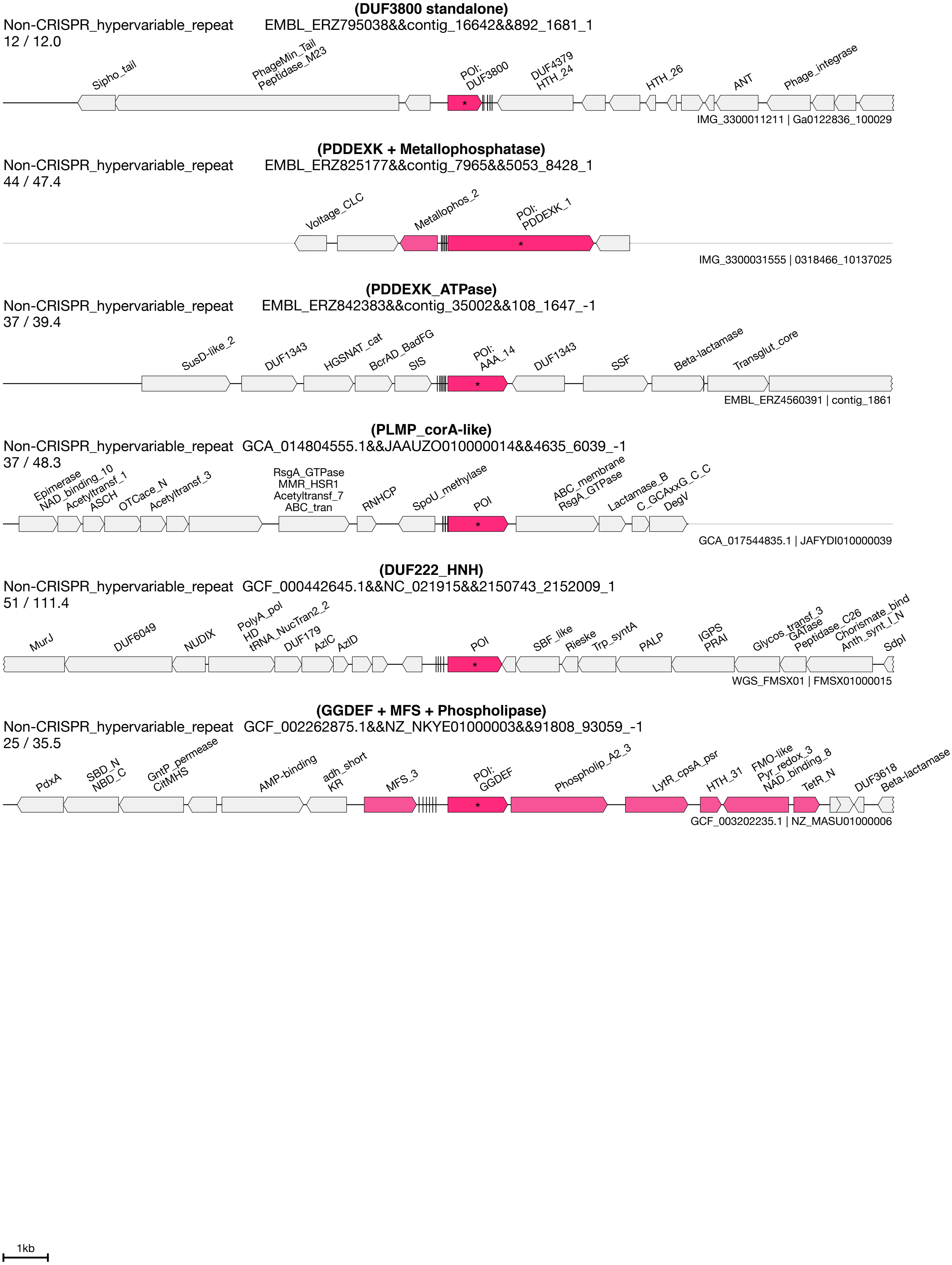
(DUF3800 standalone)
Non-CRISPR_hypervariable_repeat EMBL_ERZ795038&&contig_16642&&892_1681_1
12 / 12.0
Sipho_tail
PhageMin_Tail
Peptidase_M23
POI:
DUF3800
DUF4379
HTH_24
HTH_26
ANT
Phage_integrase
*
IMG_3300011211 | Ga0122836_100029
(PDDEXK + Metallophosphatase)
Non-CRISPR_hypervariable_repeat EMBL_ERZ825177&&contig_7965&&5053_8428_1
44 / 47.4
Voltage_CLC
Metallophos_2
POI:
PDDEXK_1
*
IMG_3300031555 | 0318466_10137025
(PDDEXK_ATPase)
Non-CRISPR_hypervariable_repeat EMBL_ERZ842383&&contig_35002&&108_1647_-1
37 / 39.4
SusD-like_2
DUF1343
HGSNAT_cat
BcrAD_BadFG
SIS
POI:
AAA_14
DUF1343
SSF
Beta-lactamase
Transglut_core
*
EMBL_ERZ4560391 | contig_1861
(PLMP_corA-like)
Non-CRISPR_hypervariable_repeat GCA_014804555.1&&JAAUZO010000014&&4635_6039_-1
37 / 48.3
Epimerase
NAD_binding_10
Acetyltransf_1
ASCH
OTCace_N
Acetyltransf_3
RsgA_GTPase
MMR_HSR1
Acetyltransf_7
ABC_tran
RNHCP
SpoU_methylase
POI
ABC_membrane
RsgA_GTPase
Lactamase_B
C_GCAxxG_C_C
DegV
*
GCA_017544835.1 | JAFYDI010000039
(DUF222_HNH)
Non-CRISPR_hypervariable_repeat GCF_000442645.1&&NC_021915&&2150743_2152009_1
51 / 111.4
MurJ
DUF6049
NUDIX
PolyA_pol
HD
tRNA_NucTran2_2
DUF179
AzlC
AzlD
POI
SBF_like
Rieske
Trp_syntA
PALP
IGPS
PRAI
Glycos_transf_3
GATase
Peptidase_C26
Chorismate_bind
Anth_synt_I_N
Sdpl
*
WGS_FMSX01 | FMSX01000015
(GGDEF + MFS + Phospholipase)
Non-CRISPR_hypervariable_repeat GCF_002262875.1&&NZ_NKYE01000003&&91808_93059_-1
25 / 35.5
PdxA
SBD_N
NBD_C
GntP_permease
CitMHS
AMP-binding
adh_short
KR
MFS_3
POI:
GGDEF
Phospholip_A2_3
LytR_cpsA_psr
HTH_31
FMO-like
Pyr_redox_3
NAD_binding_8
TetR_N
DUF3618
Beta-lactamase
*
GCF_003202235.1 | NZ_MASU01000006
1kb

Fig. S14. Limited loci of all systems identified in this study

Example loci (up to one page) of each identified system (ranked by length to contig edge of the characteristic gene corresponding to the cluster id). Associated genes are not explicitly shown but can be found in **Data S2-3** and have visualizations for a representative locus available in Fig. S13. In contrast to Fig. S13, only the characteristic gene from the cluster id is show in red (other know associated genes are not demarcated). The cluster id below the characteristic domains associated with the system (top middle bold for each system) is shown with regular font and corresponds to the gene with the asterisk. Signature effector genes are colored in darker blue (Cas6, Cas9, Cas10, Cas12, Cas13). Other Cas genes are shown as light blue. Cas6 is shown as light green. Unrelated or other genes are shown as light grey. White genes with shorter height are genes that overlap with CRISPR arrays (vertical black lines). Above each gene are redundancy reduced predictions of the protein domains via HMMER on the PFAM database using a minimum bit score threshold of 18. Enhanced CRISPR association scores for the cluster ids are shown as applicable: some systems were included if they were identified through protein-protein associations, or exceptionally low association score (in the case of PhageCas9), even if their enhanced CRISPR-association score did not meet the filtering threshold, in which case the CRISPR association-scores are not shown.

Note: Fig. S14 spans pages **87 to 283**

Sub table of contents

Effector Modules 87

Cas Fusions 100

Acquisition 107

CARF 132

Auxiliary 151

Toxin-Antitoxin 255

Massive Genes 273

Non-CRISPR tRNA arrays 275

Non-CRISPR arrays with hypervariable spacers 278

A

III-UAS **(new III subtype)** 6 / 7.0

Effector-Modules EMBL_ERZ584659&&contig_33476&&1228_2278_-1

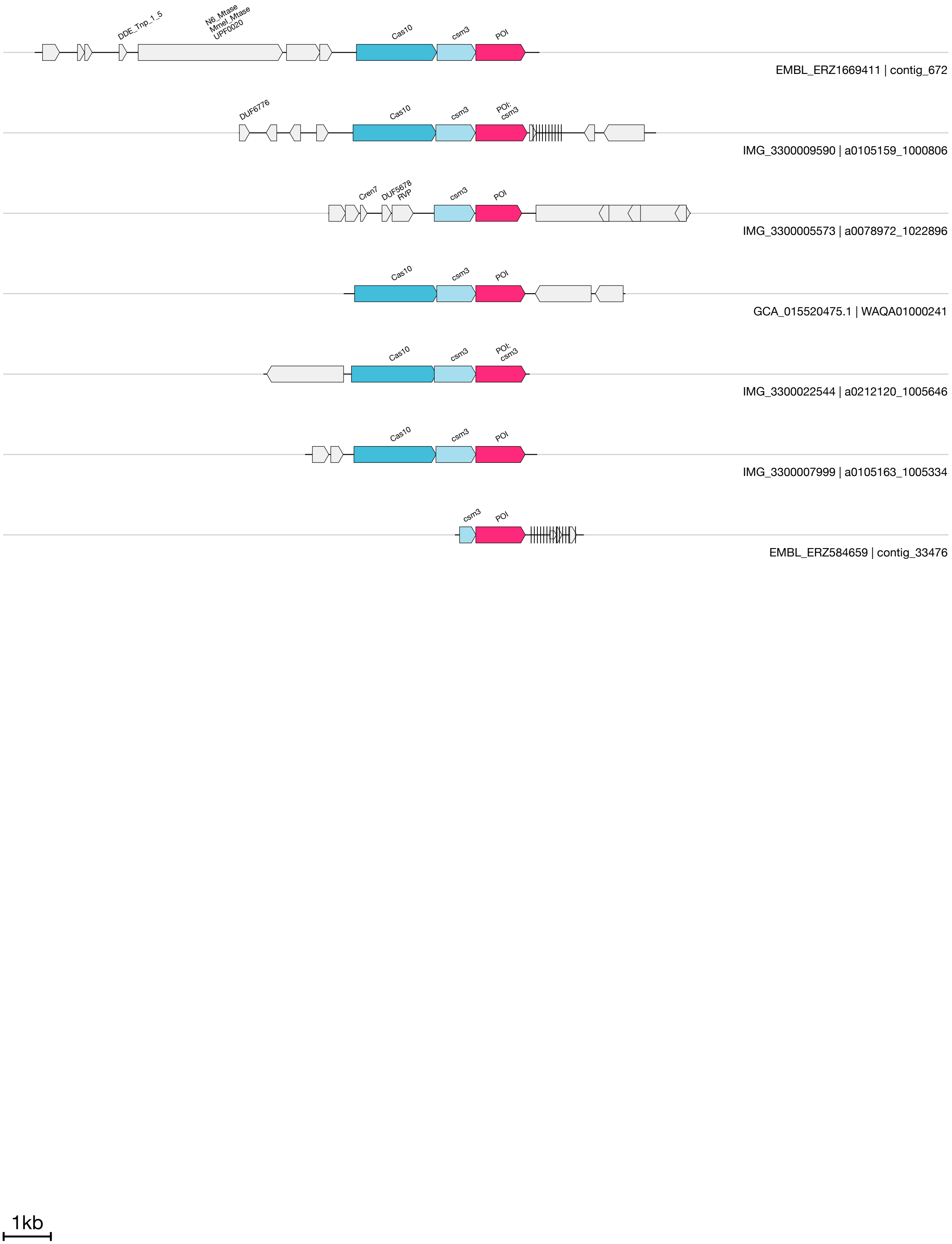

B

PhageCas9
Effector-Modules

(PhageCas9 + Helicase)
EMBL_ERZ795075&&contig_381&&19666_22555_1

N/A

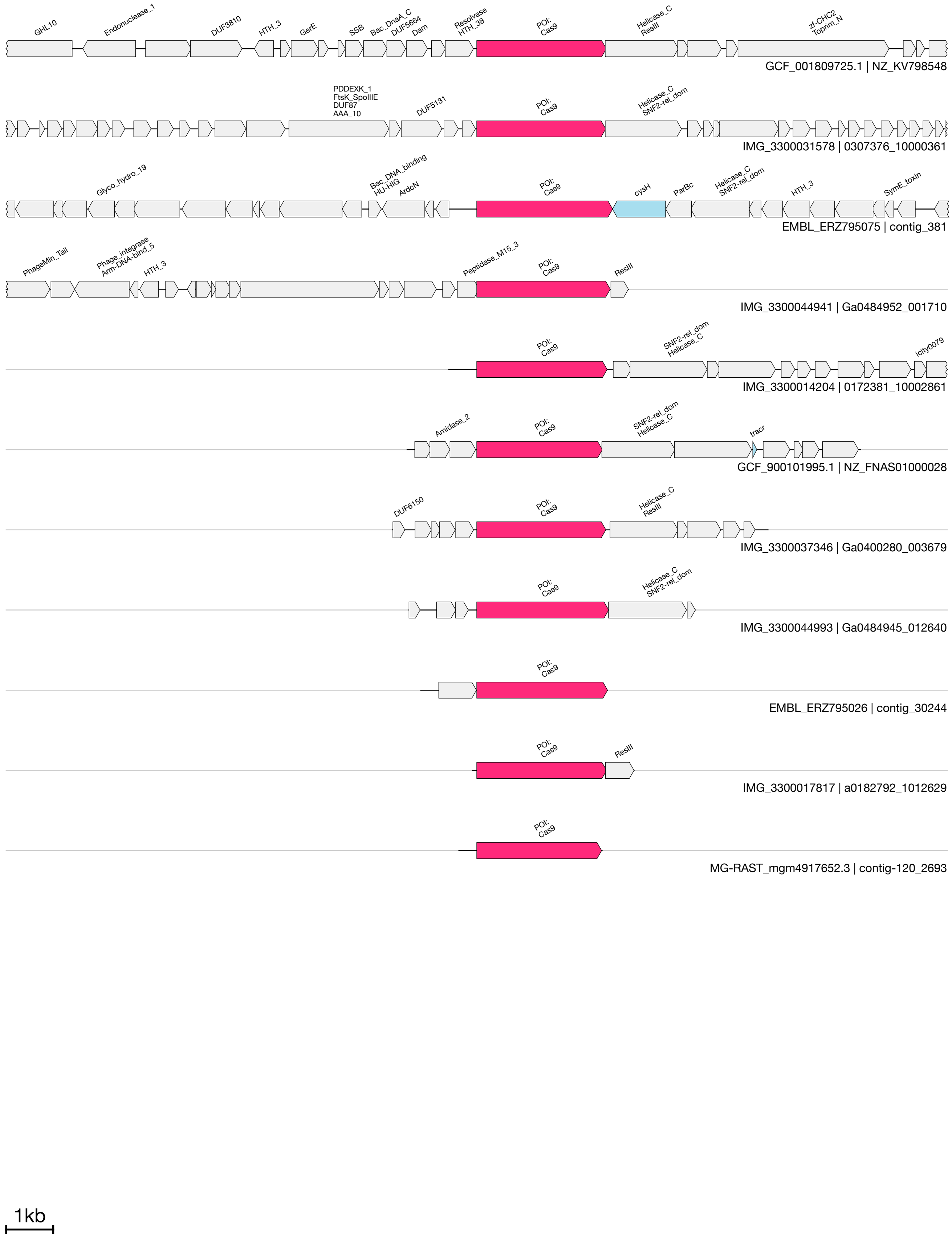

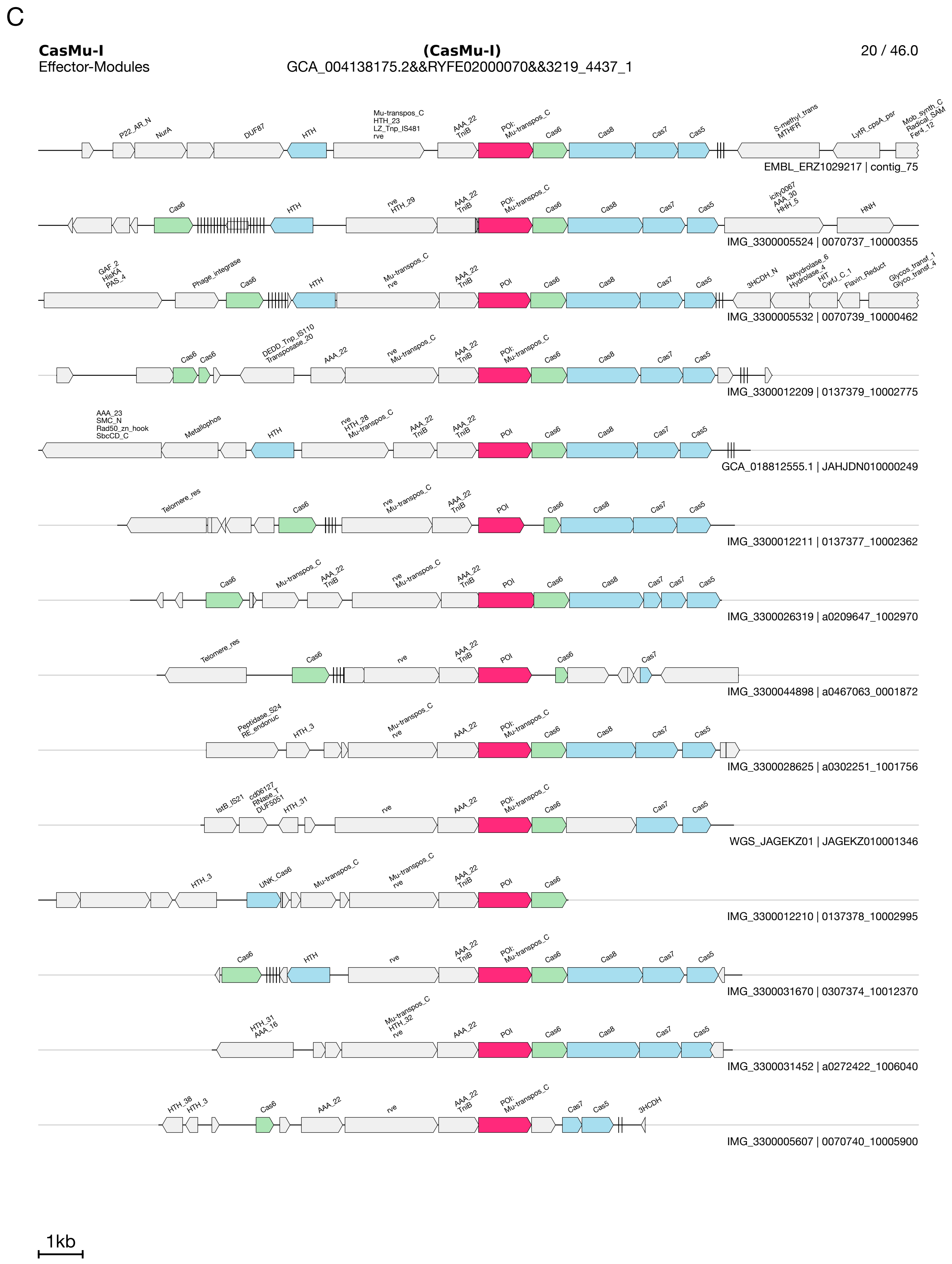
C
CasMu-I
Effector-Modules
(CasMu-I)
GCA_004138175.2&&RYFE02000070&&3219_4437_1
20 / 46.0
EMBL_ERZ1029217 | contig_75
IMG_3300005524 | 0070737_10000355
IMG_3300005532 | 0070739_10000462
IMG_3300012209 | 0137379_10002775
GCA_018812555.1 | JAHJDN010000249
IMG_3300012211 | 0137377_10002362
IMG_3300026319 | a0209647_1002970
IMG_3300044898 | a0467063_0001872
IMG_3300028625 | a0302251_1001756
WGS_JAGEKZ01 | JAGEKZ010001346
IMG_3300012210 | 0137378_10002995
IMG_3300031670 | 0307374_10012370
IMG_3300031452 | a0272422_1006040
IMG_3300005607 | 0070740_10005900
1kb

D

Cas12_DUF3800_TPR_UvrD_TPR **(DUF3800_TPR + Cas12)** 11 / 12.0

Effector-Modules GCA_018268035.1&&JAFDXI010000155&&1871_5534_-1

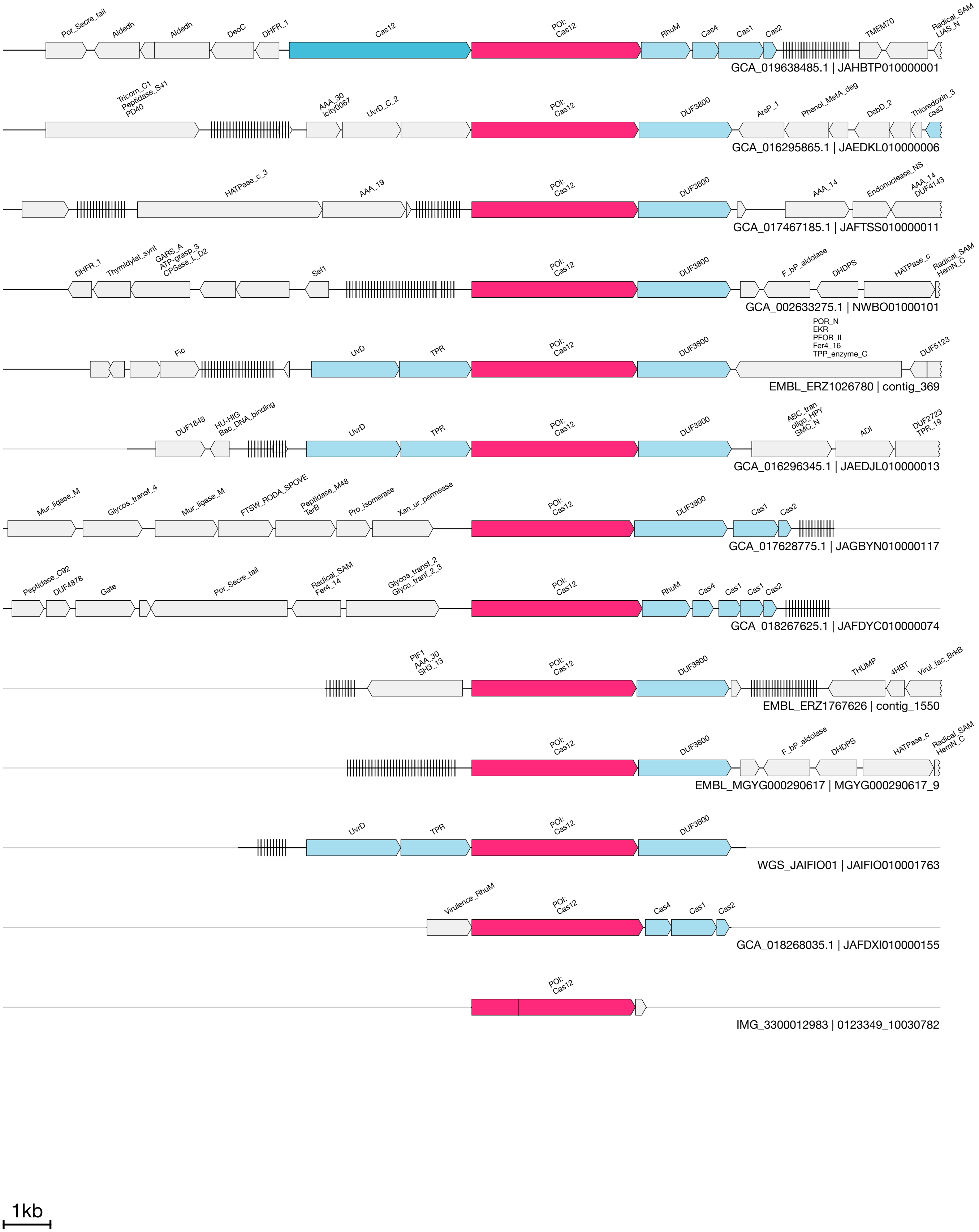

E

UAS-36 **(Cas3_PDDEXK)** 3 / 3.0

Effector-Modules GCA_018369695.1&&JAGZBW010000011&&6634_10387_-1

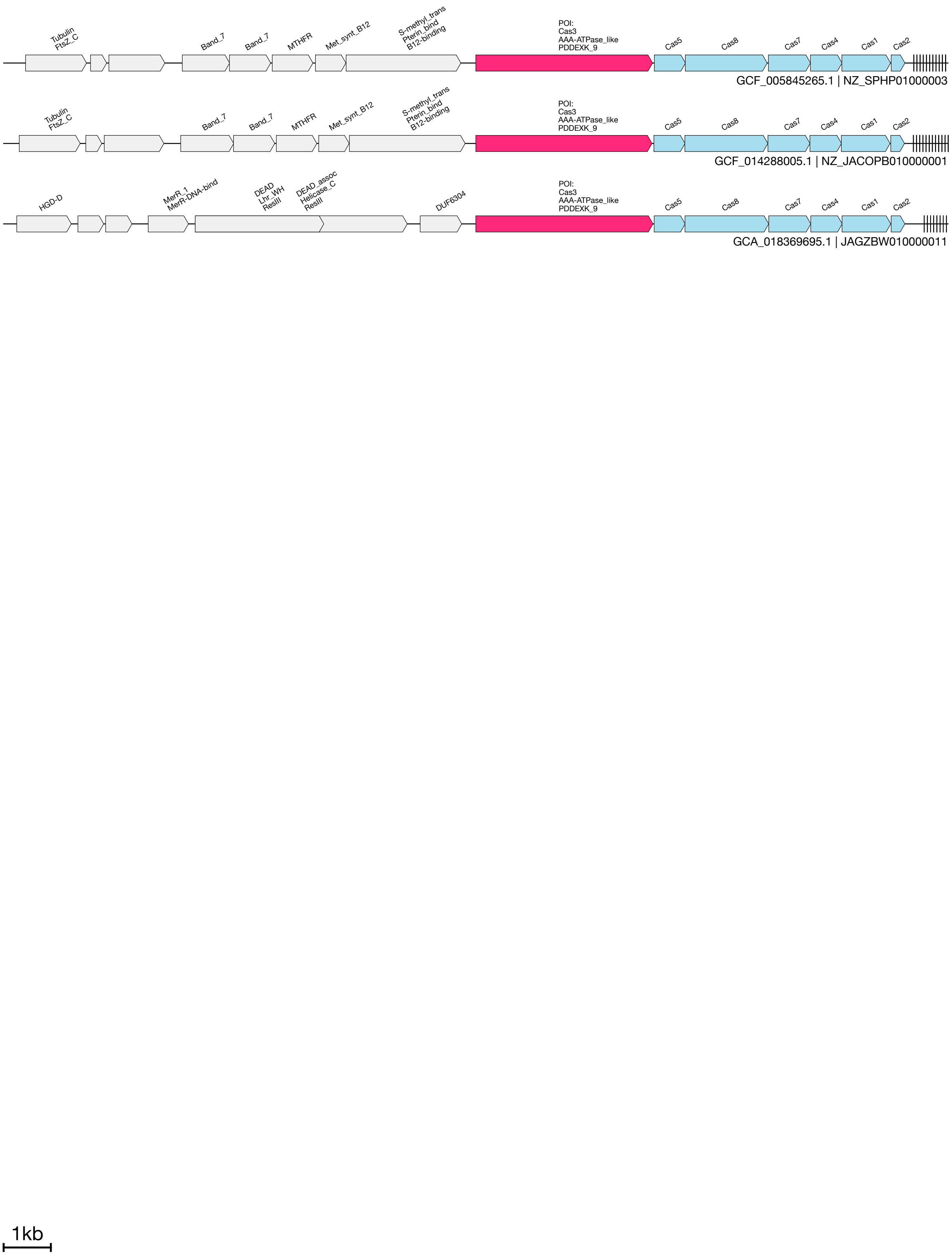

F

Cas11-VRR **(Cse2_VRR)** 7 / 7.2
Effector-Modules IMG_3300006527&&Ga0100159_100137&&71478_72558_1

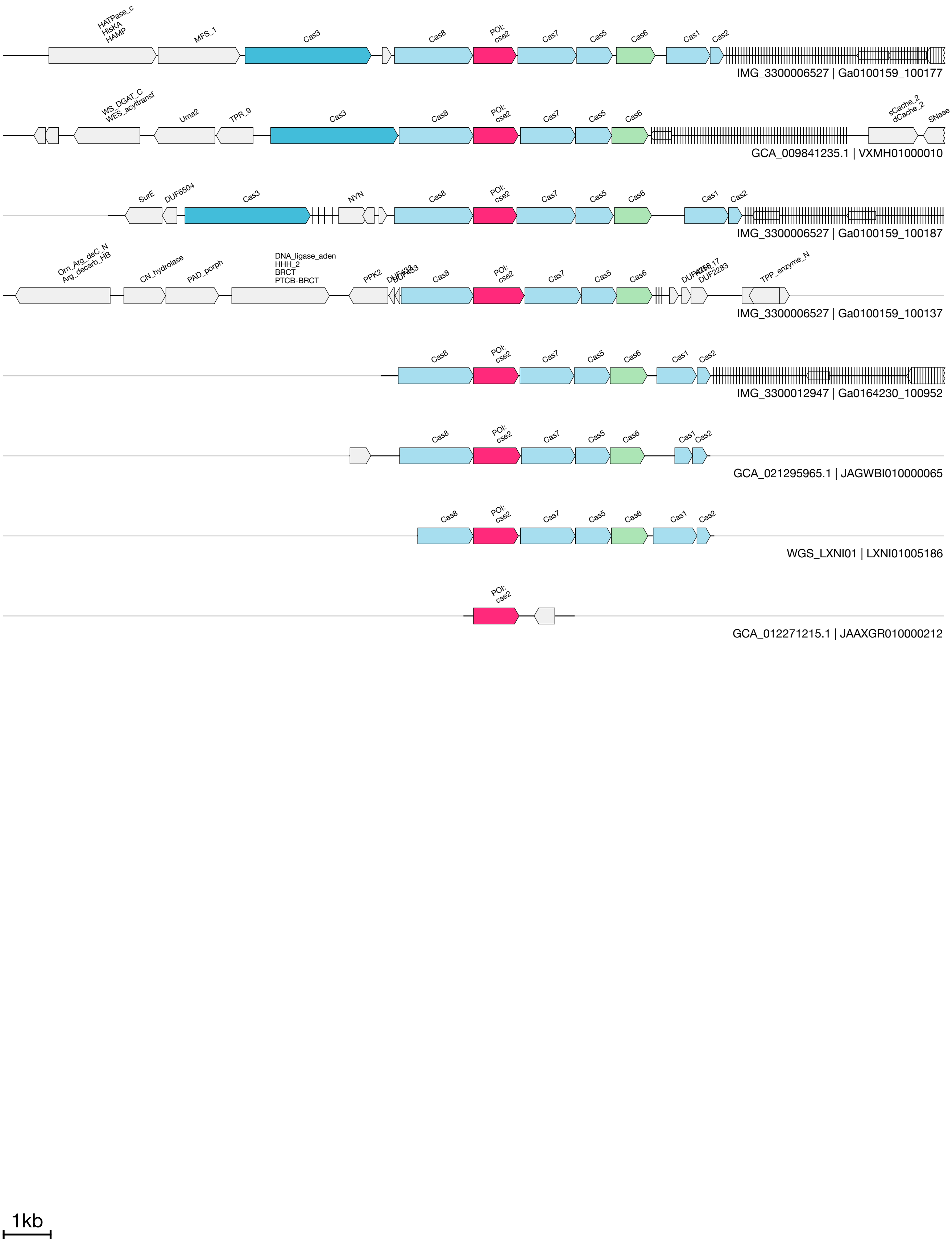

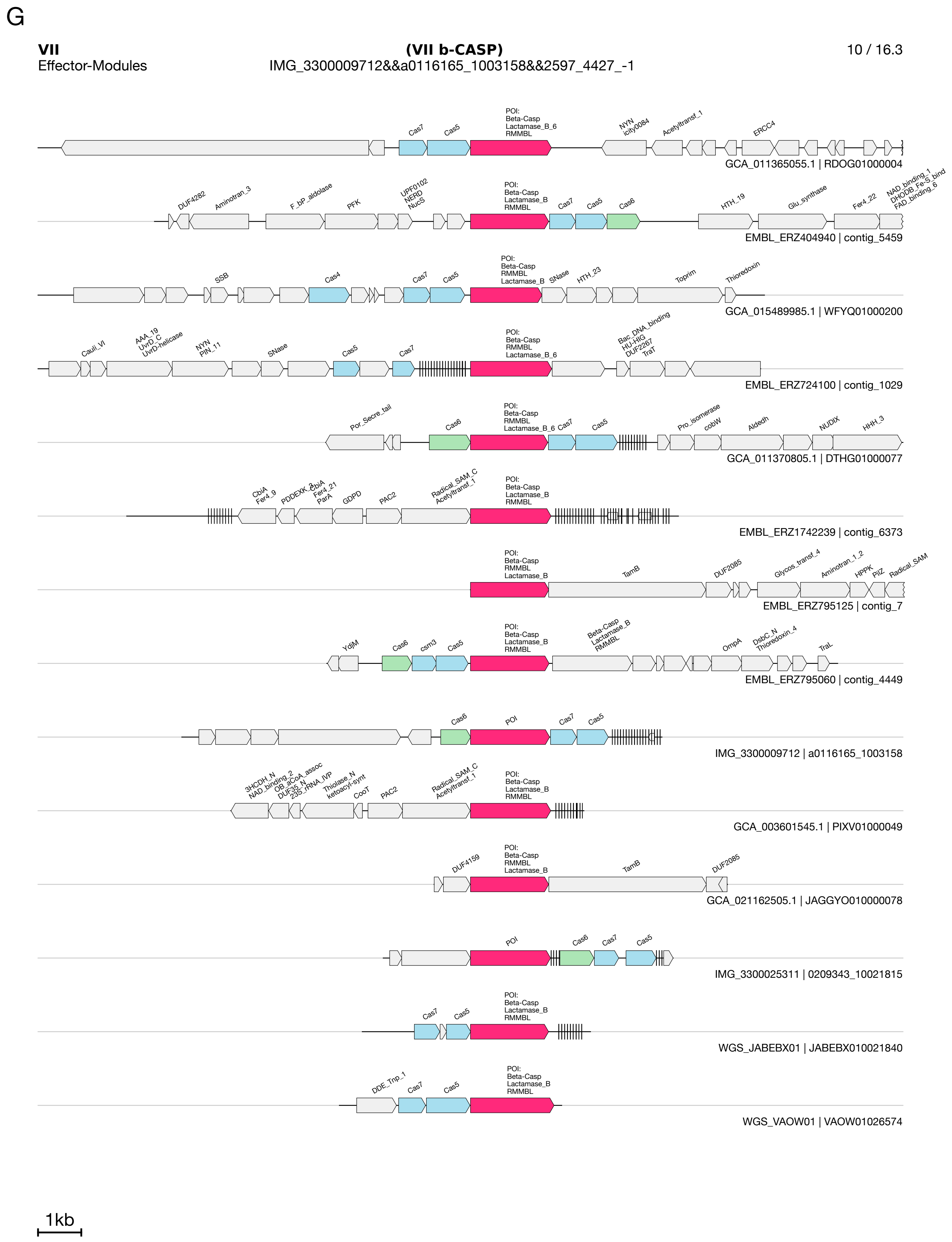
G
VII
Effector-Modules
(VII b-CASP)
IMG_3300009712&&a0116165_1003158&&2597_4427_-1
10 / 16.3
POI:
Beta-Casp
Lactamase_B_6
RMMBL
Cas7
Cas5
NYN
icity0084
Acetyltransf_1
ERCC4
GCA_011365055.1 | RDOG01000004
DUF4282
Aminotran_3
F_bP_aldolase
PFK
UPF0102
NERD
NucS
Cas6
HTH_19
Glu_synthase
Fer4_22
NAD_binding_1
DHODB_Fe-S_bind
FAD_binding_6
EMBL_ERZ404940 | contig_5459
SSB
Cas4
SNase
HTH_23
Toprim
Thioredoxin
GCA_015489985.1 | WFYQ01000200
Cauli_VI
AAA_19
UvrD_C
UvrD-helicase
NYN
PIN_11
Bac_DNA_binding
HU-HIG
DUF2267
TraT
EMBL_ERZ724100 | contig_1029
Por_Secre_tail
Pro_isomerase
cobW
Aldedh
NUDIX
HHH_3
GCA_011370805.1 | DTHG01000077
CbiA
Fer4_9
PDDEXK_2
Fer4_21
ParA
GDPD
PAC2
Radical_SAM_C
Acetyltransf_1
EMBL_ERZ1742239 | contig_6373
TamB
DUF2085
Glycos_transf_4
Aminotran_1_2
HPPK
PilZ
Radical_SAM
EMBL_ERZ795125 | contig_7
YdjM
csm3
OmpA
DsbC_N
Thioredoxin_4
TraL
EMBL_ERZ795060 | contig_4449
POI
IMG_3300009712 | a0116165_1003158
3HCDH_N
NAD_binding_2
OB_aCoA_assoc
DUF35_N
23S_rRNA_IVP
Thiolase_N
ketoacyl-synt
CooT
GCA_003601545.1 | PIXV01000049
DUF4159
GCA_021162505.1 | JAGGYO010000078
IMG_3300025311 | 0209343_10021815
WGS_JABEBX01 | JABEBX010021840
DDE_Tnp_1
WGS_VAOW01 | VAOW01026574
1kb

H

Cas5-HNH
Effector-Modules

(Cas5_HNH)
IMG_3300010327&&0116246_10008300&&2259_3435_-1

161 / 243.7

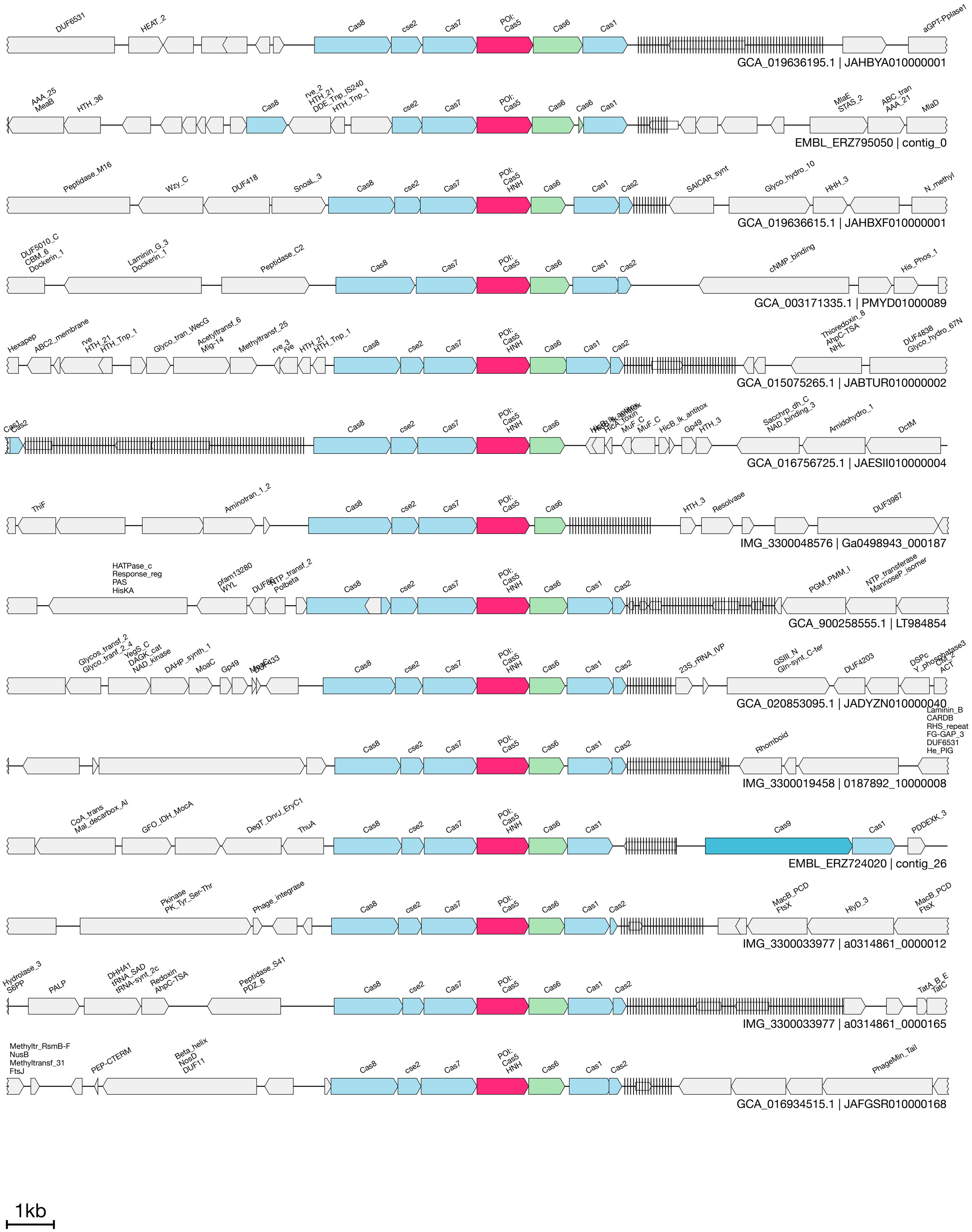

CasMu-V **(CasMuV MuB)** 2 / 4.0

Effector-Modules IMG_3300010349&&0116240_10096391&&801_1509_-1

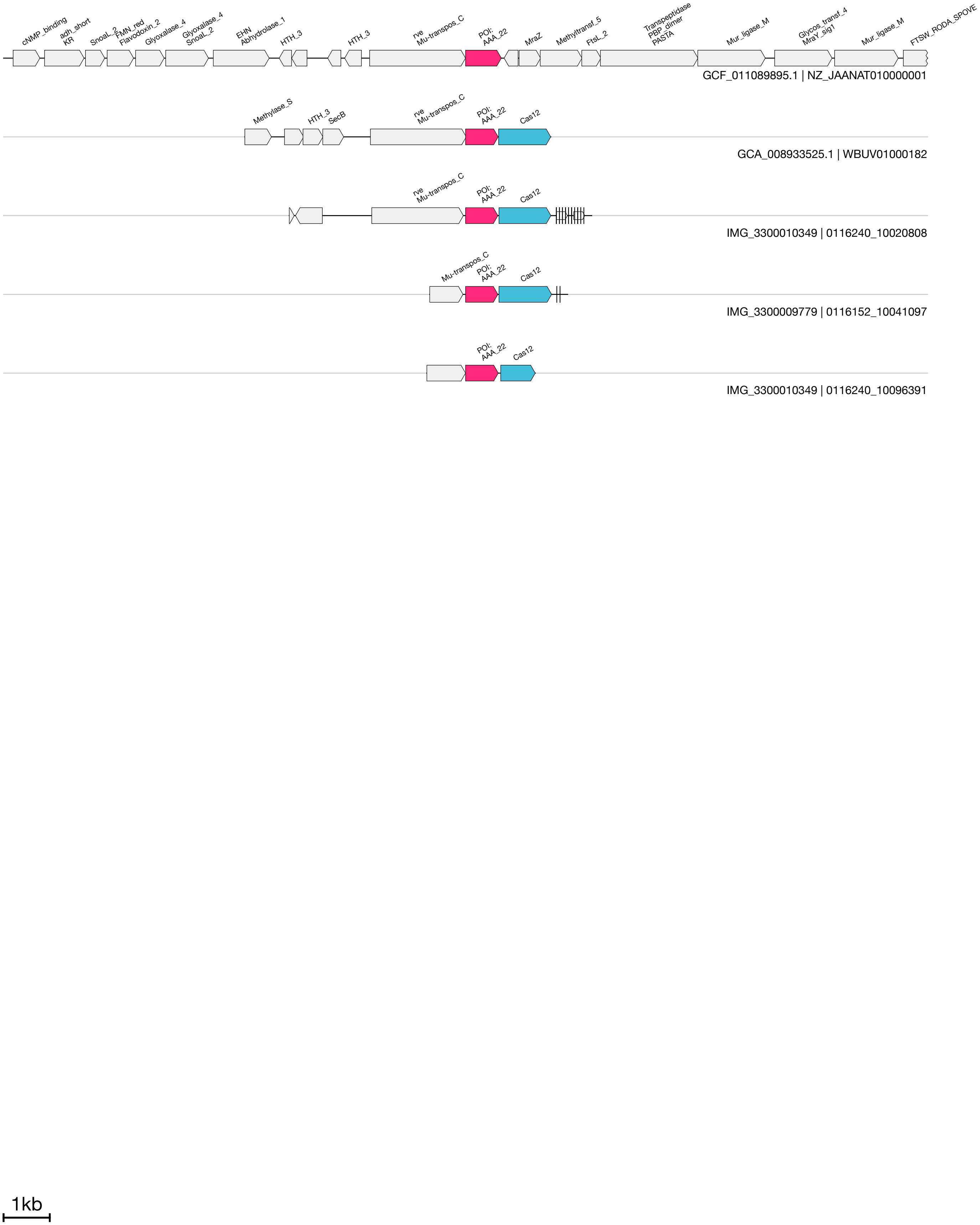

J

Cas12-Cas3 **(Cas12 + Cas3)** 7 / 8.0
Effector-Modules IMG_3300020641&&Ga0206884_1077&&6347_8576_1

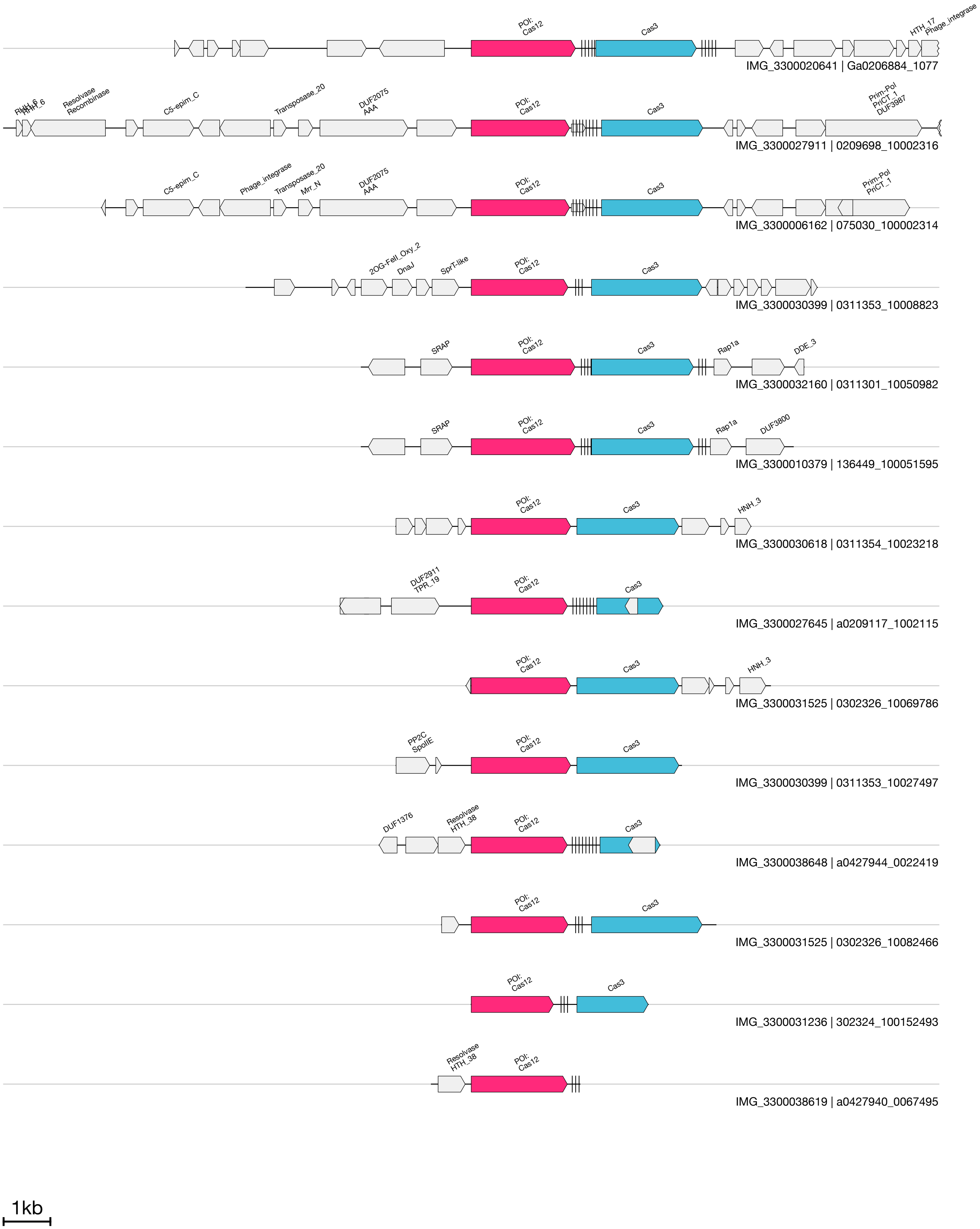

K

Cas12-IscB
Effector-Modules

(Cas12 + IscB + HTH)
IMG_3300027823&&0209490_10014851&&1048_2638_1

4 / 9.5

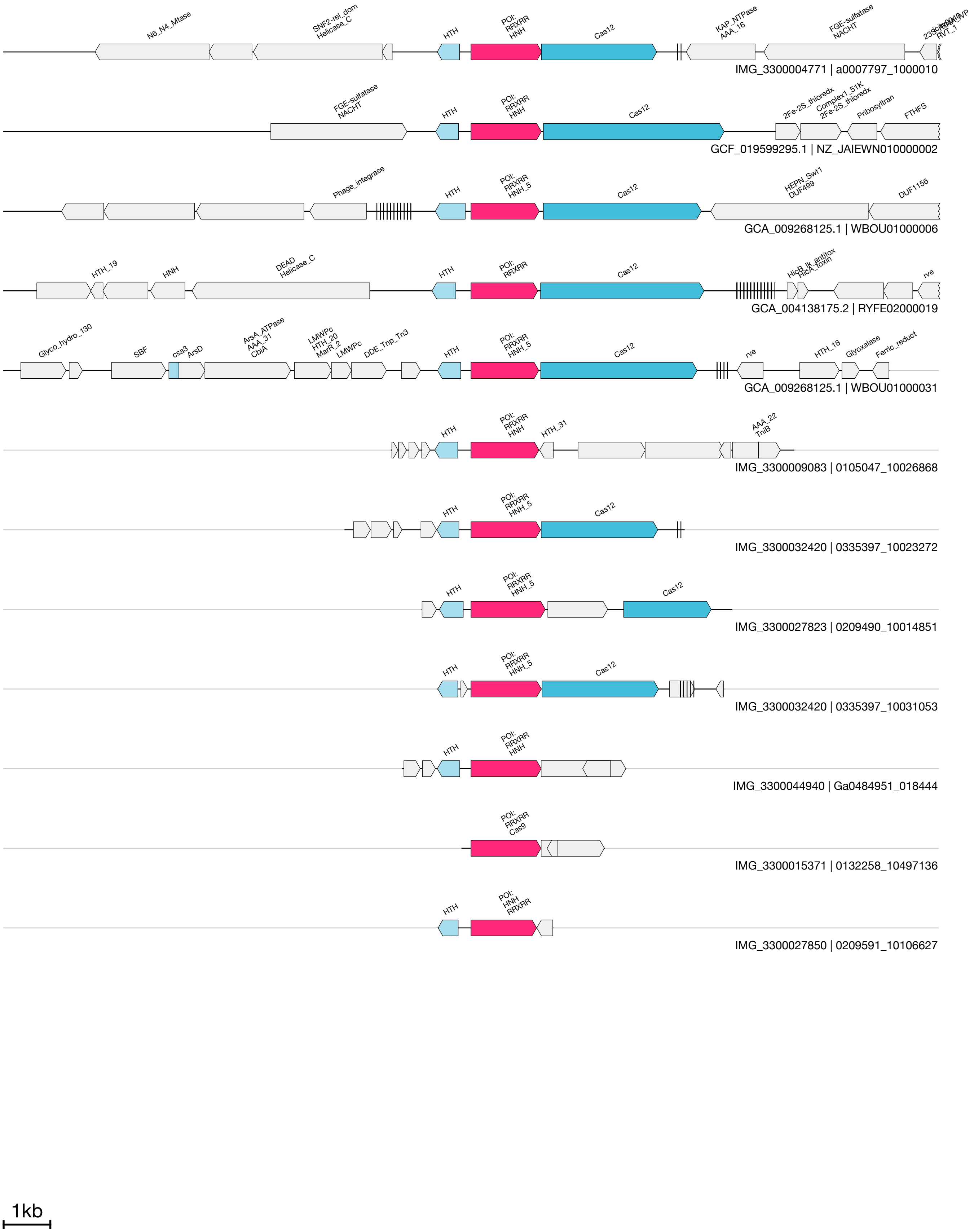

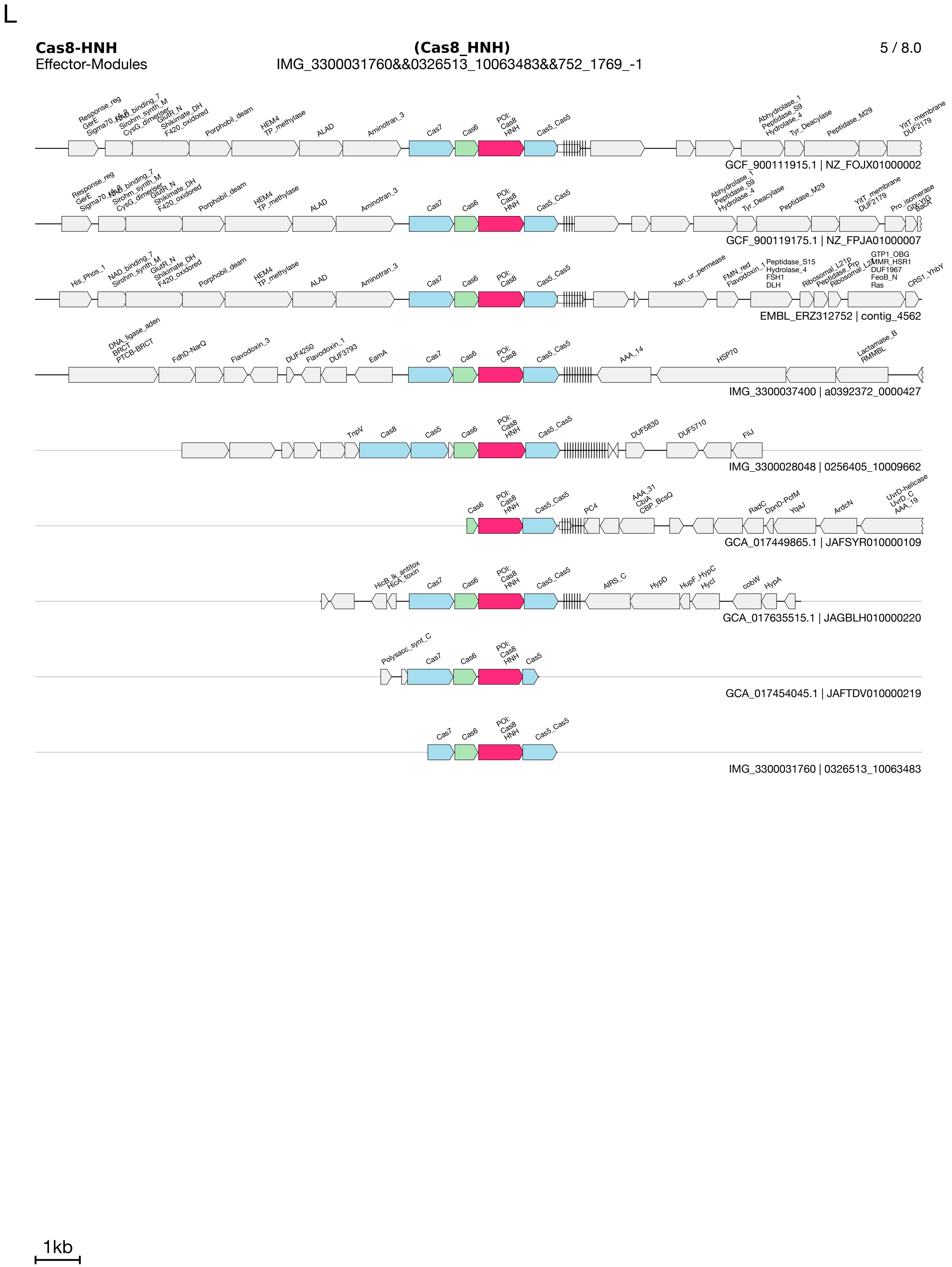
L
Cas8-HNH
Effector-Modules
(Cas8_HNH)
IMG_3300031760&&0326513_10063483&&752_1769_-1
5 / 8.0
Response_reg
GerE
Sigma70_r4_2
NAD_binding_7
Sirohm_synth_M
CysG_dimeriser
GlutR_N
Shikimate_DH
F420_oxidored
Porphobil_deam
HEM4
TP_methylase
ALAD
Aminotran_3
Cas7
Cas6
POI:
Cas8
HNH
Cas5_Cas5
Abhydrolase_1
Peptidase_S9
Hydrolase_4
Tyr_Deacylase
Peptidase_M29
YitT_membrane
DUF2179
GCF_900111915.1 | NZ_FOJX01000002
Pro_isomerase
Gly-YIG
BacA
GCF_900119175.1 | NZ_FPJA01000007
His_Phos_1
Xan_ur_permease
FMN_red
Flavodoxin_1
Peptidase_S15
Hydrolase_4
FSH1
DLH
Ribosomal_L21p
Peptidase_Prp
Ribosomal_L27
GTP1_OBG
MMR_HSR1
DUF1967
FeoB_N
Ras
CRS1_YhbY
EMBL_ERZ312752 | contig_4562
DNA_ligase_aden
BRCT
PTCB-BRCT
FdhD-NarQ
Flavodoxin_3
DUF4250
Flavodoxin_1
DUF3793
EamA
AAA_14
HSP70
Lactamase_B
RMMBL
IMG_3300037400 | a0392372_0000427
TnpV
Cas8
Cas5
DUF5830
DUF5710
FliJ
IMG_3300028048 | 0256405_10009662
PC4
AAA_31
CbiA
CBP_BcsQ
RadC
DpnD-PcfM
YqaJ
ArdcN
UvrD-helicase
UvrD_C
AAA_19
GCA_017449865.1 | JAFSYR010000109
HicB_lk_antitox
HicA_toxin
AIRS_C
HypD
HupF_HypC
HycI
cobW
HypA
GCA_017635515.1 | JAGBLH010000220
Polysacc_synt_C
Cas5
GCA_017454045.1 | JAFTDV010000219
IMG_3300031760 | 0326513_10063483
1kb

M

DinG-HNH
Effector-Modules

(DinG_HNH)
IMG_3300044617&&a0453743_0023453&&2386_4123_-1

111 / 129.0

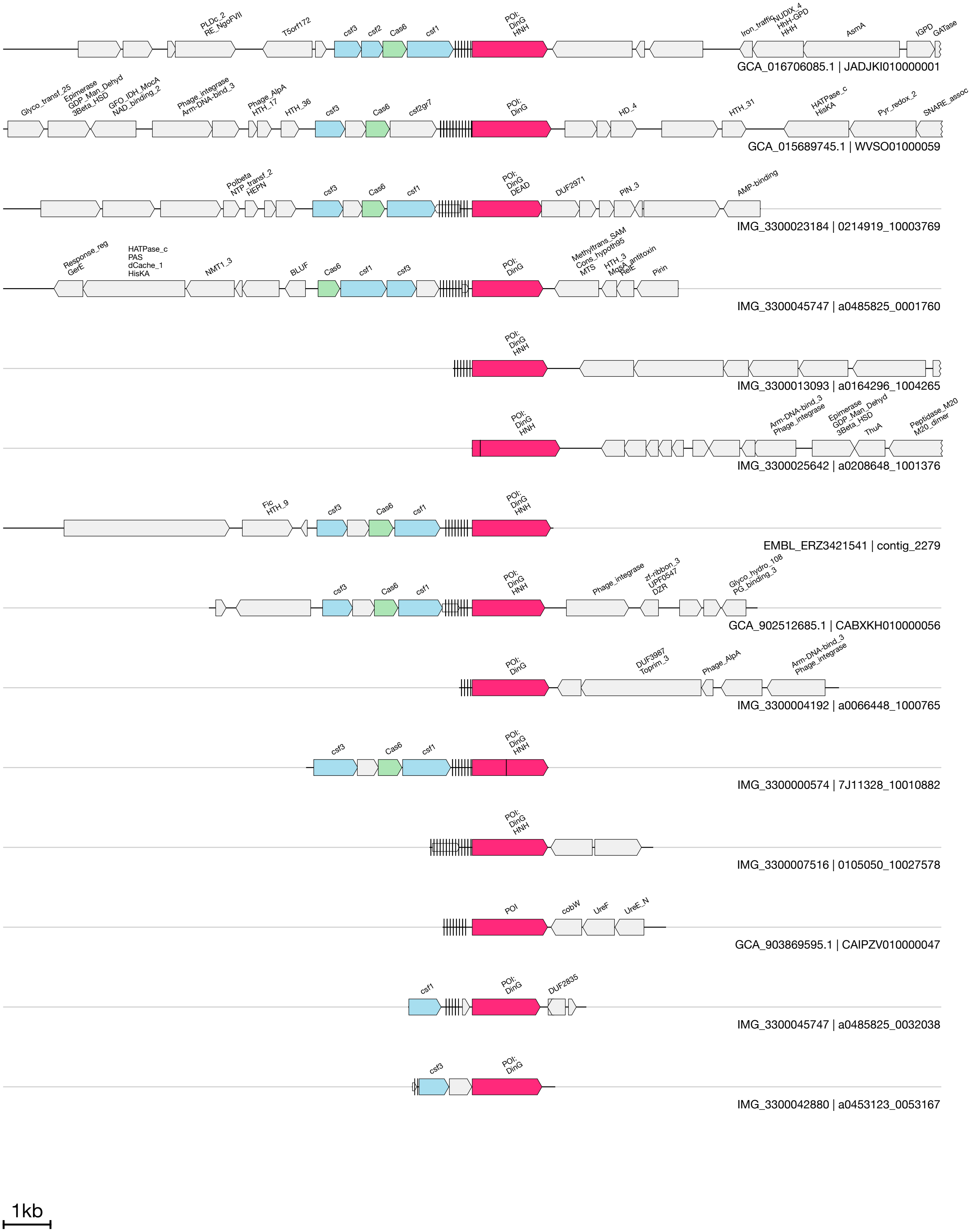

N

Cas8-Cas5
Cas-Fusions

(Cas7 + Cas8_Cas5 fusion)
EMBL_ERZ505329&&contig_129&&28461_30633_-1

3 / 4.7

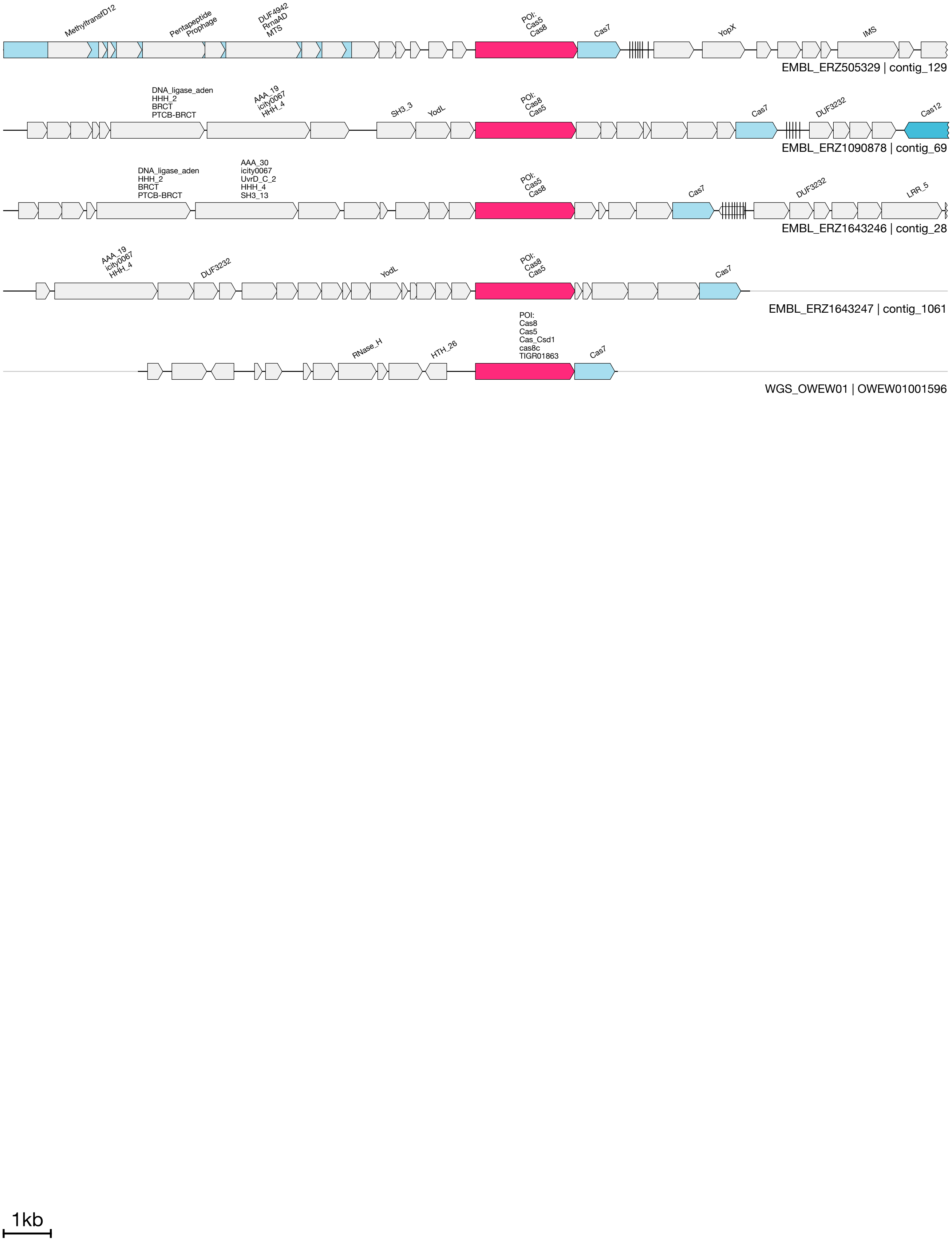

O

UAS-37 **(Cas9_Cas1 fusion)** 3 / 3.0

Cas-Fusions GCA_904380115.1&&CAJFHU010000034&&10263_14391_-1

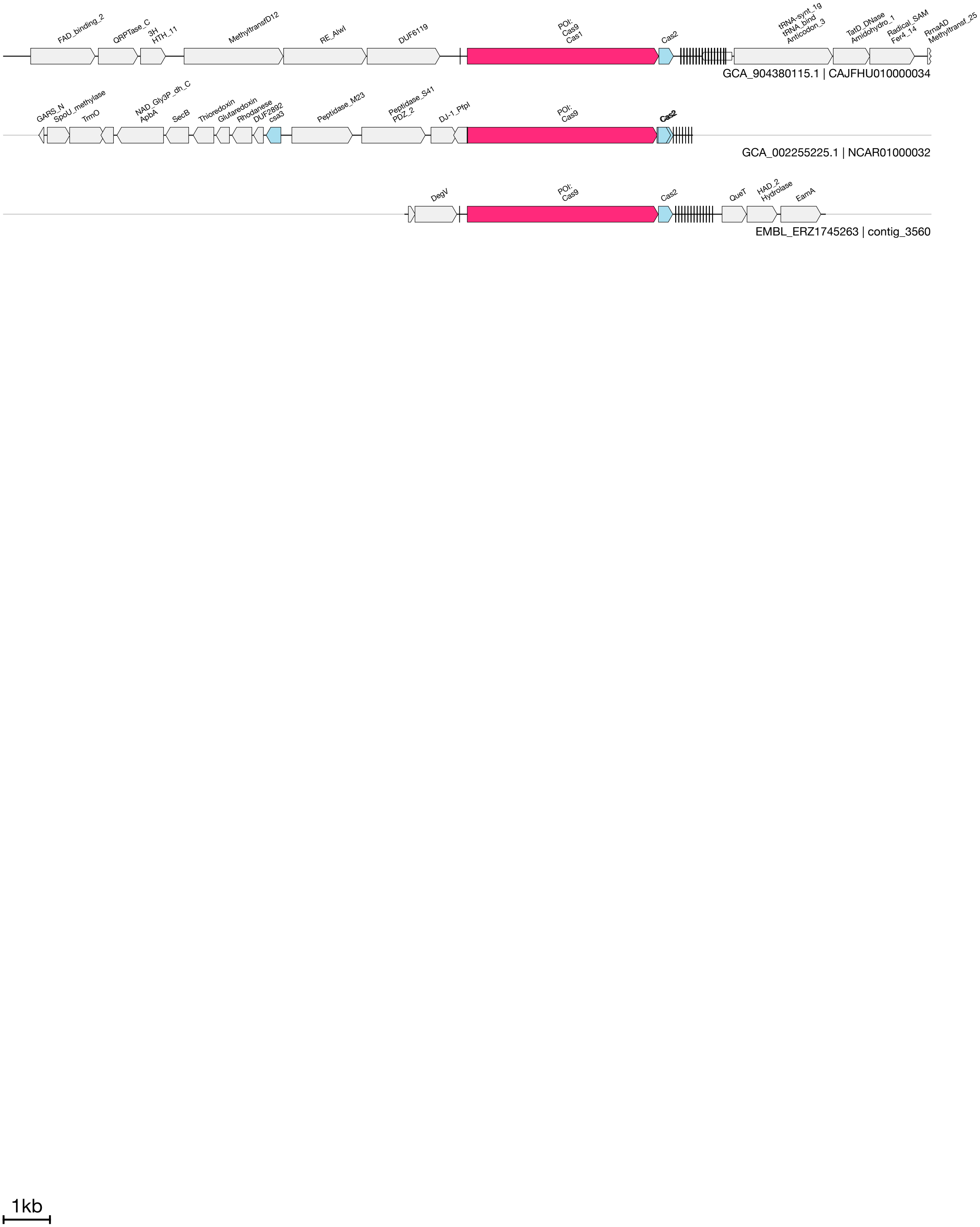

P

Cas5-Cas3 **(Cas5_Cas3 fusion)** 27 / 27.7

Cas-Fusions GCF_000058485.1&&NC_008278&&6031418_6034760_1

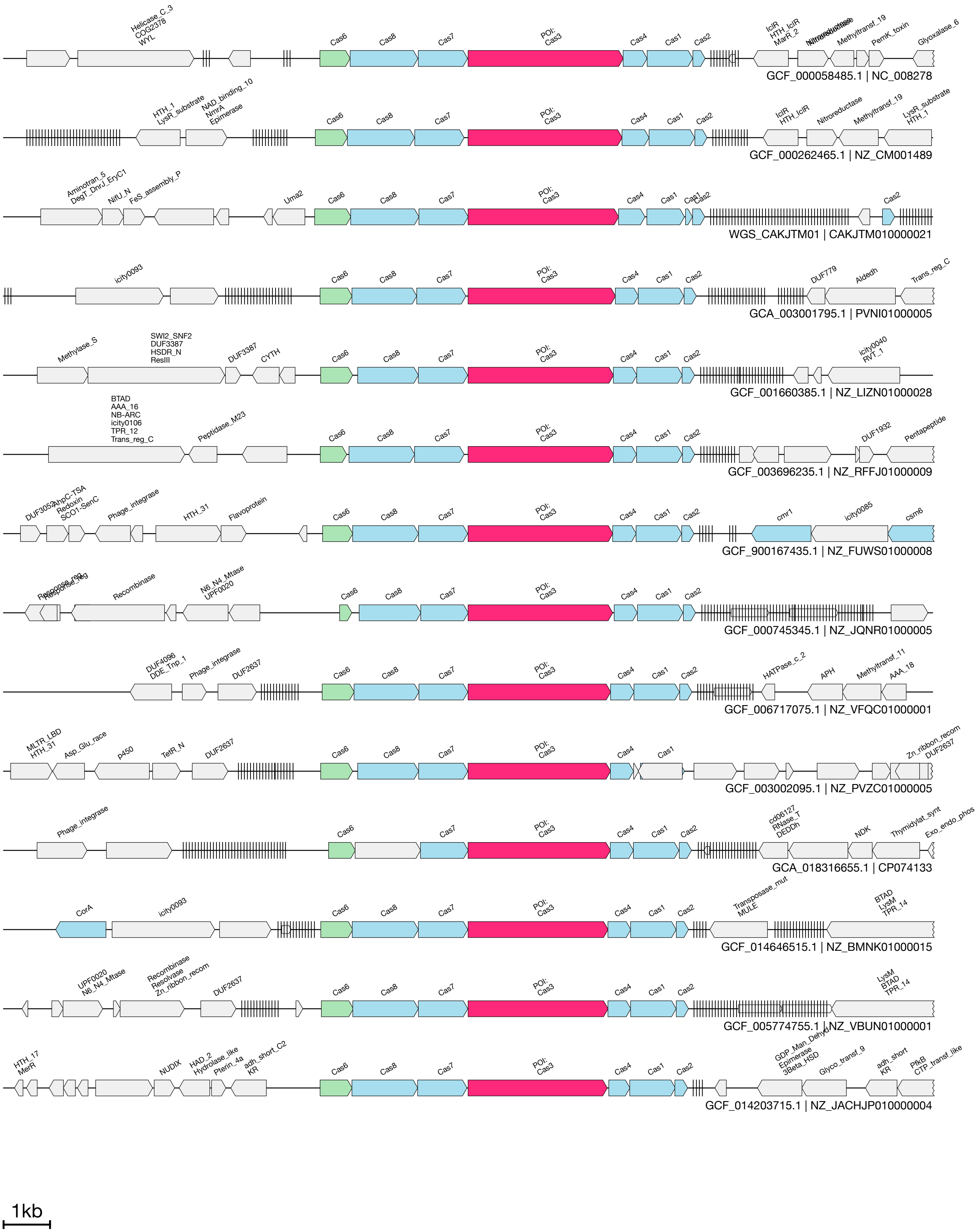

Q

UAS-38 **(Cmr6_Cmr1 fusion)** 24 / 37.1

Cas-Fusions GCF_021018745.1&&NZ_CP063845&&4894452_4896432_-1

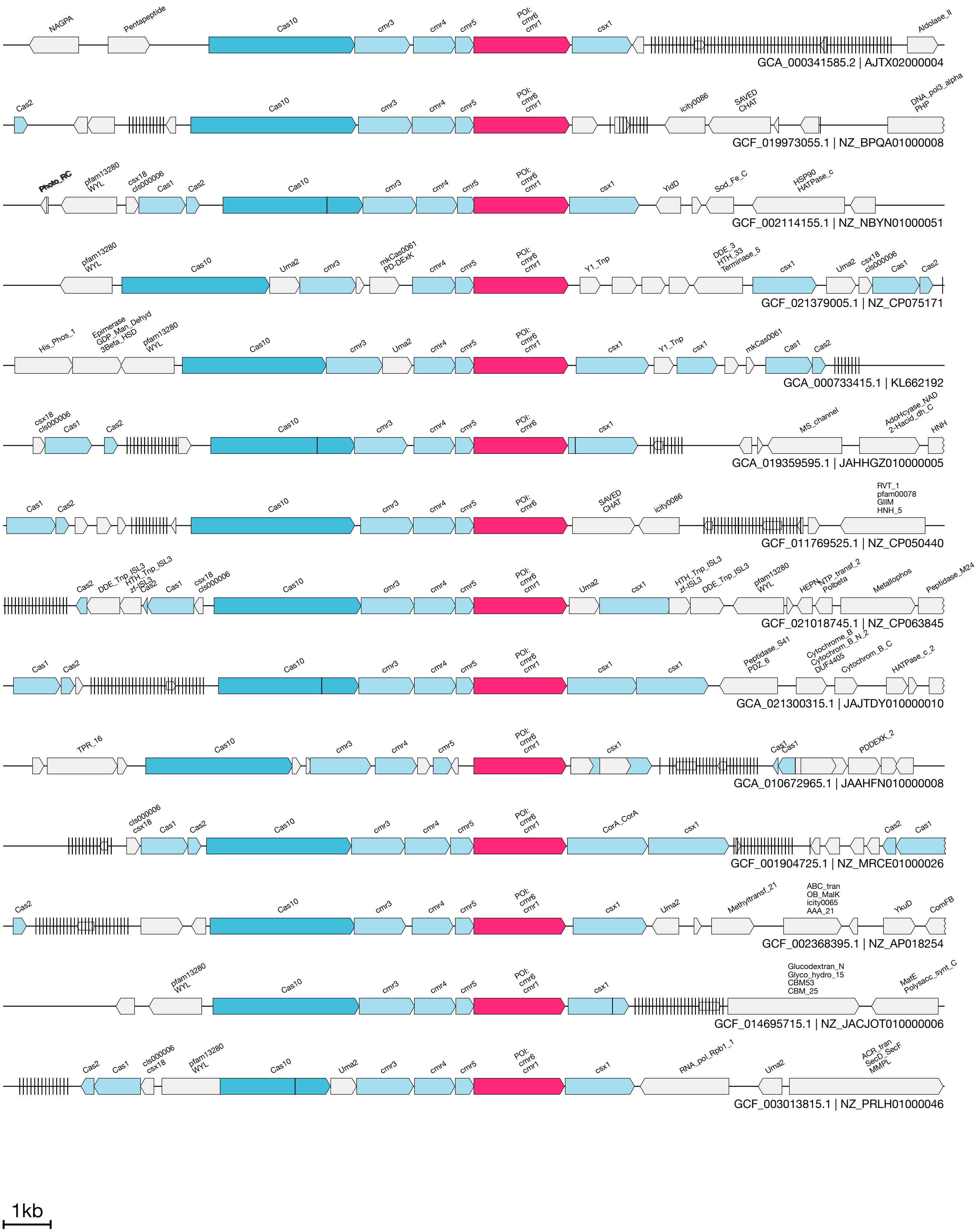

R

UAS-39
Cas-Fusions

(Cas6_Cas5_fusion)
IMG_3300001592&&Draft_10000135&&23396_24782_1

4 / 4.0

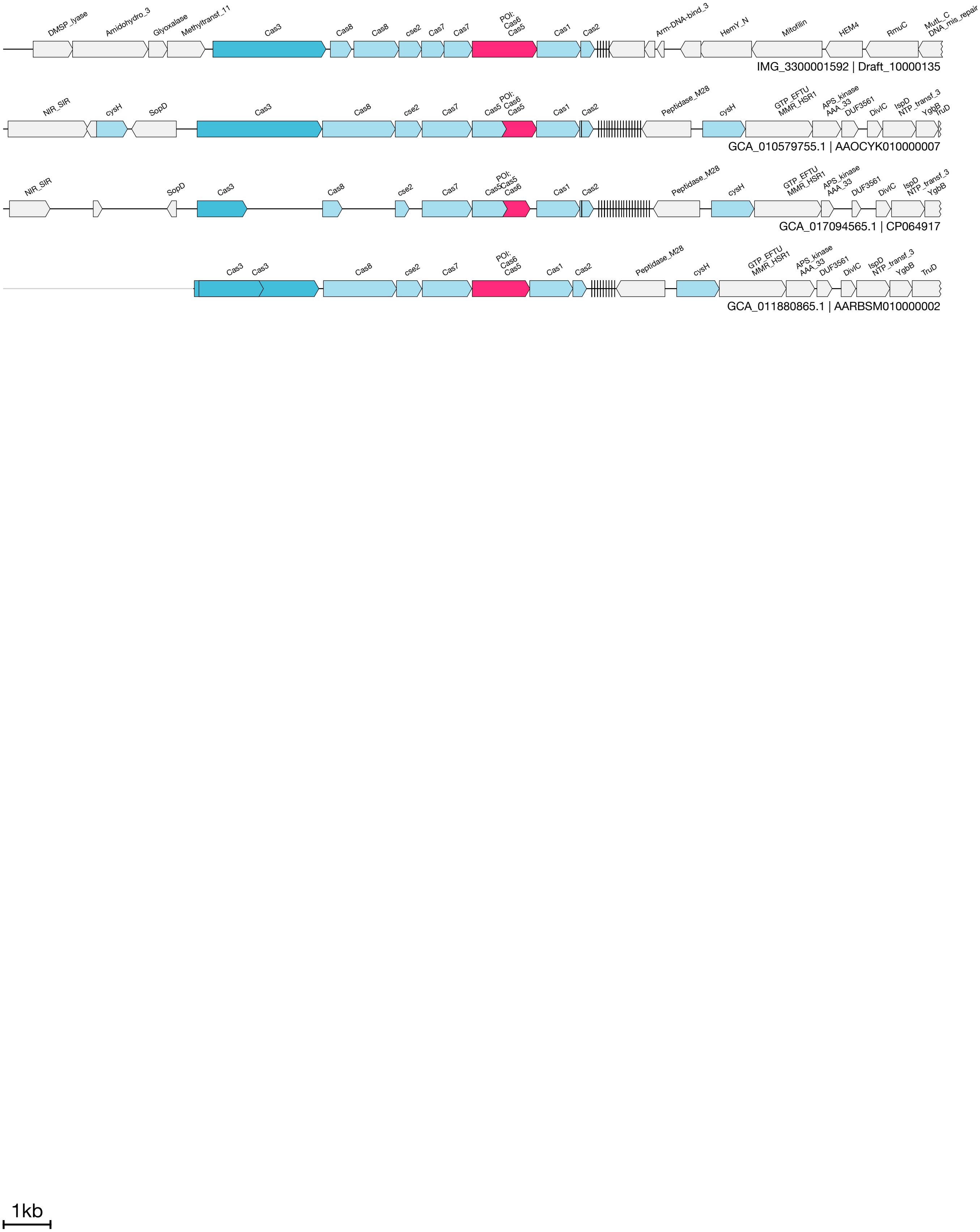

S

UAS-40 **(Csa5_Csa4 fusion)** 4 / 6.1

Cas-Fusions IMG_3300021472&&a0190363_1001919&&8327_9779_1

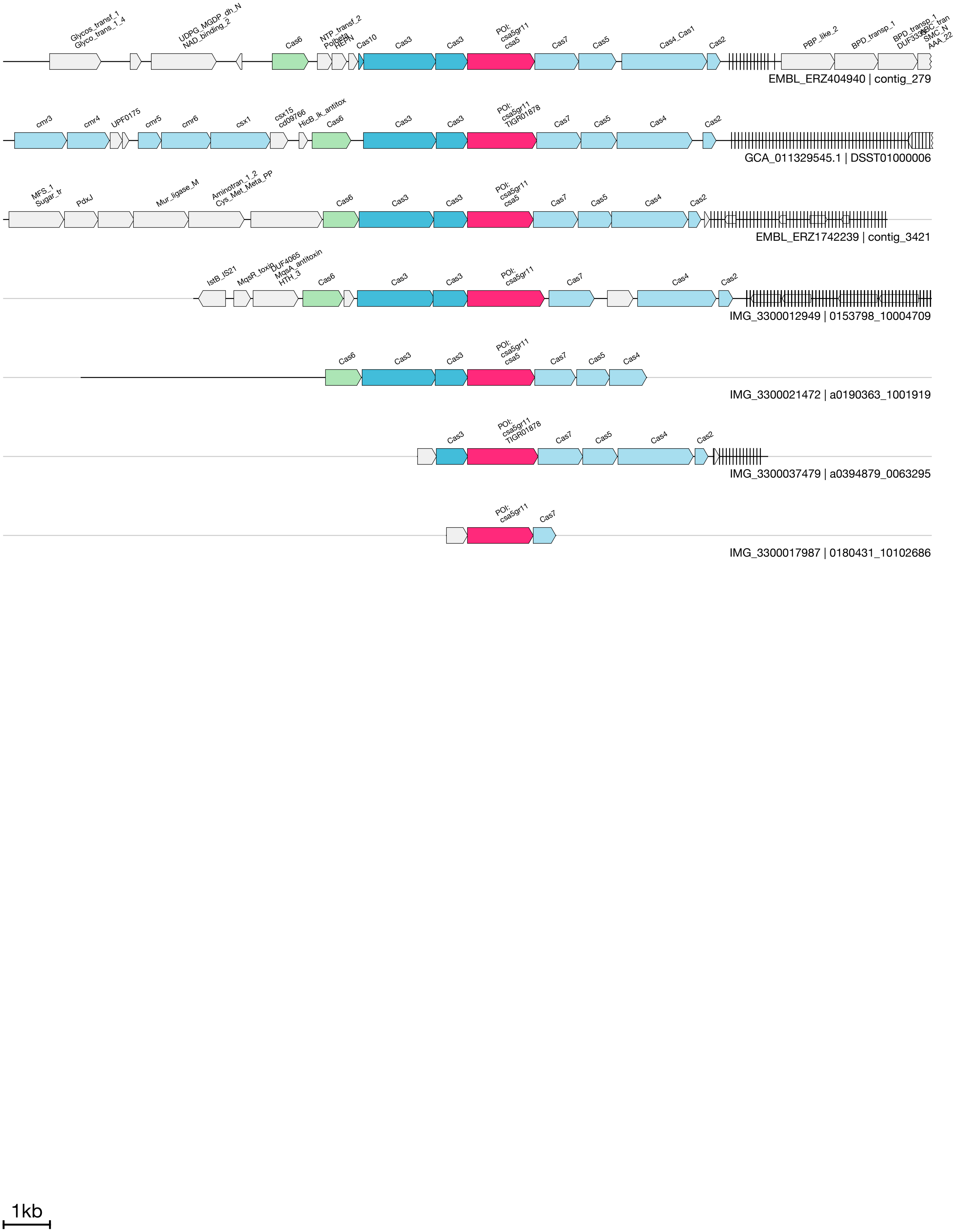

T

UAS-41 **(Cas8_Cas5 fusion)** 4 / 4.0

Cas-Fusions WGS_JABLTL01&&JABLTL010073043&&184_2911_1

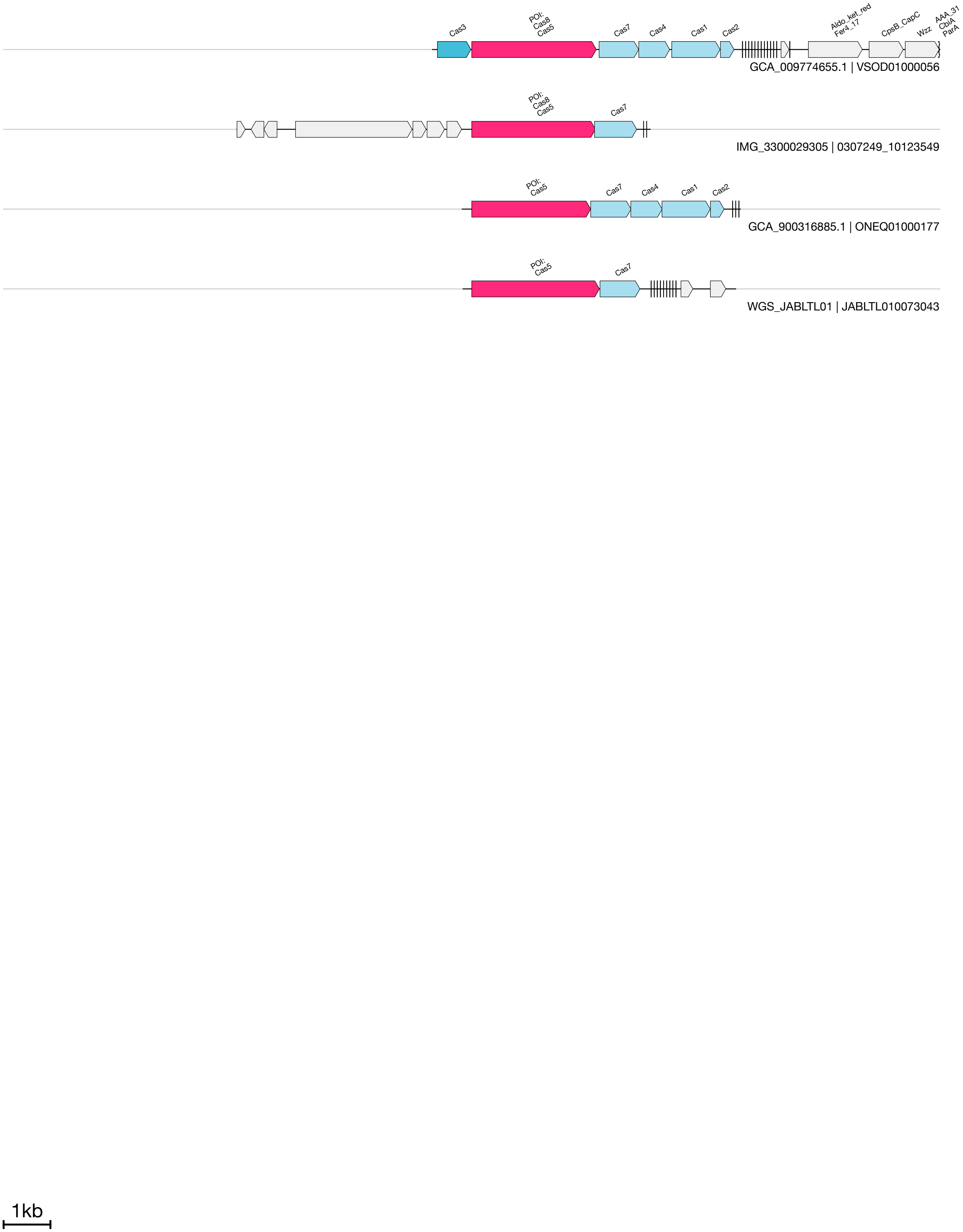

U

UAS-42 **(DUF3761)** 22 / 22.7

Acquisition EMBL_ERZ724514&&contig_3192&&343_1033_-1

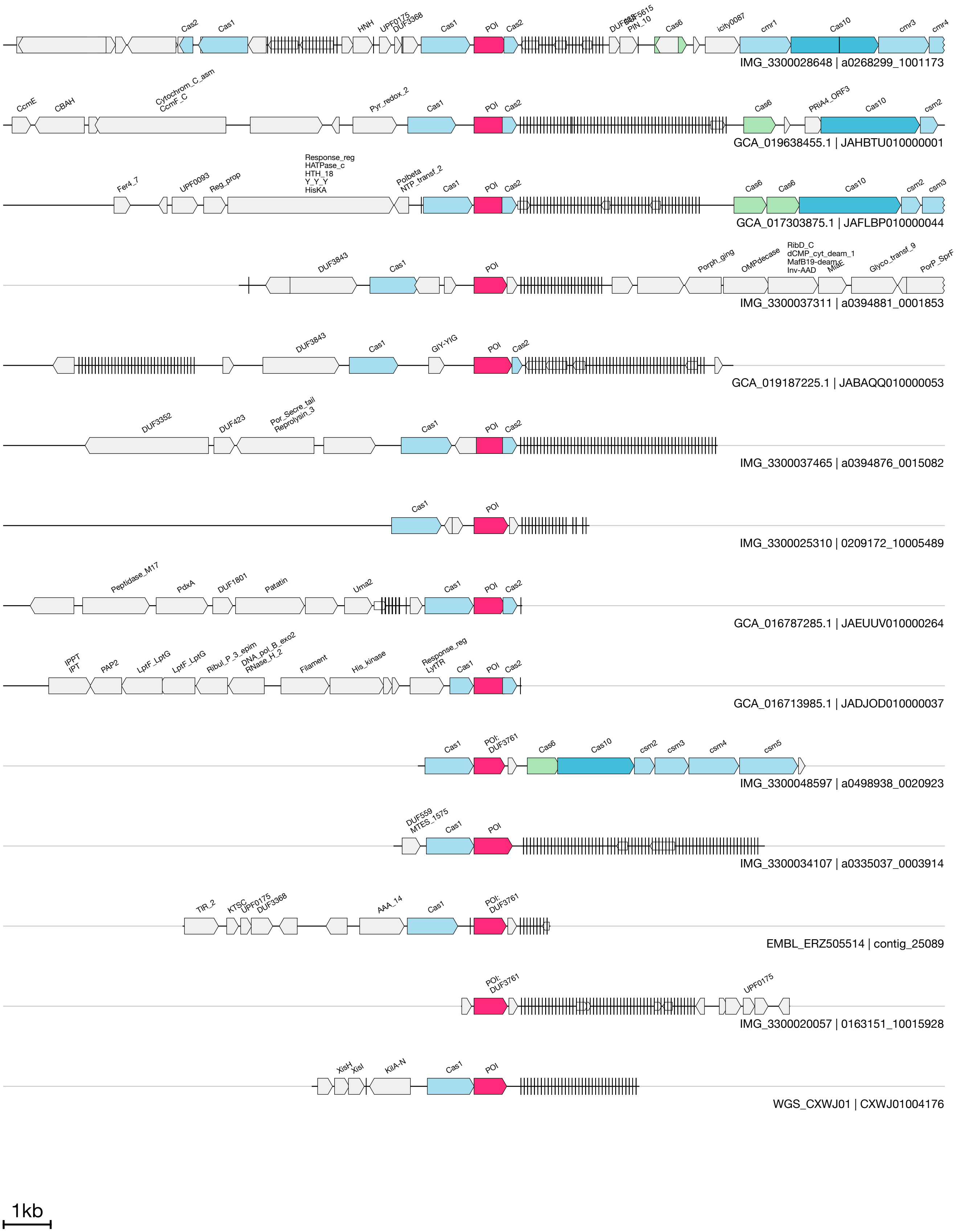

V

UAS-43 **(RVT + group II intron PrimPol)** 14 / 15.7

Acquisition GCA_002358875.1&&DEPS01000038&&15824_19211_-1

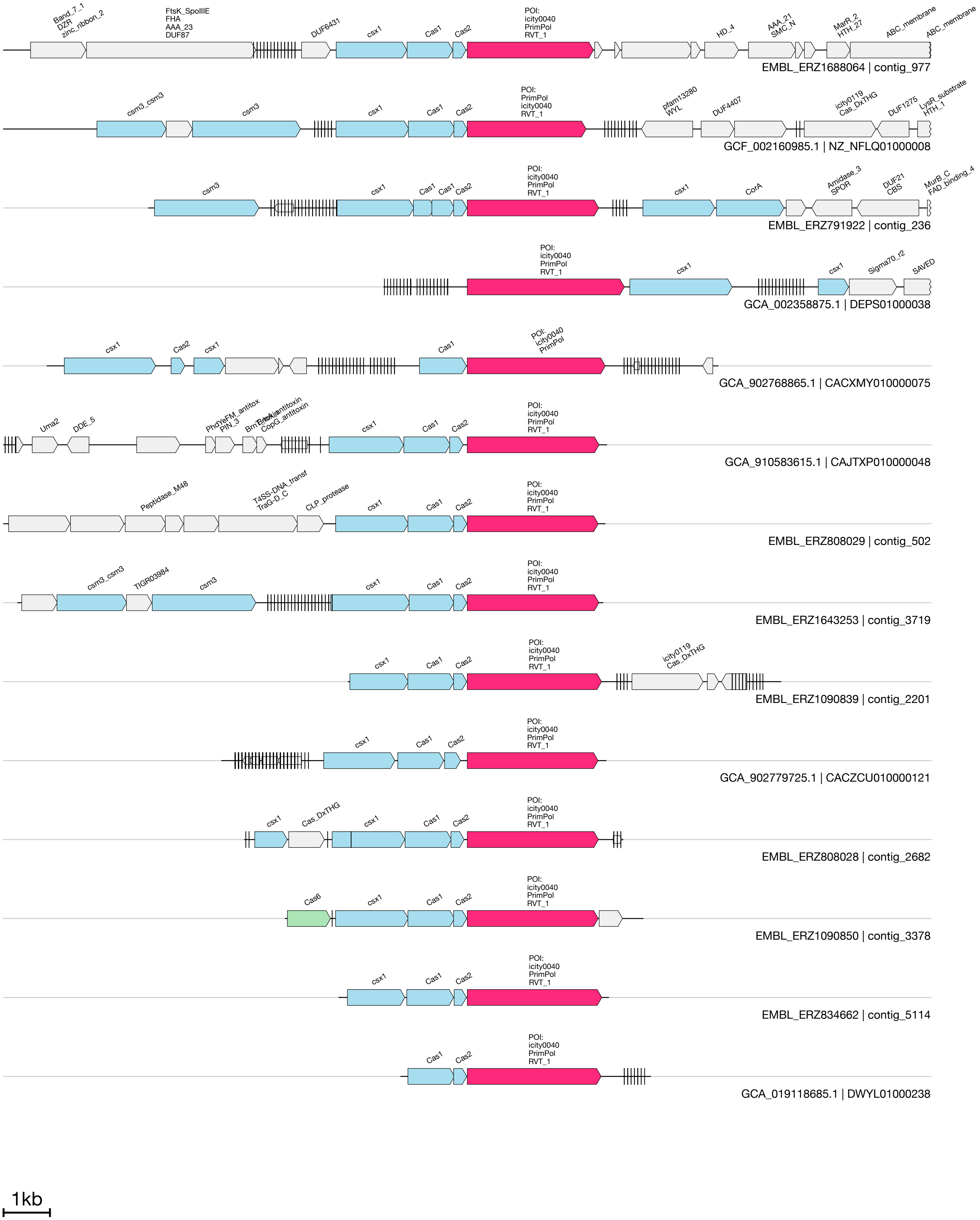

W

UAS-3 **(DUF370)** 2 / 3.5

Acquisition GCA_013359445.1&&JABWBV010000281&&2844_3129_-1

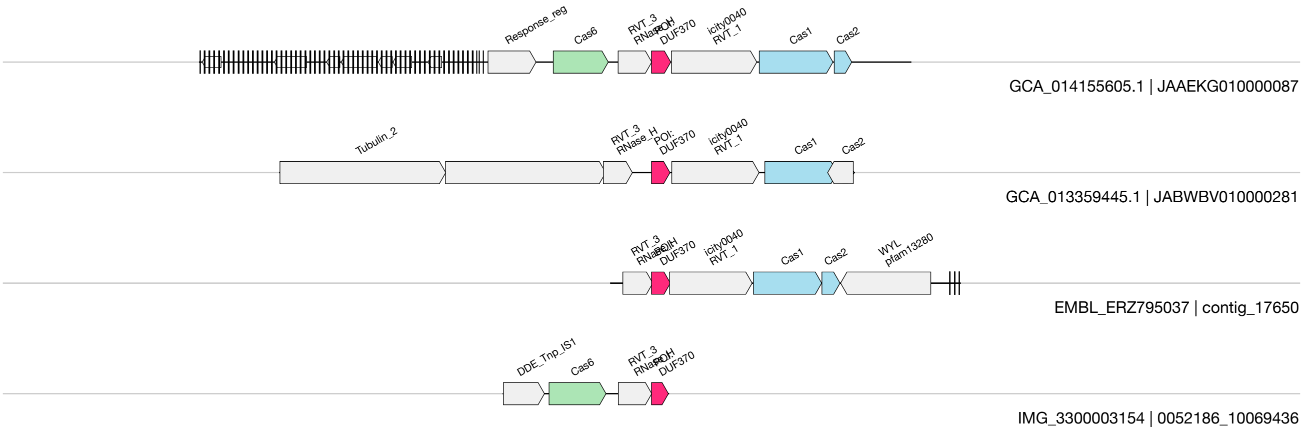

1kb

X

UAS-7 **(vWA + RDD)** 2 / 4.7

Acquisition GCA_016179065.1&&JACOSD010000405&&5712_7455_-1

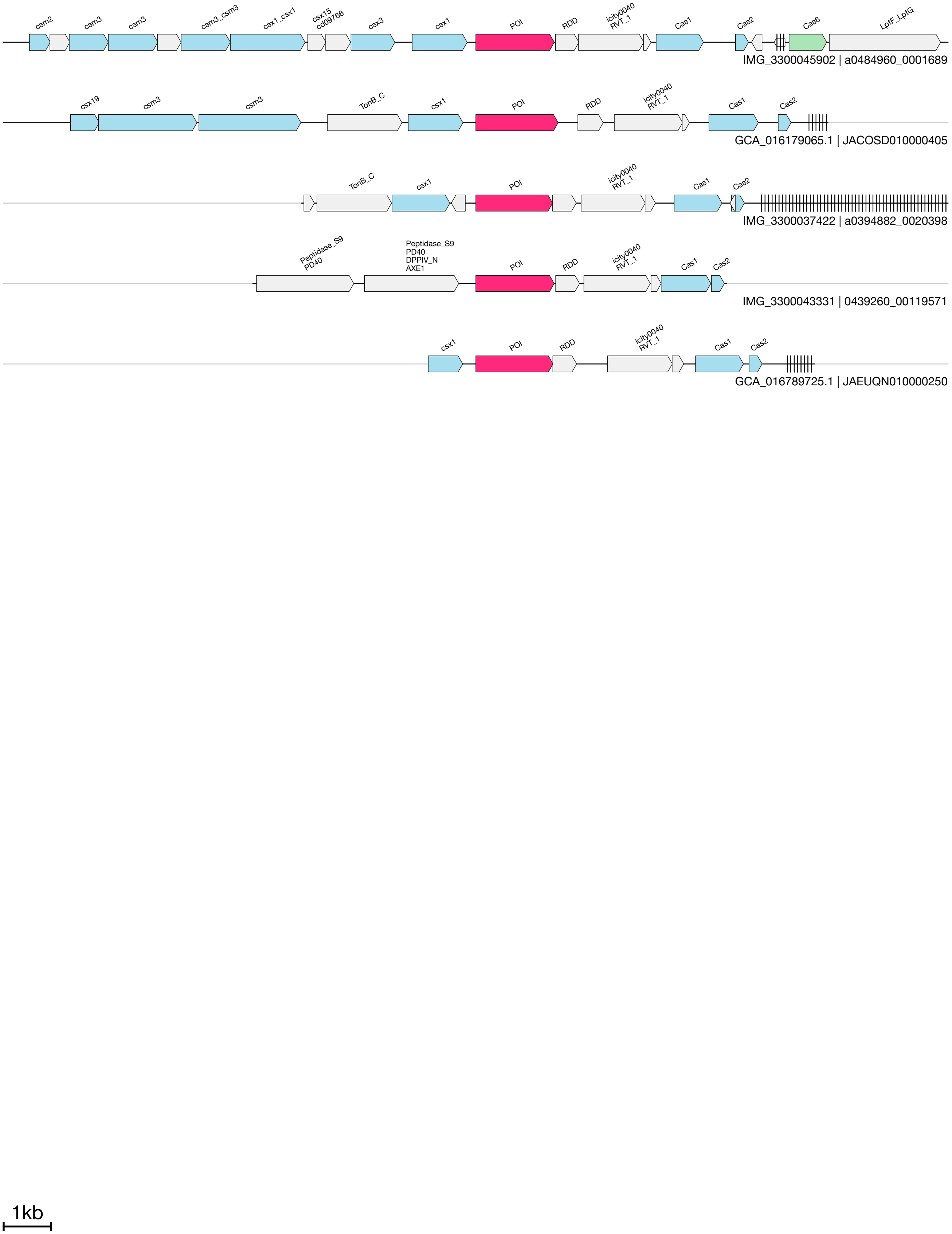

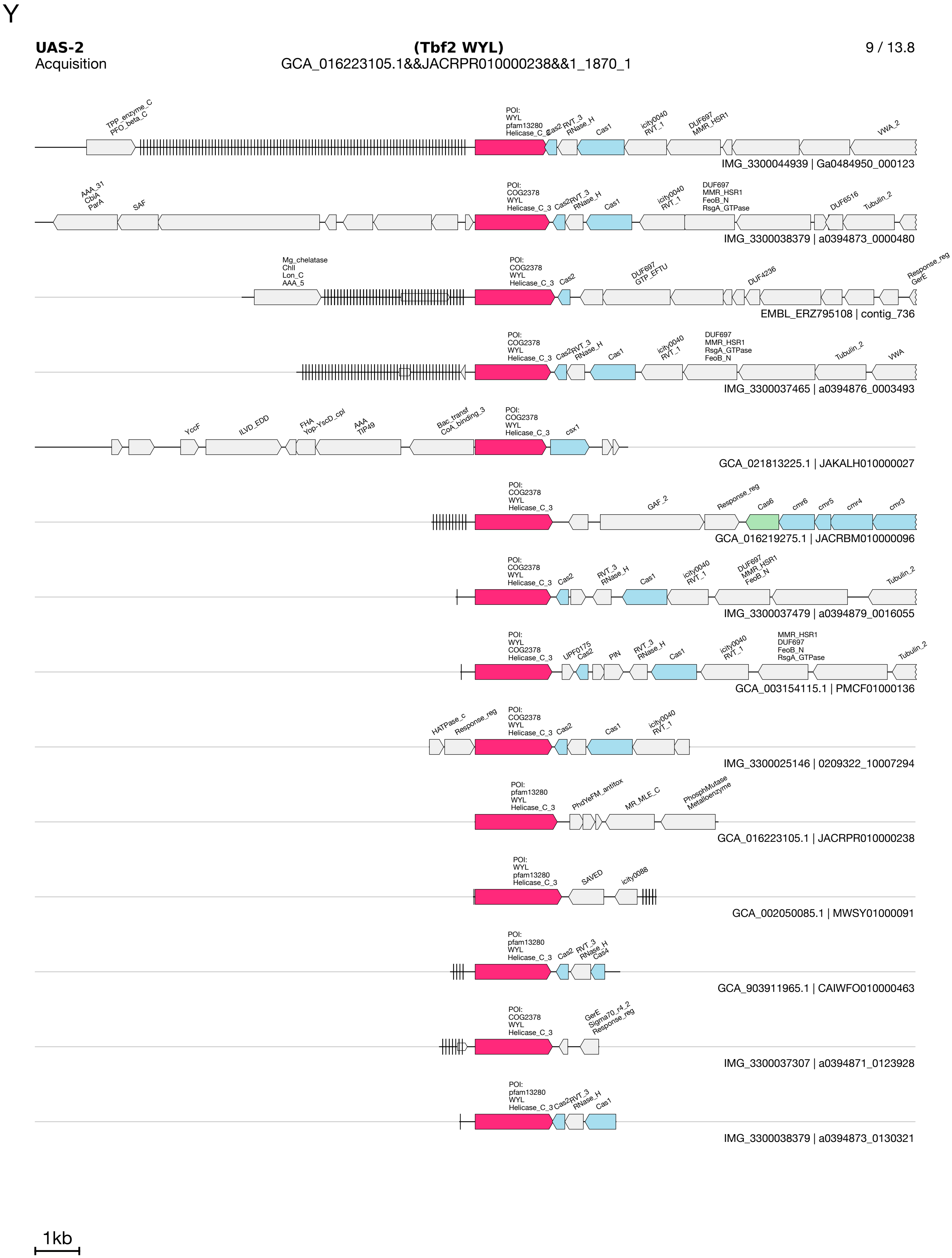
Y
UAS-2
Acquisition
(Tbf2 WYL)
GCA_016223105.1&&JACRPR010000238&&1_1870_1
9 / 13.8
IMG_3300044939 | Ga0484950_000123
IMG_3300038379 | a0394873_0000480
EMBL_ERZ795108 | contig_736
IMG_3300037465 | a0394876_0003493
GCA_021813225.1 | JAKALH010000027
GCA_016219275.1 | JACRBM010000096
IMG_3300037479 | a0394879_0016055
GCA_003154115.1 | PMCF01000136
IMG_3300025146 | 0209322_10007294
GCA_016223105.1 | JACRPR010000238
GCA_002050085.1 | MWSY01000091
GCA_903911965.1 | CAIWFO010000463
IMG_3300037307 | a0394871_0123928
IMG_3300038379 | a0394873_0130321
POI:
WYL
pfam13280
COG2378
Helicase_C_3
Cas1
Cas2
Cas4
Cas6
RVT_3
RNase_H
RVT_1
icity0040
icity0088
DUF697
MMR_HSR1
FeoB_N
RsgA_GTPase
Tubulin_2
VWA
VWA_2
TPP_enzyme_C
PFO_beta_C
AAA_31
CbiA
ParA
SAF
DUF6516
Mg_chelatase
ChlI
Lon_C
AAA_5
GTP_EFTU
DUF4236
Response_reg
GerE
YccF
ILVD_EDD
FHA
Yop-YscD_cpl
AAA
TIP49
Bac_transf
CoA_binding_3
csx1
GAF_2
cmr6
cmr5
cmr4
cmr3
UPF0175
PIN
HATPase_c
PhdYeFM_antitox
MR_MLE_C
PhosphMutase
Metalloenzyme
SAVED
Sigma70_r4_2
1kb

Z

UAS-44
Acquisition

(DUF2797 large zinc finger protein)
GCA_016840085.1&&JAEOTA010000034&&18467_19766_-1

8 / 9.2

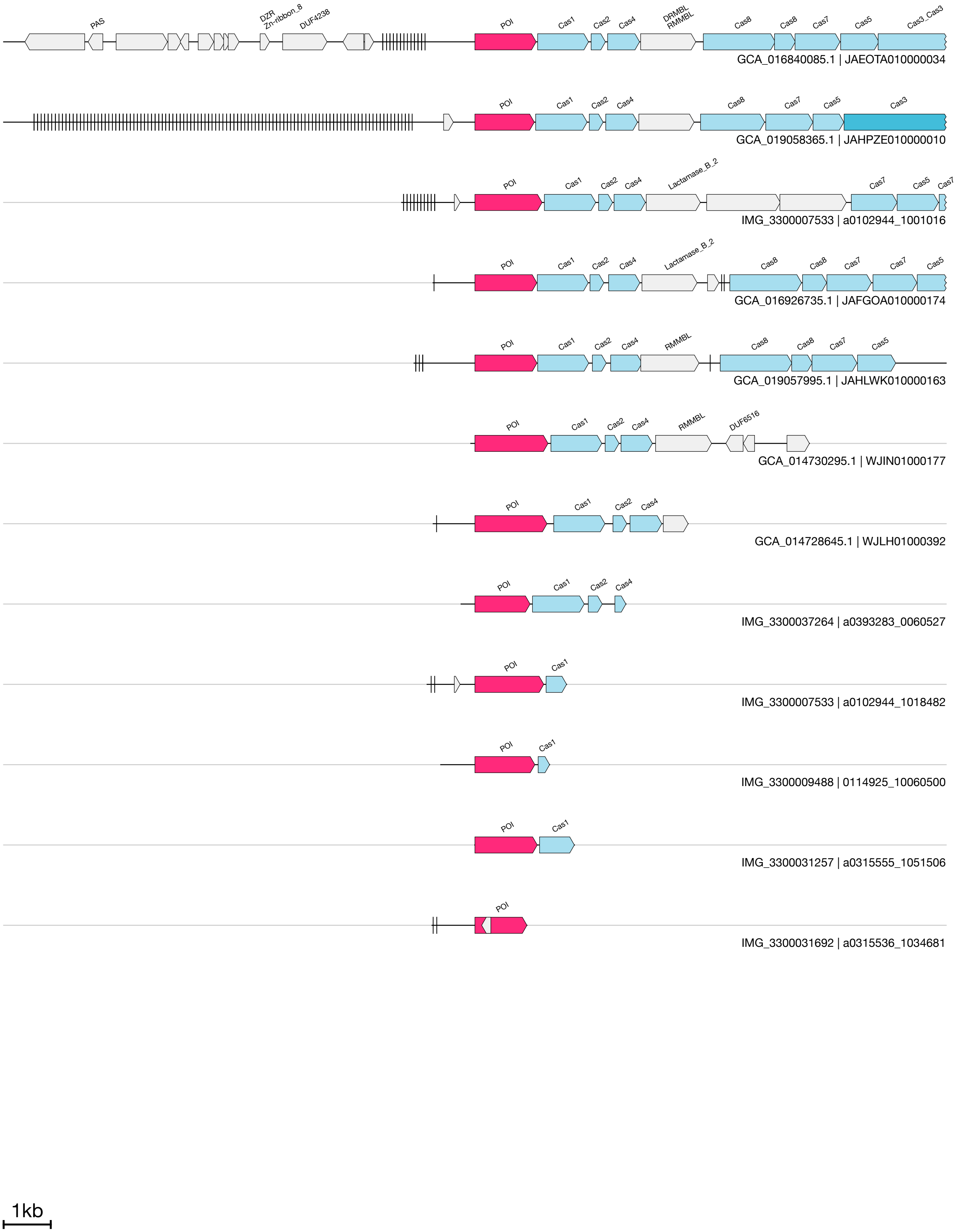

AA

UAS-45 **(RNaseH)** 11 / 21.5

Acquisition GCA_016876015.1&&VGOD01000184&&7922_8489_1

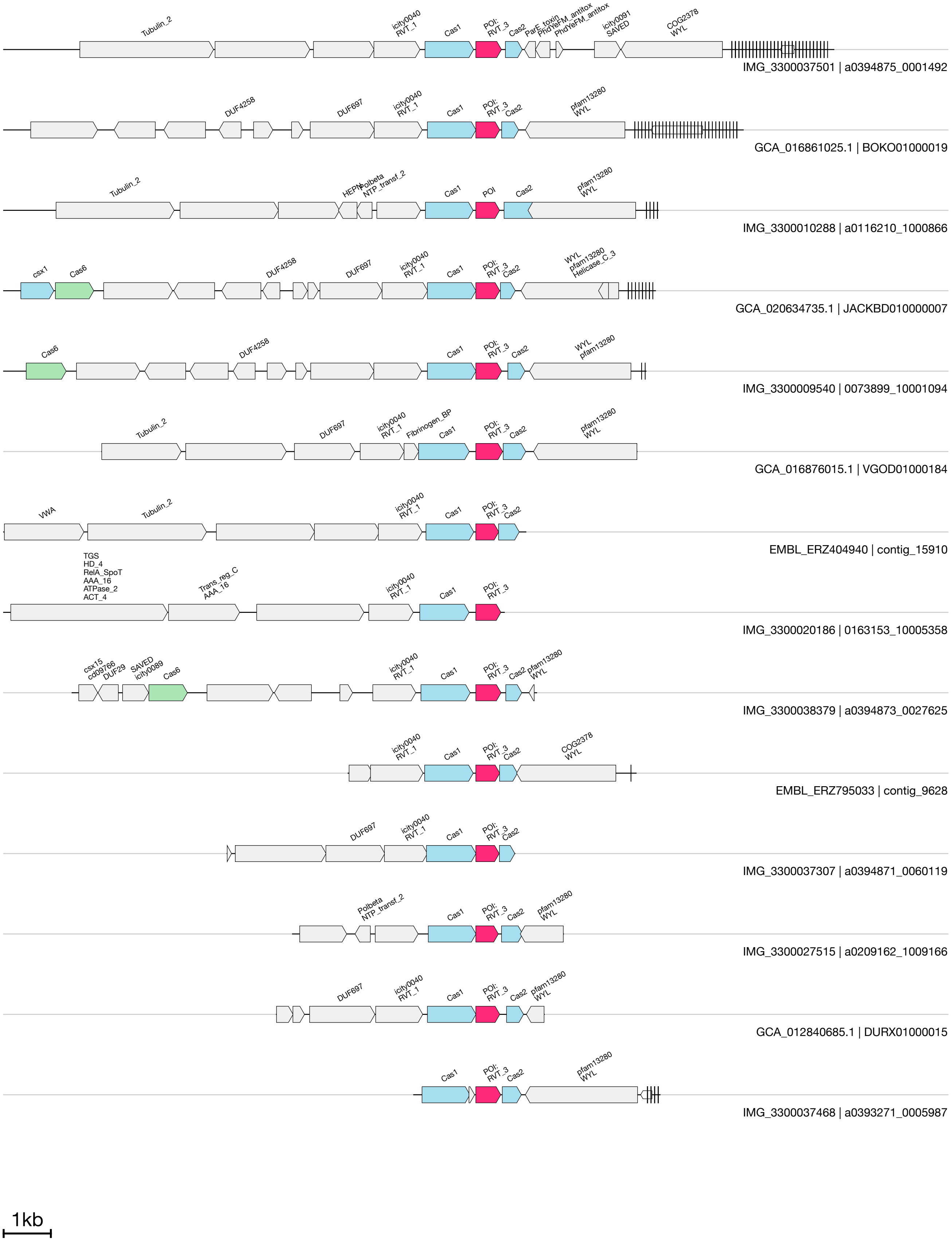

AB

UAS-13 **(DUF2797)** 6 / 7.9

Acquisition GCA_018238085.1&&JABCUA010000354&&227_1190_-1

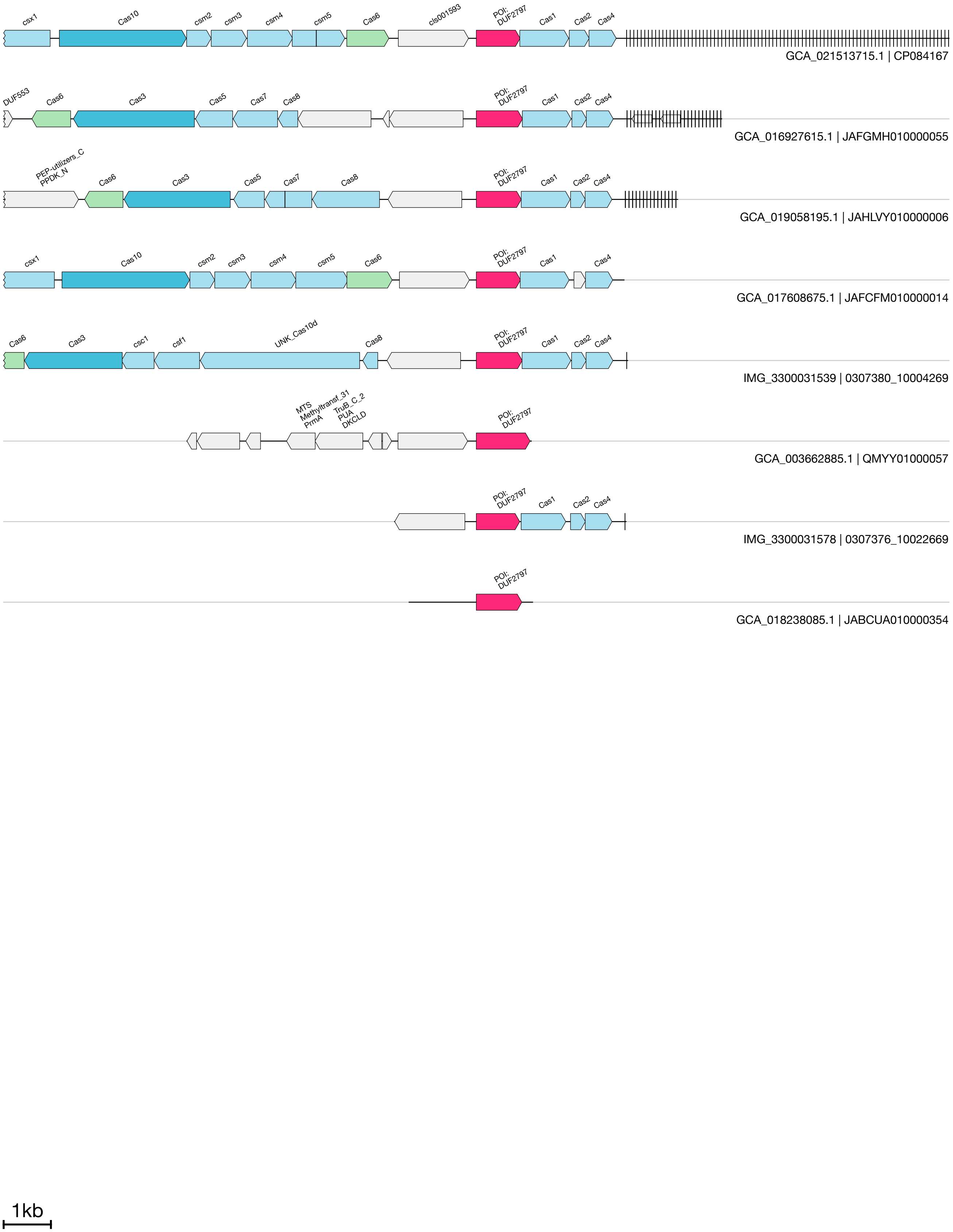

AC

UAS-4
Acquisition

(DUF1882_primase)
GCA_018712075.1&&DVHN01000112&&0_1944_1

15 / 16.5

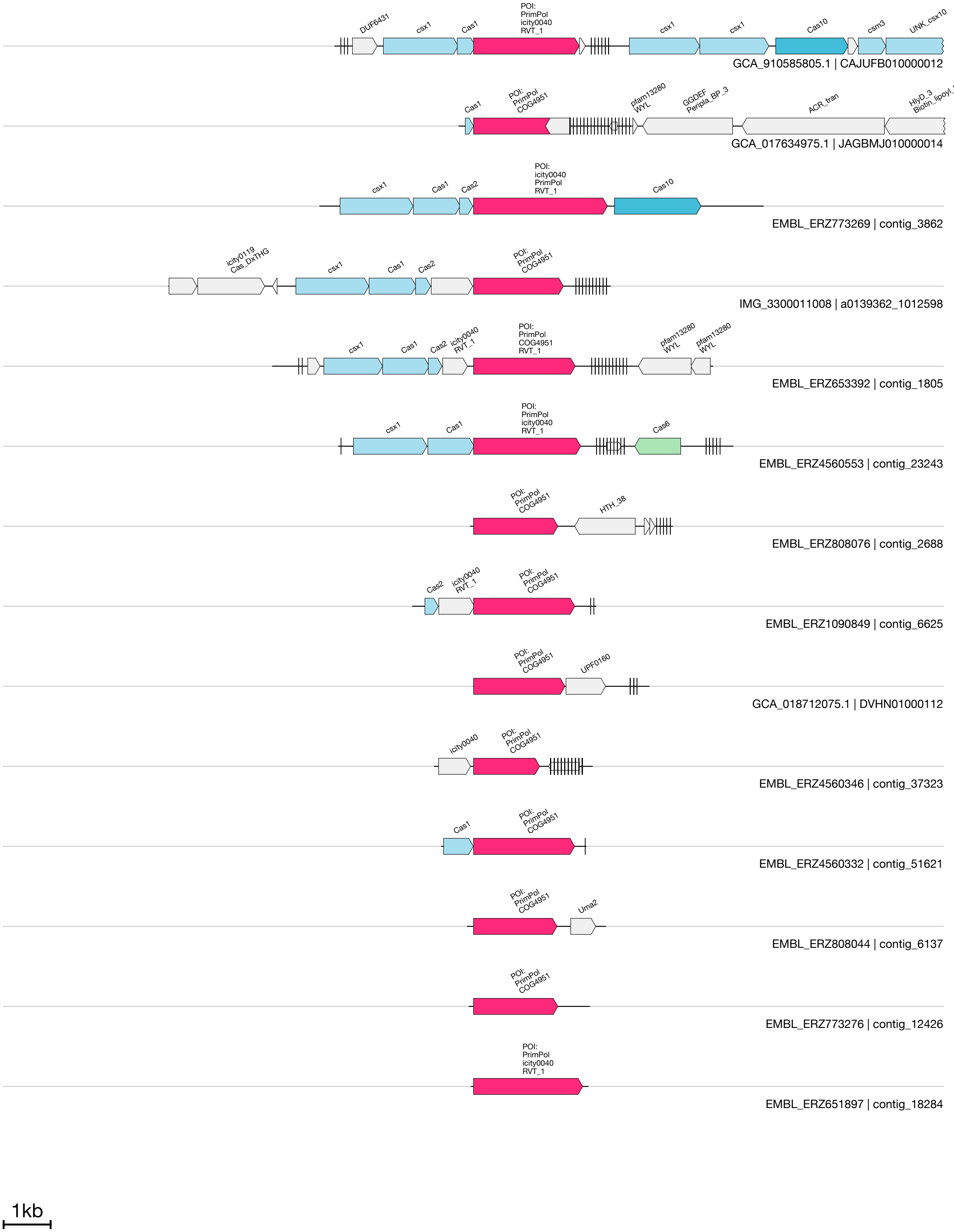

AD

UAS-5 **(DUF4384 + TPR)** 38 / 43.4
Acquisition GCA_019359625.1&&JAHHGW010000067&&164750_165512_-1

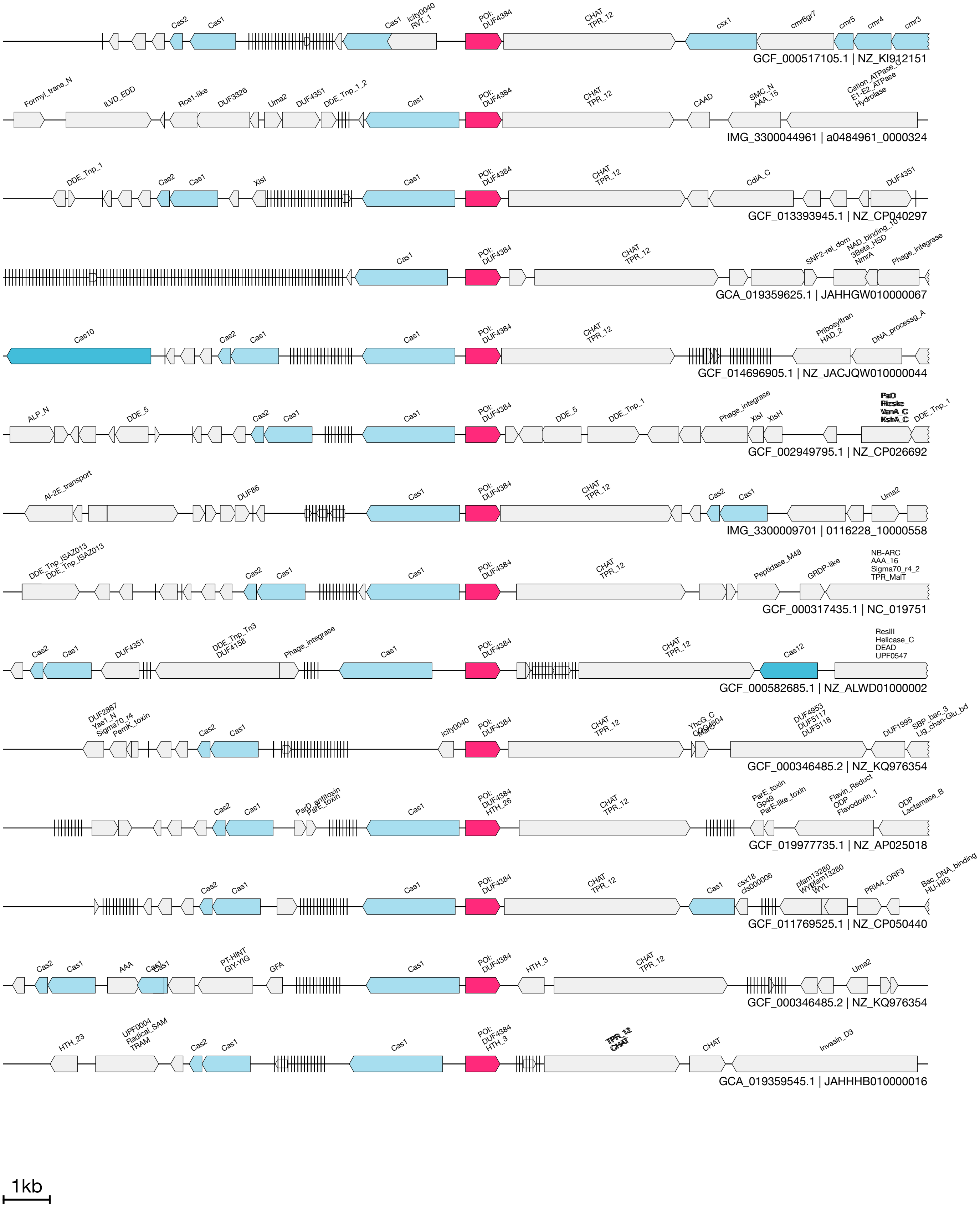

AE

UAS-46
Acquisition

(DUF3761)
GCA_020629965.1&&JAHDCG010000247&&2680_3286_1

11 / 13.8

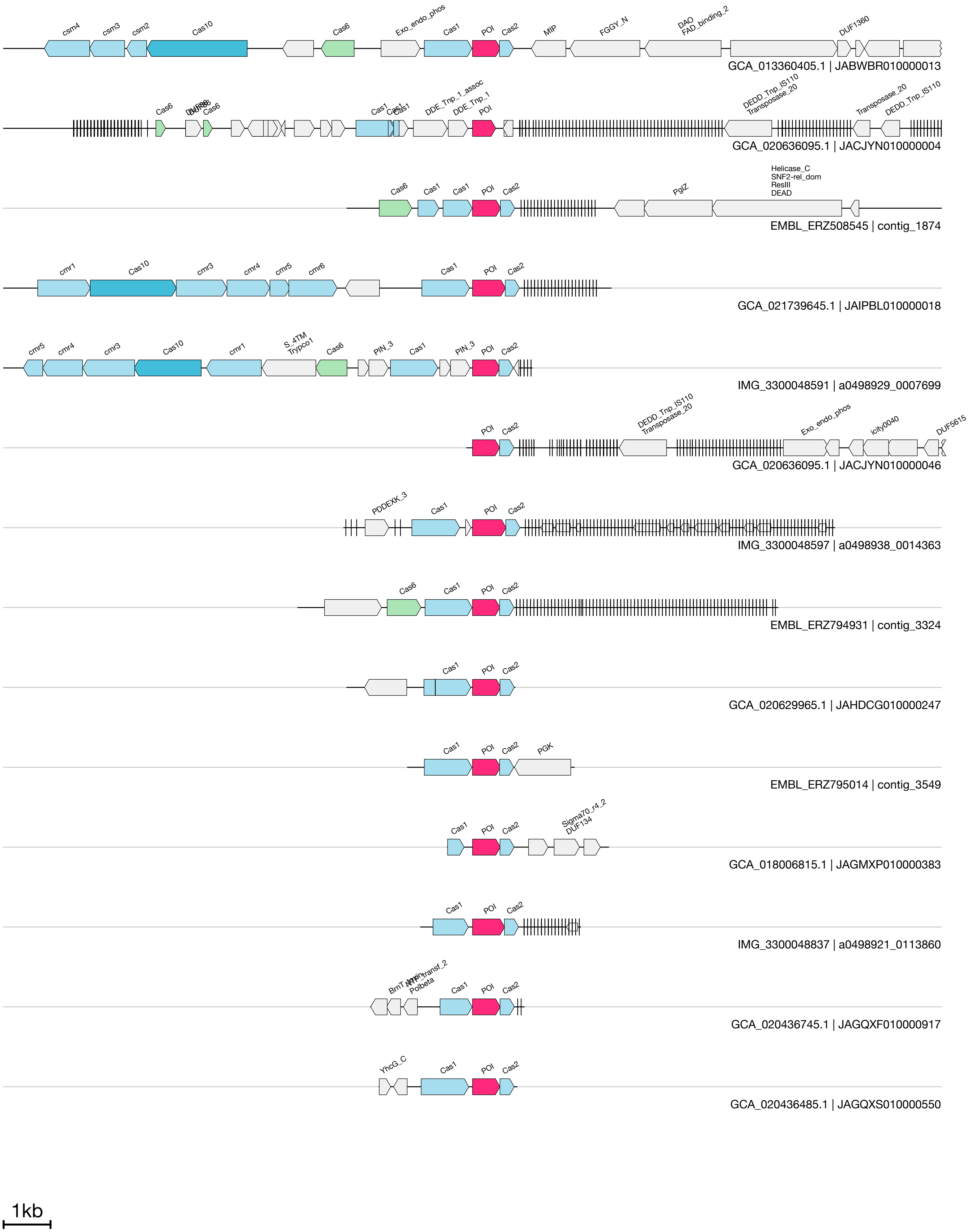

AF

UAS-10 **(HlfK)** 11 / 23.1
Acquisition GCA_903844205.1&&CAIMSW010000586&&959_2393_-1

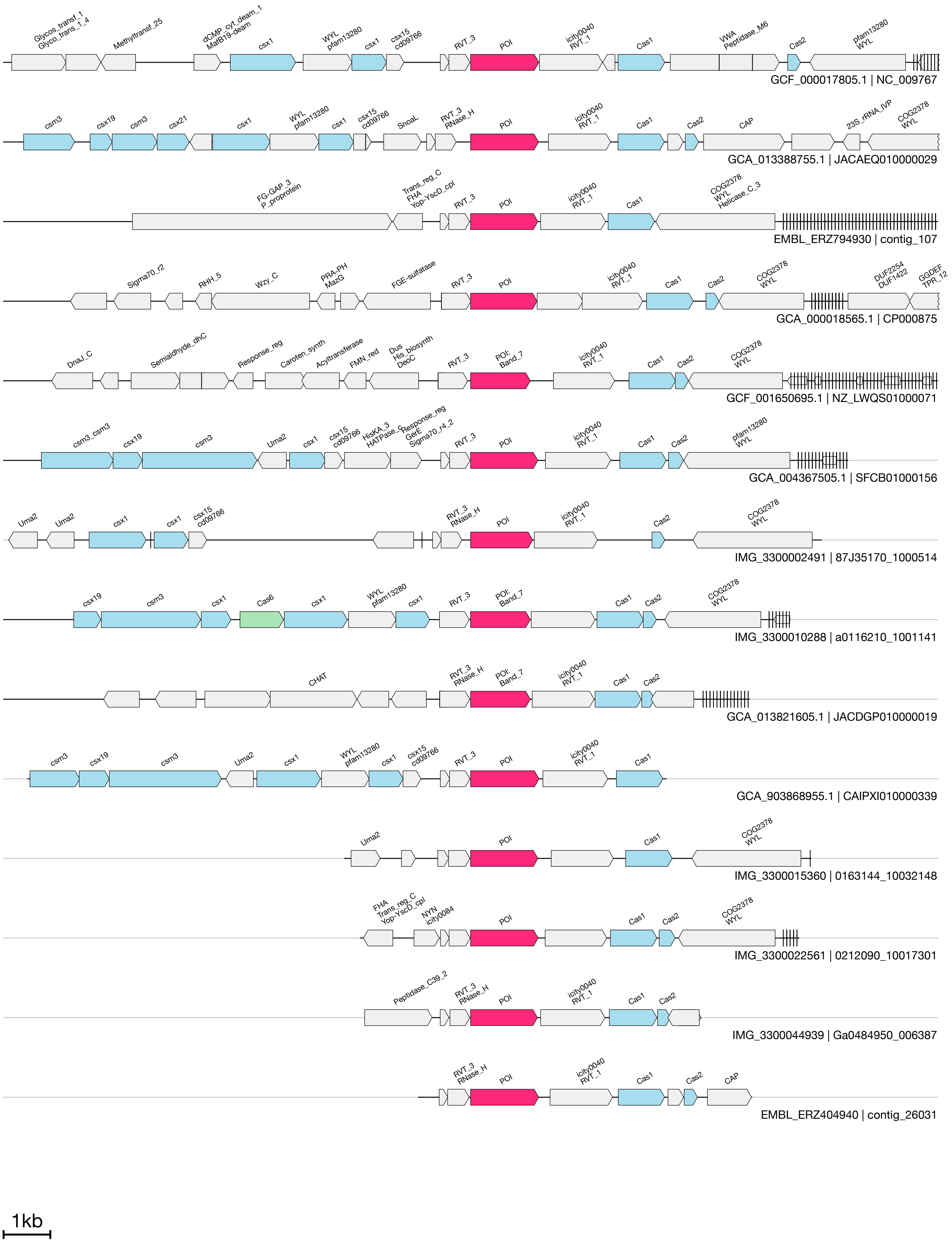

AG

UAS-8
Acquisition

(23S rRNA IVP + DUF2325 + RT sometimes)
GCF_002075285.3&&NZ_CP021983&&497963_498752_-1

18 / 22.0

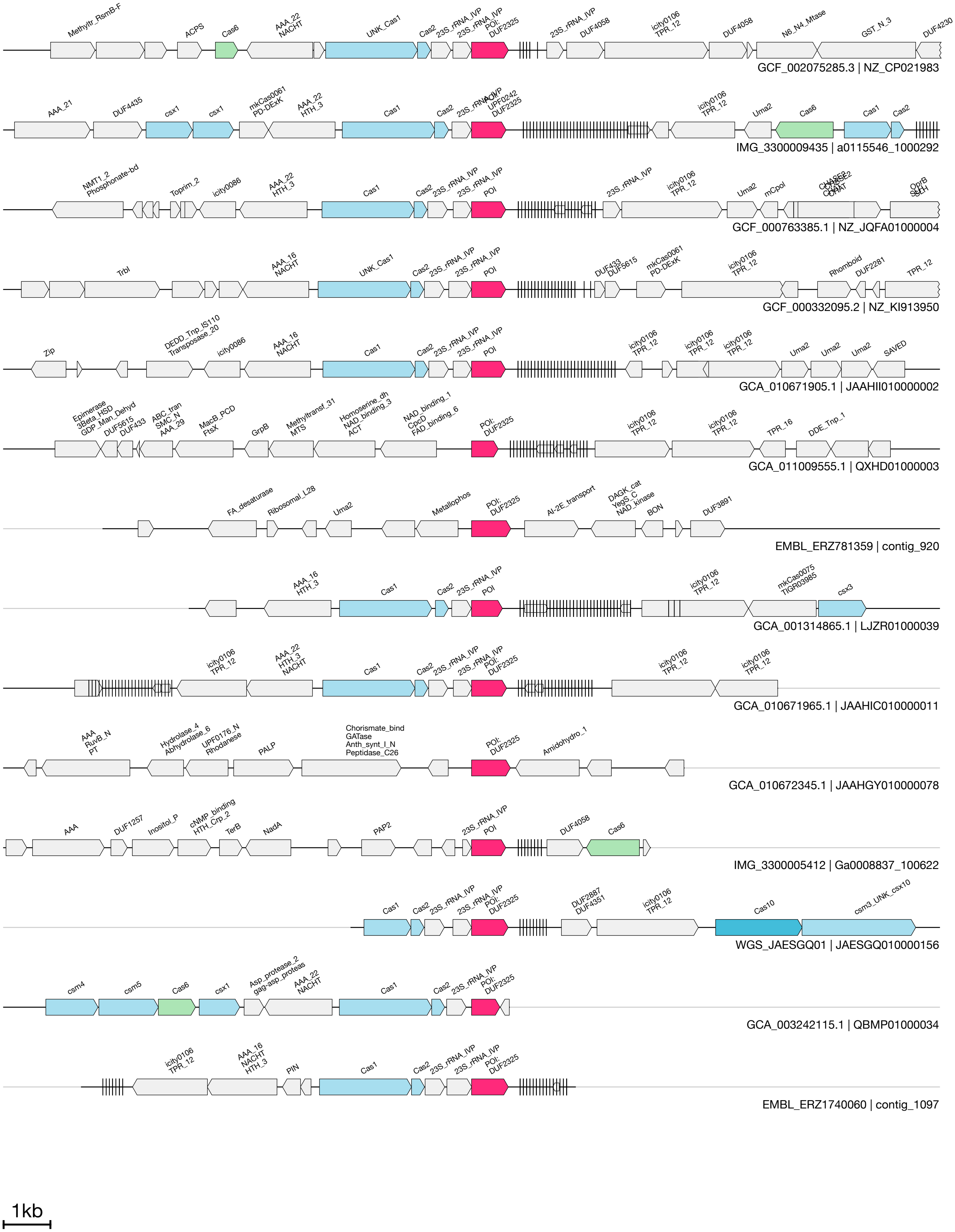

AH

UAS-11 **(type II secretion GspH)** 8 / 8.5

Acquisition GCF_014695895.1&&NZ_JACJPD010000040&&5457_6090_-1

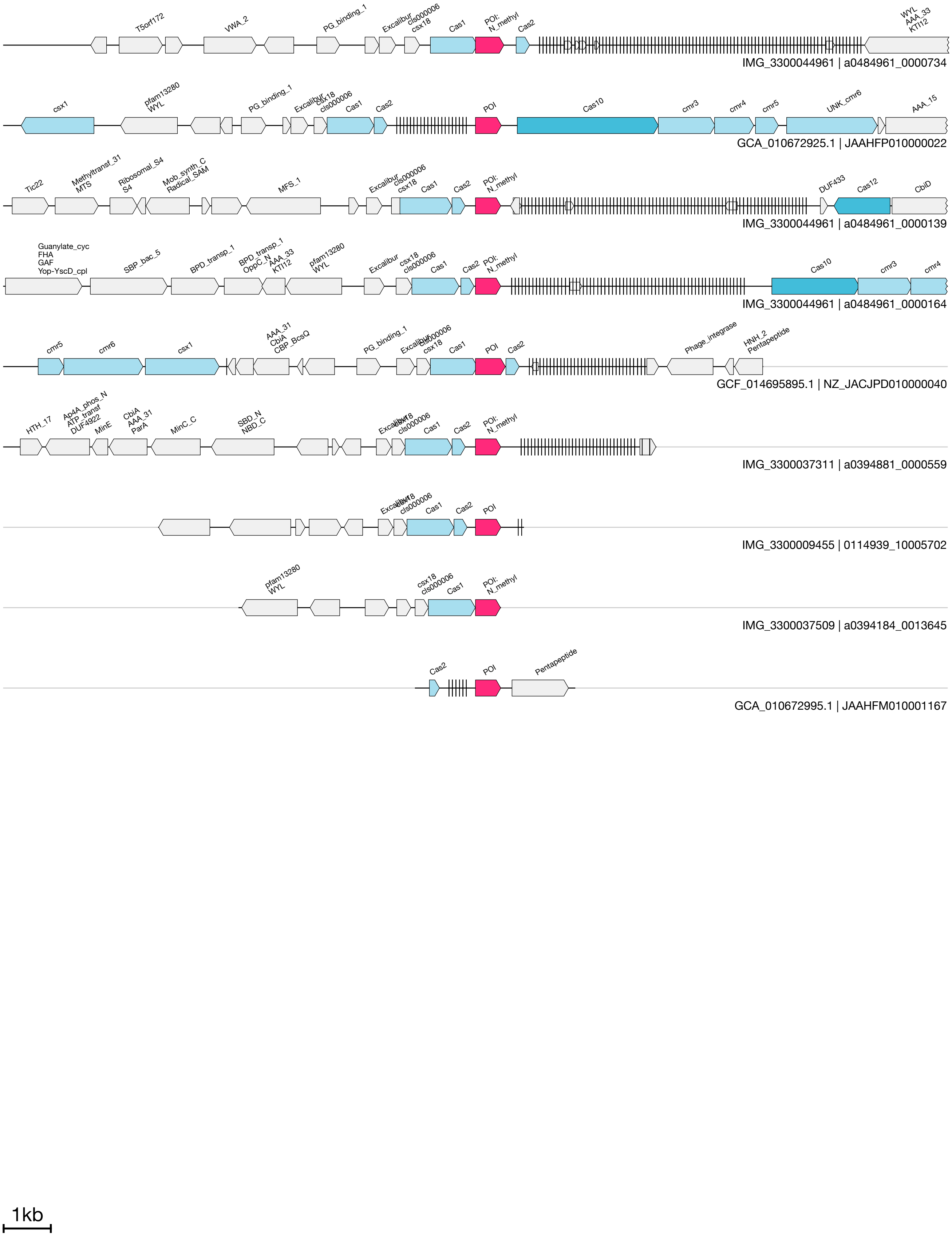

AI

UAS-17 **(Tbf2)** 13 / 32.9

Acquisition IMG_3300009499&&0114930_10001113&&3299_5312_-1

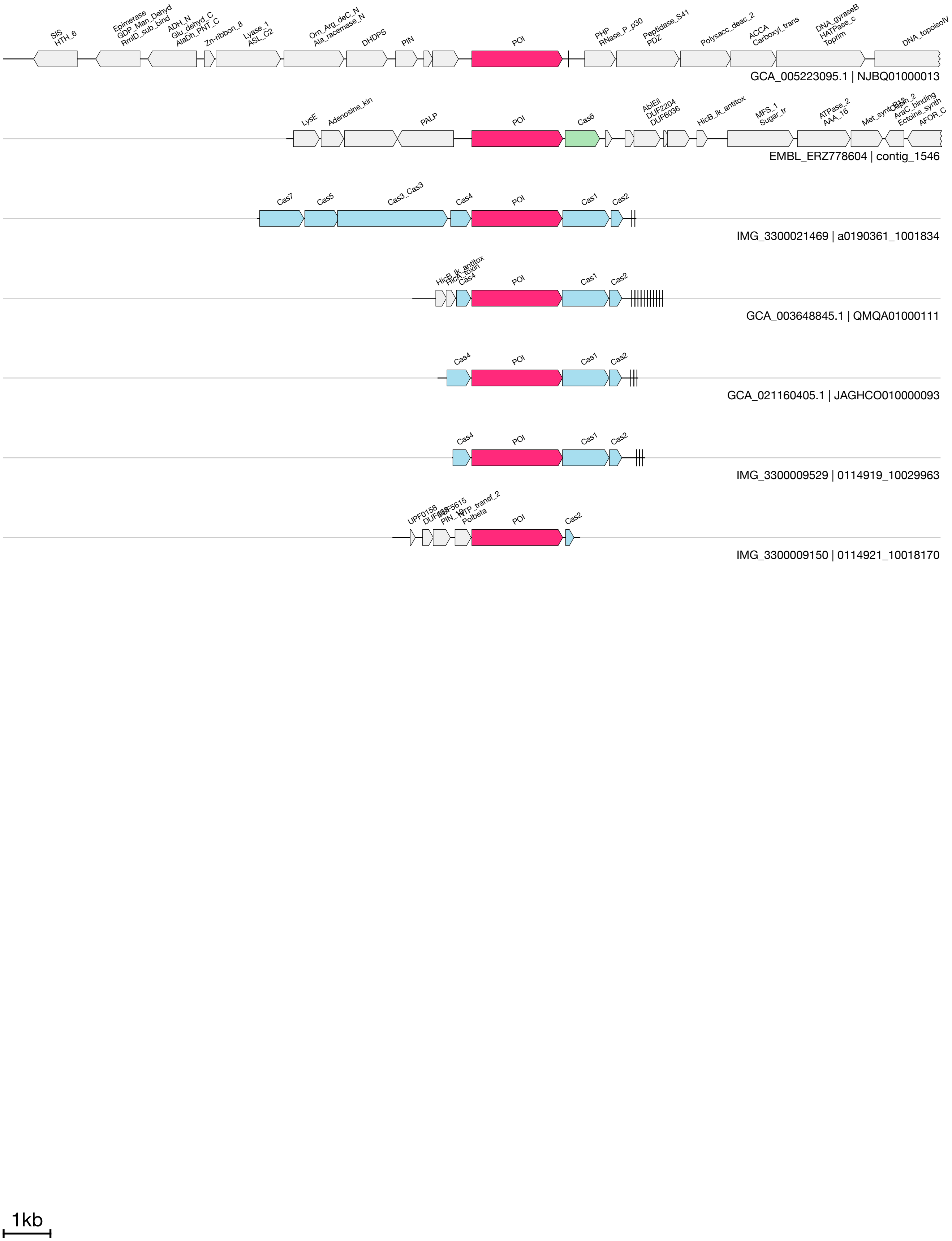

AJ

UAS-47 **(WYL)** 35 / 37.0

Acquisition IMG_3300012091&&a0136625_1012864&&2_971_1

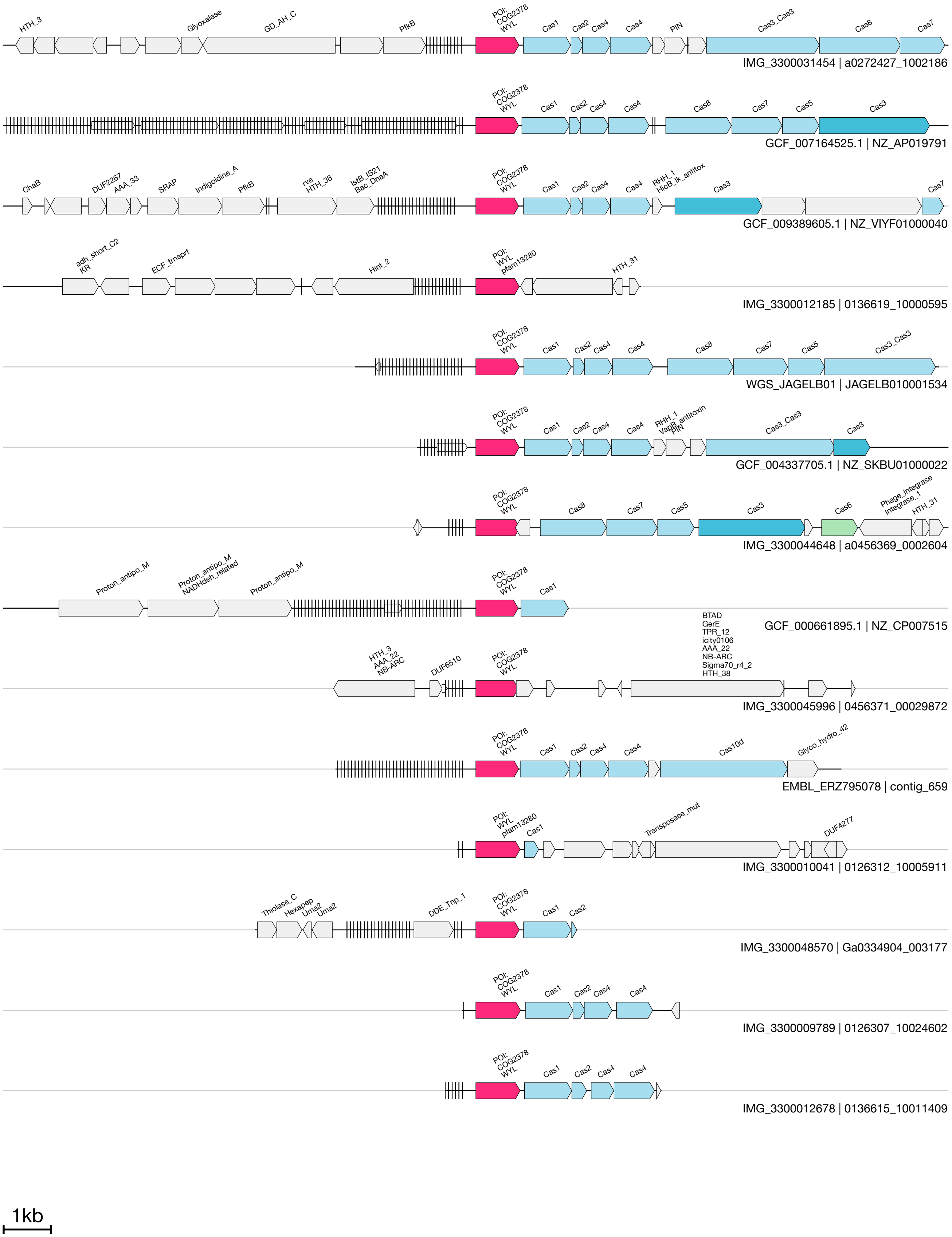

AK

UAS-1 **(Ras GTPase)** 8 / 14.3
Acquisition IMG_3300015360&&0163144_10061397&&188_1256_1

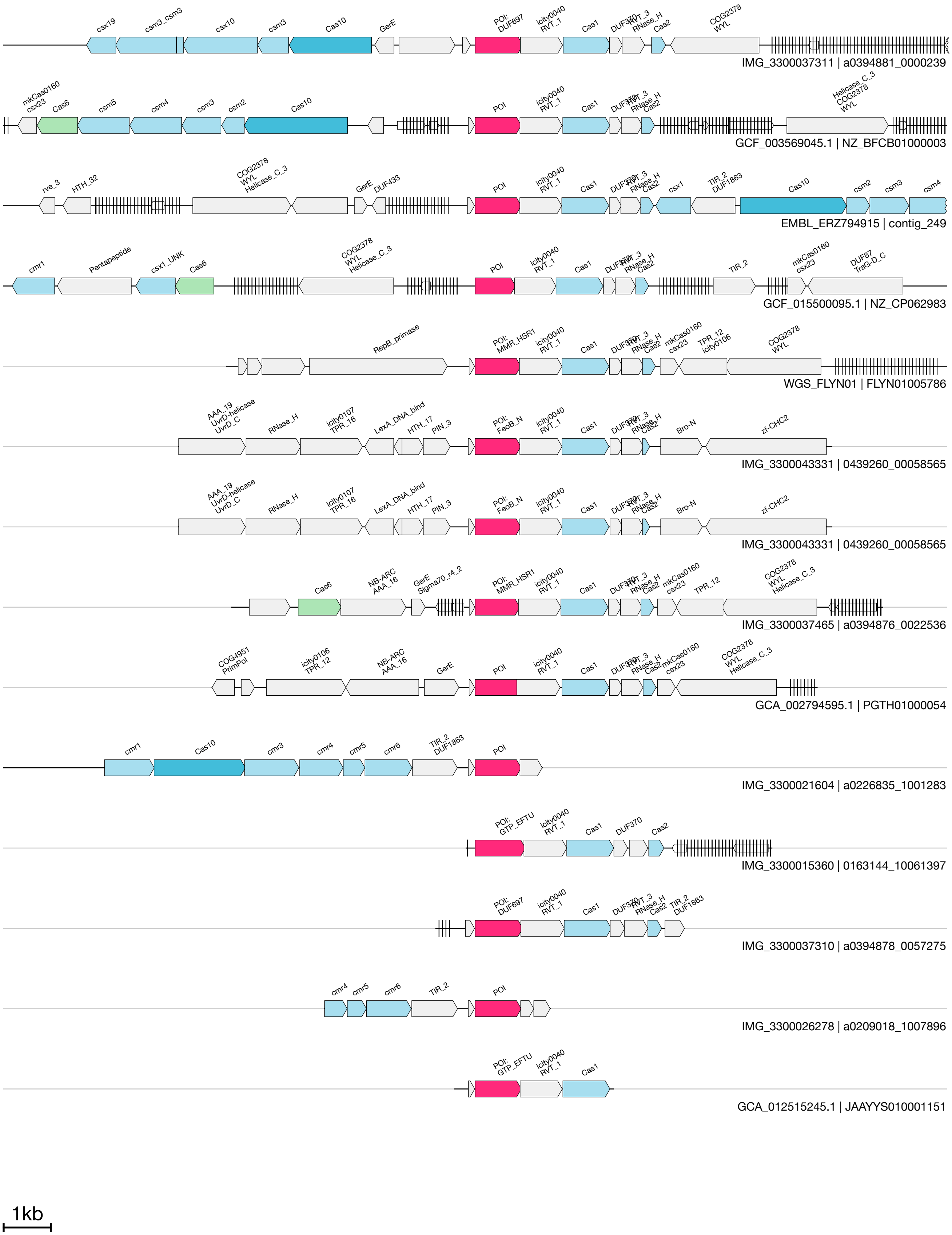

AL

UAS-48 **(Cas2 + 3'-_5' proofreading exonuclease)** 16 / 17.0
Acquisition IMG_3300019992&&Ga0197015_1040&&6129_7227_-1

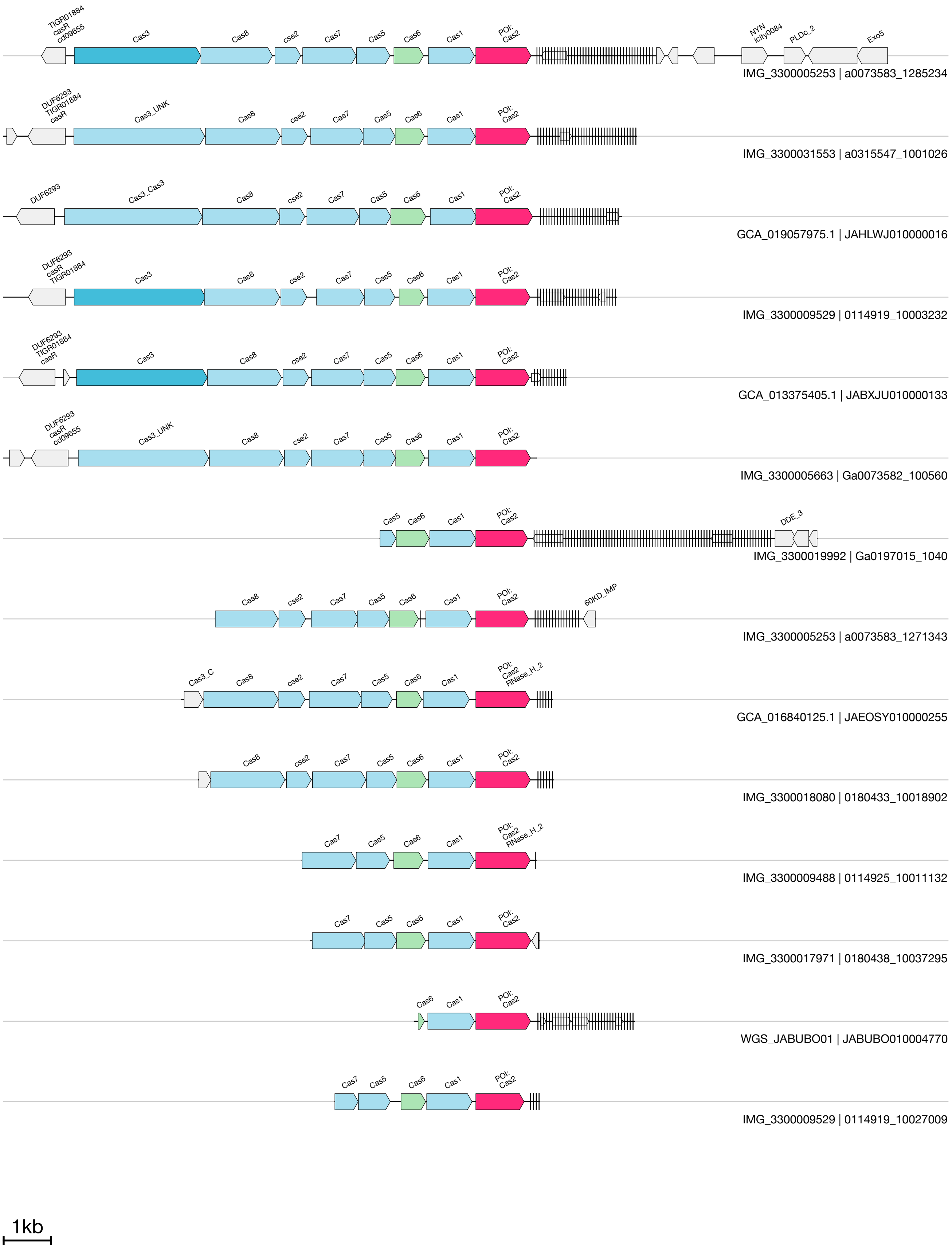

AM

UAS-12 **(FlhB)** 3 / 5.0

Acquisition IMG_3300026484&&a0256837_1016836&&561_990_-1

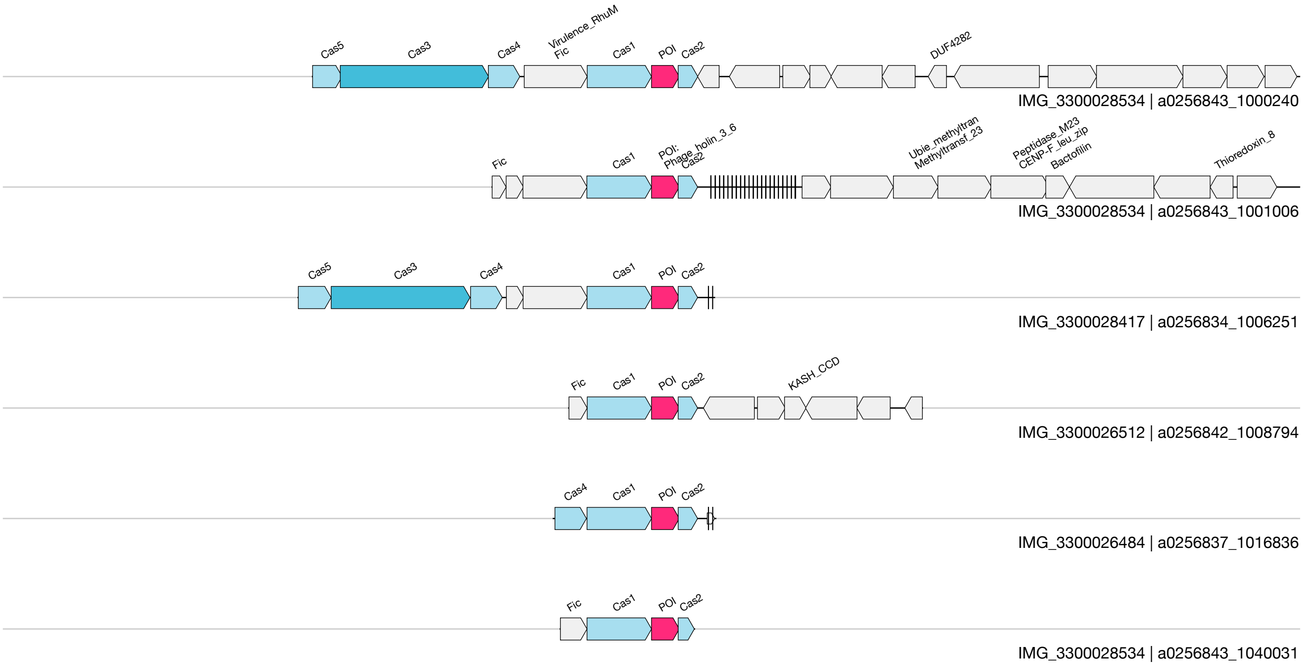

1kb

AN

UAS-9
Acquisition

(DUF4407 + other protein)
IMG_3300026531&&a0256835_1021849&&344_1451_1

23 / 39.3

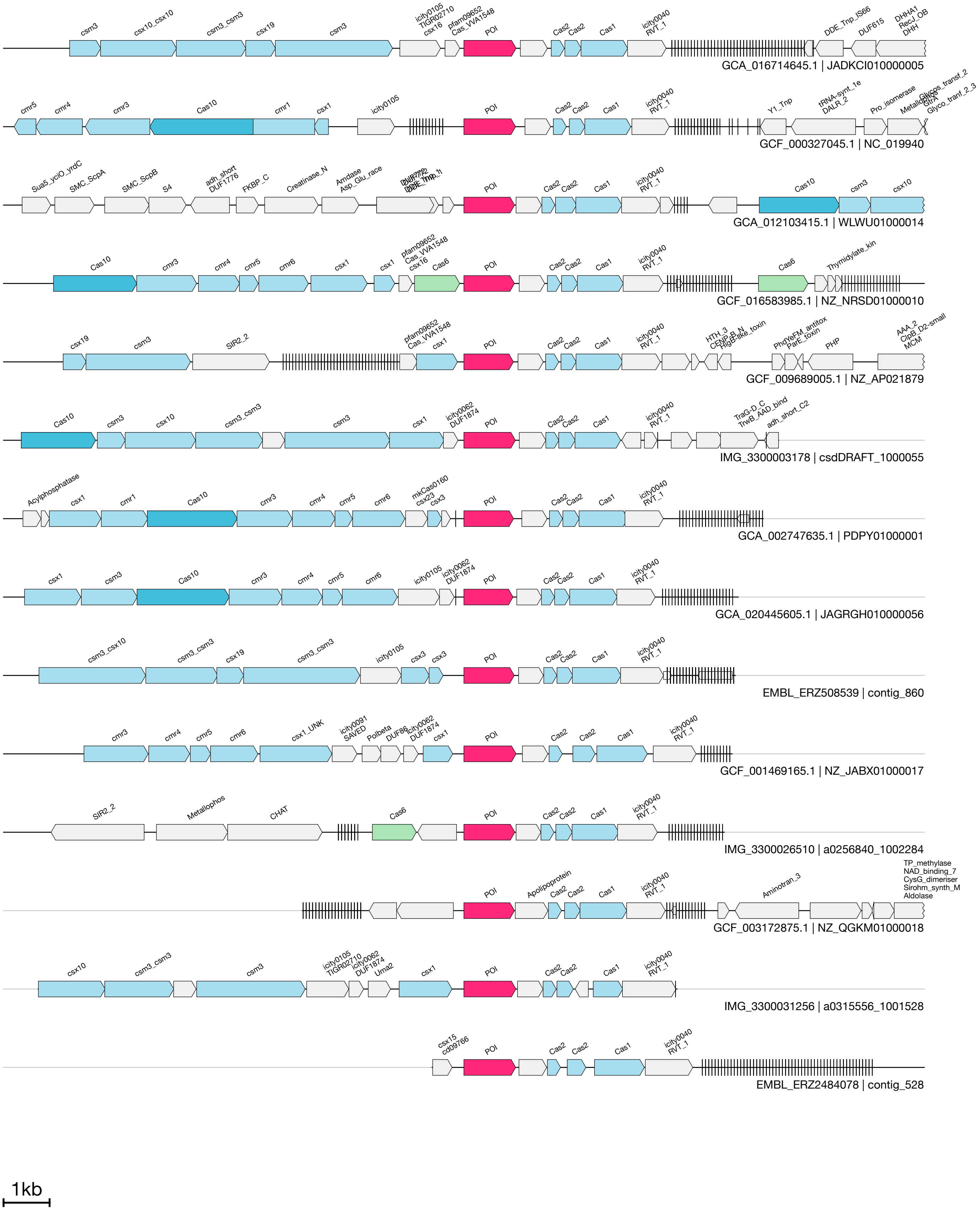

AO

UAS-14
Acquisition

(SWIM)
IMG_3300027532&&a0209544_1004784&&1334_3008_1

3 / 6.5

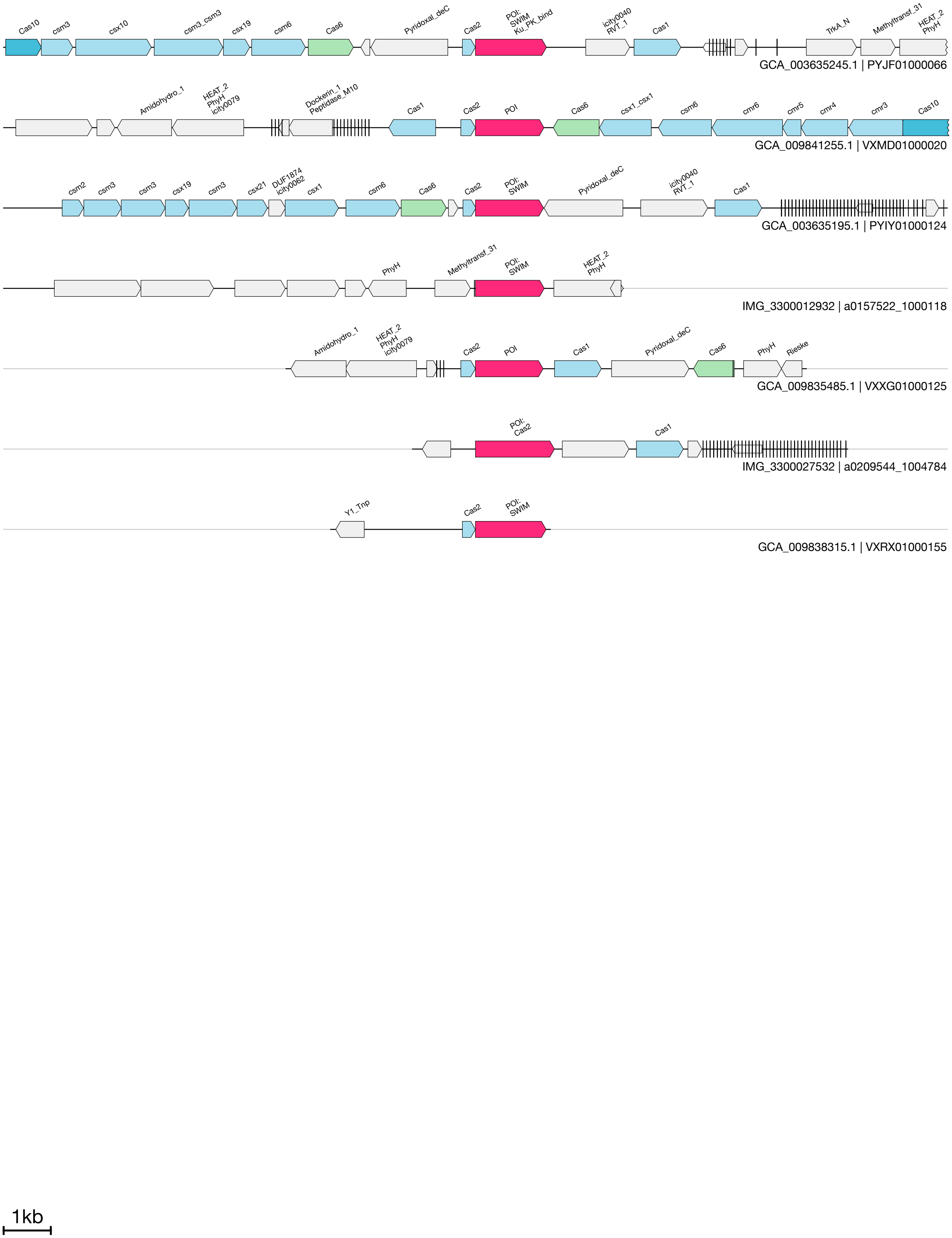

AP

UAS-6 **(Acquisition-associated NACHT)** 25 / 40.6

Acquisition IMG_3300035668&&a0372944_0001891&&1173_5076_-1

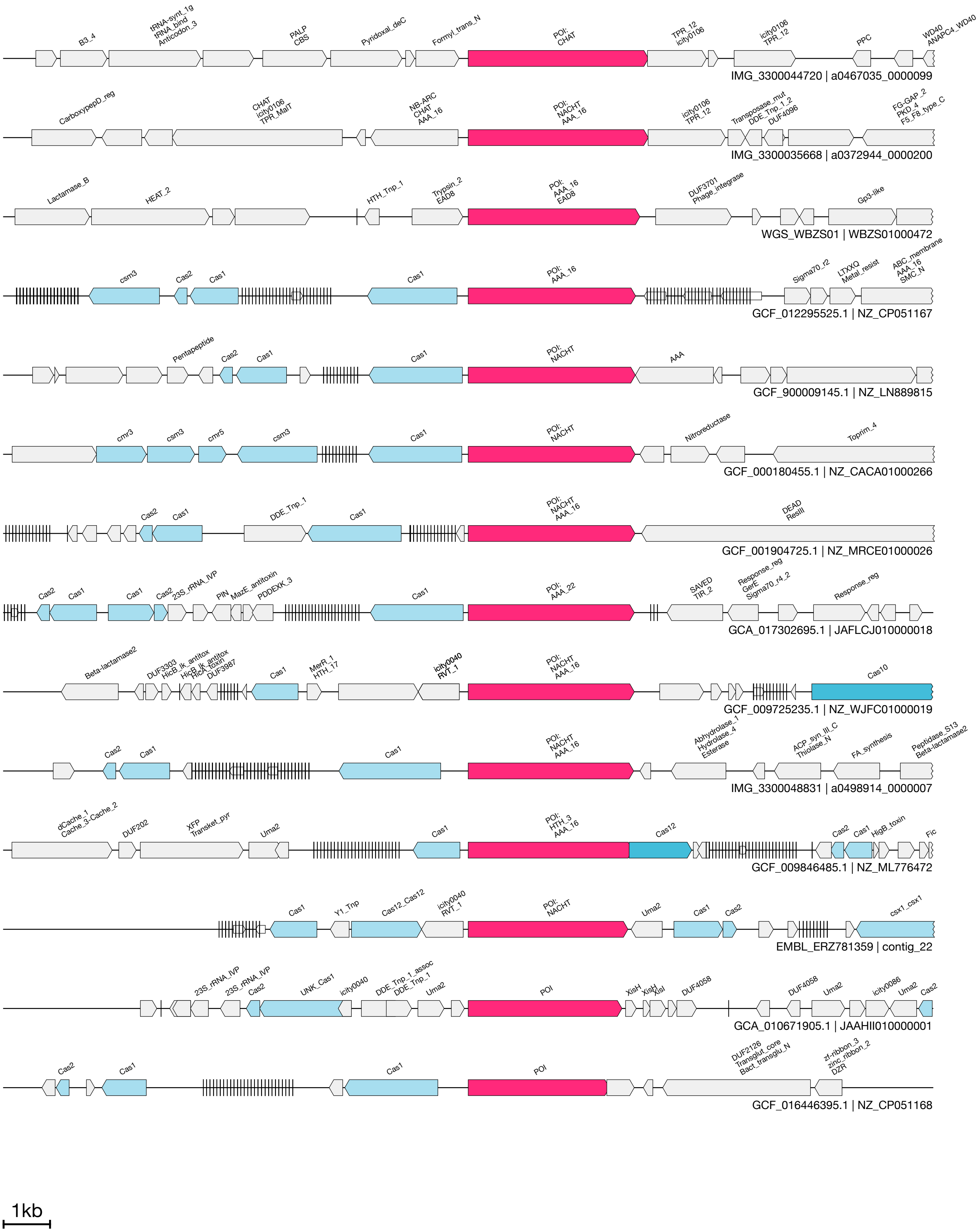

AQ

UAS-16 **(DUF3761)** 9 / 12.0

Acquisition IMG_3300037311&&a0394881_0014120&&7964_8594_-1

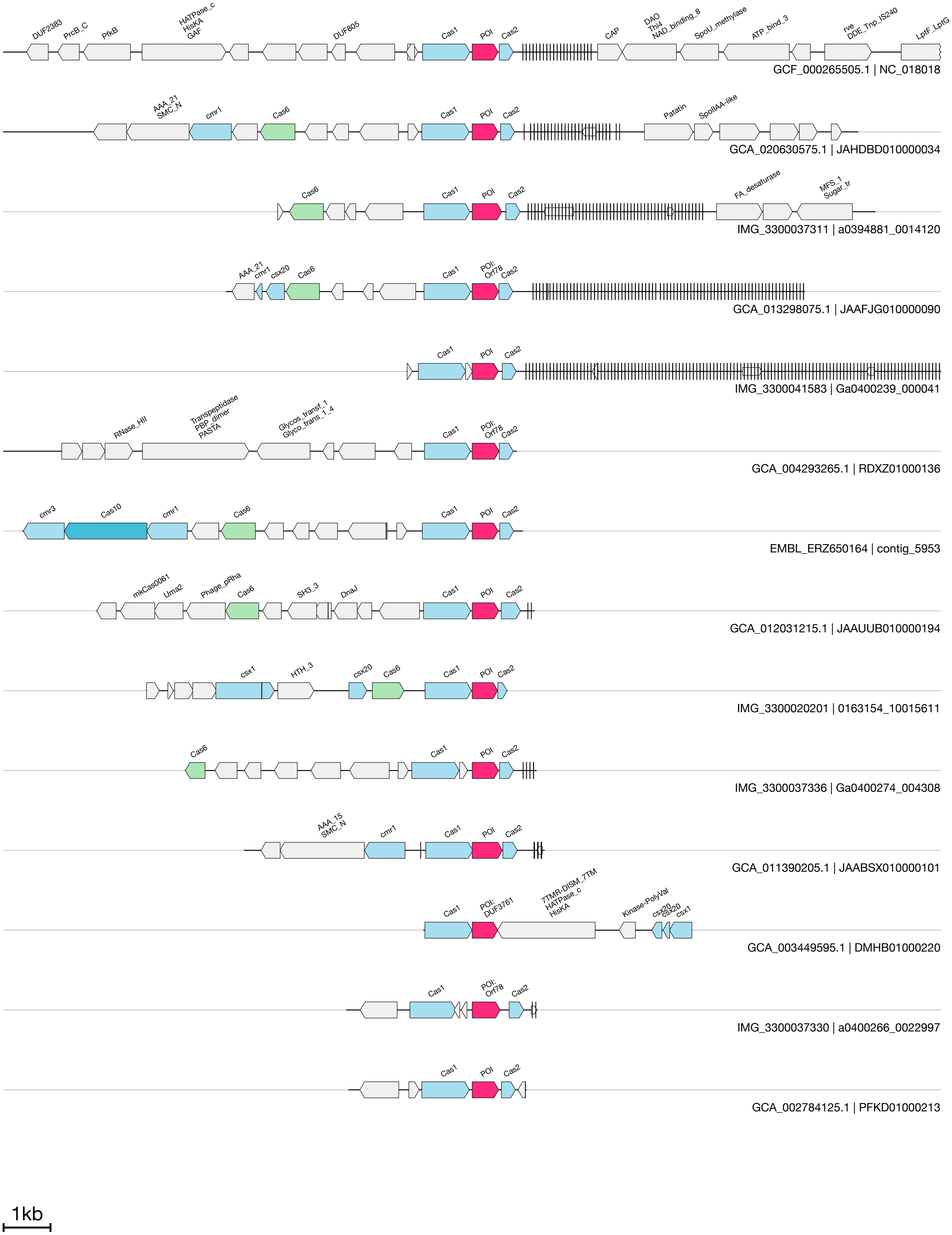

AR

UAS-15
Acquisition

(TPR + DNA polA)
WGS_JACDJC01&&JACDJC010011245&&1712_3152_-1

10 / 14.3

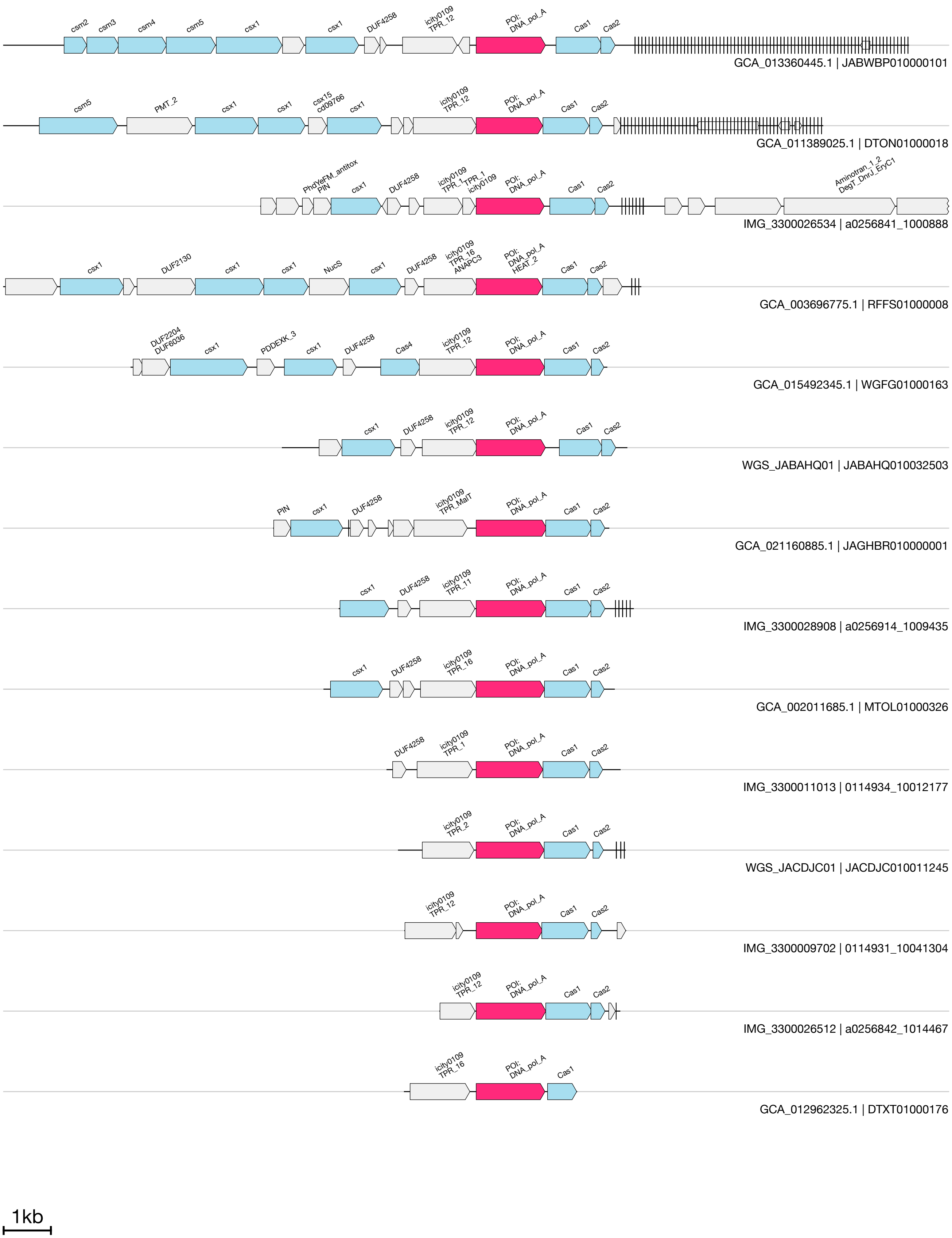

AS

UAS-49 | **(large WYL)** | 51 / 53.9
Acquisition | WGS_RRYU01&&RRYU01005723&&198_2535_1

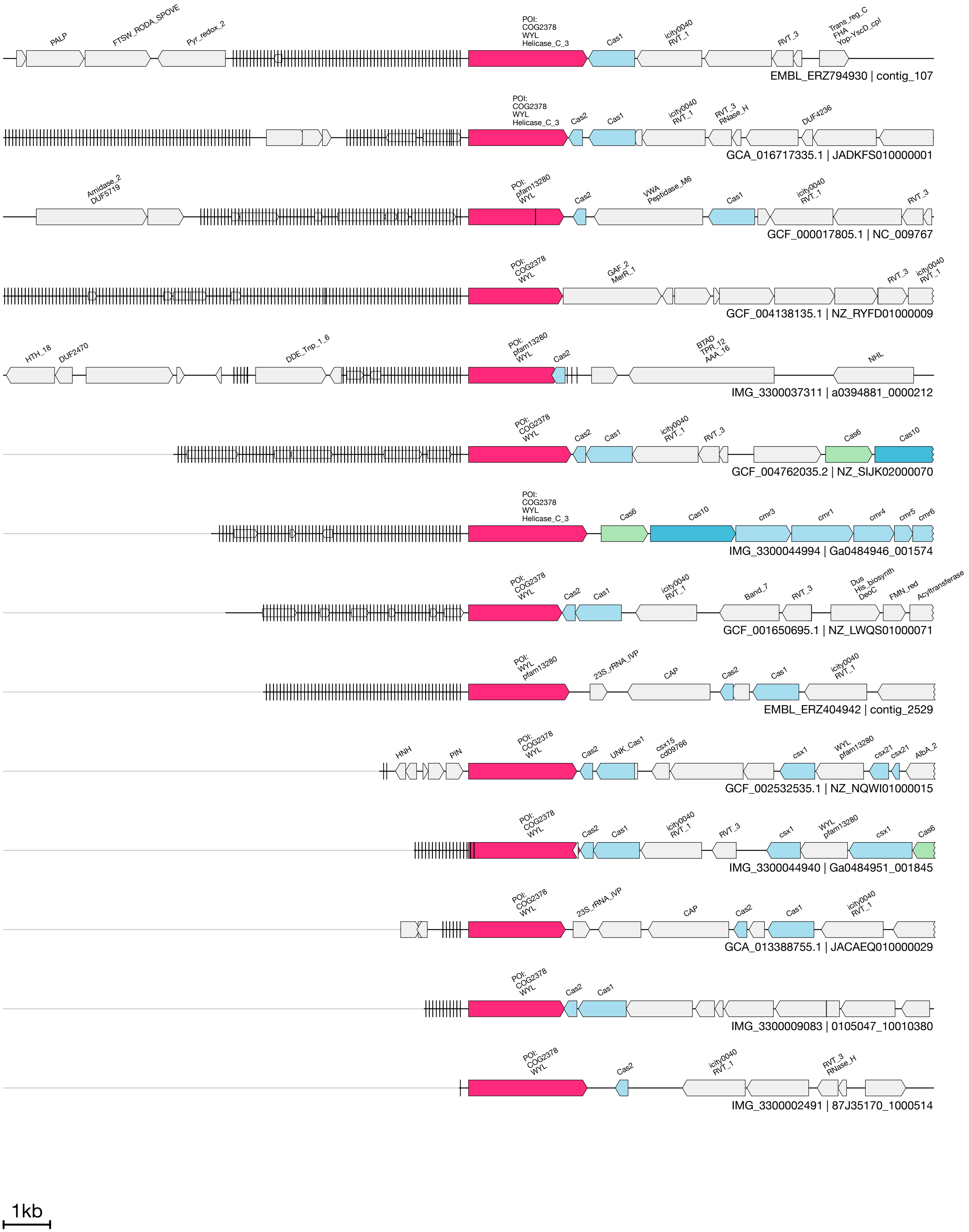

AT

UAS-50
CARF

(CARF_RelA)
EMBL_ERZ404942&&contig_42542&&228_1605_-1

8 / 11.5

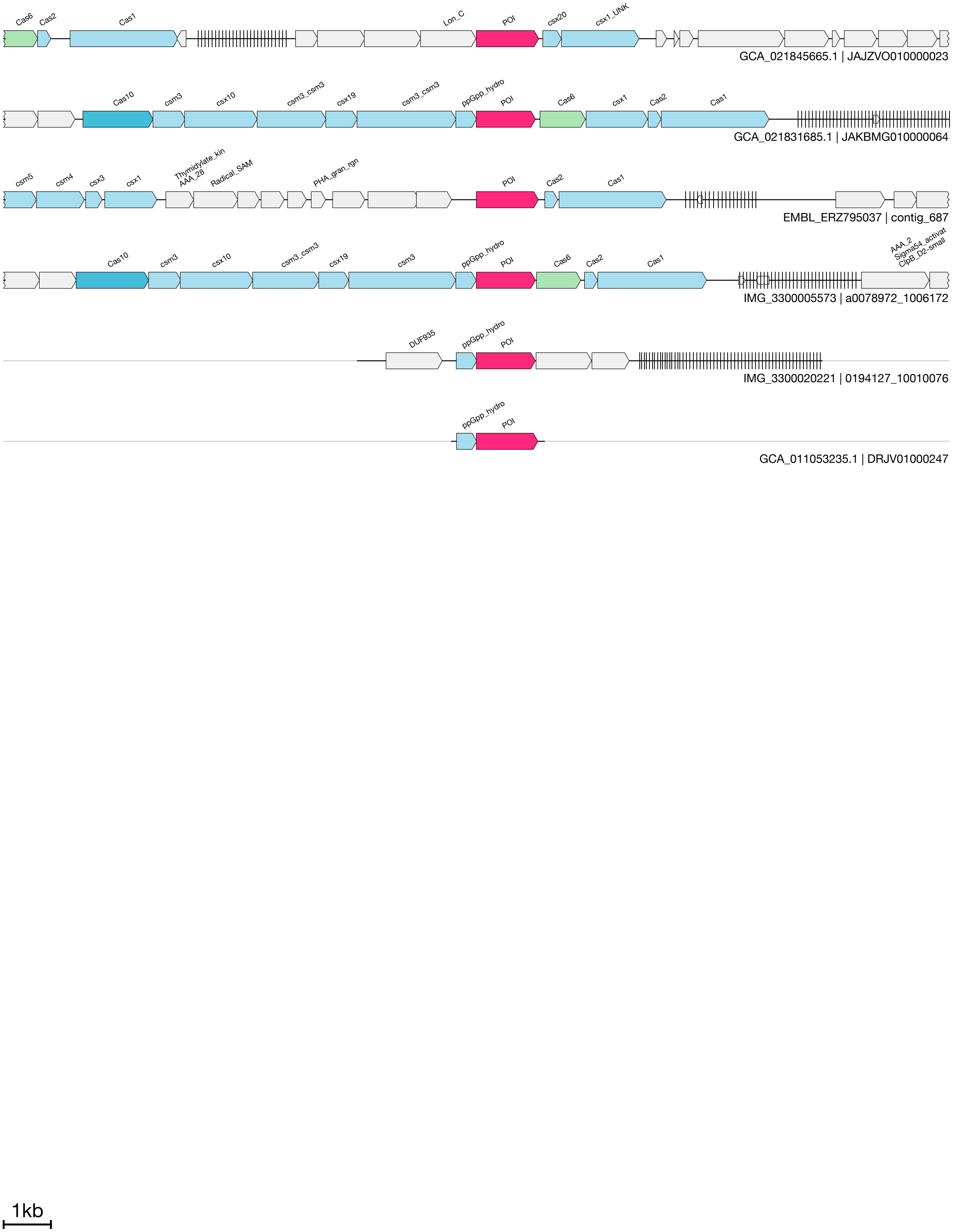

AU

UAS-51 **(SAVED_TIR2)** 4 / 5.2
CARF EMBL_ERZ773005&&contig_5&&212166_213489_1

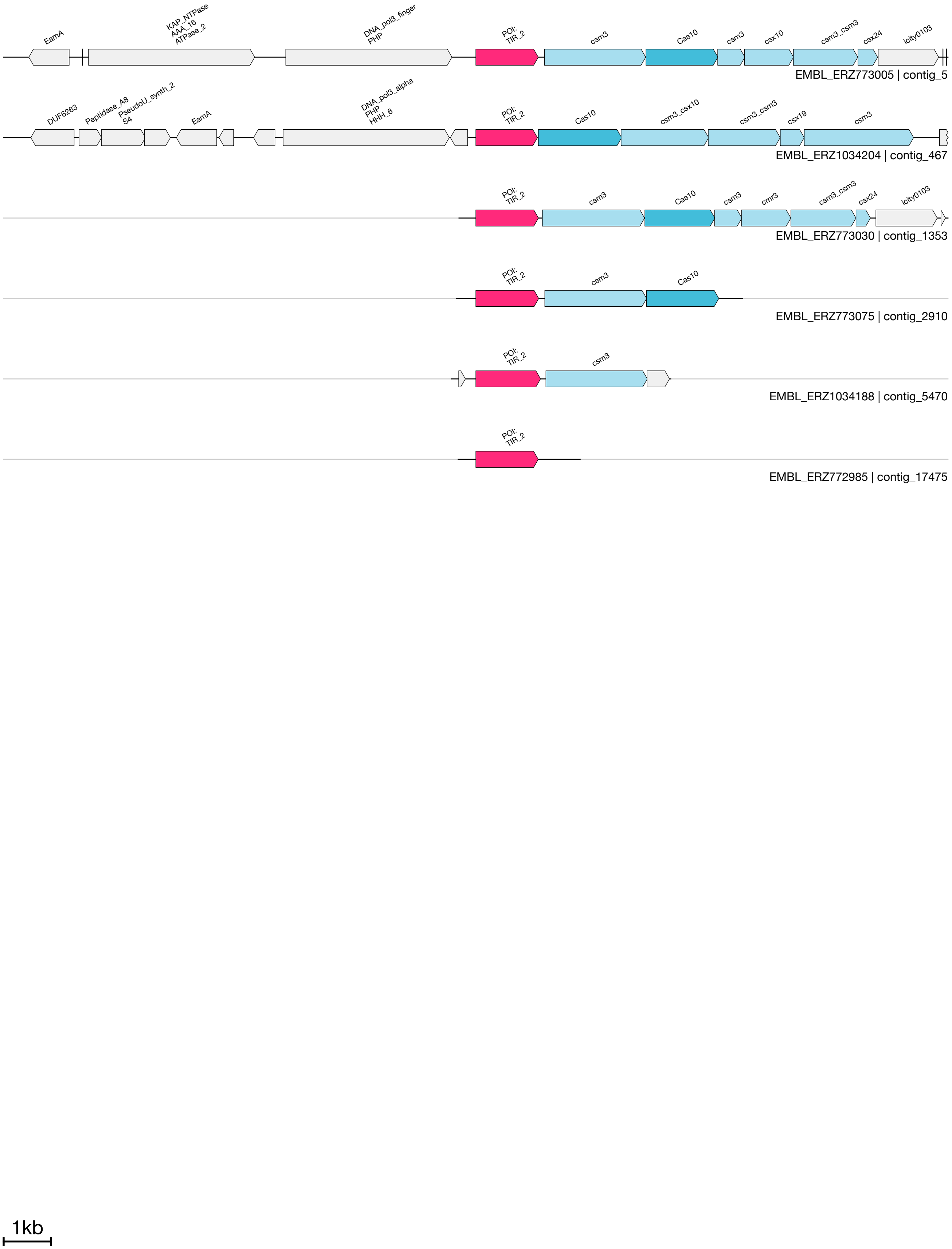

AV

UAS-52 **(CARF_Sigma)** N/A
CARF EMBL_ERZ795034&&contig_47960&&1548_2448_-1 extraction

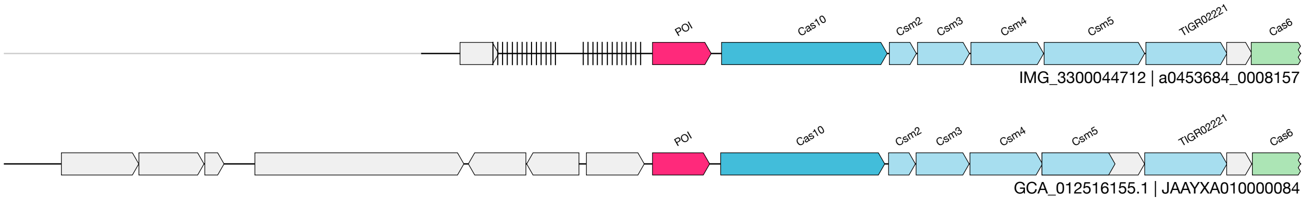

1kb

AW

UAS-53 **(CARF_DUF859 + many nearby genes)** 4 / 5.0

CARF EMBL_ERZ894180&&contig_692&&8871_11661_-1 extraction

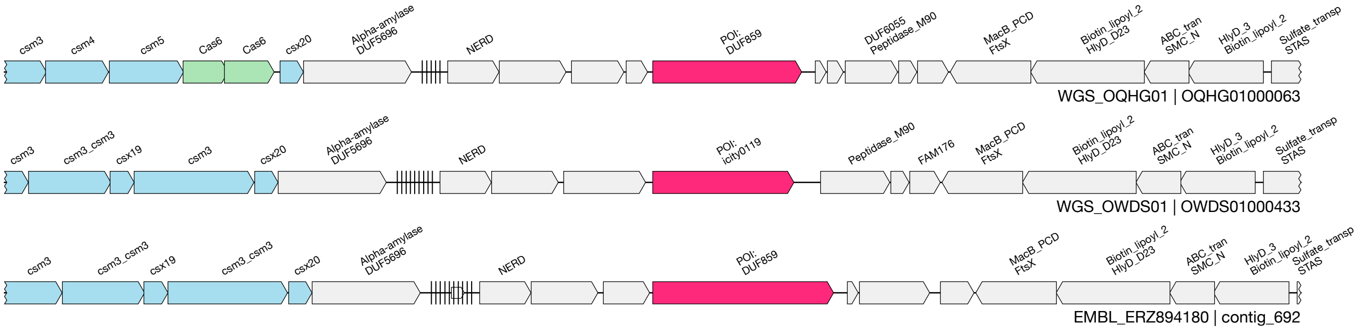

1kb

AX

UAS-54 **(SIR2 _ CHAT _ SAVED + SMODS)** 5 / 10.3

CARF GCA_016712315.1&&JADJRG010000028&&300098_302447_1

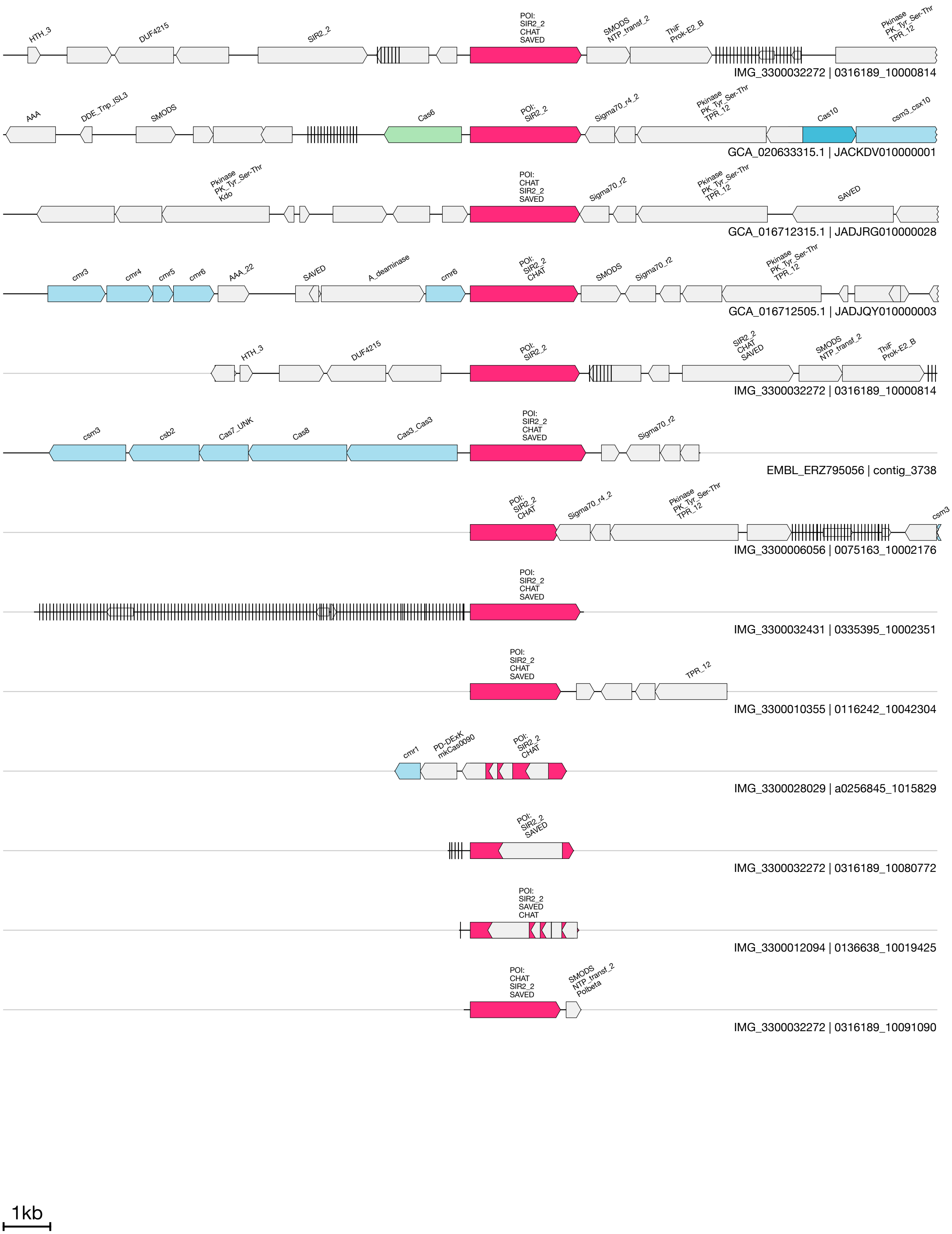

AY

UAS-55 **(EAD7 _ SAVED)** 2 / 5.6
CARF GCA_020634195.1&&JACKCE010000043&&1971_2784_-1

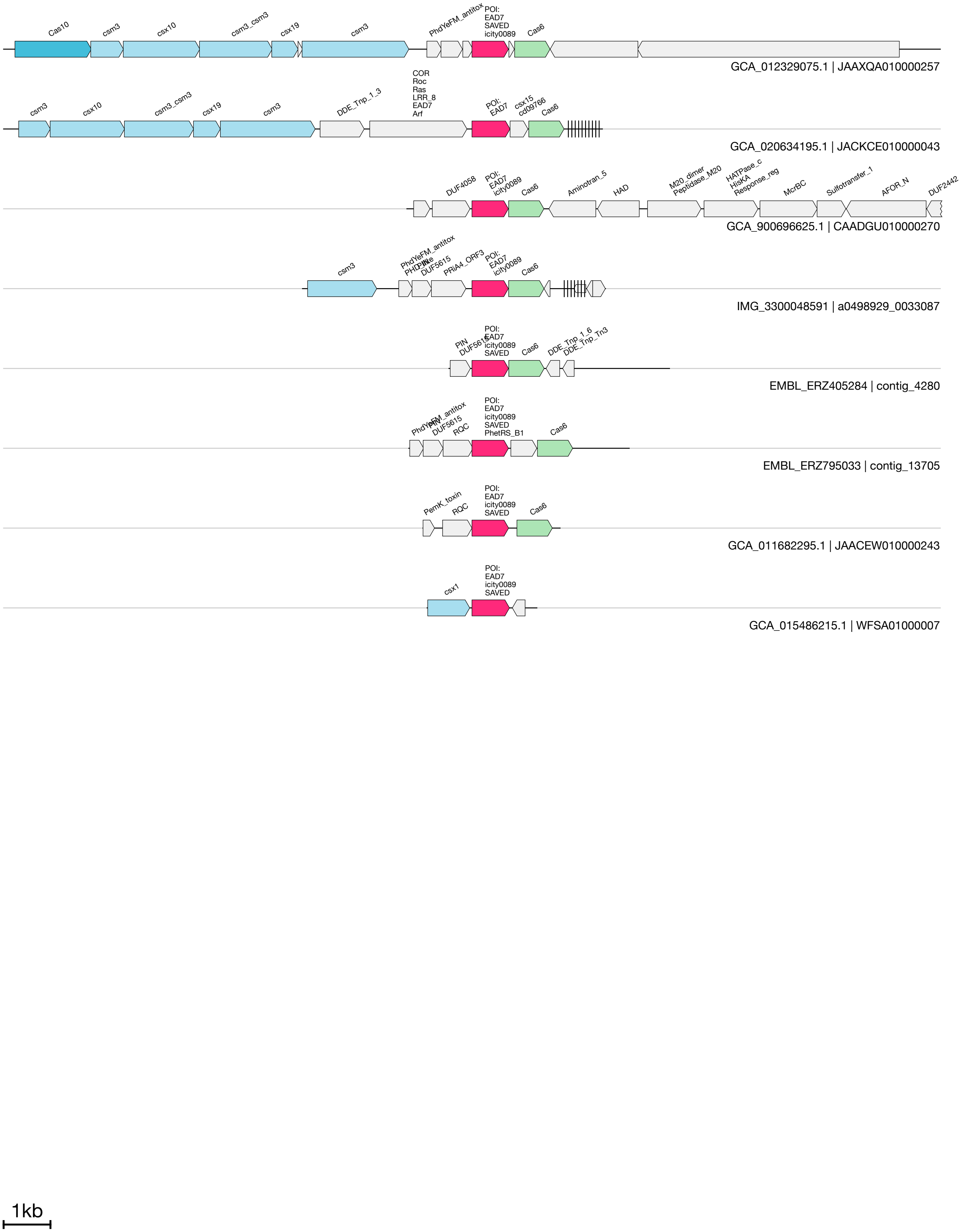

AZ

UAS-56 **(CARF_DUF859_ATPase + many nearby genes)** 7 / 8.7

CARF GCA_900541305.1&&UQJJ01000039&&1337_4901_1 extraction

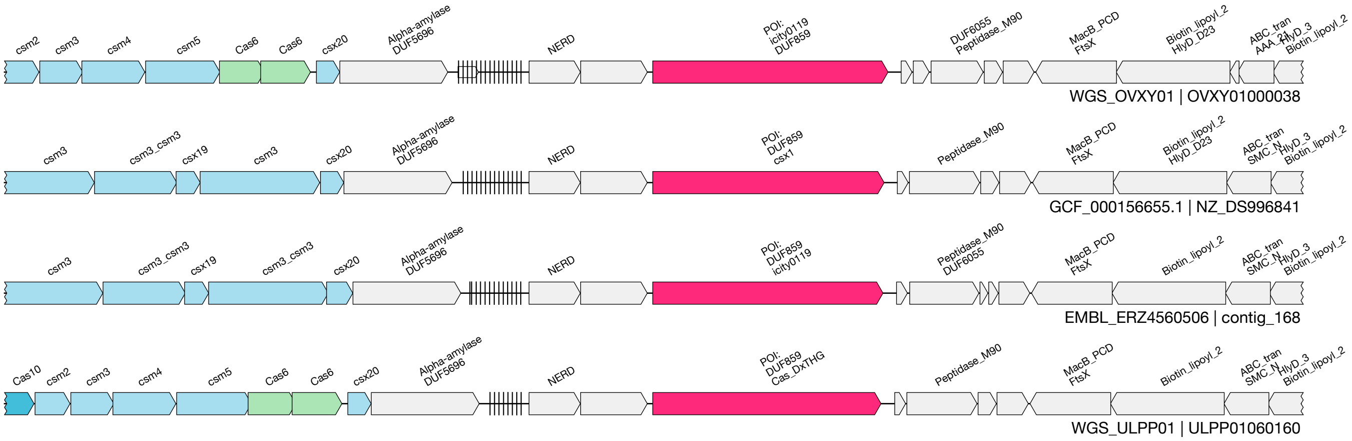

1kb

BA

UAS-57 **(SAVED TIR)** 9 / 18.6

CARF GCF_000024805.1&&NC_013440&&7674710_7676006_1

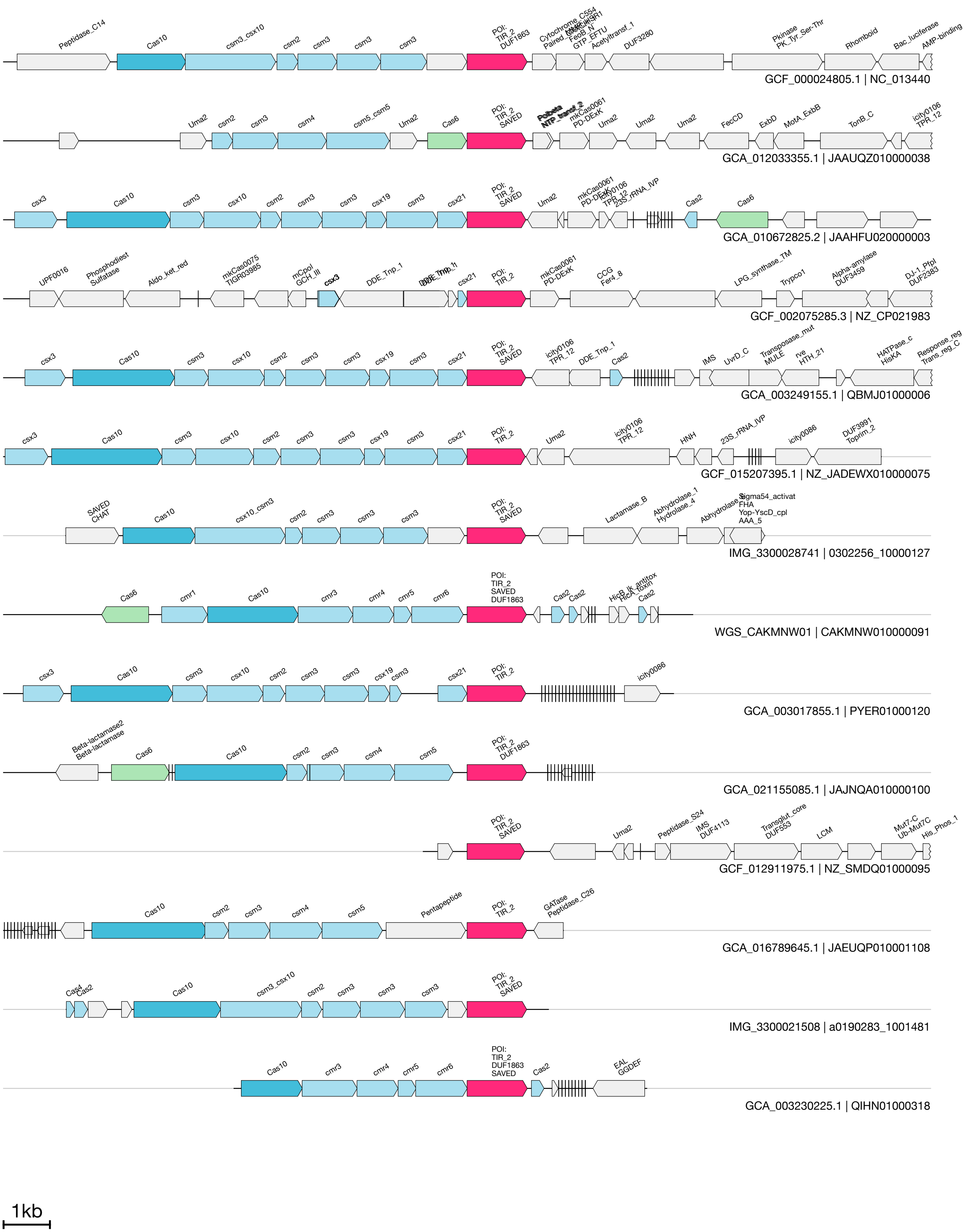

BB

UAS-58 **(CARF_ATPase_HTH + RNAPol)** N/A

CARF GCF_000224065.1&&NZ_AFWT01000006&&121441_122968_1 extraction 2

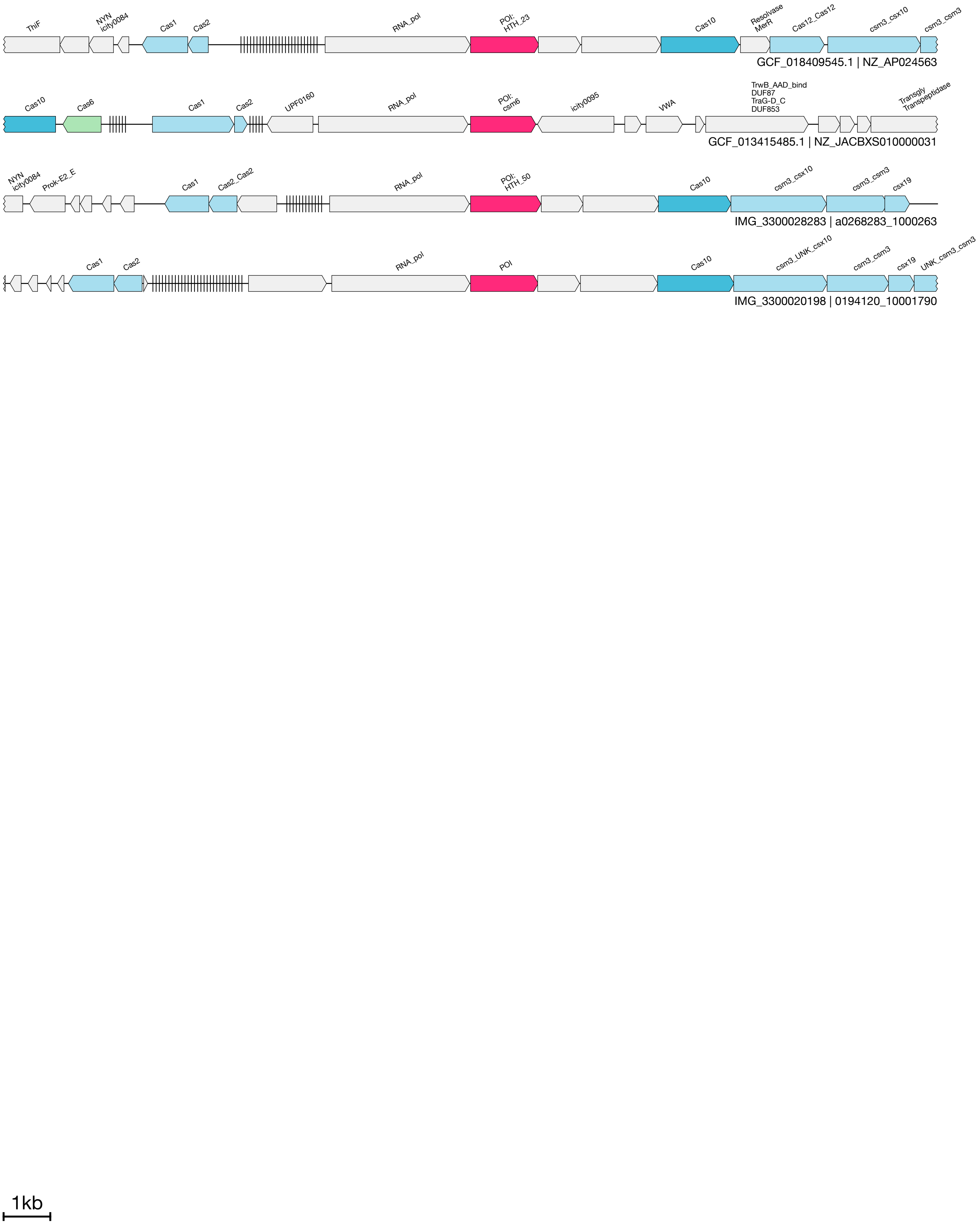

BC

UAS-59 **(CARF_CYTH_HD)** 3 / 5.1

CARF IMG_3300002498&&TOLCLC_10008146&&3727_6961_-1 extraction

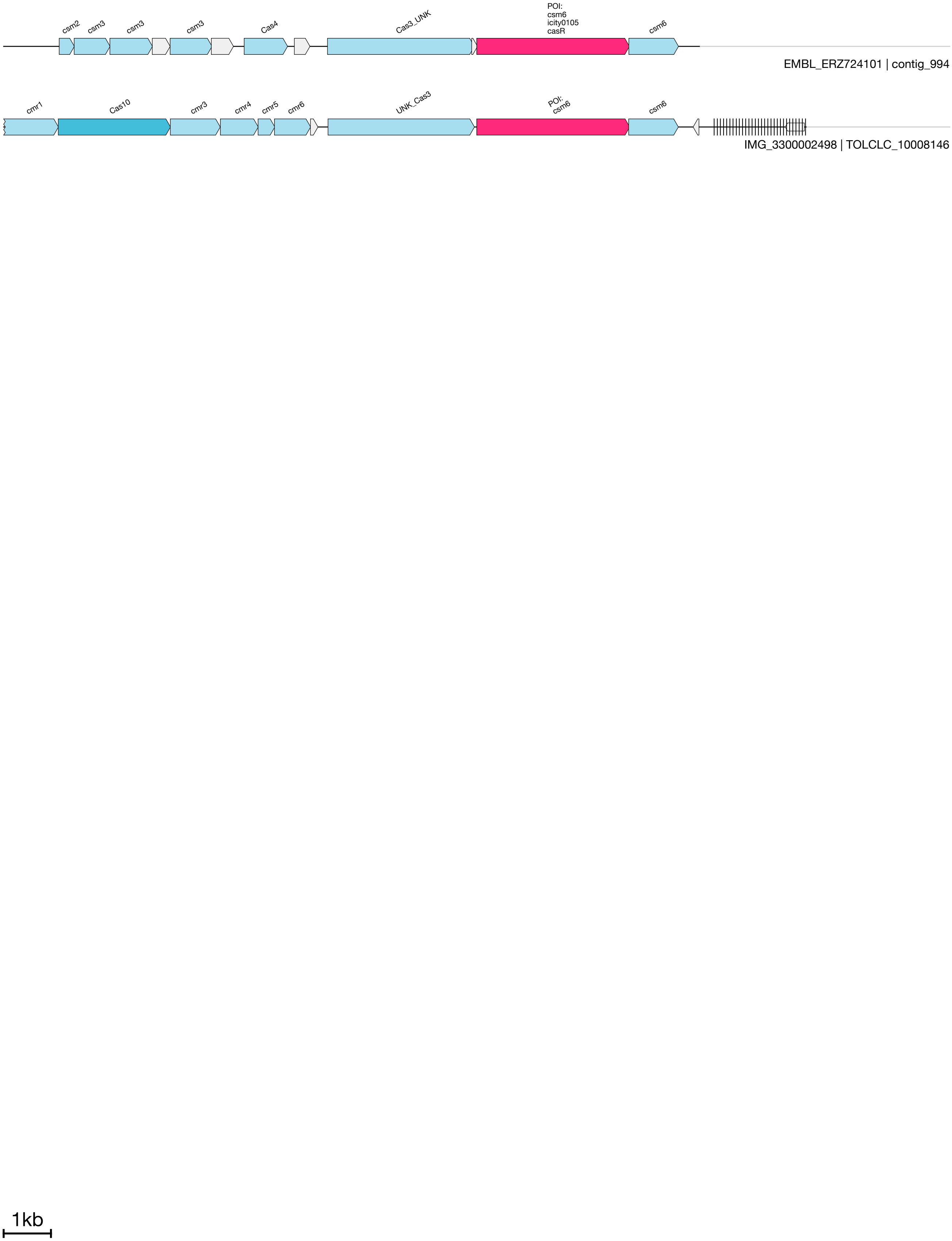

BD

UAS-60 **(CARF_SIR2)** N/A

CARF IMG_3300009523&&a0116221_1002542&&6420_8025_-1_loci_to_order

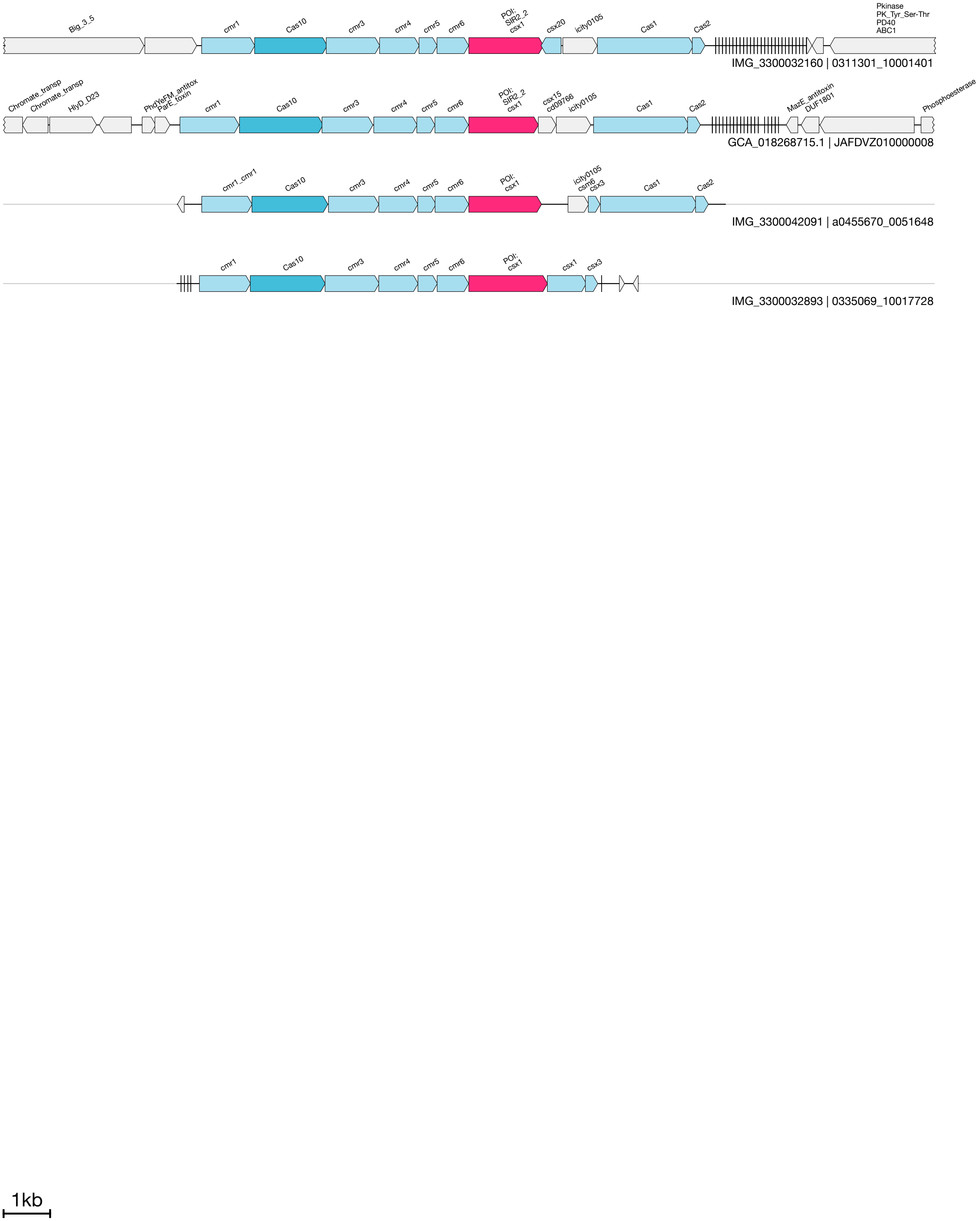

BE

UAS-61 **(CARF_TIR_Mrr)** 11 / 15.3

CARF IMG_3300020198&&0194120_10015339&&5702_6689_1 extraction

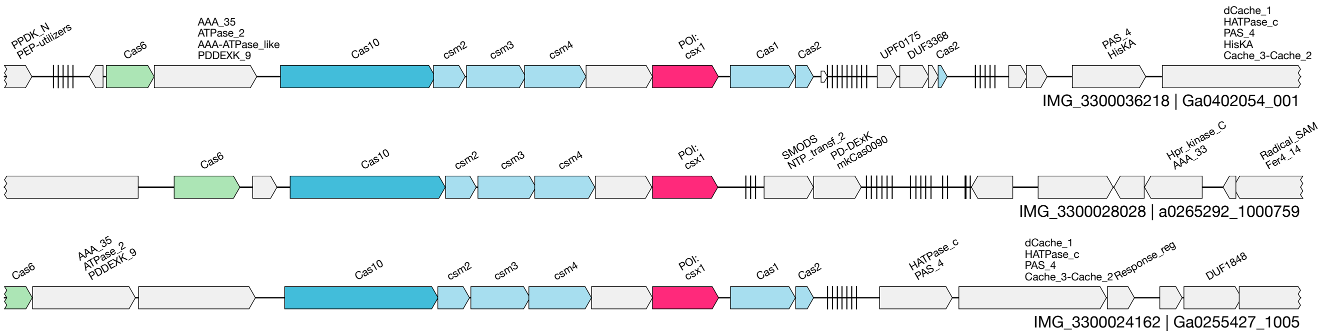

1kb

BF

UAS-62
CARF

(csx1 + Phage RNAPol)
IMG_3300026484&&a0256837_1008848&&1106_4061_-1

5 / 12.2

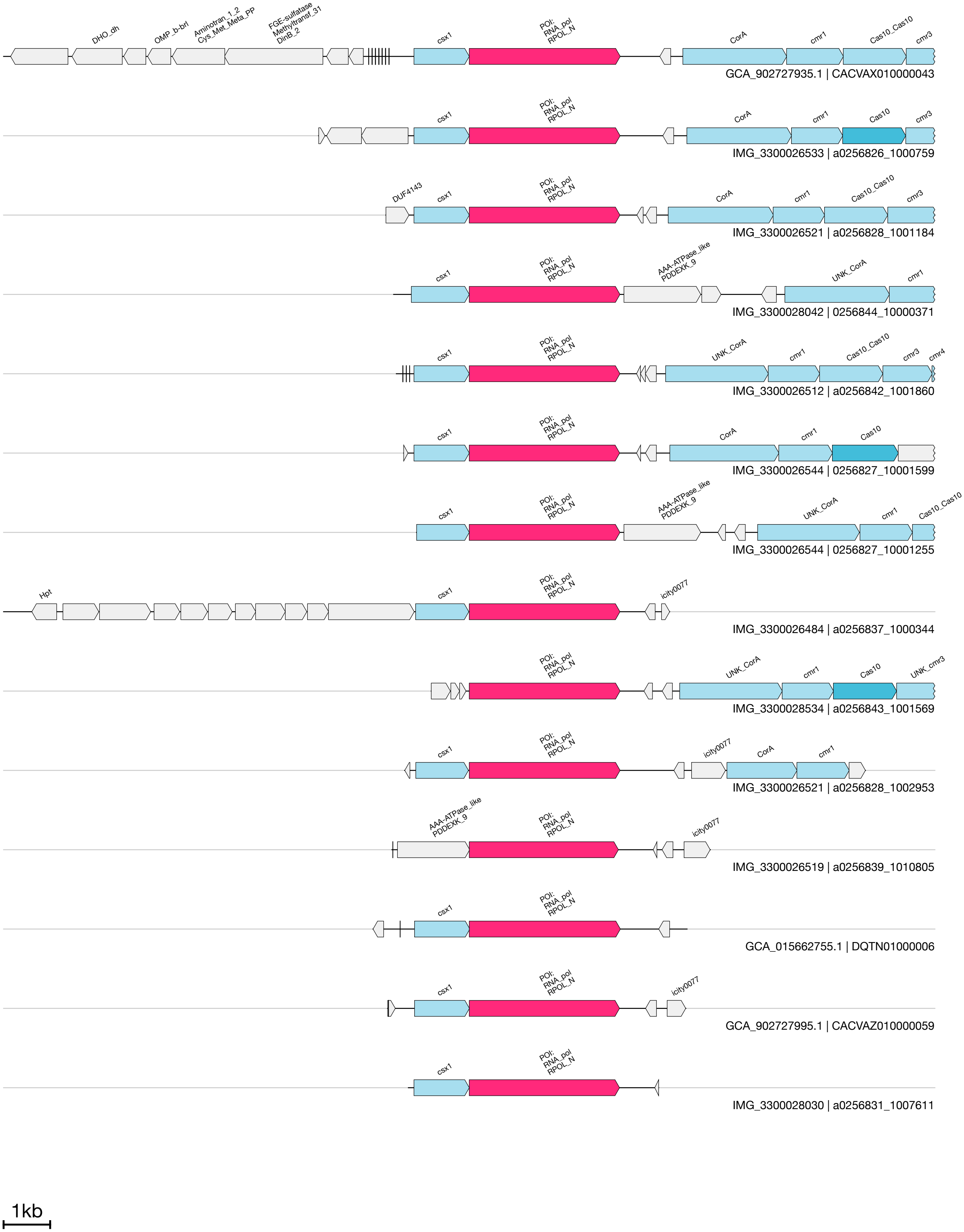

BG

UAS-63
CARF

(CARF_2p_deoxyribosyltransferase)
IMG_3300031539&&0307380_10010365&&4440_5463_-1

5 / 7.7

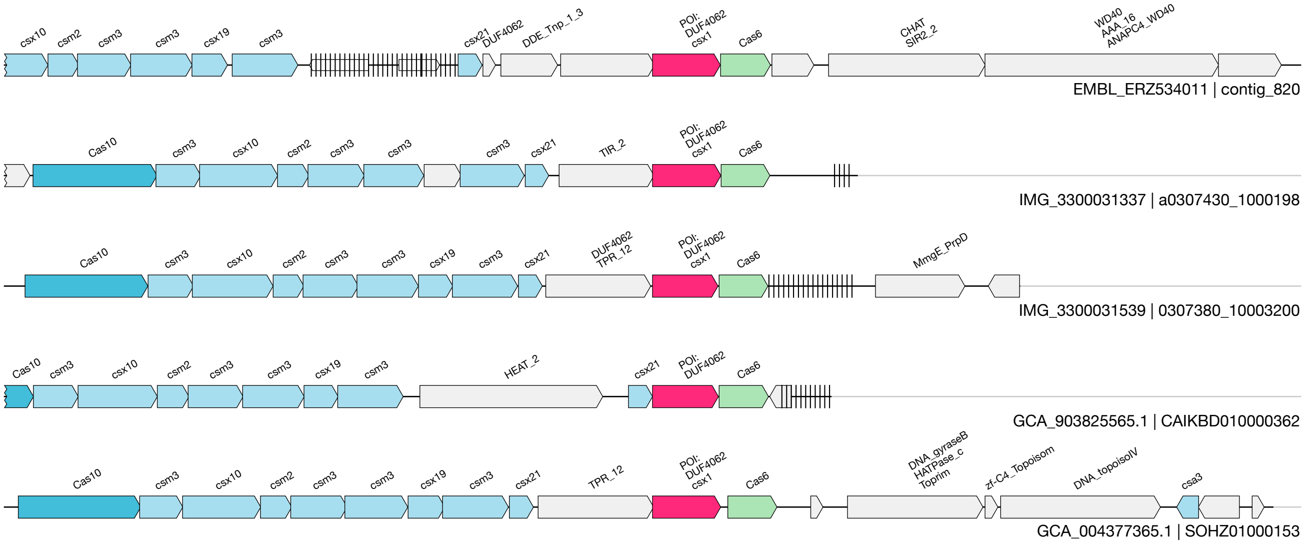

1kb

BH

UAS-64 **(CARF_EAD7)** N/A
CARF IMG_3300031566&&0307378_10017289&&164_1499_1 extraction

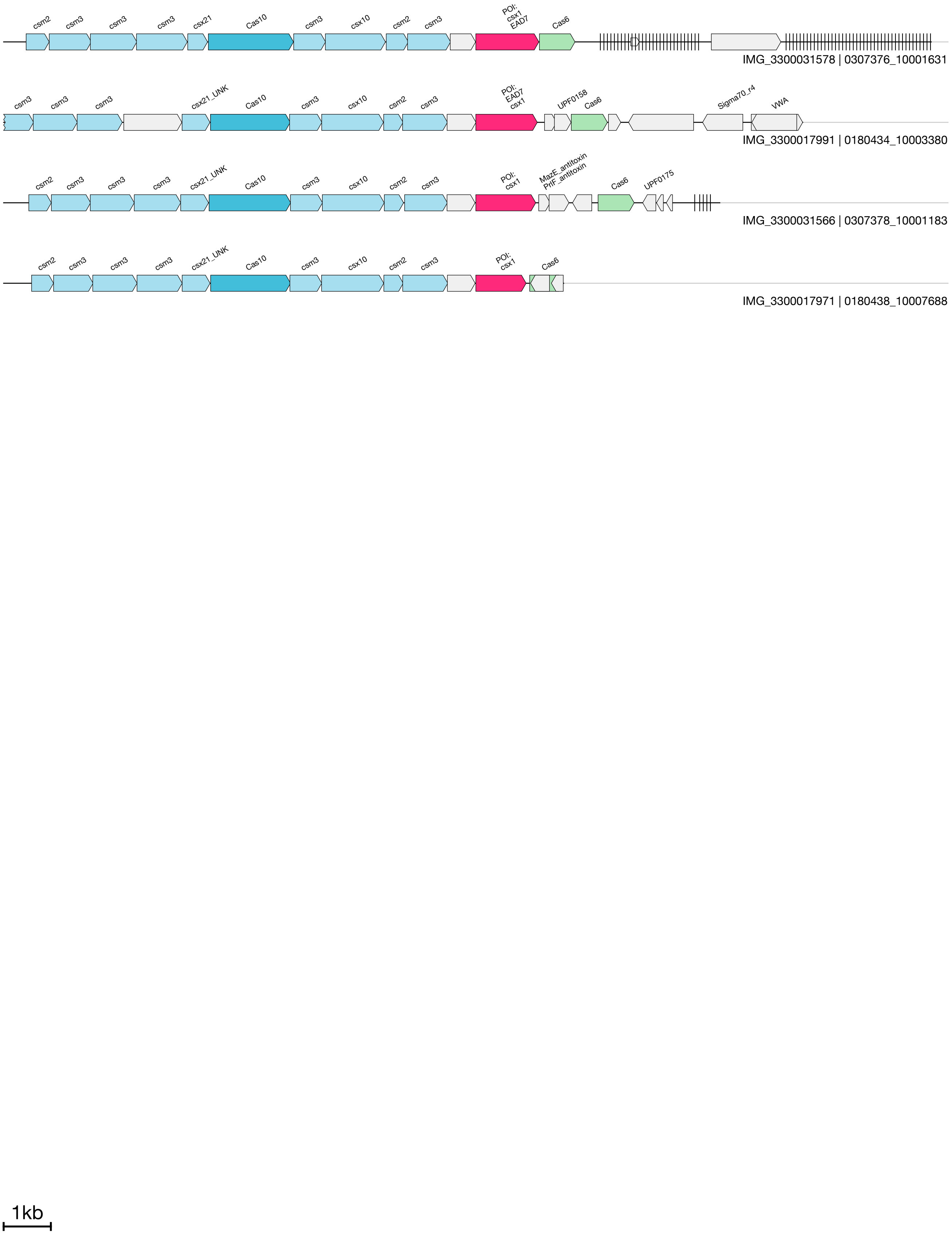

BI

UAS-65
CARF

(CARF_5p_nucleotidase)
IMG_3300031994&&0310691_10021322&&2165_3863_1 extraction

6 / 6.6

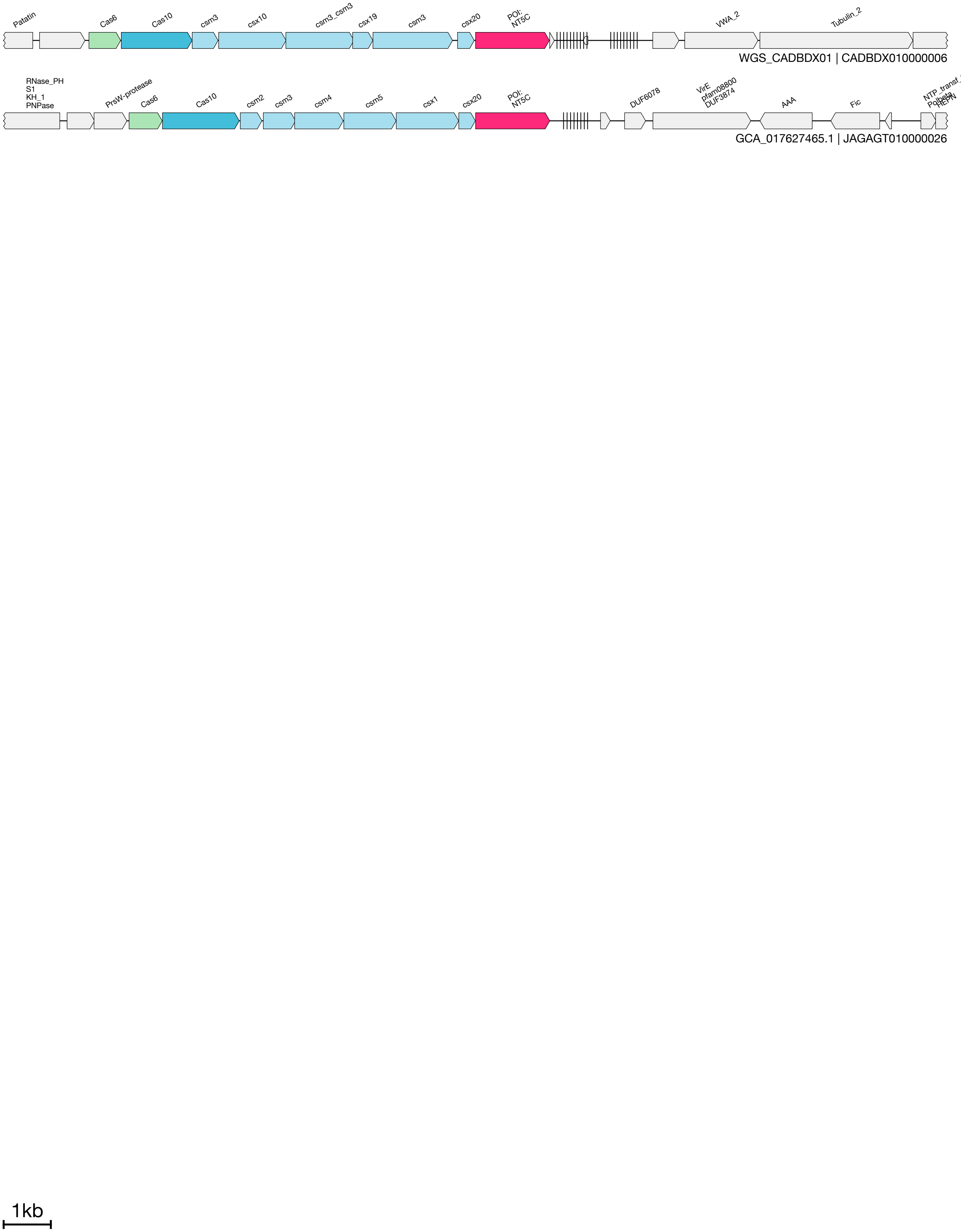

BJ

UAS-66 **(SAVED_APH_TCAD9)** 4 / 4.5
CARF IMG_3300034111&&a0335063_0013299&&1300_3253_1

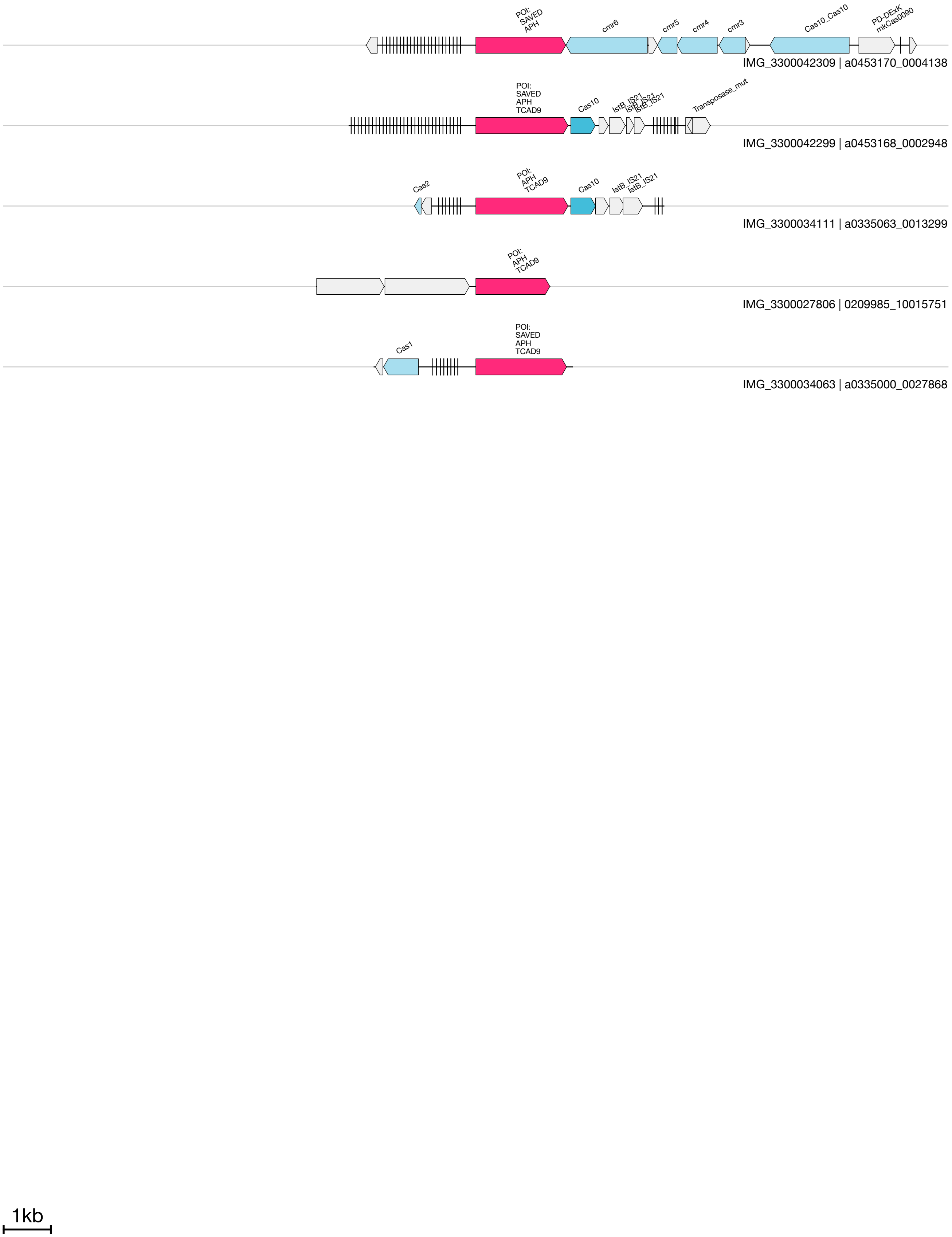

BK

UAS-67 **(Cutinase_Lipase_SAVED)** 4 / 4.7

CARF IMG_3300042939&&a0453142_0007685&&8027_9725_-1

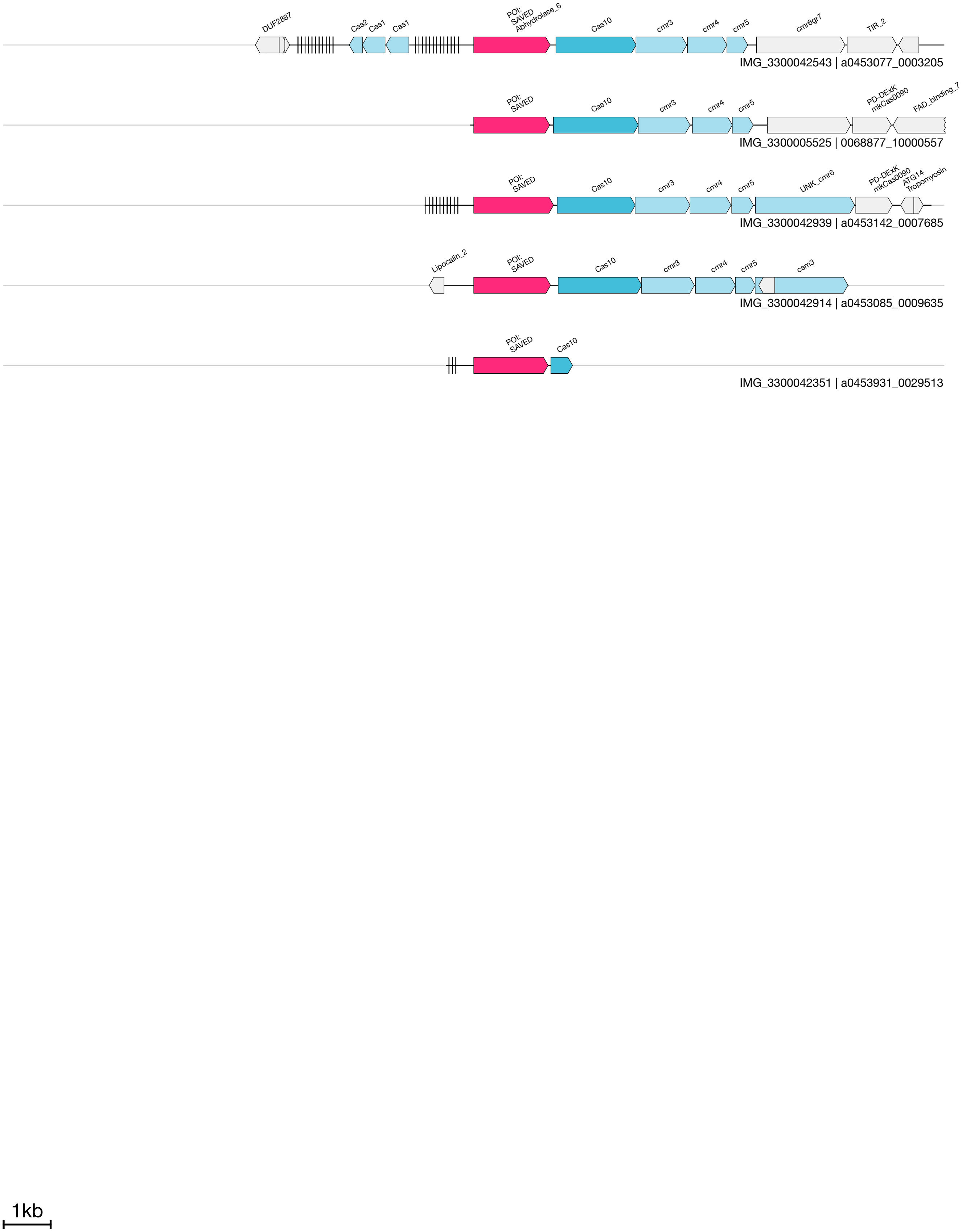

BL

UAS-68
CARF

(SMODS + SAVED CHAT)
IMG_3300046790&&a0495499_0000077&&7424_8333_1

N/A

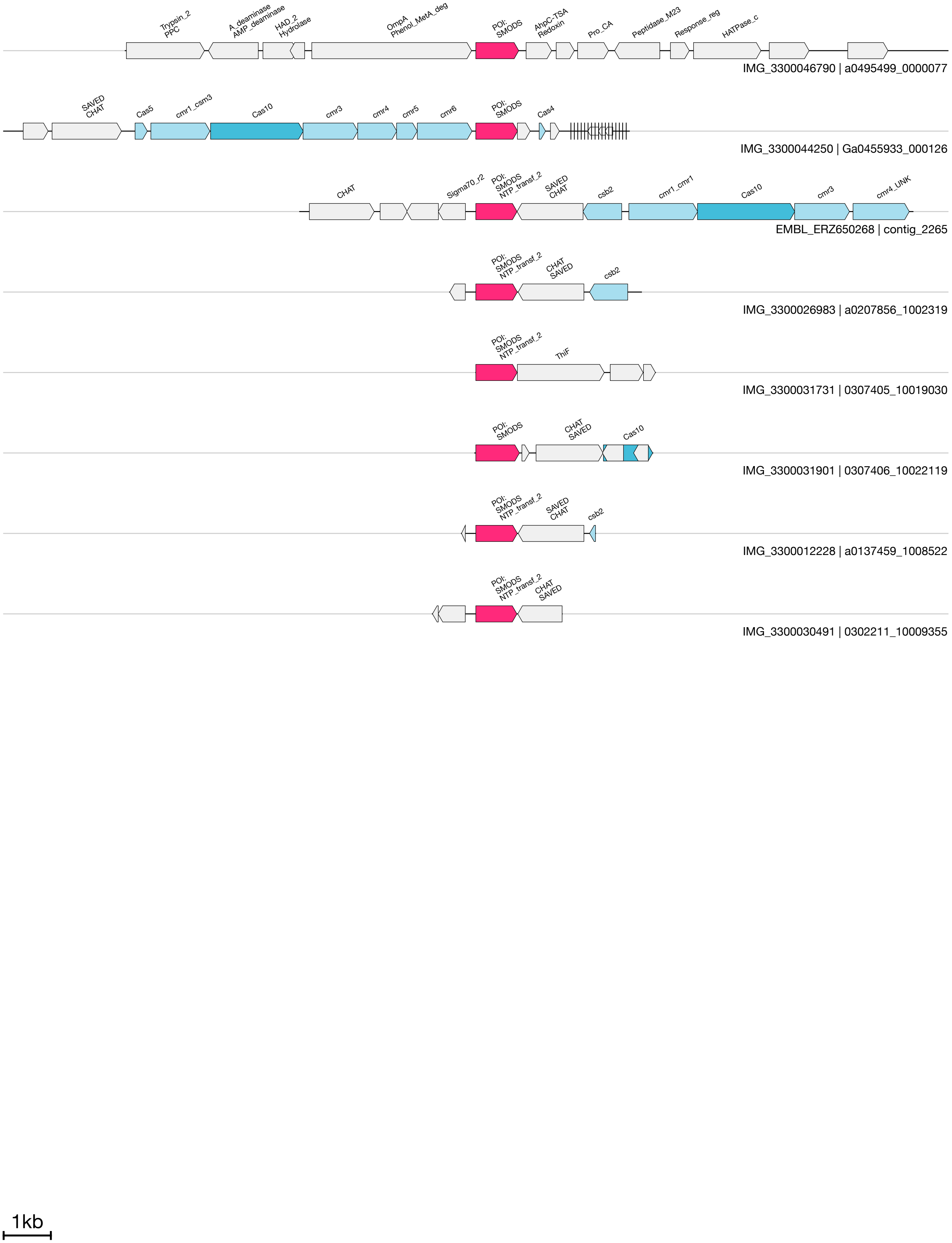

BM

UAS-69
Auxiliary

(methyltransferase)
EMBL_ERZ1030305&&contig_263&&8899_9757_1

3 / 7.0

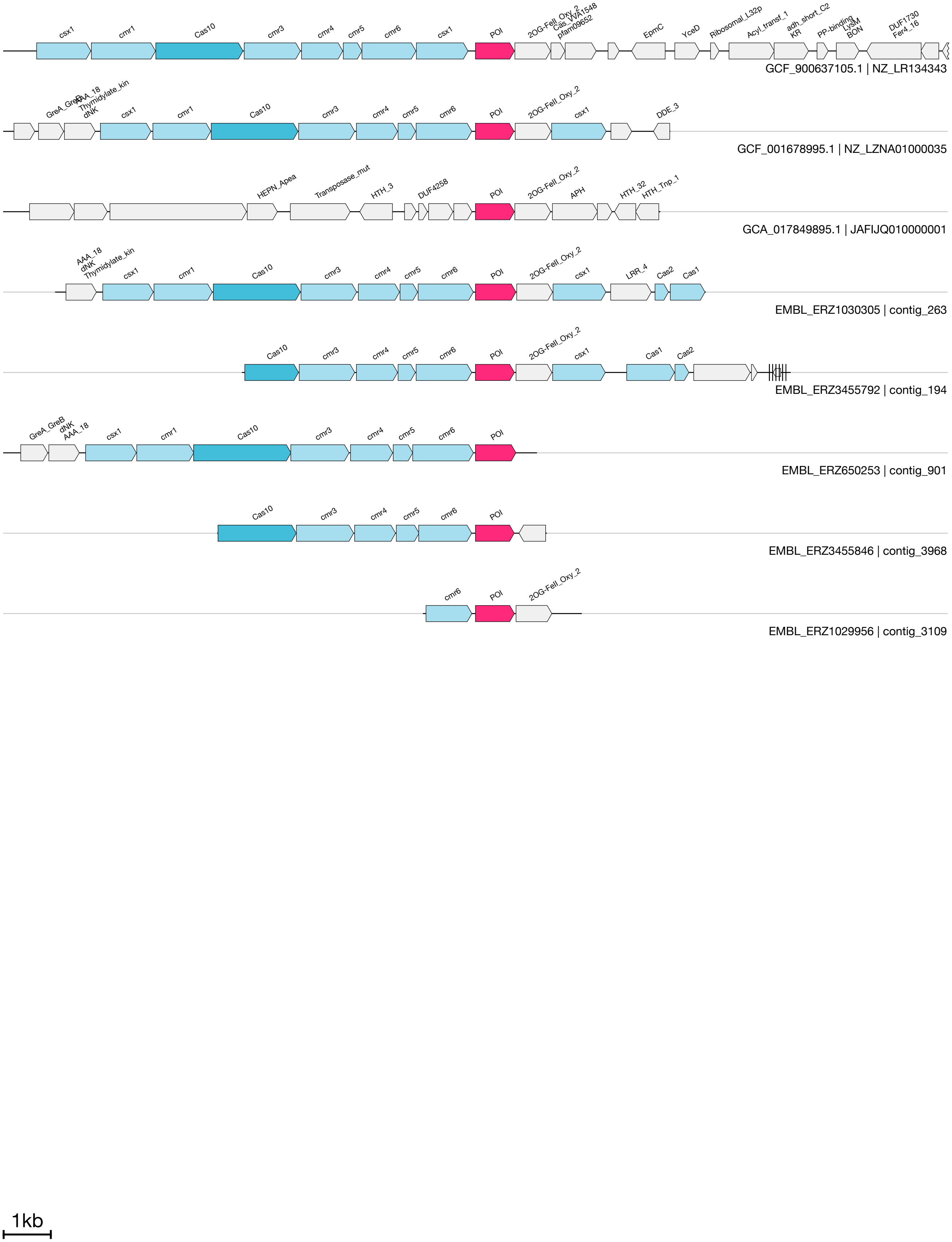

BN

UAS-70 **(vATP-synt_E)** 9 / 9.3

Auxiliary EMBL_ERZ1699236&&contig_1829&&258_2607_-1

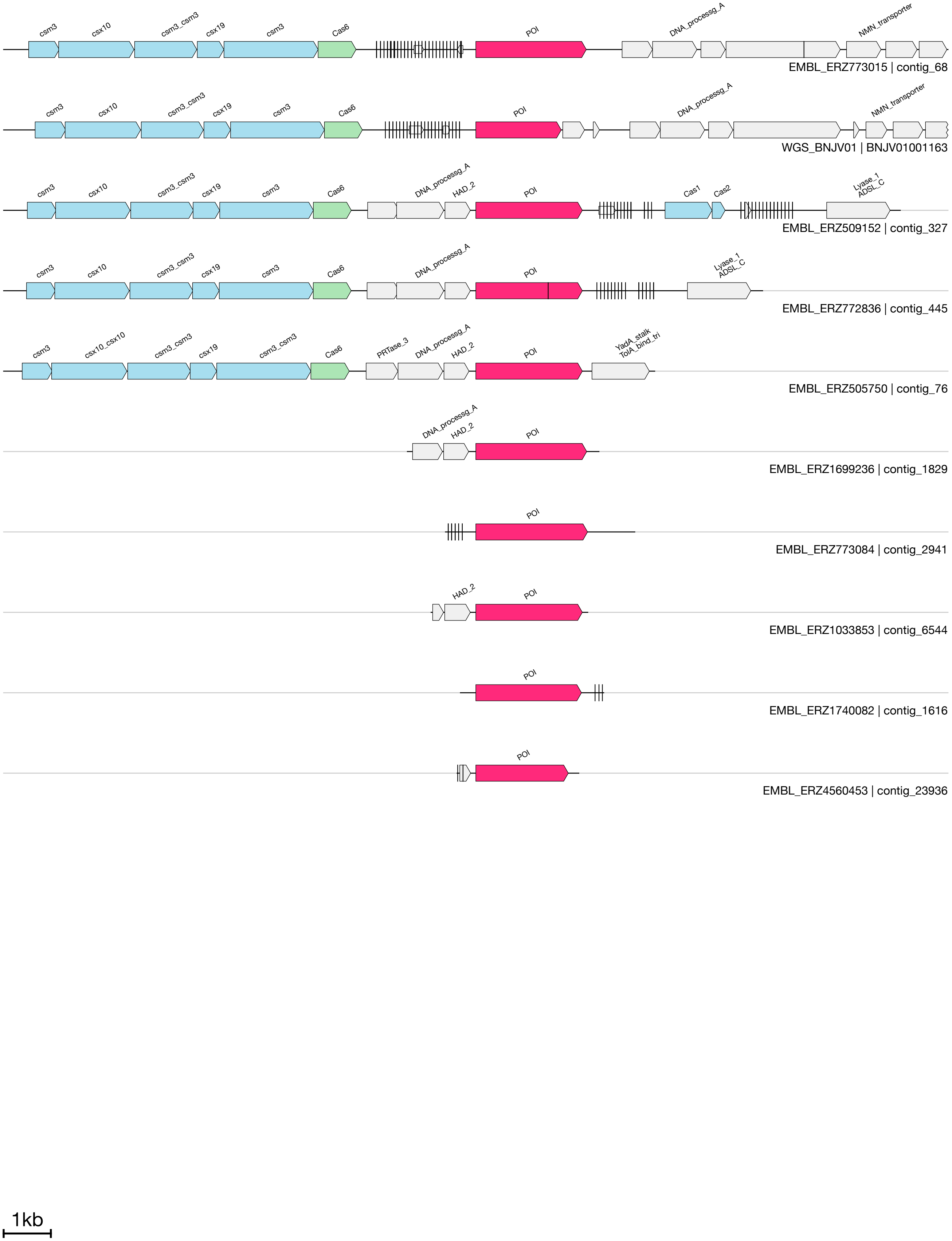

BO

UAS-71 **(NERD)** 4 / 4.7

Auxiliary EMBL_ERZ1764562&&contig_378&&16539_17331_1

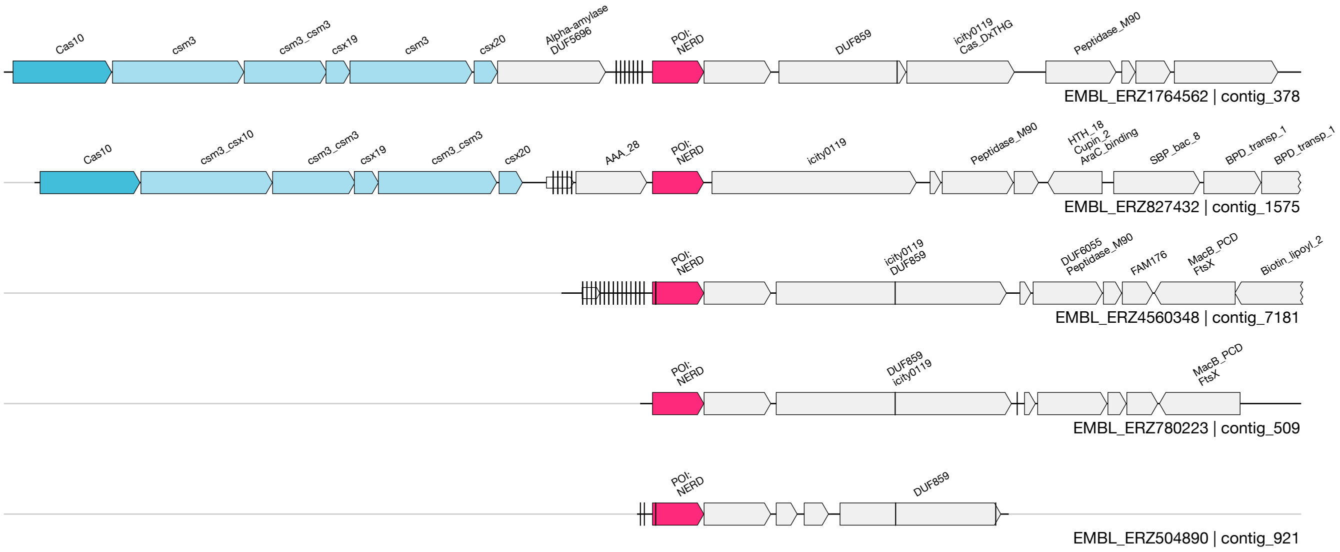

1kb

BP

UAS-72
Auxiliary

(multi-gene system including greA greB)
EMBL_ERZ3455816&&contig_9406&&2751_3312_1

20 / 26.7

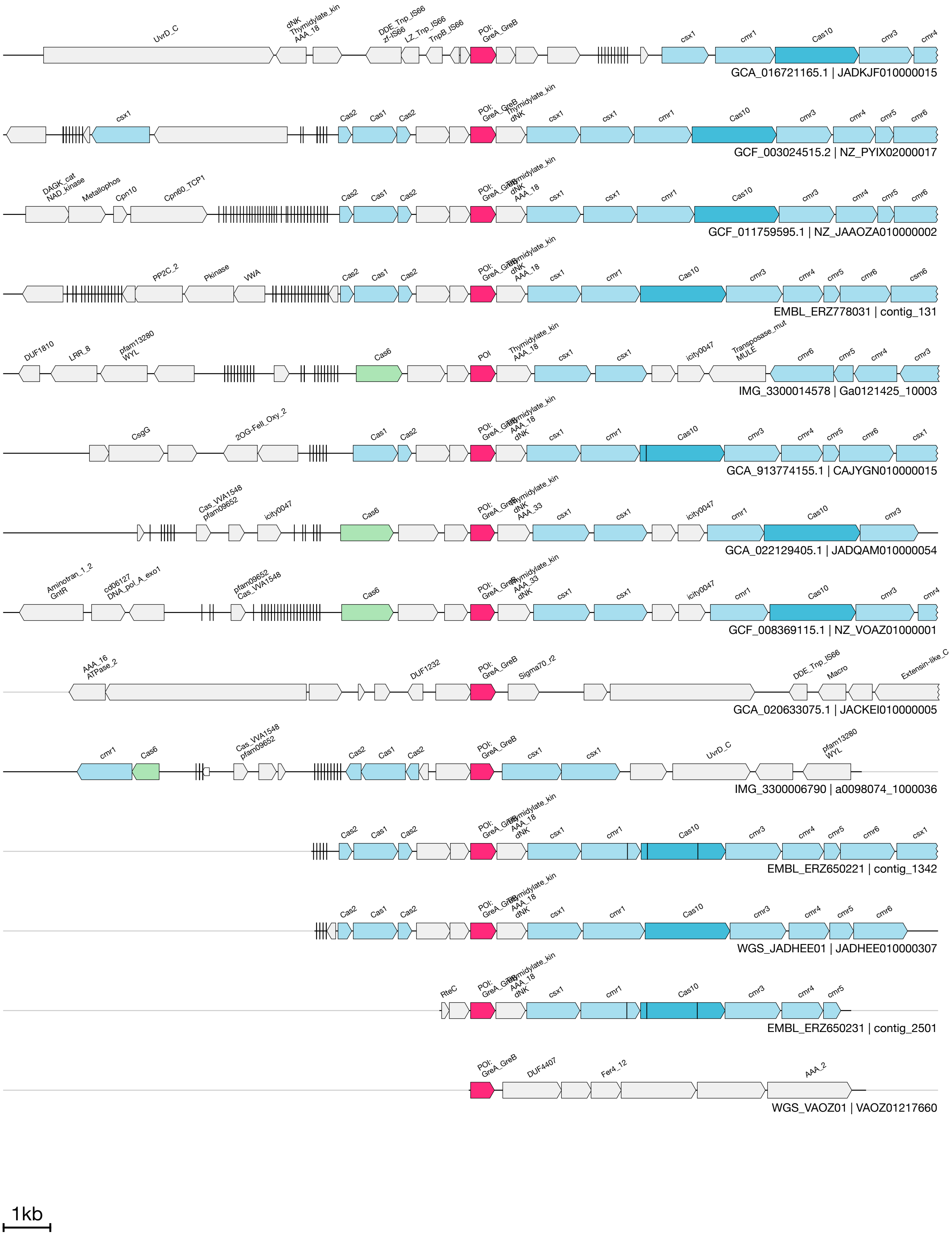

BQ

UAS-73
Auxiliary

(Mrr nuclease)
EMBL_ERZ405284&&contig_1793&&4535_5441_1

7 / 10.5

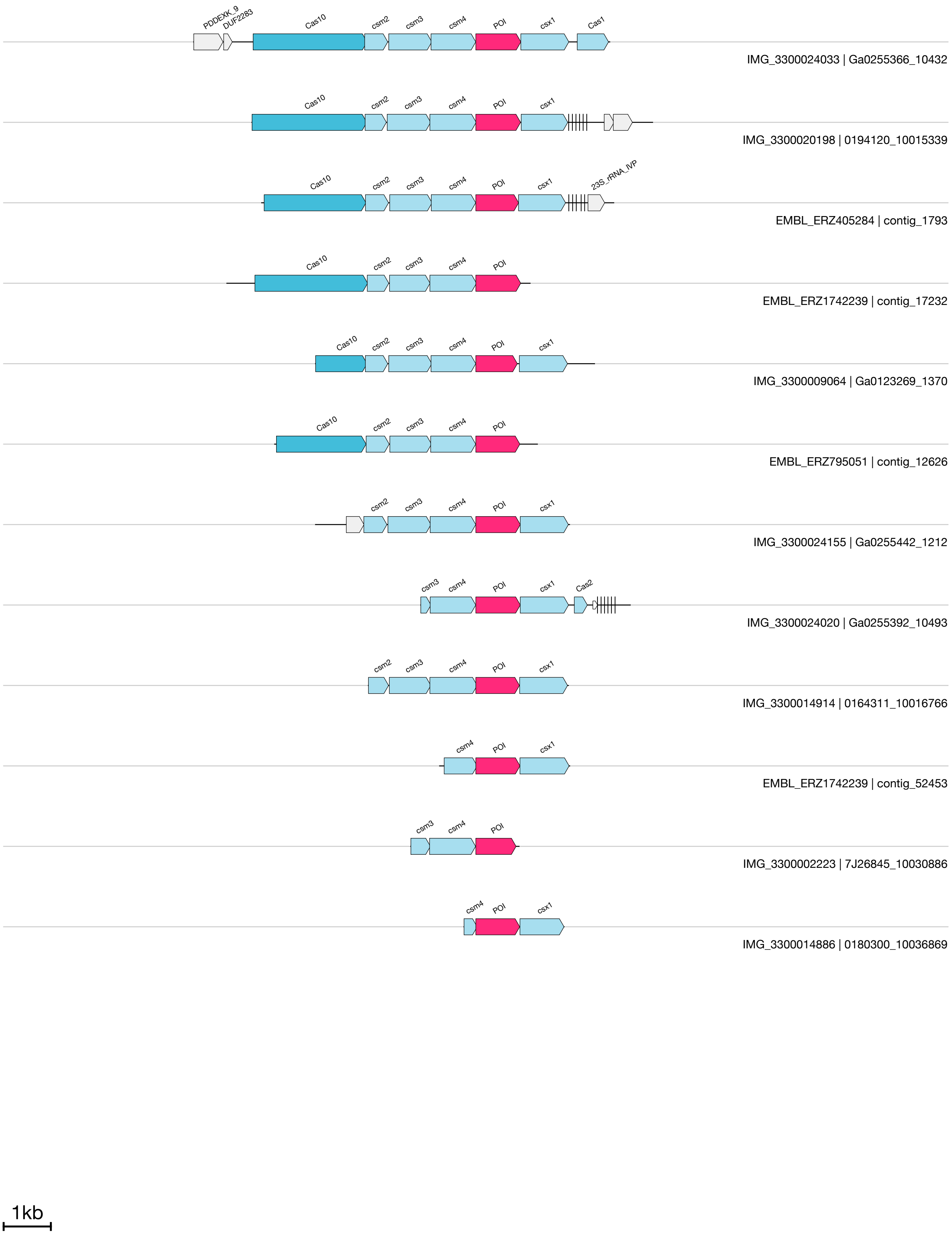

BR

UAS-74 **(PKinase)** 2 / 3.4

Auxiliary EMBL_ERZ4560480&&contig_4482&&7569_10323_-1

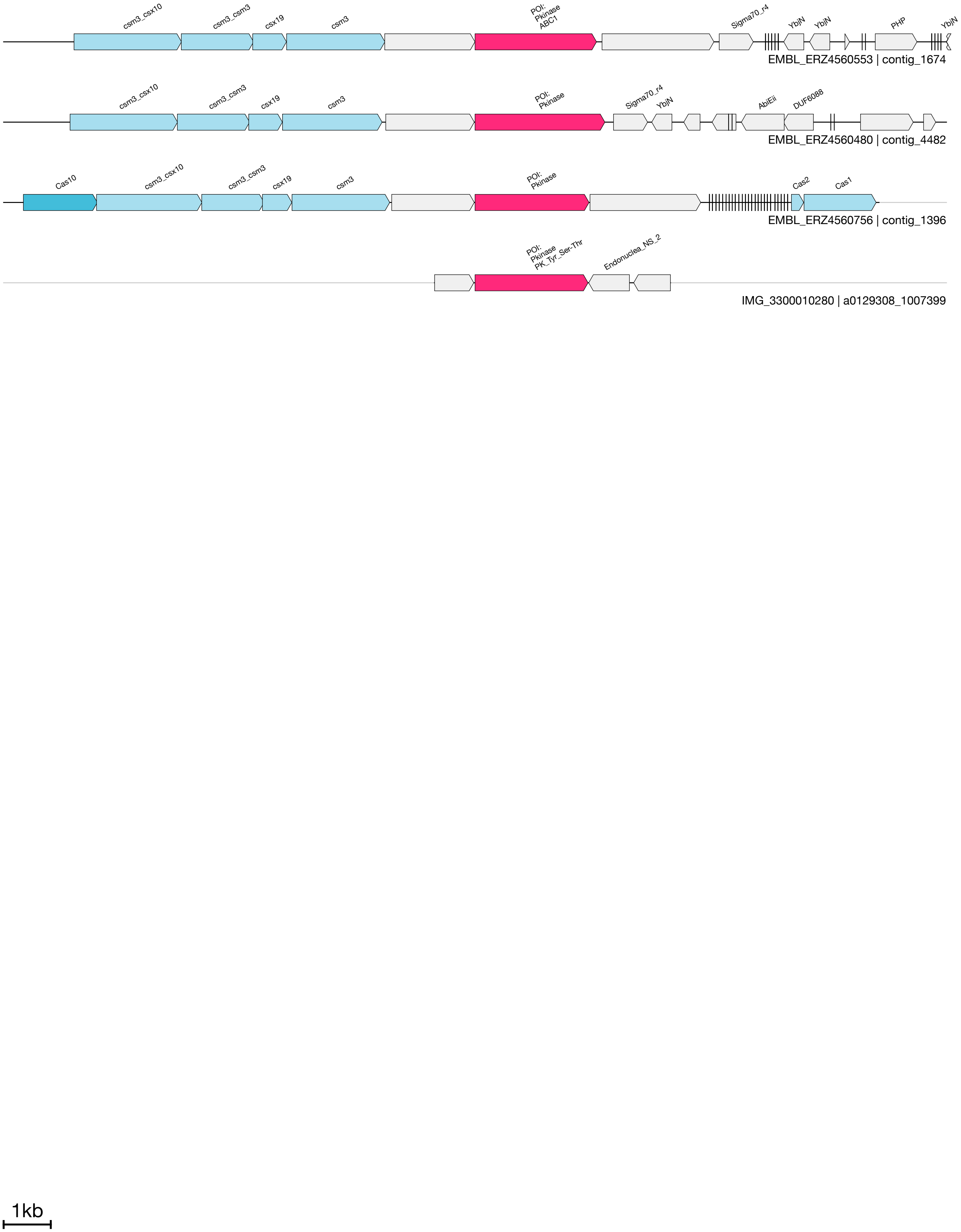

BS

UAS-75
Auxiliary

(Sigma_factor + Csx1 + large Csx1)
EMBL_ERZ4560494&&contig_2628&&7202_8435_1

7 / 7.5

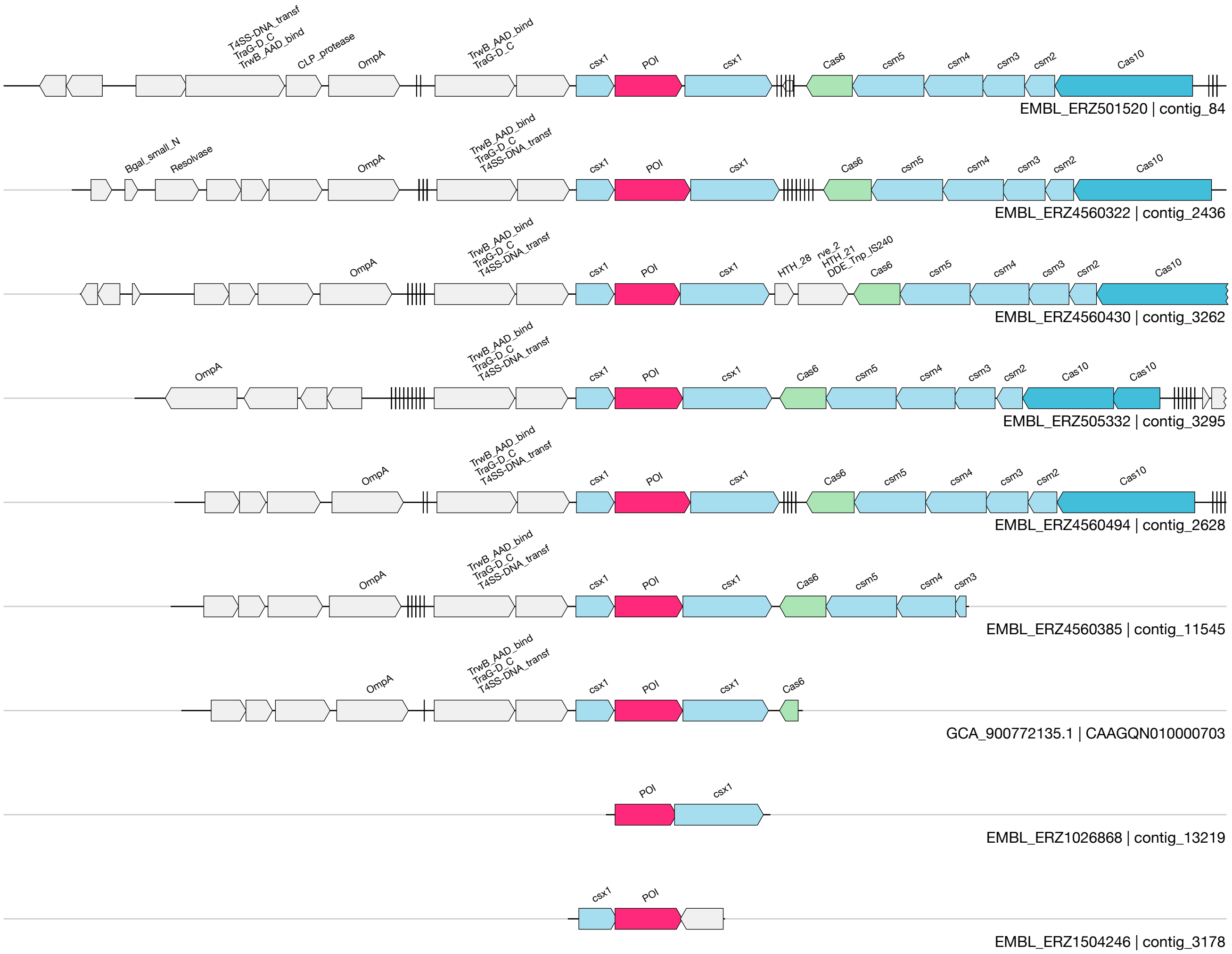

1kb

BT

UAS-76
Auxiliary

(HTH _ M78 peptidase)
EMBL_ERZ4560656&&contig_7640&&76_1108_-1

41 / 53.8

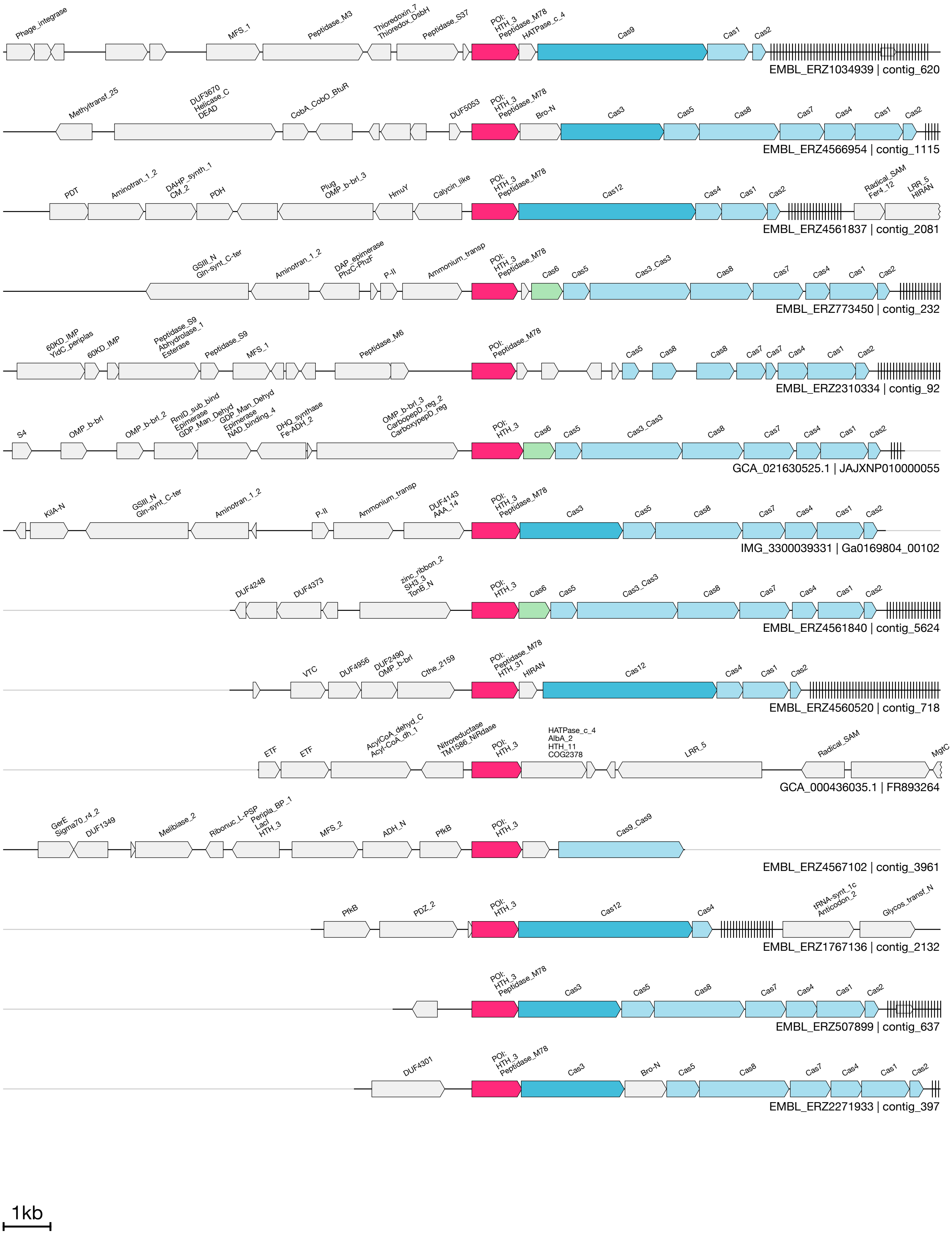

BU

UAS-77
Auxiliary

(DUF2283)
EMBL_ERZ584659&&contig_1773&&510_1521_-1

3 / 5.5

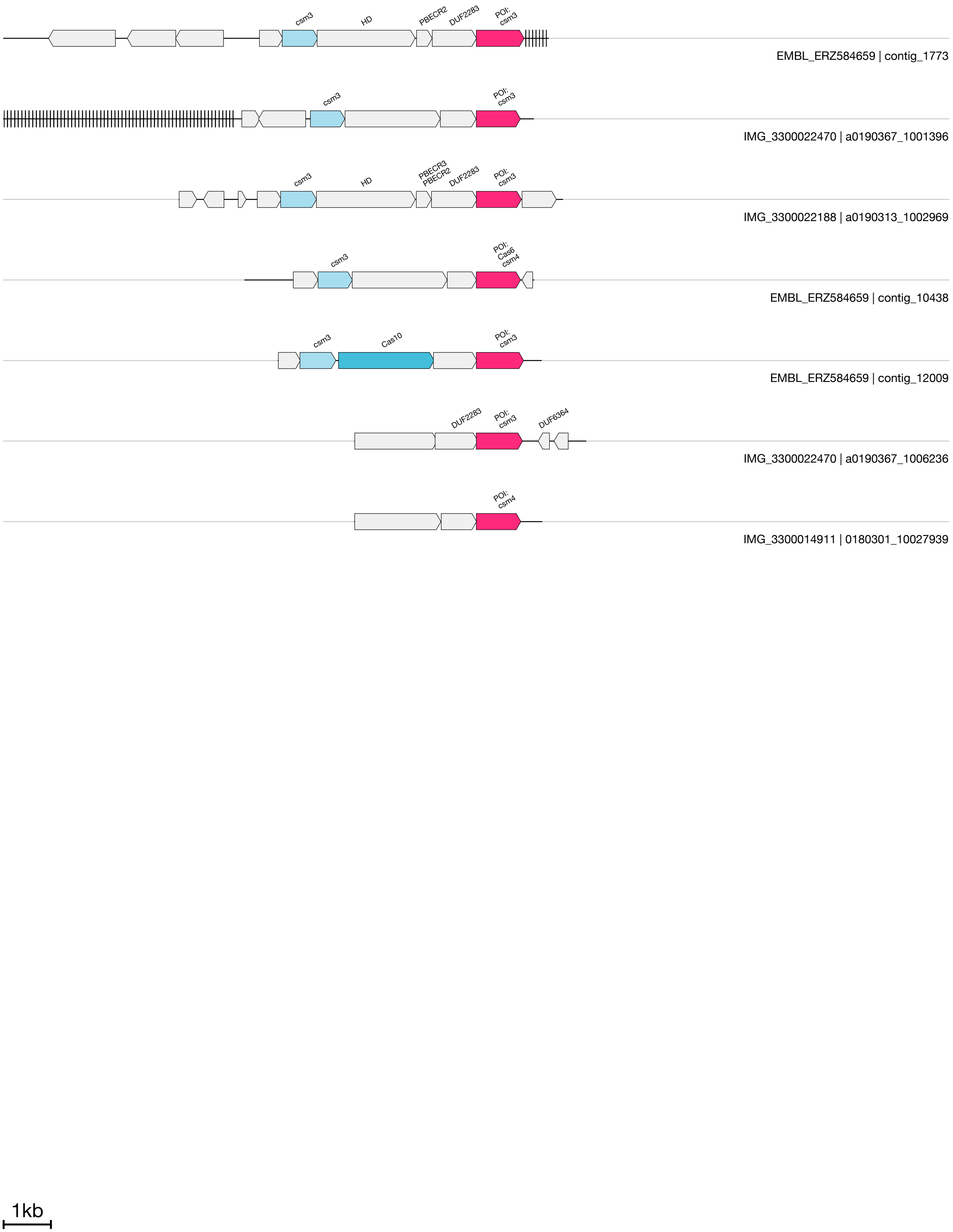

BV

UAS-78
Auxiliary

(Nif11)
EMBL_ERZ650341&&contig_14933&&186_501_1

7 / 7.7

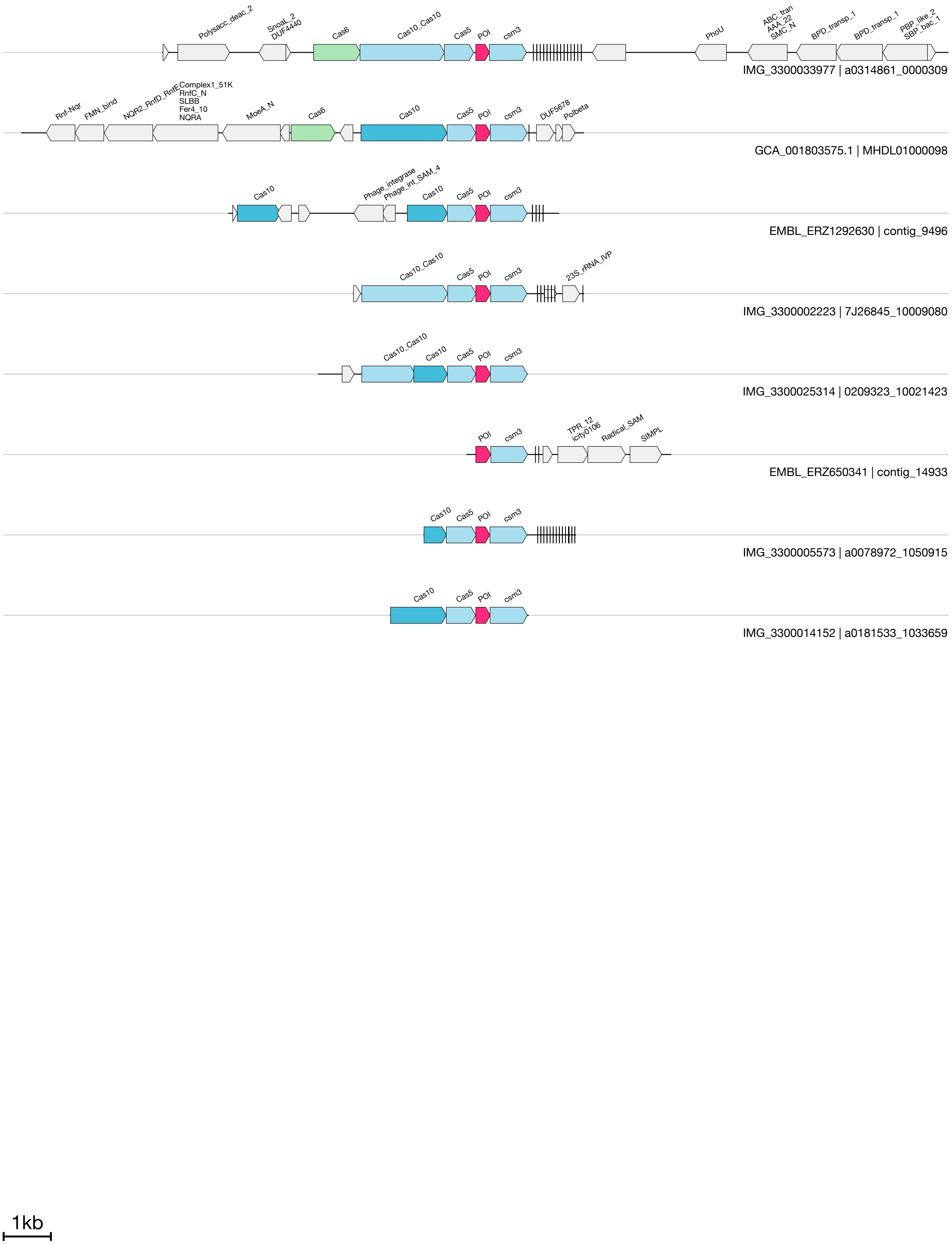

BW

UAS-79 **(XRE HTH regulator operonized with Cas effectors)** 8 / 8.9

Auxiliary EMBL_ERZ653703&&contig_1622&&2425_3673_-1

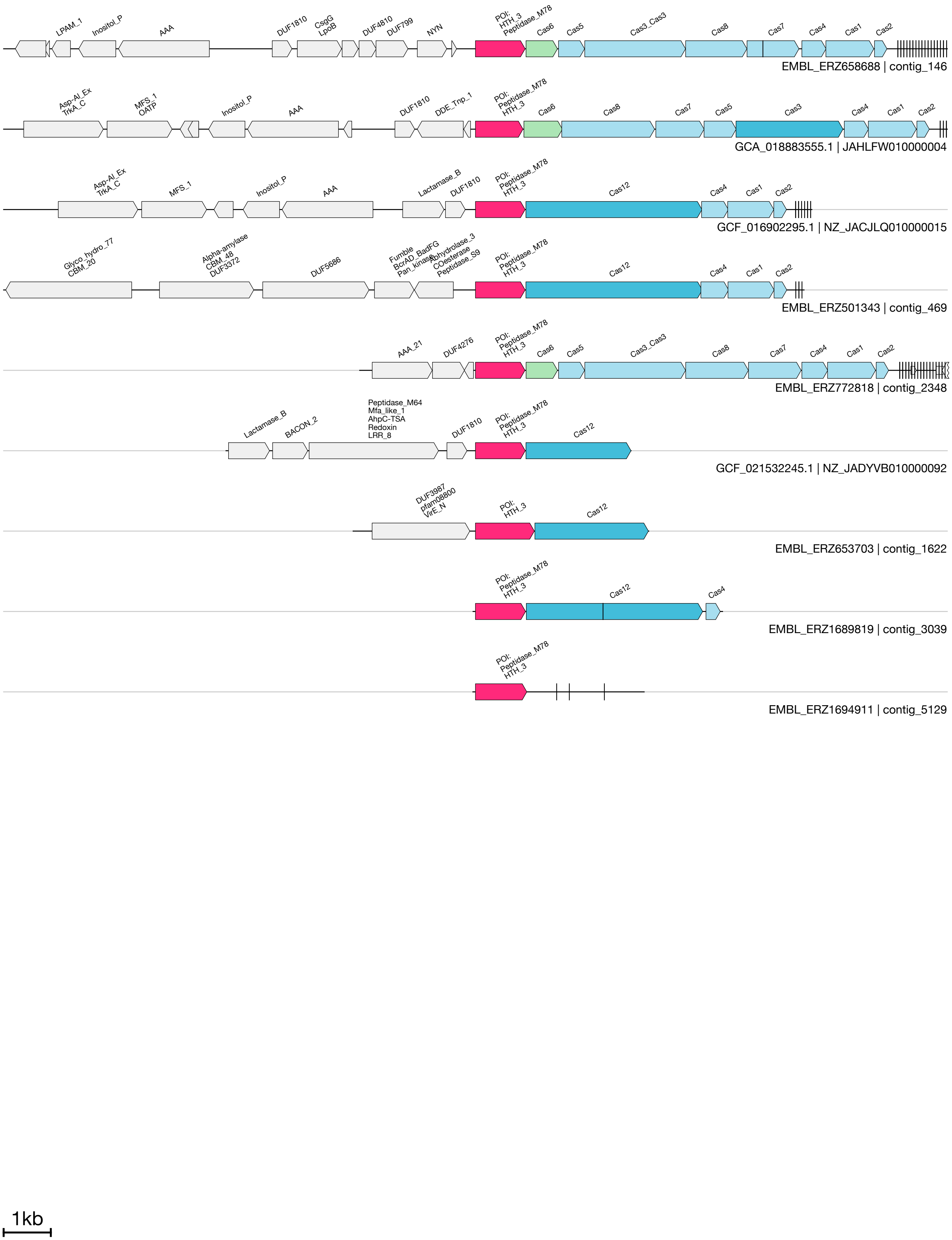

BX

UAS-80 **(RepA replication)** 5 / 5.1

Auxiliary EMBL_ERZ724078&&contig_5708&&5799_7854_-1

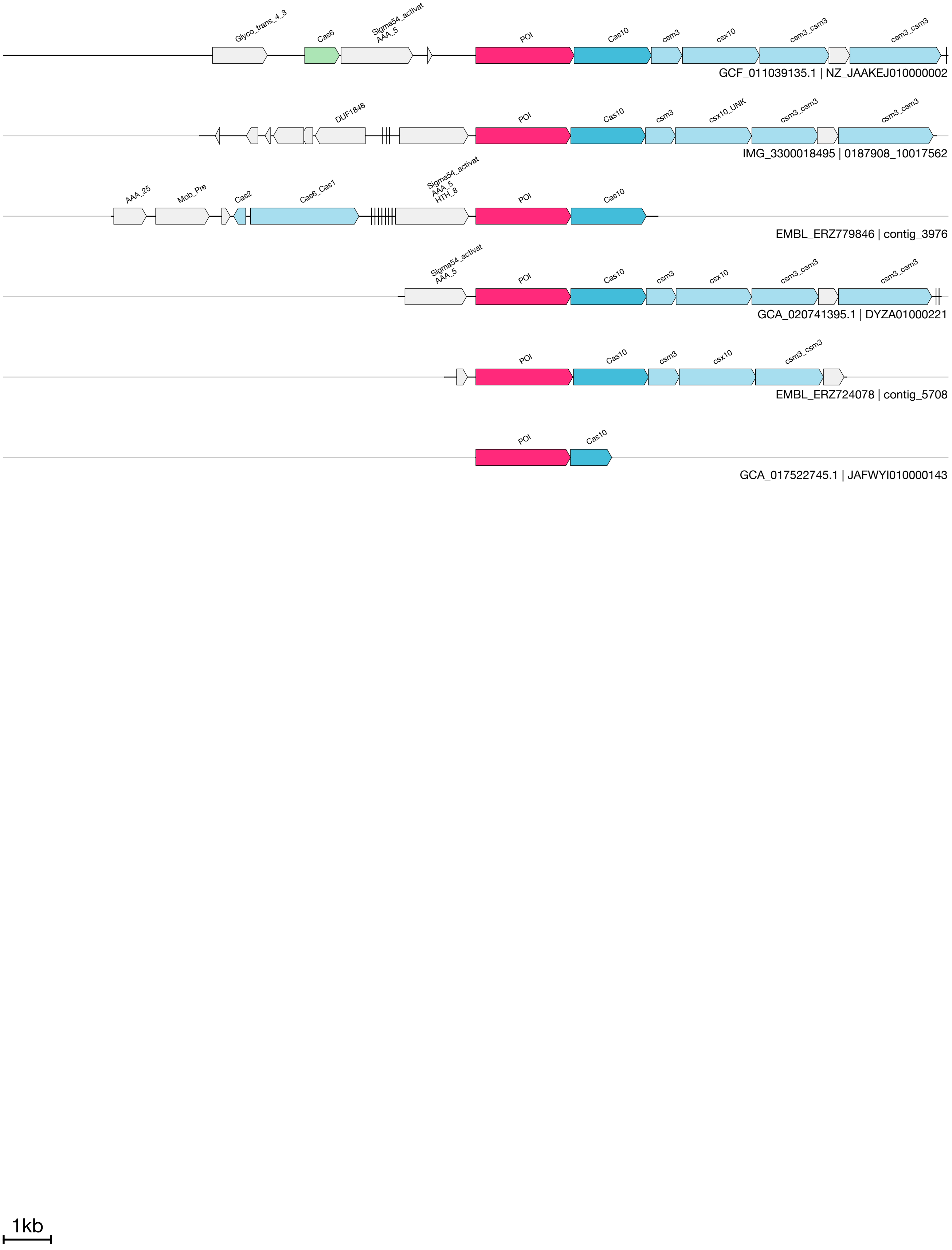

BY

UAS-81
Auxiliary

(DUF4145)
EMBL_ERZ724507&&contig_6357&&940_1633_-1

3 / 3.0

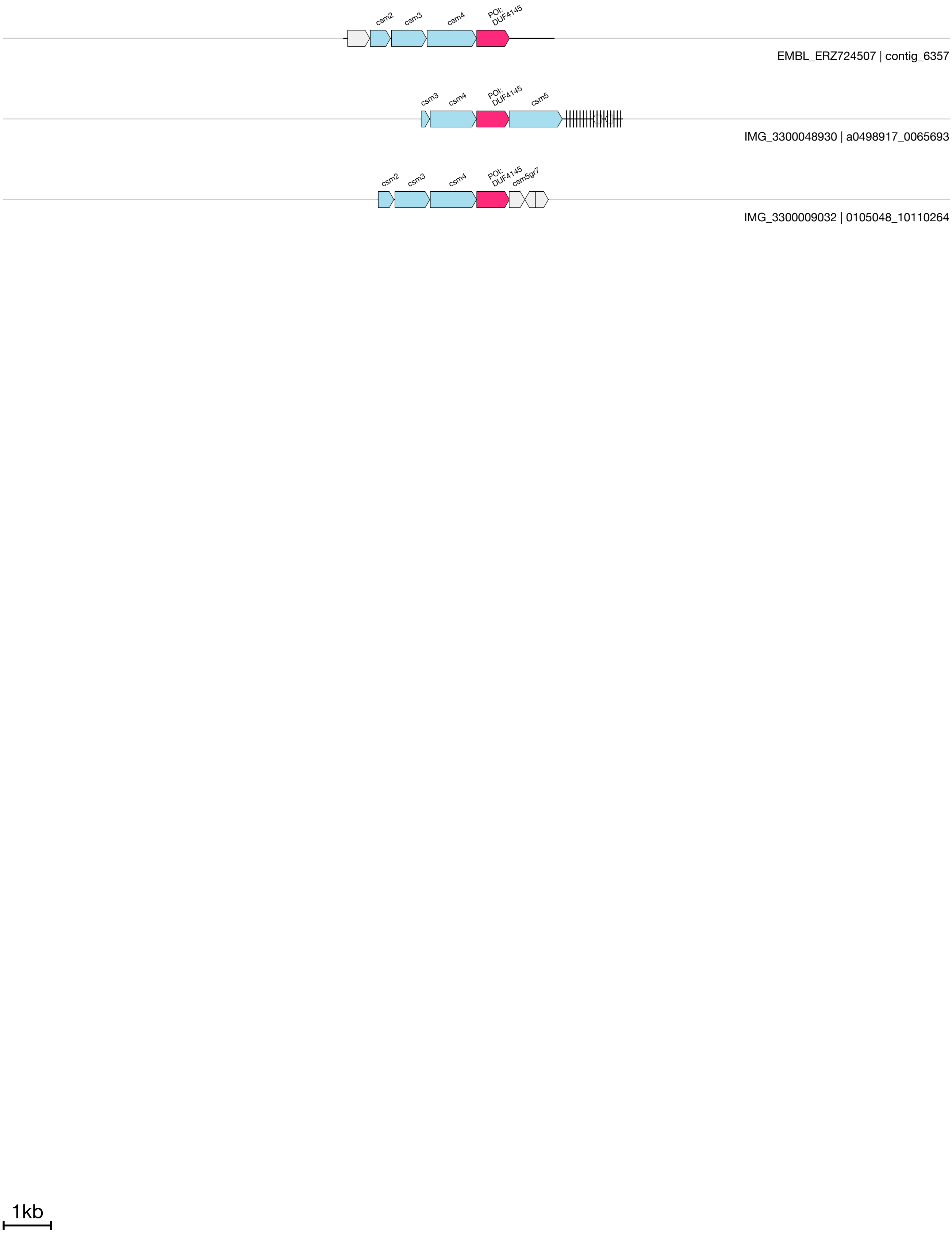

BZ

UAS-82
Auxiliary

(pentapeptide + unknown domain)
EMBL_ERZ772753&&contig_7251&&225_1815_-1

4 / 7.6

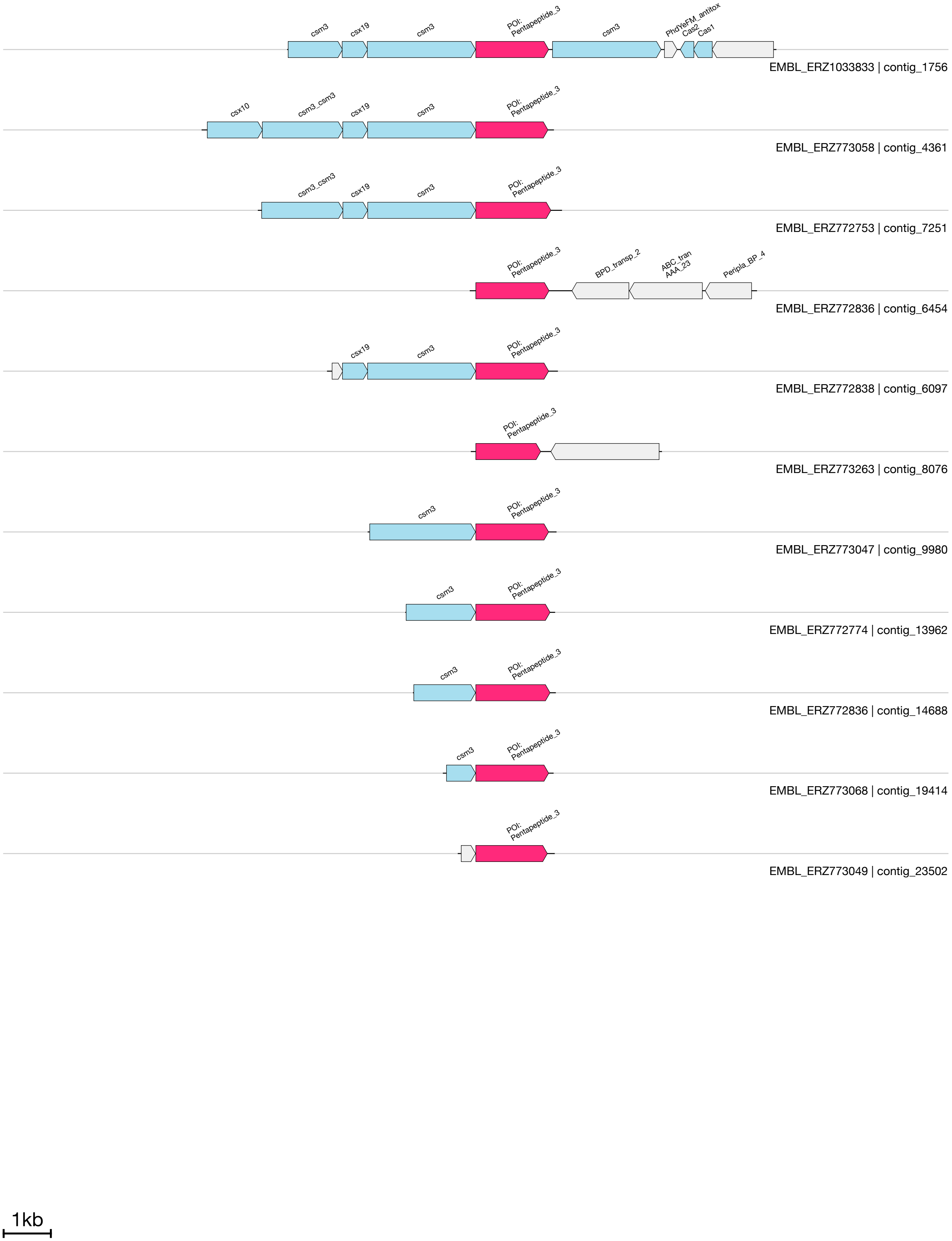

CA

UAS-83
Auxiliary

(Recep domain + 4 unknown genes)
EMBL_ERZ773219&&contig_530&&18381_19626_1

8 / 8.0

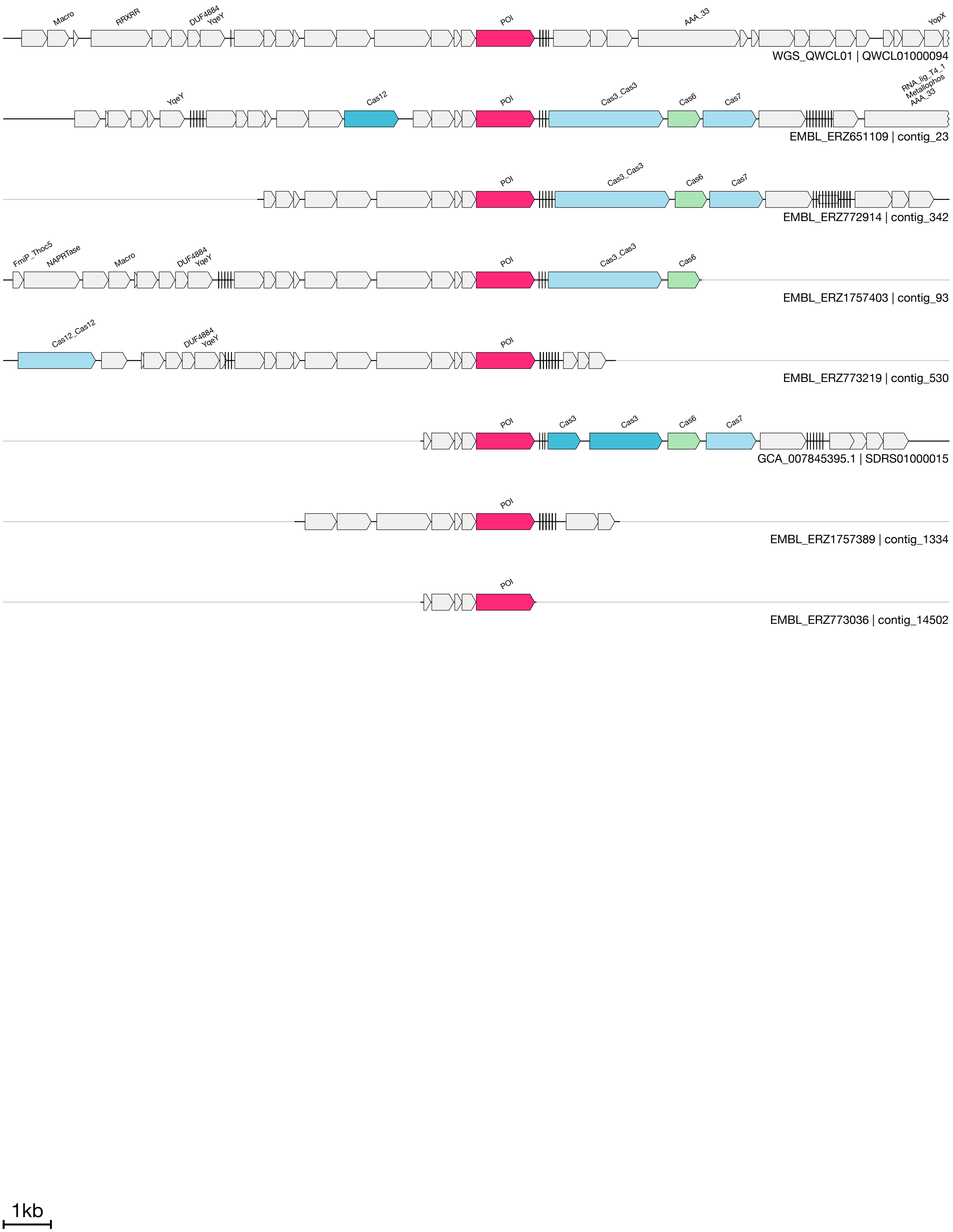

CB

UAS-84 **(MazF)** 2 / 5.3
Auxiliary EMBL_ERZ773360&&contig_3930&&5259_6435_-1

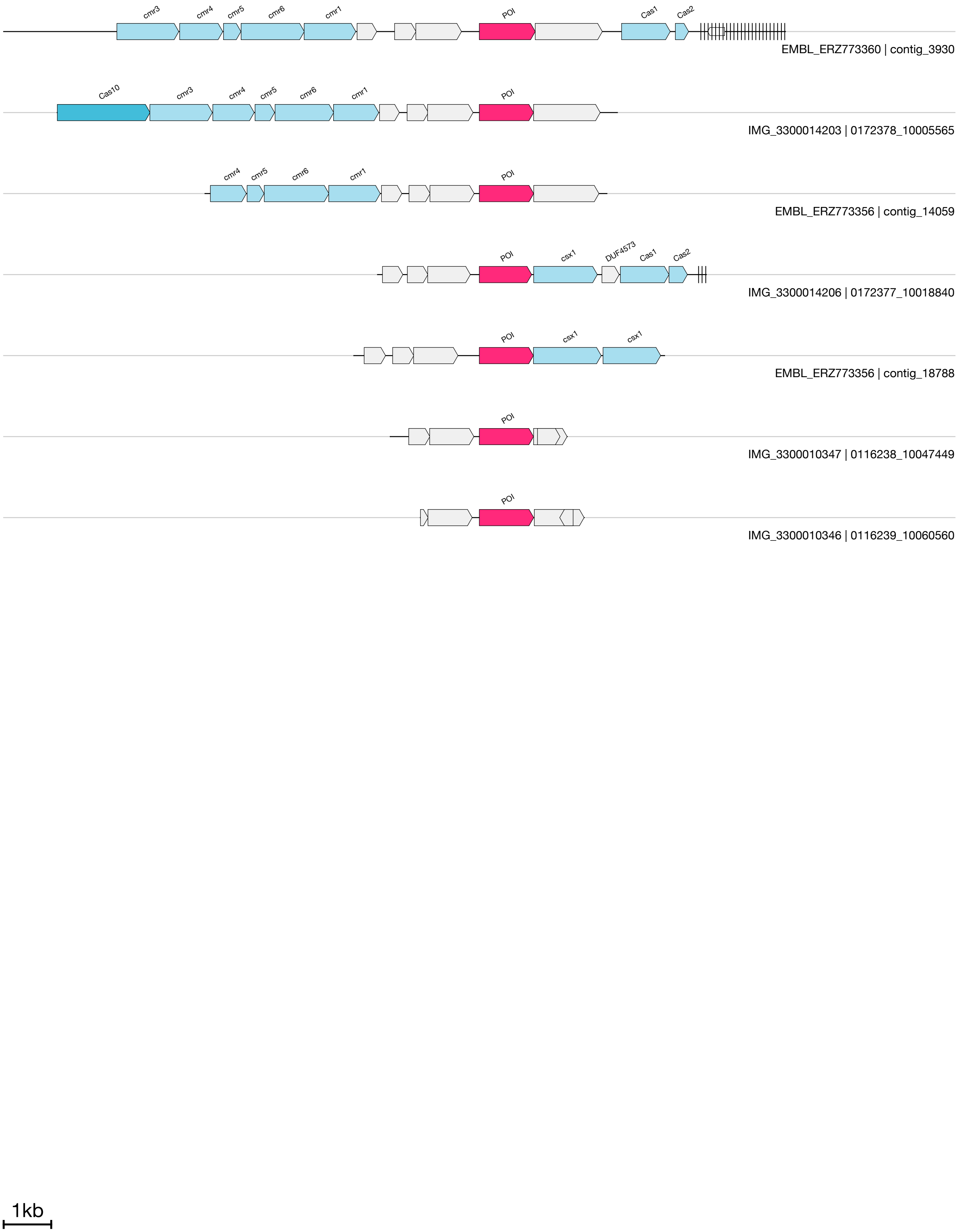

CC

UAS-31 **(Kinase)** 10 / 16.4

Auxiliary EMBL_ERZ778503&&contig_16504&&109_2353_-1

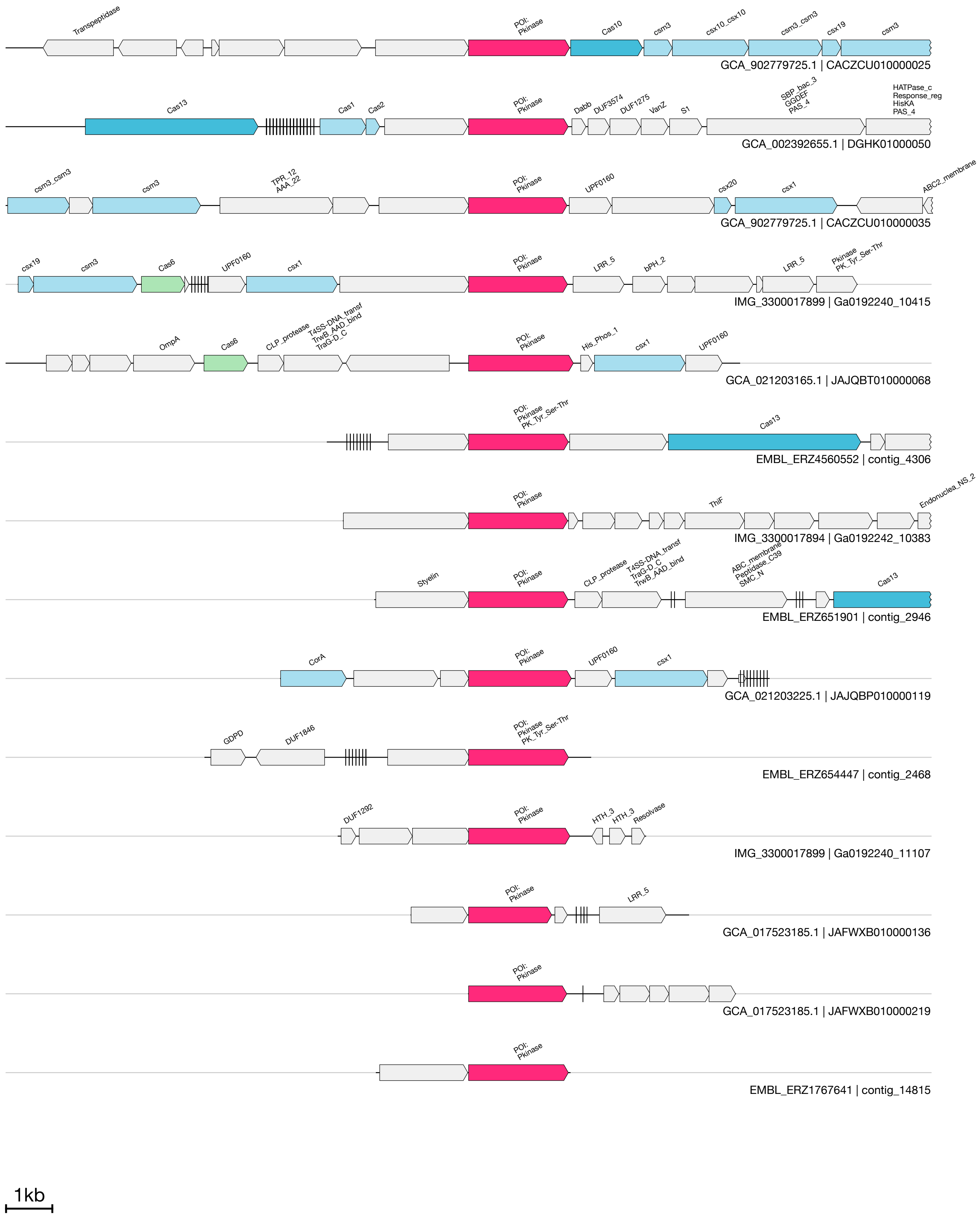

CD

UAS-85 **(betalactamase)** 2 / 5.5

Auxiliary EMBL_ERZ778607&&contig_14514&&540_2586_-1

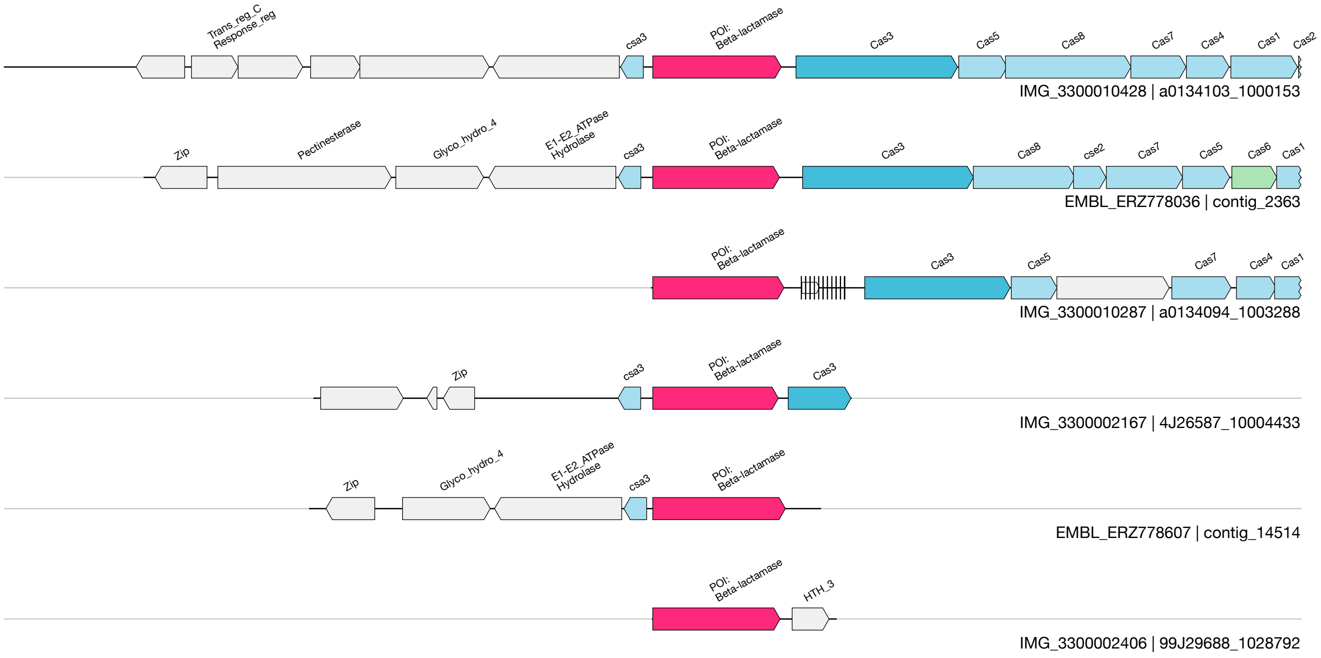

1kb

CE

UAS-86 **(large Sigma_factor)** 3 / 4.0

Auxiliary EMBL_ERZ779846&&contig_3976&&4008_5559_-1

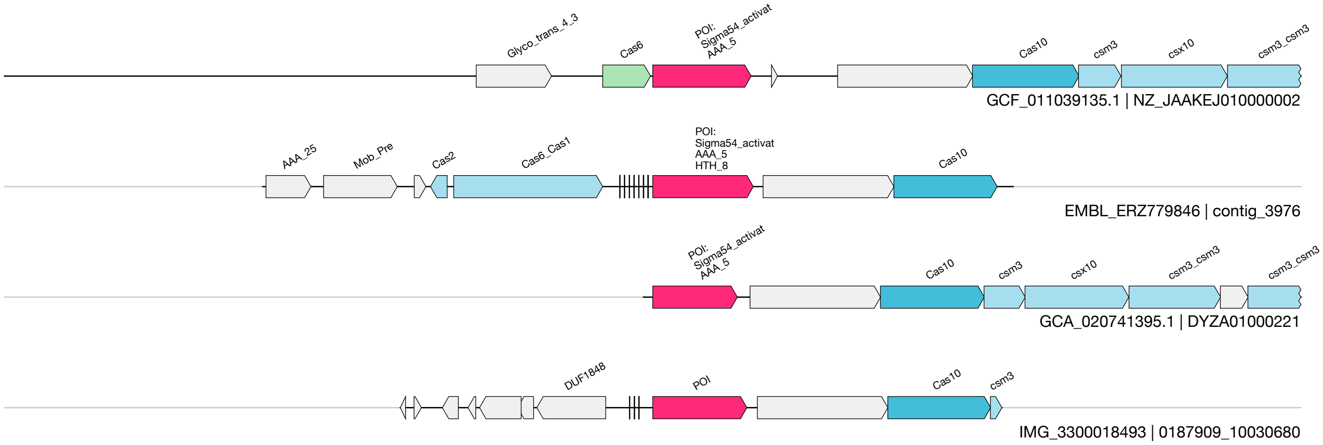

1kb

CF

UAS-87 **(vwa + PPC + kinase system)** 14 / 25.6
Auxiliary EMBL_ERZ795109&&contig_975&&4795_5770_1

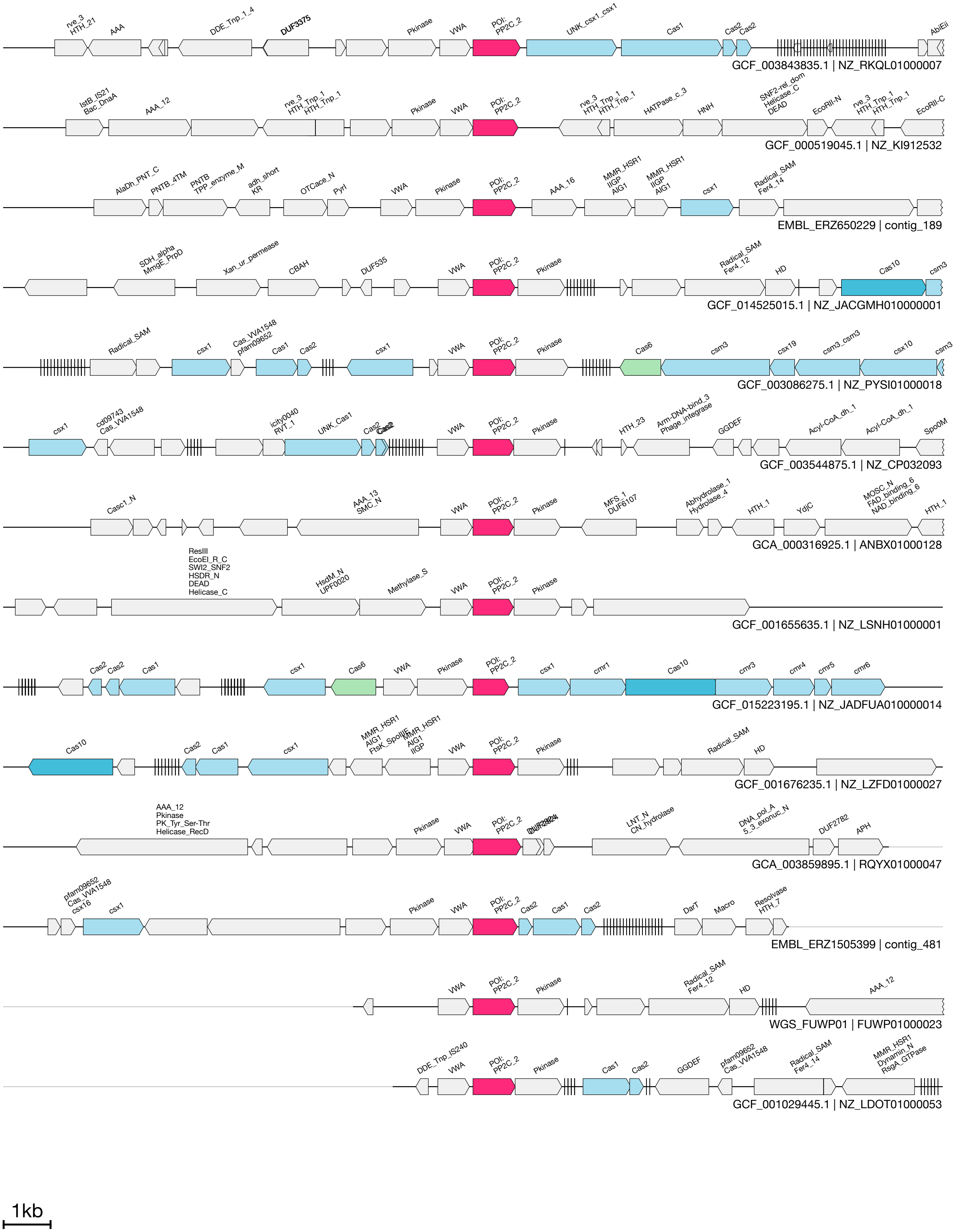

CG

UAS-24 **(RNaseT)** 3 / 3.0

Auxiliary EMBL_ERZ827088&&contig_3603&&12691_12832_1

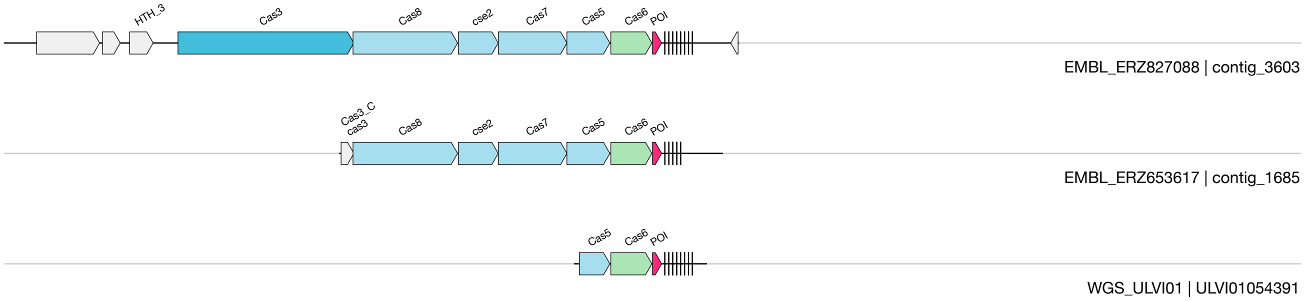

1kb

CH

UAS-88 **(Sigma_factor + Csx1)** 6 / 7.2

Auxiliary EMBL_ERZ842392&&contig_23636&&3690_4530_1

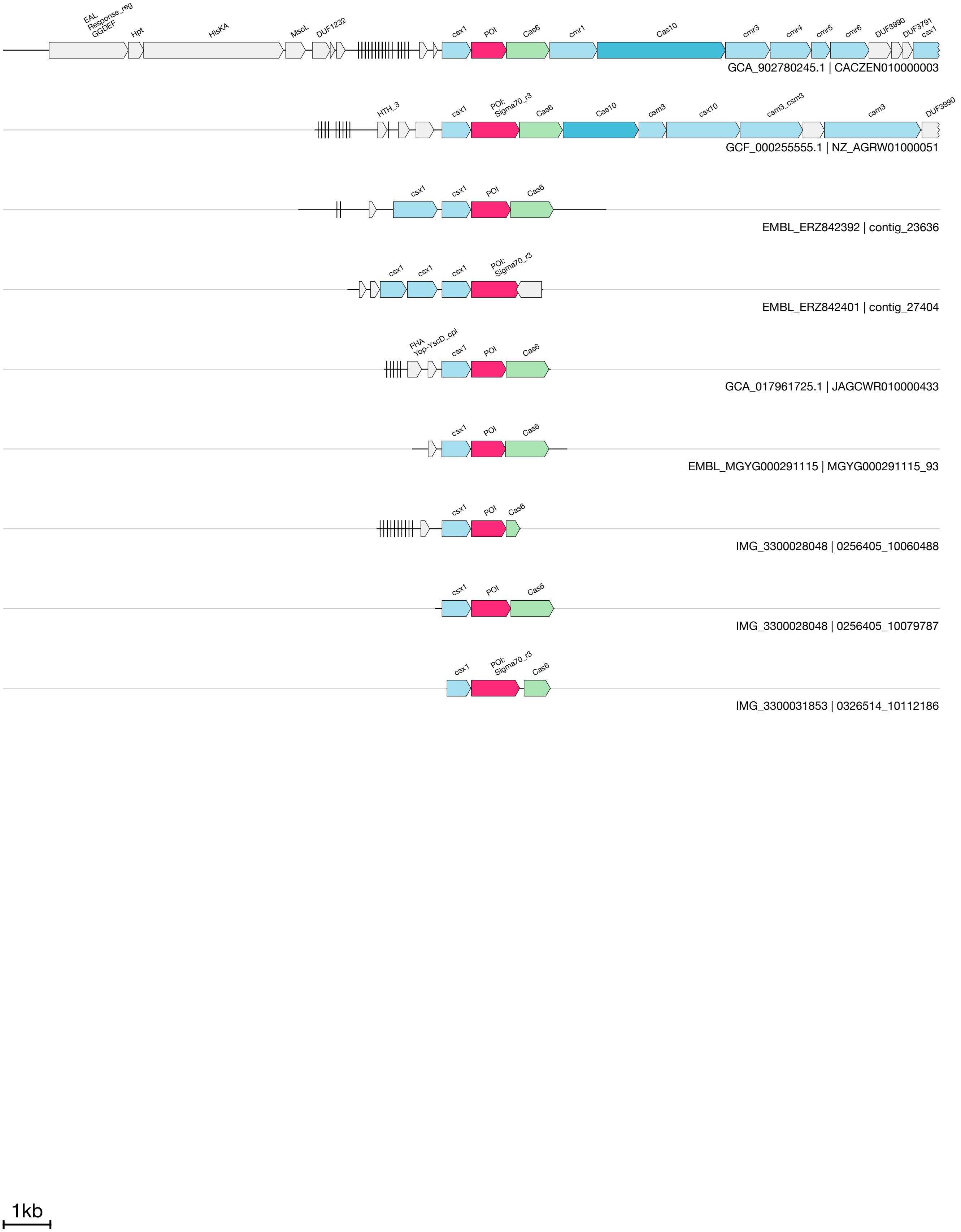

CI

UAS-19 **(Cas9 or Cas12 associated WYL)** 19 / 35.1

Auxiliary EMBL_MGYG000290606&&MGYG000290606_2&&40_988_-1

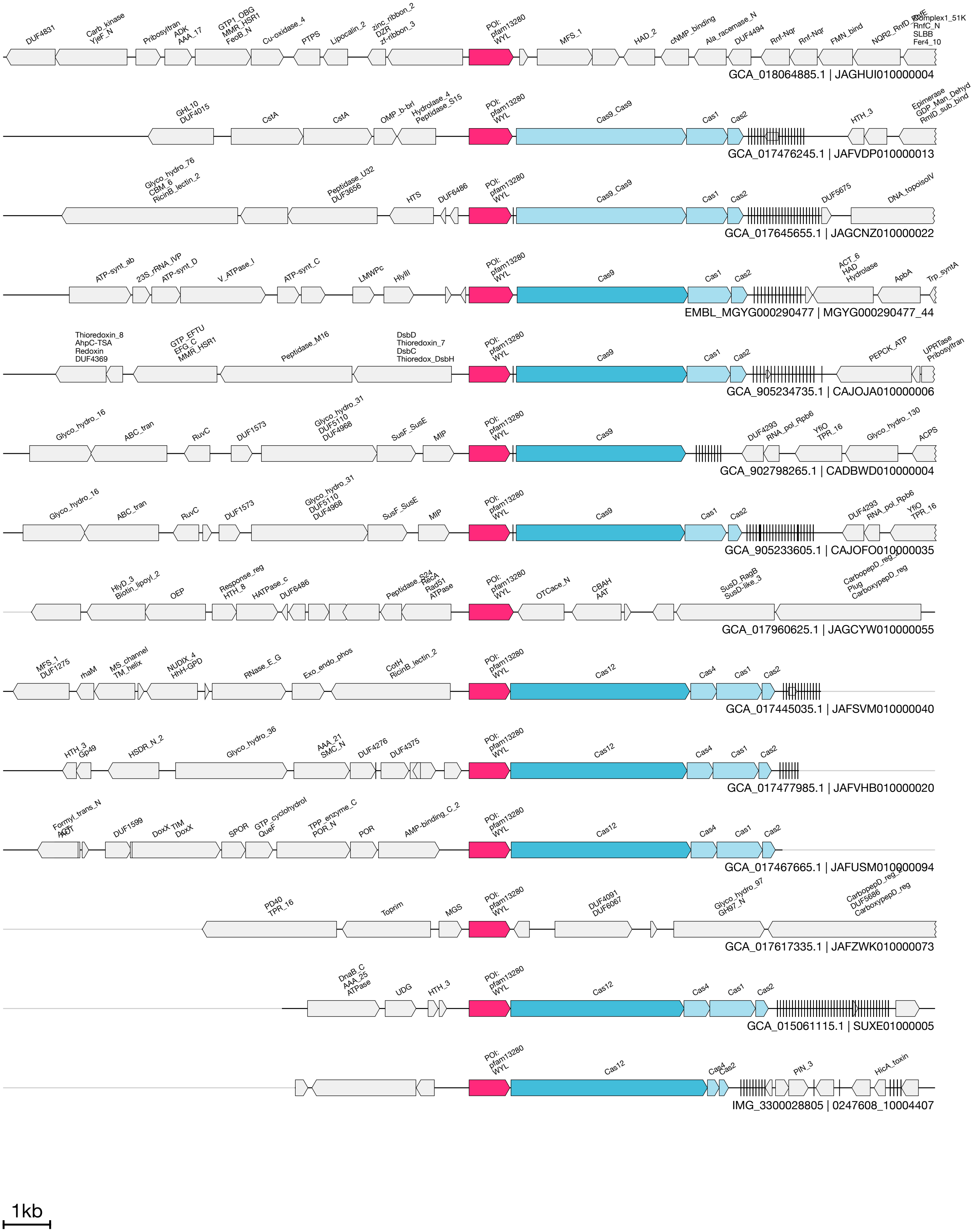

CJ

UAS-23
Auxiliary

(unknown + DUF2791 + DUF2791_ATPase)
GCA_001508235.1&&LGFR01000093&&2560_3565_-1

3 / 6.5

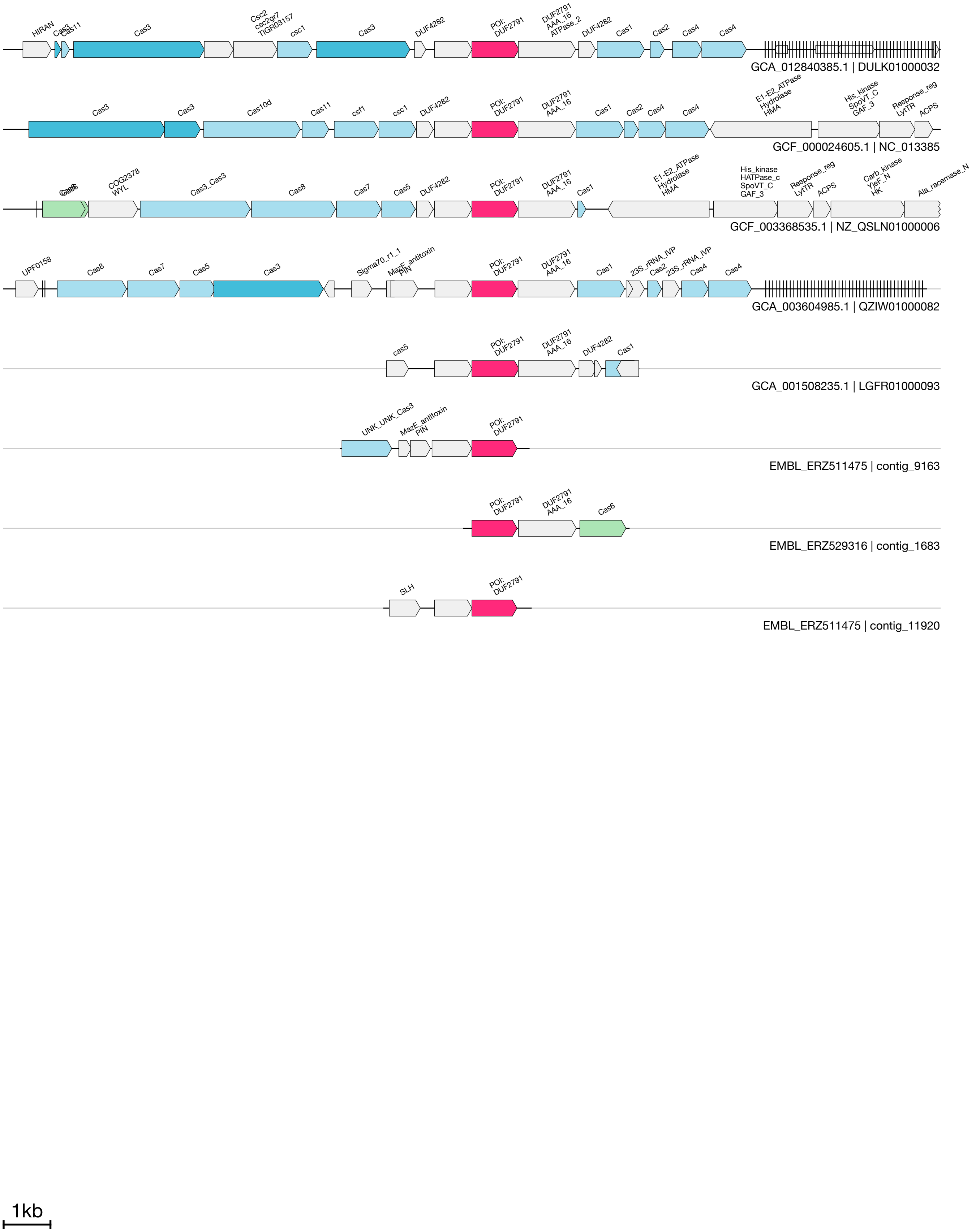

CK

UAS-89 **(unknown accessory)** 8 / 13.6

Auxiliary GCA_001570385.1&&BBYL01000030&&535_1639_-1

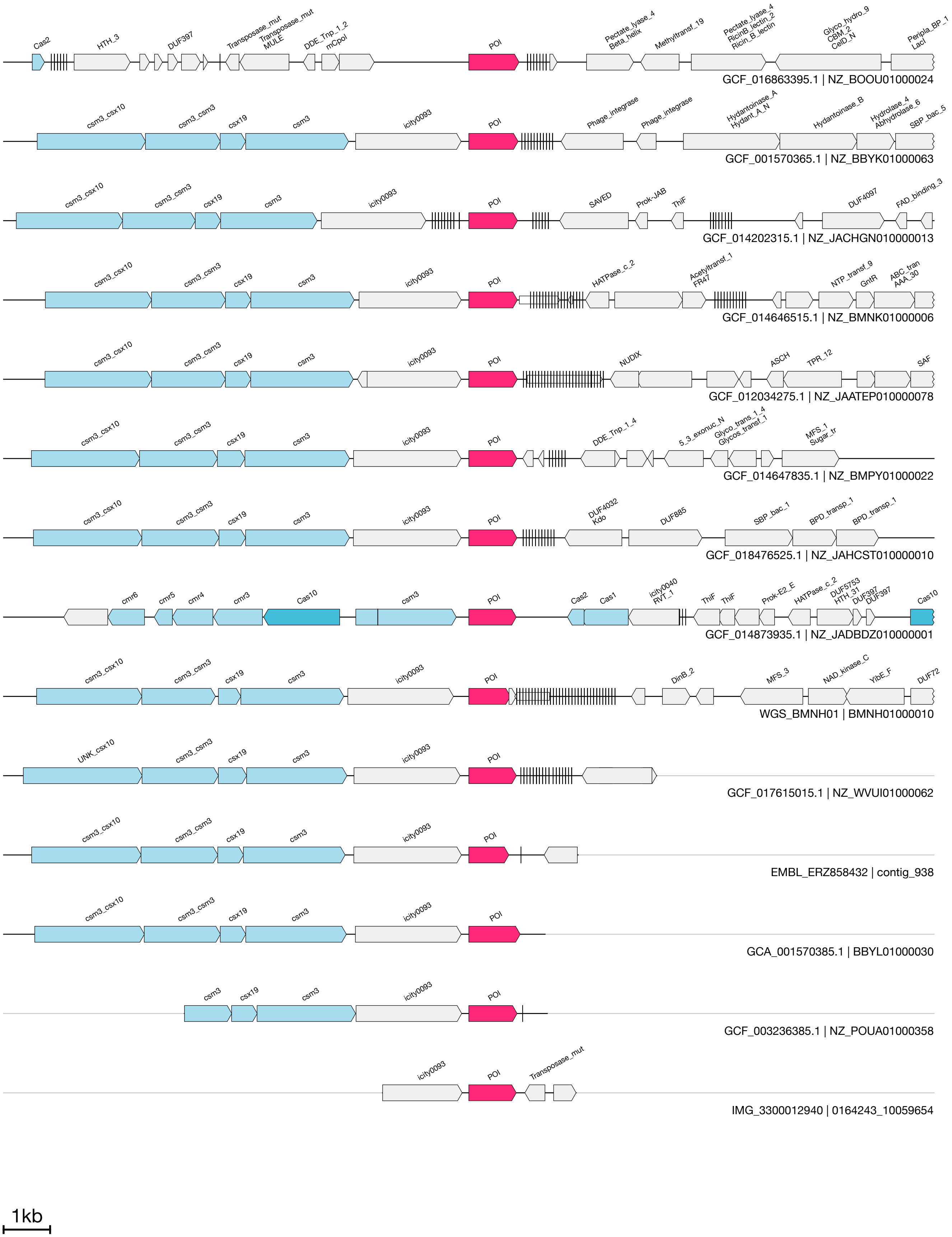

CL

UAS-90
Auxiliary

(nurA dsDNA break repair + DUF87)
GCA_003086515.1&&QEFL01000015&&20967_22101_-1

9 / 19.4

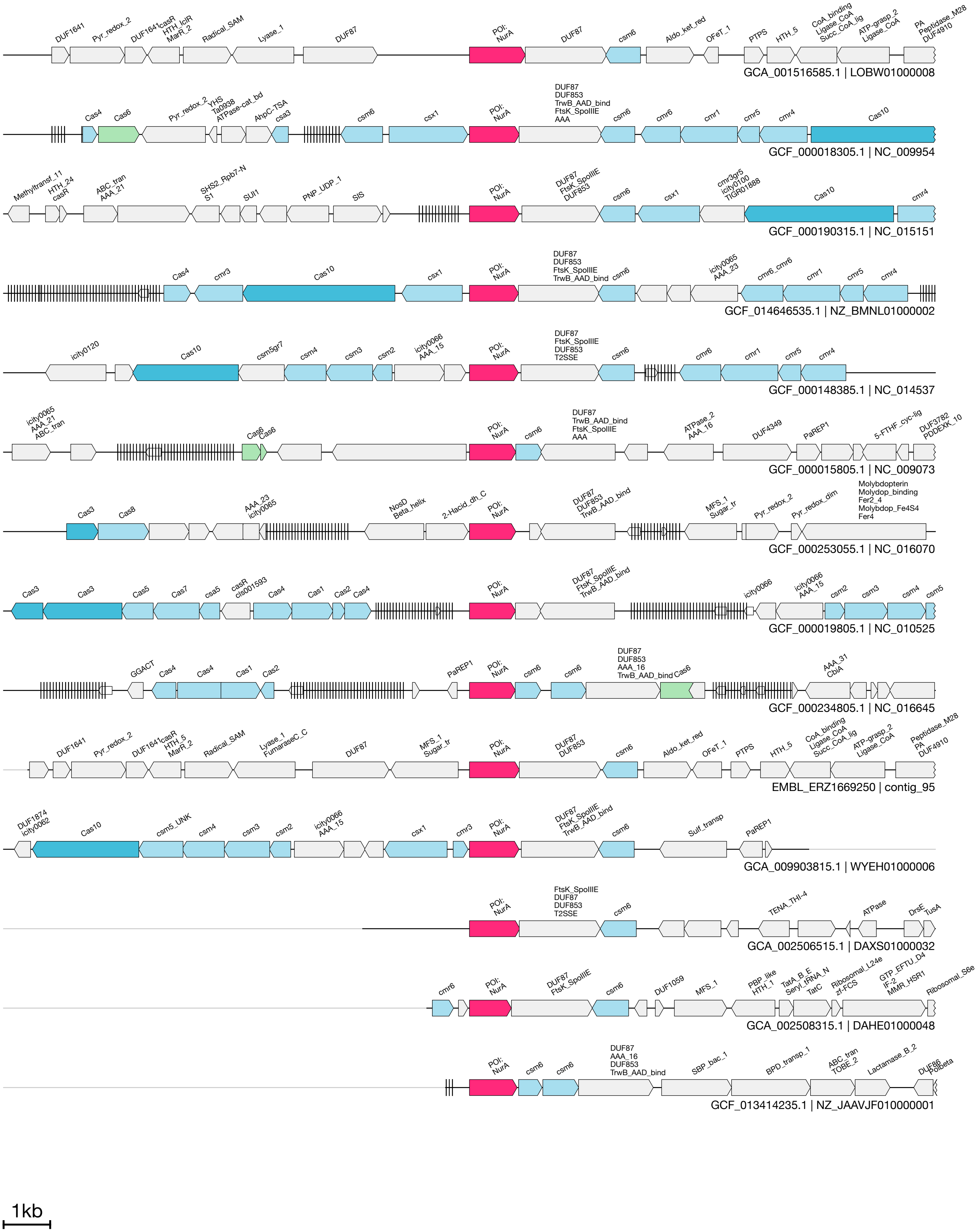

CM

UAS-91
Auxiliary

(vWA + FHA + PP2C)
GCA_011362195.1&&DTDN01000009&&39236_40742_-1

3 / 6.8

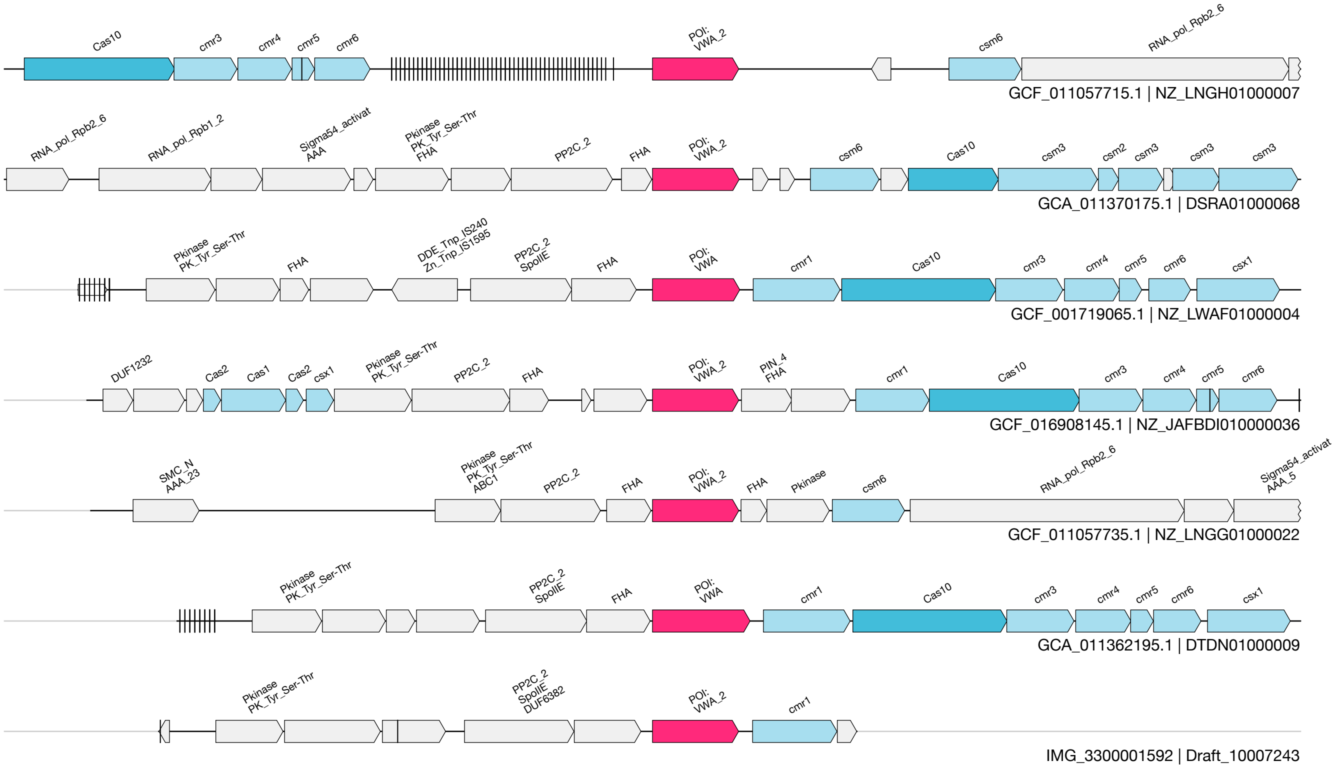

1kb

CN

UAS-92
Auxiliary

(DUF2281)
GCA_013140405.1&&JABFSV010000514&&13559_13769_-1

13 / 17.6

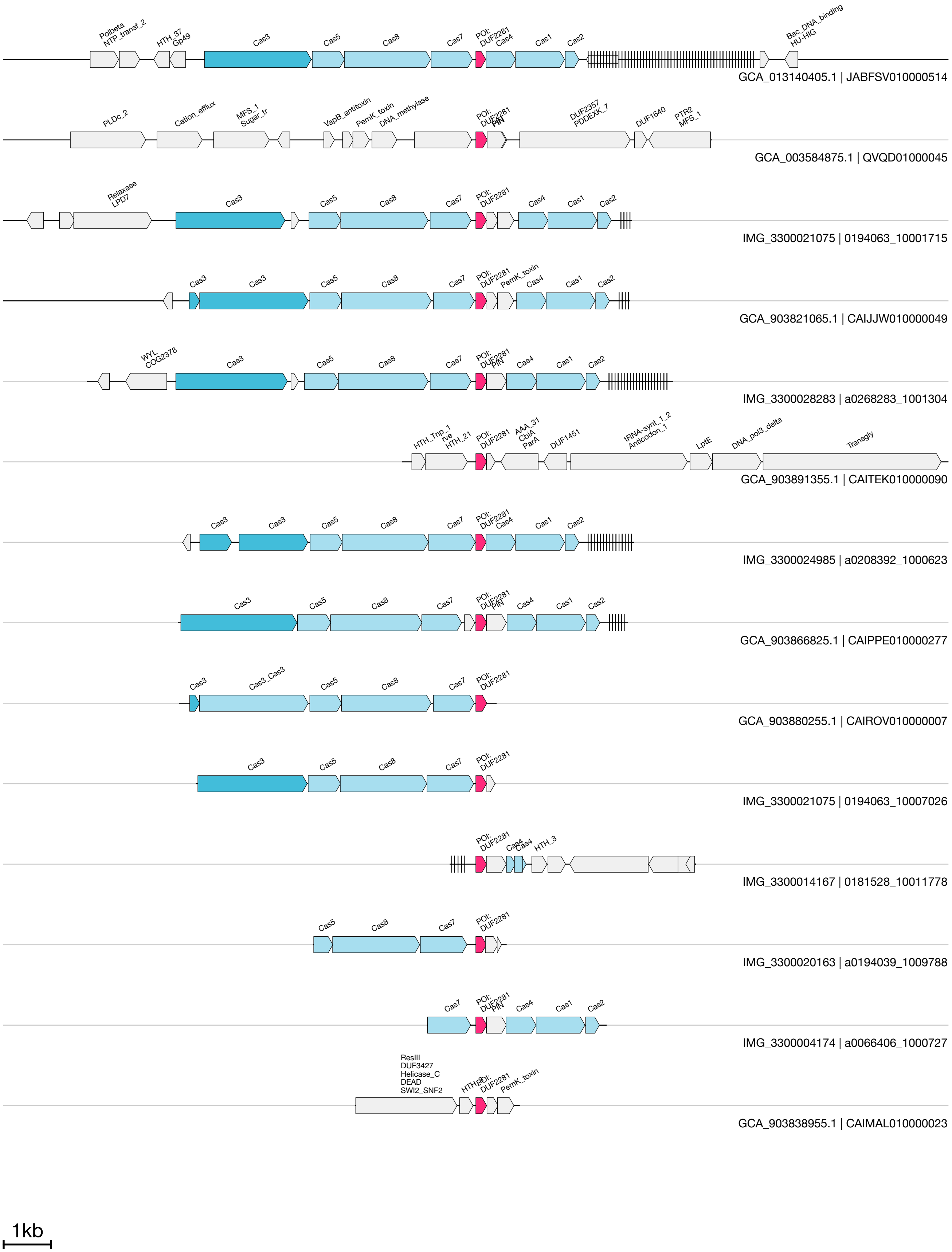

CO

UAS-93
Auxiliary

(PAP_phosphatase_CorA)
GCA_015662405.1&&DQUE01000208&&5320_7480_-1

63 / 127.1

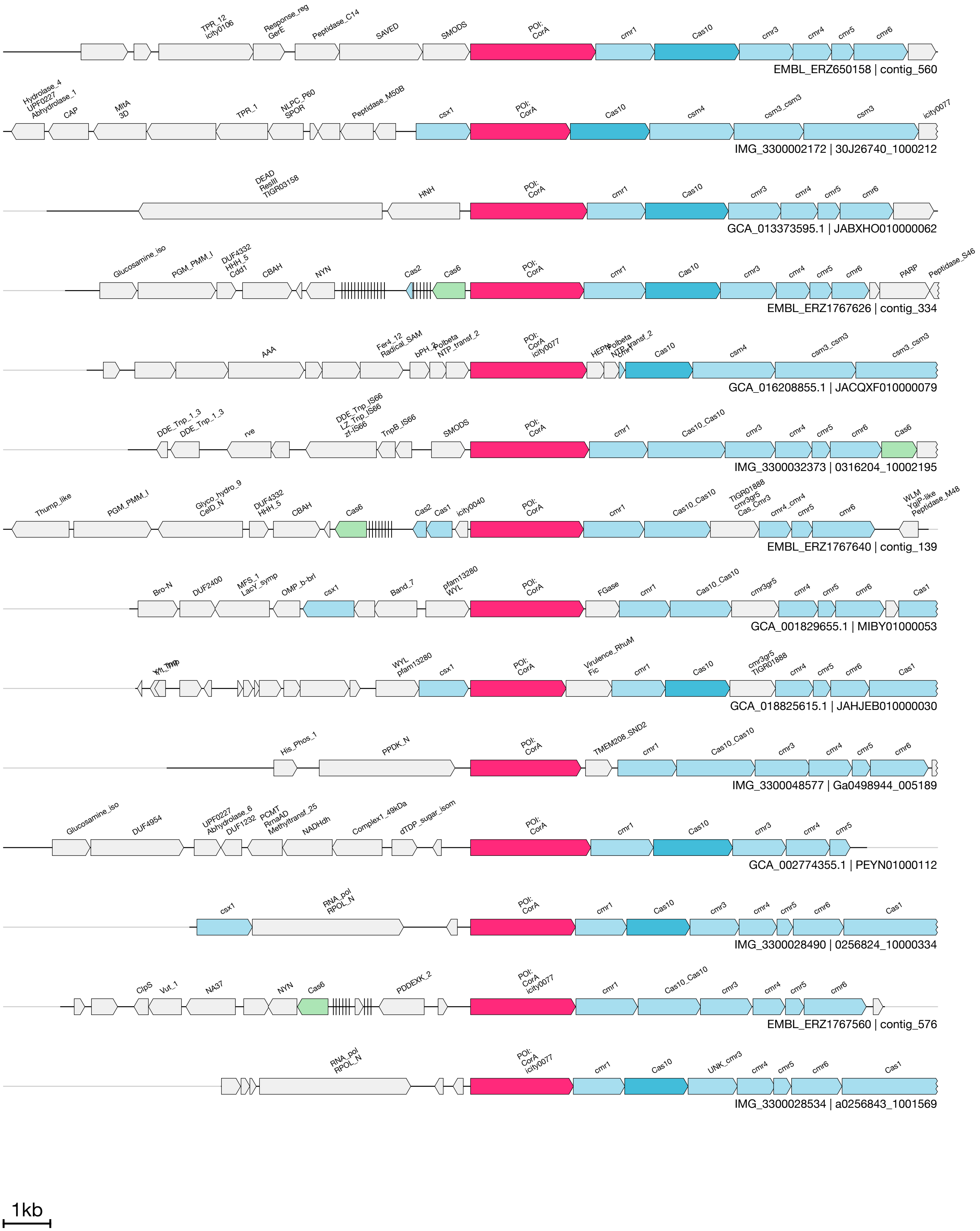

CP

UAS-28 **(Y1 TPase type III)** 6 / 7.3

Auxiliary GCA_017544725.1&&JAFYDR010000170&&27158_28184_1

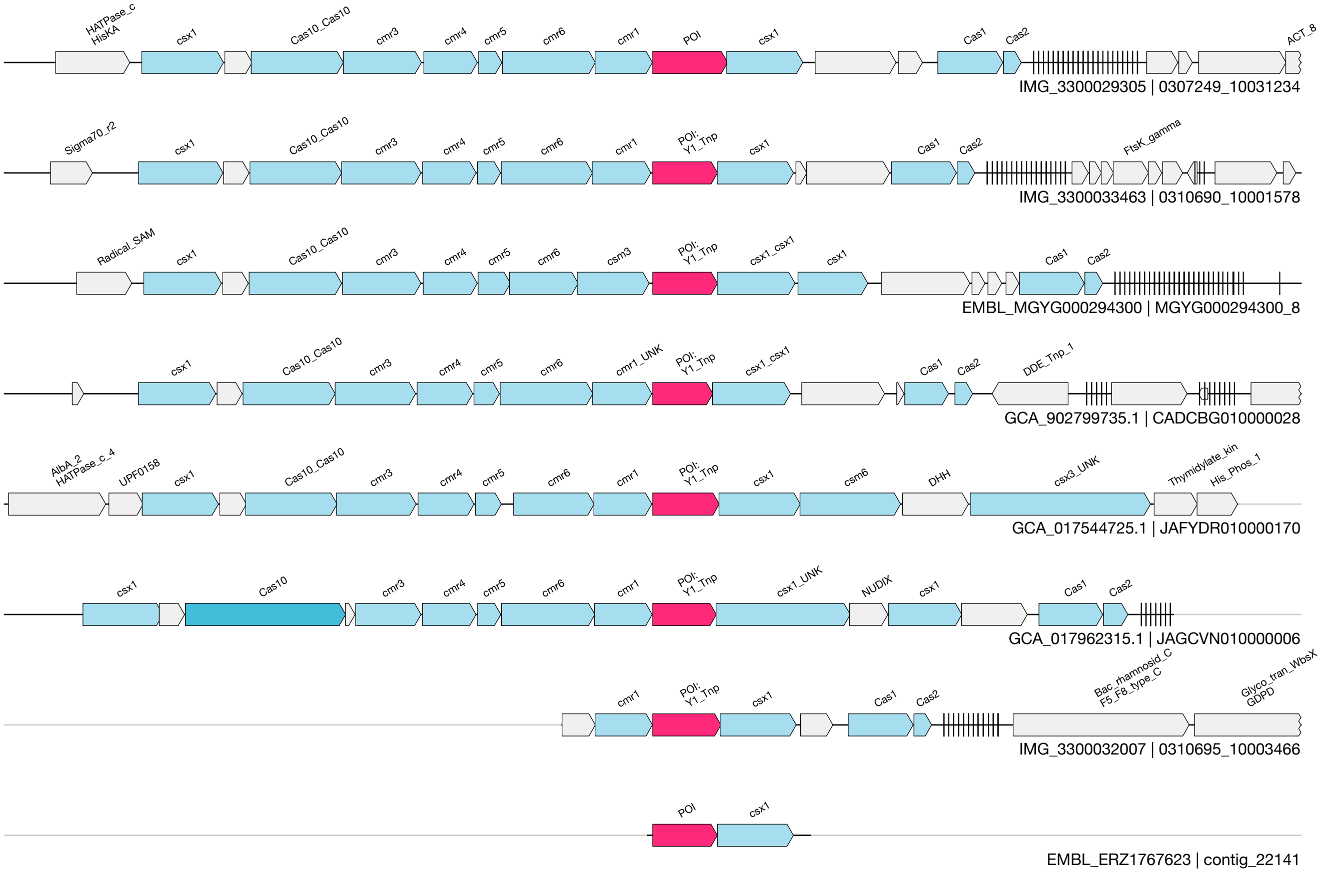

1kb

CQ

UAS-94 **(DUF3800_TPR)** 8 / 14.3

Auxiliary GCA_017934005.1&&JAGBKJ010000834&&818_3053_-1

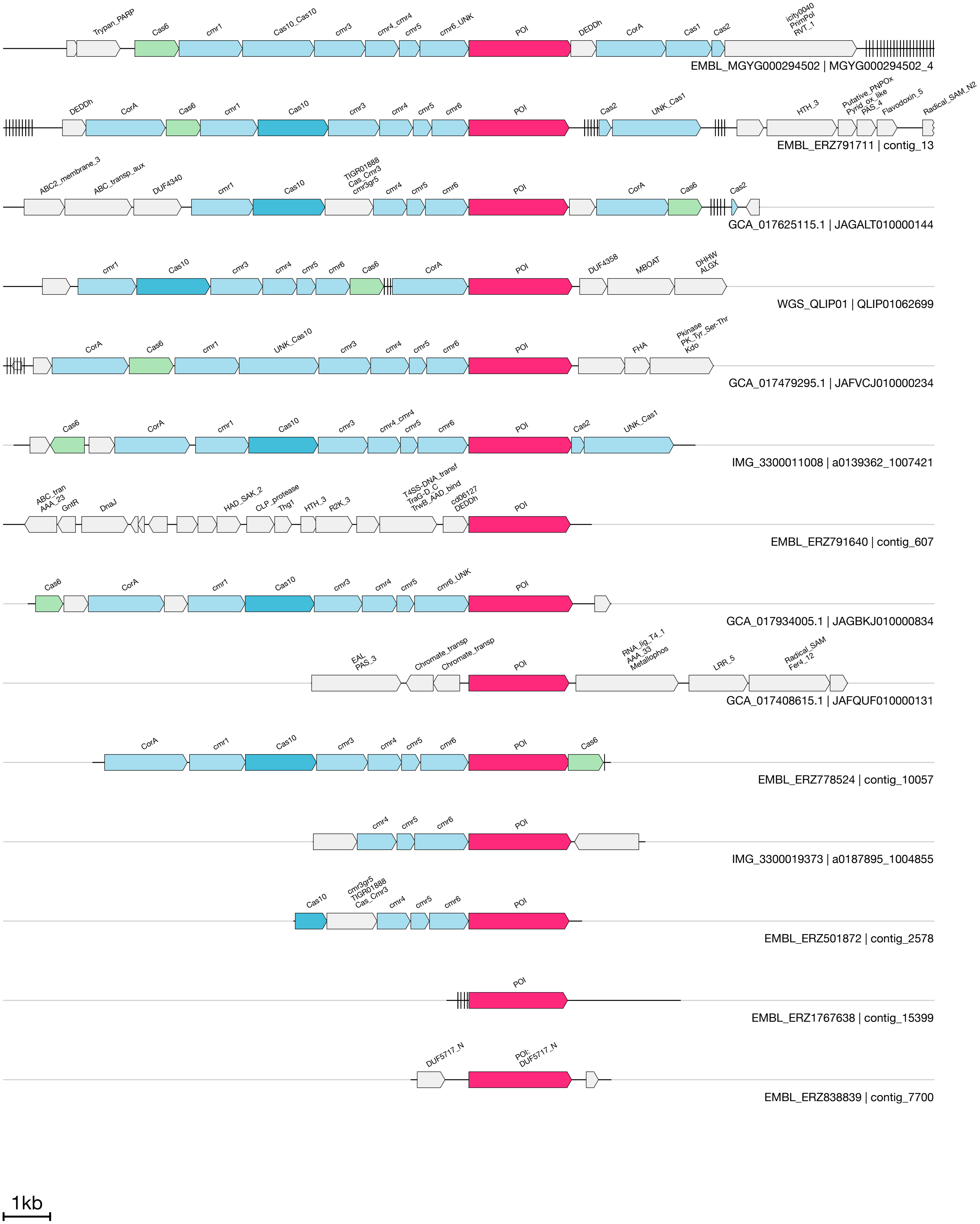

CR

UAS-95
Auxiliary

(RAMA + DUF444 + PIN)
GCA_019359445.1&&JAHHHJ010000029&&16098_16602_1

25 / 34.3

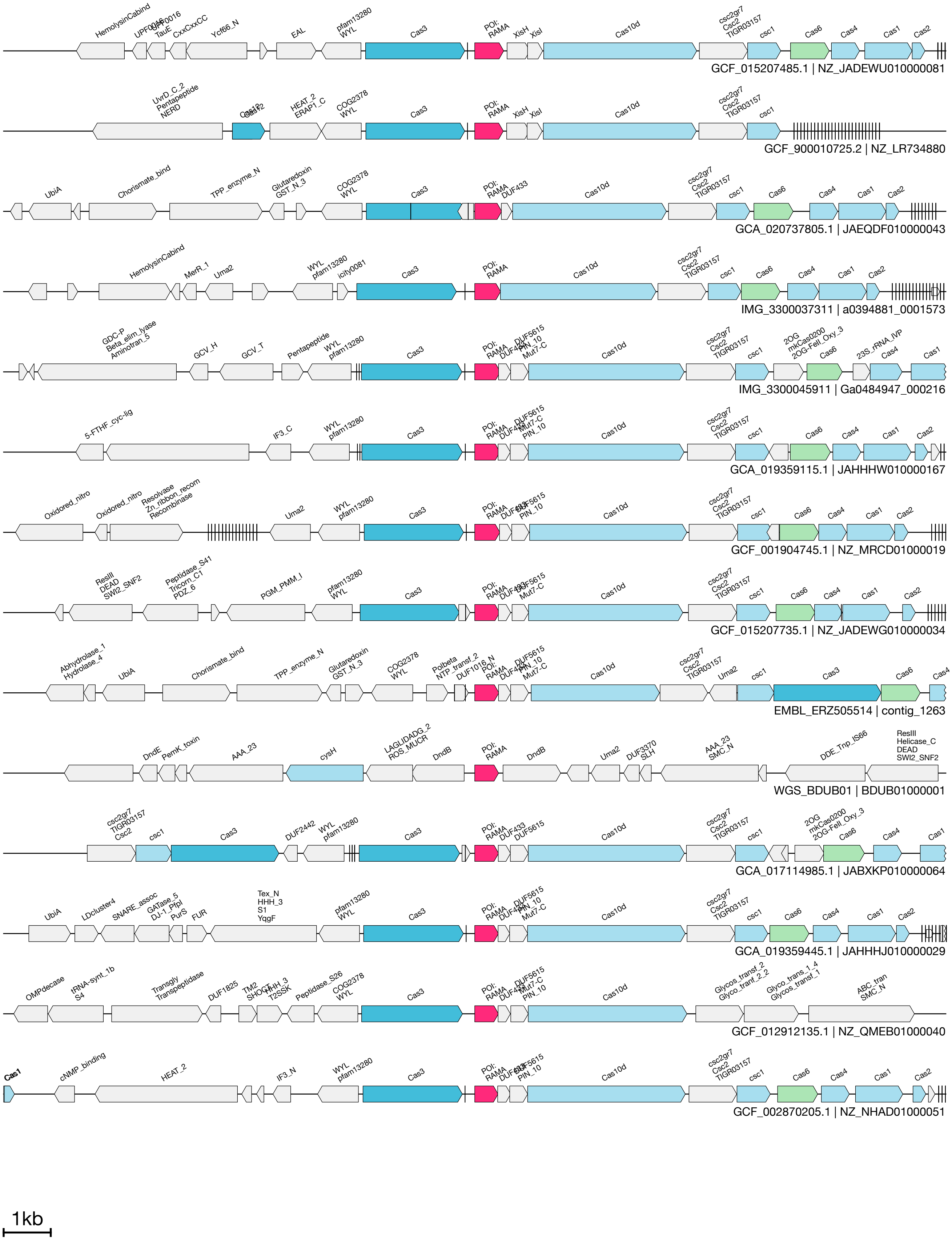

CS

UAS-96
Auxiliary

(unknown transmembrane protein)
GCA_020434175.1&&JAGRBH010000503&&340_1450_-1

N/A

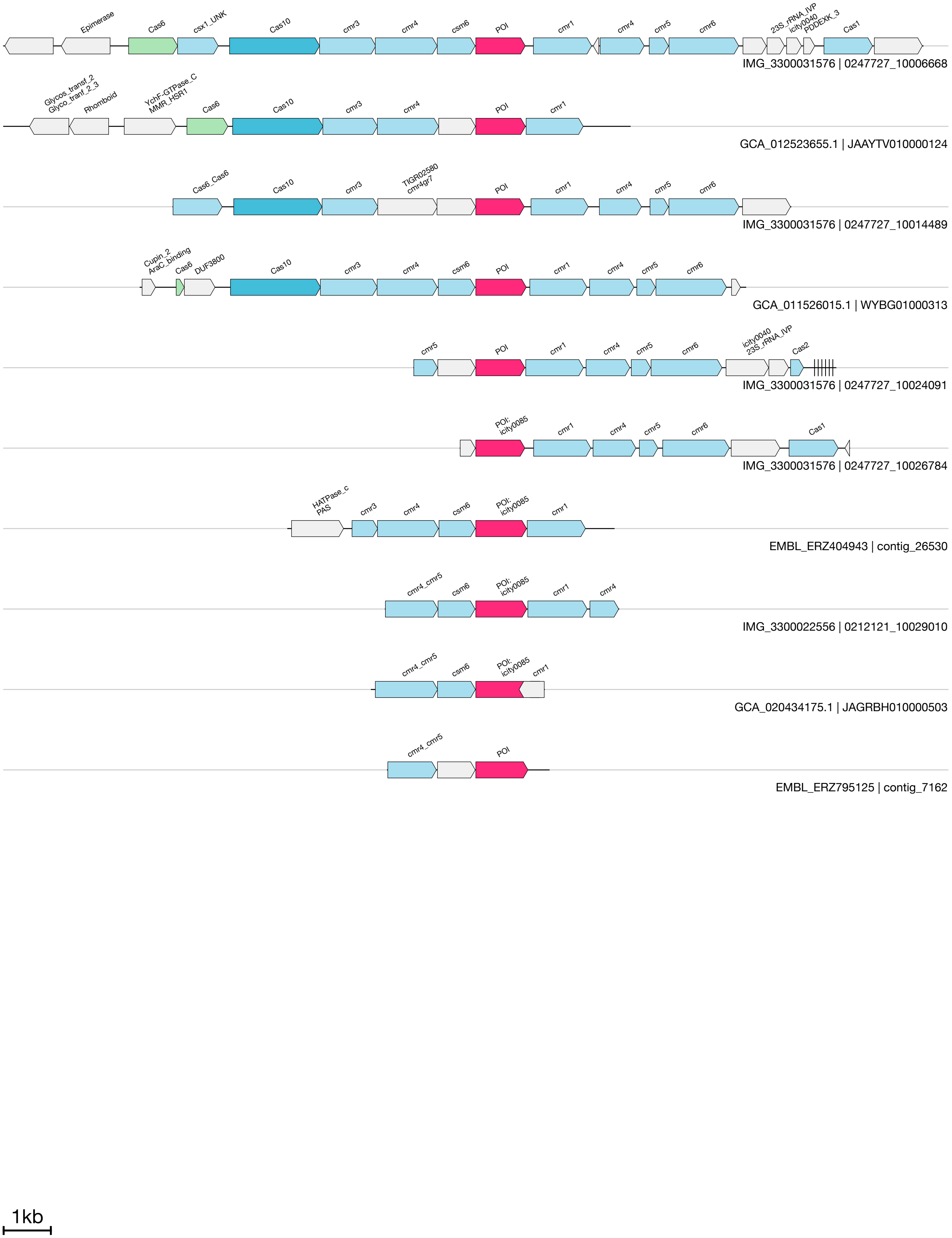

CT

UAS-97 (Fic Cascade) 2 / 4.2

Auxiliary GCA_903847485.1&&CAINDV010000696&&9392_10559_-1

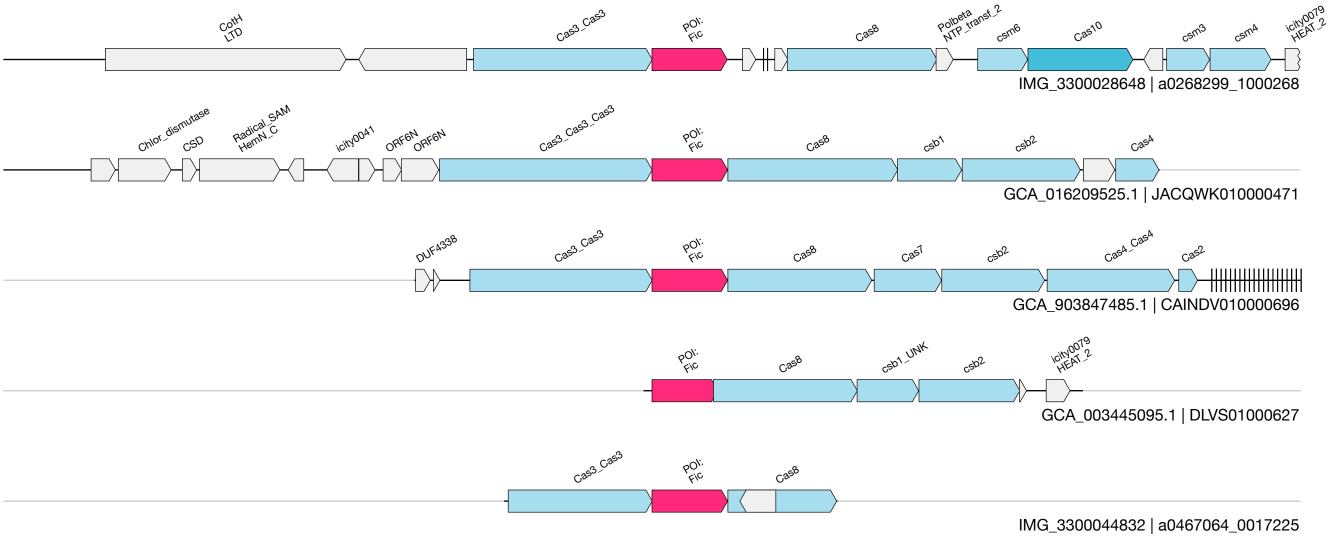

1kb

CU

UAS-98 **(TPR_MALT)** 5 / 6.3

Auxiliary GCA_910584945.1&&CAJUDY010000014&&24554_26873_1

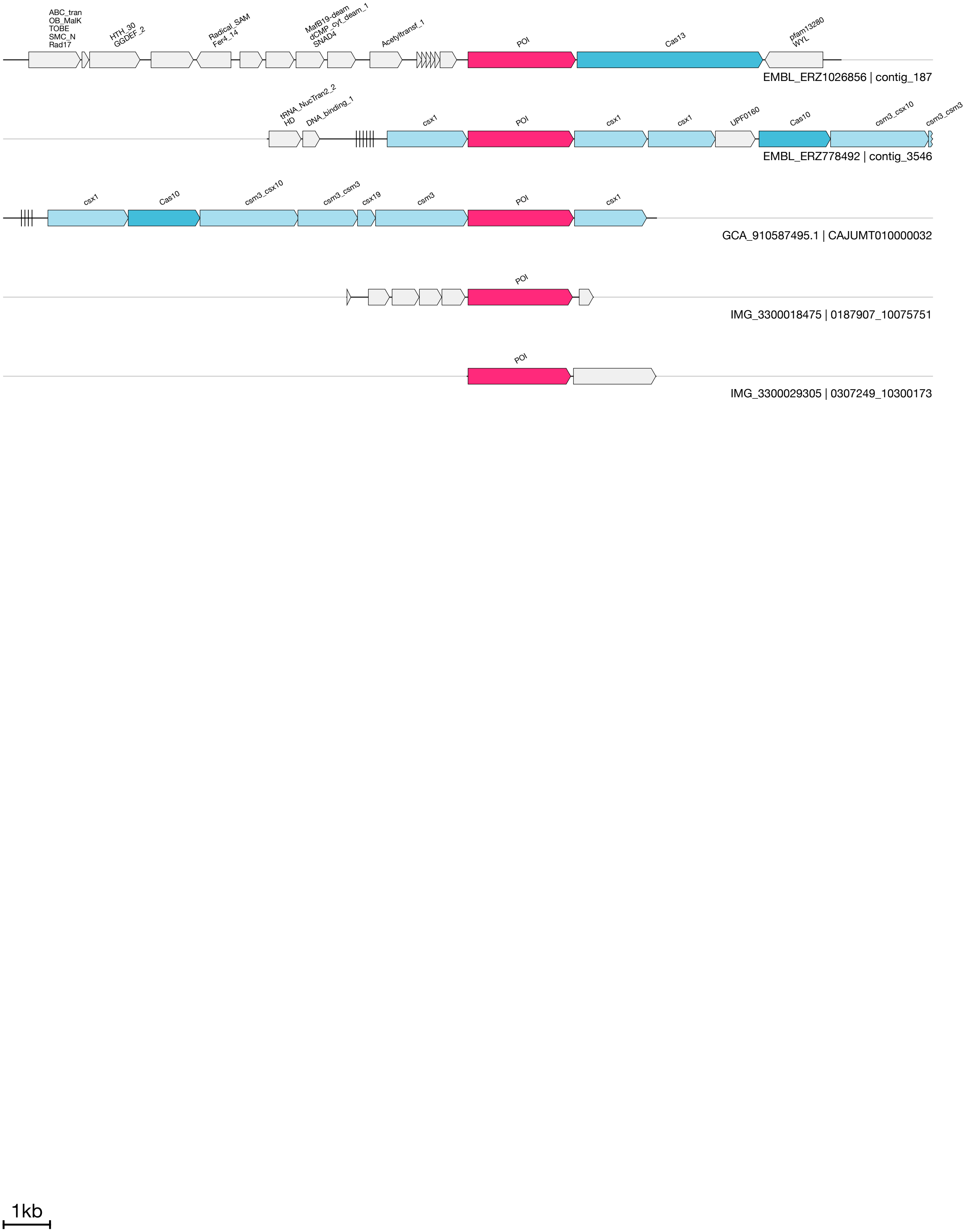

CV

UAS-30 **(UvrD_NERD nuclease)** 6 / 6.0

Auxiliary GCF_000419585.1&&NZ_ATTH01000001&&941528_943577_-1

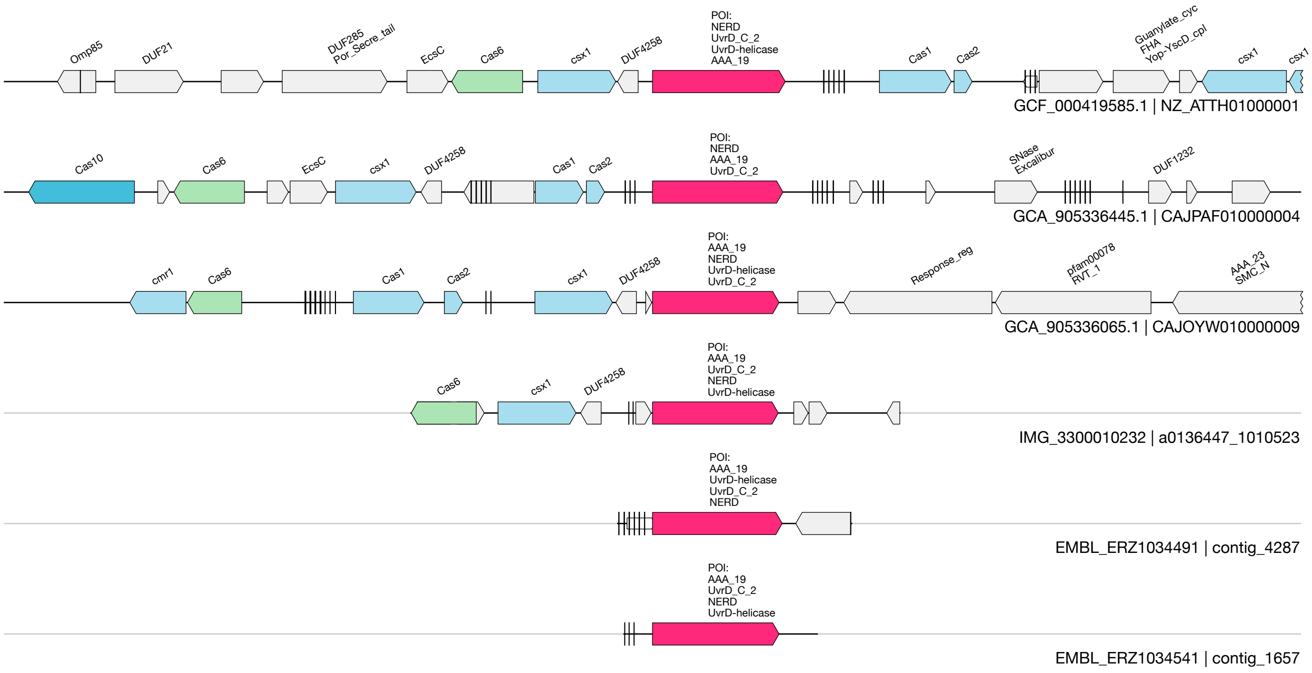

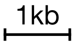

CW

UAS-99 **(Radical SAM protein)** 20 / 37.7

Auxiliary GCF_001029445.1&&NZ_LDOT01000053&&7692_9432_1

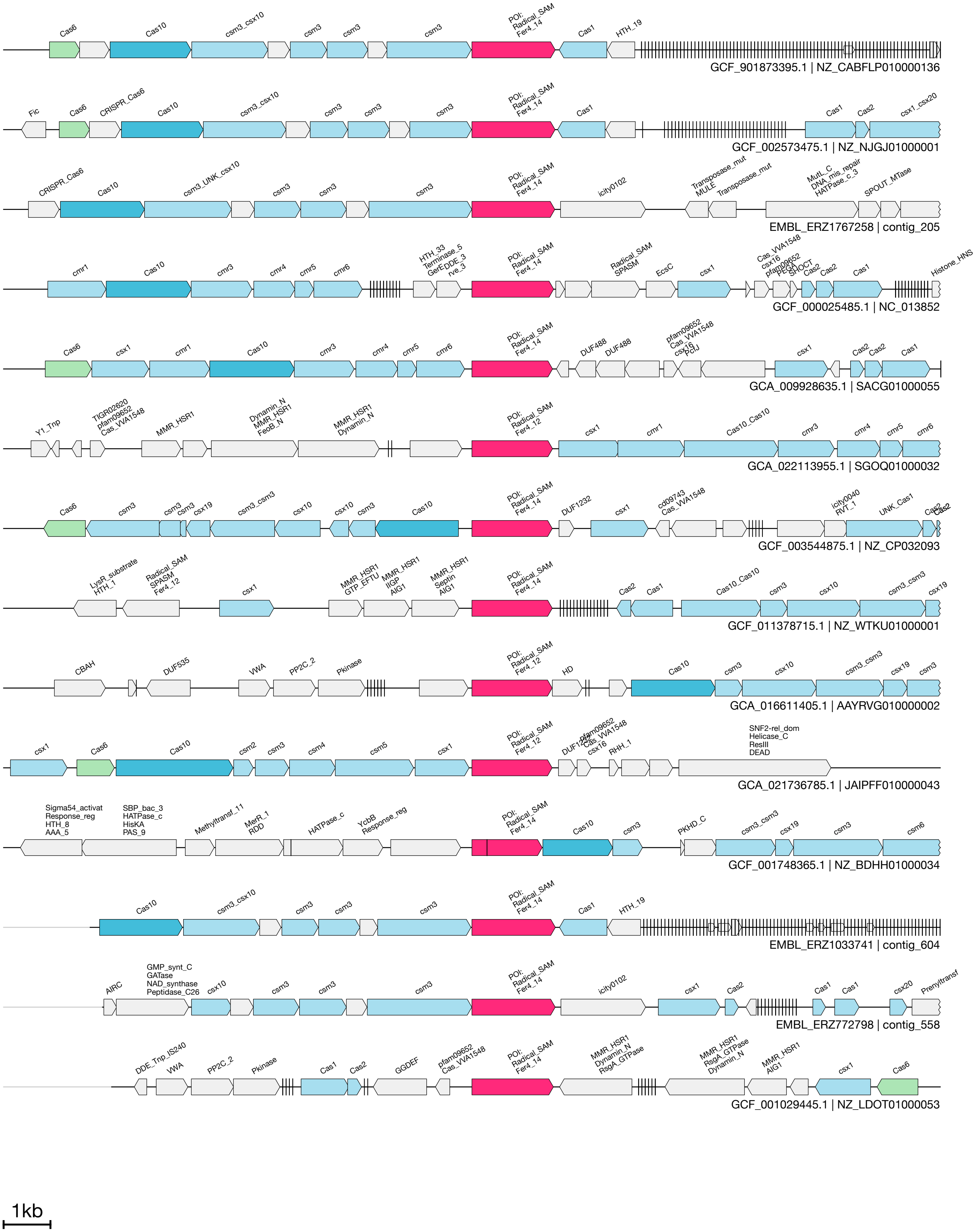

CX

UAS-100 **(wWA + kinase)** 6 / 9.9

Auxiliary GCF_001655635.1&&NZ_LSNH01000001&&2031044_2032025_-1

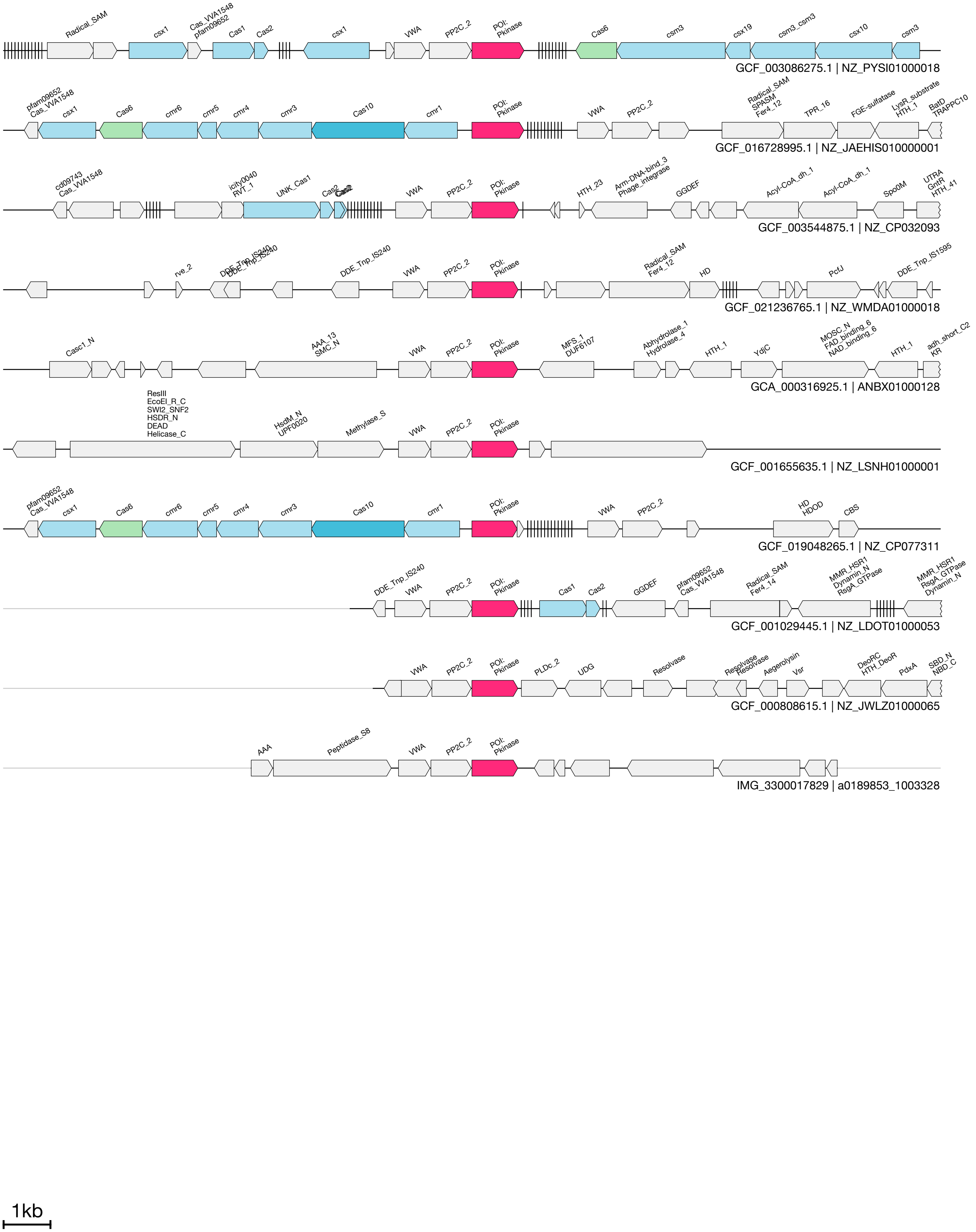

CY

UAS-101 **(DUF2204)** 19 / 47.0

Auxiliary

GCF_003668875.1&&NZ_RAWM01000178&&1907_2396_-1

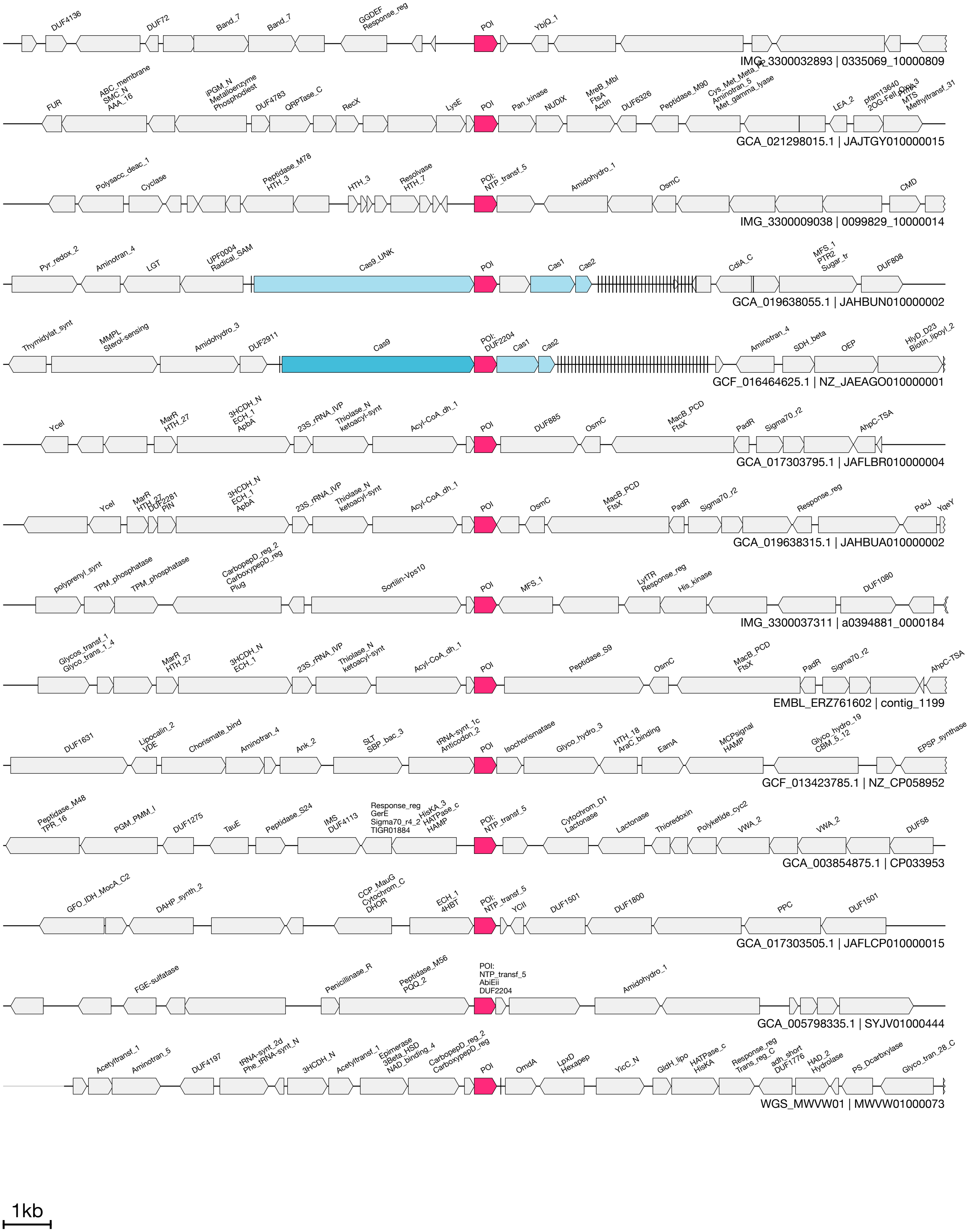

CZ

UAS-102 **(DUF5343 + Helicase_HNH_ResIII)** 3 / 3.0

Auxiliary GCF_016526145.1&&NZ_JADDUE010000010&&220137_220869_-1

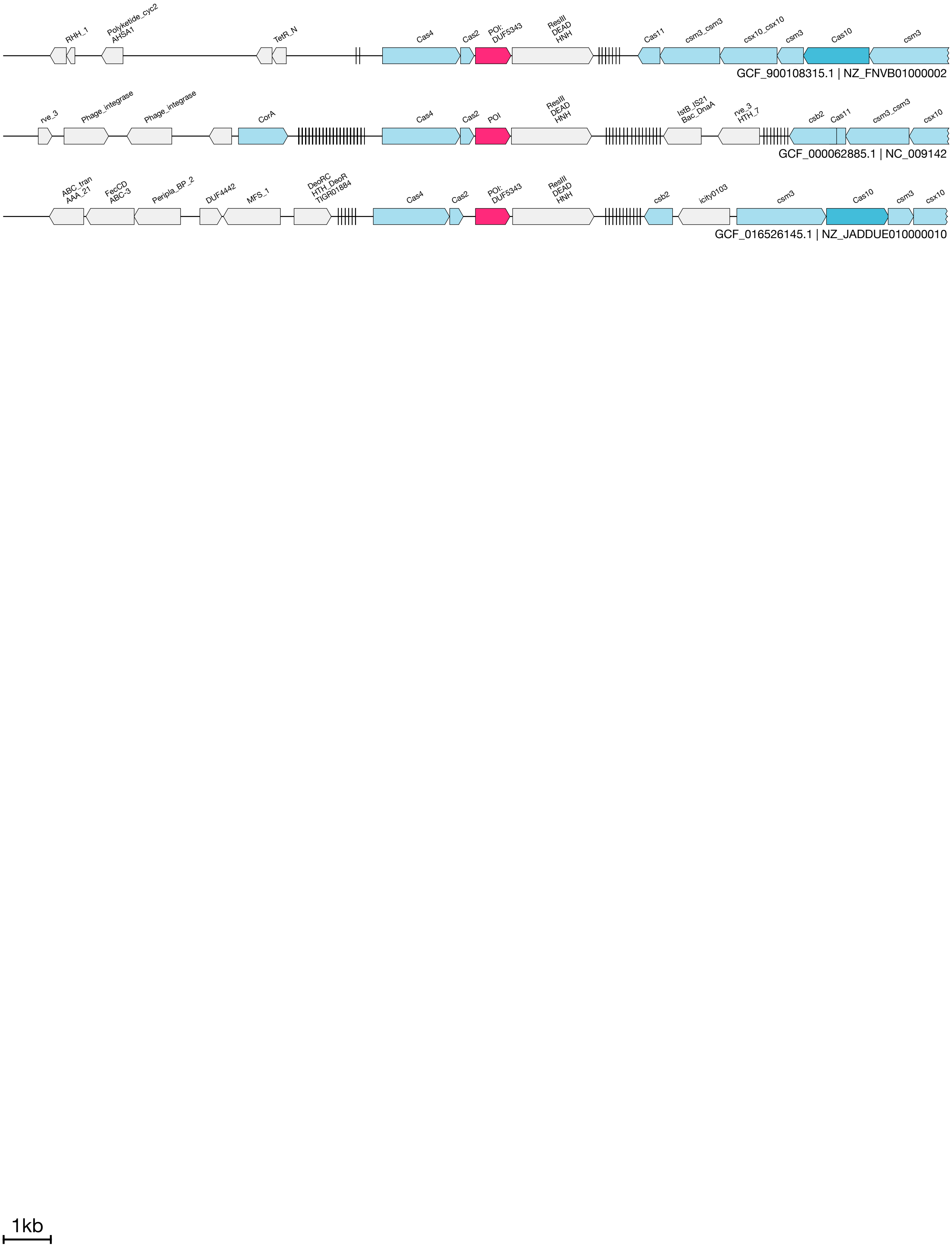

DA

UAS-103 **(TPR HATPase)** 2 / 3.0

Auxiliary GCF_016757215.1&&NZ_JAESIY010000005&&313654_315544_1

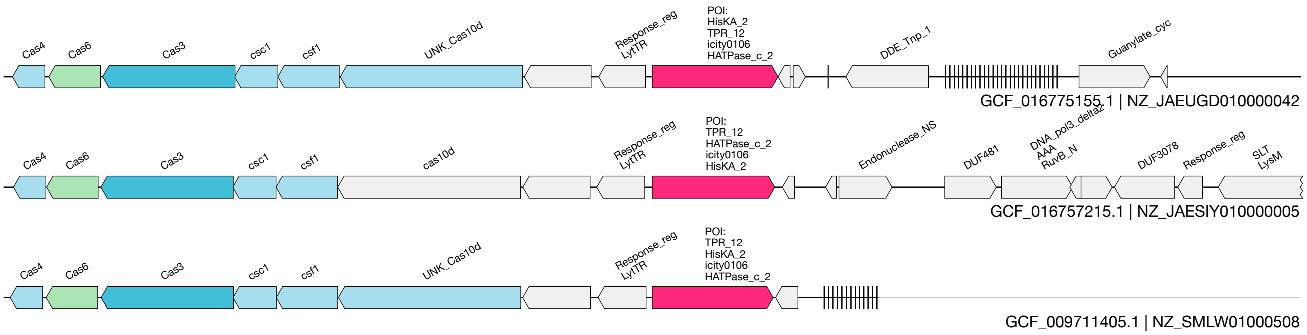

1kb

DB

UAS-104
Auxiliary

(ATPase + Metallophosphatase)
GCF_018502485.1&&NZ_CP075546&&993250_997099_1

3 / 3.0

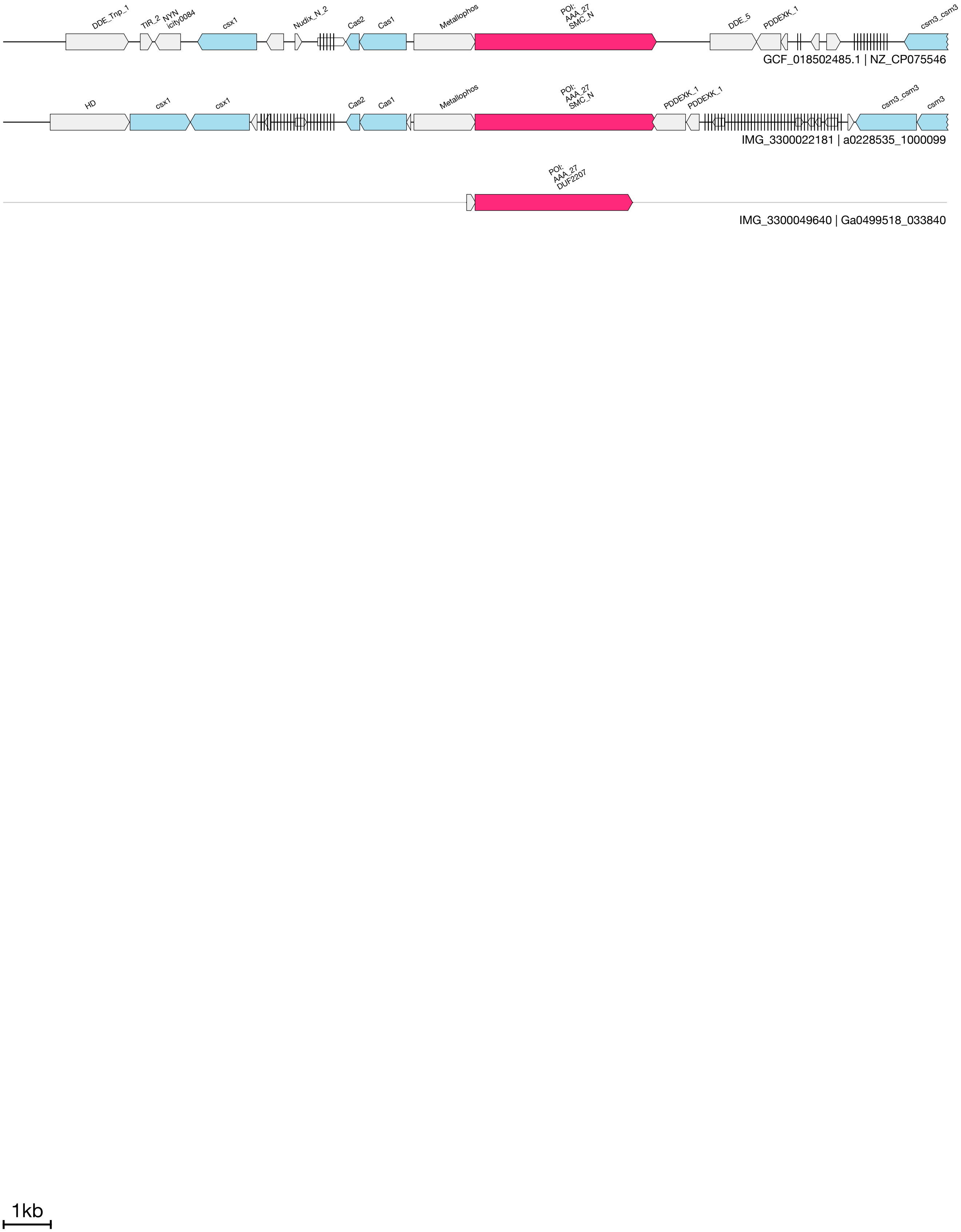

DC

UAS-105 **(M78 peptidase)** 5 / 5.6

Auxiliary GCF_021531665.1&&NZ_JADYUH010000077&&9506_10499_-1

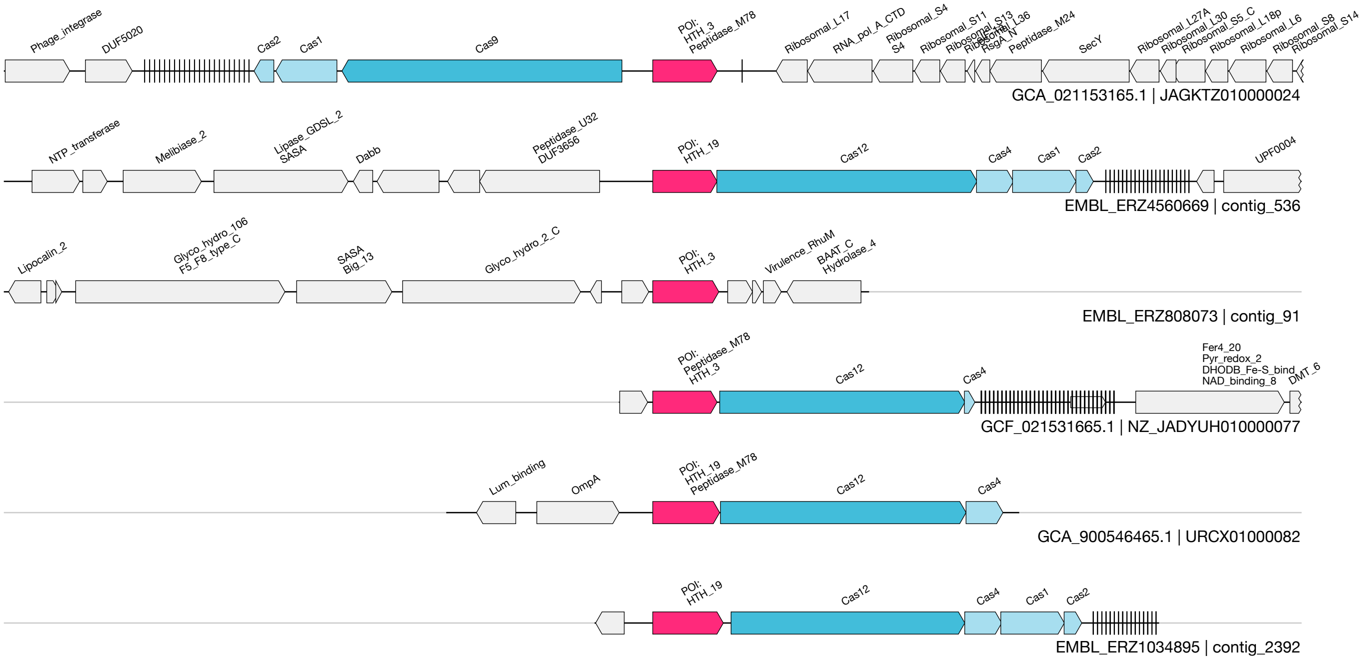

1kb

DD

UAS-106 **(DUF3800)** 3 / 3.0
Auxiliary GCF_900453405.1&&NZ_UGPM01000003&&1785257_1786514_-1

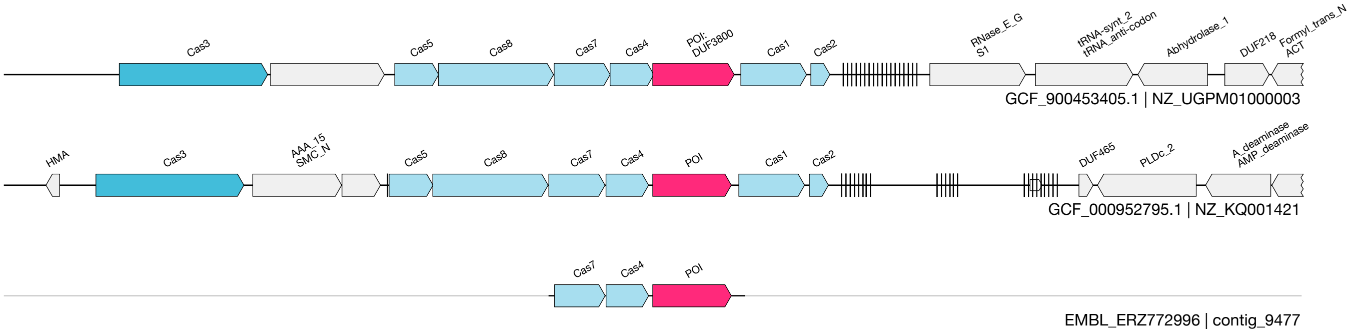

1kb

DE

UAS-107 **(Toprim)** 17 / 25.5

Auxiliary GCF_902500215.1&&NZ_CABVOU010000021&&55311_56466_-1

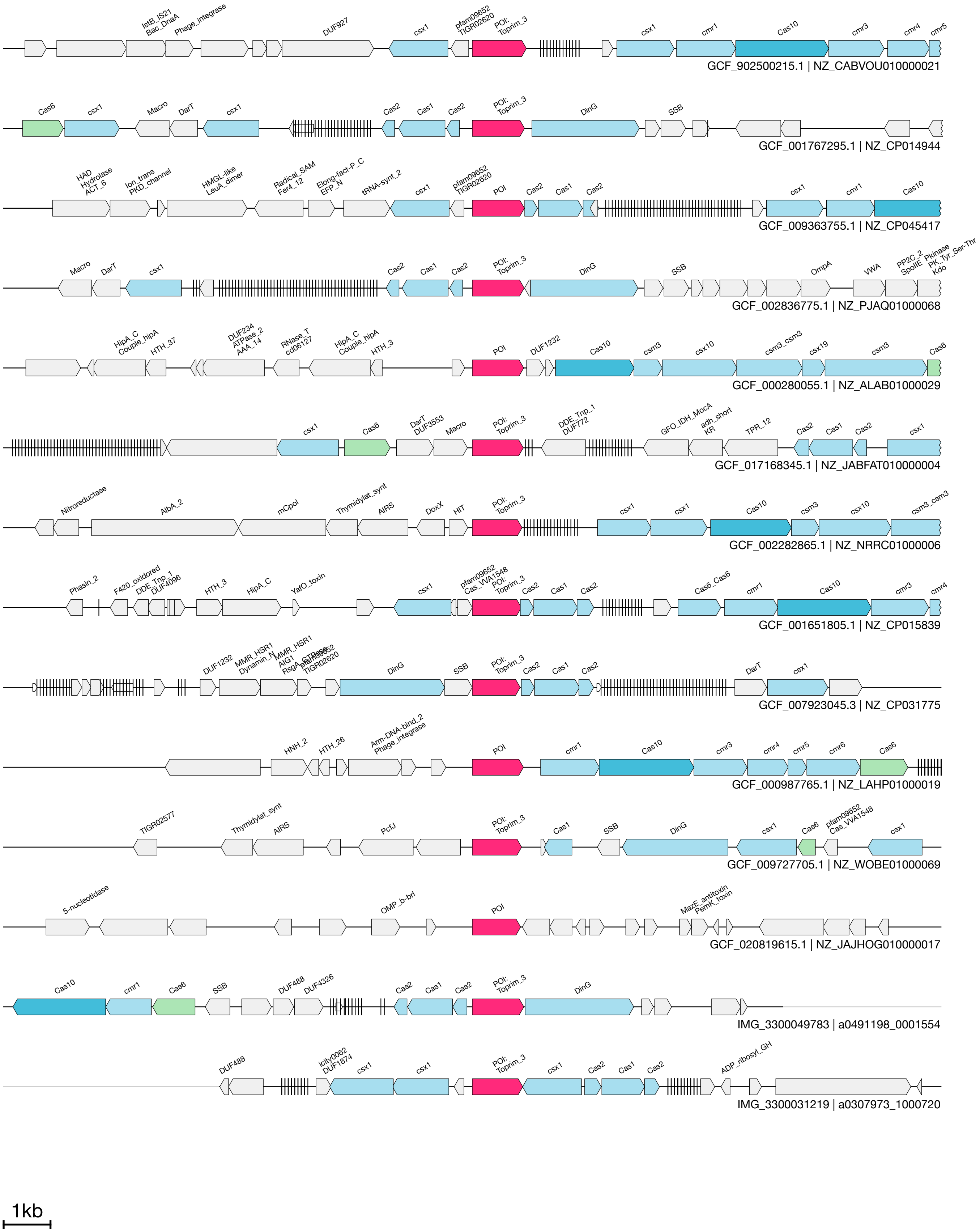

DF

UAS-108 **(DUF433)** 7 / 16.3

Auxiliary

IMG_3300000574&&7J11328_10007417&&1272_1539_-1

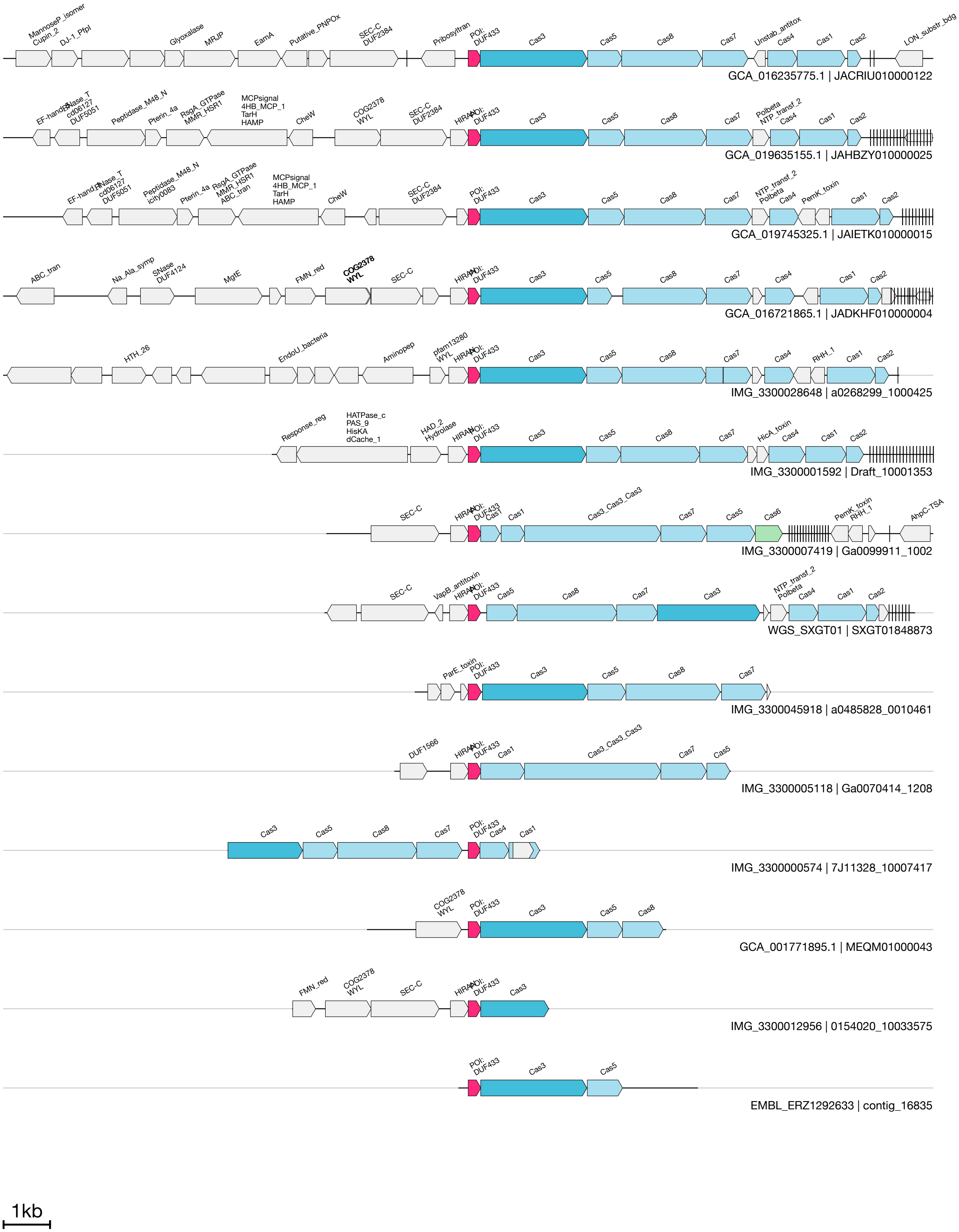

DG

UAS-109
Auxiliary

(DUF4407 + transpeptidase)
IMG_3300000574&&7J11328_10033202&&130_1651_1

2 / 5.3

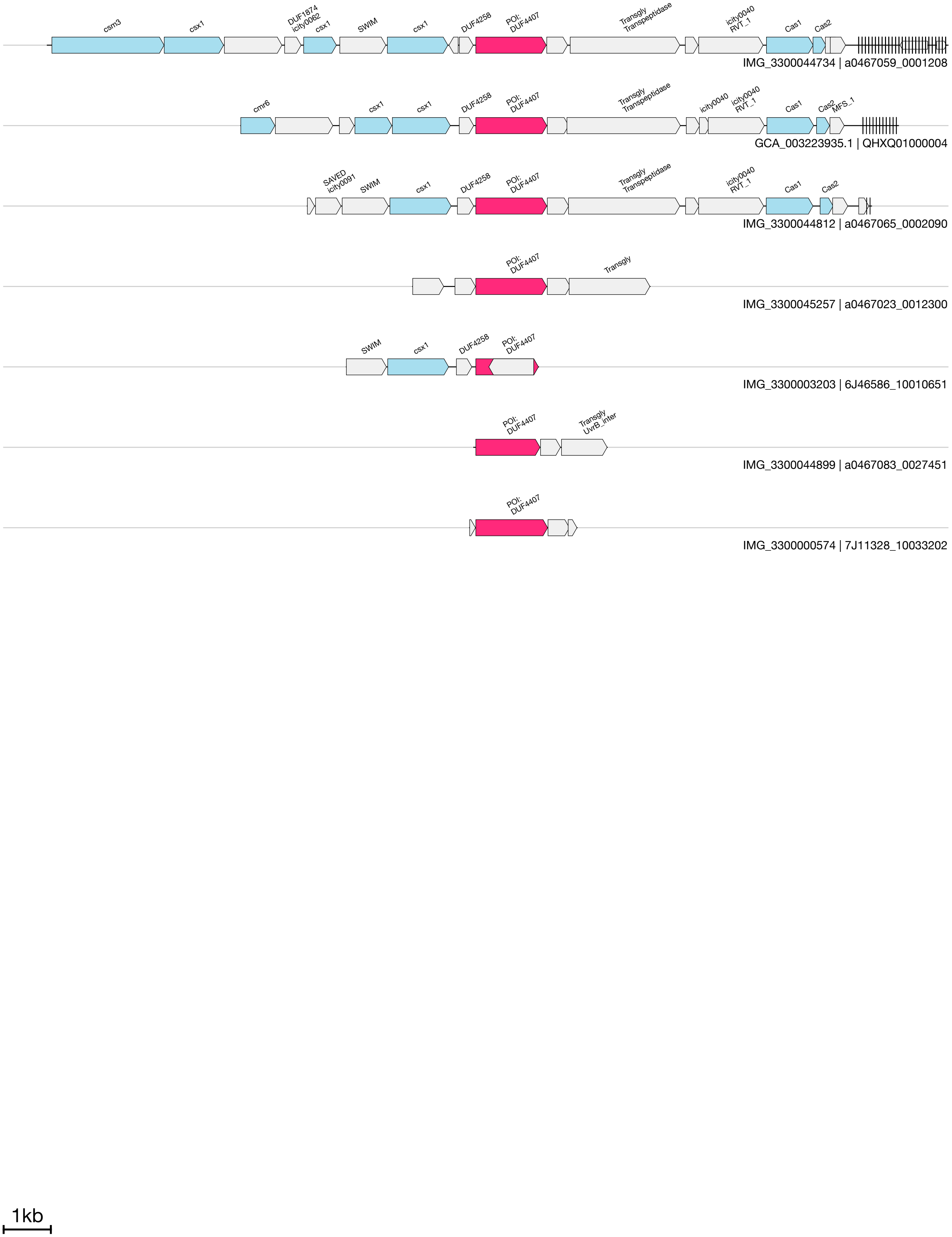

DH

UAS-110 **(mega peptidase)** 2 / 3.5
Auxiliary IMG_3300001598&&EMG_10018732&&309_3072_1

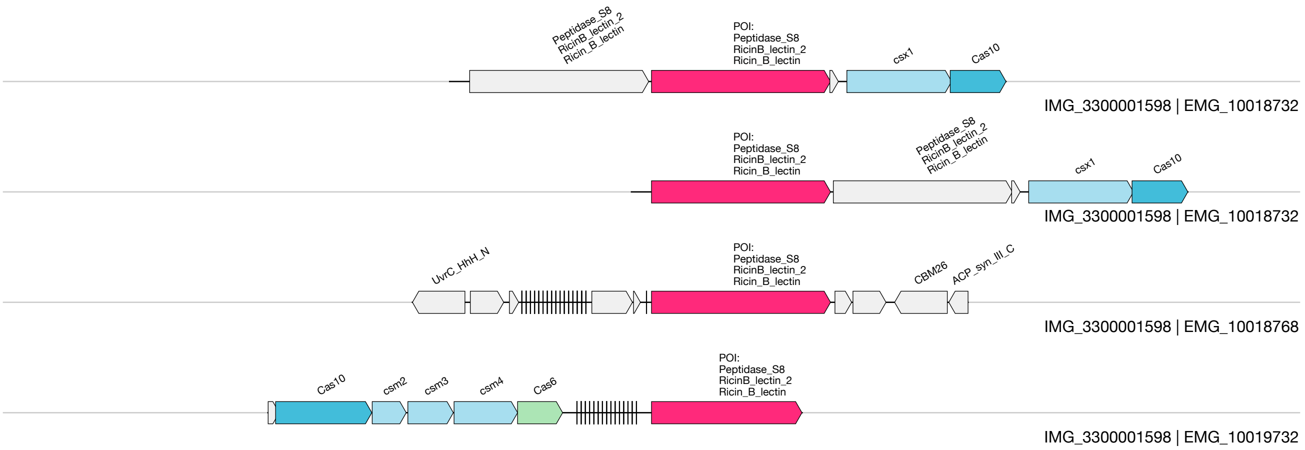

1kb

DI

UAS-111 **(TIR cascade)** 2 / 4.4

Auxiliary IMG_3300002223&&7J26845_10007859&&1305_2472_1

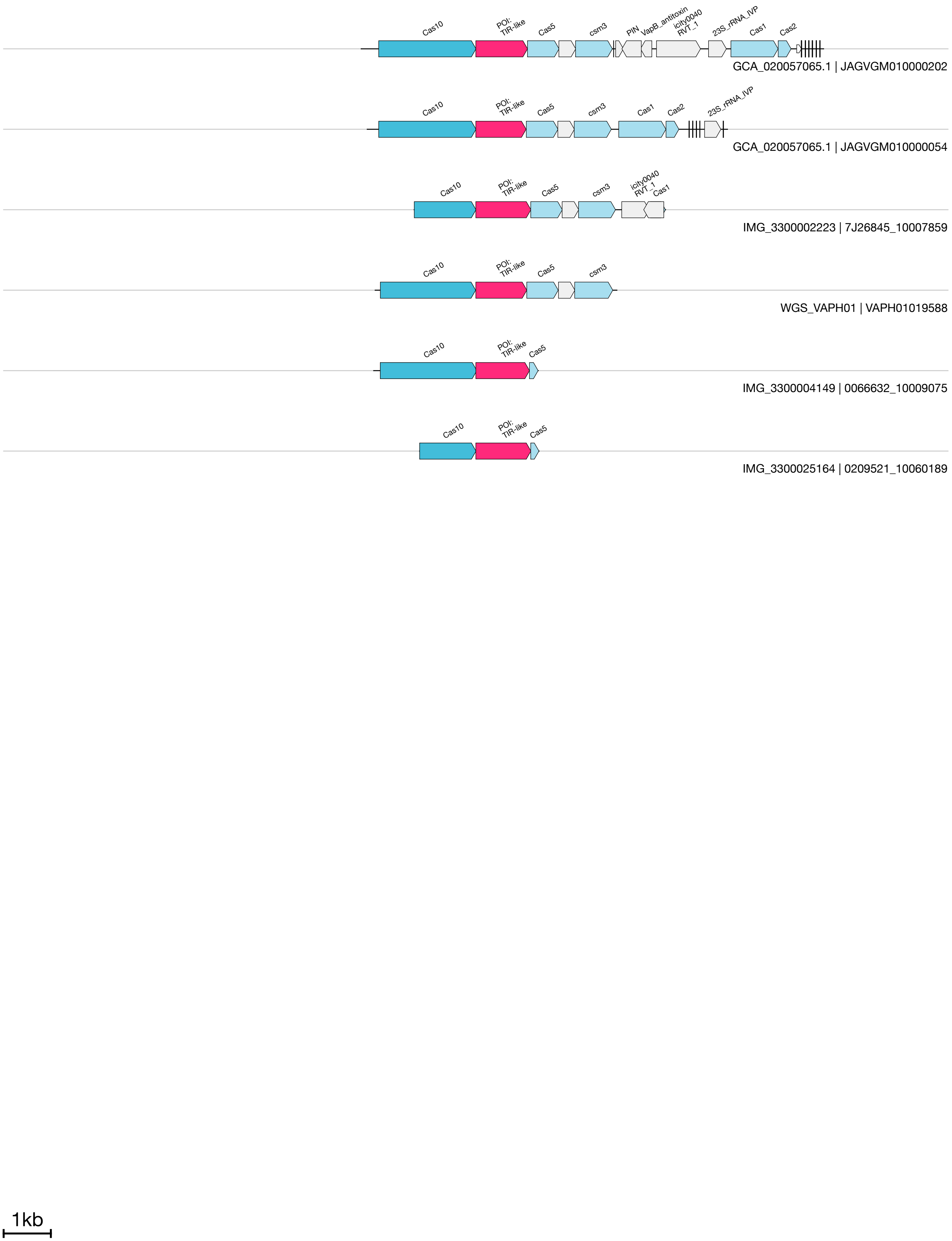

DJ

UAS-29
Auxiliary

(Phage RNAPol)
IMG_3300003177&&DffDRAFT_1003423&&1014_3828_-1

4 / 8.6

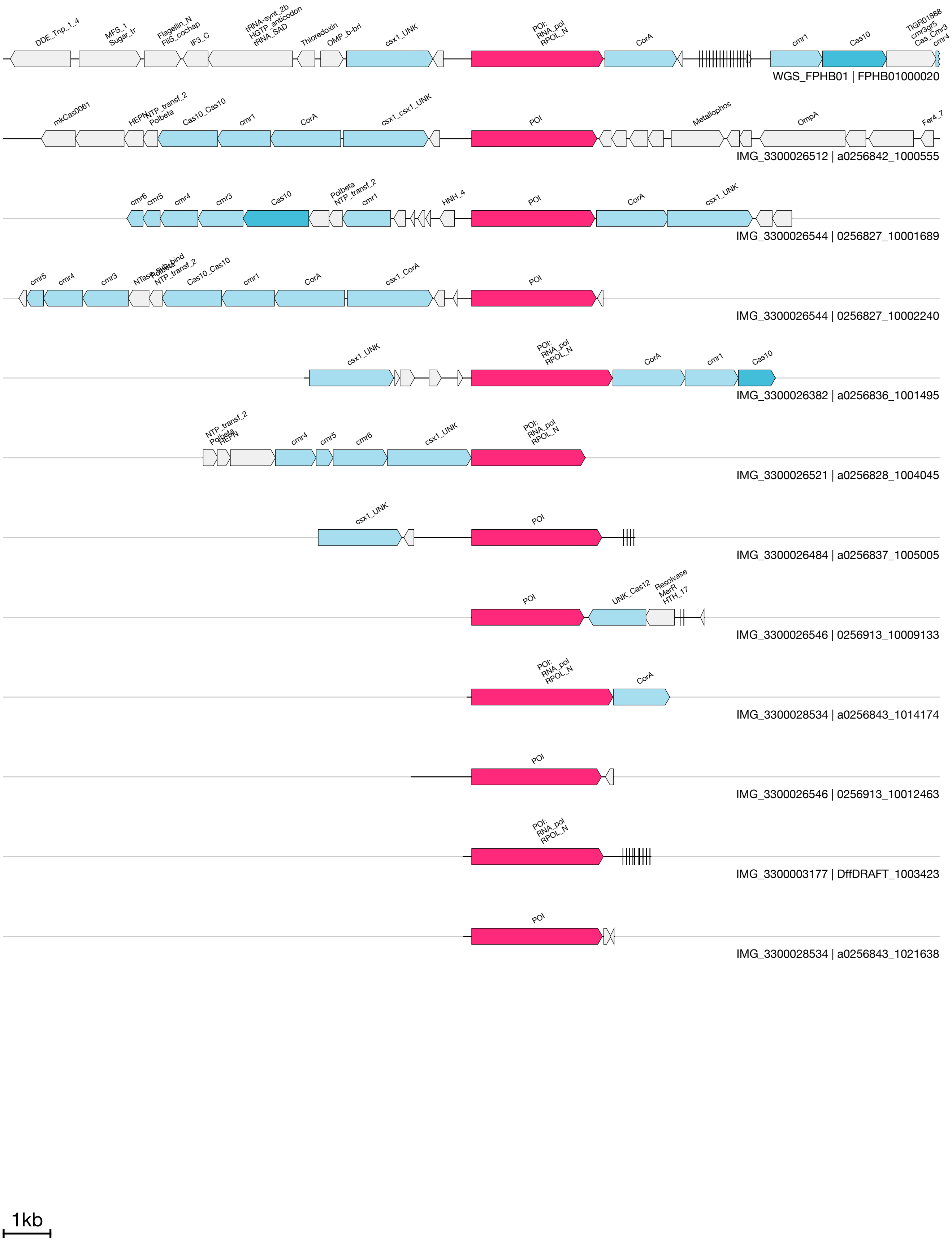

DK

UAS-112 **(Csx20 operonized with Alpha Amylase)** N/A

Auxiliary IMG_3300004628&&a0070399_1037568&&498_2109_1

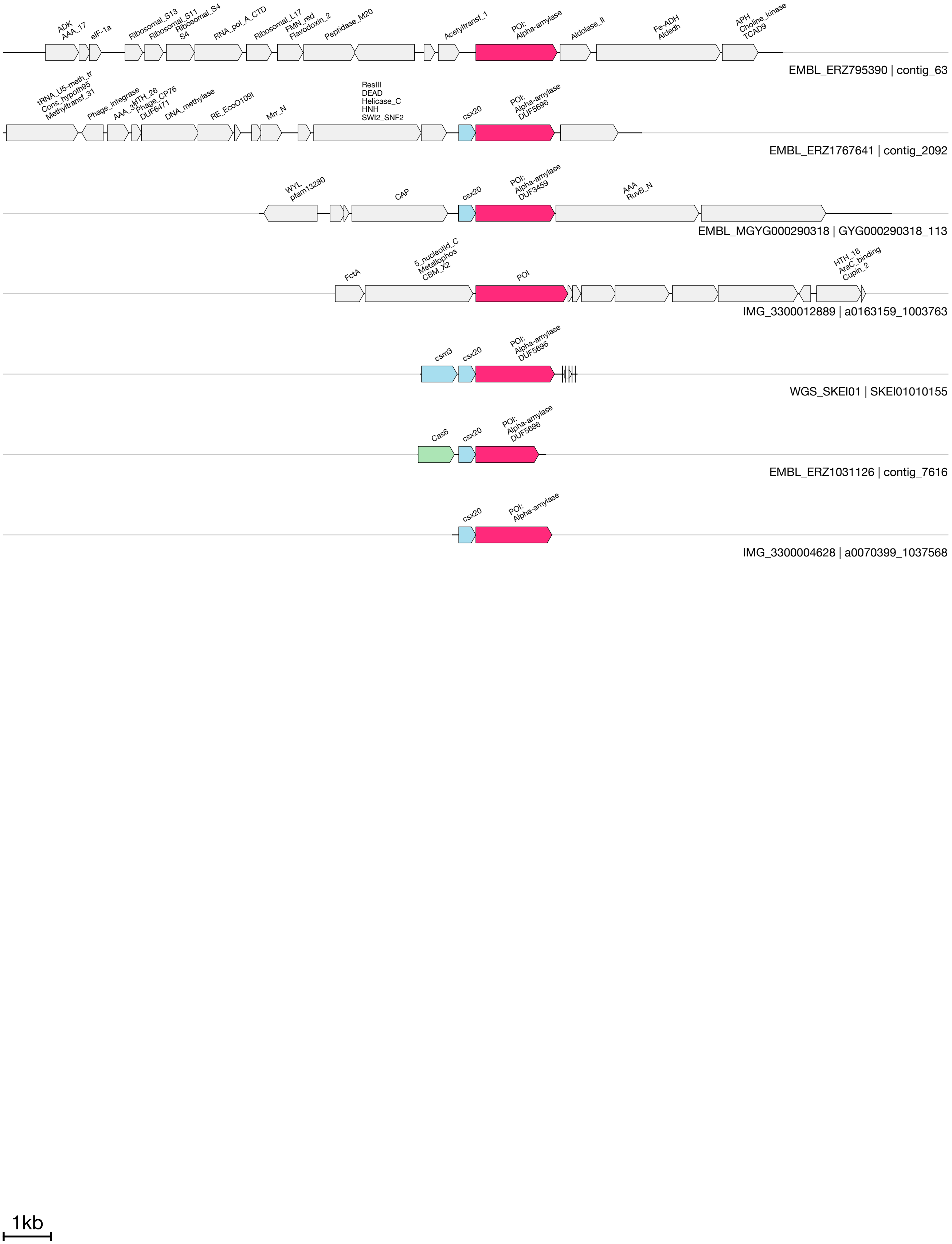

DL

UAS-113 **(RNaseH)** 10 / 14.6
Auxiliary IMG_3300005573&&a0078972_1000107&&101916_102516_1

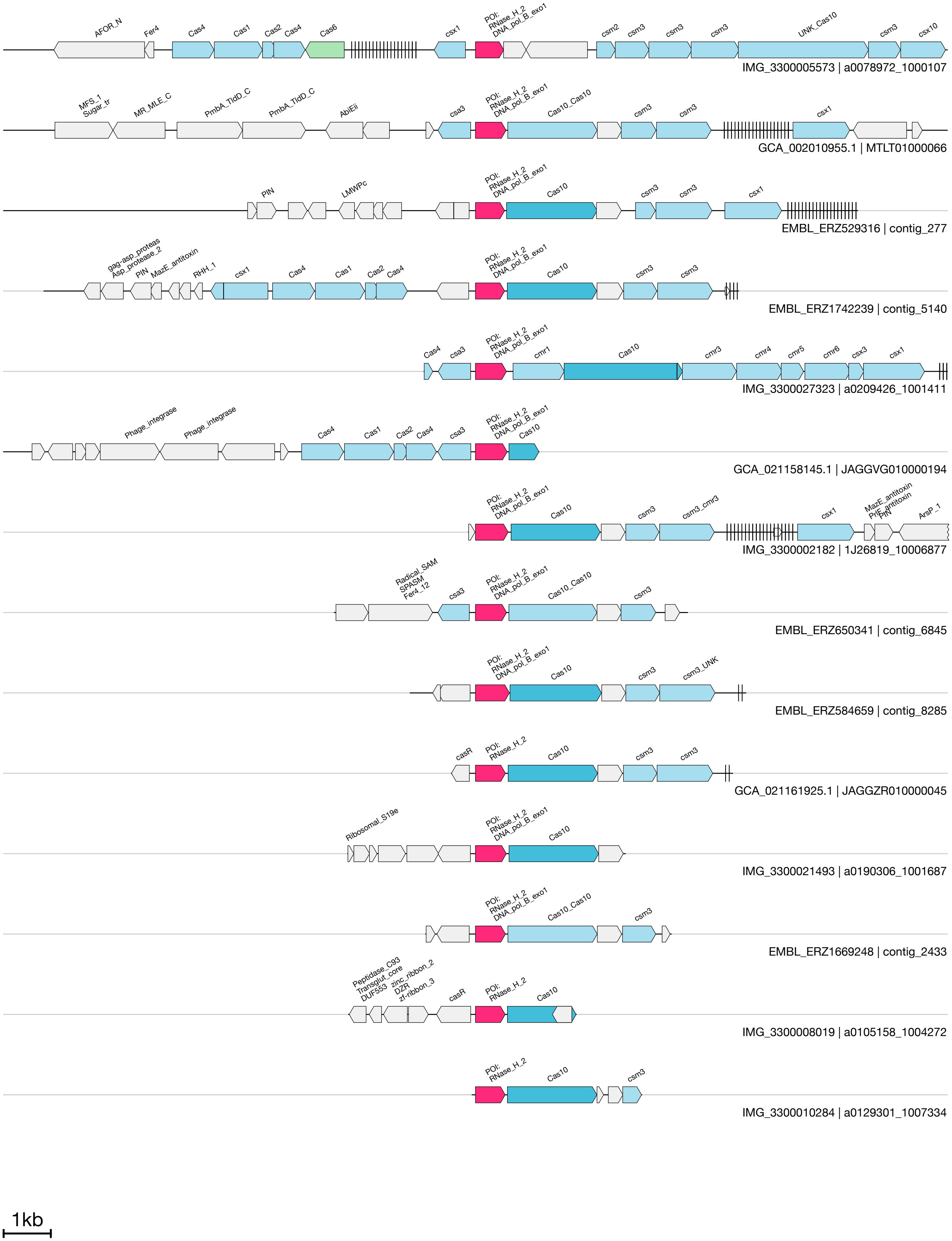

DM

UAS-114 **(Tfb2_Cas3Cterminus_WYL)** 5 / 5.5

Auxiliary IMG_3300007521&&0105044_10068709&&481_2728_1

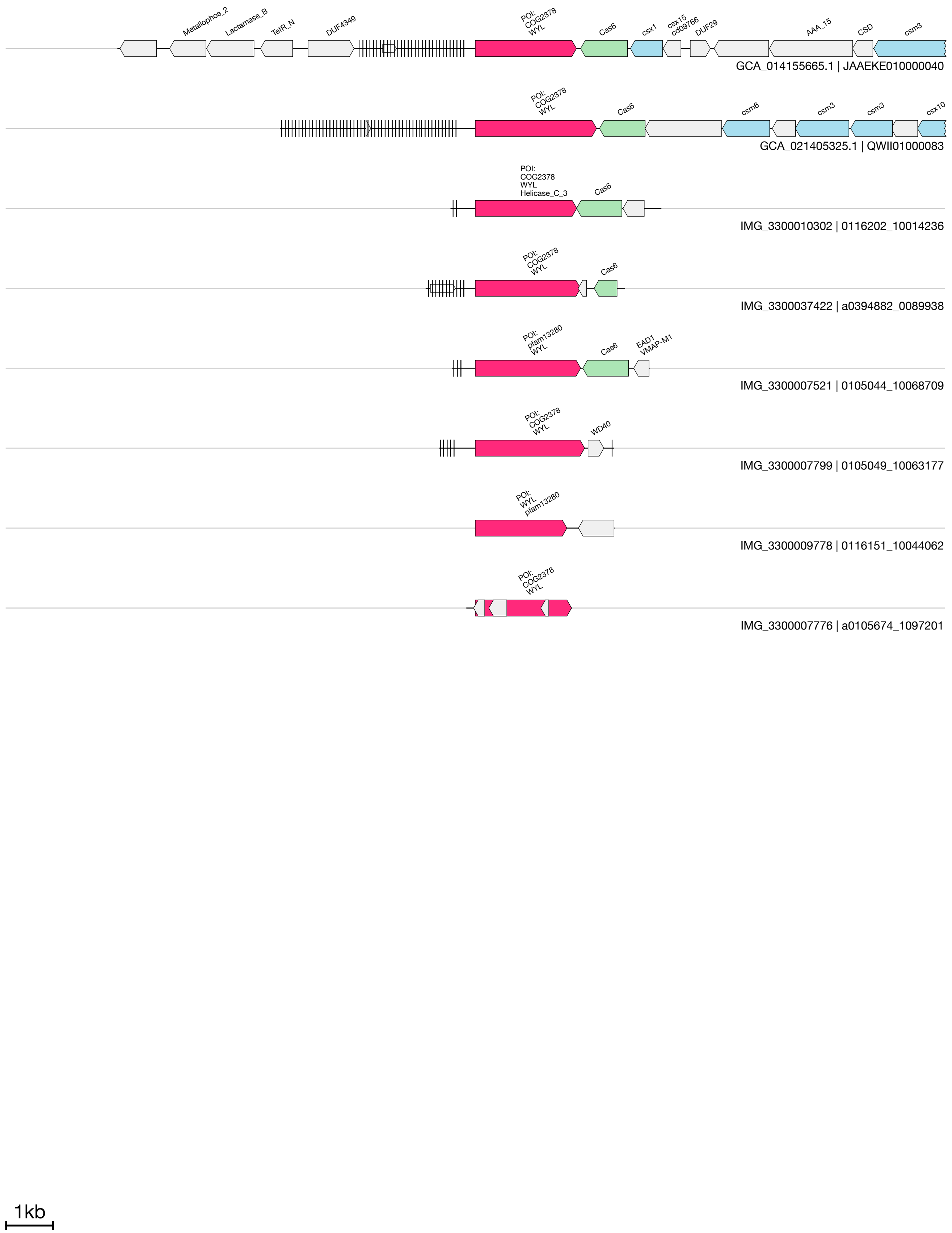

DN

UAS-115 **(SLATT)** N/A
Auxiliary IMG_3300007964&&a0100400_1019481&&1335_1944_-1

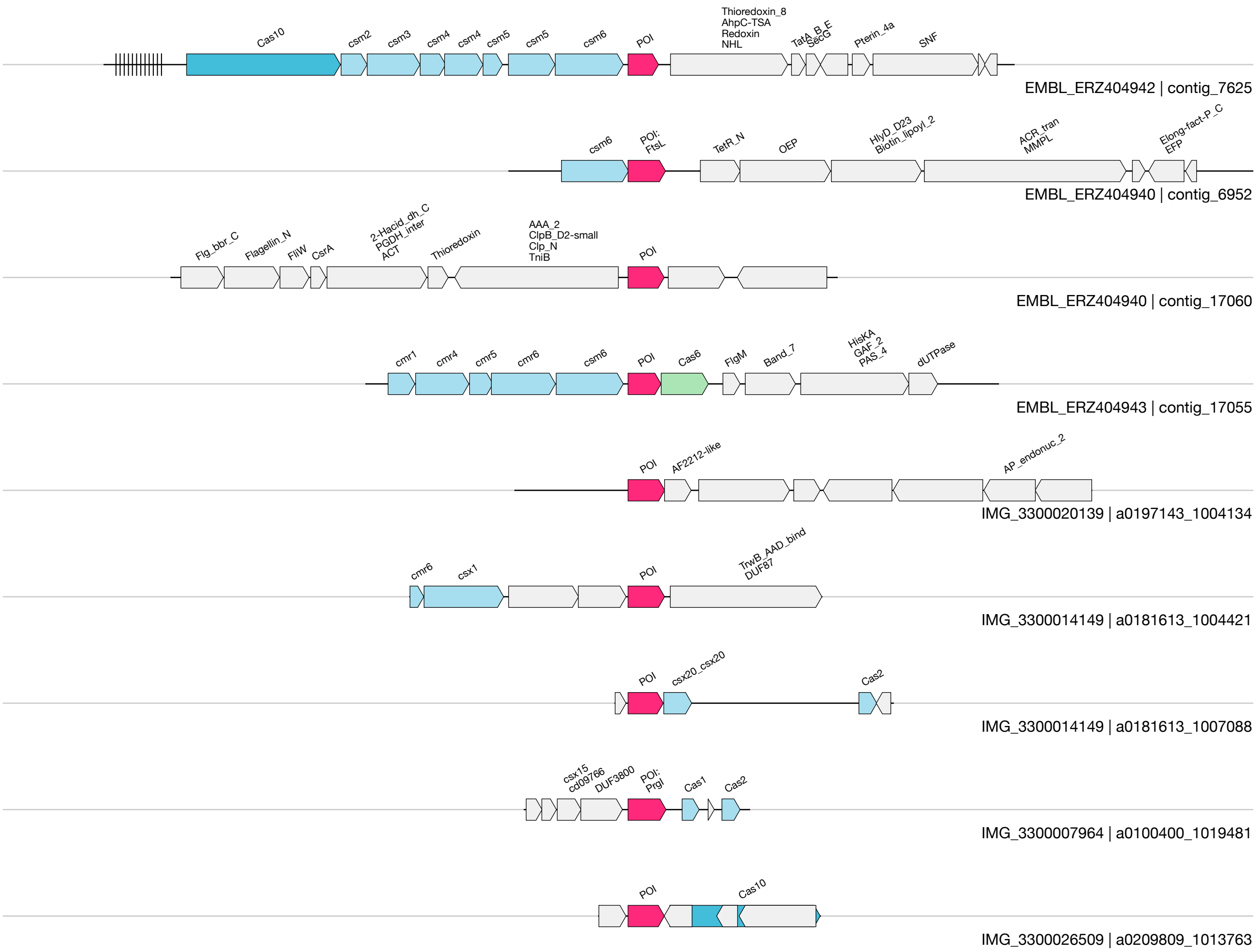

1kb

DO

UAS-116 **(Moca oxoreductase + SDR)** 21 / 38.1
Auxiliary IMG_3300008252&&0105357_10033588&&329_1373_1

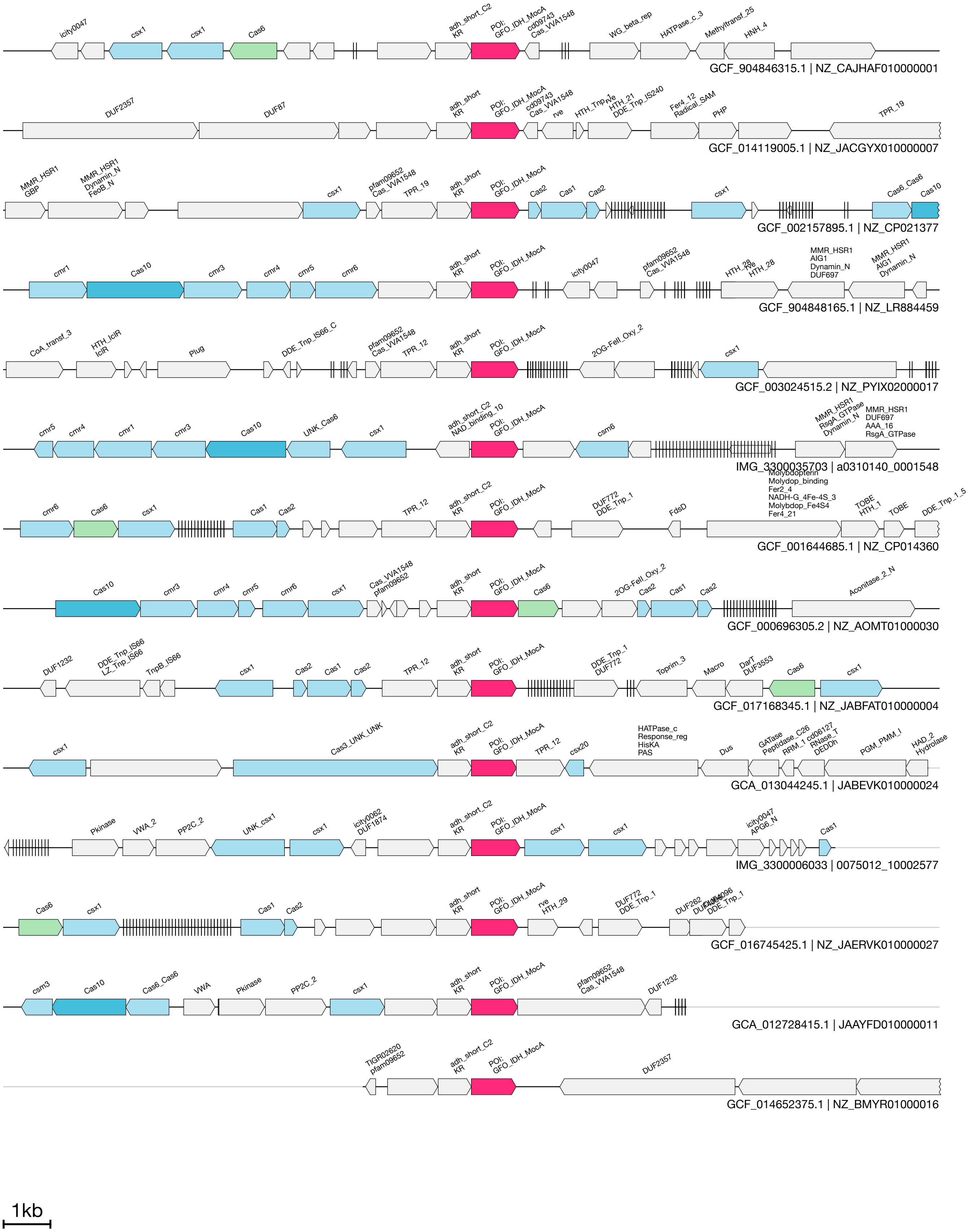

DP

UAS-117 **(DUF4231)** 3 / 4.8

Auxiliary IMG_3300009032&&0105048_10143319&&1427_2051_-1

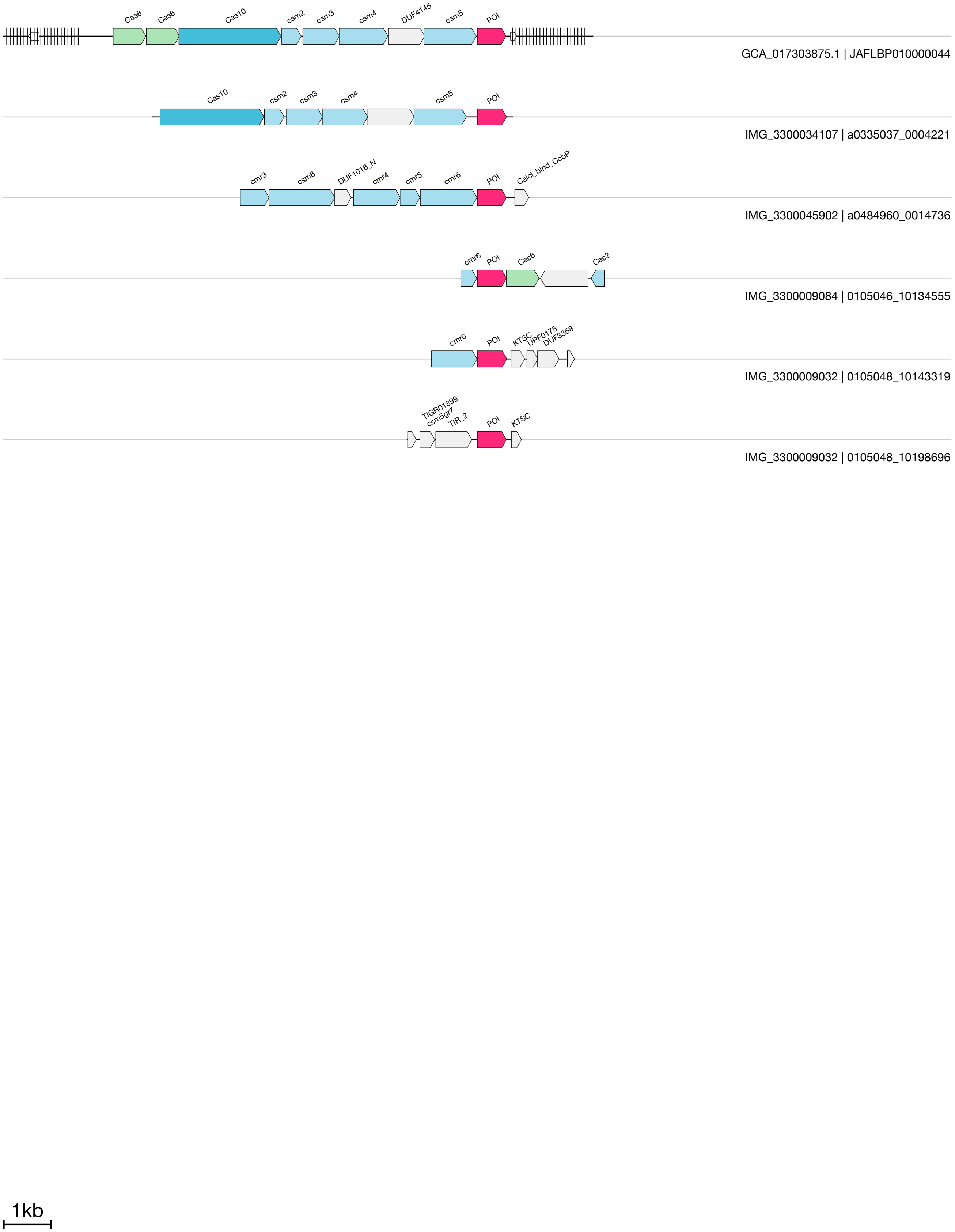

DQ

UAS-118 **(DUF3891)** 12 / 23.7
Auxiliary IMG_3300009083&&0105047_10063153&&274_994_-1

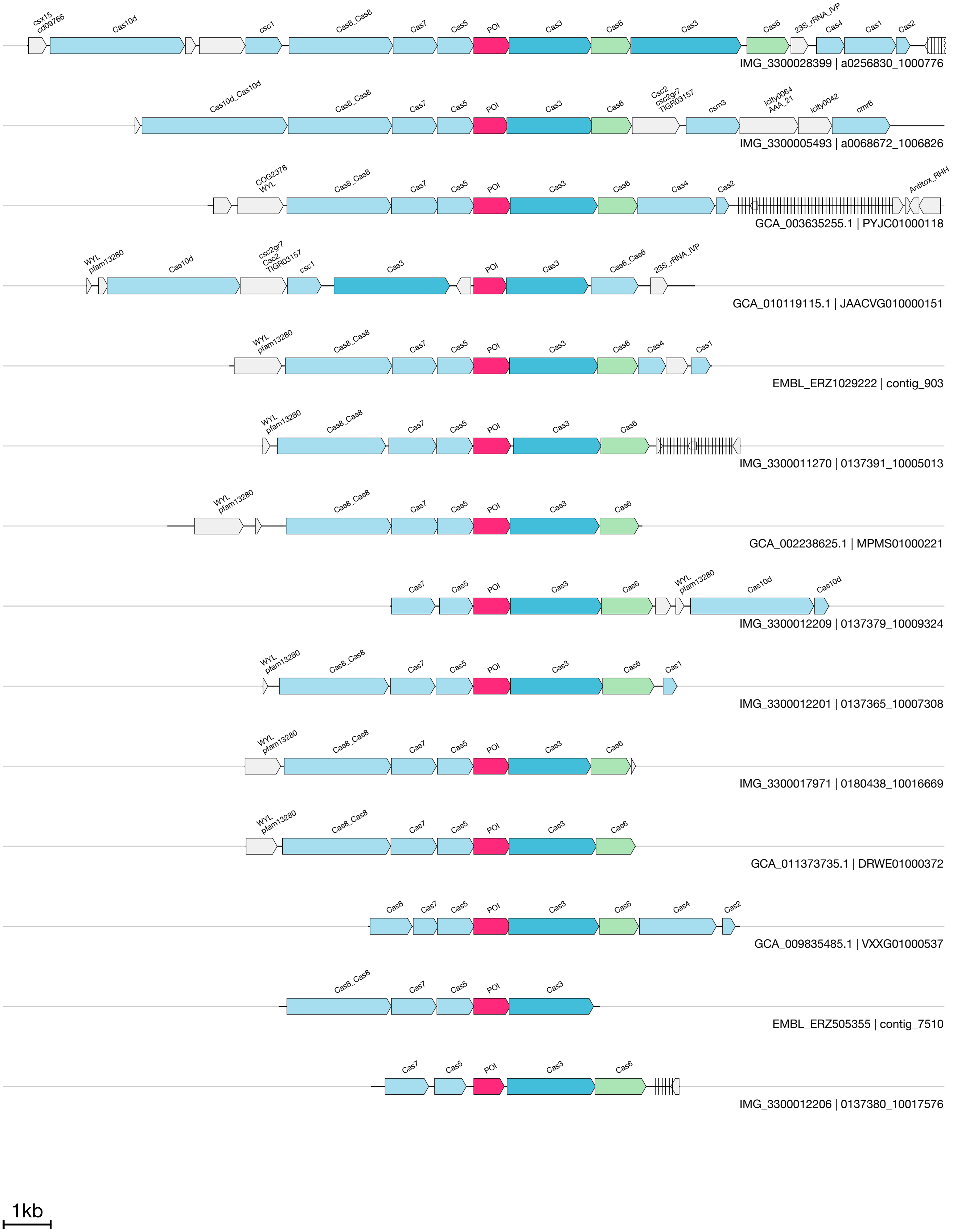

DR

UAS-119 | **(DUF5679, zinc ribbon)** | 6 / 9.9

Auxiliary

IMG_3300009175&&0073936_10076949&&1196_1451_-1

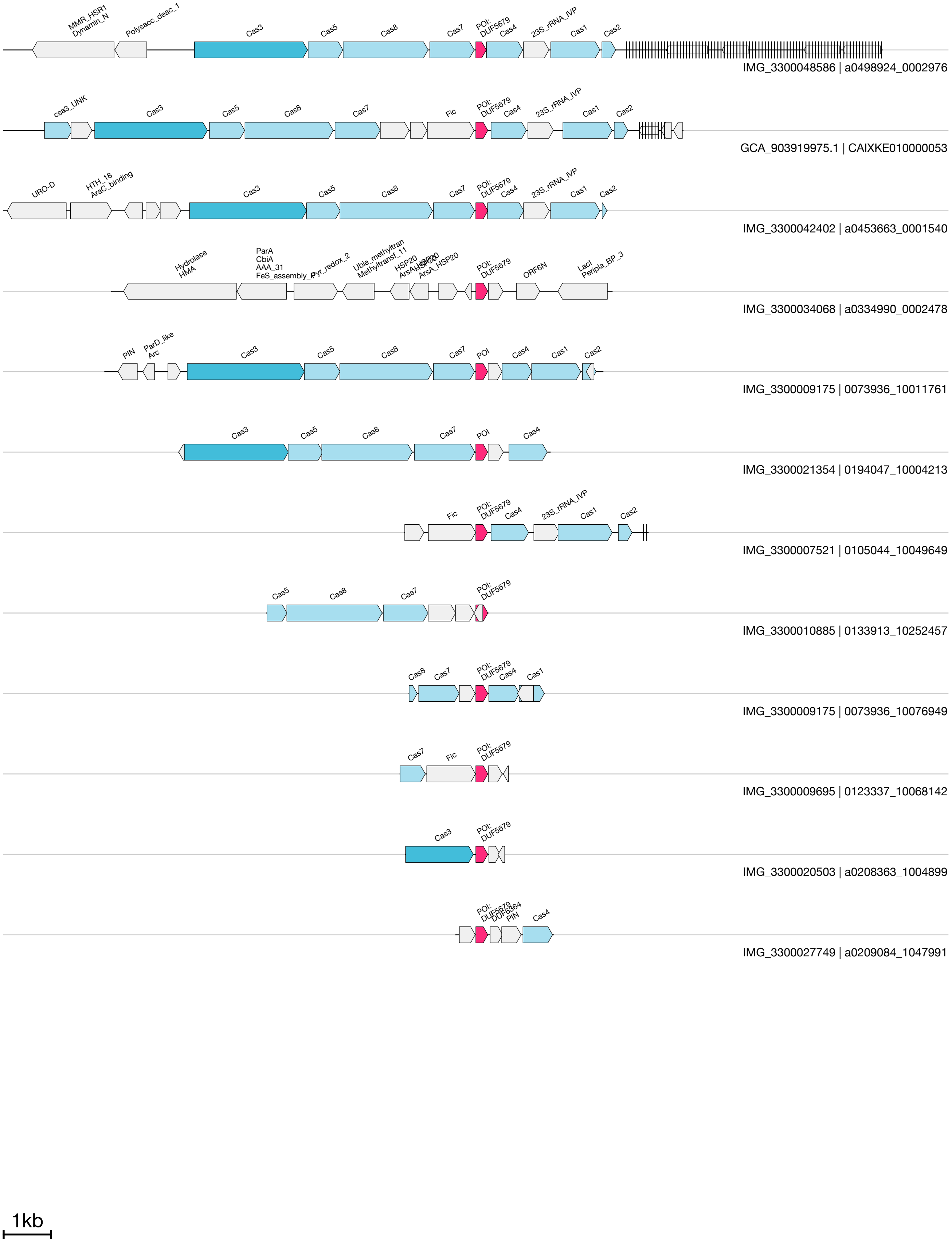

DS

UAS-120 **(DUF2225)** 2 / 4.1
Auxiliary IMG_3300009503&&0123519_10124867&&69_1659_-1

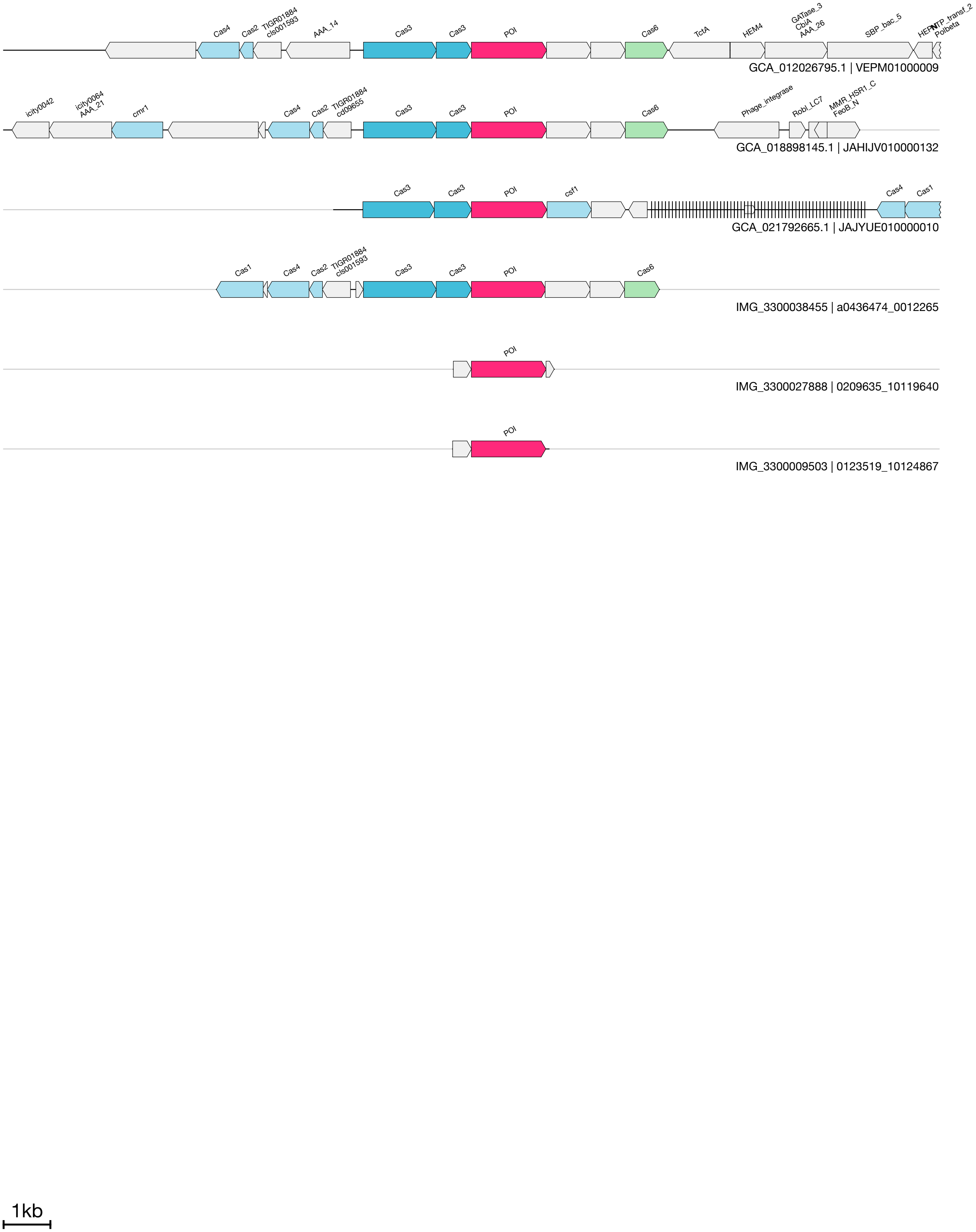

DT

UAS-121
Auxiliary

(RNAPol + Sigma_factor + unknown gene)
IMG_3300009585&&a0105160_1000939&&4188_8637_1

15 / 41.0

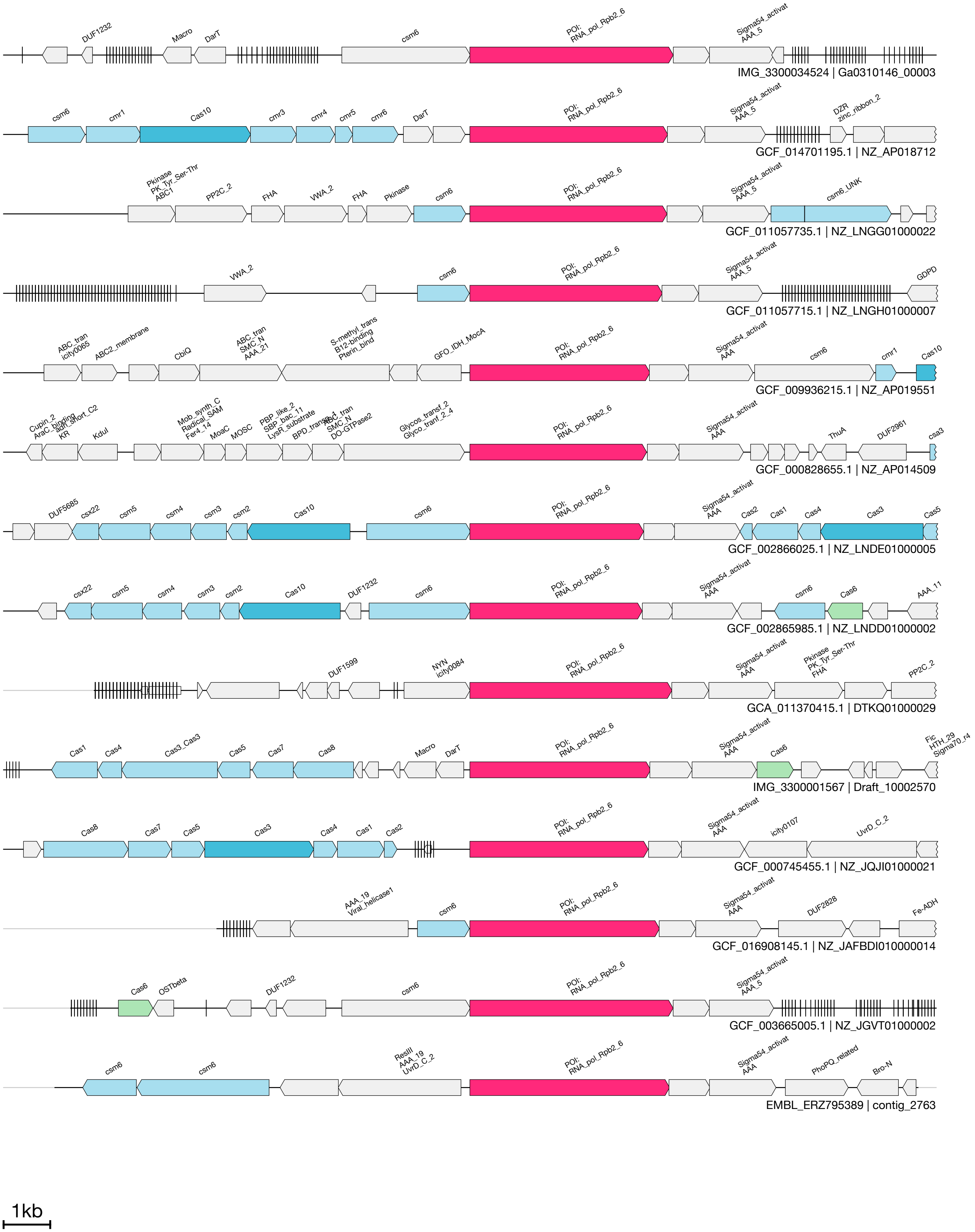

DU

UAS-122 **(PRiA3 + zf)** 15 / 36.2
Auxiliary IMG_3300012931&&0153915_10003429&&308_1193_1

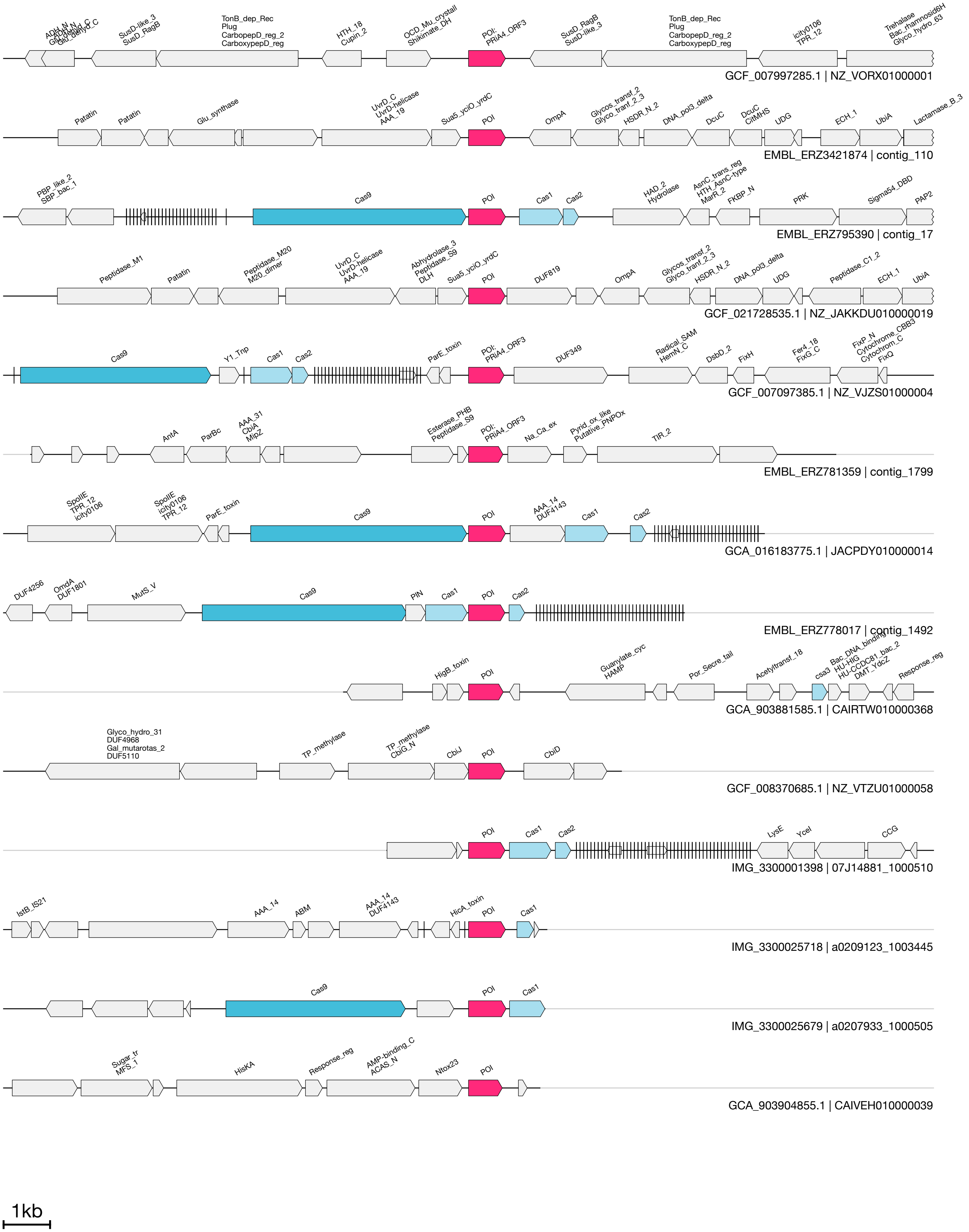

DV

UAS-22 **(Cut8_TPR)** 3 / 8.1
Auxiliary IMG_3300012943&&0164241_10011111&&1329_2733_-1

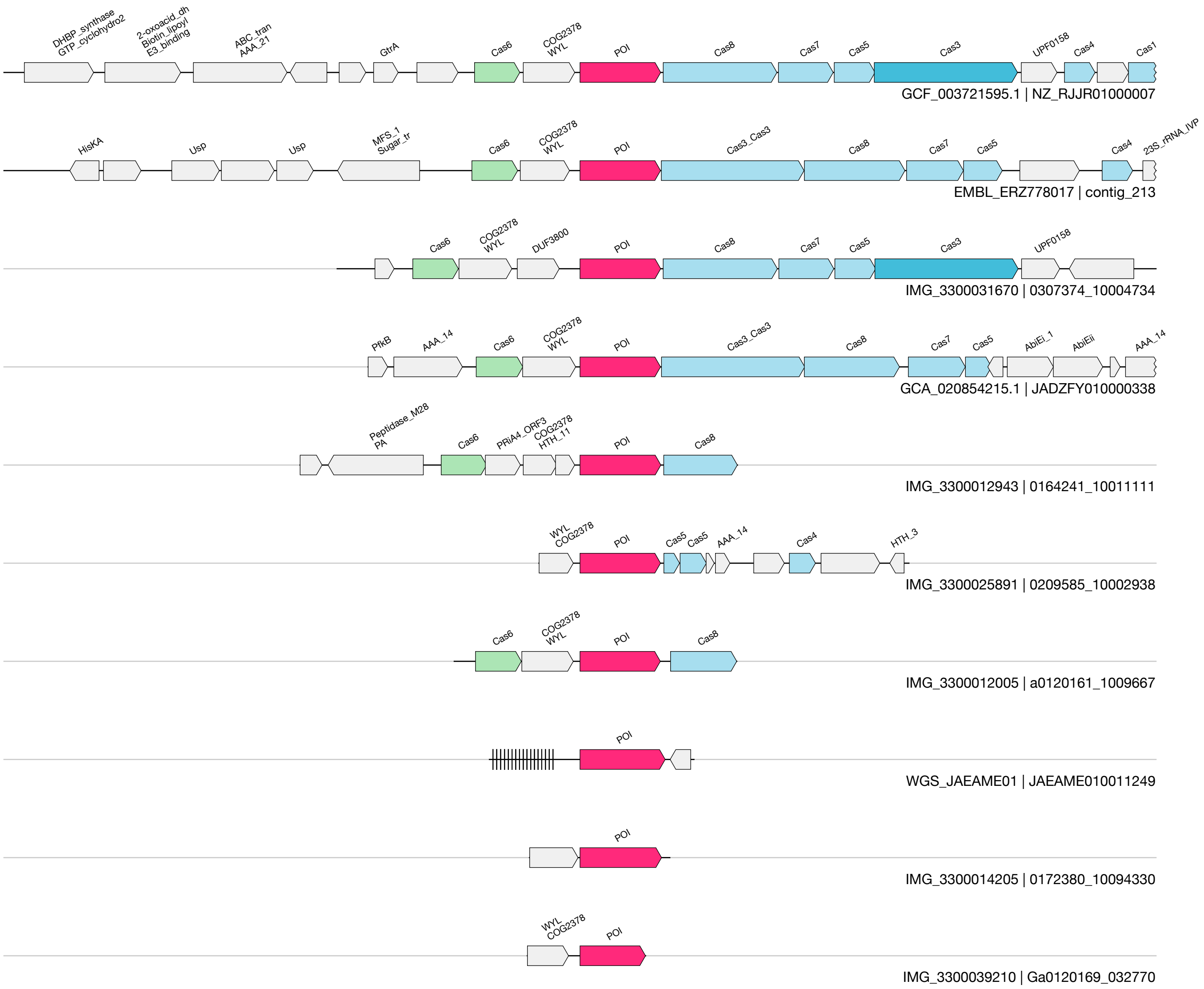

1kb

DW

UAS-21 **(Cas13b + CorA)** 3 / 3.7

Auxiliary IMG_3300012983&&0123349_10003896&&1100_3251_1

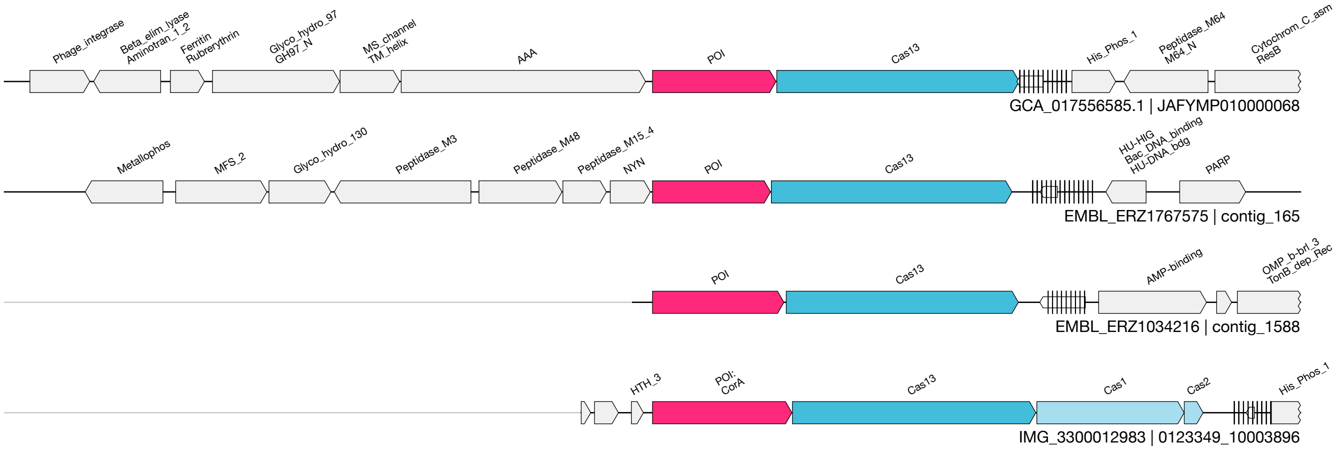

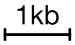

DX

UAS-123 **(NACHT)** 2 / 3.2

Auxiliary IMG_3300014149&&a0181613_1011337&&962_3059_-1

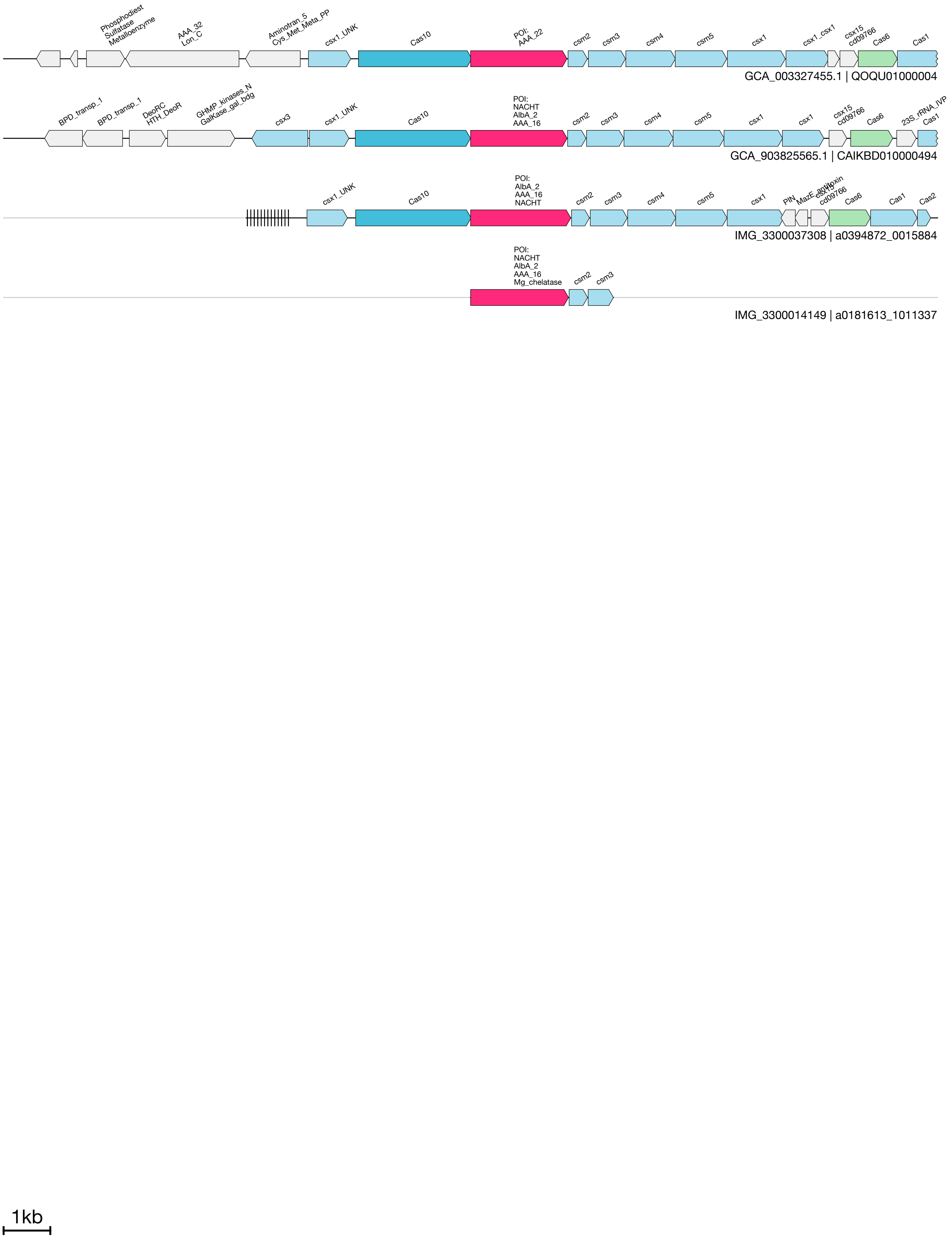

DY

UAS-124
Auxiliary

(unknown)
IMG_3300014153&&a0181527_1048931&&1181_2084_-1

3 / 3.3

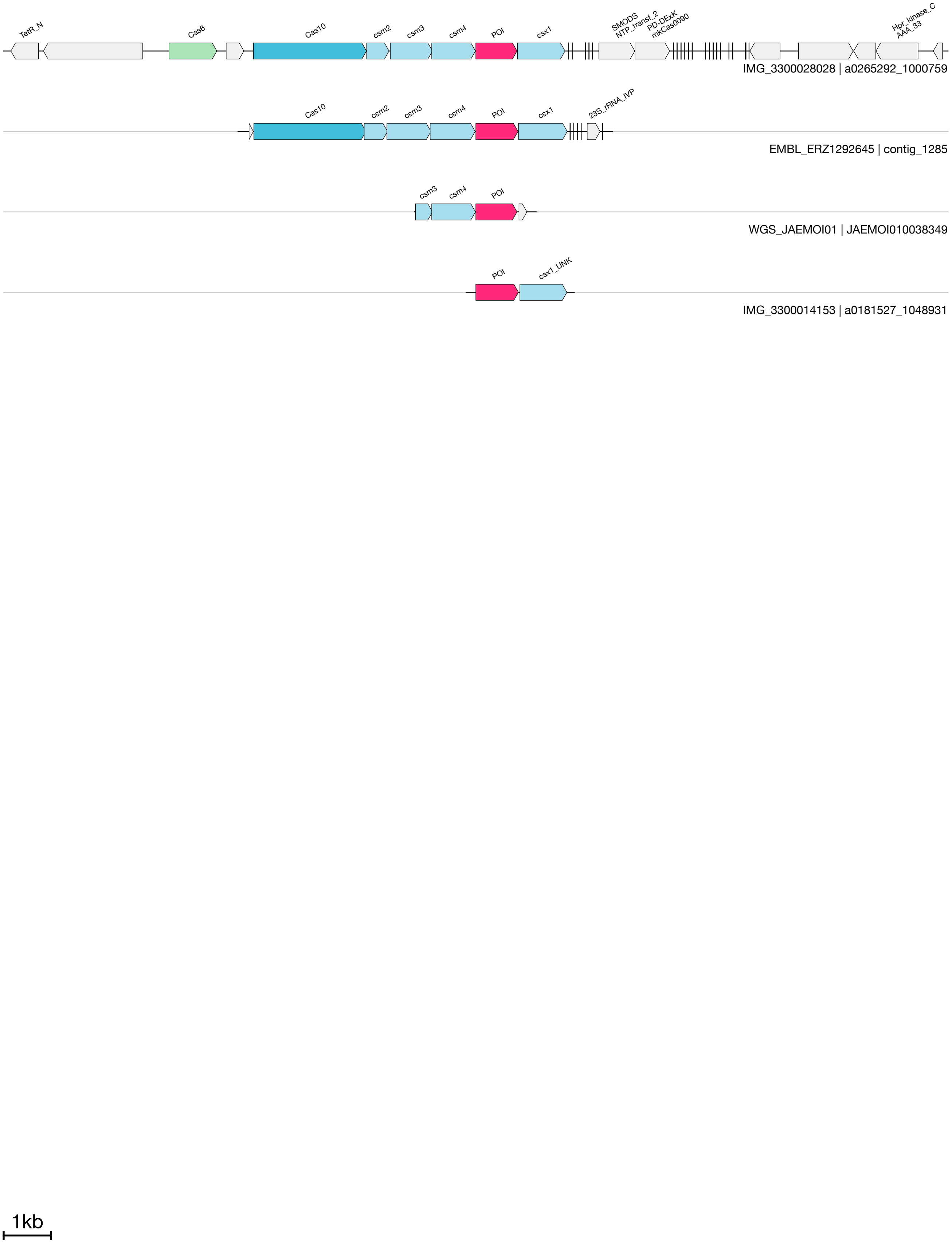

DZ

UAS-125 **(UPF0158)** 11 / 24.8
Auxiliary IMG_3300015214&&0172382_10011895&&7267_8365_1

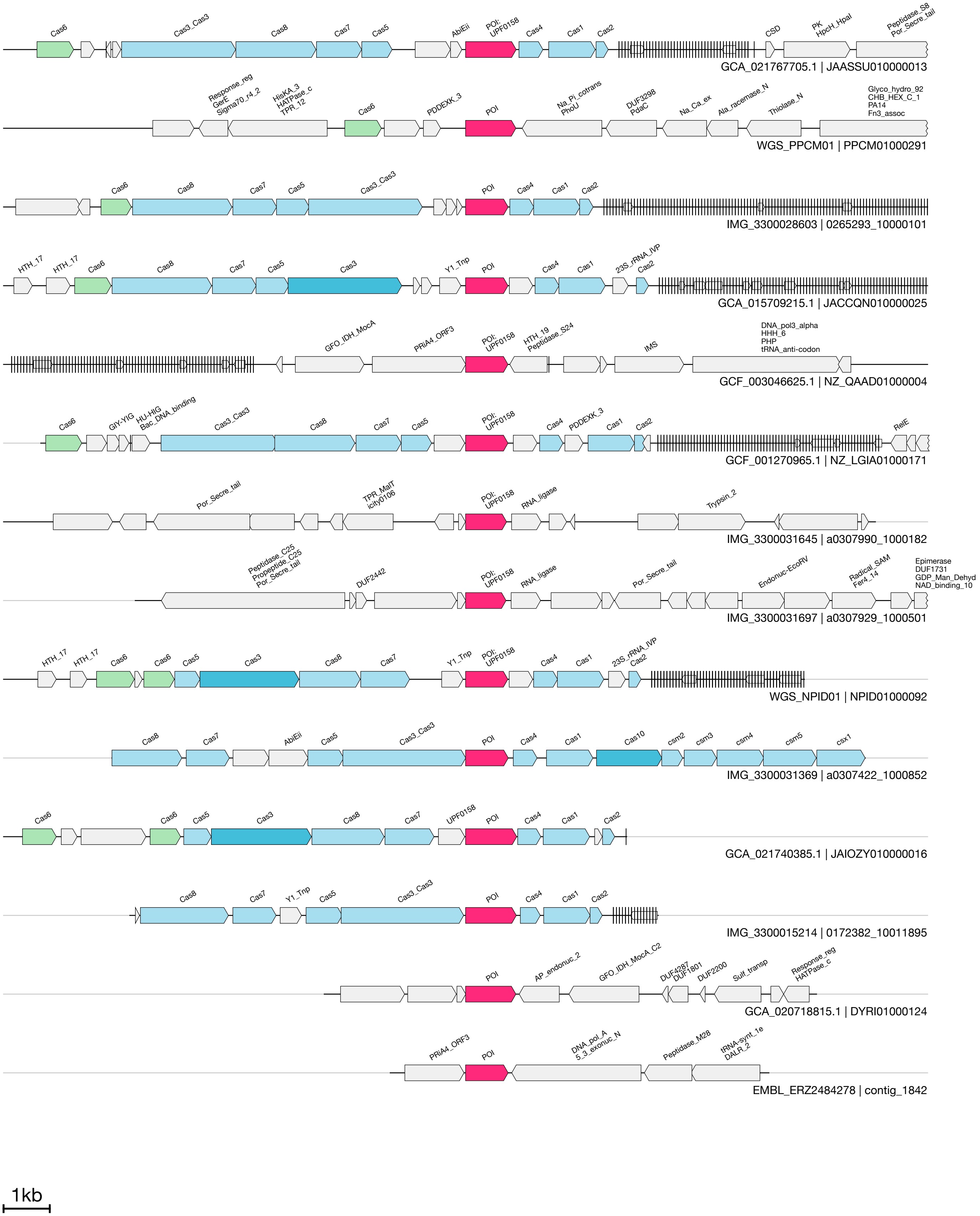

EA

UAS-126 **(Cut8)** 3 / 6.4
Auxiliary IMG_3300018012&&0187810_10022321&&90_1506_-1

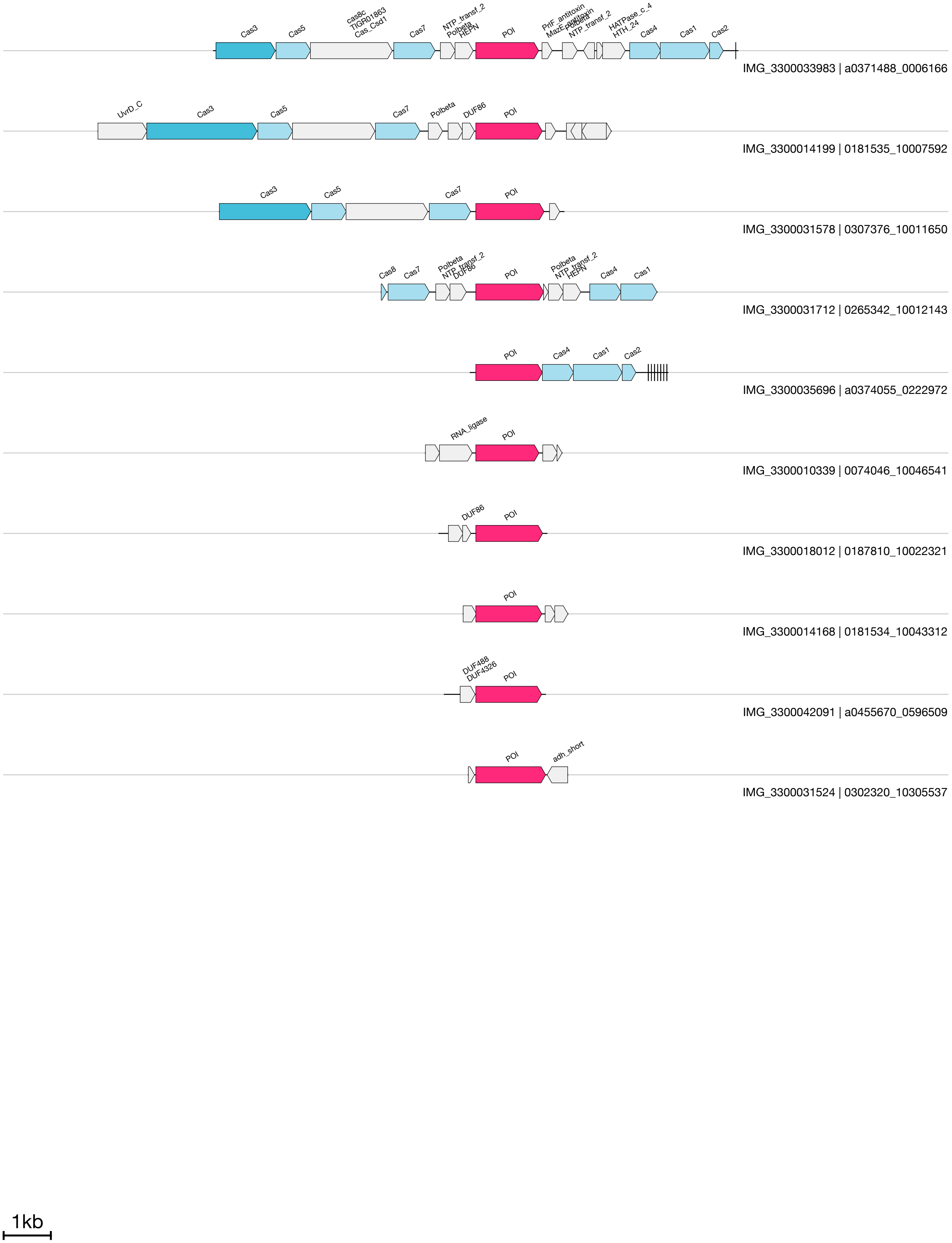

EB

UAS-26 | **(DUF87 + NurA 5' to 3' nuclease)** | 4 / 4.0
Auxiliary | IMG_3300018407&&a0187900_1000594&&12585_14151_-1

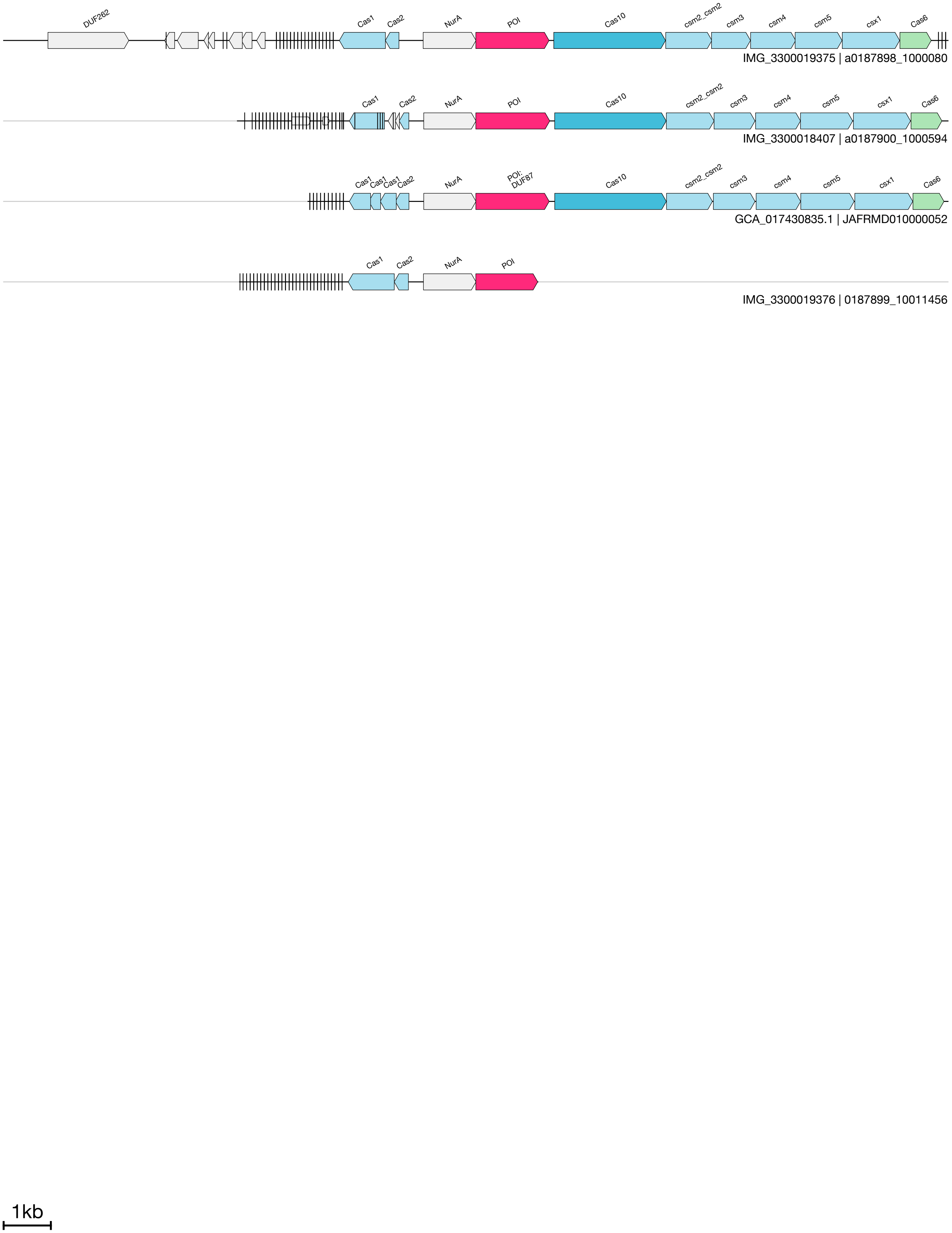

EC

UAS-127
Auxiliary

(NACHT)
IMG_3300020192&&0163147_10009373&&460_1681_-1

3 / 7.4

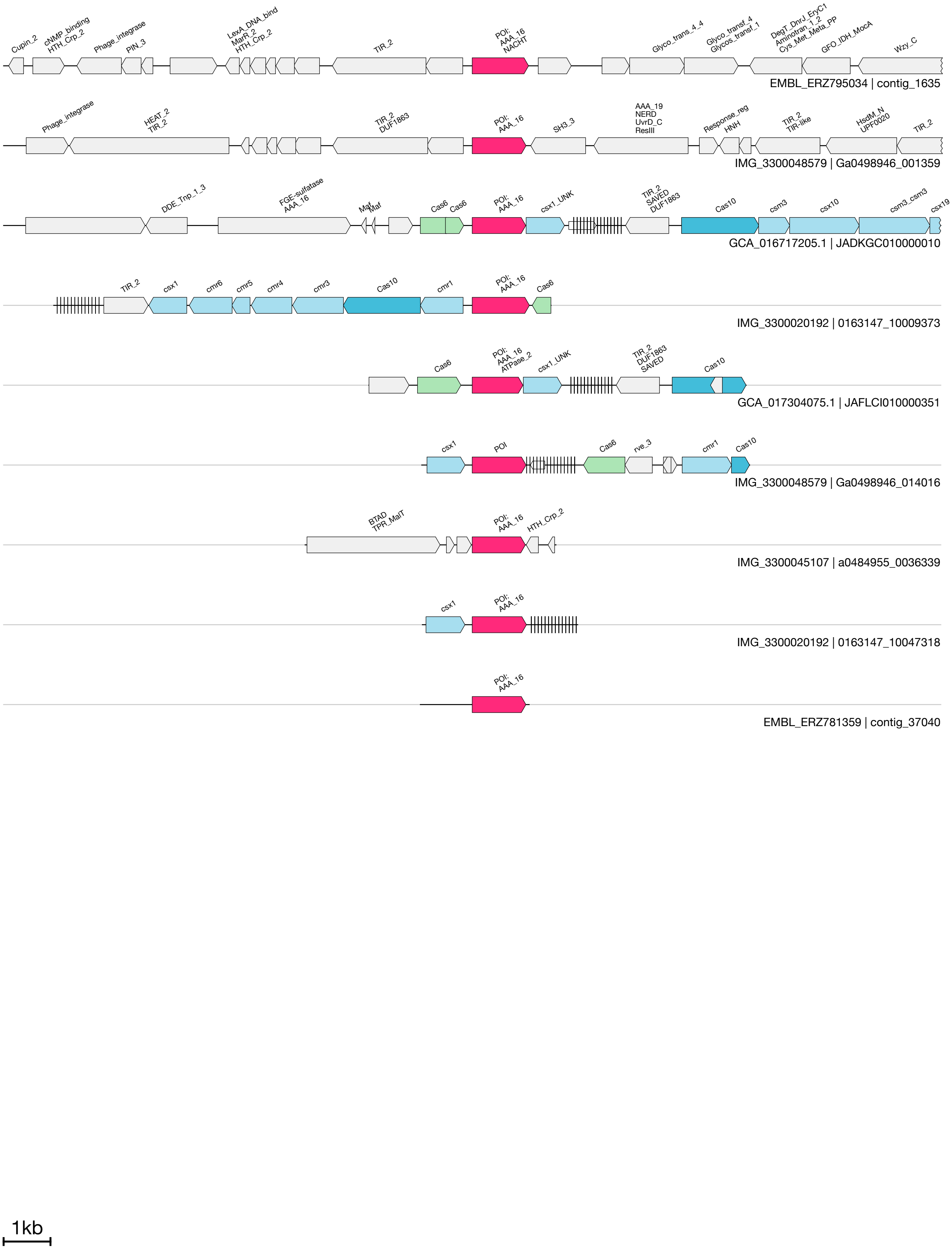

ED

UAS-128 **(Mrr nuclease)** N/A

Auxiliary IMG_3300024353&&a0209979_1002041&&5779_7255_-1

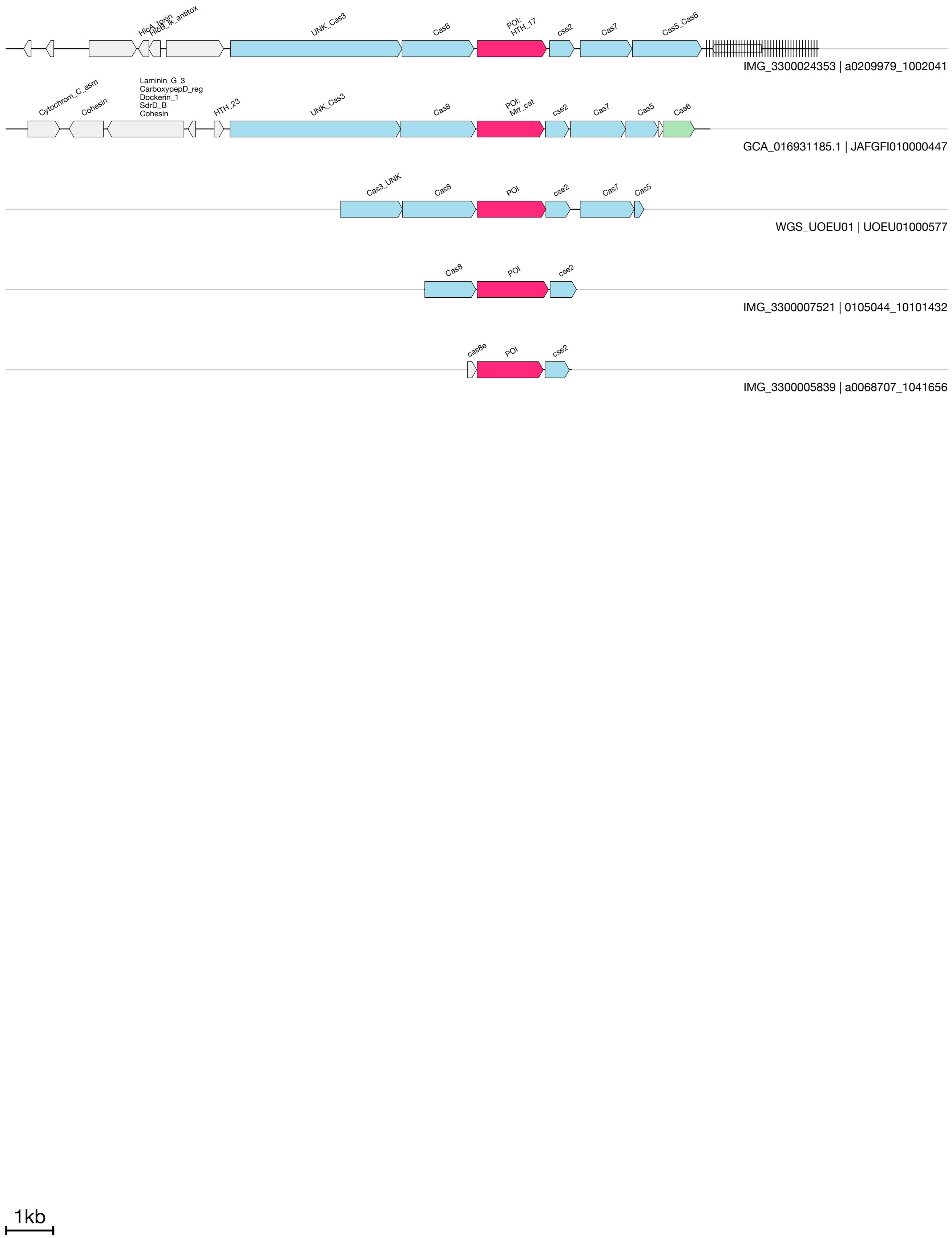

EE

UAS-129
Auxiliary

(Tubulin + other protein + vWa)
IMG_3300024423&&a0190286_1002039&&2400_5370_-1

16 / 43.6

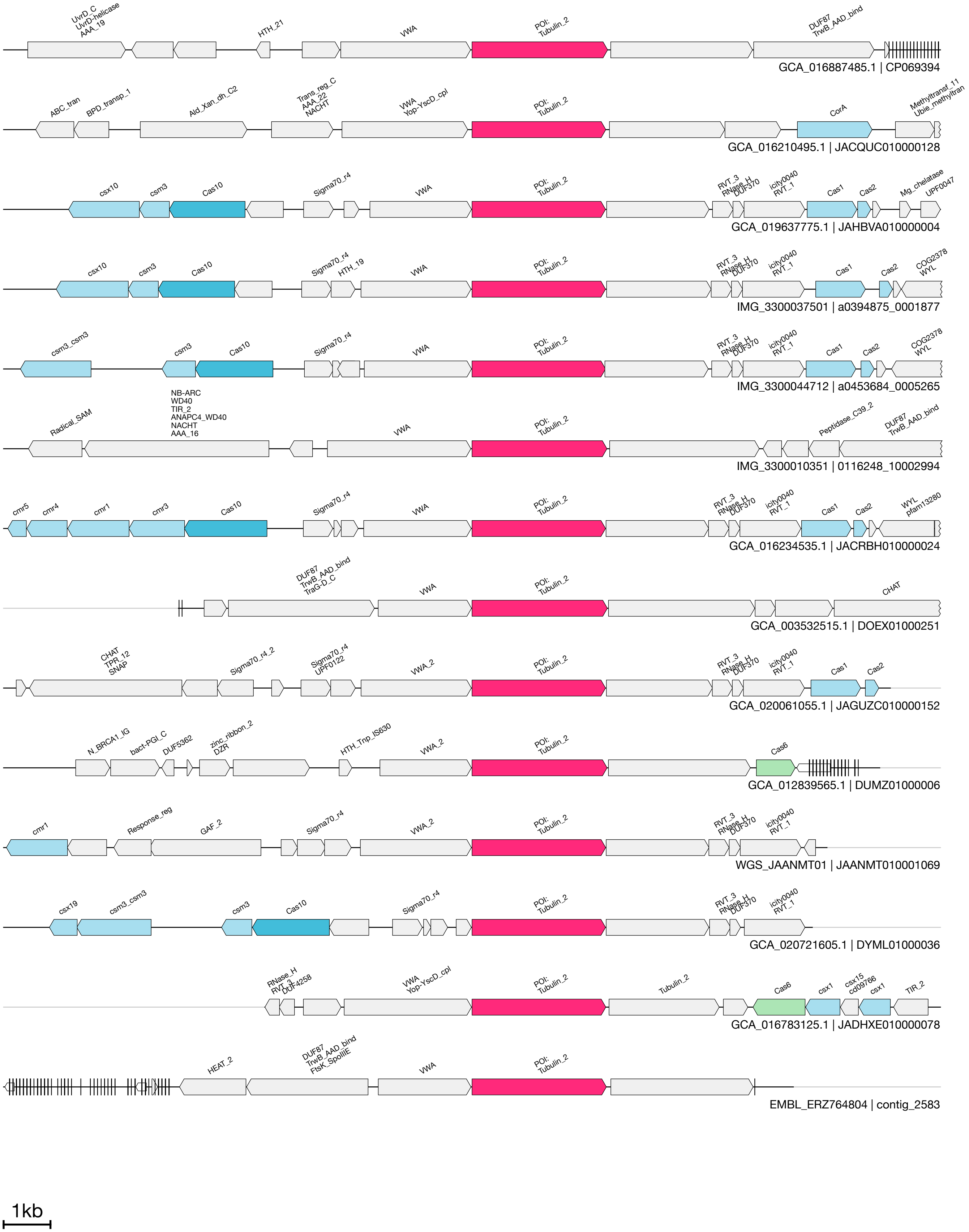

EF

UAS-130
Auxiliary

(Tubulin system)
IMG_3300025717&&a0207419_1001204&&18142_19330_1

5 / 13.1

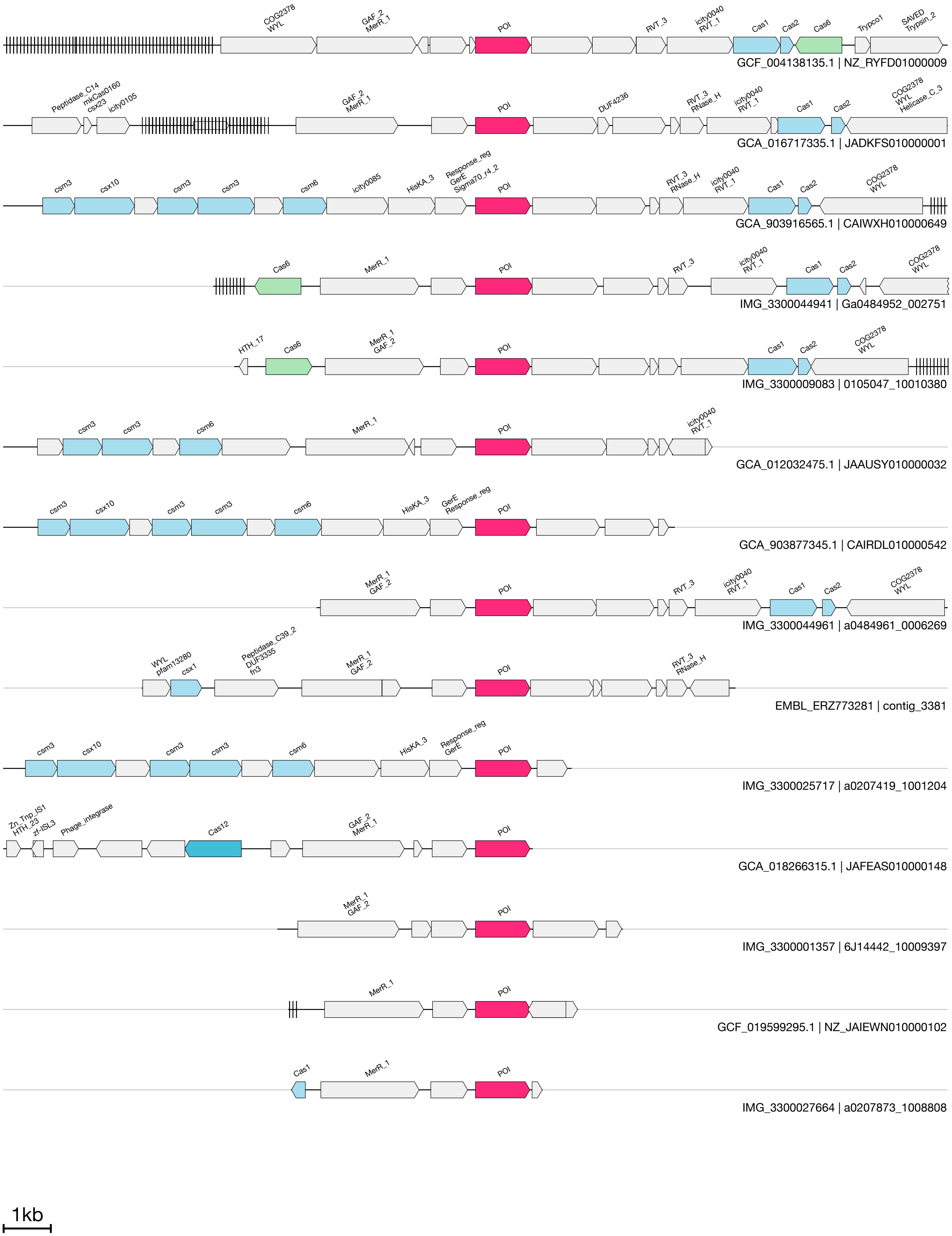

EG

UAS-131 (TIR2) 8 / 18.9

Auxiliary

IMG_3300026304&&a0209240_1021588&&337_1153_1

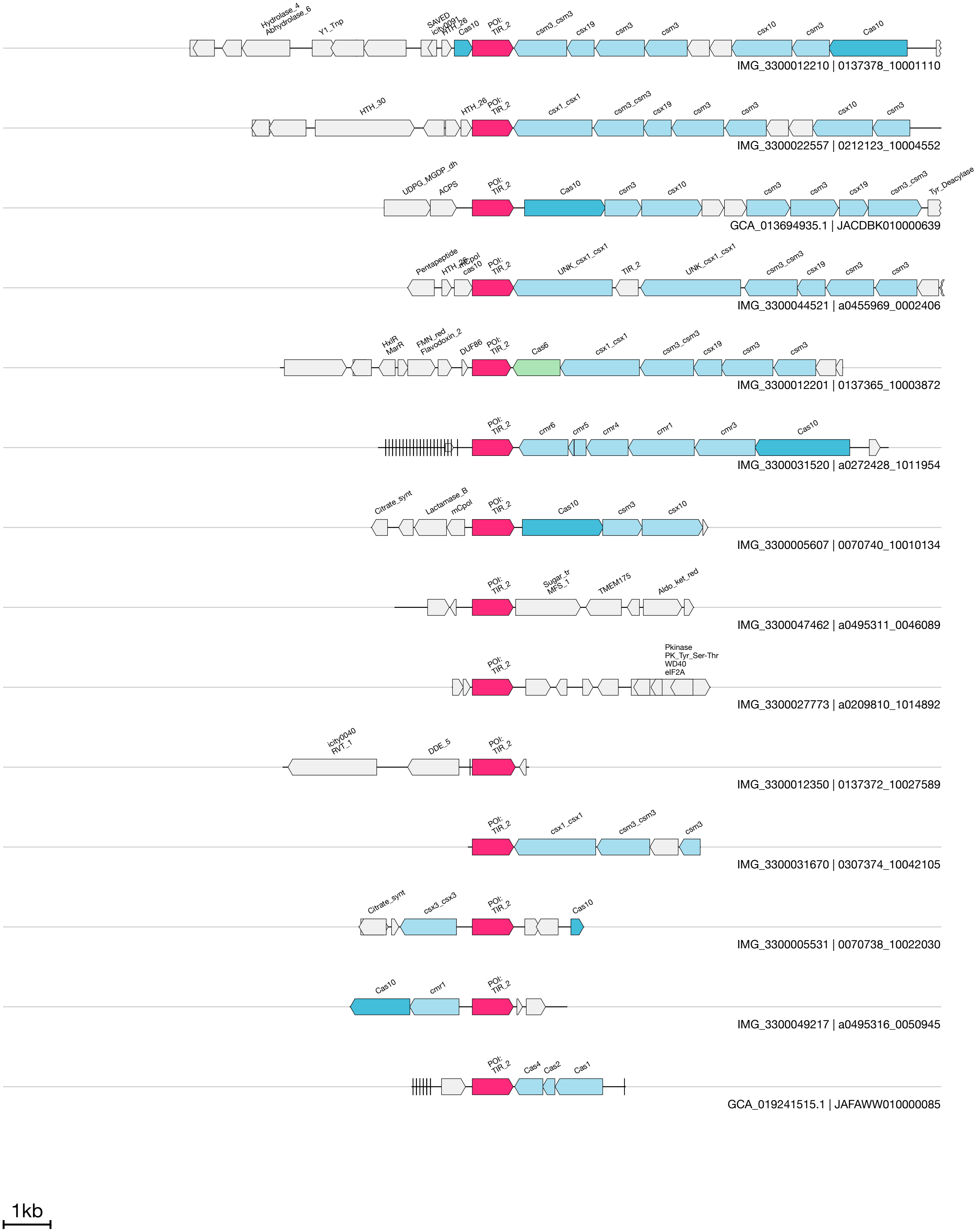

EH

UAS-132 **(Cas9 associated Y1)** 20 / 27.4
Auxiliary IMG_3300026516&&a0209573_1000325&&56350_57046_-1

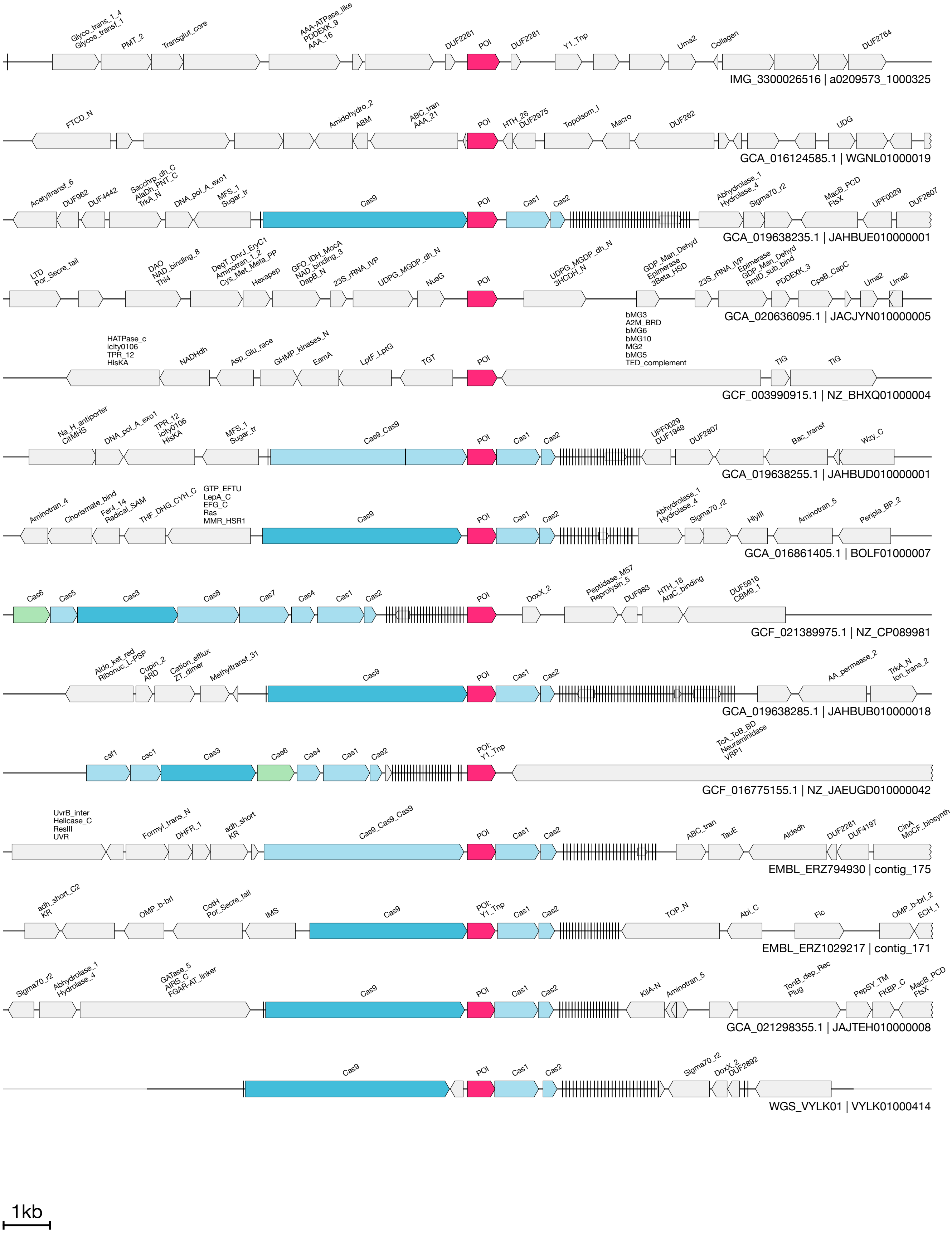

EI

UAS-133 **(TPR Rho RNA binding)** 5 / 6.4
Auxiliary IMG_3300026546&&0256913_10013832&&2_1481_1

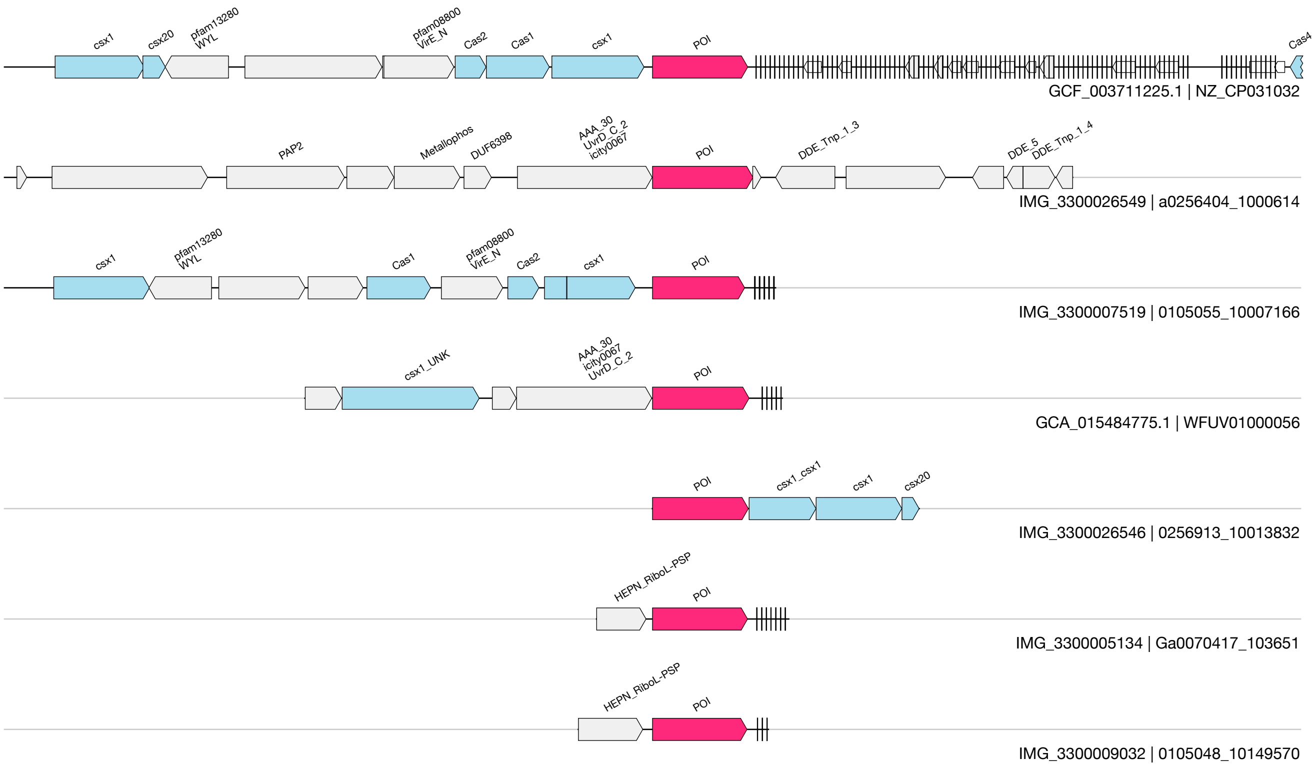

1kb

EJ

UAS-18 **(toprim)** 4 / 6.6

Auxiliary IMG_3300026546&&0256913_10022199&&462_1617_-1

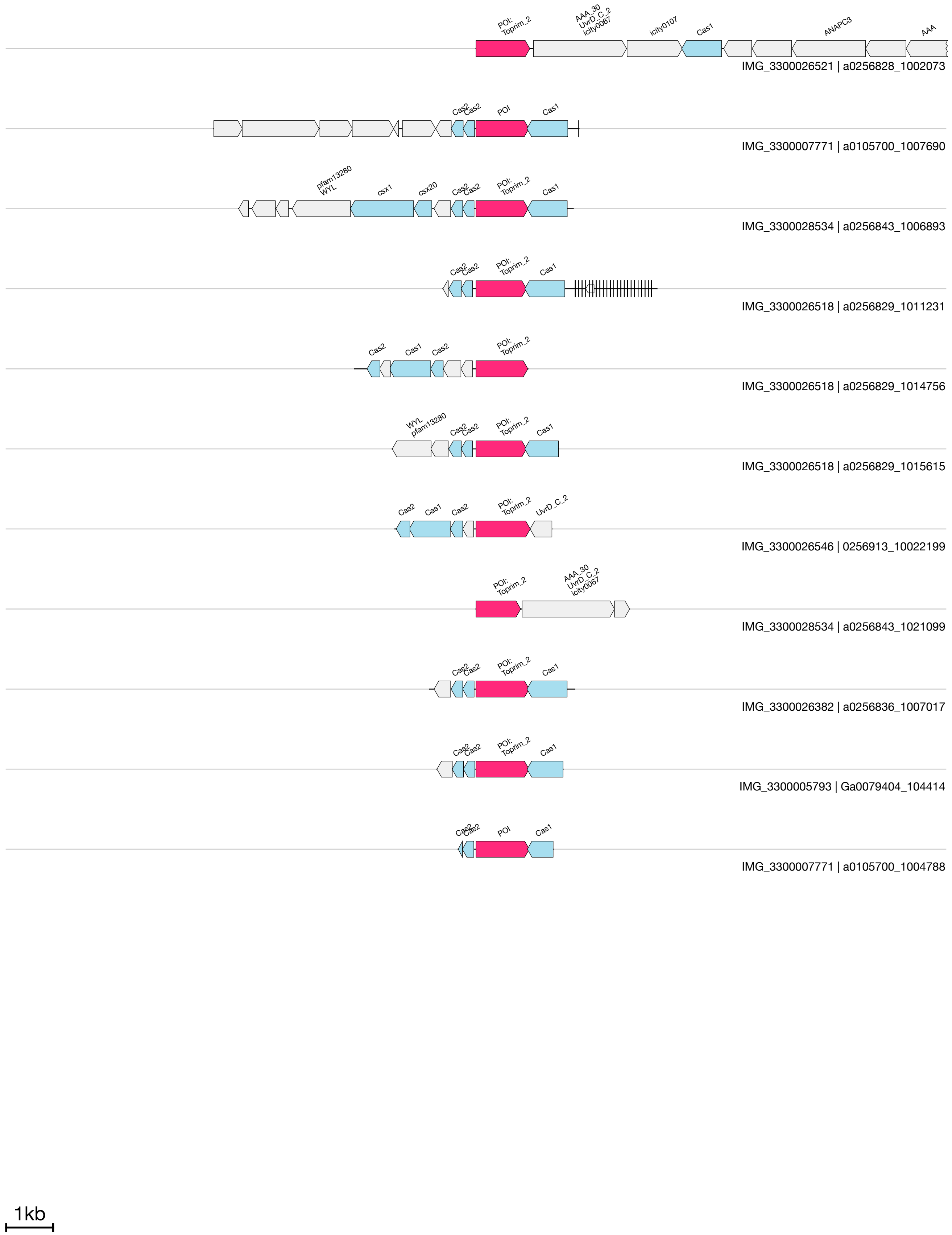

EK

UAS-134
Auxiliary

(WYL operonized with cascade)
IMG_3300027878&&0209181_10066870&&0_1041_1

15 / 29.8

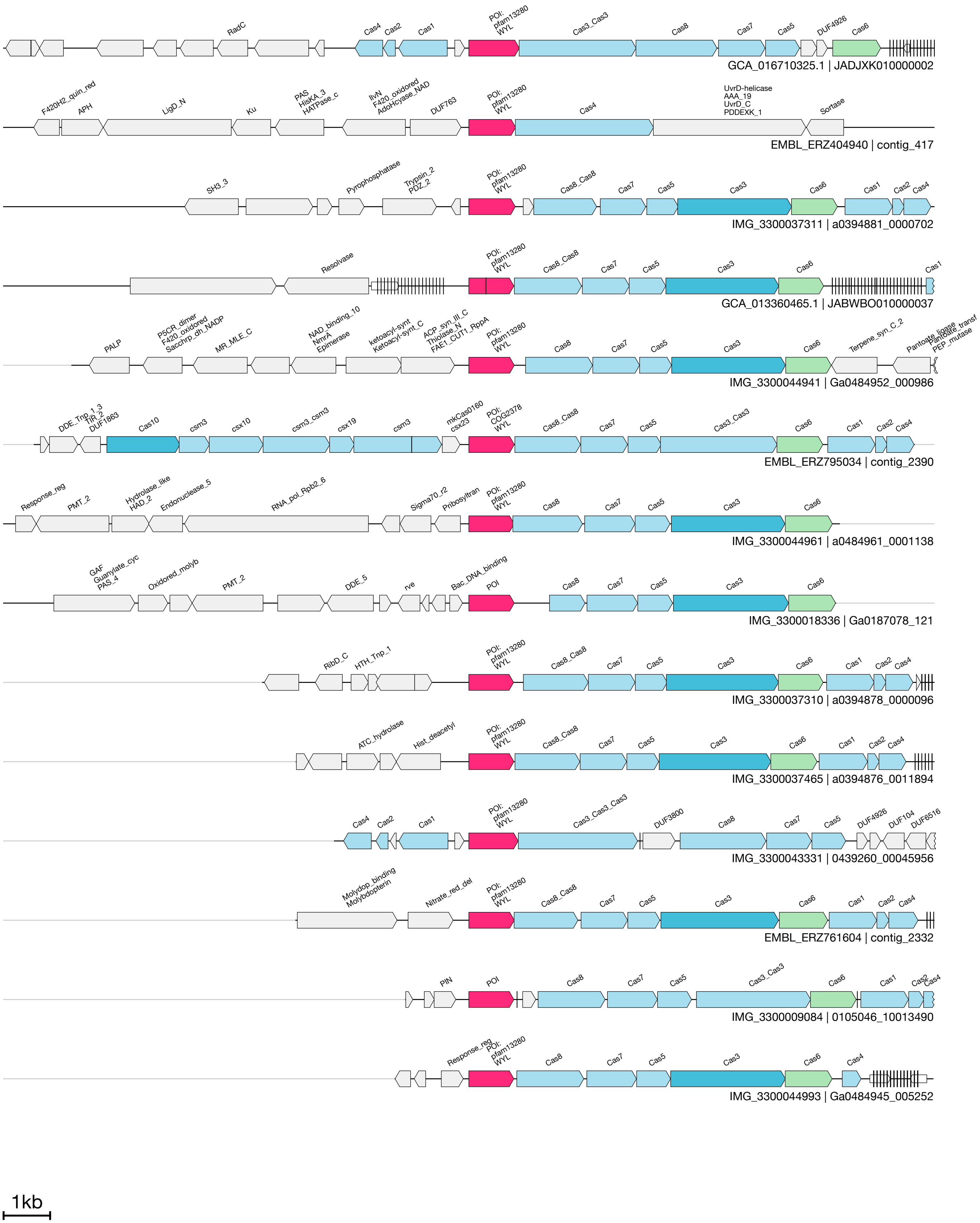

EL

UAS-135 **(ATPase_DUF4435 + Cas12)** 3 / 3.0
Auxiliary IMG_3300028048&&0256405_10015800&&2772_4671_-1

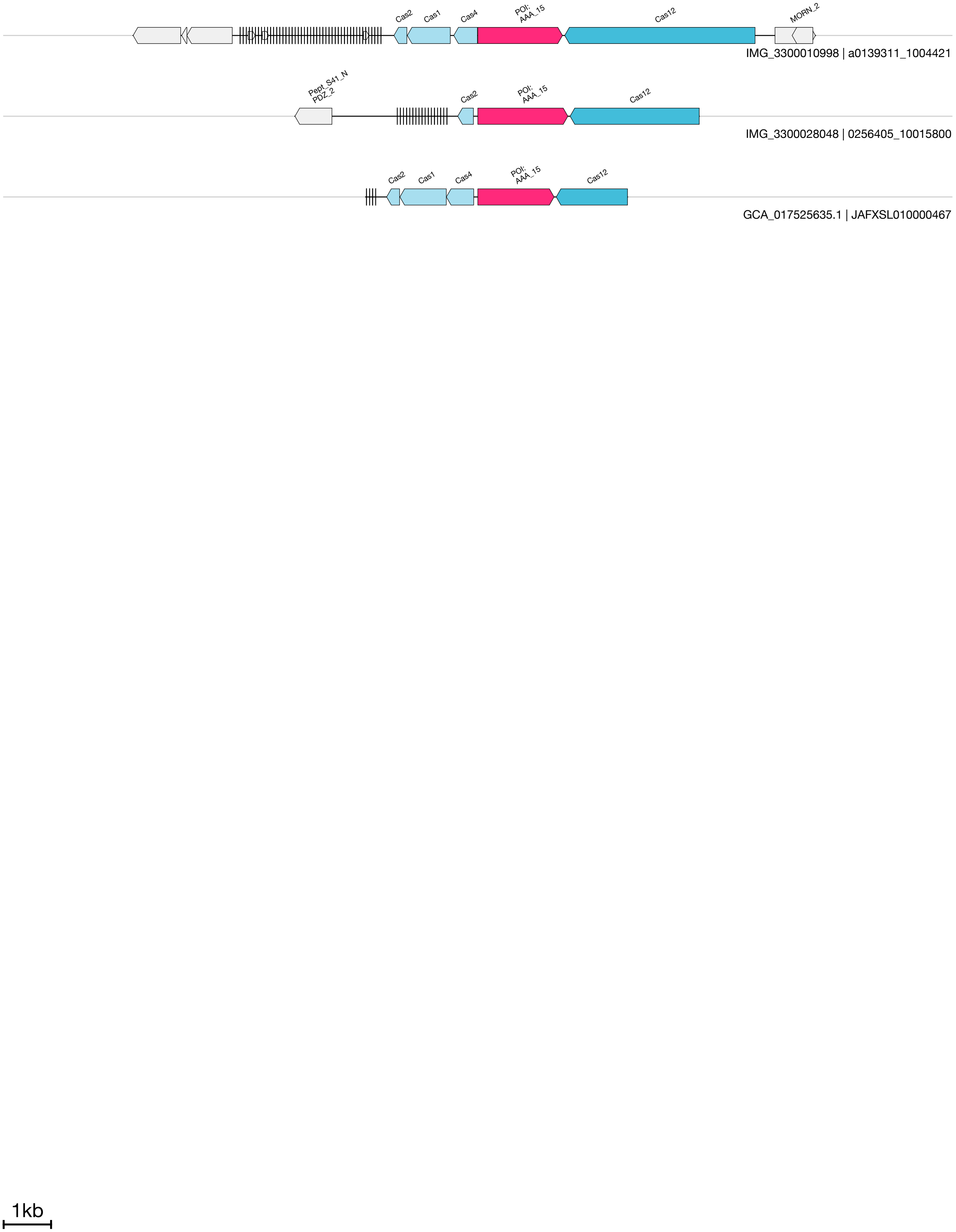

EM

UAS-136 **(DUF6155)** 4 / 6.7
Auxiliary IMG_3300028191&&a0265595_1000410&&37454_38072_1

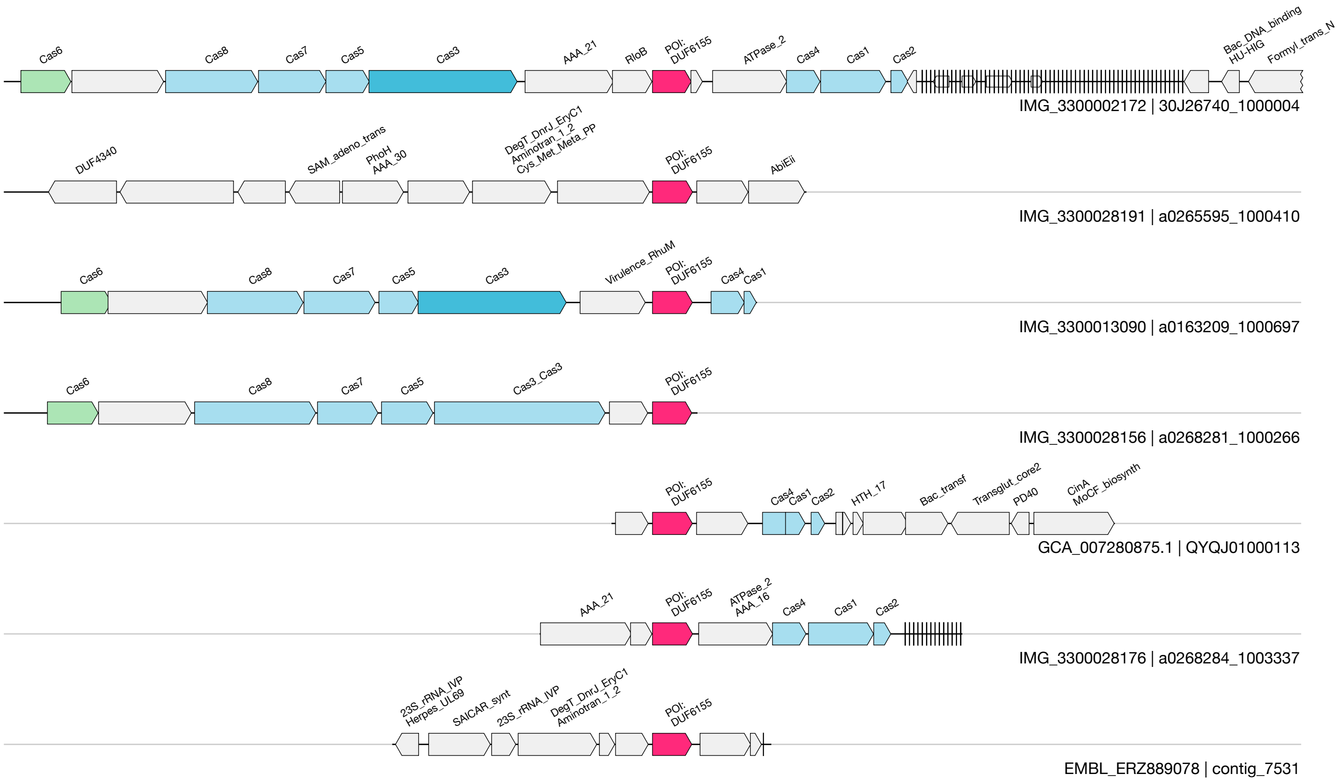

1kb

EN

UAS-137 **(Holliday Junction Resolvase)** 6 / 6.7
Auxiliary IMG_3300031520&&a0272428_1002147&&25604_25976_-1

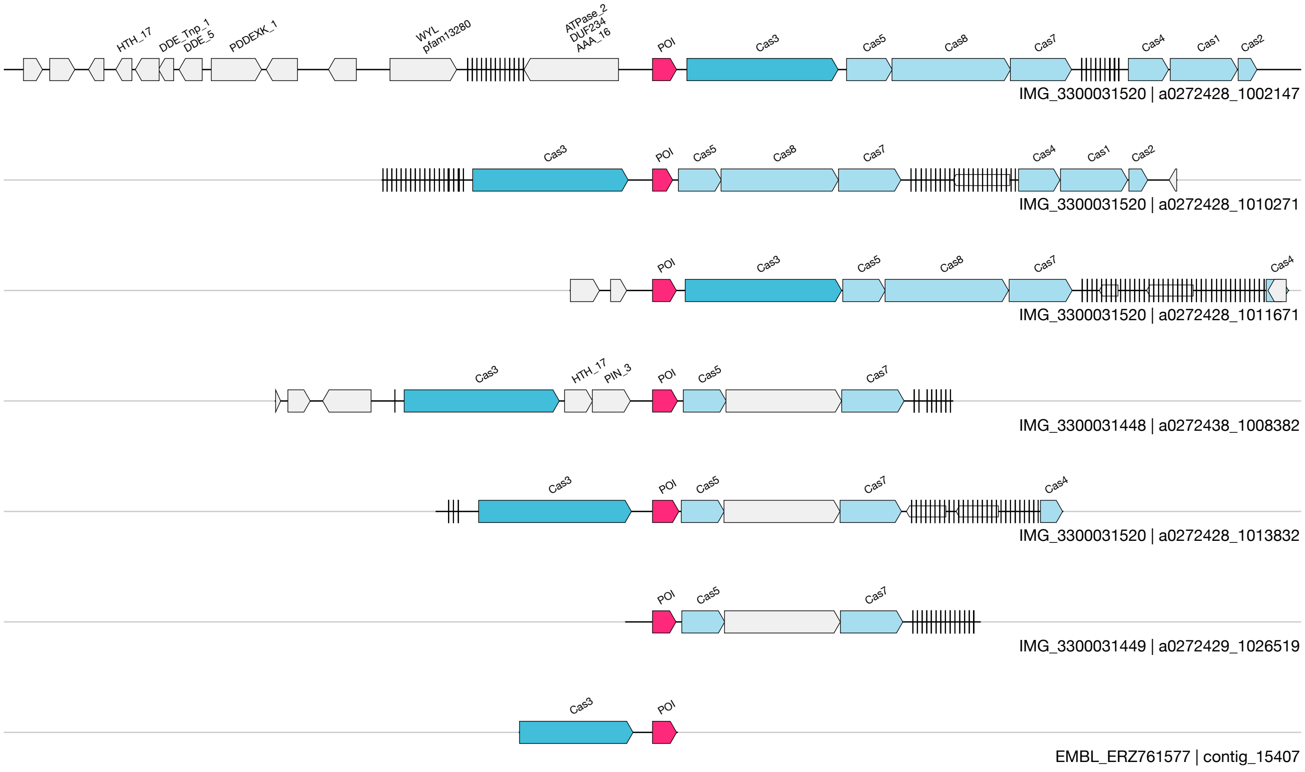

1kb

EO

UAS-138 **(TIR)** 5 / 11.2

Auxiliary IMG_3300031631&&a0307987_1000023&&70180_70972_-1

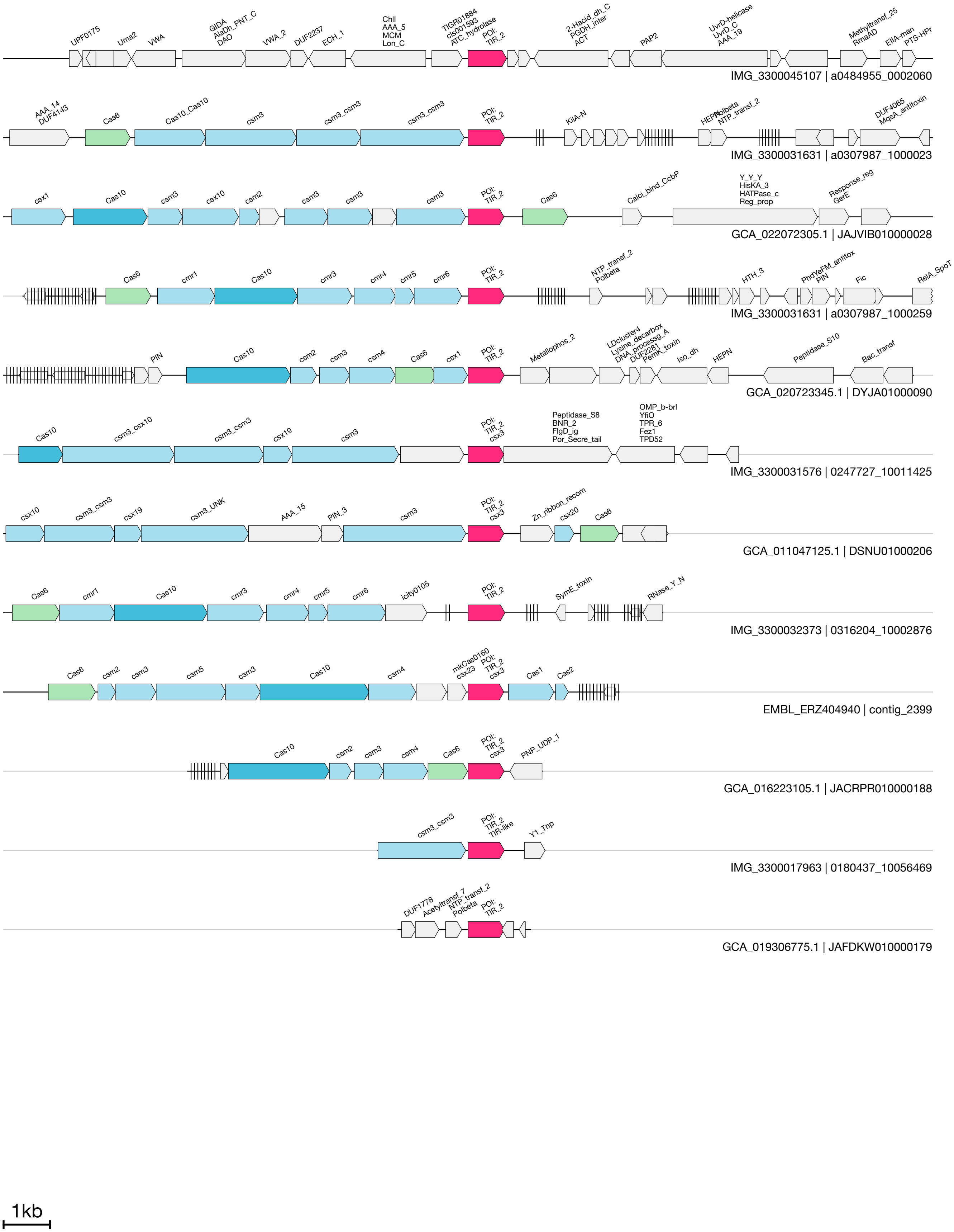

EP

UAS-139 **(metal system)** 3 / 4.3

Auxiliary IMG_3300031654&&a0315549_1044866&&183_2397_-1

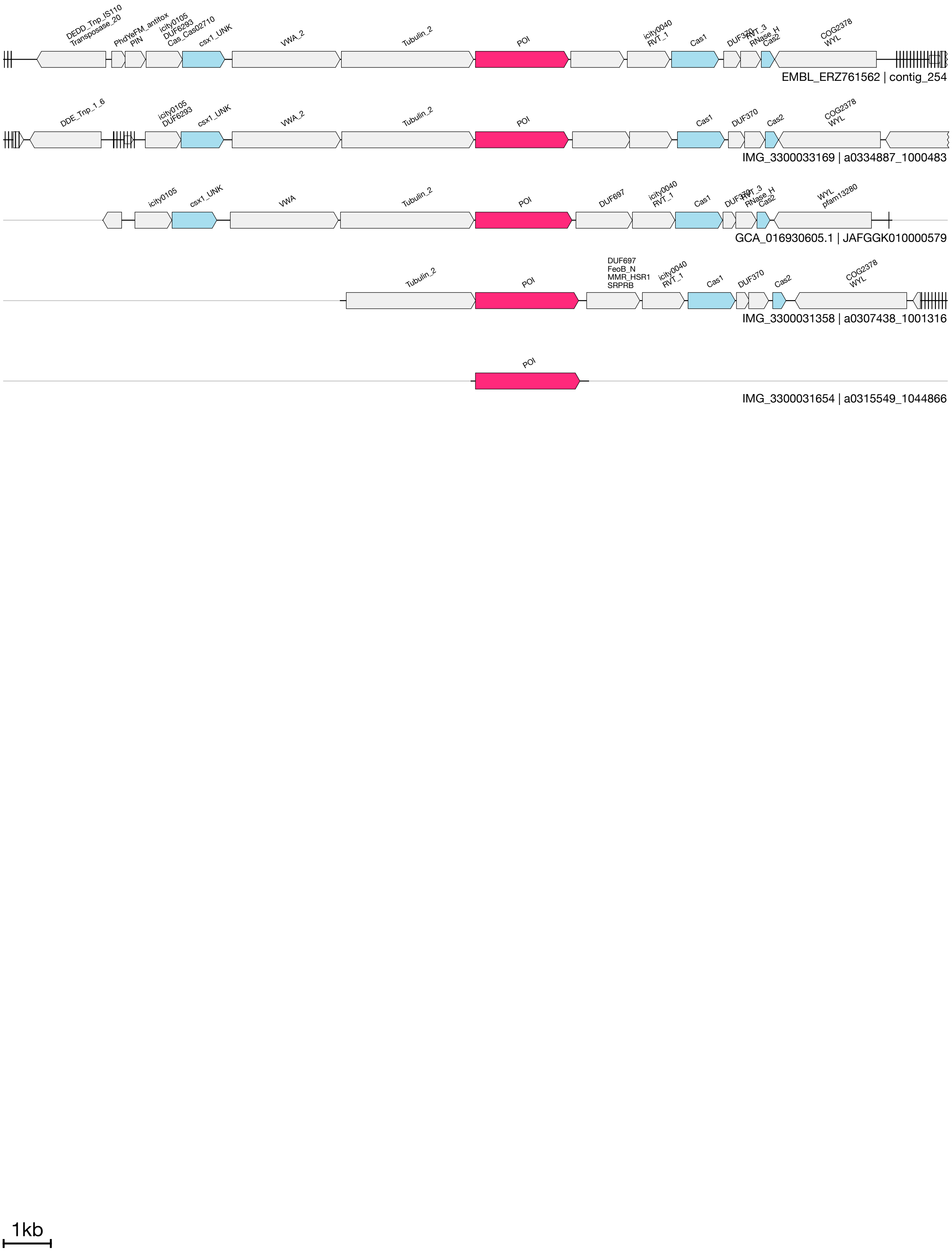

EQ

UAS-140 **(DUF5919)** 2 / 3.0

Auxiliary IMG_3300031670&&0307374_10003486&&16416_17127_1

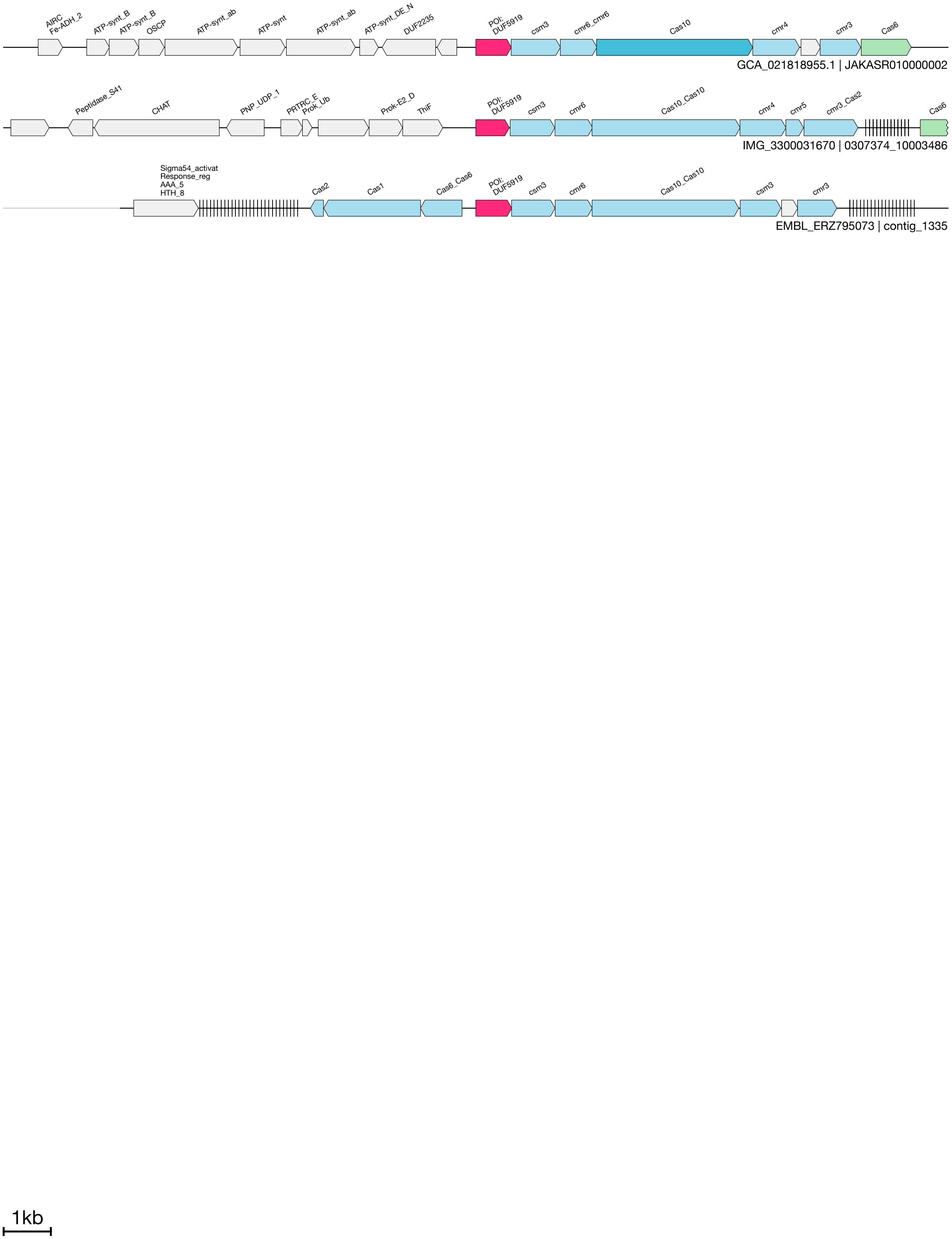

ER

UAS-141 | **(PRiA4 ORF3)** | 2 / 3.9
Auxiliary
IMG_3300031911&&0307412_10000093&&68229_68721_-1

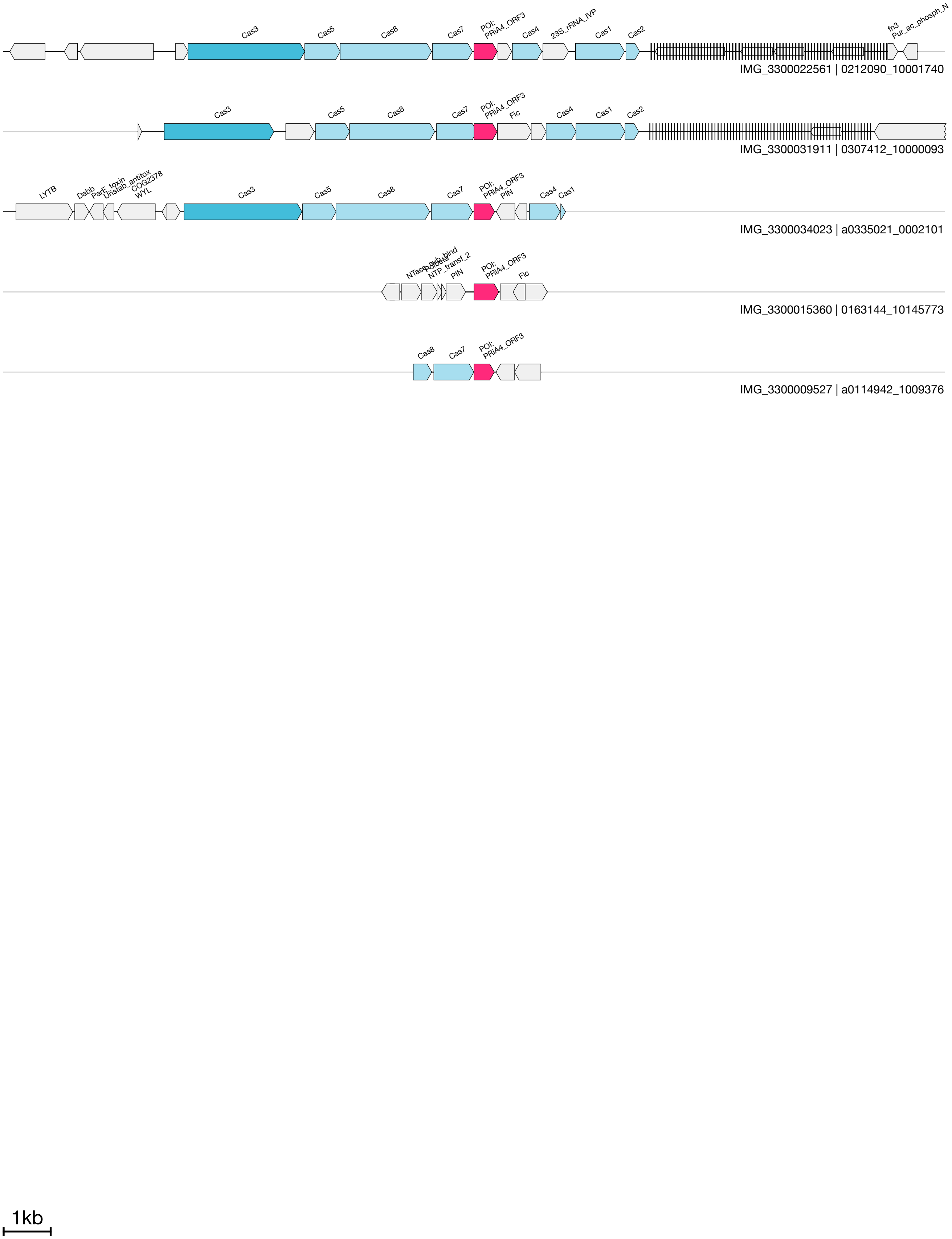

ES

UAS-142 **(TPR Protein Kinase)** 3 / 7.2

Auxiliary IMG_3300032272&&0316189_10000814&&17931_20814_1

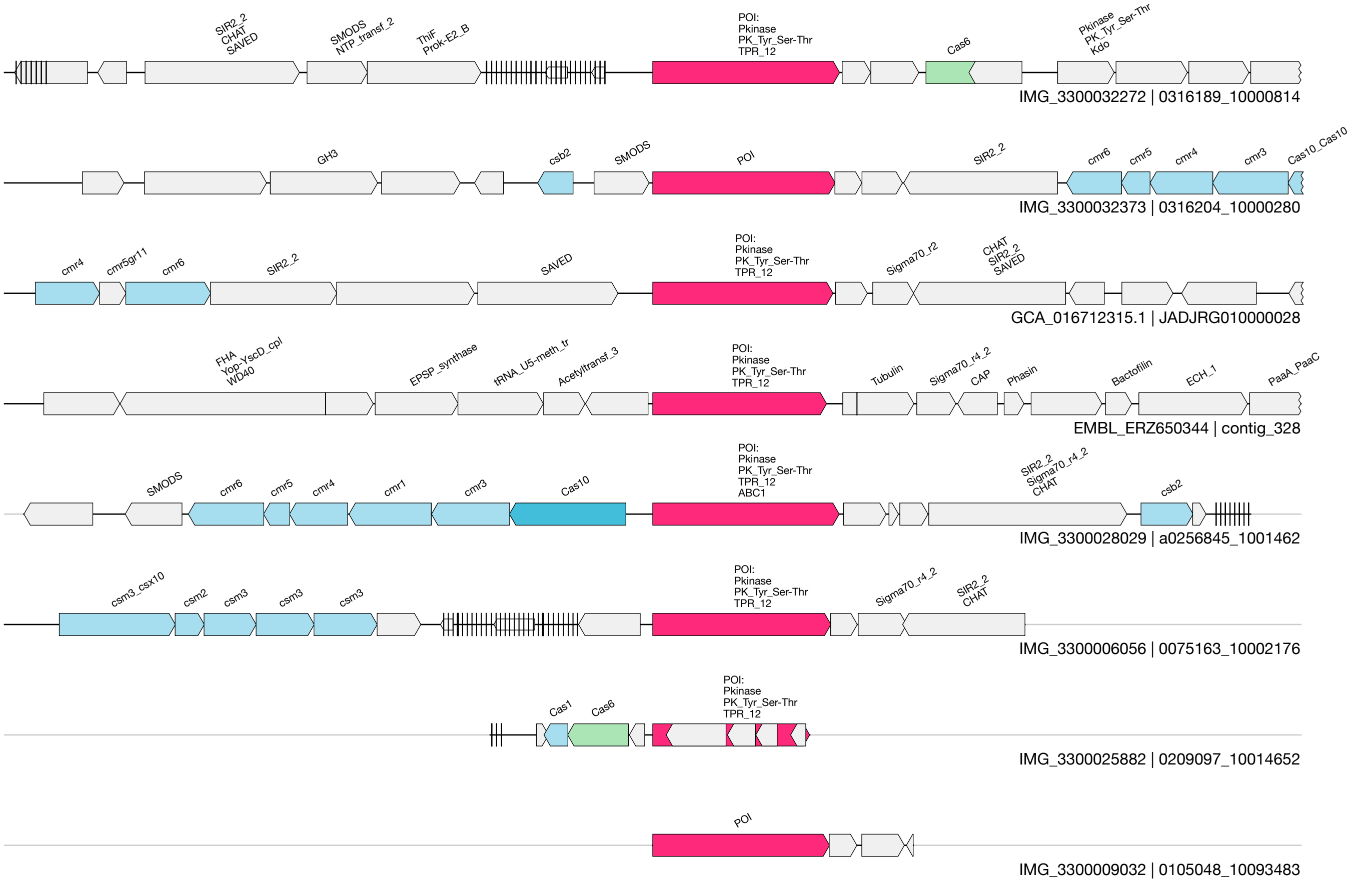

1kb

ET

UAS-143
Auxiliary

(EamA _ F420H2_quin_red)
IMG_3300033167&&a0334898_1003770&&3048_4593_-1

3 / 3.0

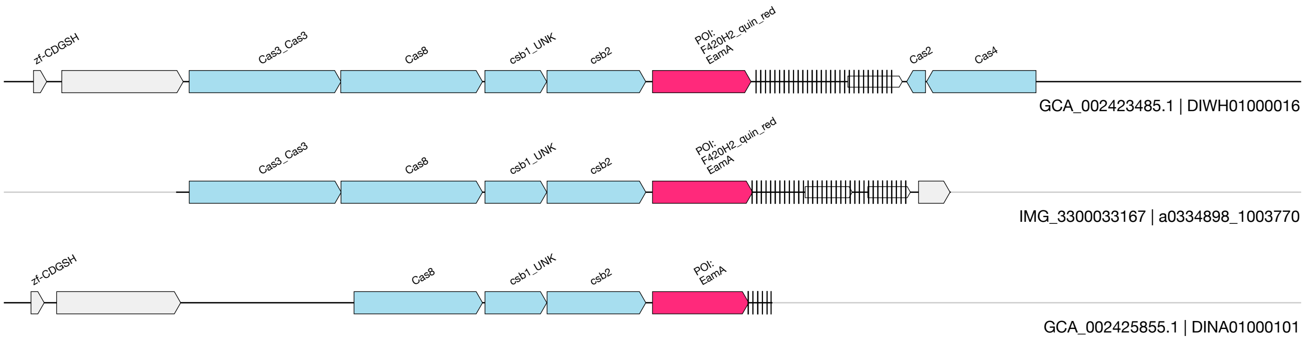

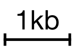

EU

UAS-144 **(TPR)** 24 / 48.9
Auxiliary IMG_3300033463&&0310690_10163143&&217_2491_-1

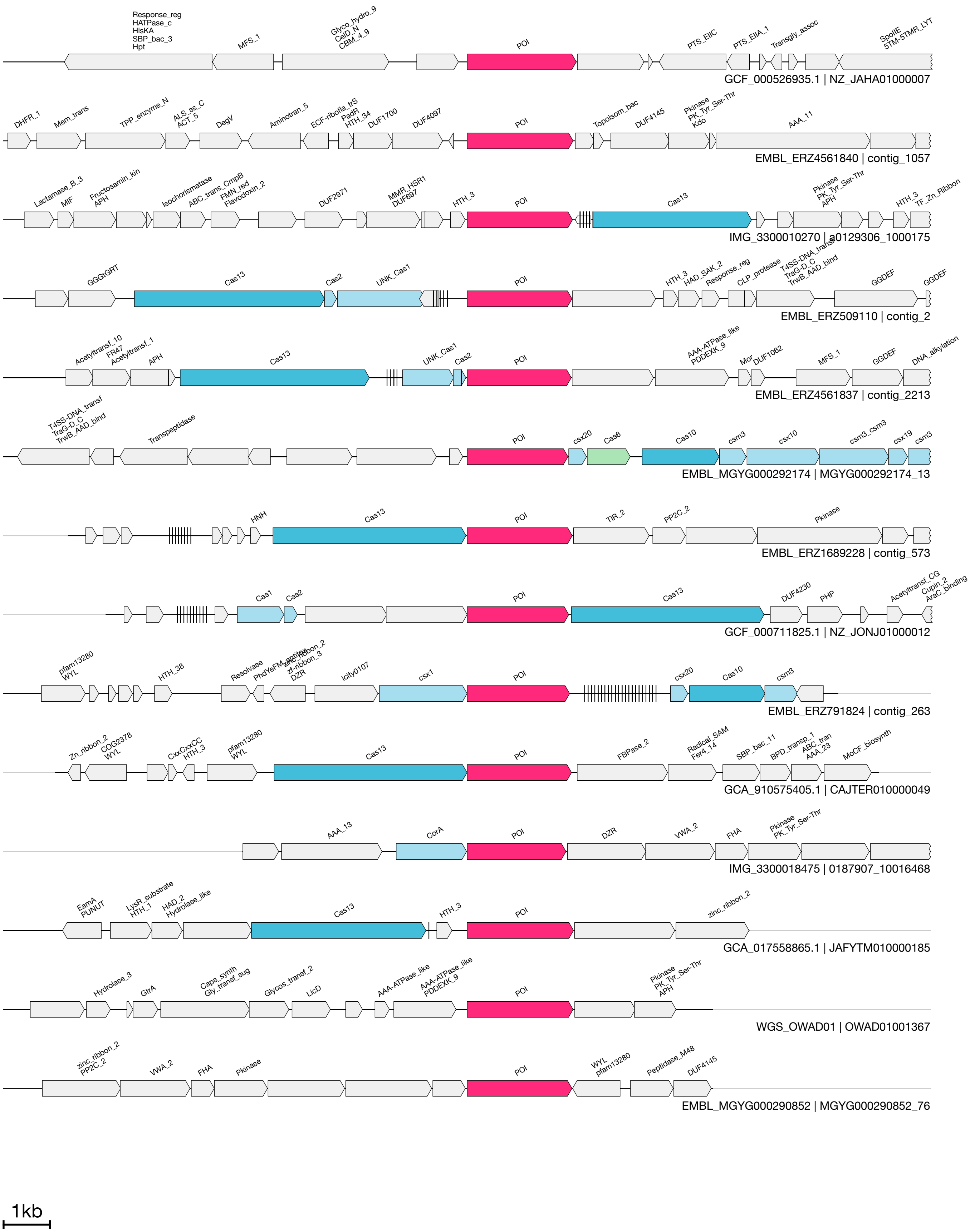

EV

UAS-145 **(transmembrane NARF)** 5 / 10.7

Auxiliary IMG_3300034107&&a0335037_0001347&&11328_12051_-1

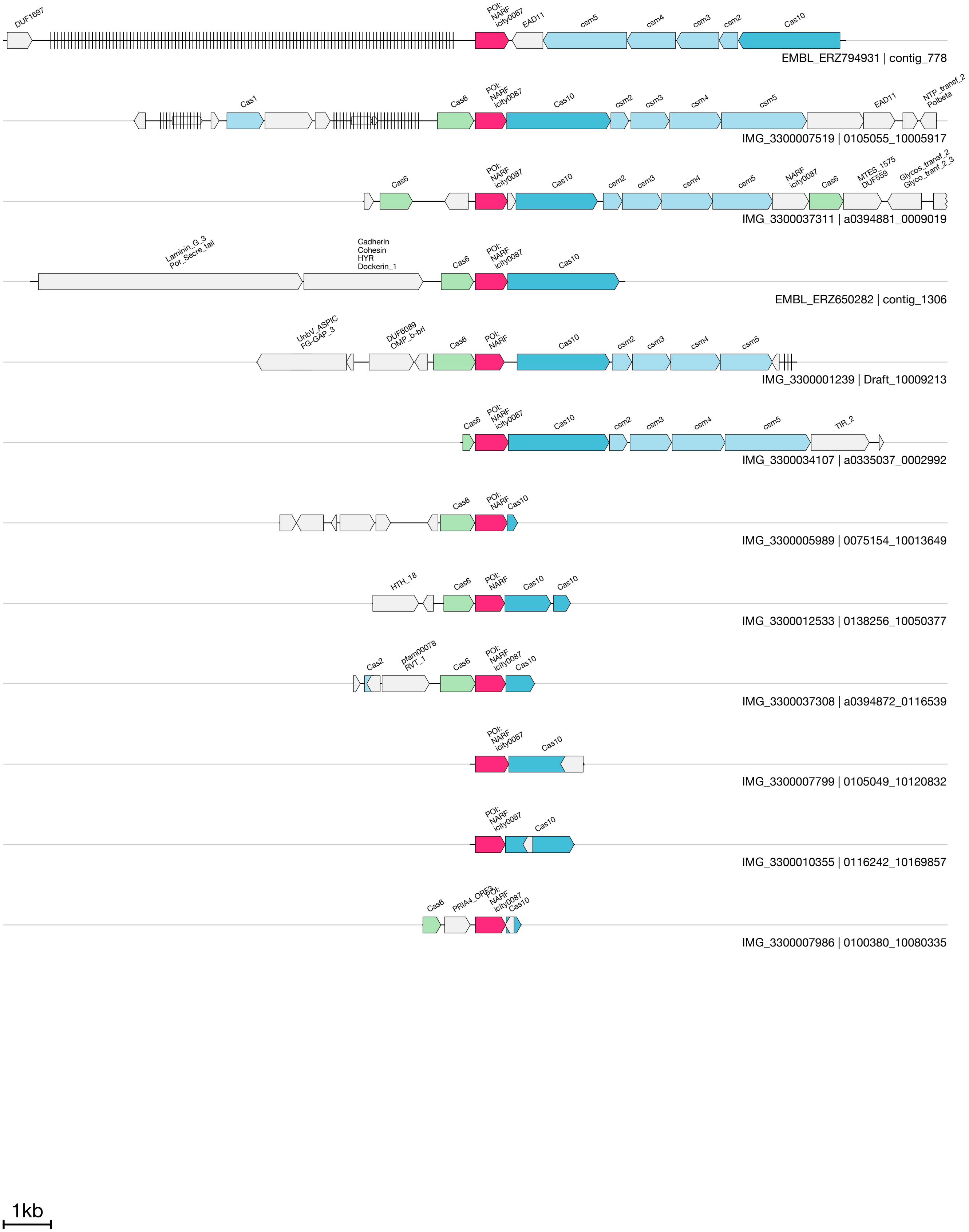

EW

UAS-25 **(NACHT_PDDEXK + cascade)** 8 / 12.8

Auxiliary IMG_3300035698&&Ga0374944_616918&&9967_11707_-1

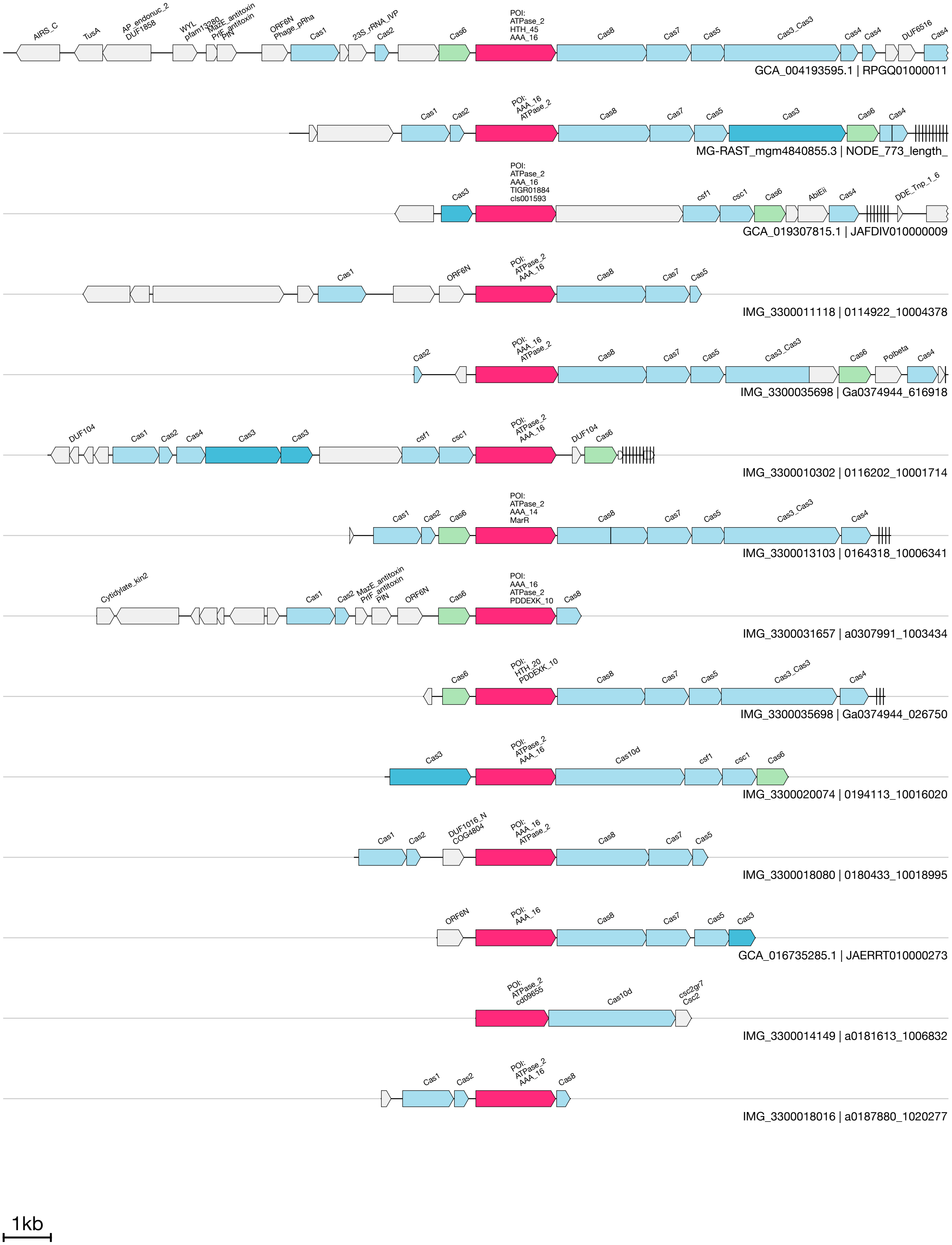

EX

UAS-146 **(EAD11)** 5 / 8.2

Auxiliary IMG_3300037311&&a0394881_0008304&&11617_12277_1

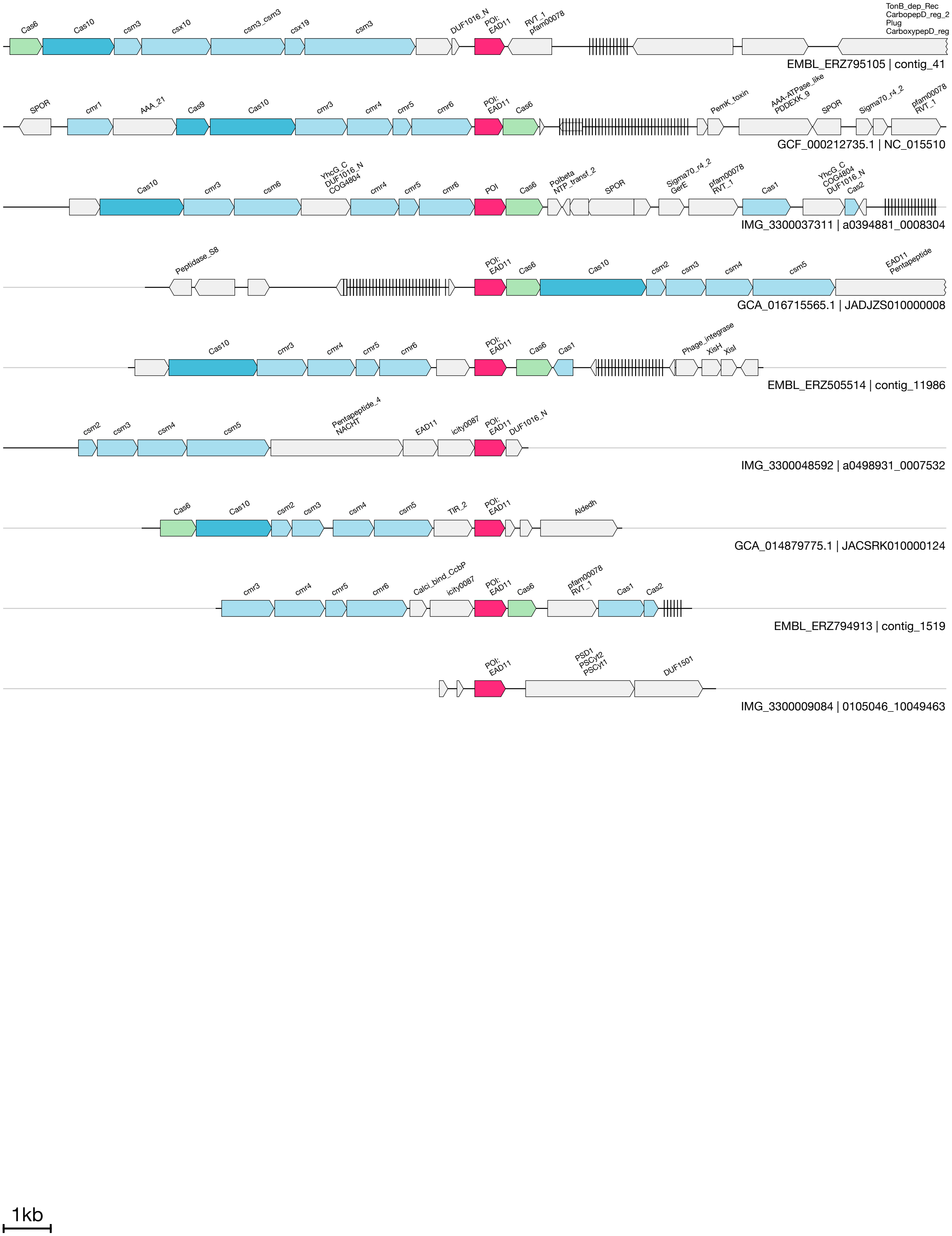

EY

UAS-147 **(WYL I-D)** 36 / 66.5

Auxiliary IMG_3300037509&&a0394184_0000482&&19069_20062_1

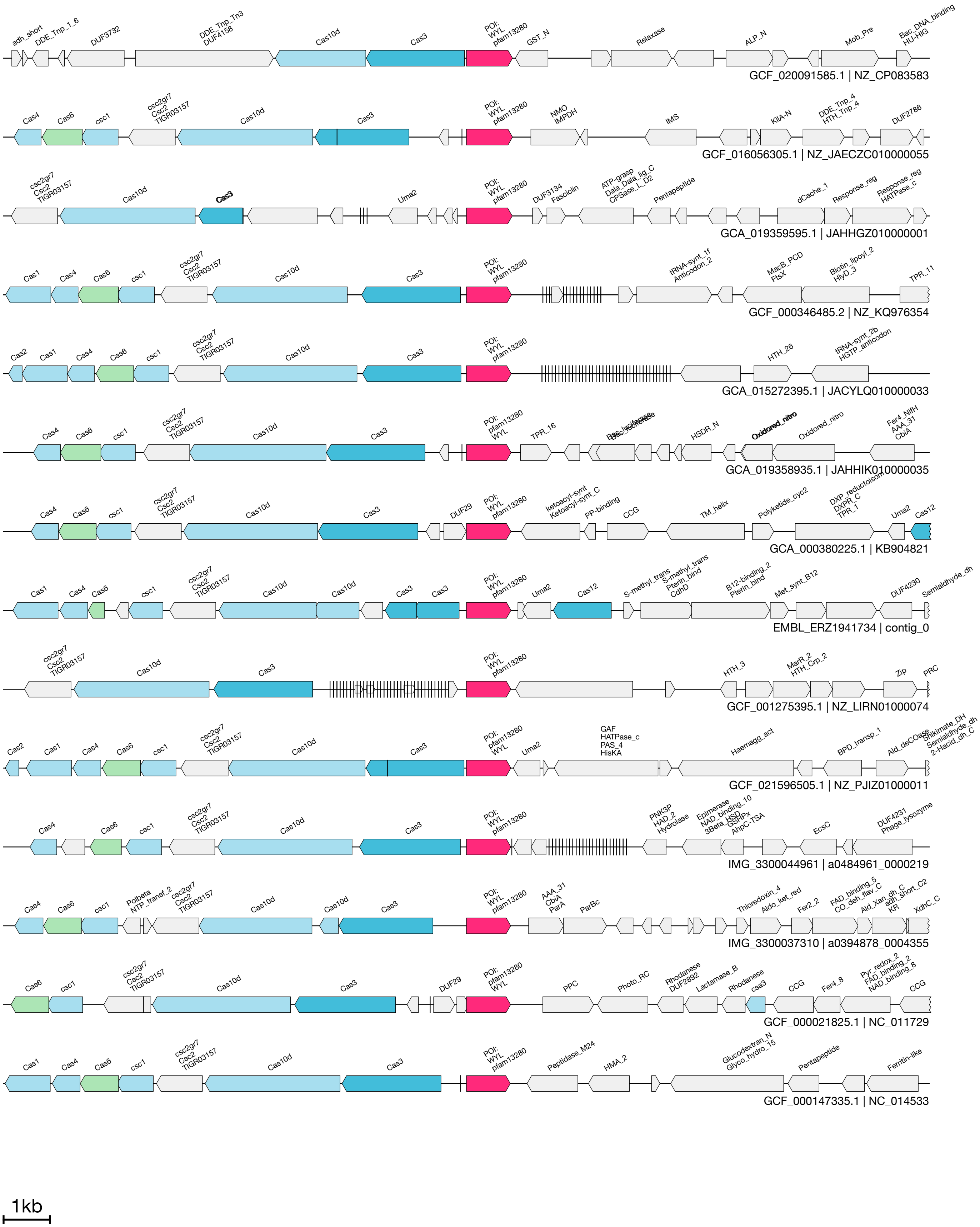

EZ

UAS-27
Auxiliary

(methyltransferase + cascade)
IMG_3300041417&&a0439242_0072051&&1423_2242_1

2 / 3.2

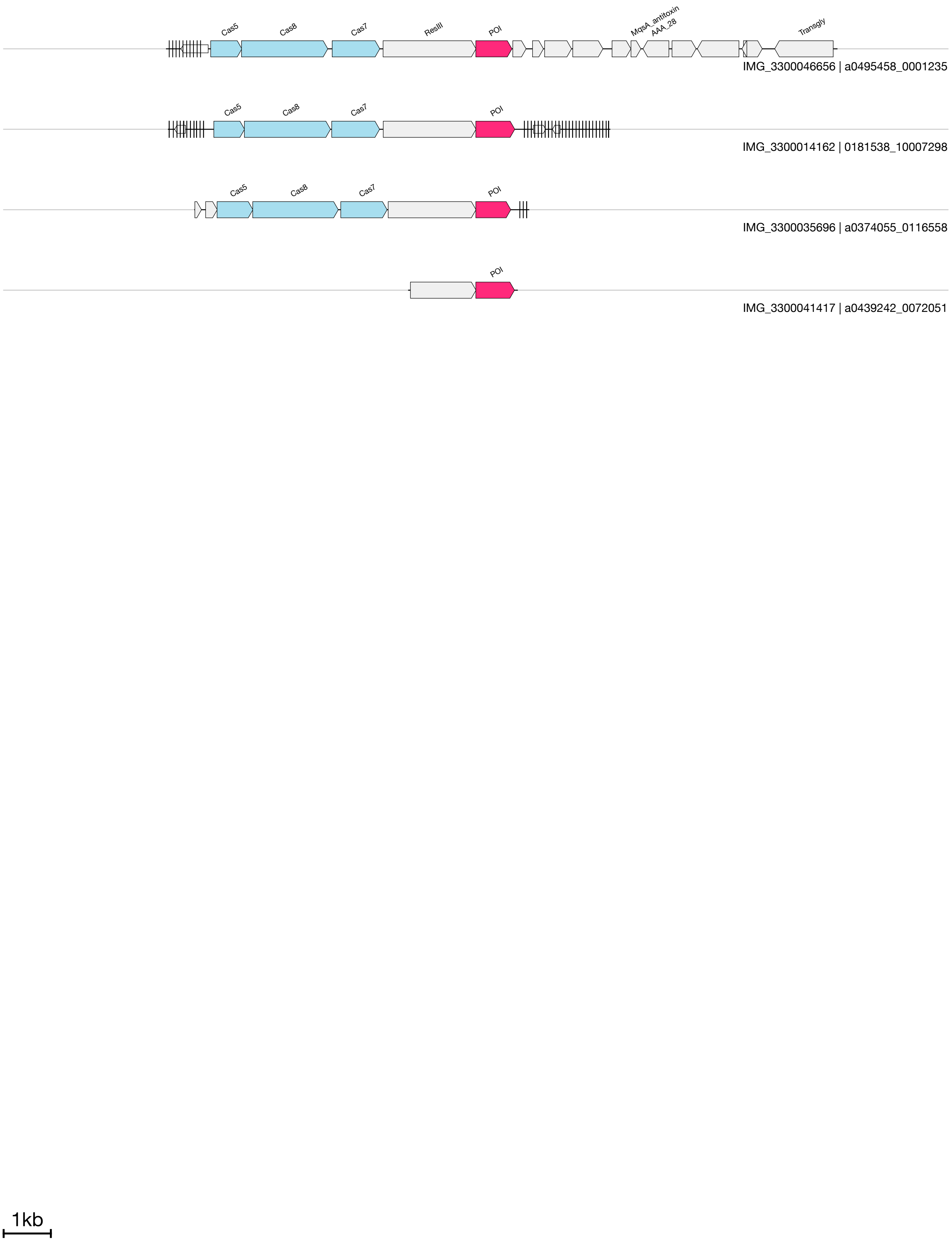

FA

UAS-148 **(mega Cas8-like)** 37 / 51.3

Auxiliary IMG_3300042340&&a0453917_0000106&&32810_35291_-1

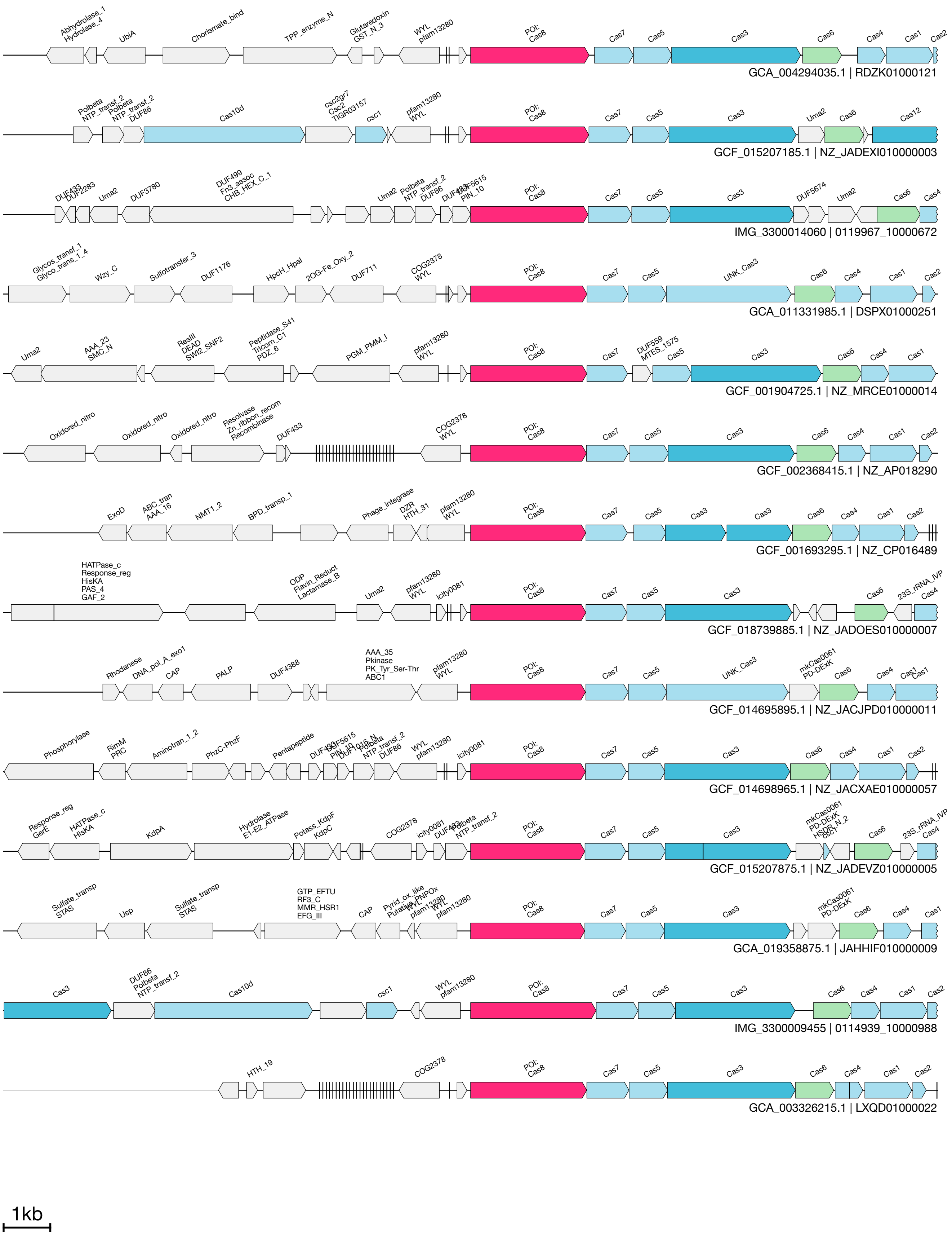

FB

UAS-149
Auxiliary

(vWA + Tubulin)
IMG_3300043390&&Ga0454692_000076&&4479_7614_1

N/A

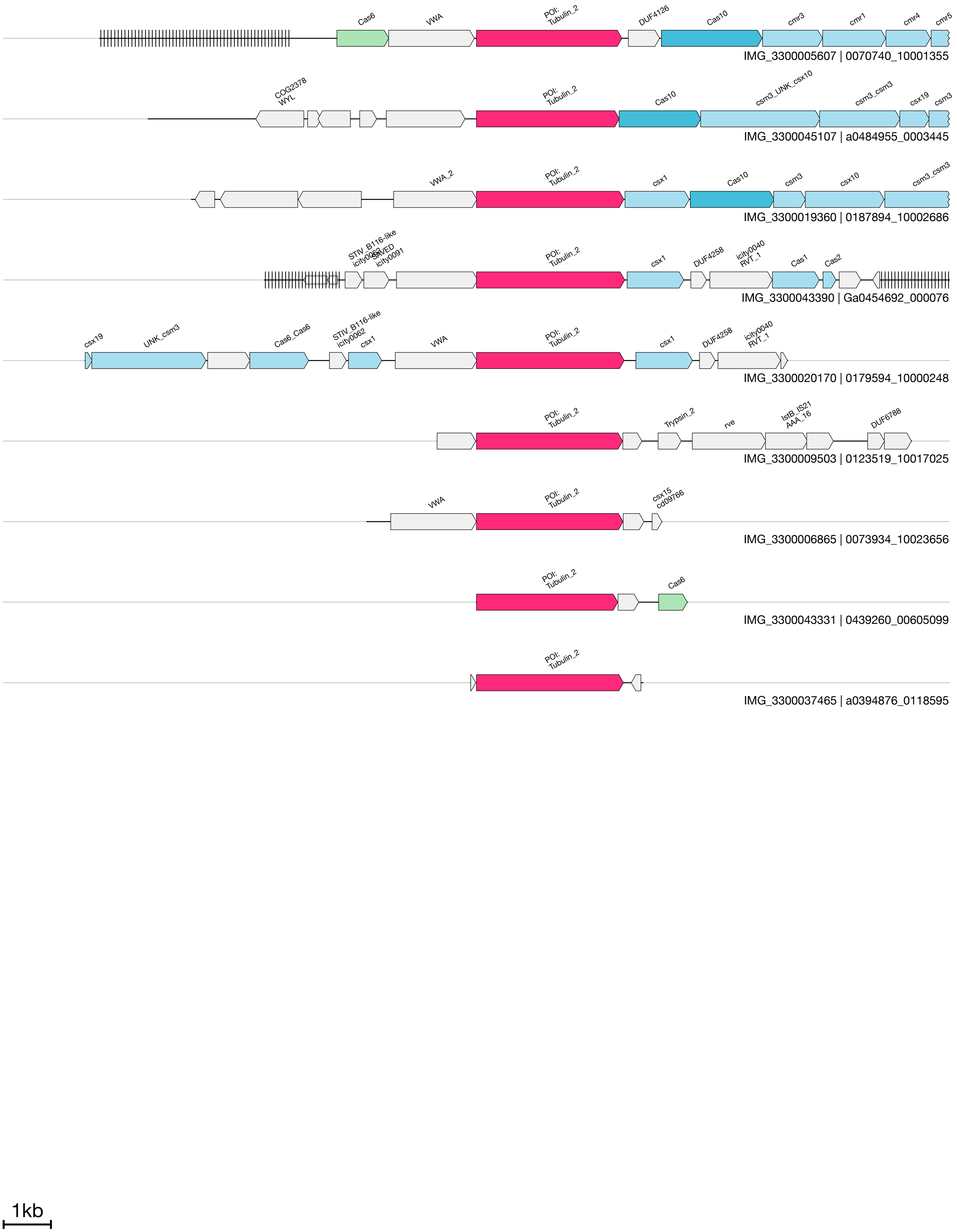

FC

UAS-150 **(APH_EcKinase)** 3 / 3.0

Auxiliary IMG_3300044730&&a0453757_0004423&&6344_8234_-1

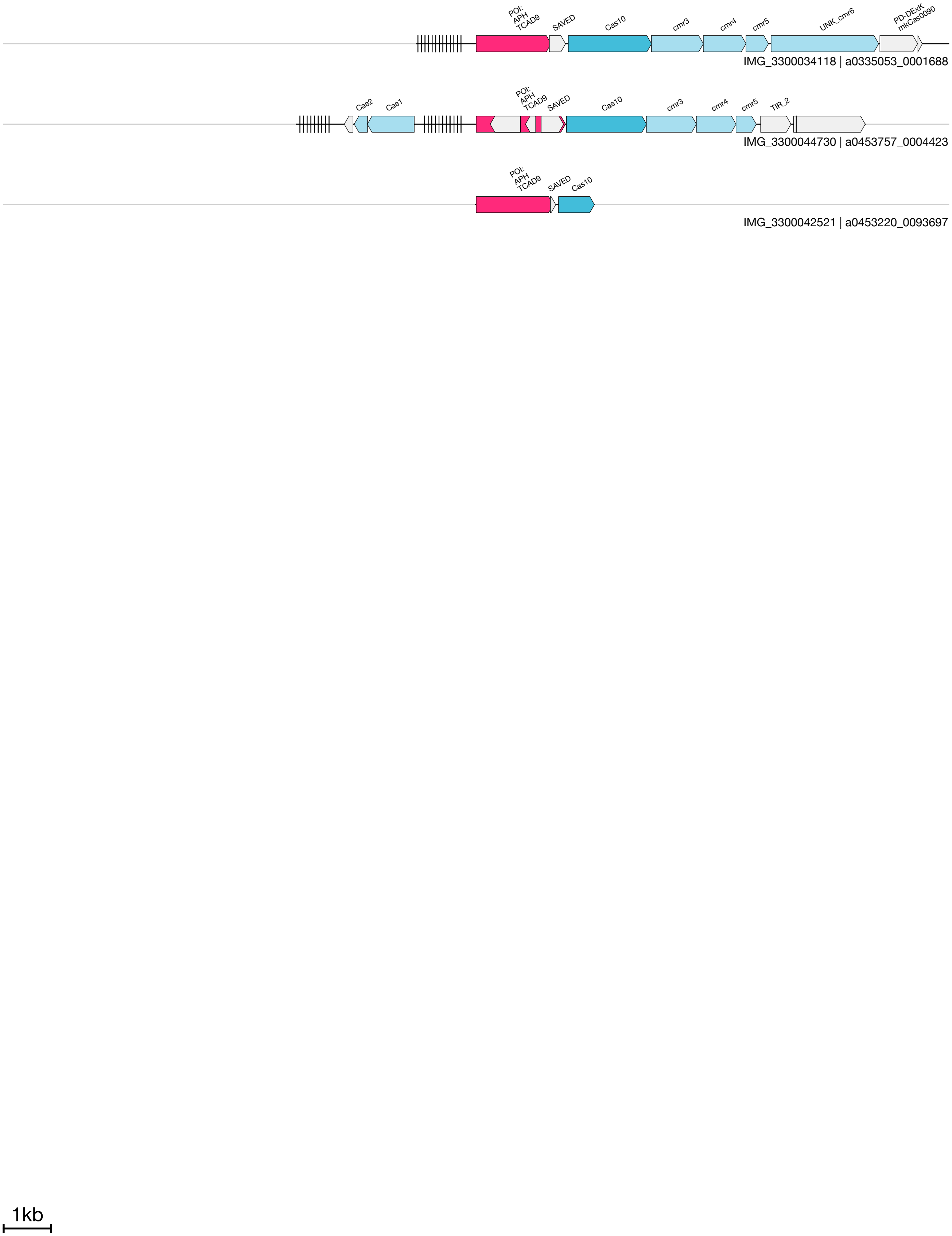

FD

UAS-151 **(Cas8_Fic fusion)** 2 / 5.0
Auxiliary IMG_3300045107&&a0484955_0013512&&3038_6074_-1

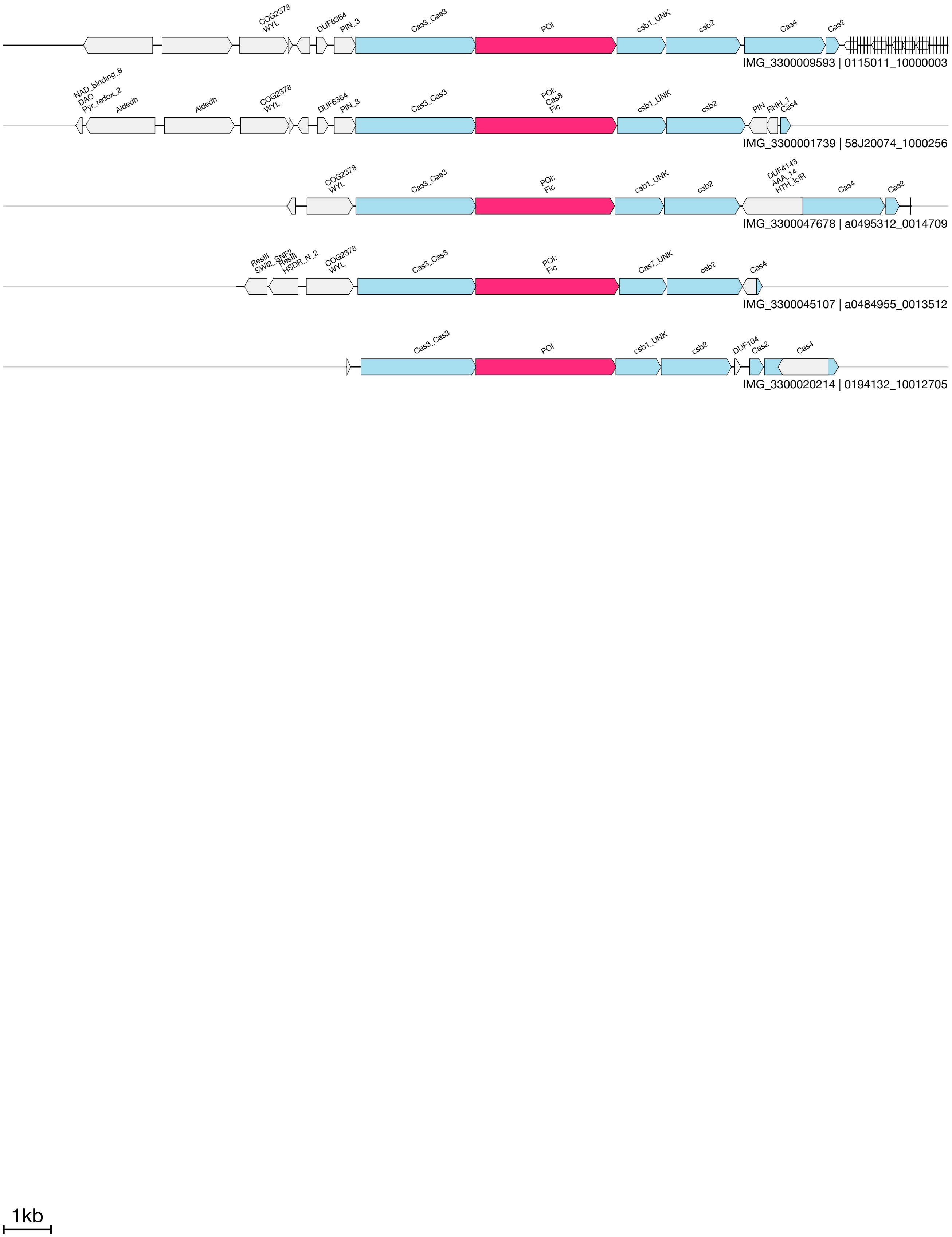

FE

UAS-152 **(RNaseT)** 4 / 7.4

Auxiliary

IMG_3300046534&&a0491194_0030312&&2144_2783_-1

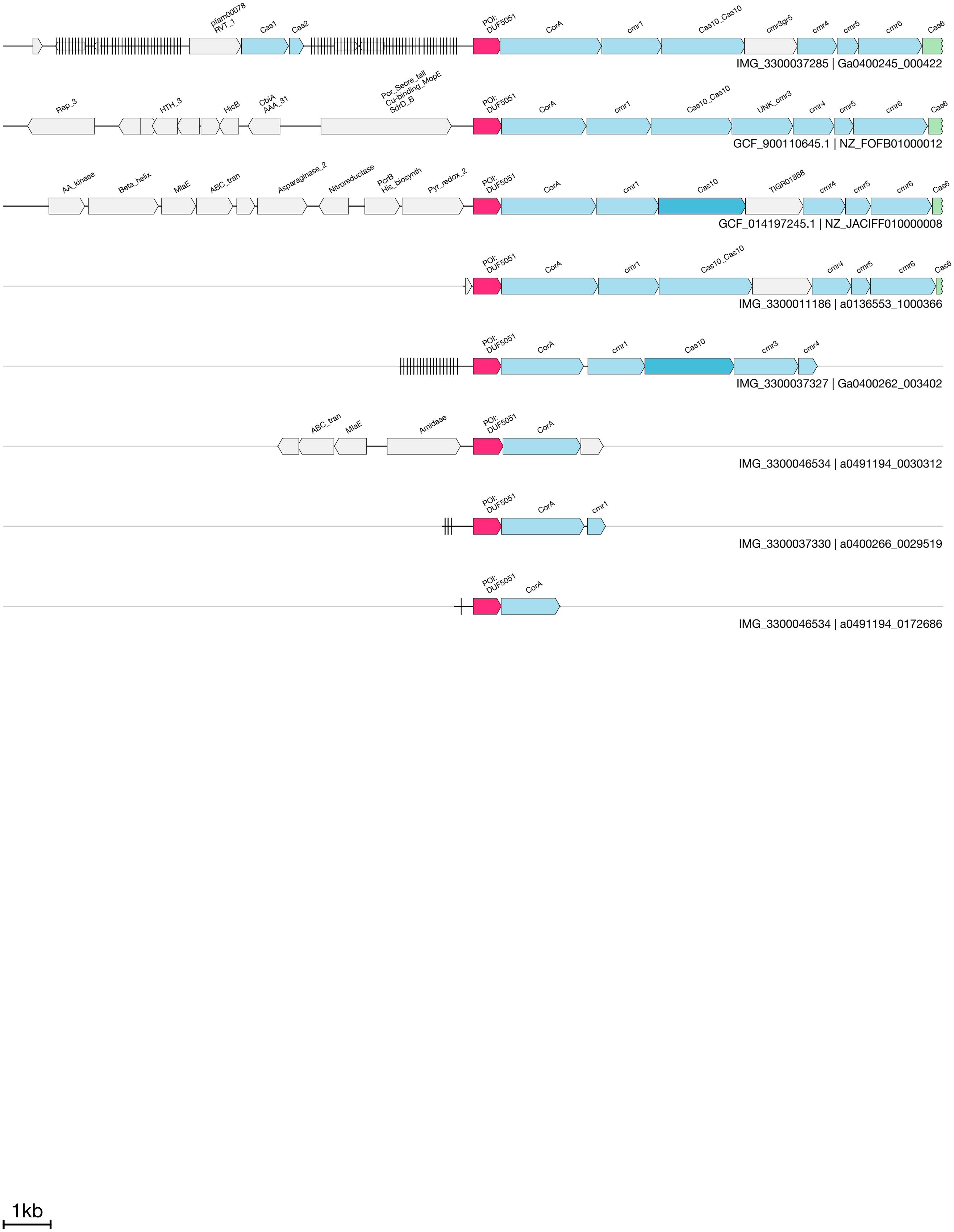

FF

UAS-153 **(SNF2 instead of Cas3)** 7 / 9.9

Auxiliary IMG_3300047317&&a0495604_0021803&&2293_4057_-1

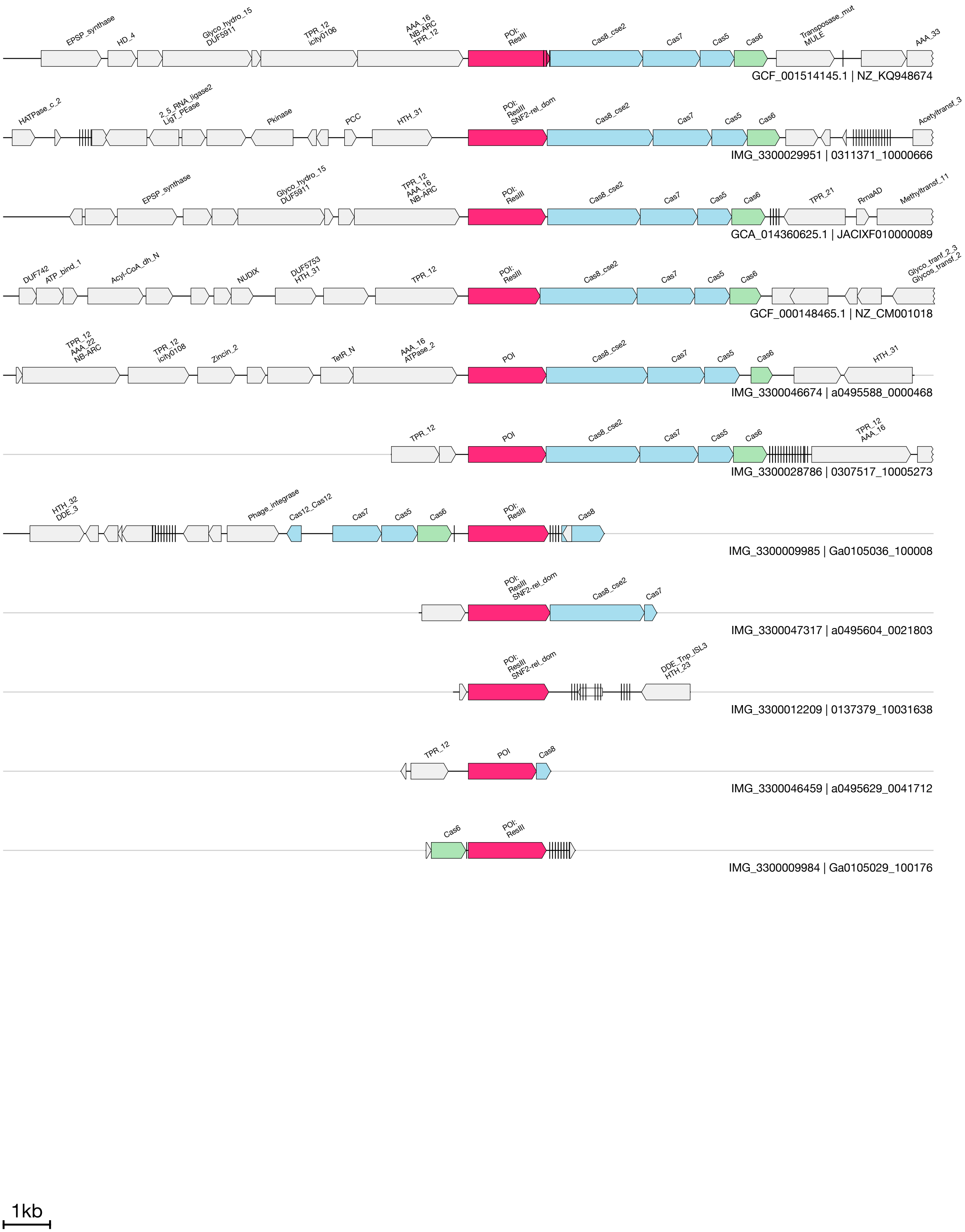

FG

UAS-154 **(HIRAN Cas9, one case ATPase)** 28 / 44.9
Auxiliary IMG_3300048583&&a0498911_0096679&&1007_1916_1

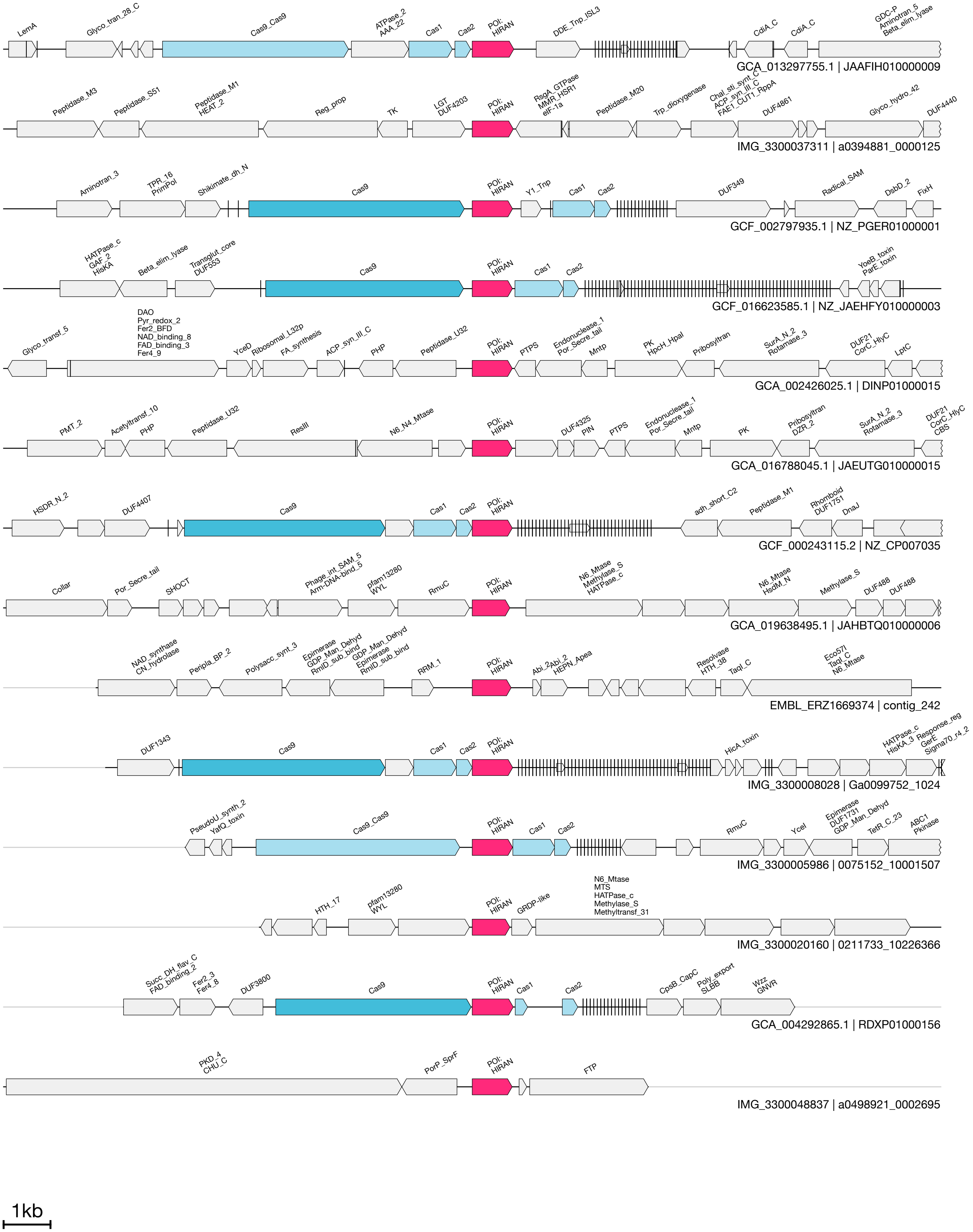

FH

UAS-20
Auxiliary

(Cas12 + bzip)
IMG_3300048589&&a0498927_0037426&&1005_1464_1

7 / 7.4

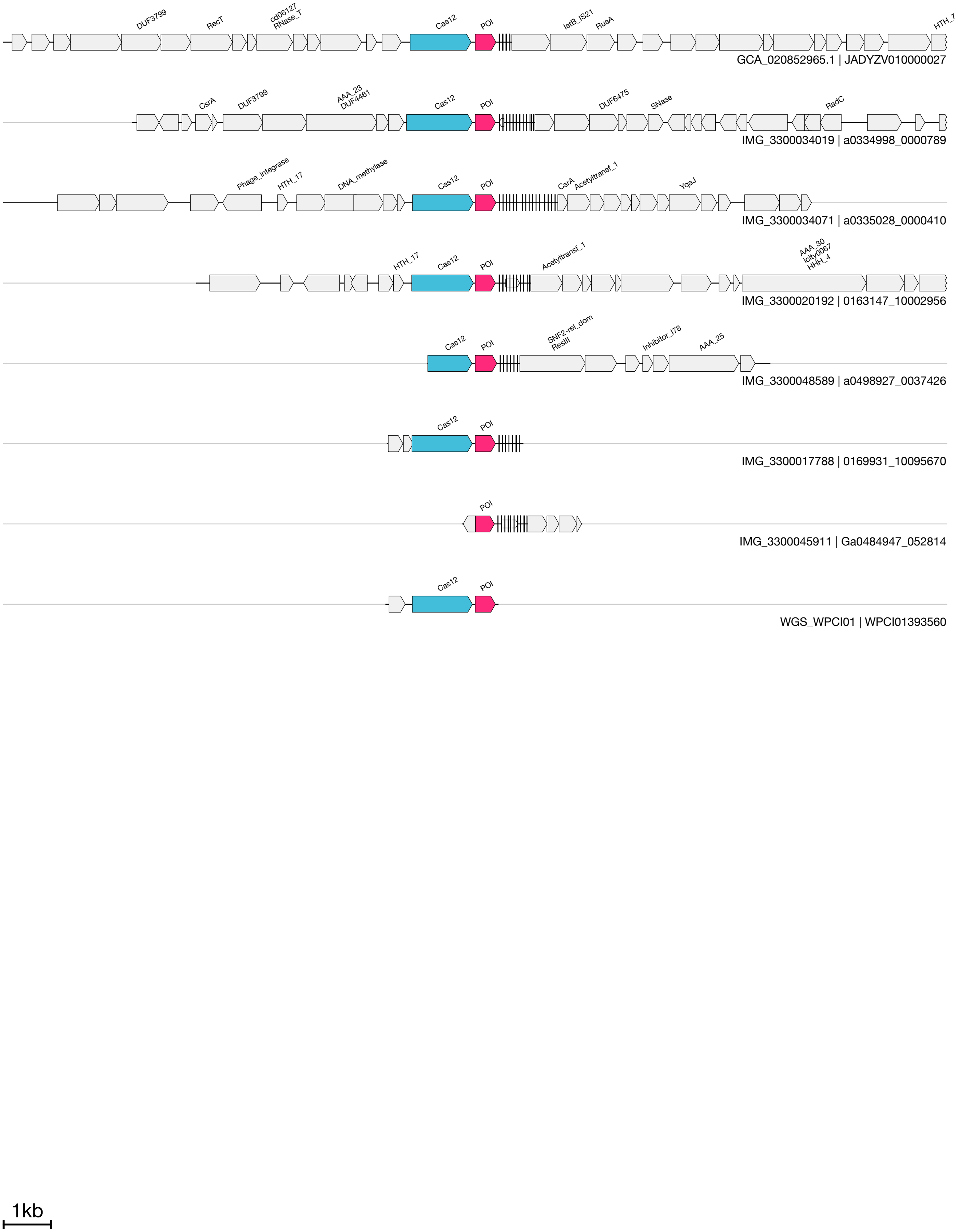

FI

UAS-155 **(transmembrane_vWA + DUF4407)** 3 / 4.4

Auxiliary IMG_3300048987&&a0467049_0000162&&19133_19934_1

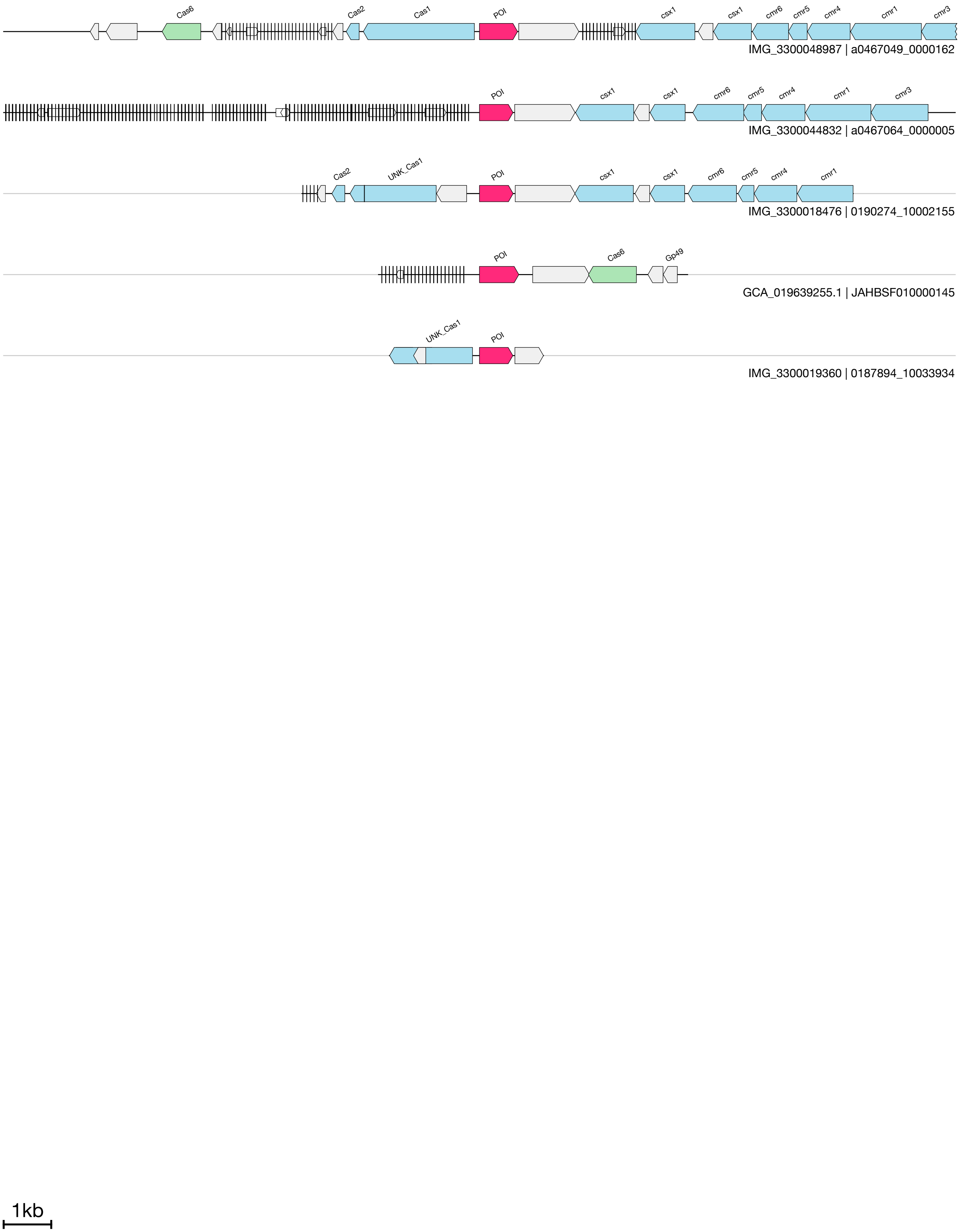

FJ

UAS-156 **(Mrr nuclease + ATPase)** N/A

Auxiliary WGS_CADHRT01&&CADHRT010000022&&25862_26885_1

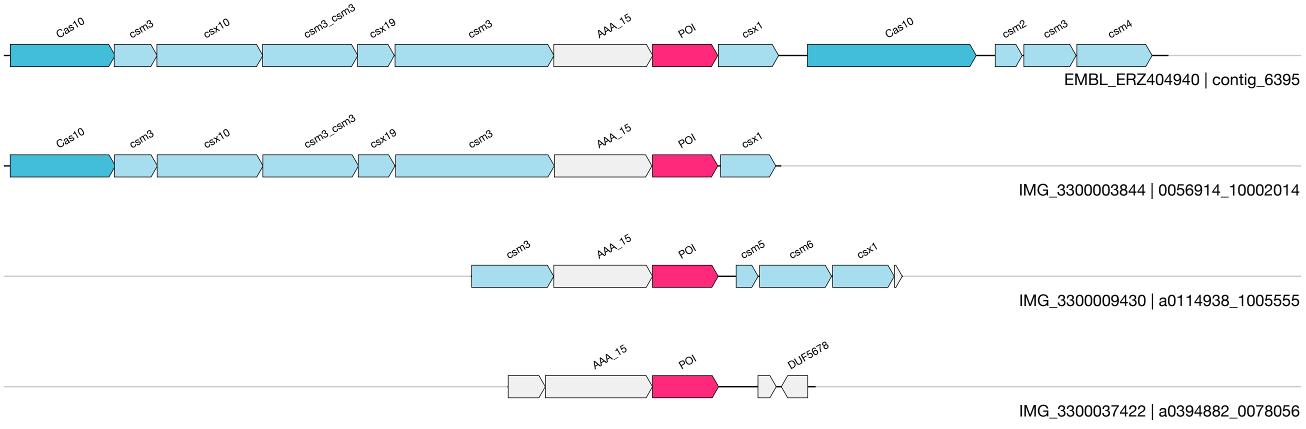

FK

UAS-33 **(Prok_E2 + ThiF + ThiS + NYN + CorA)** 34 / 50.0

Auxiliary WGS_JAAUYB01&&JAAUYB010070855&&3244_3730_1

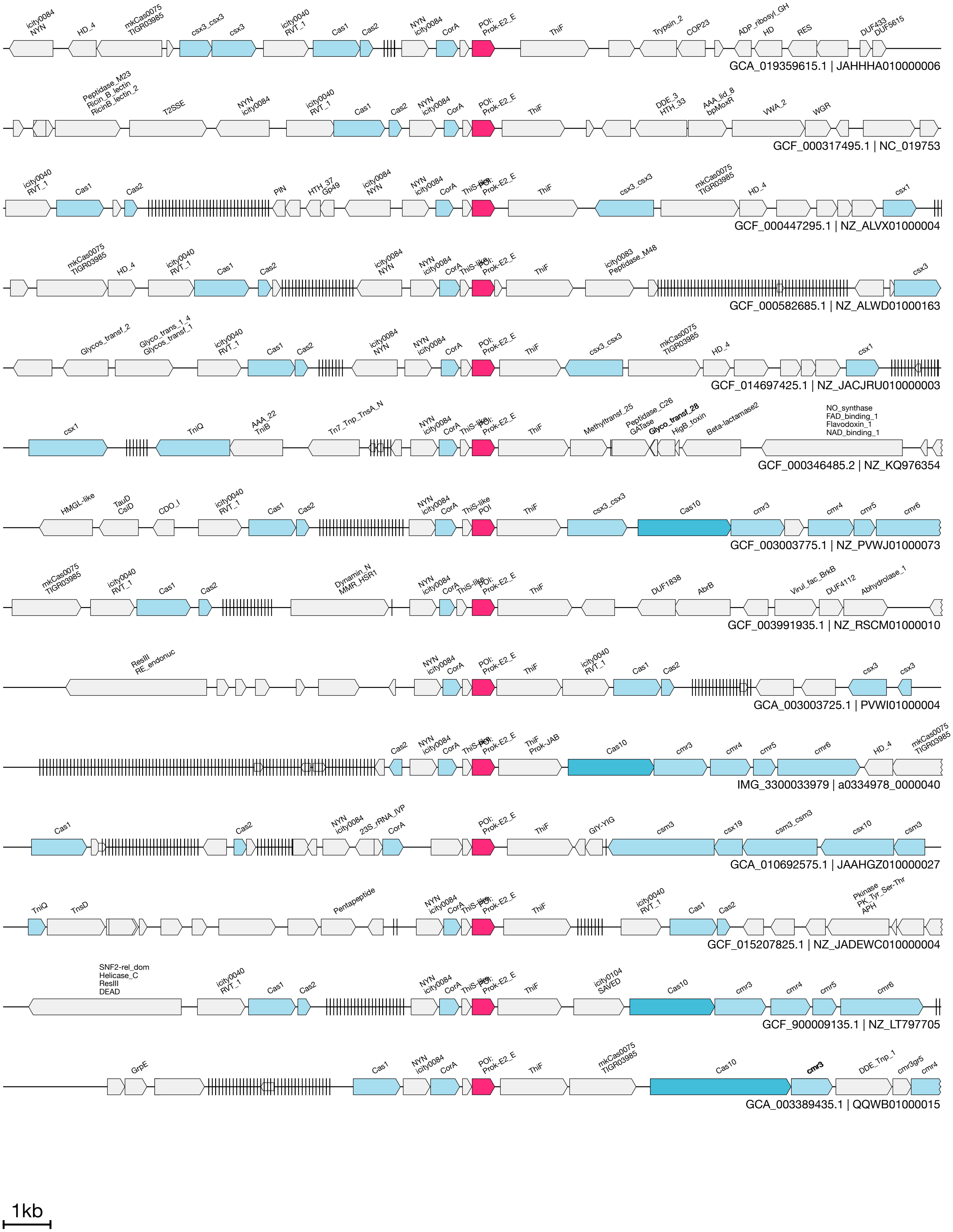

FL

UAS-157
Auxiliary

(RecJ_HD)
WGS_MTBK01&&MTBK01177382&&2891_4688_1

19 / 25.4

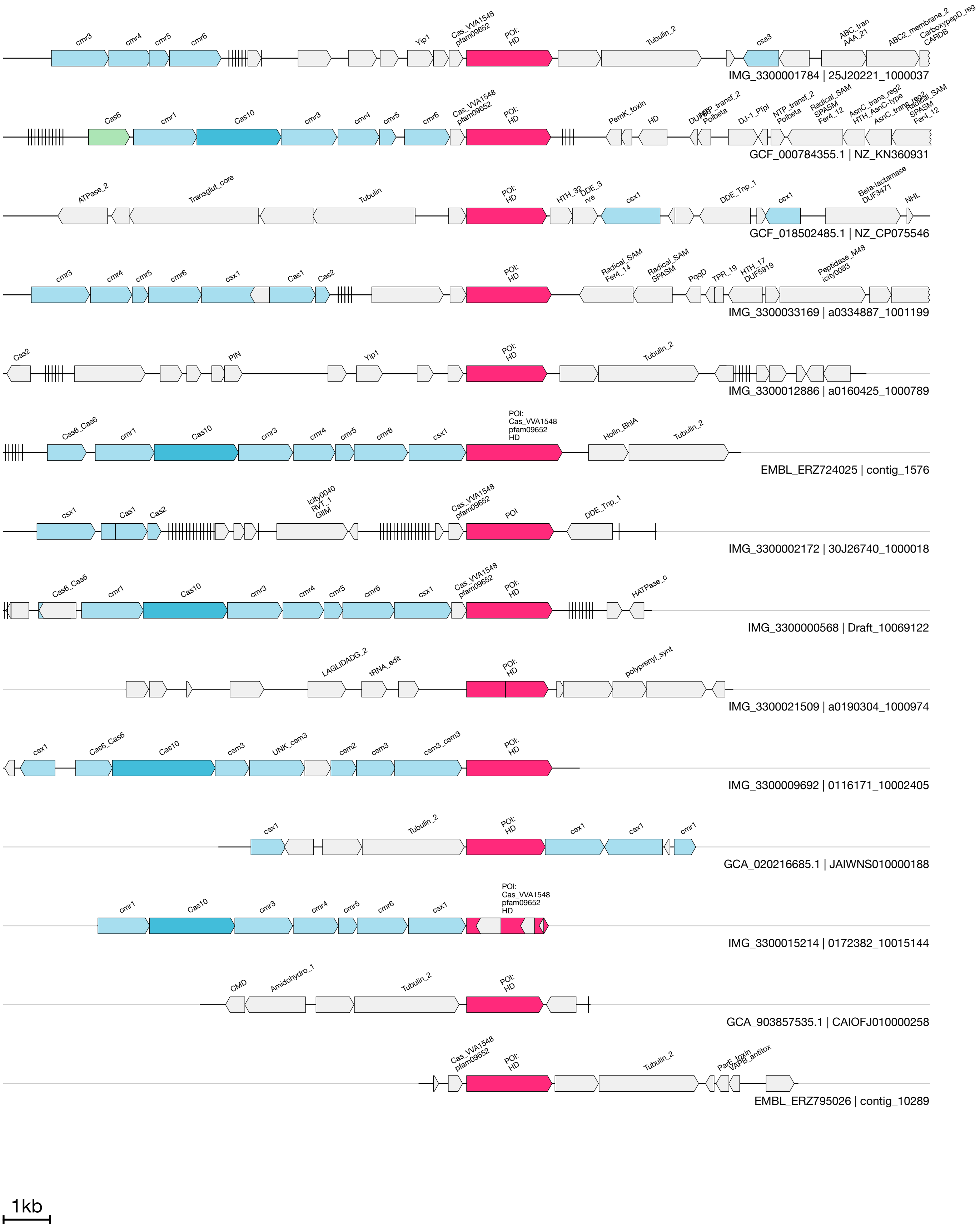

FM

UAS-34
Toxin-Antitoxin

(DarT DarG toxin-antitoxin ribosylation)
EMBL_ERZ1033877&&contig_98&&37922_38591_-1

15 / 22.9

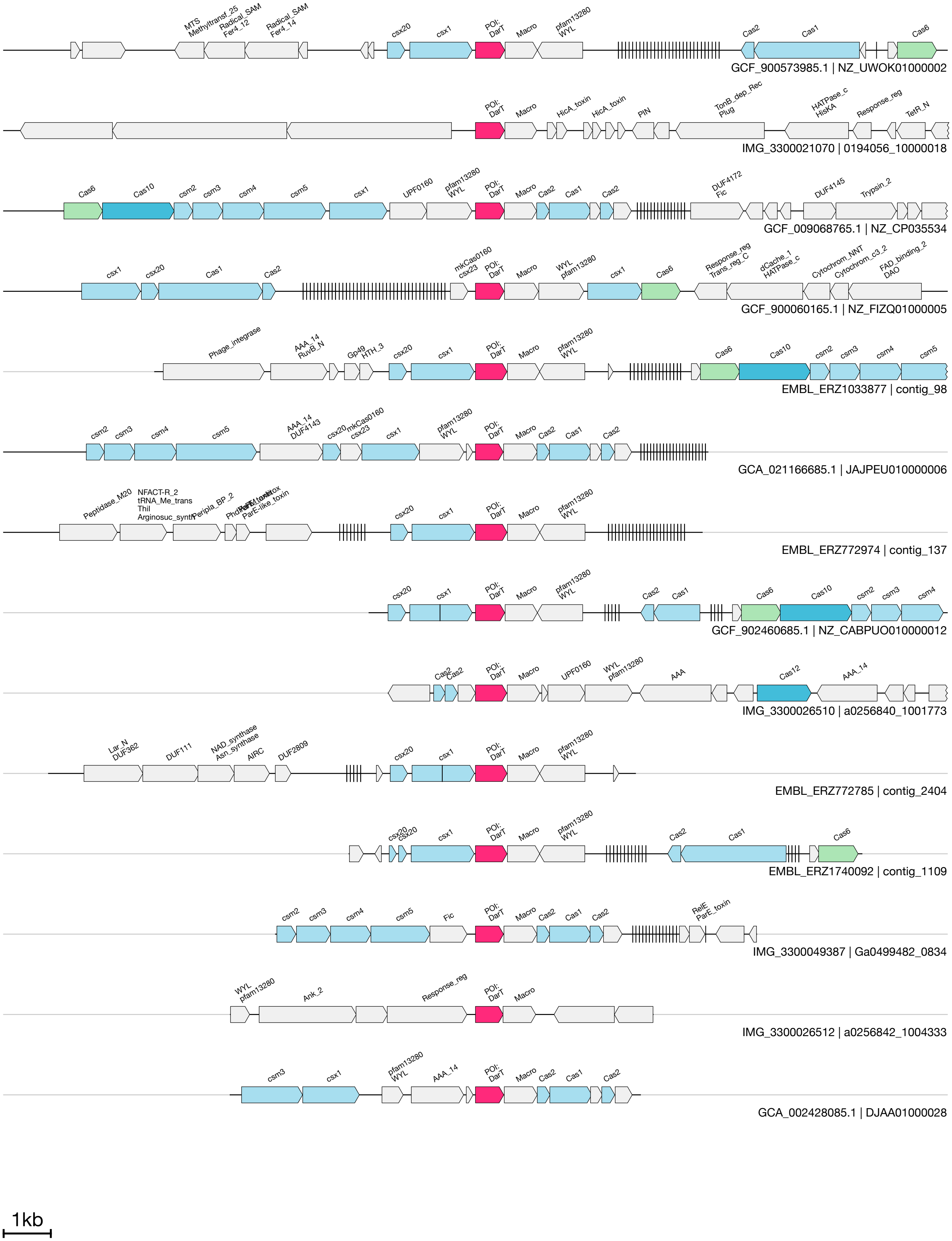

FN

UAS-35 **(addiction gene orf associated with Cas9)** 9 / 9.0

Toxin-Antitoxin EMBL_ERZ1505400&&contig_18950&&117_258_1

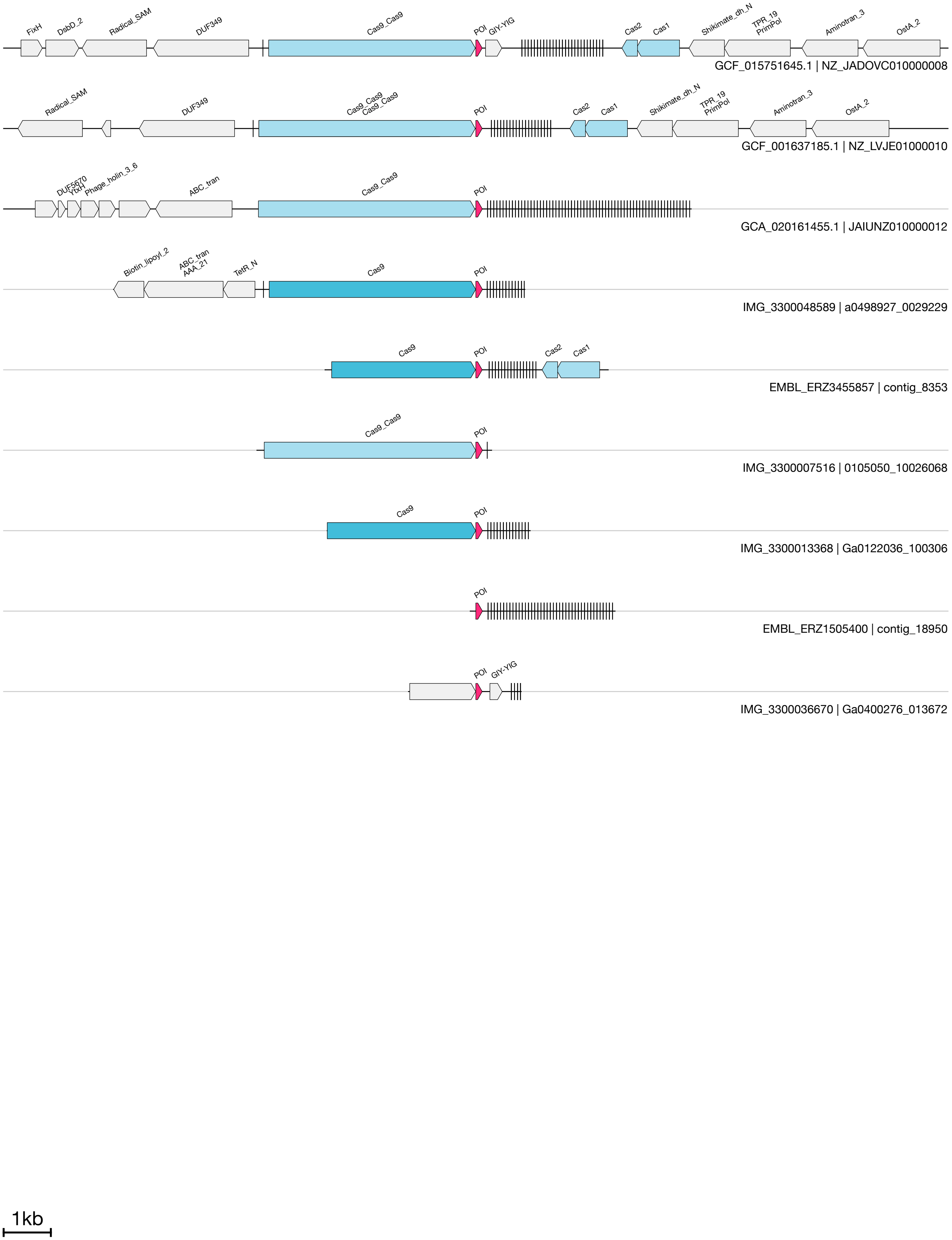

FO

UAS-158 **(AbiE + DUF87)** 8 / 18.7
Toxin-Antitoxin EMBL_ERZ1742239&&contig_29570&&162_1638_1

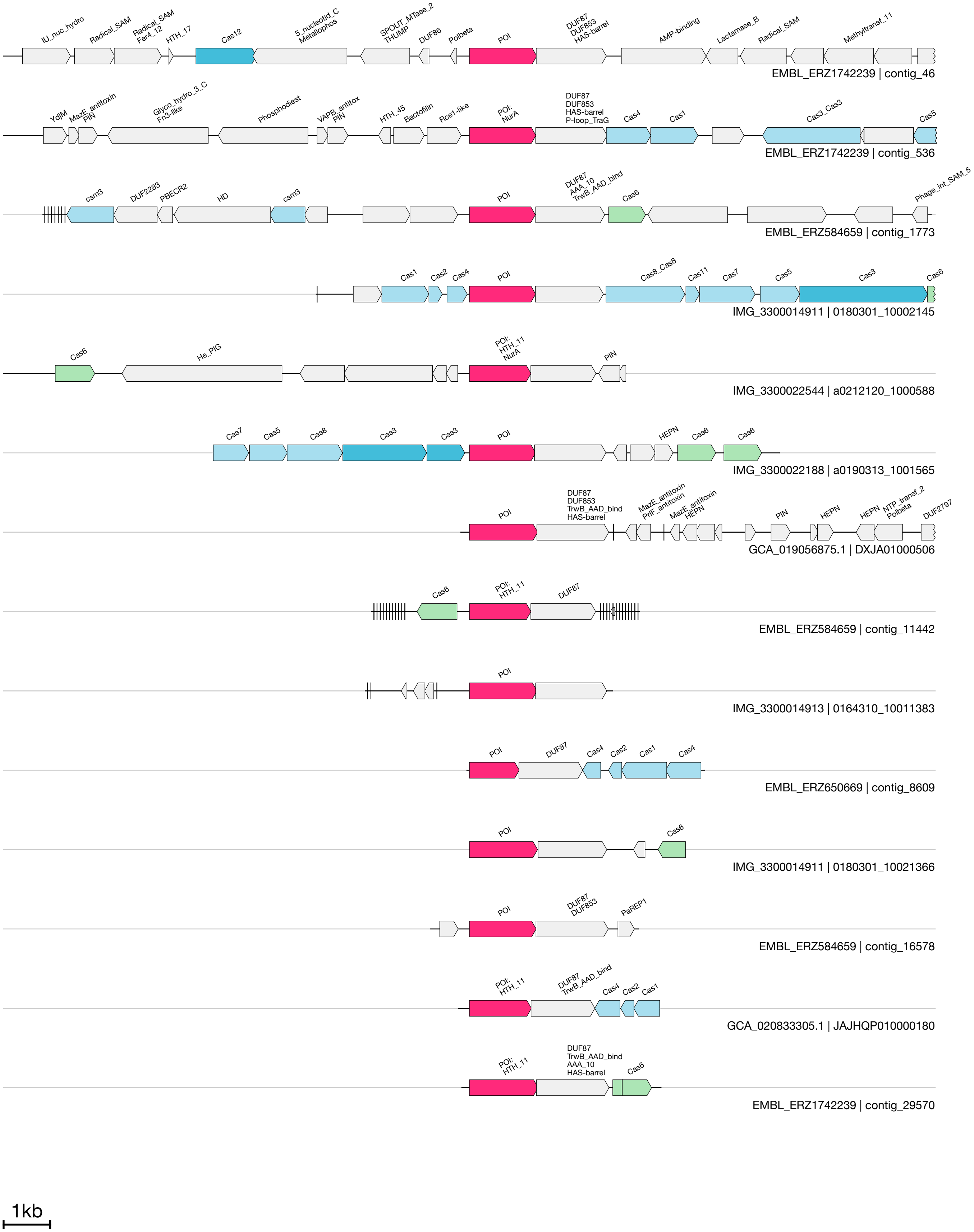

FP

UAS-159 | **(Nucleotidyltransferase HEPN, antitoxin)** | 18 / 48.1

Toxin-Antitoxin

EMBL_ERZ511474&&contig_784&&79398_79716_-1

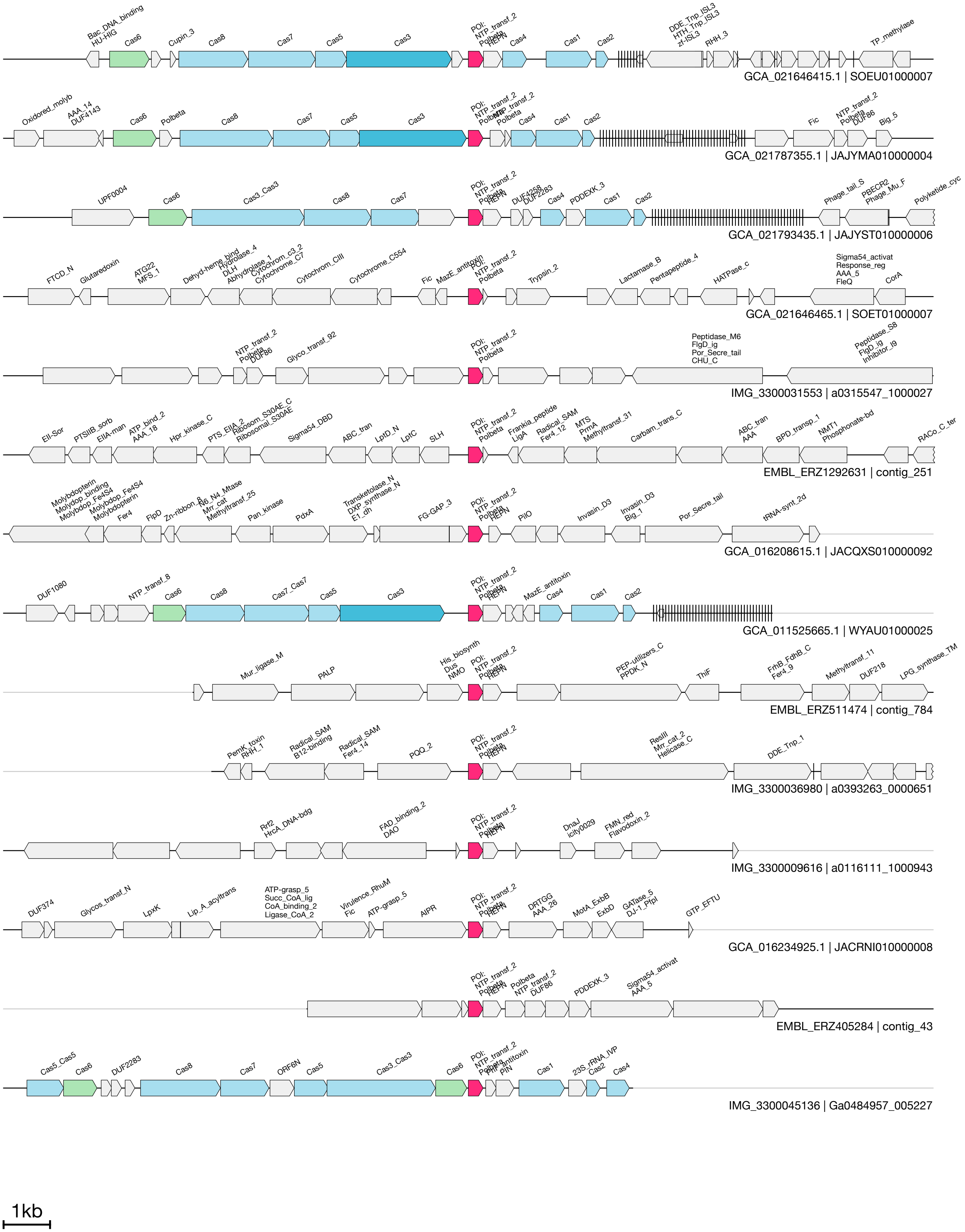

FQ

UAS-160
Toxin-Antitoxin

(DUF86 + Nucleotidyltransferase)
GCA_013285545.1&&JAAXLU010000071&&33674_34109_-1

104 / 173.3

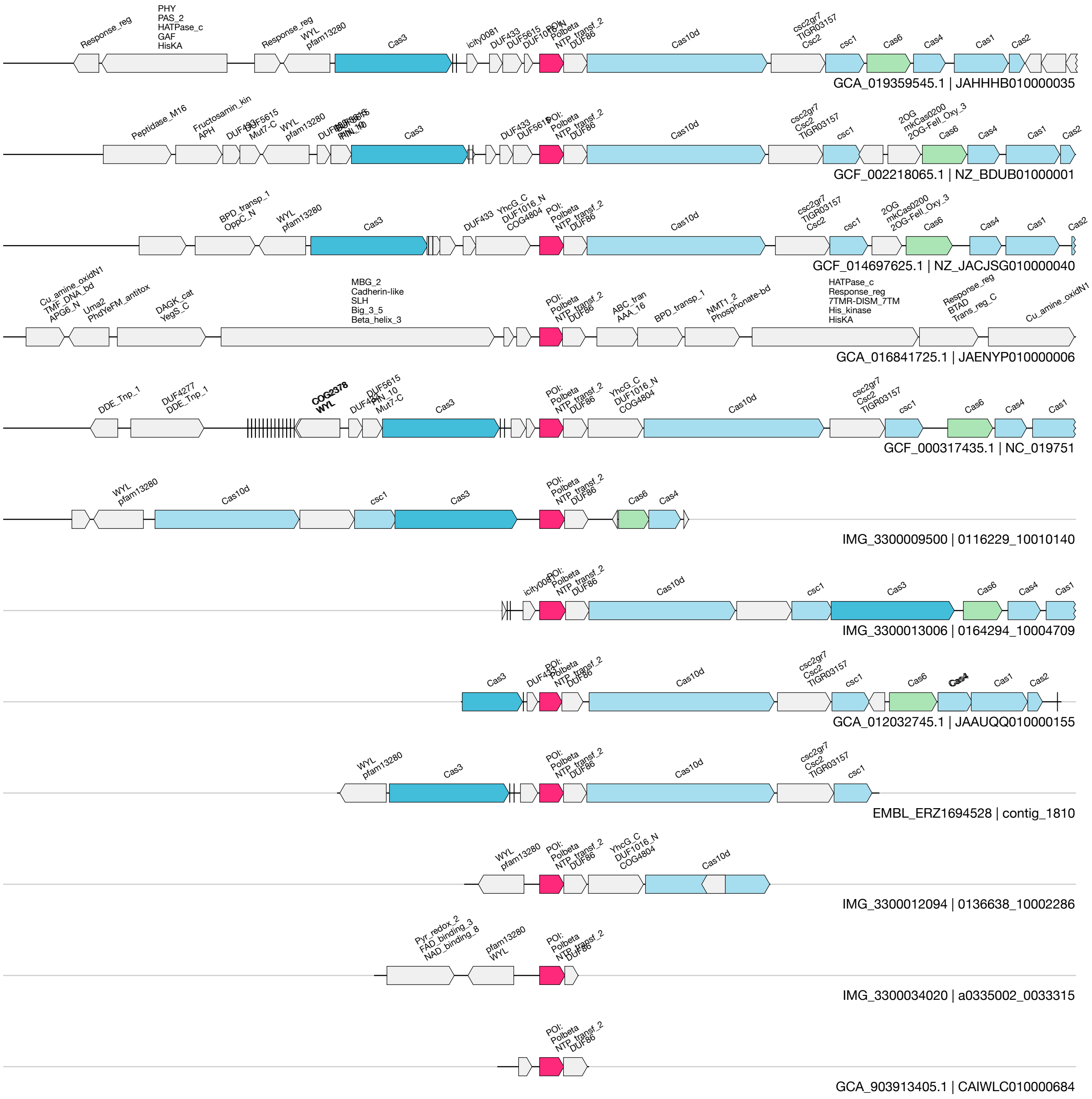

1kb

FR

UAS-161 **(Cas9 HicAB TA)** 3 / 3.0

Toxin-Antitoxin GCF_003337175.1&&NZ_NETH01000035&&6357_6546_1

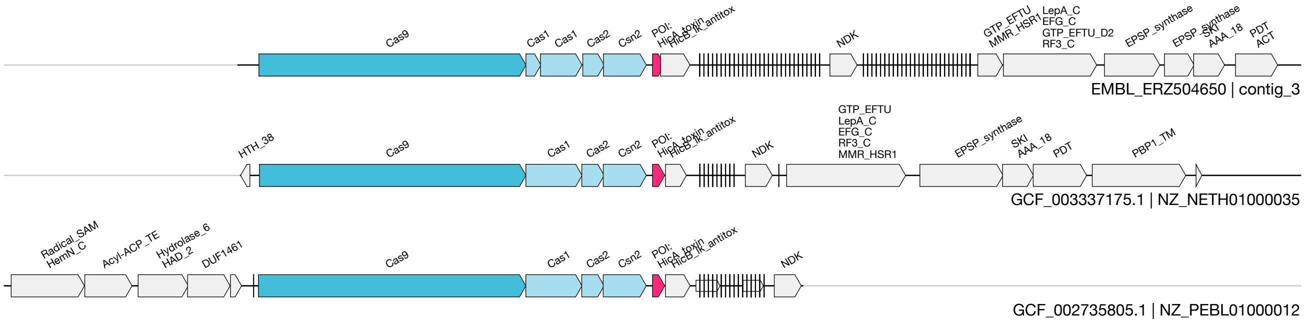

1kb

FS

UAS-162
Toxin-Antitoxin

(Cas9 TA system)
GCF_012037625.1&&NZ_JAAVJS010000002&&280535_280832_-1

25 / 36.4

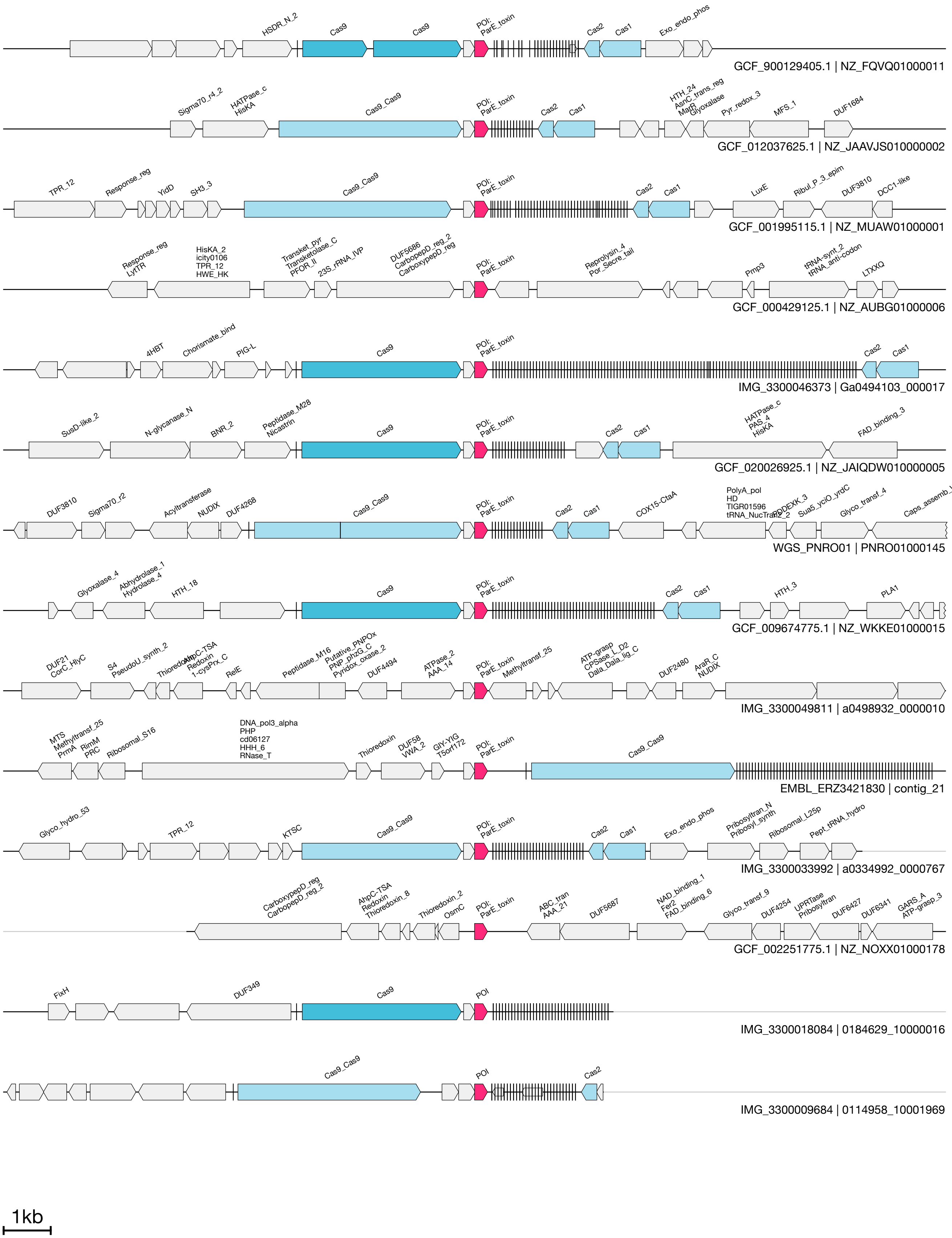

FT

UAS-163 **(nucleotidyltransferase + HTH)** 4 / 6.9
Toxin-Antitoxin IMG_3300001380&&6J14229_10033242&&787_1210_1

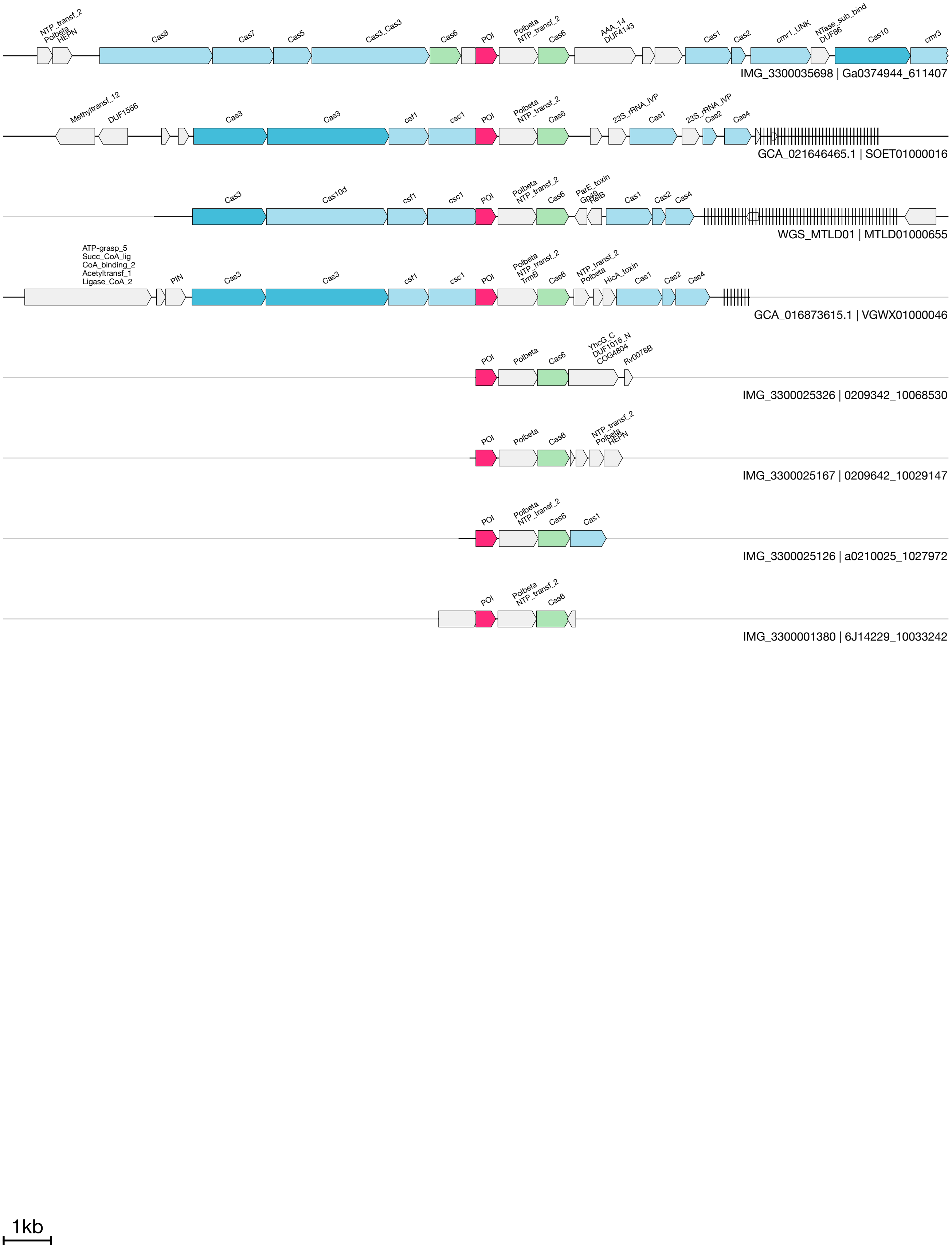

UAS-164 **(AbiEii)** 2 / 5.2
Toxin-Antitoxin IMG_3300005452&&a0068706_1004208&&1242_2085_1

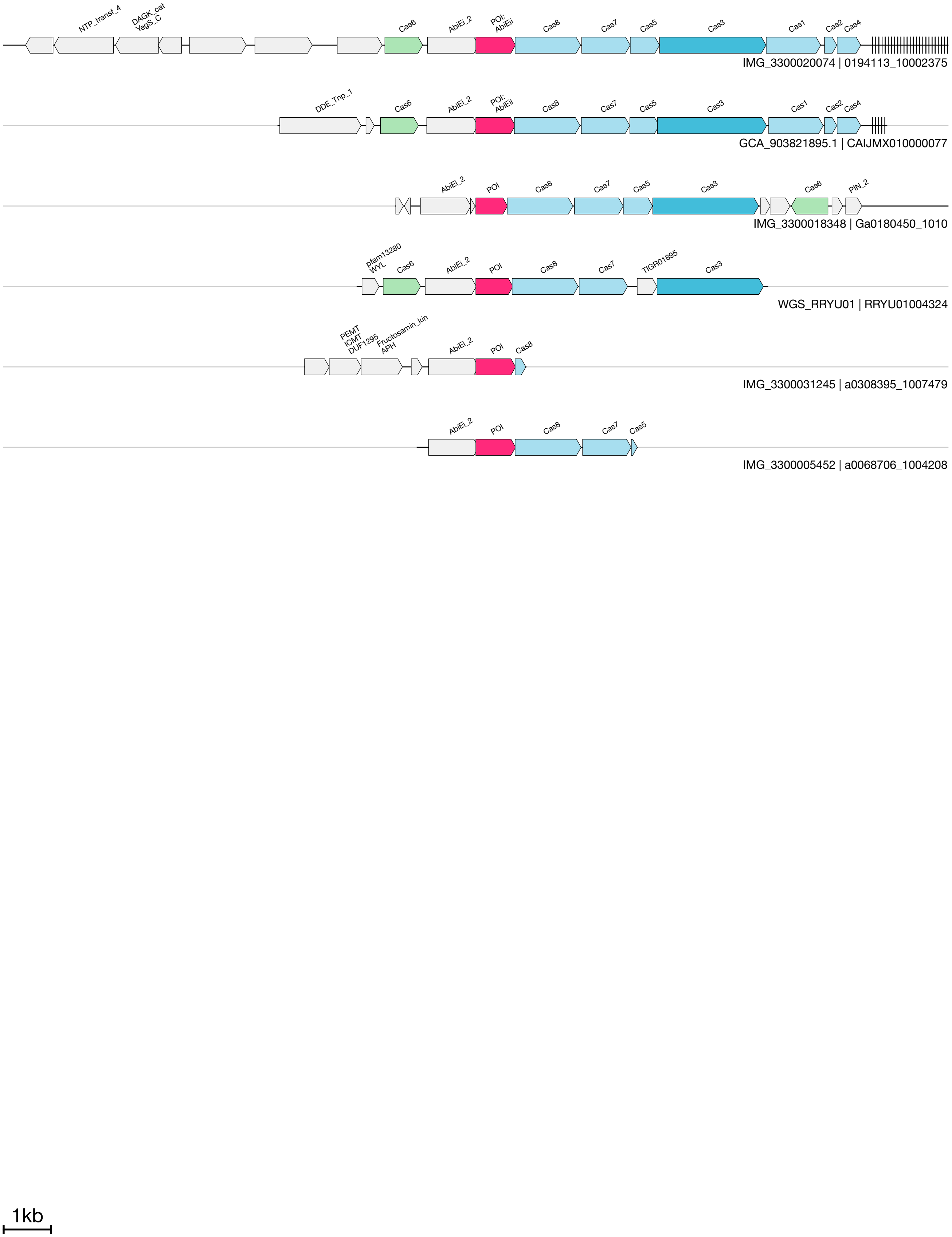

FV

UAS-165
Toxin-Antitoxin

(TA DUF3368 _ UPF0175)
IMG_3300009455&&0114939_10000745&&24543_24870_1

11 / 16.6

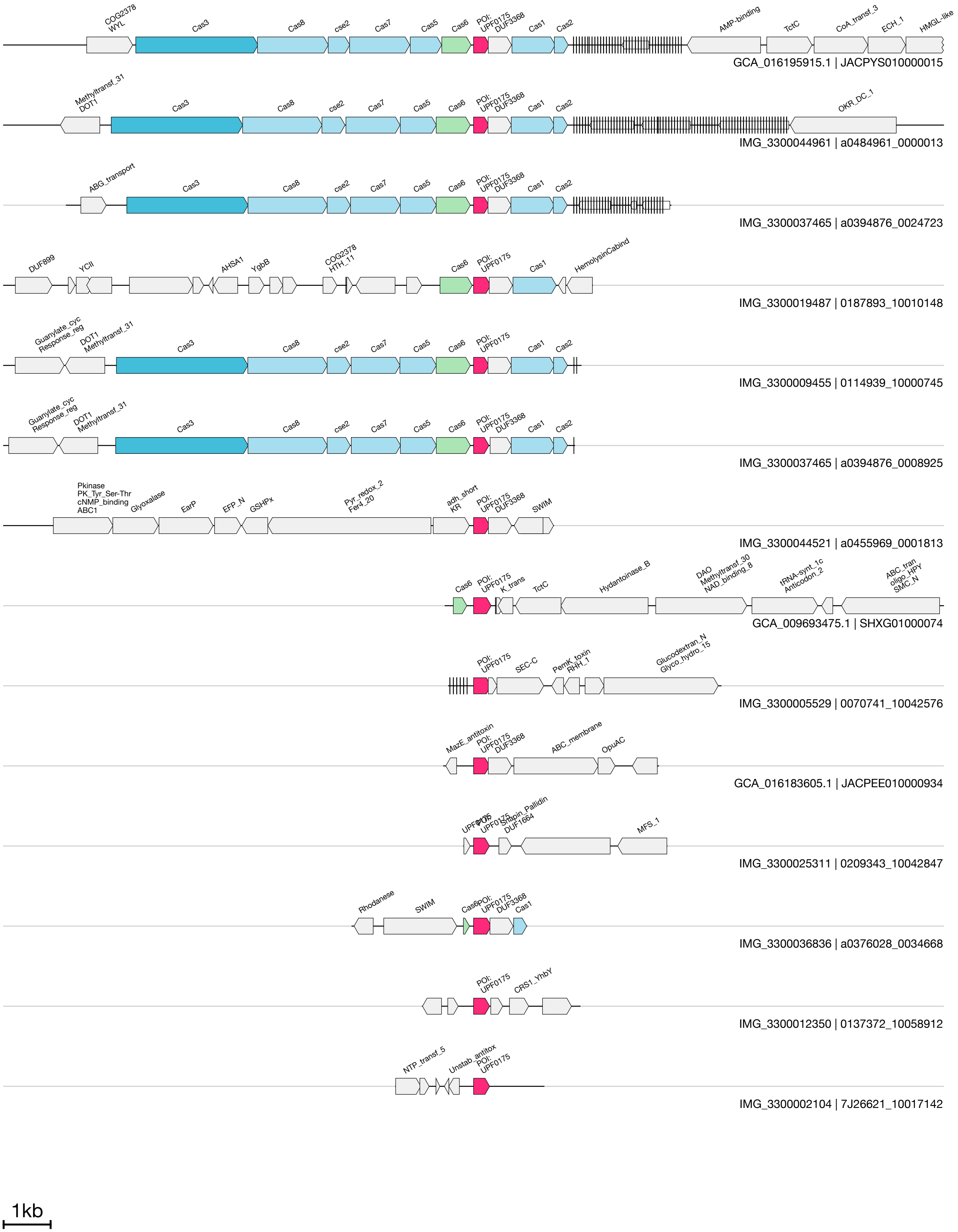

FW

UAS-166
Toxin-Antitoxin

(AbiEi + AbiEii)
IMG_3300013103&&0164318_10140015&&1204_2056_1

N/A

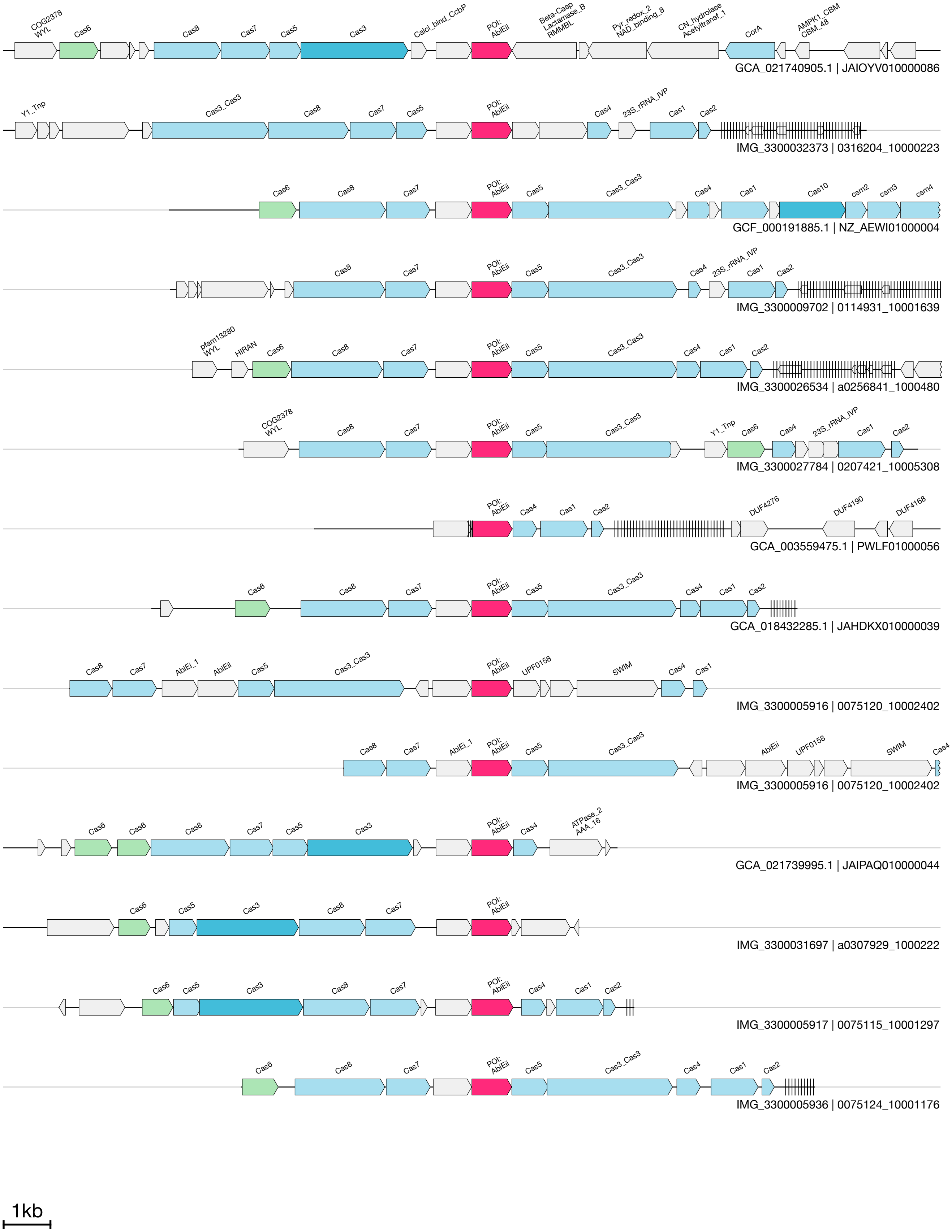

FX

UAS-167 **(TA system)** 7 / 7.3

Toxin-Antitoxin IMG_3300014205&&0172380_10021645&&1390_1759_1

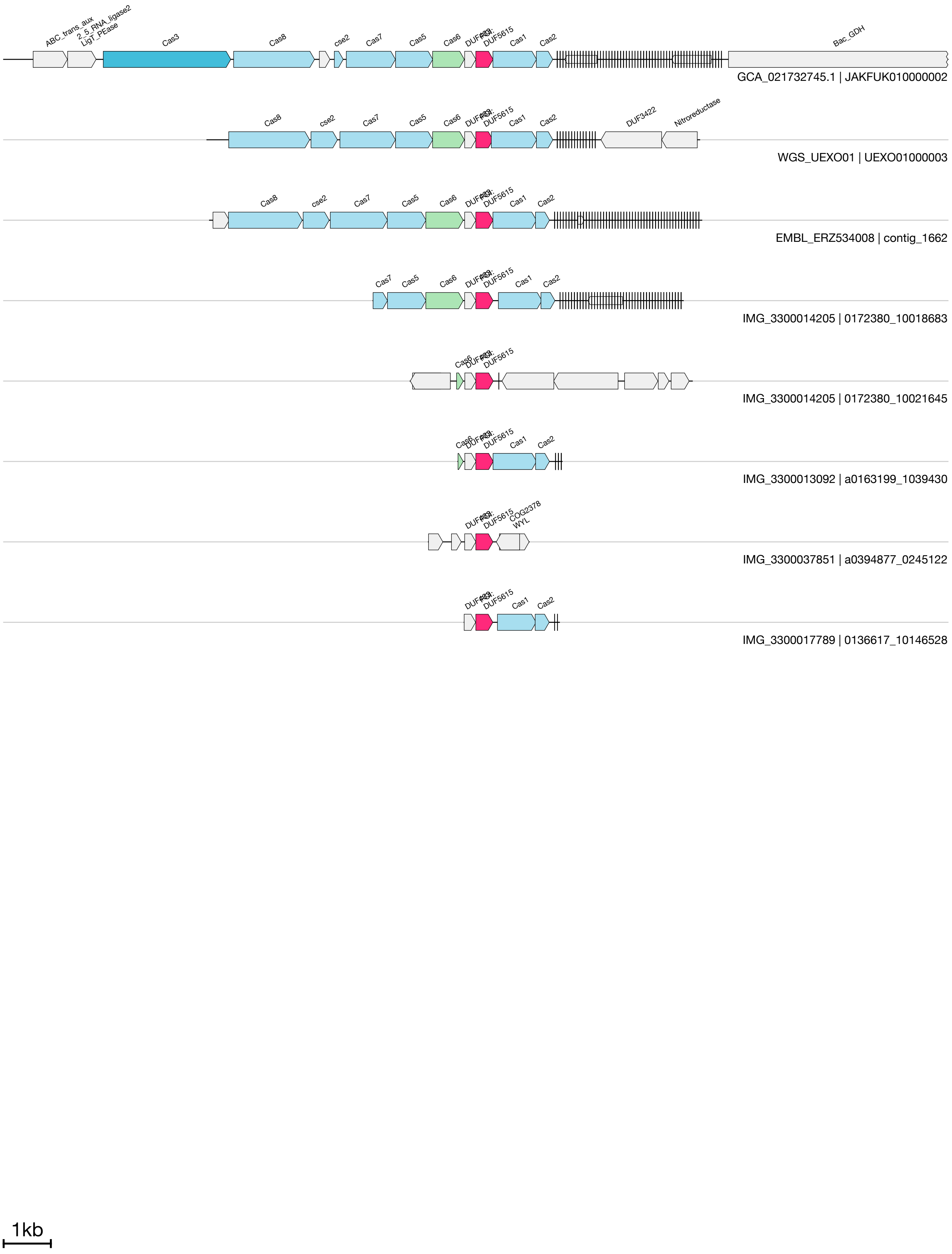

FY

UAS-168 **(PHD + PIN TA)** 3 / 3.9

Toxin-Antitoxin IMG_3300025308&&0209211_10037079&&3340_3646_-1

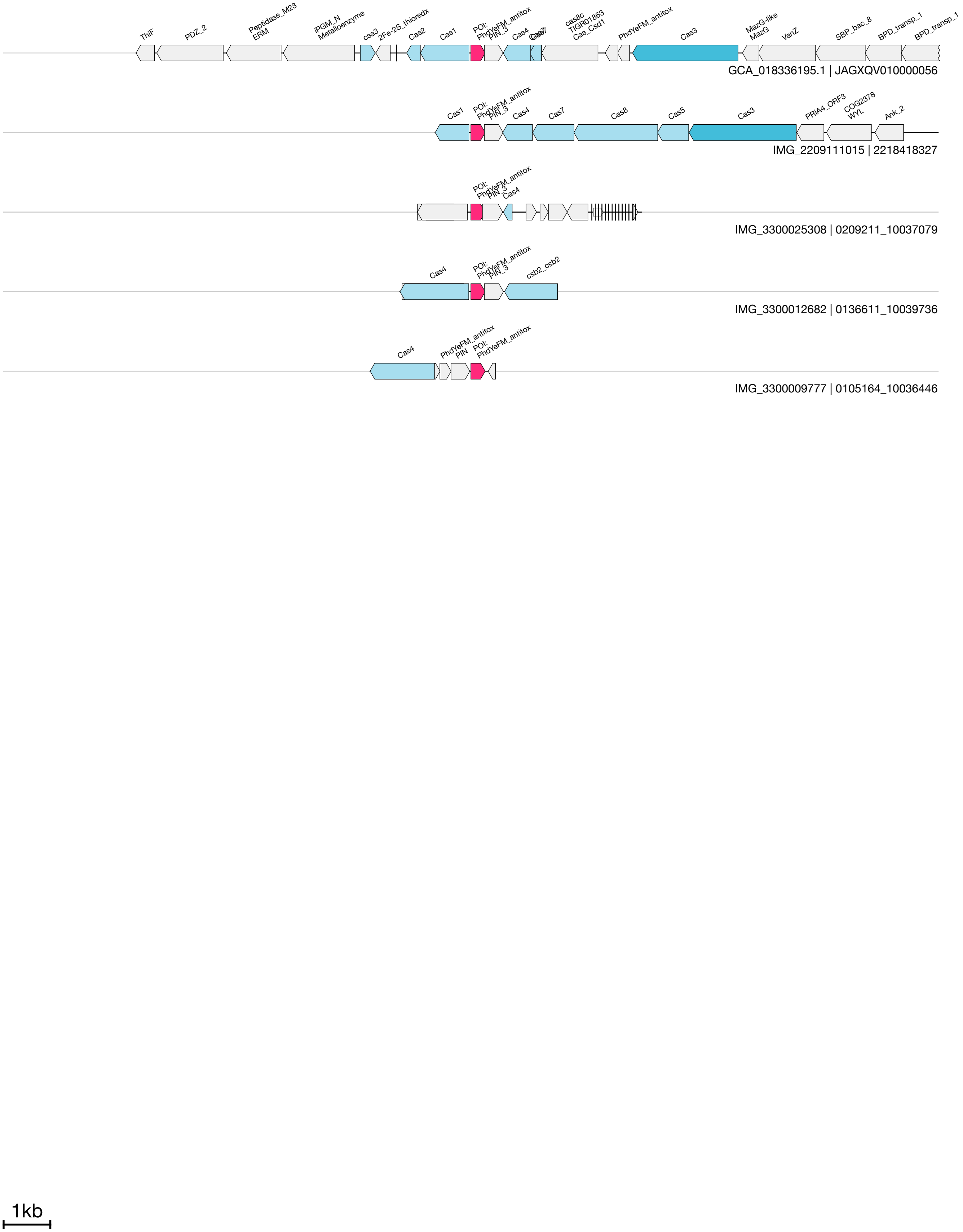

FZ

UAS-169 **(DUF370 + RNaseH TA)** 3 / 5.2

Toxin-Antitoxin IMG_3300031358&&a0307438_1001316&&8395_8671_1

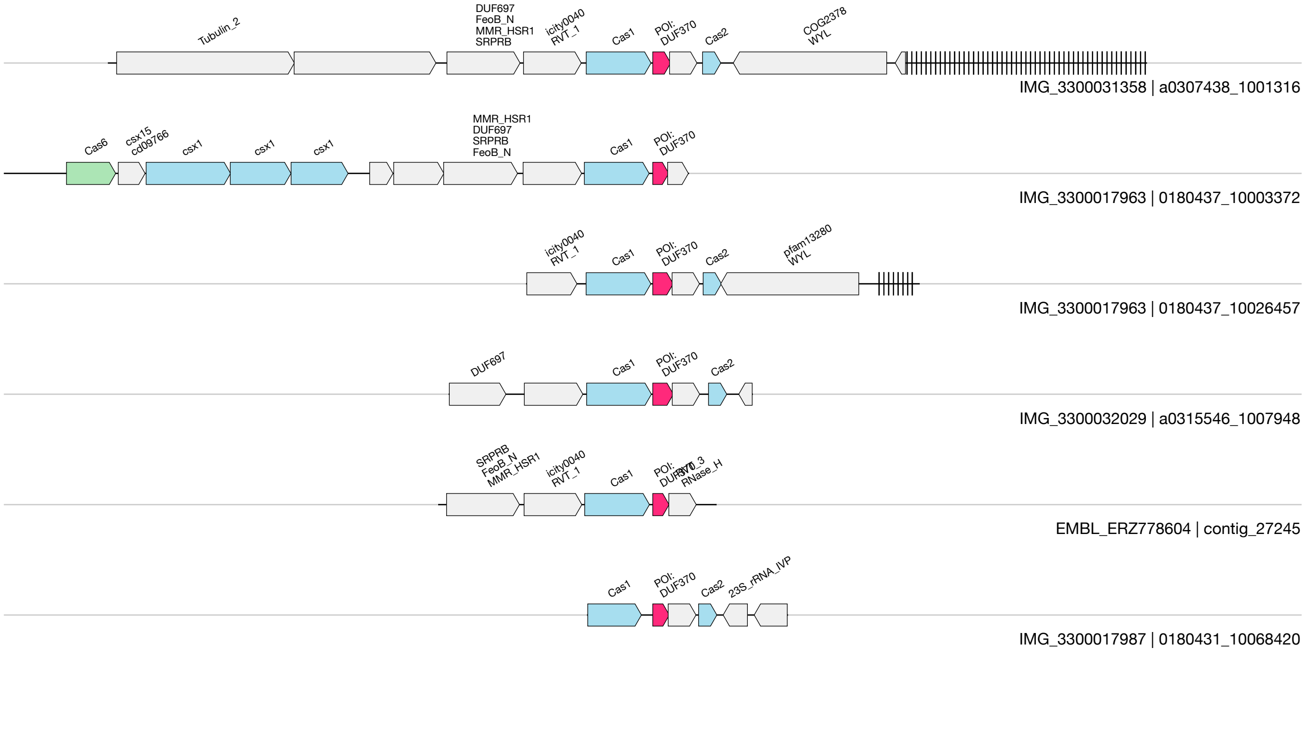

GA

UAS-170
Toxin-Antitoxin

(DUF2442 + DUF4160)
IMG_3300034066&&a0335019_0002251&&7799_8117_-1

3 / 3.0

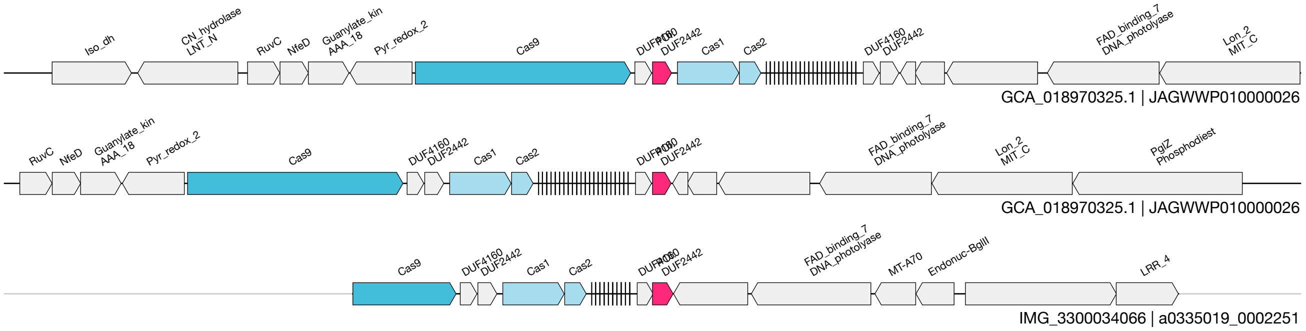

1kb

GB

UAS-171 **(small ORFs)** 43 / 48.3

Toxin-Antitoxin IMG_3300044960&&Ga0484958_001236&&27209_27470_-1

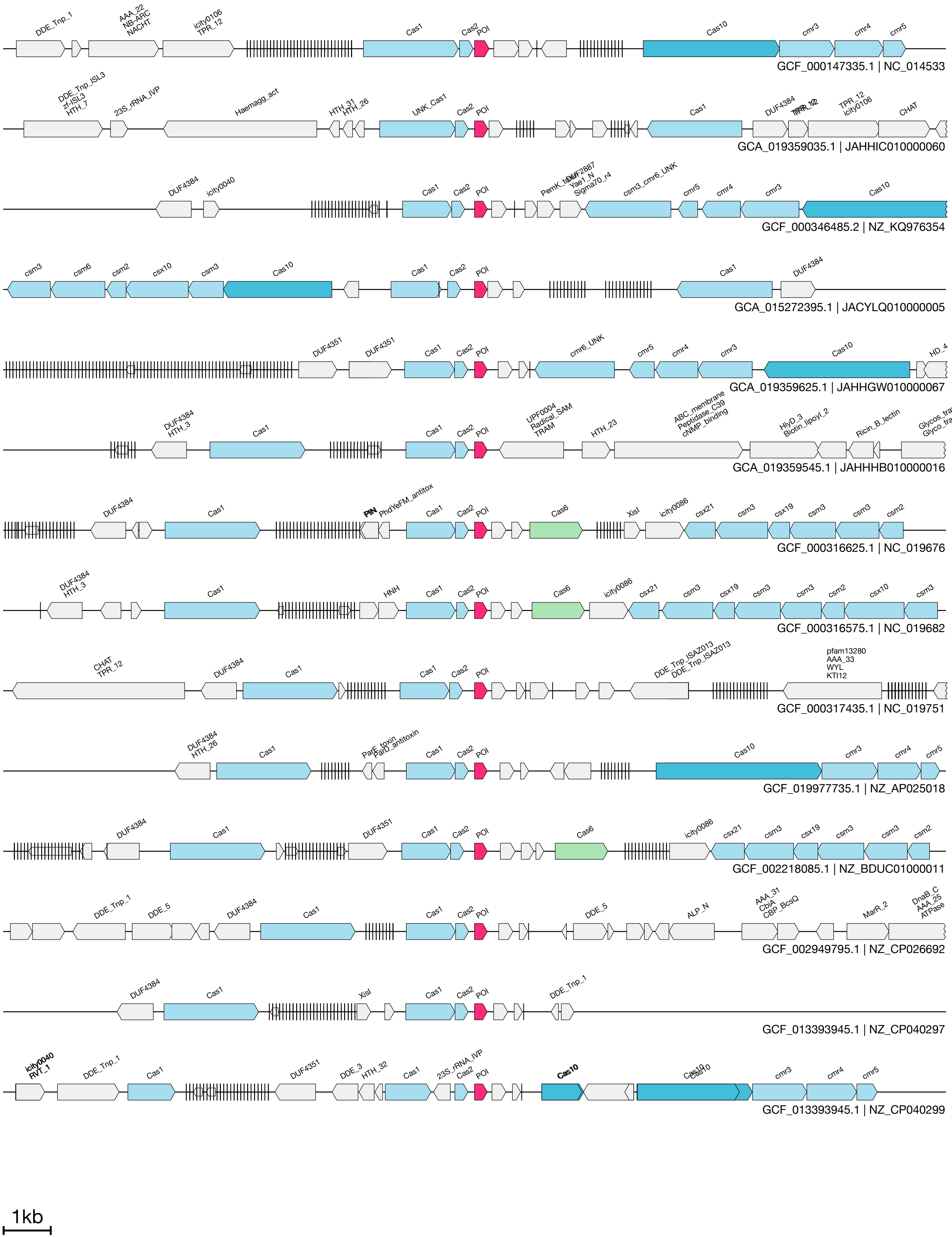

GC

UAS-172 **(RHH TA system)** 23 / 35.0

Toxin-Antitoxin IMG_3300045747&&a0485825_0053347&&1617_1944_1

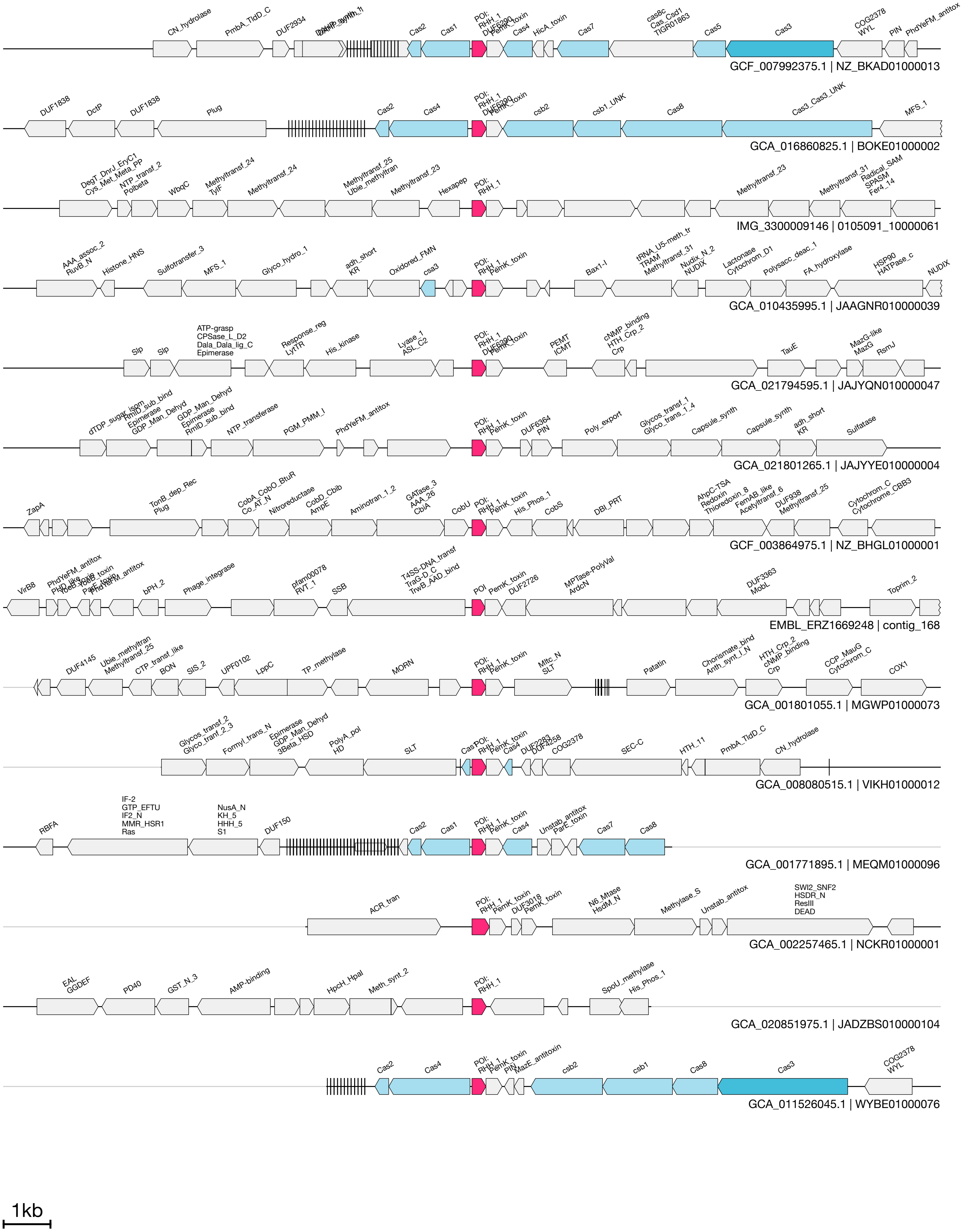

GD

UAS-173
Toxin-Antitoxin

(DUF3368 TA system)
MG-RAST_mgm4611649.3&&465870&&0_567_1

42 / 56.2

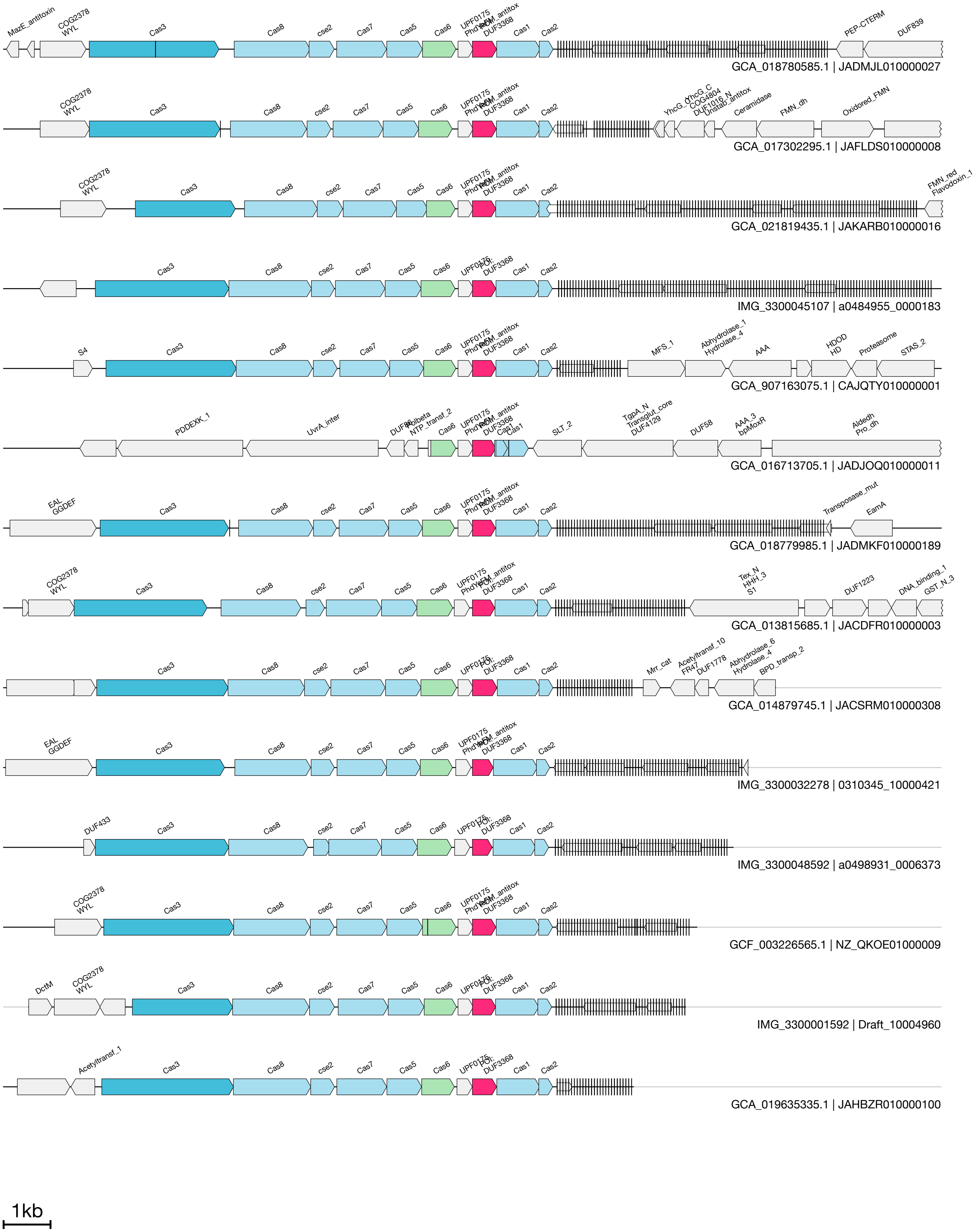

GE

M2 **(massive defense genes)** 3 / 3.0

Giant EMBL_MGYG000003147&&MGYG000003147_7&&47692_56296_-1

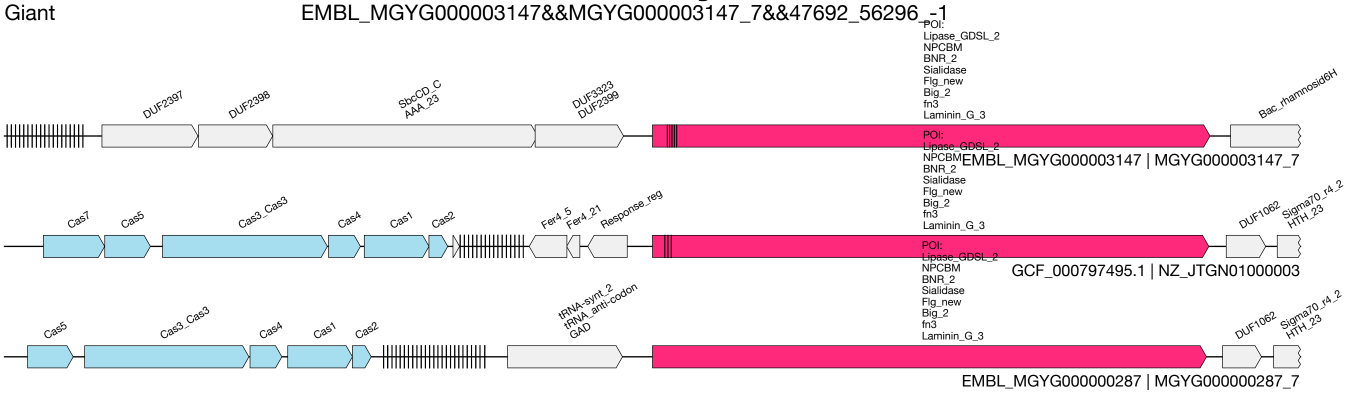

1kb

GF

M1
Giant

(massive defense system)
WGS_BOFR01&&BOFR01000007&&165916_179089_-1

6 / 13.3

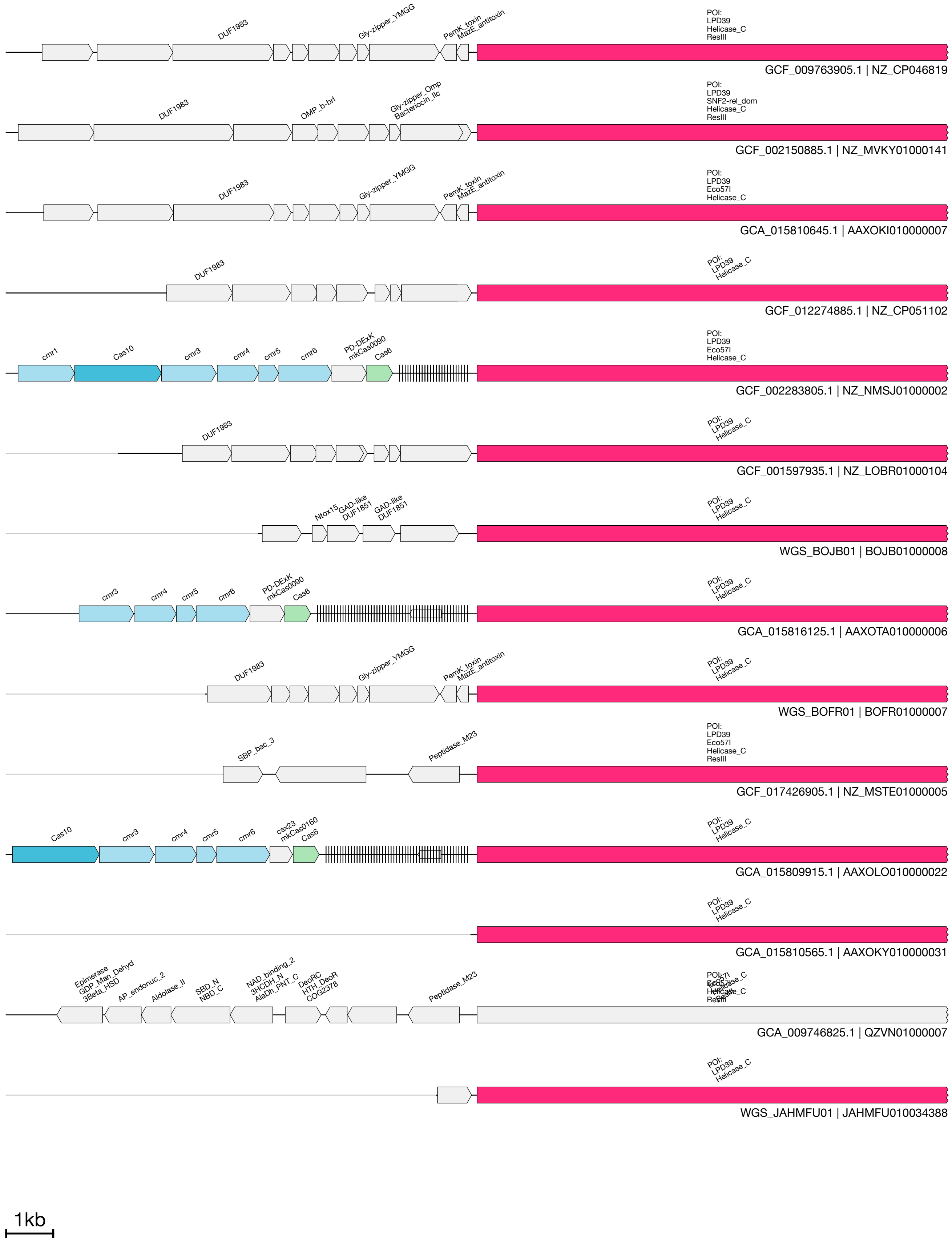

GG

(GGDEF tRNA array)

174 / 347.6

Non-CRISPR_tRNA_arrays

EMBL_ERZ650666&&contig_7067&&448_2380_1

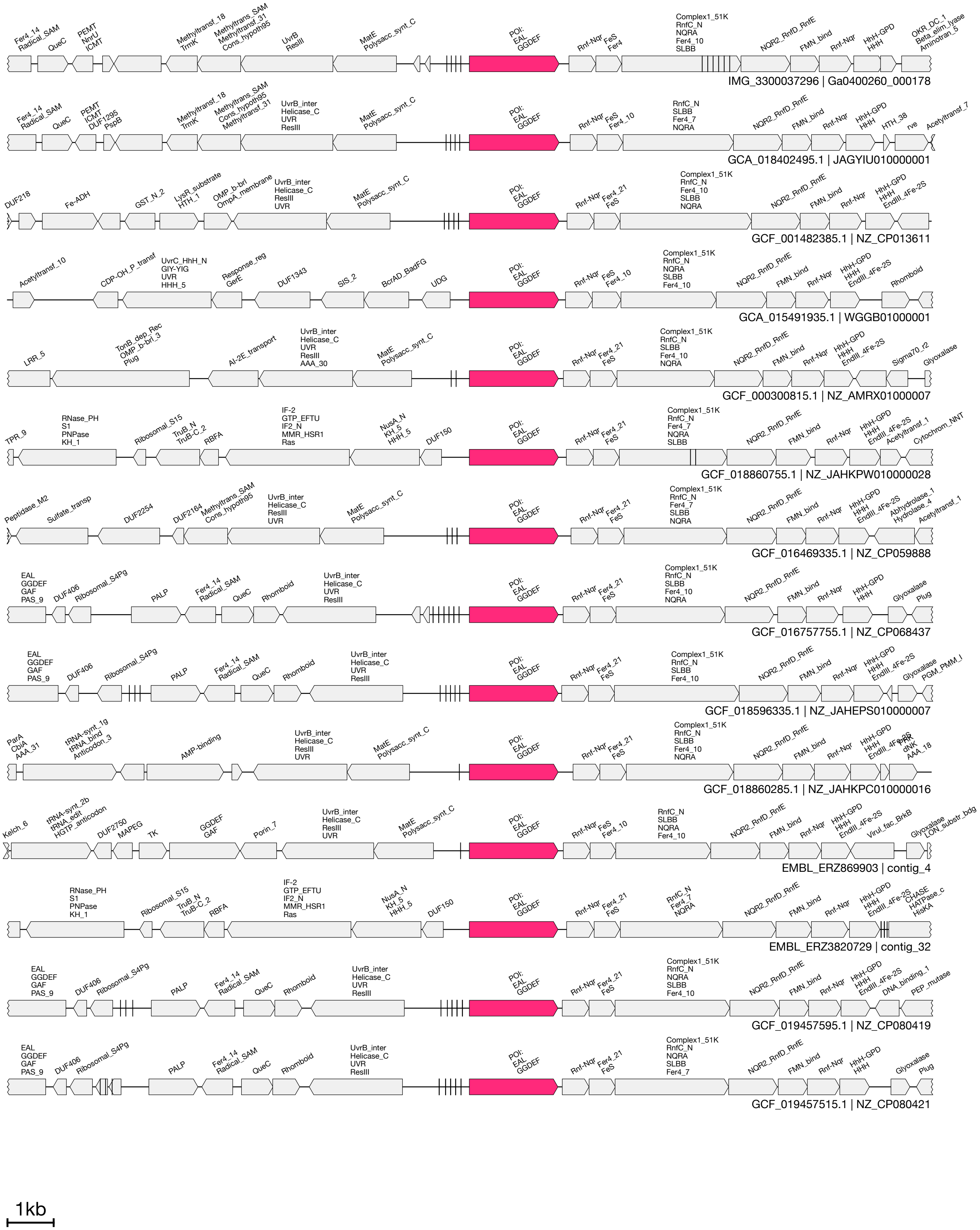

GH

(tRNA array DEDDh)

Non-CRISPR_tRNA_arrays GCF_016469335.1&&NZ_CP059888&&1400143_1401604_1

483 / 551.2

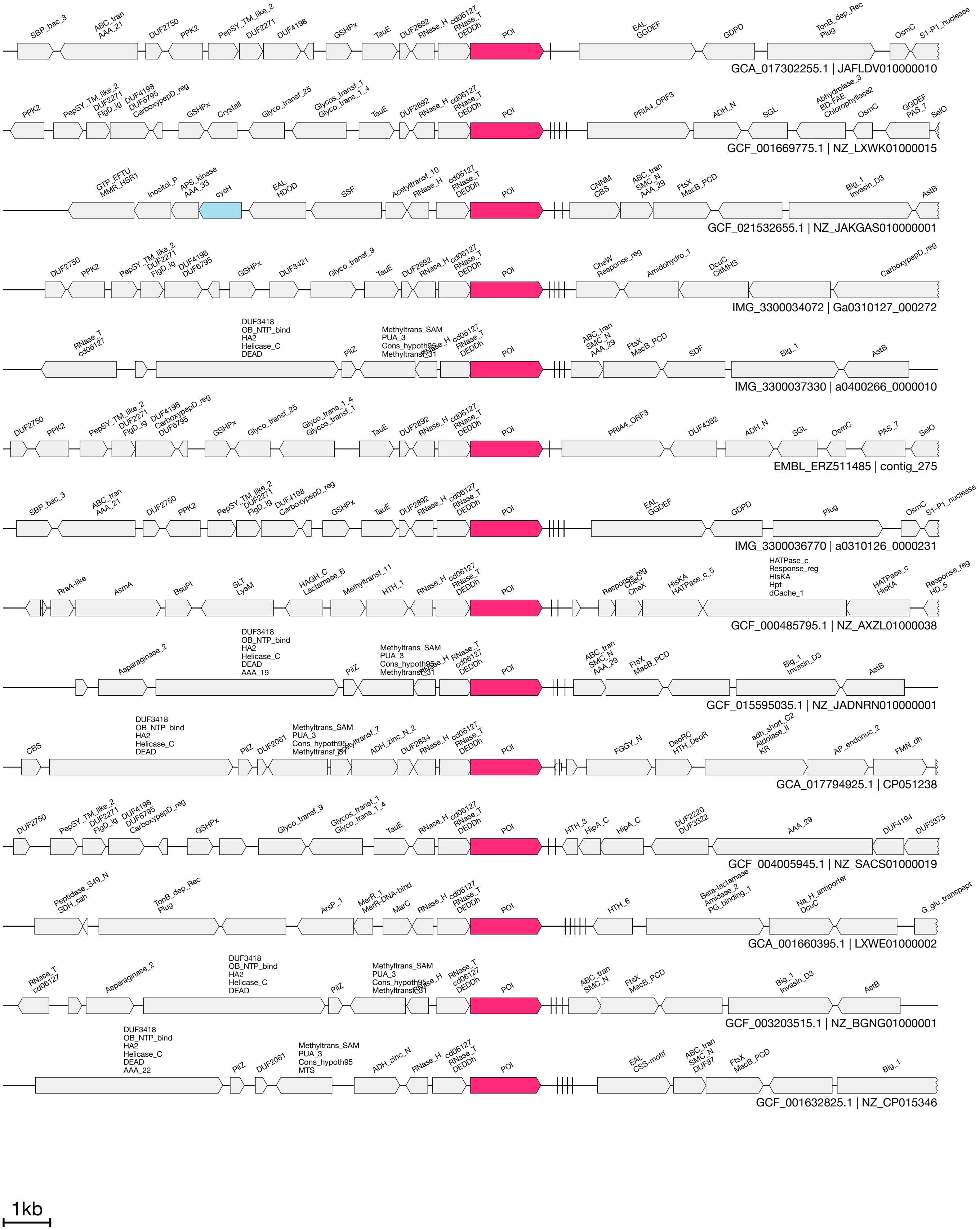

GI

(tRNA array ribonuclease)

104 / 112.6

Non-CRISPR_tRNA_arrays

IMG_3300012943&&0164241_10000118&&68729_72041_1

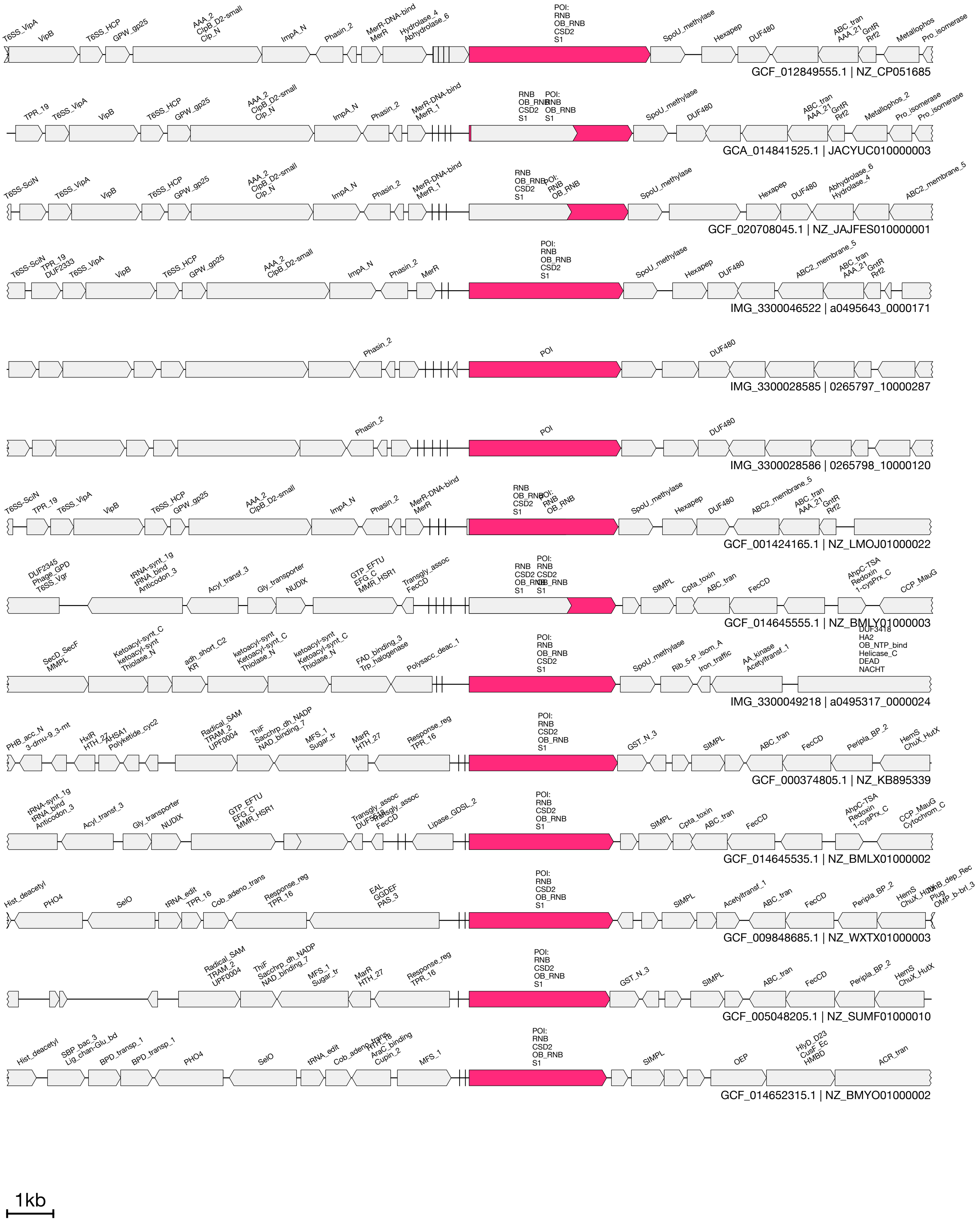

GJ

(DUF3800 standalone)

12 / 12.0

Non-CRISPR_hypervariable_repeat EMBL_ERZ795038&&contig_16642&&892_1681_1

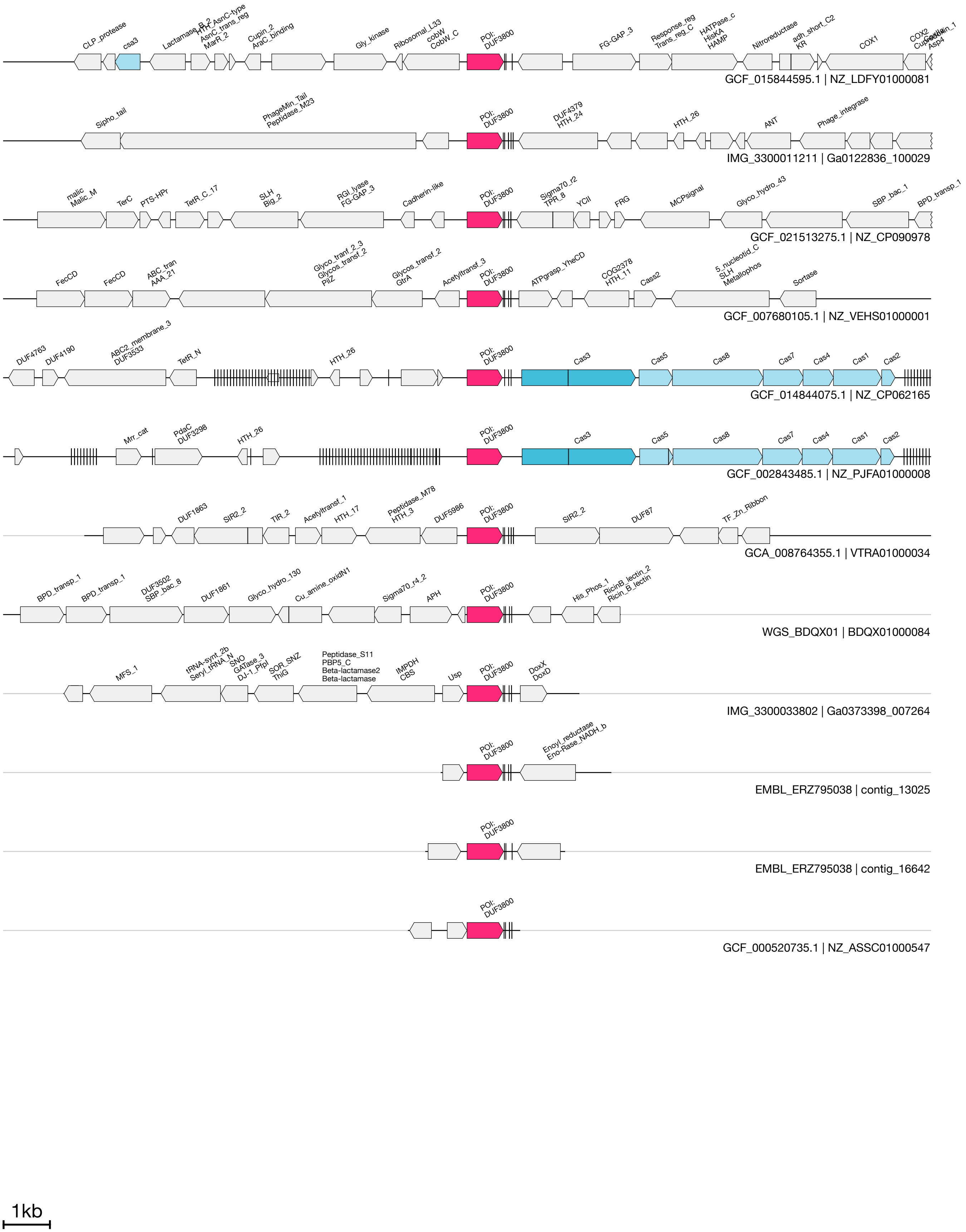

GK

(PDDEXK + Metallophosphatase) 44 / 47.4

Non-CRISPR_hypervariable_repeat EMBL_ERZ825177&&contig_7965&&5053_8428_1

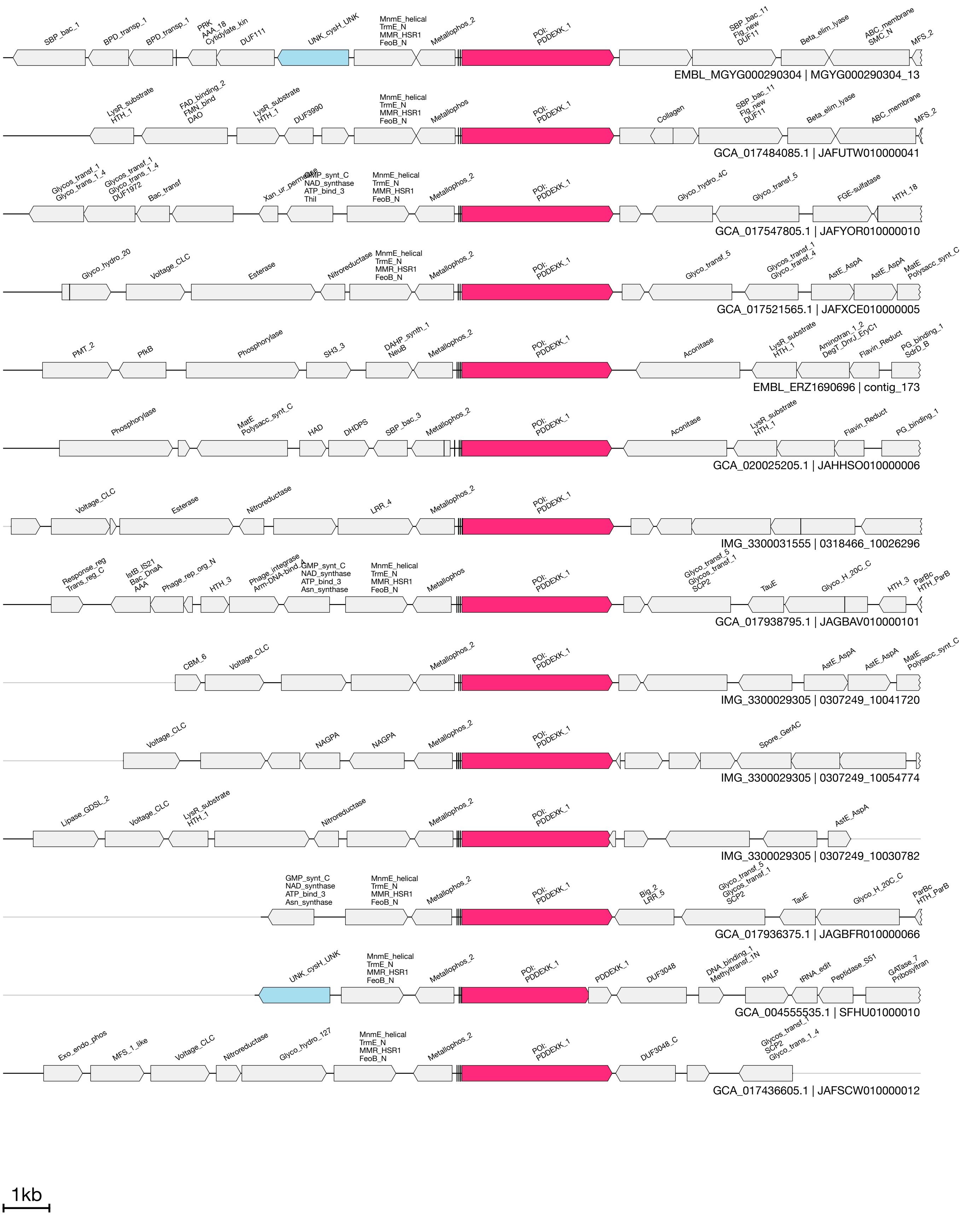

GL

(PDDEXK_ATPase)

37 / 39.4

Non-CRISPR_hypervariable_repeat EMBL_ERZ842383&&contig_35002&&108_1647_-1

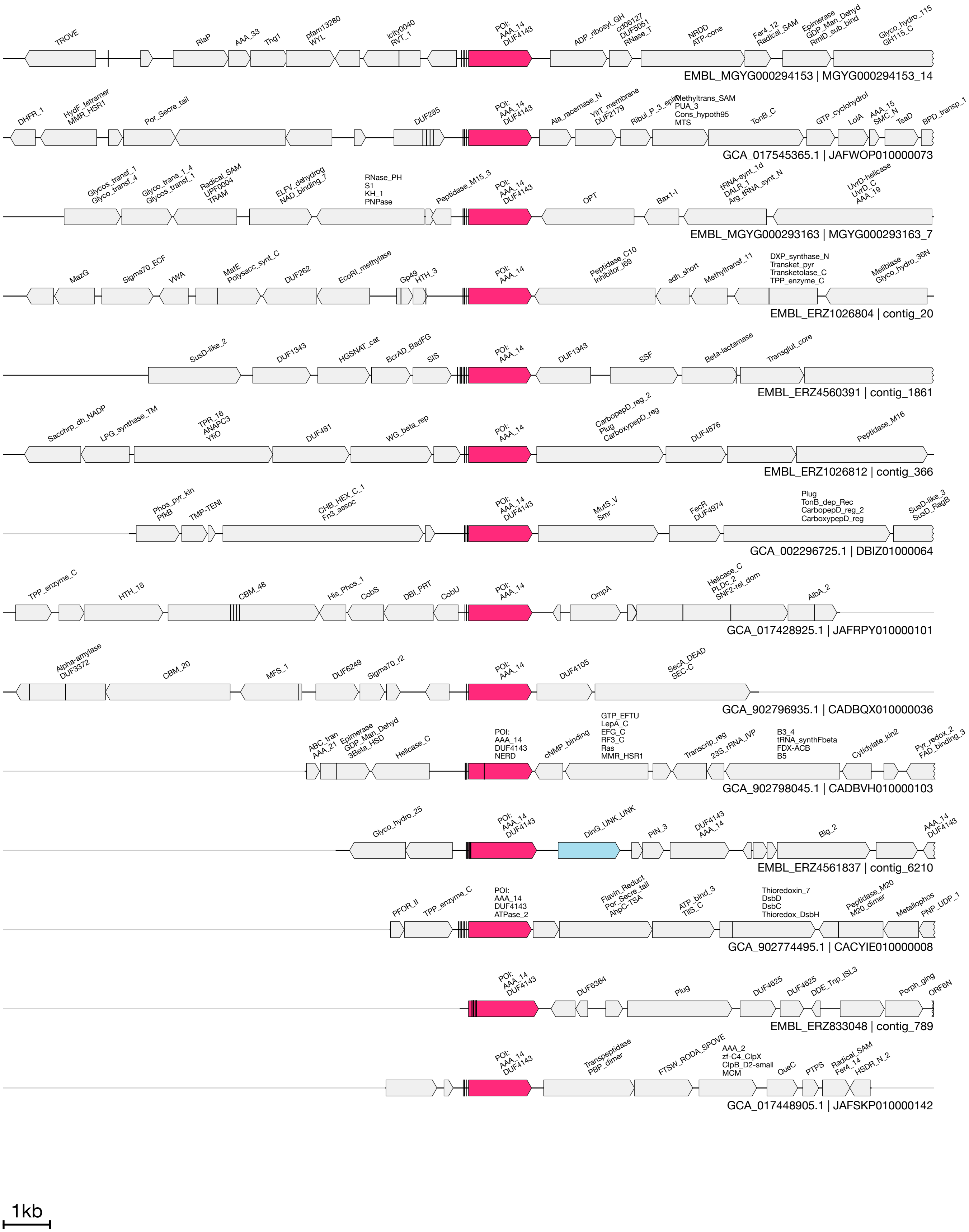

GM

(PLMP_corA-like) 37 / 48.3

Non-CRISPR_hypervariable_repeat GCA_014804555.1&&JAAUZO010000014&&4635_6039_-1

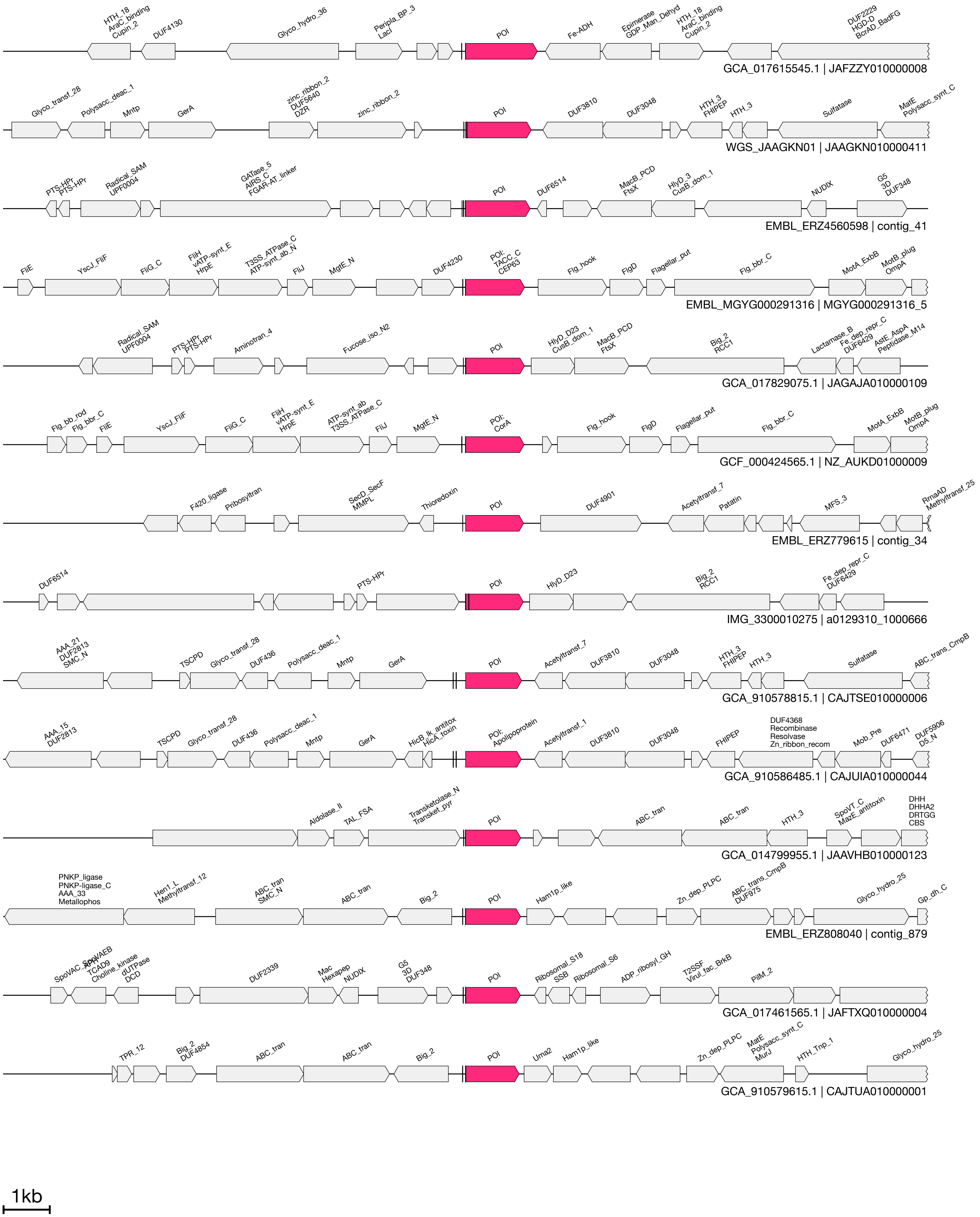

GN

(DUF222_HNH) 51 / 111.4

Non-CRISPR_hypervariable_repeat GCF_000442645.1&&NC_021915&&2150743_2152009_1

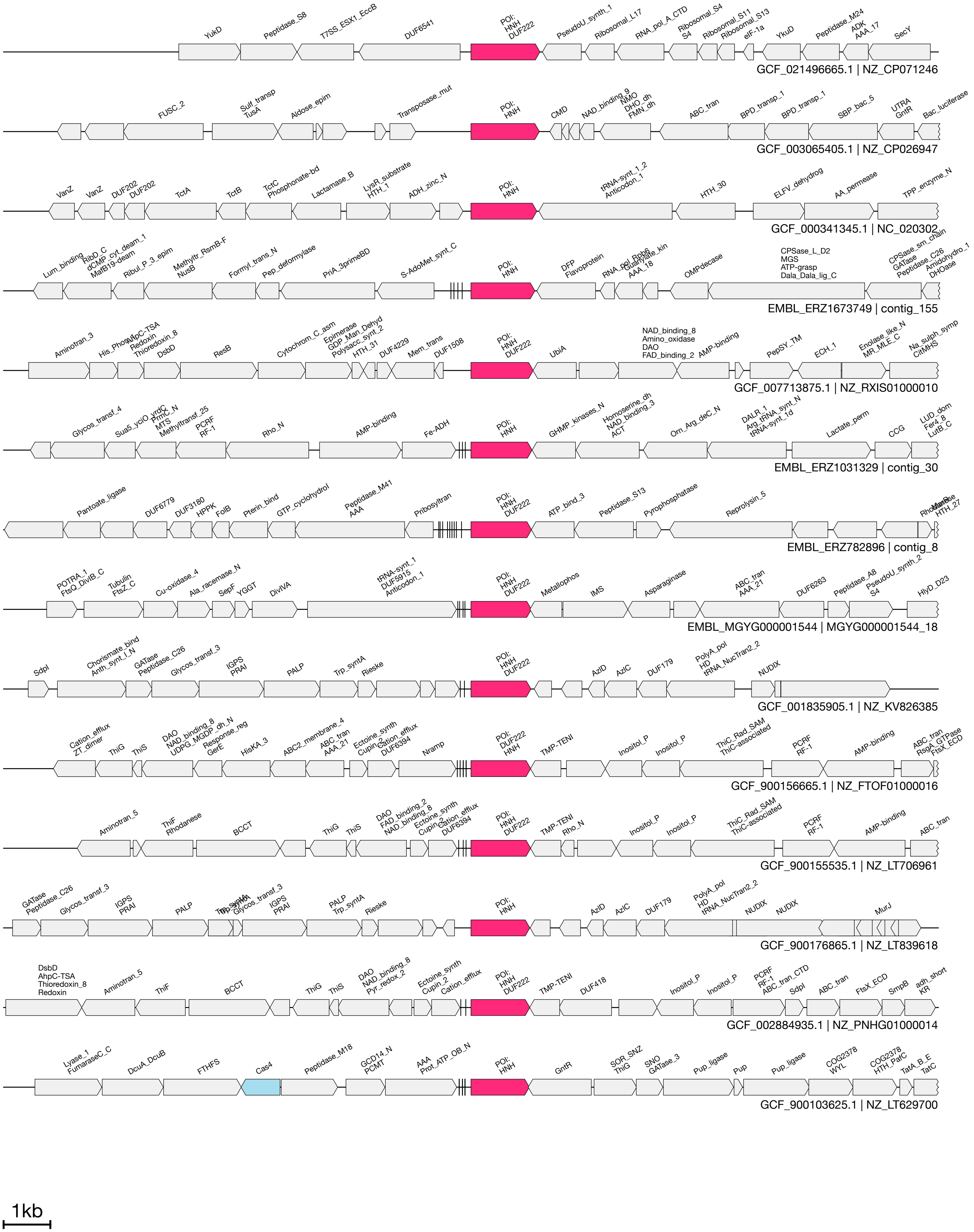

GO

(GGDEF + MFS + Phospholipase)

25 / 35.5

Non-CRISPR_hypervariable_repeatGCF_002262875.1&&NZ_NKYE01000003&&91808_93059_-1

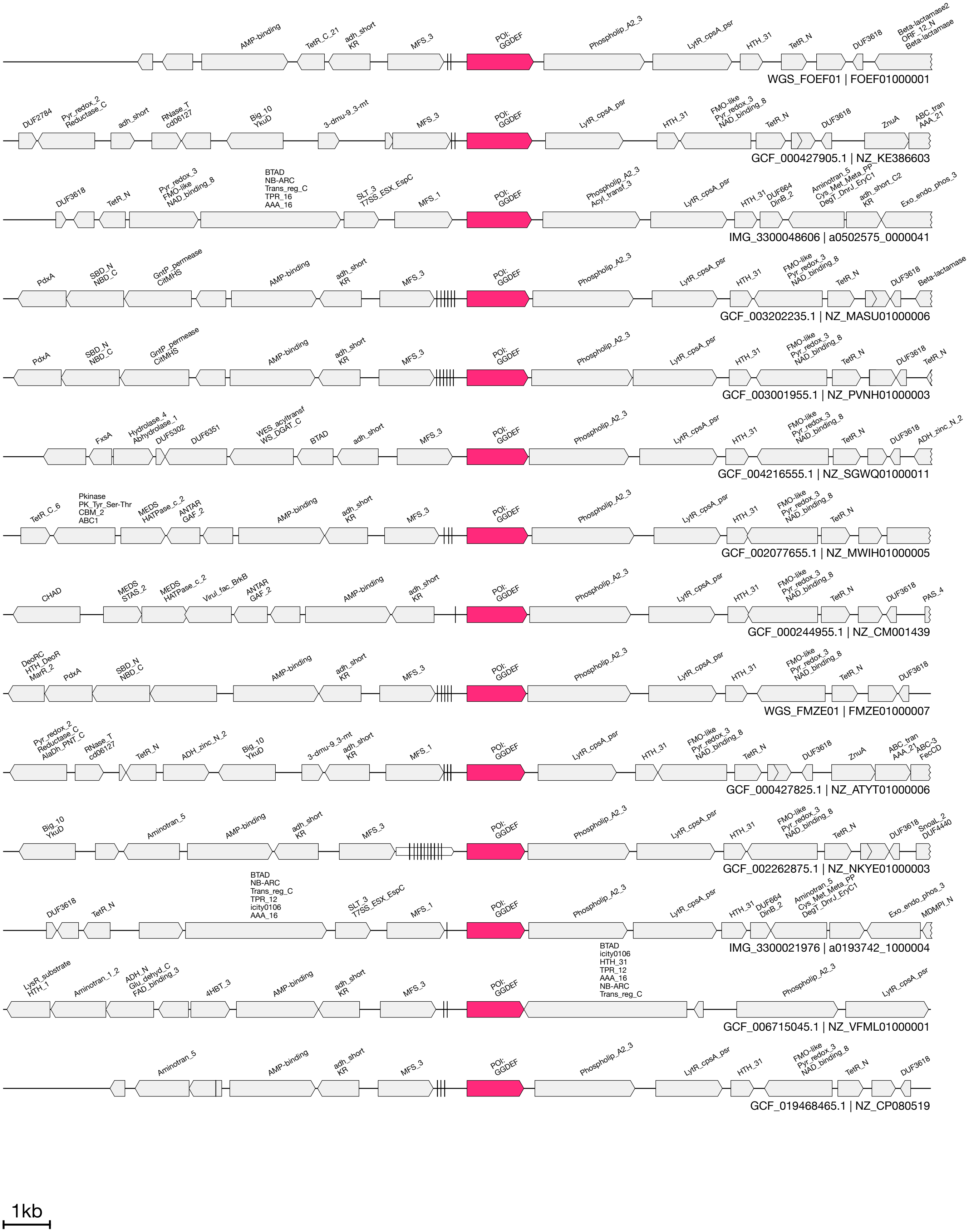

Fig. S15.

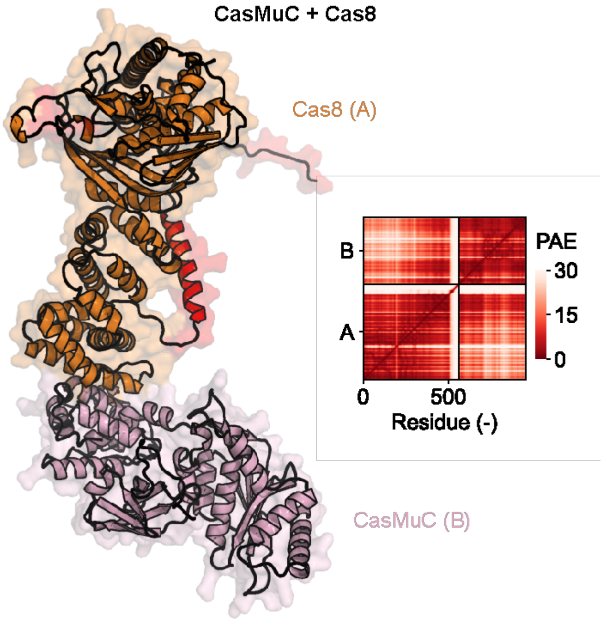

Fig. S15. AlphaFold2 predictions of CasMu-I system

AlphaFold2-multimer structural prediction of Cas8 interaction with CasMuC. PAE plot shown on the right.

Fig. S16.

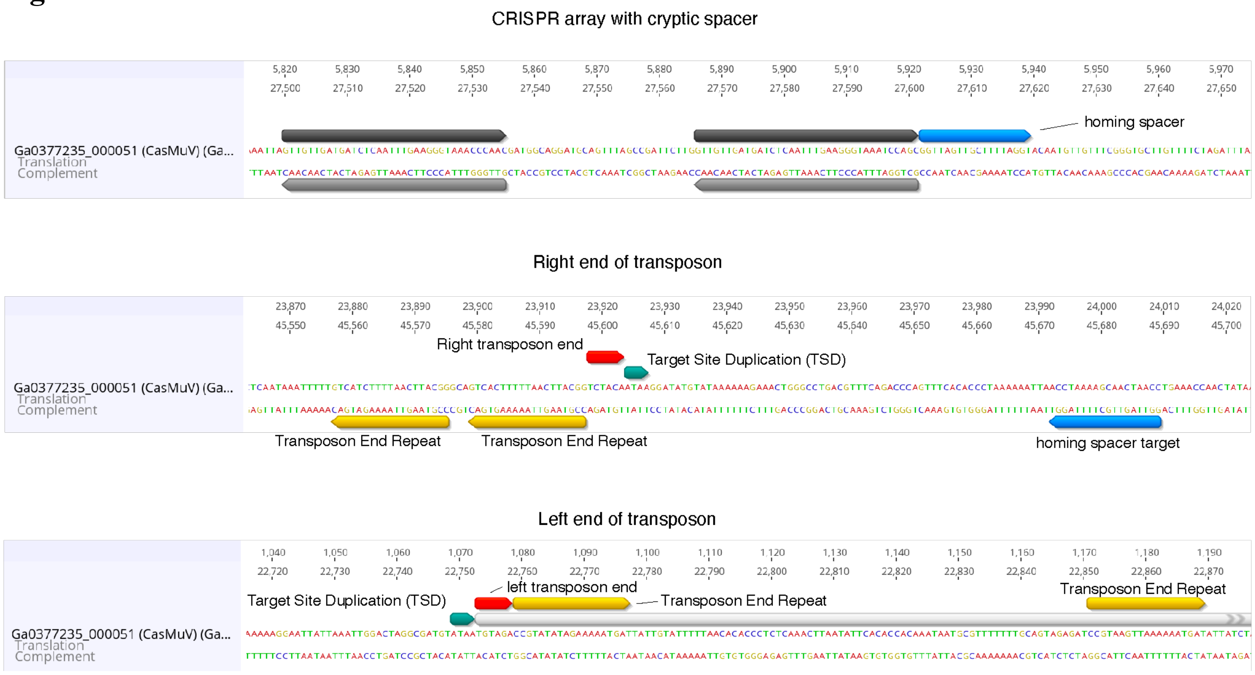

Fig. S16. CasMu-V Transposon ends and homing spacer

CasMu-V CRISPR array along with a cryptic spacer outside of the array is shown along with the spacer's target site 68 bp downstream from the right end of the transposon. The transposon end repeats are present in copies of two, and the exact boundary of the transposon contains the motif TGT which is consistent with Mu-like transposon ends. A target site duplication of length 4 outside of the transposon is detectable as well.

Fig. S17.

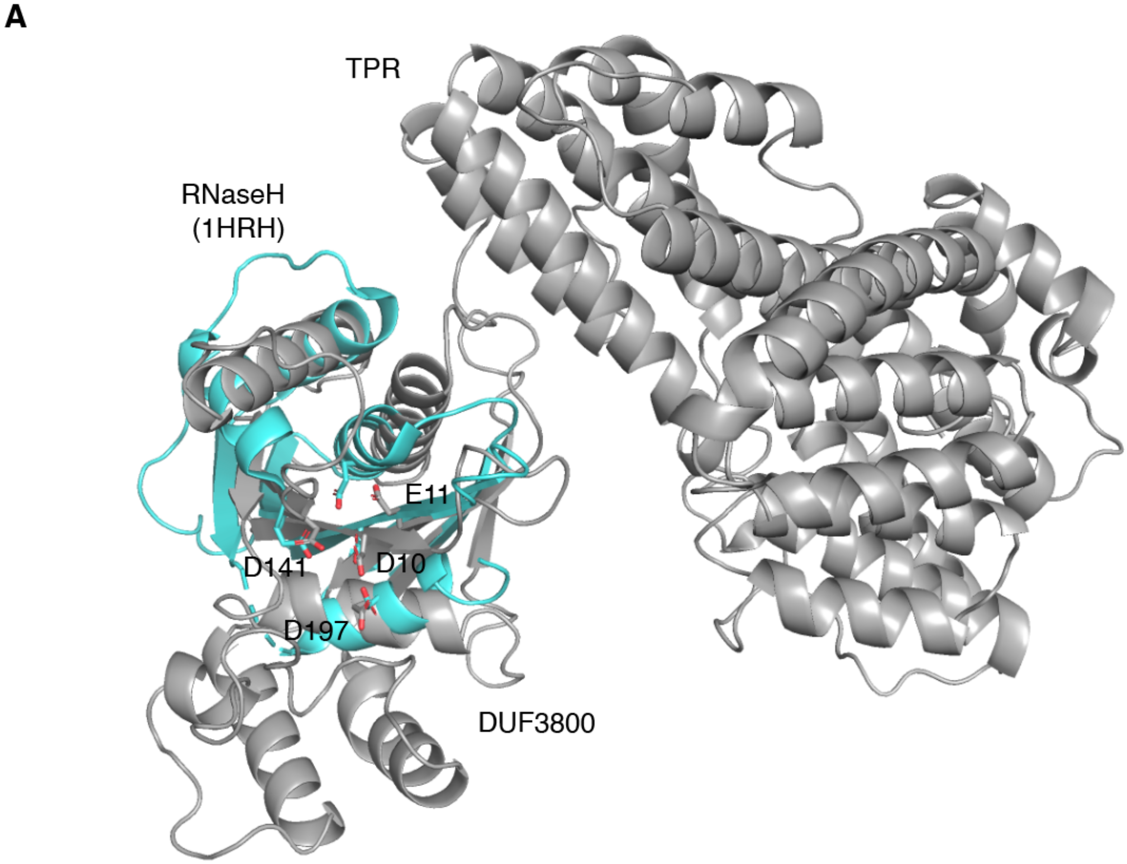

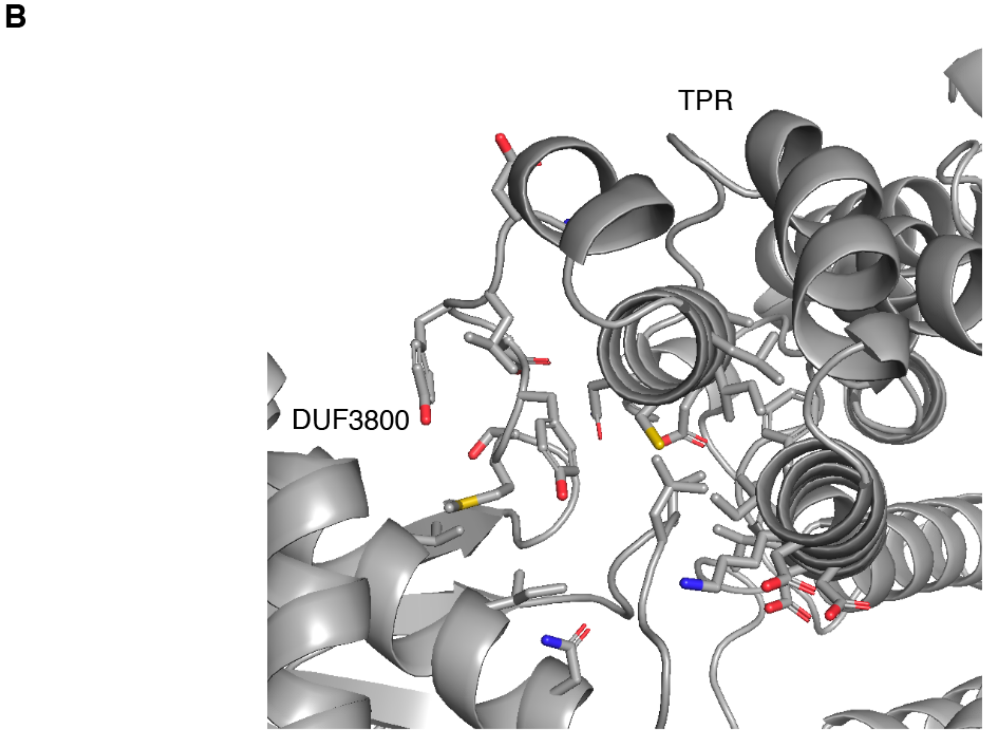

Fig. S17. Structural comparison of DUF3800-TPR protein

(A) The structure DUF3800-TPR protein from the Cas12a2 associated system was predicted using AlphaFold2 and superimposed with the RNaseH domain from the HIV reverse transcriptase domain (PDH: 1HRH). All catalytic residues from the RNaseH fold was found in the DUF3800 protein, barring one catalytic rearrangement (E11), which takes place of E478 from the HIV reverse transcriptase RNaseH domain. The DUF3800 domain contains an additional segment of alpha helices that are not found in the typical RNaseH fold. **(B)** The TPR domain is apparently connected to the DUF3800 protein via a linker but makes multiple contacts with the DUF3800 domain.

Fig. S18.

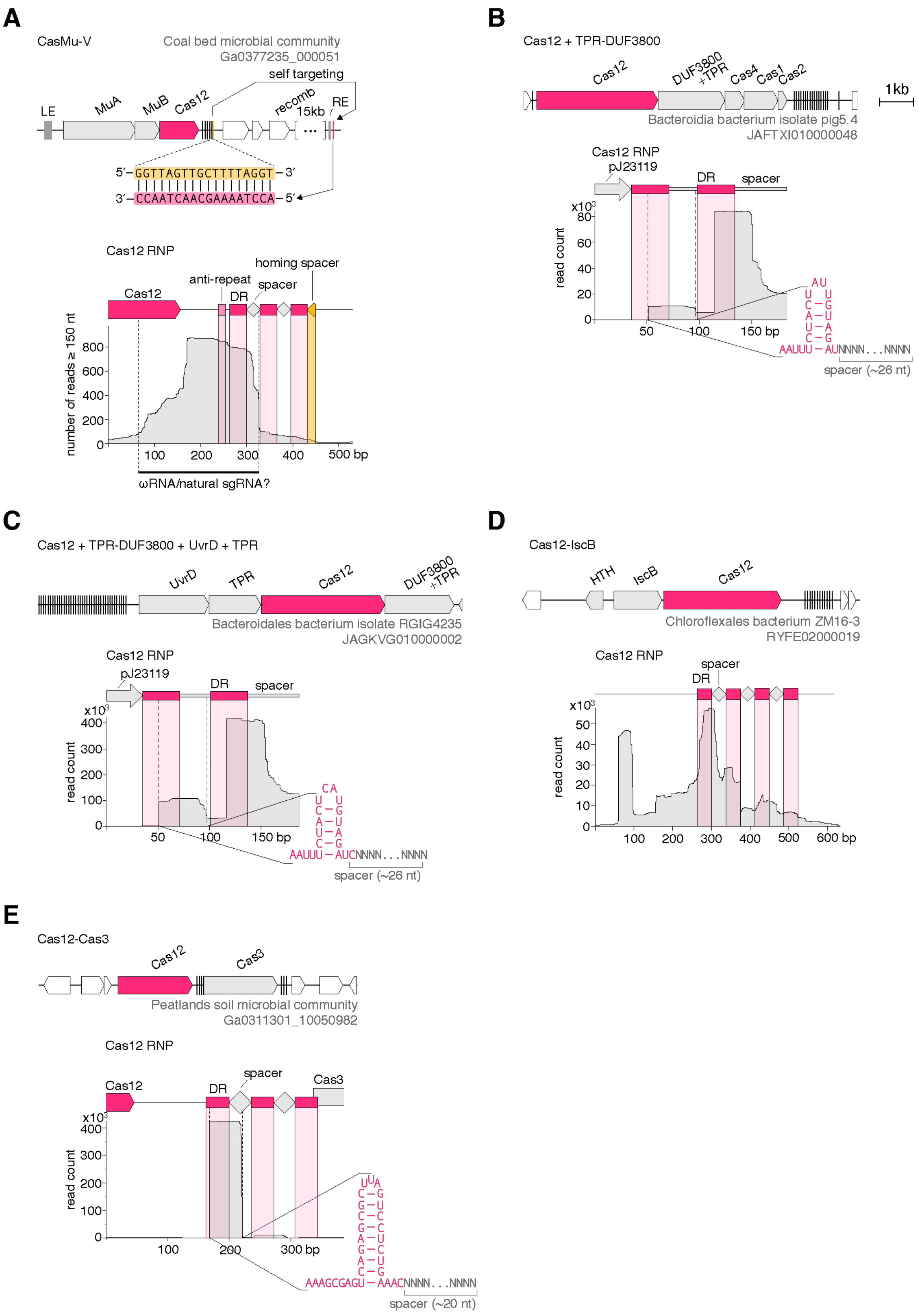

A
CasMu-V
Coal bed microbial community
Ga0377235_000051
self targeting
LE
MuA
MuB
Cas12
recomb
15kb
RE
5′–GGTTAGTTGCTTTTAGGT–3′
3′–CCAATCAACGAAAATCCA–5′
Cas12 RNP
homing spacer
anti-repeat
spacer
DR
number of reads ≥ 150 nt
ωRNA/natural sgRNA?
B
Cas12 + TPR-DUF3800
DUF3800 +TPR
Cas4
Cas1
Cas2
1kb
Bacteroidia bacterium isolate pig5.4
JAFTXI010000048
Cas12 RNP
pJ23119
read count
spacer (~26 nt)
C
Cas12 + TPR-DUF3800 + UvrD + TPR
UvrD
TPR
Bacteroidales bacterium isolate RGIG4235
JAGKVG010000002
D
Cas12-IscB
HTH
IscB
Chloroflexales bacterium ZM16-3
RYFE02000019
E
Cas12-Cas3
Cas3
Peatlands soil microbial community
Ga0311301_10050982
spacer (~20 nt)

Fig. S18. Small RNA-seq of Cas12 RNPs from new Type-V associated systems

Small RNA-seq of affinity purified Cas12 RNPs from **(A)** CasMu-V, **(B)** Cas12 + TPR-DUF3800, **(C)** Cas12 + TPR-DUF3800 + UvrD + TPR, **(D)** Cas12-IscB and **(E)** Cas12-Cas3. Cas12s from (B) and (C) were co-expressed with a CRISPR array expressed under a pJ23119 promoter, and all other Cas12s were co-expressed with the full locus encoding the system, with RNA components under control of natural promoters in the locus. All Cas12s co-purified with ncRNA overlapping with the predicted CRISPR array, as shown.

Fig. S19.

A

Template alignment | Template 3D Structure | PDBe

1. **6KC8_A CRISPR-associated endonuclease Cas9; CRISPR-Cas9, NmeCas9, Nme1Cas9, hydrolase, ternary complex, HYDROLASE-RNA-DNA complex; 2.9A {Neisseria meningitidis 8013}**

Probability: 100%, E-value: 1.5e-134, Score: 1351.84, Aligned cols: 977, Identities: 25%, Similarity: 0.372, Template Neff: 7.7

```
Q ss_pred              CCCCeEEEEEeCCCeeeeEEeecccCCCCccceecceeeecCCccccccccCCCCCHHHHHHHHHHHHHHHHHHHHHHH
Q Q_6839663_1   1      MKPKRILGLDLGTNSIGWALVEESASIEQPSKIVKLGVRVNPLTTDEKNNFEAGKPLSTNADRTTKRGARRNLQRYKLRR   80 (1385)
Q Consensus     1      ~~mk~iLGLDlGt~SIGWAvi~~d~~~~~~~~~I~~~GvriF~~~~~~~~~~~~~~g~~~s~a~~RR~~R~~RR~~~Rr~~R~   80 (1385)
                       +||+++|||||||||||||||++++++ .|..|+++||||||.+.+++    +|  .|+|++||.+|++||+++||++|+
T Consensus     8      ~~~~~~LGLDlG~~SiGwAvi~~d~~~~~~~~Ii~~GvriF~~~~~~k---------s~~~~RR~~R~~RR~~rRr~~R~   80 (1083)
T 6KC8_A        8      NSINYILGLDIGIASVGWAMVEIDEEE-NPIRLIDLGVRVFERAEVPK----TG--DSLAMARRLARSVRRLTRRRAHRL   80 (1083)
T ss_dssp               -CCCEEEEEEEECSSEEEEEEEEEECTTC-CEEEEEEEEEEECCCCCCC------C--CCHHHHHHHHHHHHHHHHHHHHHHHH
T ss_pred               cCCCceEEEEEeCCCeeEEEEEEEeCCCC-CeeEEEEcceEEecccCCCC----CC--CCHHHHHHHHHHHHHHHHHHHHHHHH
```

```
Q ss_pred              CCCeEEEEeccccCCHHHHHHHHHHHHHHHHHHHHHHHHHHHHHHhC--CCCCCHHHHHHHHHHHHHhccCCccccCCCCCcc
Q Q_6839663_1   701    KPDKVRIELARELKKSAQEREKMTKQINAATKQNEEYRKILTKEFG--IPNPTRNDVIRYRLWLELENNAFKDIFSNKQI   778 (1385)
Q Consensus     701    ~P~~I~IElAReLk~s~~~R~~i~k~q~~n~k~ne~~~~~l~e~~g----------i~KyrLw~Eq~~~~~~C~YtG~~I   778 (1385)
                       .|+.|+||||||+++|.++|+++.+.|++|+++++++.+.|.+.++  ...++..++.|++||.+|++   +|||||++|
T Consensus     498    ~p~~I~IElaRe~~~s~~~R~~i~k~q~~n~~~~~~~~~~~l~~~~~~~~~~~~~~~~~k~~L~~~q~~----C~ycg~~i   574 (1083)
T 6KC8_A        498    SPARIHIETAREVGKSFKDRKEIEKRQEENRKDREKAAAKFREYFPNFVGEPKSKDILKLRLYEQQHG---KCLYSGKEI   574 (1083)
T ss_dssp               SCSEEEEEESCCCCCHHHHHHHHHHHHHHHHHHHHHHHHHHHHHHHHC--------CHHHHHHHHHTTTTT---BCTTTCCBC
T ss_pred               CCCeEEeeeHhhcCCCHHHHHHHHHHHHHHHHHHHHHHHHHHHHHHHCcccCCCCChHHHHHHHHHHHhcC---ccCCCCCCC
```

```
Q ss_pred              CHHHccCCC-CcccccccccccCCCccccceEEEEhhhhHHHccccchhHHhccc-ccccHHHHHHHHHHHHcCCCCCHHHH
Q Q_6839663_1   779    KKEDLFSSK-IDIEHIIPKALLYDDSFSNKTLAFKDINLKKANRTGYDFISED-YHENLDDYVQRVEMLYNGGAISRAKK   856 (1385)
Q Consensus     779    ~~~~L~~~~~~eIDHIiP~S~s~DdS~~NkvL~~~~~~N~~KgnrtP~e~~~~~~~~~~~~~~~~~~~rv~~l~~~~~~~~~~~~K~   856 (1385)
                       ++.+|+++. ++||||+|+|.++|||++|+|||..||+.|+++||++|+...  ....|++|.++|..+    .++..|+
T Consensus     575    ~~~~l~~~~~~~eidHIiP~s~~~dds~~Nlvl~~~~cN~~K~~~~p~e~~~~~~~~~~~w~~~~~~v~~~~~~~~~~~~~~~K~   650 (1083)
T 6KC8_A        575    NLGRLNEKGYVEIDHALPFSRTWDDSFNNKVLVLGSENQNKGNQTPYEYFNGKDNSREWQEFKARVETS----RFPRSKK   650 (1083)
T ss_dssp               CSCCSSSTTSEEEEESSCTTTSCCCSTTSEEEEETHHHHTTSSCCHHHHTTTTSCCHHHHHHHHHHHTC----CSCHHHH
T ss_pred               CHHHhcCCCCceEEEEEeecccCCCCChhheeEhhhhhhhcCCCCHHHhcCCCCchHHHHHHHHHHHc----CCCHHHH
```

```
Q ss_pred              CceeeeecccCCCceeeceecCCCcccccchhHHHHHHHHHHHcCHHHHHHHHHHHhhccccccccccccHhcC-----cc
Q Q_6839663_1   930    LTQKEQRFDIGRGETKEVEVITDWTKRNDHRHHAMDALTVAFTKLNHTQYLNRVNTANDDSIDNEKIIERVNG-----KK   1004 (1385)
Q Consensus     930    ~~~~~~~~~~~~~~~~~~~~~~~~~~K~R~dhrHHAiDA~viA~~~~~~~iq~l~~~~~~~~~~~~~~~~~~~~~~~~~~~~~~~   1004 (1385)
                                                +.+.||+|||+||+||||++++++++++.+....+..........+.+      ..
T Consensus     716    -------------------------~~~~d~rHHA~DA~viA~~~~~~~~~~~~~l~~~~~~~~~~~~~~~~~~~~~ge~~~~~~   771 (1083)
T 6KC8_A        716    ------------------------VRAENDRHHALDAVVVACSTVAMQQKITRFVRYKEMNAFDGKTIDKETGEVLHQKT   771 (1083)
T ss_dssp               ------------------------CSTTCTTHHHHHHHHHHHCCHHHHHHHHHHHHHHSCCCTTTCEECC--CCCCEETTT
T ss_pred               ------------------------ccccccchHHHHHHHHHhcCHHHHHHHHHHHHHHHHHHhccCCCcccccCCchhhcc
```

Fig. S19. (cont'd)

B

Template alignment | Template 3D Structure | PDBe

1. **6KC8_A CRISPR-associated endonuclease Cas9; CRISPR-Cas9, NmeCas9, Nme1Cas9, hydrolase, ternary complex, HYDROLASE-RNA-DNA comple› 2.9A {Neisseria meningitidis 8013}**

Probability: 100%, E-value: 1.4e-128, Score: 1225.89, Aligned cols: 852, Identities: 18%, Similarity: 0.216, Template Neff: 7.7

```
Q ss_pred               CEEEEEeCCCeeEEEEEeCc------ceeccEeEEeccCcCccccchhcCCCCCHHHHHHHHHHHHHHHHHHHHHHHHHH
Q Q_6839663      1      MRLNIYCGLRAIGWIVTNGN------KAVQYGIKRLNISFDNYYEYIAGLPVSKRINRRLKRGSRKNLHRYKRRREKLIN   74 (889)
Q Consensus      1      y~LGLDlG~~SIGwAvi~~~------~i~~~GvriF~~~~~~~~~~~~g~~~s~~~~RR~~R~~RR~~~Rr~~R~~~l~~   74 (889)
                        |+|||||+|||||+|++|      +|+++||||||.+.+|+    +  +.|++++||.+|++||+++||++|+..|.+
T Consensus     12      ~~LGLDlG~~SiGwAvi~~d~~~~~~~Ii~~GvriF~~~~~~k----~----~s~~~~RR~~R~~RR~~rRr~~R~~~l~~   85 (1083)
T 6KC8_A        12      YILGLDIGIASVGWAMVEIDEEENPIRLIDLGVRVFERAEVPK----T--GDSLAMARRLARSVRRLTRRRAHRLLRTRR   85 (1083)
T ss_dssp               EEEEEEECSSEEEEEEEEECTTCCEEEEEEEEEEECCCCCCC--------CCCHHHHHHHHHHHHHHHHHHHHHHHHHHH
T ss_pred               eEEEEEeCCCeeEEEEEEeCCCCCeeEEEEcceEEecccCCCC----C--CCCHHHHHHHHHHHHHHHHHHHHHHHHHHH

Q ss_pred               HHHHHHHHHHHHHHHcC-CCeEEEEeccccCCHHHHHHHHHHHHHHHHHHHHHHHHHHHhc--------CCCHHHHHHHHHHH
Q Q_6839663    403      YYAMQSLVERLKQKYP-IEEMNFEIDMLLKMGNKARKQLSSNRRKDLALKKQYAKELE--------GKTEYEFQKLKAWL  473 (889)
Q Consensus    403      l~q~rkvvn~iik~yG-P~~I~IElaRel~~s~~~r~~i~~~~~~n~k~~~~~~~~~l~--------~~~~~~i~K~~Lw~  473 (889)
                        |+|+|+|||+|+++|| |+.|+||||||++++.++|+++.+.|++|+++++++.+.|.        .+++.++.|++||.
T Consensus    482      l~q~rkvvn~ii~~yG~p~~I~IElaRe~~~s~~~R~~i~k~q~~n~~~~~~~~~~l~~~~~~~~~~~~~~~~~~~k~~L~~  561 (1083)
T 6KC8_A       482      LSQARKVINGVVRRYGSPARIHIETAREVGKSFKDRKEIEKRQEENRKDREKAAAKFREYFPNFVGEPKSKDILKLRLYE  561 (1083)
T ss_dssp               HHHHHHHHHHHHHHHHCSCSEEEEEESCCCCCCHHHHHHHHHHHHHHHHHHHHHHHHHHHHHHHC--------CHHHHHHHHHT
T ss_pred               HHHHHHHHHHHHHHHHCCCCeEEeeeHhhcCCCHHHHHHHHHHHHHHHHHHHHHHHHHHHHHHCcccCCCCChHHHHHHHHHH

Q ss_pred               HhcCCCCCcCCCCCCCCCCCeeCHHHHcCCC-CccceecccccCCCcccceEEEeHHHHHHHcCCCHHHHHhhc--c-cH
Q Q_6839663    474      ELKGNKQDQTAVSPLEPDVEITLEMALSDK-YNFDHIVPKSQLFERGQNNLILCRKSVNEDKGKKTAYDYAKEK--G-FL  549 (889)
Q Consensus    474      eQ~~~~~~~~~~~C~~YtG~~I~~~~L~~~~~-eIDHIiP~S~s~DdS~~NkvL~~~~~~N~~KgnrtP~e~~~~~~~~~~--~w  549 (889)
                        +|++       +| +|||++|+.++|+++. ++||||+|+|.++|||++|+||||..||+.||++||++|+...  . .|
T Consensus    562      ~q~~-------~~C--~ycg~~i~~~~~l~~~~~~~eidHIiP~s~~~dds~~Nlvl~~~~cN~~K~~~~p~e~~~~~~~~~w  633 (1083)
T 6KC8_A       562      QQHG-------KC-LYSGKEINLGRLNEKGYVEIDHALPFSRTWDDSFNNKVLVLGSENQNKGNQTPYEYFNGKDNSREW  633 (1083)
T ss_dssp               TTTT-------BC-TTTCCBCCSCCSSSTTSEEEEESSCTTTSCCCSTTSEEEEETHHHHTTSSCCHHHHTTTTSCCHHH
T ss_pred               HhcC-------cc-CCCCCCCCHHHhcCCCceEEEEEeecccCCCCChhheeEhhhhhhhhcCCCCHHHhcCCCCchhH

Q ss_pred               chhhhccCCCCChhHHHHHHHHHHhCCHHHHHHHHHHHHHHHhcCCccCCcccccCC-C-----CCCcCCCccc-------
Q Q_6839663    612      REWLKNLYSSDDARYALQKAWVLSNFTQGDLEQLDNIKEVKQNPYGKNPNMELIDF-E-----KSPIFLPRIK-------  678 (889)
Q Consensus    612      l~~~~~~k~r~d~rHHAiDA~via~~~~~~~~~~~l~~~~~~~~~~~~~~~~~~~~~~~~~~~~~~~~~~~~~~~~~pw-------  678 (889)
                        +    .++.+.||+|||+||+||||++++++++++.+....+...   .....  .+. .      ....+++||.
T Consensus    713      l----k~~~~d~rHHA~DA~viA~~~~~~~~~~~l~~~~~~~~~~~~~~~~~~~~~~~~~~~ge~~~~~~~~~~~~pw~~f~~~v~  785 (1083)
T 6KC8_A       713      L----RKVRAENDRHHALDAVVVACSTVAMQQKITRFVRYKEMNA-FDGKT--IDKETGEVLHQKTHFPQPWEFFAQEVM  785 (1083)
T ss_dssp               C----CCCSTTCTTHHHHHHHHHHHCCHHHHHHHHHHHHHHSCCCT-TTCEE--CC--CCCCEETTTTSCCSSSSHHHHHH
T ss_pred               C----cccccccccchHHHHHHHHHhcCHHHHHHHHHHHHHHHHHHHHHhc-cCCCc--cccccCCchhhccCCCCCCCccHHHHHH
```

Fig. S19. (cont'd)

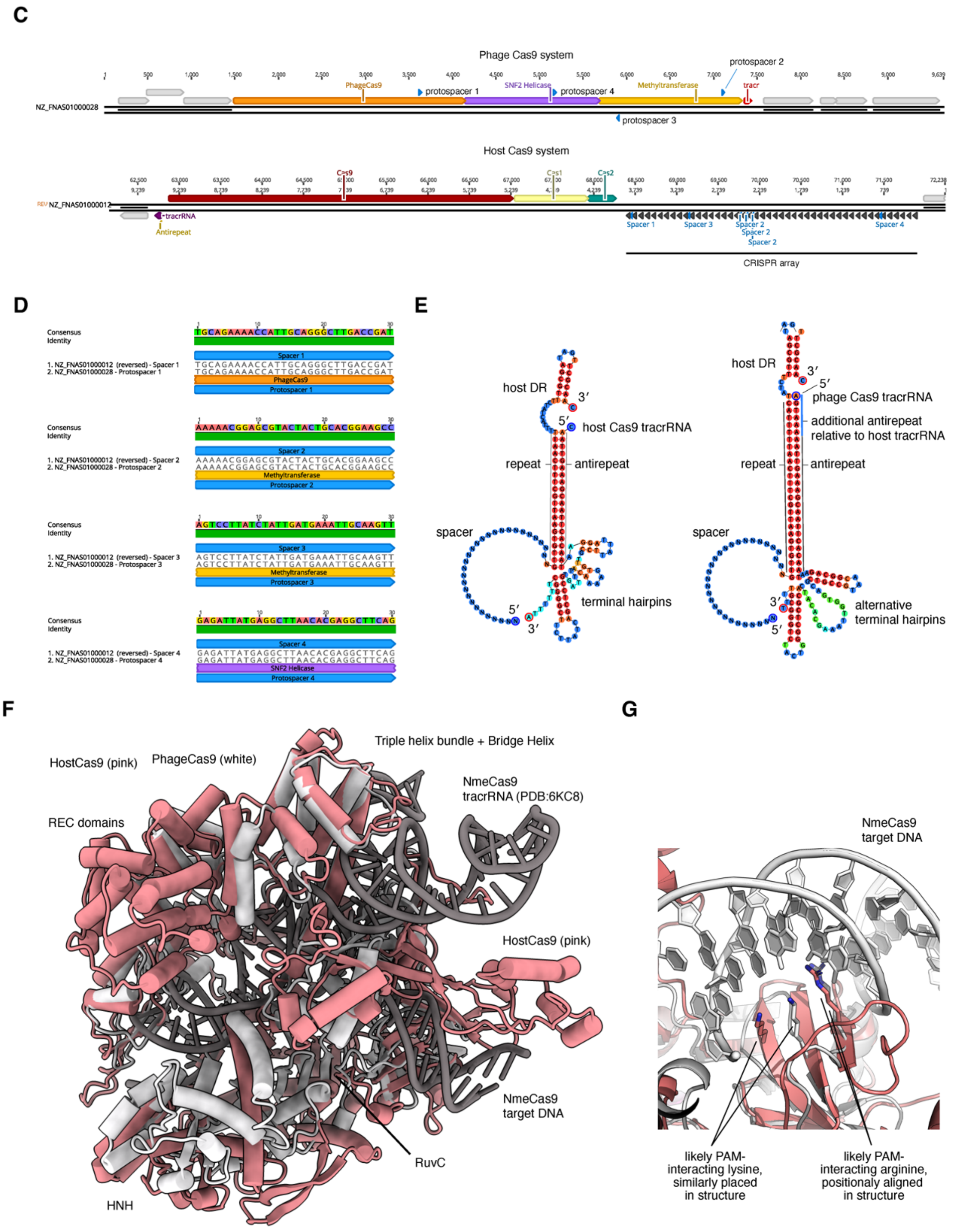

Fig. S19. PhageCas9 compared to a corresponding Cas9 in the host organism

(A) HHpred of the phage Cas9 protein from *Riemerella columbipharyngis* strain DSM 24015-infecting phage along with the catalytic sites shown with black arrows. The RuvC-I and RuvC-III domains are inactivated. **(B)** HHpred of the host Cas9 protein from *Riemerella*

columbipharyngis strain DSM 24015 along with the catalytic sites shown with arrows. All catalytic residues are present. **(C)** Gene locus architectures of the phage Cas9 along with the corresponding host Cas9. The phage Cas9 system has a PhageCas9 along with a SNF2 helicase, a potentially linked methyltransferase, followed by a tracrRNA. 6 CRISPR spacers from the host Cas9 system (with 4 unique sequences) have matches directly to the Phage Cas9 system. **(D)** Matching spacers along with their protospacer sequences in the phage Cas9 system. All hits are exact matches, indicating ongoing conflict between the two systems. **(E)** Comparison of the tracrRNAs from the host Cas9 and the phage Cas9 as they anneal to the host CRISPR direct repeats. Secondary structure predictions were performed with RNAFold from the Vienna RNA package at an annealing temperature of 37°C. **(F)** AlphaFold2 predictions and structural comparison of the phage Cas9 and the host Cas9, which is substantially larger. The two Cas9s are superimposed onto the RNA components from the structure of NmeCas9 (PDB: 6KC8) with the NmeCas9 protein not shown.

Fig. S20.

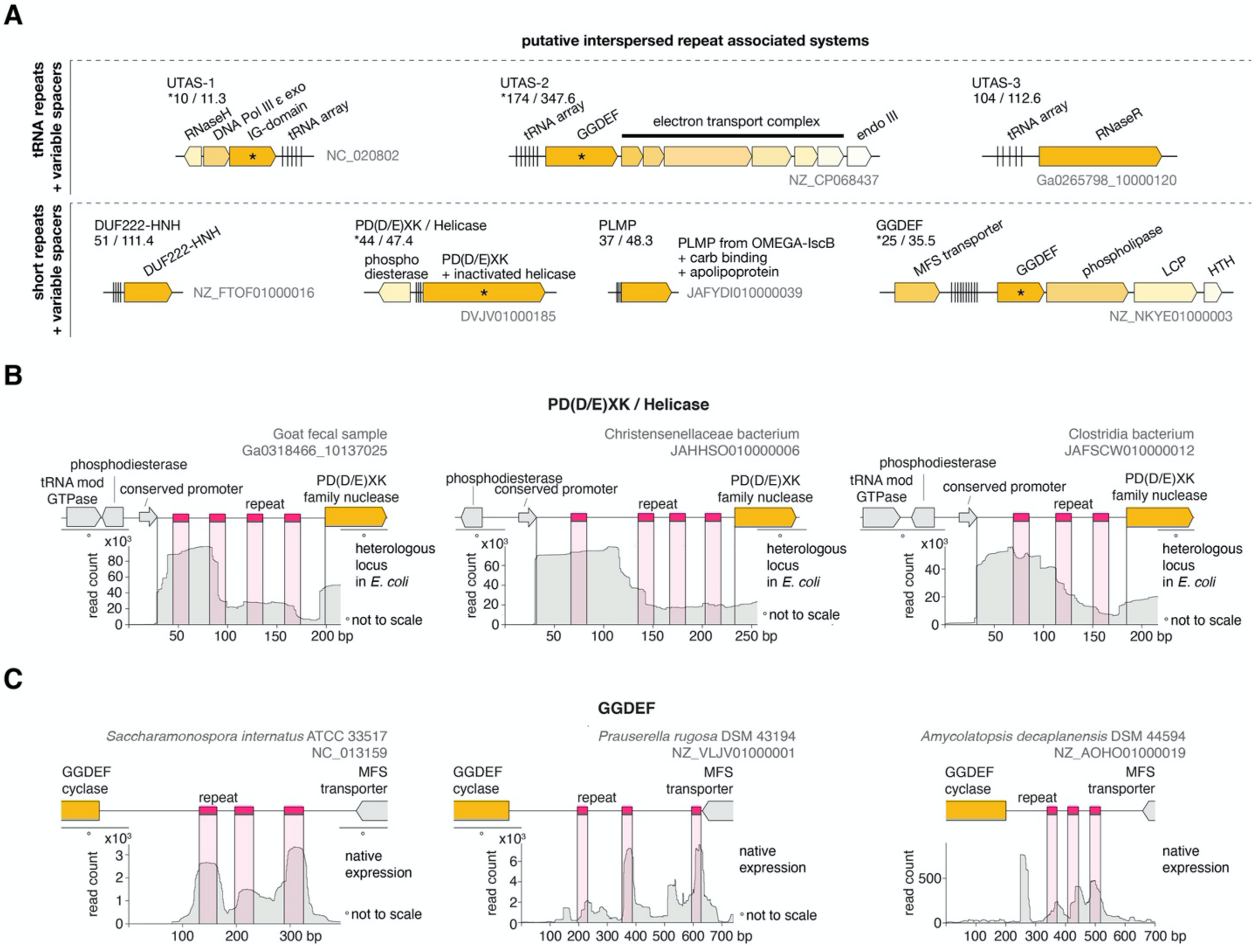

Fig. S20. Additional interspaced repeat array systems

(A) Systems identified in this study associated with repeat arrays interspaced with hypervariable sequences with no association to known CRISPR-Cas genes. All enhanced CRISPR association scores are shown below the system name as determined by the pipeline with the numerator corresponding to the number of repeat-associated loci in a given cluster and the denominator being the effective sample size of that cluster. * denotes the gene for which the score is calculated where multiple genes are present in the system. **(B)** Small RNA-seq of *E. coli* heterologously expressing PD(D/E)XK loci from plasmids. **(C)** Small RNA-seq of native organisms harboring instances of the GGDEF system.

Fig. S21.

A

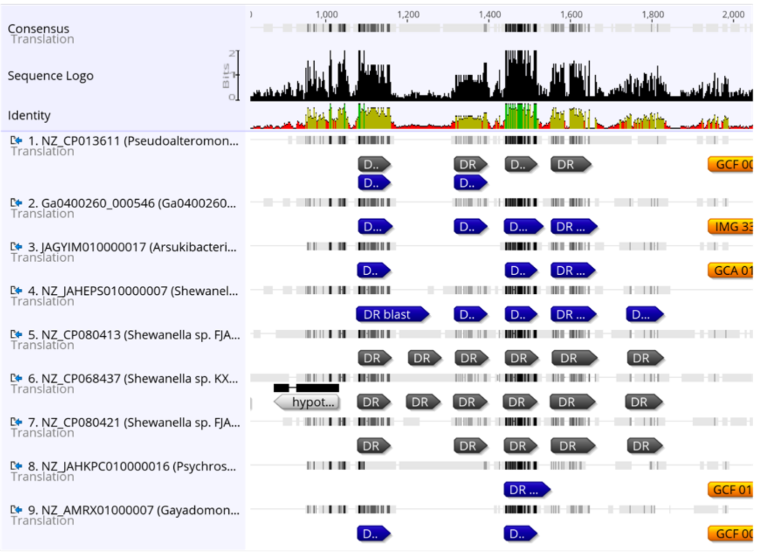

Consensus
Translation
Sequence Logo
Identity
1,000
1,200
1,400
1,600
1,800
2,000
1. NZ_CP013611 (Pseudoalteromon...
2. Ga0400260_000546 (Ga0400260...
3. JAGYIM010000017 (Arsukibacteri...
4. NZ_JAHEPS010000007 (Shewanel...
5. NZ_CP080413 (Shewanella sp. FJA...
6. NZ_CP068437 (Shewanella sp. KX...
7. NZ_CP080421 (Shewanella sp. FJA...
8. NZ_JAHKPC010000016 (Psychros...
9. NZ_AMRX01000007 (Gayadomon...
DR blast
hypot...

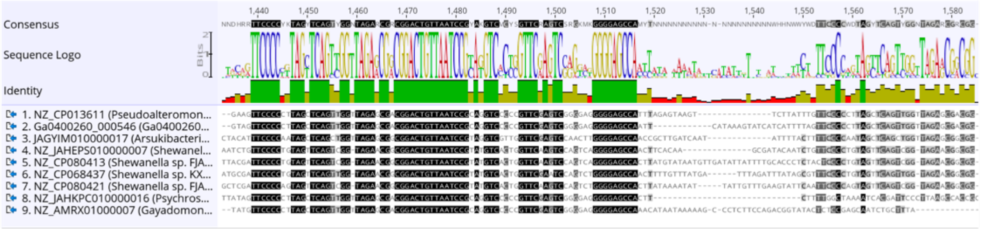

Consensus
Sequence Logo
Identity
1,440
1,450
1,460
1,470
1,480
1,490
1,500
1,510
1,520
1,530
1,540
1,550
1,560
1,570
1,580
1. NZ_CP013611 (Pseudoalteromon...
2. Ga0400260_000546 (Ga0400260...
3. JAGYIM010000017 (Arsukibacteri...
4. NZ_JAHEPS010000007 (Shewanel...
5. NZ_CP080413 (Shewanella sp. FJA...
6. NZ_CP068437 (Shewanella sp. KX...
7. NZ_CP080421 (Shewanella sp. FJA...
8. NZ_JAHKPC010000016 (Psychros...
9. NZ_AMRX01000007 (Gayadomon...

Fig. S21. (cont'd)

B

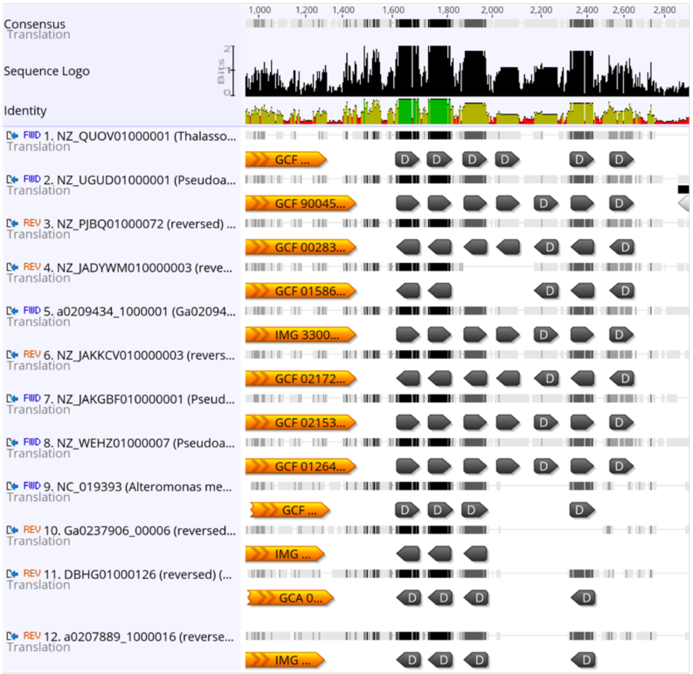

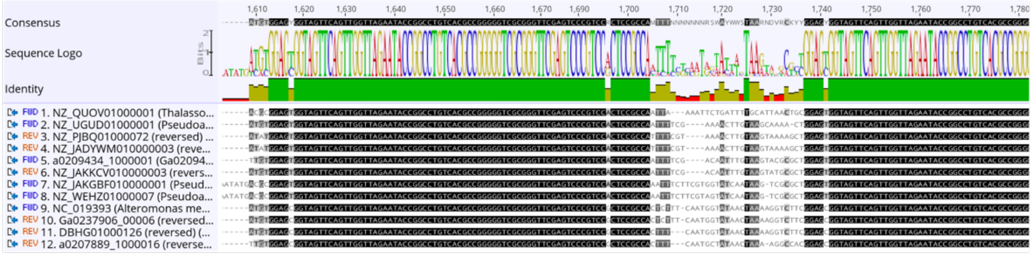

C

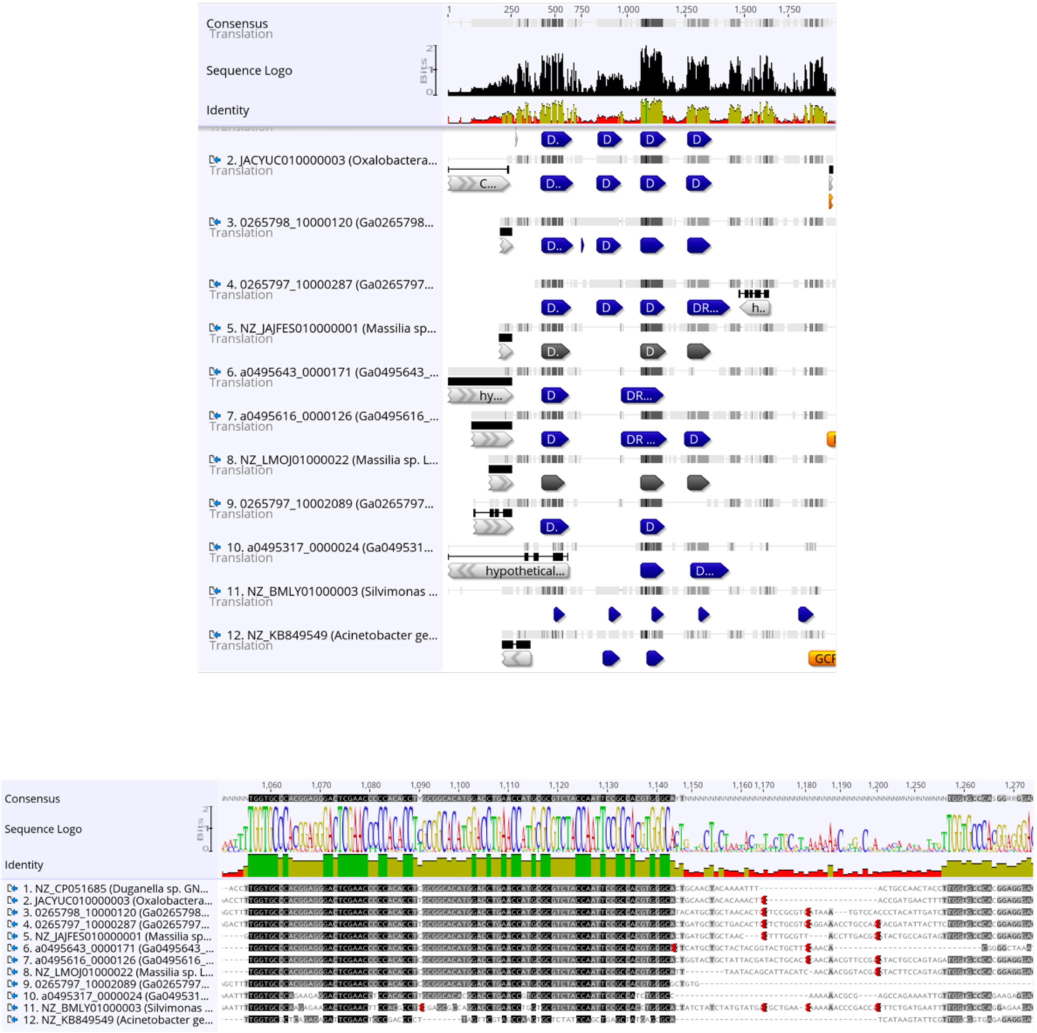

Fig. S21. Alignments of various tRNA associated systems with variable spacer regions.

Alignments of representative tRNA arrays from example identified tRNA array systems with variable spacer regions **(A)** GGDEF tRNA system. **(B)** DEDDh/DNA Pol III epsilon + RNaseH + TIGR03503 tRNA system. **(C)** Ribonuclease R tRNA system.

Table S1. CRISPR association scores of clusters passing filters

Data for main CRISPR association analysis of passing clusters. Columns are as follows:

c30_id:	cluster id
N_cr:	number of CRISPR-associated, non-redundant loci,
N_cr_search:	number of CRISPR-associated, non-redundant loci including DRs found from BLAST search of representative DR for the cluster,
N:	Number of non-redundant loci,
N_eff: function	Effective number of non-redundant loci, as determined by weighting
N_eff_search:	Effective number of non-redundant loci, incorporating DRs found from BLAST search, as determined by weighting function
weighted_icity:	Weighted association score (N_cr / N_eff)
weighted_icity_search:	Weighted association score with DRs found with BLAST (N_cr_search / N_eff_search)
aa_seq:	Amino acid sequence of the representative cluster member

Table S2. Redundant names for Cas proteins

Example equivalences from legacy Cas names to add clarity to the discussion.

Table S3. Pipeline comparisons

Comparison of 5 pipeline variations in their ability to recover key hits found in the study using comparable thresholds.

Table S4. Guide sequences, data and statistical analysis related to genome editing experiments

Sequence, indel, and statistical testing information from the mammalian genome editing experiments.

Table S5. Taxonomic distribution of β-CASP proteins

Distribution of the types of organisms that various β-CASP proteins are found in.

Table S6. List of plasmids used in this study and links to sequences

Information or plasmids used in the experiments.

Data S1. Appearance of CRISPR systems in public data

Files contain the data for appearance of CRISPR systems in public data. Each entry in the list of data contains 3 columns. The first column contains the system identifier. The second column contains the list of p_id's (protein identifiers with the format genome_name && contig_name && start _ end _ strand),, c30_ids (30% cluster ids), and with their date of appearance (time at which 4 non-redundant proteins (90% clusters) found within the 30% cluster appeared in the public data). The third columns contain the list of p_id's (protein identifiers with the format genome_name && contig_name && start _ end _ strand), c90_id's, and and dates of appearance for all non-redundant proteins (90% clusters) within the first 30% cluster to appear in the public data.

Data S2. Genbank files of all redundant loci associated with the manually curated set of hits

Protein IDs list the original contig accession of each cluster, and start, end and strand of the protein of interest are noted for each as in Data S1. Available on Zenodo.

Data S3. Representative proteins from systems identified in this study

Fasta format file of all proteins associated with CRISPR-linked systems identified in this study, using the uas#A, uas#B… nomenclature.

Data S4. Protein-protein association analysis results

Tabular format table with all protein-protein association scores against passing clusters identified in the CRISPR association pipeline. Accession information is encoded in c30_ids as described in Data S1: (genome_name && contig_name && start _ end _ strand) for retrieving protein sequence ids from public data. Available on Zenodo.

Data S5. Spacer analysis for candidate type VII system

All spacer hits and search for the experimentally studied candidate type VII system.

Data S6. Genbank files of all plasmids

All plasmid maps for plasmids used in this study.
